# Classification of *Aspergillus*, *Penicillium*, *Talaromyces* and related genera (*Eurotiales*): An overview of families, genera, subgenera, sections, series and species

**DOI:** 10.1016/j.simyco.2020.05.002

**Published:** 2020-06-27

**Authors:** J. Houbraken, S. Kocsubé, C.M. Visagie, N. Yilmaz, X.-C. Wang, M. Meijer, B. Kraak, V. Hubka, K. Bensch, R.A. Samson, J.C. Frisvad

**Affiliations:** 1 Westerdijk Fungal Biodiversity Institute, Utrecht, The Netherlands; 2 Department of Microbiology, Faculty of Science and Informatics, University of Szeged, Szeged, Hungary; 3 Department of Biochemistry, Genetics and Microbiology, Forestry and Agricultural Biotechnology Institute (FABI), University of Pretoria, P. Bag X20, Hatfield, Pretoria, 0028, South Africa; 4 State Key Laboratory of Mycology, Institute of Microbiology, Chinese Academy of Sciences, No. 3, 1st Beichen West Road, Chaoyang District, Beijing, 100101, China; 5 Department of Botany, Charles University in Prague, Prague, Czech Republic; 6 Department of Biotechnology and Biomedicine Technical University of Denmark, Søltofts Plads, B. 221, Kongens Lyngby, DK 2800, Denmark

**Keywords:** Classification, Infrageneric classification, New taxa, Nomenclature, Phylogeny, Polythetic classes, **Taxonomic novelties: New family**, *Penicillaginaceae* Houbraken, Frisvad & Samson, **New genera**, *Acidotalaromyces* Houbraken, Frisvad & Samson, *Ascospirella* Houbraken, Frisvad & Samson, *Evansstolkia* Houbraken, Frisvad & Samson, *Pseudohamigera* Houbraken, Frisvad & Samson, **New sections**, in *Aspergillus*: sect. *Bispori* S.W. Peterson, Varga, Frisvad, Samson ex Houbraken, sect. *Polypaecilum* Houbraken & Frisvad, sect. *Raperorum* S.W. Peterson, Varga, Frisvad, Samson ex Houbraken, sect. *Silvatici* S.W. Peterson, Varga, Frisvad, Samson ex Houbraken, sect. *Vargarum* Houbraken & Frisvad, in *Penicillium*: sect. *Alfrediorum* Houbraken & Frisvad, sect. *Crypta* Houbraken & Frisvad, sect. *Eremophila* Houbraken & Frisvad, sect. *Formosana* Houbraken & Frisvad, sect. *Griseola* Houbraken & Frisvad, sect. *Inusitata* Houbraken & Frisvad, sect. *Lasseniorum* Houbraken & Frisvad, **New series**, in *Aspergillus*: ser. *Acidohumorum* Houbraken & Frisvad, ser. *Alliacei* Houbraken & Frisvad, ser. *Ambigui* Houbraken & Frisvad, ser. *Arxiorum* Houbraken & Frisvad, ser. *Aurantiobrunnei* Houbraken & Frisvad, ser. *Avenacei* Houbraken & Frisvad, ser. *Bertholletiarum* Houbraken & Frisvad, ser. *Biplani* Houbraken & Frisvad, ser. *Brevipedes* Houbraken & Frisvad, ser. *Brunneouniseriati* Houbraken & Frisvad, ser. *Calidousti* Houbraken & Frisvad, ser. *Canini* Houbraken & Frisvad, ser. *Carbonarii* Houbraken & Frisvad, ser. *Cavernicolarum* Houbraken & Frisvad, ser. *Cervini* Houbraken & Frisvad, ser. *Chevalierorum* Houbraken & Frisvad, ser. *Circumdati* Houbraken & Frisvad, ser. *Conjuncti* Houbraken & Frisvad, ser. *Coremiiformes* Houbraken & Frisvad, ser. *Cremei* Houbraken & Frisvad, ser. *Deflecti* Houbraken & Frisvad, ser. *Egyptiaci* Houbraken & Frisvad, ser. *Fennelliarum* Houbraken & Frisvad, ser. *Flavi* Houbraken & Frisvad, ser. *Flavipedes* Houbraken & Frisvad, ser. *Fumigati* Houbraken & Frisvad, ser. *Funiculosi* Houbraken & Frisvad, ser. *Halophilici* Houbraken & Frisvad, ser. *Heteromorphi* Houbraken & Frisvad, ser. *Homomorphi* Houbraken & Frisvad, ser. *Implicati* Houbraken & Frisvad, ser. *Japonici* Houbraken & Frisvad, ser. *Kalimarum* Houbraken & Frisvad, ser. *Kitamyces* Houbraken & Frisvad, ser. *Leporum* Houbraken & Frisvad, ser. *Leucocarpi* Houbraken & Frisvad, ser. *Monodiorum* Houbraken & Frisvad, ser. *Multicolores* Houbraken & Frisvad, ser. *Neoglabri* Houbraken & Frisvad, ser. *Neonivei* Houbraken & Frisvad, ser. *Nidulantes* Houbraken & Frisvad, ser. *Nigri* Houbraken & Frisvad, ser. *Nivei* Houbraken & Frisvad, ser. *Nomiarum* Houbraken & Frisvad, ser. *Noonimiarum* Houbraken & Frisvad, ser. *Ochraceorosei* Houbraken & Frisvad, ser. *Olivimuriarum* Houbraken & Frisvad, ser. *Penicillioides* Houbraken & Frisvad, ser. *Polypaecilum* Houbraken & Frisvad, ser. *Pulvini* Houbraken & Frisvad, ser. *Restricti* Houbraken & Frisvad, ser. *Rubri* Houbraken & Frisvad, ser. *Salinarum* Houbraken & Frisvad, ser. *Sclerotiorum* Houbraken & Frisvad, ser. *Sparsi* Houbraken & Frisvad, ser. *Spathulati* Houbraken & Frisvad, ser. *Spelaei* Houbraken & Frisvad, ser. *Speluncei* Houbraken & Frisvad, ser. *Stellati* Houbraken & Frisvad, ser. *Steyniorum* Houbraken & Frisvad, ser. *Tamarindosolorum* Houbraken & Frisvad, ser. *Teporium* Houbraken & Frisvad, ser. *Terrei* Houbraken & Frisvad, ser. *Thermomutati* Houbraken & Frisvad, ser. *Unguium* Houbraken & Frisvad, ser. *Unilaterales* Houbraken & Frisvad, ser. *Usti* Houbraken & Frisvad, ser. *Versicolores* Houbraken & Frisvad, ser. *Viridinutantes* Houbraken & Frisvad, ser. *Vitricolarum* Houbraken & Frisvad, ser. *Wentiorum* Houbraken & Frisvad, ser. *Whitfieldiorum* Houbraken & Frisvad, ser. *Xerophili* Houbraken & Frisvad, in *Penicillium*: ser. *Adametziorum* Houbraken & Frisvad, ser. *Angustiporcata* Houbraken & Frisvad, ser. *Atramentosa* Houbraken & Frisvad, ser. *Brevicompacta* Houbraken & Frisvad, ser. *Buchwaldiorum* Houbraken & Frisvad, ser. *Cinnamopurpurea* Houbraken & Frisvad, ser. *Clavigera* Houbraken & Frisvad, ser. *Copticolarum* Houbraken & Frisvad, ser. *Corylophila* Houbraken & Frisvad, ser. *Costaricensia* Houbraken & Frisvad, ser. *Dalearum* Houbraken & Frisvad, ser. *Estinogena* Houbraken & Frisvad, ser. *Euglauca* Houbraken & Frisvad, ser. *Fortuita* Houbraken & Frisvad, ser. *Gallaica* Houbraken & Frisvad, ser. *Georgiensia* Houbraken & Frisvad, ser. *Goetziorum* Houbraken & Frisvad, ser. *Gracilenta* Houbraken & Frisvad, ser. *Herqueorum* Houbraken & Frisvad, ser. *Hoeksiorum* Houbraken & Frisvad, ser. *Idahoensia* Houbraken & Frisvad, ser. *Improvisa* Houbraken & Frisvad, ser. *Indica* Houbraken & Frisvad, ser. *Jiangxiensia* Houbraken & Frisvad, ser. *Kiamaensia* Houbraken & Frisvad, ser. *Livida* Houbraken & Frisvad, ser. *Longicatenata* Houbraken & Frisvad, ser. *Macrosclerotiorum* Houbraken & Frisvad, ser. *Nodula* Houbraken & Frisvad, ser. *Osmophila* Houbraken & Frisvad, ser. *Paradoxa* Houbraken & Frisvad, ser. *Paxillorum* Houbraken & Frisvad, ser. *Phoenicea* Houbraken & Frisvad, ser. *Quercetorum* Houbraken & Frisvad, ser. *Raistrickiorum* Houbraken & Frisvad, ser. *Ramigena* Houbraken & Frisvad, ser. *Robsamsonia* Houbraken & Frisvad, ser. *Rolfsiorum* Houbraken & Frisvad, ser. *Roseopurpurea* Houbraken & Frisvad, ser. *Samsoniorum* Houbraken & Frisvad, ser. *Saturniformia* Houbraken & Frisvad, ser. *Scabrosa* Houbraken & Frisvad, ser. *Sclerotigena* Houbraken & Frisvad, ser. *Sclerotiorum* Houbraken & Frisvad, ser. *Sheariorum* Houbraken & Frisvad, ser. *Simplicissima* Houbraken & Frisvad, ser. *Soppiorum* Houbraken & Frisvad, ser. *Spinulosa* Houbraken & Frisvad, ser. *Sublectatica* Houbraken & Frisvad, ser. *Sumatraensia* Houbraken & Frisvad, ser. *Thiersiorum* Houbraken & Frisvad, ser. *Thomiorum* Houbraken & Frisvad, ser. *Verhageniorum* Houbraken & Frisvad, ser. *Virgata* Houbraken & Frisvad, ser. *Westlingiorum* Houbraken & Frisvad, **New combinations, series**, in *Aspergillus*: ser. *Inflati* (Stolk & Samson) Houbraken & Frisvad, in *Penicillium*: ser. *Alutacea* (Pitt) Houbraken & Frisvad, ser. *Crustacea* (Pitt) Houbraken & Frisvad, ser. *Erubescentia* (Pitt) Houbraken & Frisvad, ser. *Lapidosa* (Pitt) Houbraken & Frisvad, ser. *Pinetorum* (Pitt) Houbraken & Frisvad, series *Tularensia* (Pitt) Houbraken & Frisvad, **New combinations, species**, *Acidotalaromyces lignorum* (Stolk) Houbraken, Frisvad & Samson, *Ascospirella lutea* (Zukal) Houbraken, Frisvad & Samson, *Evansstolkia leycettana* (H.C. Evans & Stolk) Houbraken, Frisvad & Samson, *Hamigera brevicompacta* (H.Z. Kong) Houbraken, Frisvad & Samson, *Paecilomyces lagunculariae* (C. Ram) Houbraken, Frisvad & Samson, *Penicillago kabunica* (Baghd.) Houbraken, Frisvad & Samson, *Penicillago mirabilis* (Beliakova & Milko) Houbraken, Frisvad & Samson, *Penicillago moldavica* (Milko & Beliakova) Houbraken, Frisvad & Samson, *Phialomyces arenicola* (Chalab.) Houbraken, Frisvad & Samson, *Phialomyces humicoloides* (Bills & Heredia) Houbraken, Frisvad & Samson, *Pseudohamigera striata* (Raper & Fennell) Houbraken, Frisvad & Samson, *Talaromyces resinae* (Z.T. Qi & H.Z. Kong) Houbraken & X.C. Wang, *Thermoascus verrucosus* (Samson & Tansey) Houbraken, Frisvad & Samson, **New names**, *Aspergillus chaetosartoryae* Hubka, Kocsubé & Houbraken, *Talaromyces striatoconidius* Houbraken, Frisvad & Samson, *Thermoascus yaguchii* Houbraken, Frisvad & Samson

## Abstract

The *Eurotiales* is a relatively large order of *Ascomycetes* with members frequently having positive and negative impact on human activities. Species within this order gain attention from various research fields such as food, indoor and medical mycology and biotechnology. In this article we give an overview of families and genera present in the *Eurotiales* and introduce an updated subgeneric, sectional and series classification for *Aspergillus* and *Penicillium*. Finally, a comprehensive list of accepted species in the *Eurotiales* is given. The classification of the *Eurotiales* at family and genus level is traditionally based on phenotypic characters, and this classification has since been challenged using sequence-based approaches. Here, we re-evaluated the relationships between families and genera of the *Eurotiales* using a nine-gene sequence dataset. Based on this analysis, the new family *Penicillaginaceae* is introduced and four known families are accepted: *Aspergillaceae*, *Elaphomycetaceae*, *Thermoascaceae* and *Trichocomaceae*. The *Eurotiales* includes 28 genera: 15 genera are accommodated in the *Aspergillaceae* (*Aspergillago*, *Aspergillus*, *Evansstolkia*, *Hamigera*, *Leiothecium*, *Monascus*, *Penicilliopsis*, *Penicillium*, *Phialomyces*, *Pseudohamigera*, *Pseudopenicillium*, *Sclerocleista*, *Warcupiella*, *Xerochrysium* and *Xeromyces*), eight in the *Trichocomaceae* (*Acidotalaromyces*, *Ascospirella*, *Dendrosphaera*, *Rasamsonia*, *Sagenomella*, *Talaromyces*, *Thermomyces*, *Trichocoma*), two in the *Thermoascaceae* (*Paecilomyces*, *Thermoascus*) and one in the *Penicillaginaceae* (*Penicillago*). The classification of the *Elaphomycetaceae* was not part of this study, but according to literature two genera are present in this family (*Elaphomyces* and *Pseudotulostoma*). The use of an infrageneric classification system has a long tradition in *Aspergillus* and *Penicillium*. Most recent taxonomic studies focused on the sectional level, resulting in a well-established sectional classification in these genera. In contrast, a series classification in *Aspergillus* and *Penicillium* is often outdated or lacking, but is still relevant, *e.g.*, the allocation of a species to a series can be highly predictive in what functional characters the species might have and might be useful when using a phenotype-based identification. The majority of the series in *Aspergillus* and *Penicillium* are invalidly described and here we introduce a new series classification. Using a phylogenetic approach, often supported by phenotypic, physiologic and/or extrolite data, *Aspergillus* is subdivided in six subgenera, 27 sections (five new) and 75 series (73 new, one new combination), and *Penicillium* in two subgenera, 32 sections (seven new) and 89 series (57 new, six new combinations). Correct identification of species belonging to the *Eurotiales* is difficult, but crucial, as the species name is the linking pin to information. Lists of accepted species are a helpful aid for researchers to obtain a correct identification using the current taxonomic schemes. In the most recent list from 2014, 339 *Aspergillus*, 354 *Penicillium* and 88 *Talaromyces* species were accepted. These numbers increased significantly, and the current list includes 446 *Aspergillus* (32 % increase), 483 *Penicillium* (36 % increase) and 171 *Talaromyces* (94 % increase) species, showing the large diversity and high interest in these genera. We expanded this list with all genera and species belonging to the *Eurotiales* (except those belonging to *Elaphomycetaceae*). The list includes 1 187 species, distributed over 27 genera, and contains MycoBank numbers, collection numbers of type and ex-type cultures, subgenus, section and series classification data, information on the mode of reproduction, and GenBank accession numbers of ITS, beta-tubulin (*BenA*), calmodulin (*CaM*) and RNA polymerase II second largest subunit (*RPB2*) gene sequences.

## Introduction

The order *Eurotiales* harbours various economically important genera, such as *Aspergillus*, *Penicillium*, *Rasamsonia* and *Talaromyces*. Species classified in this order have diverse properties and include (one of) the most important food spoilage organisms (*e.g.*, *Aspergillus proliferans* (eurotium morph), *Paecilomyces variotii*), mycotoxin producers (*e.g*., *Aspergillus flavus*; aflatoxins), human pathogens (*Aspergillus fumigatus*, *A*. *flavus*, *Talaromyces marneffei*) and indoor contaminants (*e.g*., *Aspergillus versicolor*, *A*. *penicillioides*, *Penicillium chrysogenum*) (Frisvad et al., 2019, Samson et al., 2019, van den Brule et al., 2019, and references therein). Besides their negative impact on human activities, these species are also used in food fermentations (*e.g*., *A*. *oryzae*: soy sauce, miso; *P*. *roqueforti*: blue-veined cheese (Roquefort); *P*. *camemberti*: Camembert cheese), in biotechnology to produce organic acids and enzymes (*e.g*., *Aspergillus niger*: citric acid; *Rasamsonia emersonii*; *Thermomyces lanuginosus*: enzyme production) and for the production of pharmaceuticals (*e.g*., *Aspergillus terreus*: lovastatin; *Penicillium brevicompactum*: mycophenolic acid; *P*. *rubens*: penicillin) (Houbraken et al., 2012a, Houbraken et al., 2014a, Frisvad et al., 2019).

The use of an infrageneric classification system has a long tradition in *Aspergillus* and *Penicillium* (Bainier, 1907, Biourge, 1923, Zaleski, 1927). Thom and his co-workers recognised distinct clusters of species in these genera and named those “groups” or “series” (Thom and Church, 1926, Thom and Raper, 1945, Raper and Thom, 1949, Raper and Fennell, 1965). However, their concept of “groups” does not have any nomenclatural status, and the “series” were wrongly introduced and therefore invalid (Art. 21.1 and 36.1). To avoid confusion and to promote taxonomic stability, a formal infrageneric classification system was needed. Pitt (1980) replaced the “group” structure in *Penicillium* by a subgeneric and sectional structure, and Gams *et al.* (1985) carried out the same changes to *Aspergillus*. The phenotype-based infrageneric classification system proposed in *Aspergillus* was primarily based on conidium colour, conidiophore morphology and growth rates on agar media. This classification system still has a large overlap with the current system that is based on molecular data (Raper and Fennell, 1965, Houbraken and Samson, 2011, Jurjević et al., 2015, Chen et al., 2016a). Pitt (1980) formally introduced an infrageneric classification system for *Penicillium* and its sexual morphs *Talaromyces* and *Eupenicillium* (10 sections, 21 series), and various other systems have been proposed afterwards. For example, Stolk & Samson (1983) introduced a sectional classification system in *Eupenicillium* with four sections, Stolk & Samson (1985) subdivided *Penicillium* into 10 sections and 18 series, and Frisvad & Samson (2004b) recognised five sections and 17 series in subgen. *Penicillium*. These phenotype-based sectional classifications are nowadays replaced by a system based on DNA sequence data (Houbraken and Samson, 2011, Houbraken et al., 2016). In contrast to *Aspergillus*, this DNA-based sectional classification system is often not congruent with the old, phenotype-based systems. Although subgeneric, sectional and/or series classifications have a long tradition in *Aspergillus* and *Penicillium*, they are not widely used in mycology; however, they are (being) implemented for some economically significant and speciose genera such as *Trichoderma*, *Alternaria*, *Hebeloma* and *Talaromyces*. The use of infrageneric ranks for phylogenetic clades is useful for managing large speciose genera, like *Aspergillus*, *Penicillium* and *Talaromyces*. Despite molecular reassessments over the last decade, the classification of *Aspergillus*, *Penicillium* and *Talaromyces* species into subgenera and sections has been rather stable. *Aspergillus* currently includes 25 sections, *Penicillium* 26 sections and *Talaromyces* seven sections. In contrast to the classical monographs on *Aspergillus* and *Penicillium* (Thom and Church, 1926, Thom and Raper, 1945, Raper and Fennell, 1965, Pitt, 1980), a series level classification based on DNA sequence data is lacking in these genera.

Subgenera, sections, subsections, series and subseries are useful categories between genus and species level and are official nomenclatural taxonomic ranks. Well-supported clades discovered by DNA sequence analyses often indicate natural groups of species that can be translated into a subgenus, section or series. Therefore, these ranks can be highly predictive in what functional characters the species might have. For example, *Penicillium* subgen. *Penicillium* sect. *Roquefortorum* ser. *Roquefortorum* is a clear clade based on DNA sequence data (Samson et al., 2004, Houbraken et al., 2010a, Houbraken and Samson, 2011). A character analysis of the species from this series shows that they have many characters in common: all grow well on 0.5 % acetic acid and on creatine sucrose agar, all have large globose conidia, rough-walled conidiophore stipes, all grow at elevated carbon dioxide levels, and all produce roquefortine C, noting that they are also polythetic in the production of other extrolites. *Penicillium carneum*, *P*. *paneum*, *P*. *psychrosexuale* and *P*. *roqueforti* (all members of sect. and ser. *Roquefortorum*) produce different combinations of mycophenolic acids, isofumigaclavines, patulin, botrydiploidin, marcfortines, penipalines, penipacids, penipanoids and eremofortines (Boysen et al., 1996, Frisvad and Samson, 2004b, Frisvad et al., 2004, Nielsen et al., 2006, O'Brien et al., 2006, Houbraken et al., 2010a, Li et al., 2011, Li et al., 2013, Li et al., 2014). Thus, the classification of a species, like the newly described species *P*. *mediterraneum* (Guevara-Suarez *et al.* 2020) to sect. and ser. *Roquefortorum*, is highly predictive in what functional characters they might have.

According to Gould (2000), Linnaeus’ binomial nomenclatural system for the species has survived to this day because the genus reflects the phylogeny and the species epithet reflects the functional phenotype and phylotype: “Linnaeus’s classification scheme can be visualised as a series of nested boxes in which *the species is the irreducible category*” (our italics). We concur, and a consequence of this is that subspecific levels such as subspecies, varieties, *forma specialis etc*. should not be used in taxonomy, at least not in a formal way (see Wilson & Brown 1953). However, some of the more interesting categories are those between the genus and the species: subgenera, sections, subsections, series and subseries (Kirk *et al.* 2008). Do these levels have a nomenclatural status and are they predictive for characters? Should they be formally used in taxonomy and cladonomy?

In this study, the families and genera of the *Eurotiales* were re-evaluated using a nine-gene sequence dataset. New names for lineages representing a new family and four new genera are proposed in the Taxonomy section of this article. The same dataset was used to study the currently defined subgeneric and sectional classification system in *Aspergillus* and *Penicillium*. The relationship within *Aspergillus* and *Penicillium* was studied using a 4-gene sequence dataset and a novel, sequence-based series classification is proposed. Finally, a list of accepted species in the *Eurotiales* (except *Elaphomycetaceae*) is given. This overview updates the *Aspergillus* (Samson *et al.* 2014), *Penicillium* (Visagie *et al.* 2014b) and *Talaromyces* (Yilmaz *et al.* 2014) lists and is expanded with data of other genera and species belonging to the *Eurotiales*.

## Materials and methods

### Strain selection for datasets

The phylogenies presented in this study are based on sequences obtained from the NCBI nucleotide database (GenBank), genome-sequenced strains (GenBank, DOE Joint Genome Institute (JGI)) and sequences newly generated in this study. A selection of strains was made to study the phylogenetic relationships within the *Eurotiales*. The selection aimed to include the current known diversity in the order. In most cases, the types of the species and genera were included. An overview of strains and species is given in Table S1 (Supplementary Information - online only). The phylogenetic relationship of the accepted *Aspergillus* and *Penicillium* species was determined with the aim to introduce a new series classification in those genera. We aimed to include all *Aspergillus* and *Penicillium* species from the list of accepted species (see below) that had tubulin (*BenA*), calmodulin (*CaM*) and/or RNA polymerase II second largest subunit (*RPB2*) sequences. Species belonging to the same subgenus were analysed together in one dataset, resulting in eight datasets (*Aspergillus*, *Circumdati*, *Cremei*, *Fumigati*, *Nidulantes*, *Polypaecilum* (in *Aspergillus*)); *Aspergilloides* and *Penicillium* (in *Penicillium*). Steenwyk *et al.* (2019), using a phylogenomic approach, showed that sect. *Nigri* does not belong to subgen. *Circumdati* and the species belonging to this section were therefore analysed in a separate dataset. Finally, in order to determine the taxonomic position of *Aspergillus texensis* and *Penicillium cellarum*, two separate datasets were constructed and analysed. Publicly available sequences on GenBank were supplemented with newly generated sequences of *A*. *minisclerotigenes* and *P*. *aurantiogriseum* strains (for the *A*. *texensis* and *P*. *cellarum* datasets, respectively) present in the CBS and DTO culture collection housed at the Westerdijk Fungal Biodiversity Institute, Utrecht, the Netherlands.

### DNA isolation, sequencing

Genomic DNA was extracted from cultures grown on malt extract agar (MEA) using the DNeasy® UltraClean® Microbial Kit (Qiagen, Germany) following the manufacturer's instructions. The following primers were used for PCR amplification: T10 (O'Donnell & Cigelnik 1997) or Bt2a (Glass & Donaldson 1995) & Bt2b (Glass & Donaldson 1995) for the partial beta-tubulin (*BenA*) gene region; Cmd5 (Hong *et al.* 2006) or CF1 (Peterson 2008) & Cmd6 (Hong *et al.* 2006) for the calmodulin (*CaM*) gene region; V9G (de Hoog & Gerrits van den Ende 1998) & LS266 (Masclaux *et al.* 1995) for the internal transcribed spacer regions (ITS), including 5.8S nrRNA gene region; LR0R & LR5 (Vilgalys & Sun 1994) for a part of the 28S nrDNA (large subunit rDNA, LSU), and NS1 & NS4 (White *et al.* 1990) for a part of the 18S nrDNA (small subunit rDNA, SSU). Parts of the *Tsr1* (gene coding for a putative ribosome biogenesis protein), *Cct8* (gene coding for the theta subunit of the TCP-1 chaperonin complex) and *RPB1* (RNA polymerase II largest subunit) genes were amplified and sequenced using the methods described previously by Houbraken & Samson (2011). A part of the *RPB2* gene was amplified and sequenced using the primers RPB2-F1 (GCITTYTTCYTIGGITAYATGG) & RPB2-7CR_1 (CATRGCYTGYTTRCCCATIGC). The PCR mixes containing dimethylsulfoxide (DMSO) were ran at an annealing temperature of 48 °C; the mixes containing bovine serum albumine (BSA) at 55 °C. Each of the amplicons was sequenced in both directions using the PCR primers and the BigDye Terminator v. 3.1 Cycle Sequencing Kit (Applied Biosystems, Foster City, CA, USA) following the manufacturer's instructions. Sequencing was performed with an Applied Biosystems™ 3730xl DNA Analyzer (ThermoFisher Scientific, CA, USA). Consensus sequences for each locus were assembled using SeqMan Pro v.15 (DNASTAR). Novel sequences generated in this study were deposited in the GenBank database under accession numbers MN431358–MN431418, MN969061–MN969442, MT024497–MT024529 and MT066177–MT066186.

### Study on phylogenetic relationships above section level

The families and genera of the *Eurotiales* were re-evaluated using a nine-gene sequence dataset and the same dataset was used to study the currently defined subgeneric and sectional classification system in *Aspergillus* and *Penicillium*. The analysis included DNA sequences of nine loci (*BenA*, *CaM*, *Cct8*, ITS, LSU, *RPB1*, *RPB2*, SSU and *Tsr1*) from 263 species belonging to the order *Eurotiales* and 16 species from the order *Onygenales* as outgroup. The dataset was compiled using publicly and newly generated sequences listed in Table S1. Sequences of the *RPB1*, *RPB2*, *Cct8*, SSU and LSU loci were aligned using PRANK v. 140603 (Löytynoja 2014) with the -F option. As *CaM*, *BenA*, *Tsr1* and the ITS loci are difficult to align, a guide tree based on a per-gene partitioned dataset of *RPB1*, *RPB2*, *Cct8*, SSU and LSU sequences was applied with the -F and -prunetree option. The guide tree was inferred by maximum likelihood (ML) using RAxML-NG v. 0.9.0 (Kozlov *et al.* 2019) under the GTR model with gamma-distributed rate heterogeneity. For the final inference, the best fitting model for each locus was determined by ModelTest-NG v. 0.1.4 (Darriba *et al.* 2019) based on the corrected Akaike Information Criterion (Sugiura, 1978, Hurvich and Tsai, 1989) with a maximum likelihood starting tree, set to choose between all models implemented in RAxML-NG with discrete gamma rate categories or FreeRate (Yang 1995) model. For model selection, *CaM* and *BenA* datasets were partitioned to exons and introns, while the ITS dataset was partitioned to ribosomal rDNA and ITS1-ITS2 regions. Alignments of *CaM*, *BenA*, *Tsr1* and ITS datasets contained a high number of indels with important phylogenetic signal (Nagy *et al.* 2012), therefore gaps were recoded as absence/presence characters by 2matrix (Salinas & Little 2014) implementing the simple indel coding algorithm (Simmons & Ochoterena 2000). The four indel matrices were treated as a single partition and added to the concatenated dataset. As indel-based datasets do not contain constant sites, the ascertainment bias correction described by Lewis (2001) was used during the analysis. Branch supports of the best ML tree were estimated by 500 bootstrap replicates.

### Phylogenetic analysis of series relationships within *Aspergillus* and *Penicillium*

Separate phylograms were made of each *Aspergillus* and *Penicillium* subgenus based on a combined dataset of *BenA*, *CaM* and *RPB2* gene sequences. The combined datasets were made from sequences derived from representative *Aspergillus* and *Penicillium* species. An overview of species and their corresponding GenBank accession numbers can be found in the “list of accepted species” below. The separate sequence data sets were aligned using MAFFT v. 7.427 (Katoh & Standley 2013), and subsequently combined with BioEdit v. 7.0.5.3 (Hall 1999) into a three-locus dataset. Phylogenetic analyses were inferred from Maximum Likelihood (ML) and Bayesian inference (BI). Maximum Likelihood analysis was performed using RAxML-HPC2 on XSEDE v. 8.2.12 via the CIPRES Science Gateway (www.phylo.org) with the default GTRCAT model. Bayesian inference analysis was performed with MrBayes v. 3.2.6 (Ronquist *et al.* 2012) using a Markov Chain Monte Carlo (MCMC) algorithm. *Hamigera avellanea* CBS 295.48^T^ and *Penicillium expansum* CBS 325.48^T^ served as outgroup in the *Aspergillus* phylogenies; *H*. *avellanea* CBS 295.48^T^ and *Aspergillus glaucus* CBS 516.65^T^ were the outgroup species in the *Penicillium* phylogenies.

### Extrolites

Secondary metabolite data from literature were used if they were based on reliable identifications. In some cases, isolates of newly described species were analyzed using high performance liquid chromatography with diode array detection (HPLC-DAD) and/or using UHPLC-DAD-MS-MS (Frisvad and Thrane, 1987, Nielsen et al., 2011, Kildgaard et al., 2014, Klitgaard et al., 2014).

## Results and discussion

### Study on phylogenetic relationships above section level

**Fig. 1:**
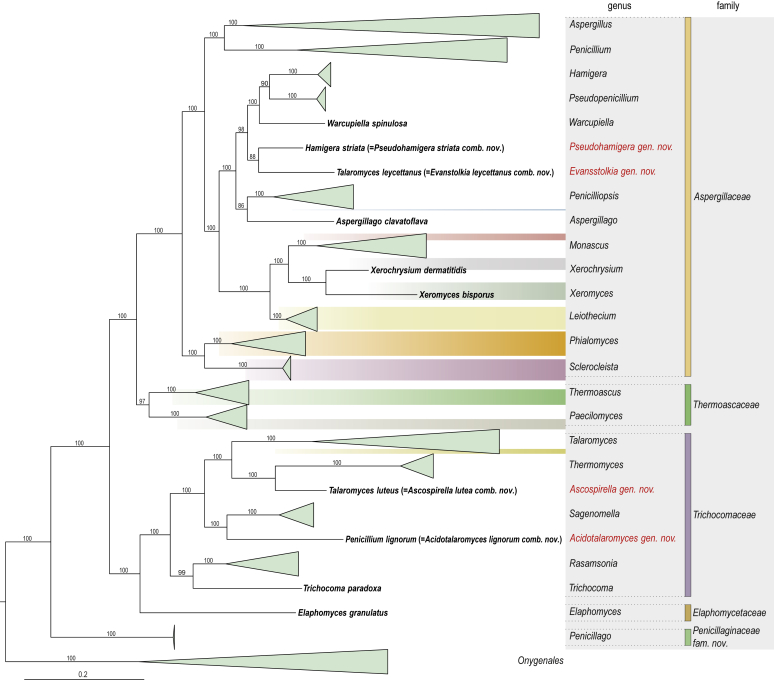
Combined phylogeny using nine loci (*RPB1*, *RPB2*, *Cct8*, *Tsr1*, *CaM*, *BenA*, SSU, LSU, ITS). Clades in the phylogram are collapsed showing the relationship between genera and families in the *Eurotiales*. The phylogram is based on 263 species belonging to the order *Eurotiales* and 16 species from the order *Onygenales* (used an outgroup). The species used in the analysis can be found in Supplementary Fig. S1 and Supplementary Table S1.

**Fig. 2:**
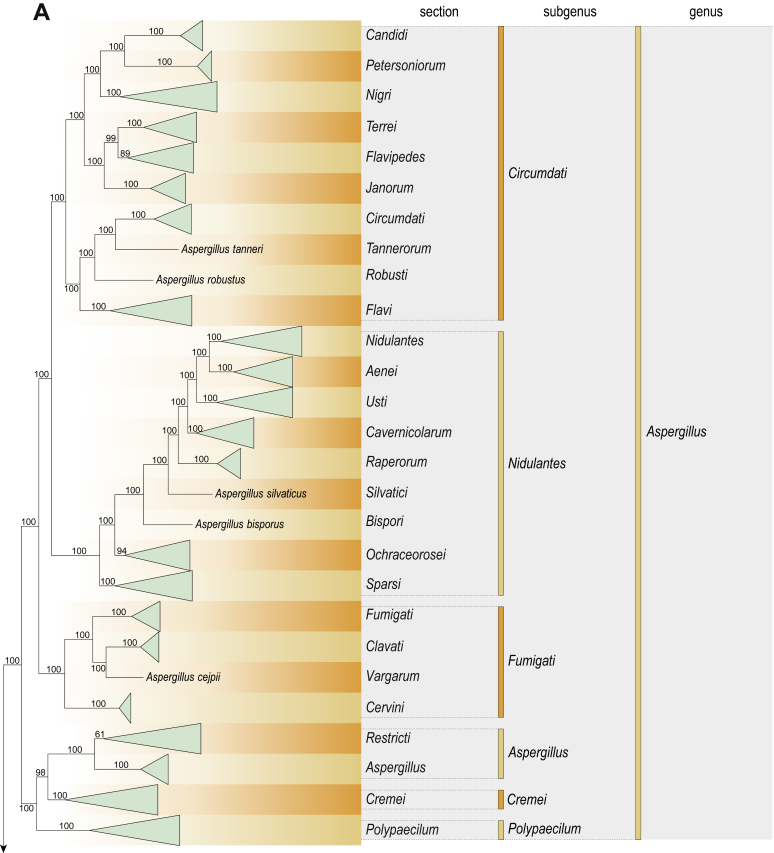

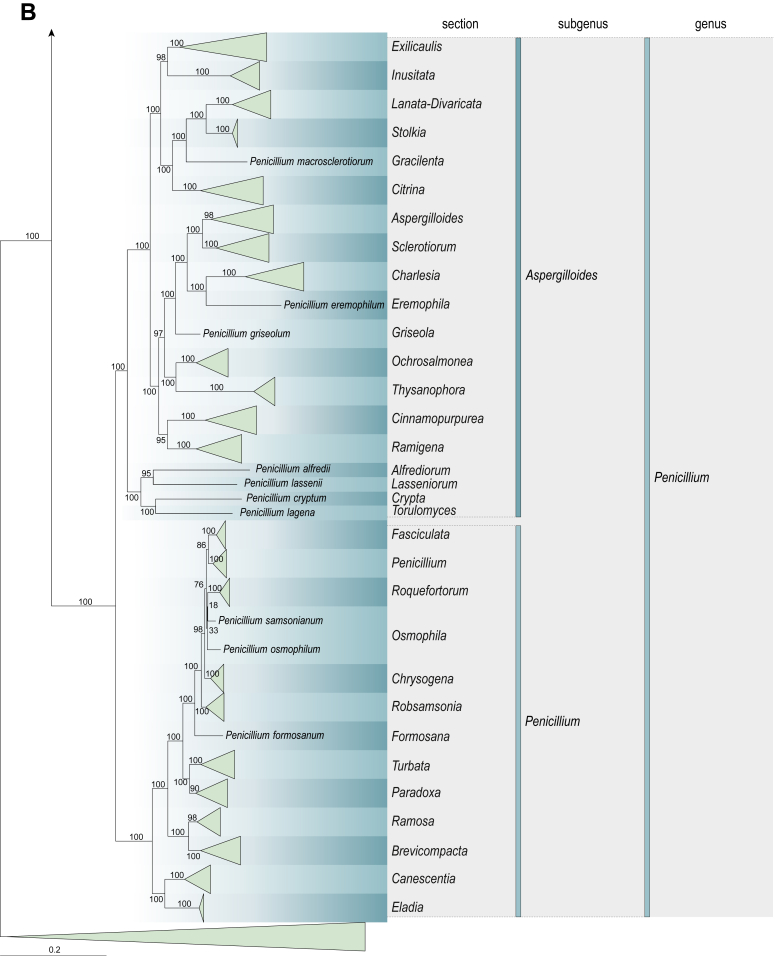
Combined phylogeny using nine loci (*RPB1*, *RPB2*, *Cct8*, *Tsr1*, *CaM*, *BenA*, SSU, LSU, ITS). In the phylogram, only the subgenera and sections of *Aspergillus* and *Penicillium* are shown; the other genera are collapsed as one outgroup clade. The phylogram is based on 263 species belonging to the order *Eurotiales* and 16 species from the order *Onygenales* (used an outgroup). The species used in the analysis can be found in Supplementary Fig. S1 and Supplementary Table S1.

A phylogenetic analysis based on the combined nine-gene dataset was conducted to determine the relationship within the *Eurotiales*. A total of 279 species were included (incl. outgroup species) in the analysis and an overview of the best-fit models for each partition is given in Table 1. The full result of the phylogenetic analysis is shown in Supplementary Fig. S1, and two phylograms with collapsed nodes are shown here (Fig. 1, Fig. 2). Fig. 1 shows the relationship between families and genera within the *Eurotiales*. Five main lineages are present within the order, and those lineages are treated as families: *Aspergillaceae*, *Elaphomycetaceae*, *Thermoascaceae*, *Trichocomaceae*, and an unnamed lineage containing species originally described in *Penicillium* (*P*. *kabunicum*, *P*. *moldavicum*). *Penicillium nodisitatum* also belongs to this lineage (see results below) and this species was recently combined in *Penicillago*. This lineage is therefore named after this genus (*Penicillaginaceae*, see Taxonomy section).

**Table 1:** The best-fit models for each partition proposed by ModelTest-NG based on the corrected Akaike Information Criterion.

Partition	Model
*BenA* exon	TVM+R4+F
*BenA* intron	TIM3+R4+F
*CCT8*	HKY+R4+F
*CaM* exon	TPM1uf+R4+F
*CaM* intron	HKY+R4+F
rDNA of ITS region	TrN+G4+F
ITS1-ITS2	GTR+G4+F
LSU	TIM3+G4+F
*RPB1*	TVM+R4+F
*RPB2*	TPM1uf+R4+F
SSU	TIM2+G4+F
*TSR1*	TrN+R4+F
Indel	BIN+ASC_LEWIS

The accepted, known genera of the *Eurotiales* (*e.g*., Houbraken and Samson, 2011, Kocsubé et al., 2016, Guevara-Suarez et al., 2020) are indicated in Fig. 1. *Hamigera striata* and *Talaromyces leycettanus* form a unique lineage in the *Aspergillaceae* and *Penicillium lignorum* and *Talaromyces luteus* represent independent lineages in the *Trichocomaceae*. *Hamigera striata* is phylogenetically most closely related to *Talaromyces leycettanus* (88 % bootstrap (BS) support) and those two species are sister to a clade including taxa classified in *Hamigera*, *Pseudopenicillium* and *Warcupiella* (98 % BS). *Penicillium lignorum* is sister to a clade containing *Sagenomella* species (100 % BS) and *Talaromyces luteus* is sister to *Thermomyces dupontii* and *Tm*. *lanuginosus* (100 % BS). These four species are combined in new genera below and the relationship with other genera is discussed in the notes (see Taxonomy section).

Fig. 2 shows the relationship between subgenera and sections within *Aspergillus* and *Penicillium*. Six main lineages are present in *Aspergillus*, representing the subgenera in the genus (subgen. *Aspergillus*, *Circumdati*, *Cremei*, *Fumigati*, *Nidulantes* and *Polypaecilum*). Two clades (sections) are present in subgen. *Aspergillus*, ten in subgen. *Circumdati*, one in subgen. *Cremei*, four in subgen. *Fumigati*, nine in subgen. *Nidulantes* and one in subgen. *Polypaecilum*. The phylogenetic relationship within *Aspergillus* is well-resolved and the bootstrap values are generally higher than 95 % (Fig. 2). Exceptions are the nodes of sections *Flavipedes* (89 % BS), *Ochraceorosei* (94 % BS) and *Restricti* (61 % BS). In sect. *Flavipedes*, the relationship of *A*. *neoniveus* with the other taxa of the section is moderately supported, while the relationship of *A*. *penicillioides* (sect. *Restricti*) with other representatives of the section (*A*. *conicus*, *A*. *restrictus*, *A*. *glabripes*, *A*. *halophilicus*) is poorly supported (Supplementary Fig. S1).

Two main, well-supported lineages are present in *Penicillium*, representing subgenera *Aspergilloides* and *Penicillium*. Subgenus *Aspergilloides* was divided into two clades: one clade containing the majority of subgen. *Aspergillioides* taxa, the other including four species: *Penicillium alfredii*, *P*. *cryptum*, *P*. *lagena* and *P*. *lassenii*. New sections for these species are introduced in the Taxonomy section. Nineteen lineages (sections) are present in subgen. *Aspergilloides* and 13 lineages in subgen. *Penicillium*. The majority of branches gained good or full statistical support (>95 % BS) (Fig. 2). The main exception was a clade containing taxa classified in sections *Fasciculata*, *Osmophila*, *Penicillium* and *Roquefortorum*. The statistical support within this clade was generally moderate or poor.

### Phylogenetic analysis of series relationships within *Aspergillus* and *Penicillium*

The phylogenetic relationships among members of *Aspergillus* and *Penicillium* were studied using a combined 3-gene dataset (*BenA*, *CaM*, *RPB2*). The number of included strains and the length of each partition is given in Table 2. The results of the phylogenetic analyses are discussed in the notes in the Taxonomy section.

**Table 2:** Details on the combined datasets used in this study.

Figure number	Dataset	No. species	Length of dataset (bp)		
			*BenA*	*CaM*	*RPB2*
Fig. 6	*Penicillago*	8	467	530	945
Fig. 11	*Aspergillus* subgen. *Aspergillus*	55	458	767	1007
Fig. 12	*Aspergillus* subgen. *Circumdati*	113	693	811	1017
Fig. 13	*Aspergillus* subgen. *Cremei*	19	424	812	1013
Fig. 14	*Aspergillus* subgen. *Fumigati*	76	594	717	1014
Fig. 15	*Aspergillus* subgen. *Nidulantes*	124	456	805	1014
Fig. 16	*Aspergillus* subgen. *Nigri*	32	562	654	1014
Fig. 17	*Aspergillus* subgen. *Polypaecilum*	17	538	642	977
Fig. 18	*Penicillium* subgen. *Aspergilloides*	331	711	922	801
Fig. 19	*Penicillium* subgen. *Penicillium*	145	531	622	978
Fig. 20	*Aspergillus texensis*	35	875	1194	n/a
Supplementary Fig. S2	*Hamigera*	12	488	591	1011
Supplementary Fig. S3	*Talaromyces*	170	633	894	852
Supplementary Fig. S4	*Penicillium cellarum*	45	429	492	767

### Families in *Eurotiales*

The phylogenetic relationship of families and genera belonging to the *Eurotiales* is given in Fig. 1. Five lineages, representing families, were present in our phylogenetic analysis. Based on a 4-gene phylogeny and phenotypic characters, Houbraken & Samson (2011) segregated the *Trichocomaceae* in three families (*Aspergillaceae*, *Thermoascaceae* and *Trichocomaceae*). No representatives of the *Elaphomycetaceae* were included in that study. Based on a phylogenetic analysis of 320 orthologous clusters from selected species, Quandt *et al.* (2015) showed that *Elaphomyces granulatus* (*Elaphomycetaceae*) is a sister to *Trichocomaceae*. This relationship is confirmed in our analysis (Fig. 1, Supplementary Fig. S1). *Elaphomyces* species are ectomycorrhizal (like hypogeous truffles in the *Pezizales*) and produce subglobose, hypogeous 'truffle' fruiting bodies, which have an organised outer layer of tissue (peridium) that enclose the gleba or spore-bearing tissue (Trappe 1979). The position of *Elaphomycetaceae* in the *Eurotiales* is therefore remarkable, and this family represents one of the few independent lineages of the mycorrhizal symbiosis in *Ascomycota* (Tedersoo *et al.* 2010). The uniting character of the *Elaphomycetaceae* with the other families in the *Eurotiales* is the production of cleistothecia, although there are exceptions (*e.g*., *Trichocoma*). Furthermore, a lineage containing *Penicillium kabunicum* and *P*. *moldavicum* is sister to the other families in the *Eurotiales* and is named *Penicillaginaceae* below. This indicates that a penicillium-like conidiophore was the basal morphology in the *Eurotiales* and that this has been lost in *Elaphomycetaceae*. A comparative genome analysis, including the *Penicillaginaceae*, might shed insight into the evolution of the ectomycorrhizal association within the *Eurotiales*. Summarised, our analysis shows that the order *Eurotiales* contains five families: *Aspergillaceae*, *Elaphomycetaceae*, *Penicillaginaceae*, *Thermoascaceae* and *Trichocomaceae*.  

***Aspergillaceae*** Link, Abh. Königl. Akad. Wiss. Berlin: 165. 1826 [1824]. MycoBank MB80489.  

*Type*: *Aspergillus* P. Micheli ex Haller  

*Description*: See Houbraken & Samson (2011) (morphology, phylogeny); Fig. 1, this study (phylogeny).  

***Elaphomycetaceae*** Tul. ex Paol., in Saccardo, Syll. Fung. 8: 863. 1889. MycoBank MB80727.  

*Type*: *Elaphomyces* T. Nees  

*Description*: See Miller & Miller Jr (1984) (morphology), Castellano and Stephens, 2017, Paz et al., 2017 (morphology, phylogeny); Fig. 1, this study (phylogeny).  

***Penicillaginaceae*** Houbraken, Frisvad & Samson, ***fam*. *nov*.** MycoBank MB832568.  

*Etymology*: This family is named after the sole genus in this family, *Penicillago*.  

*Type*: *Penicillago* Guevara-Suarez, Gené & Dania García  

*Diagnosis*: This family is phylogenetically distinct and sister to the families *Aspergillaceae*, *Elaphomycetaceae*, *Thermoascaceae* and *Trichocomaceae* in the order *Eurotiales* (Fig. 1). Conidiophores are penicillium-like and the phialides have a long, narrow neck.  

*Notes*: The newly introduced family *Penicillaginaceae* includes one genus, *Penicillago*. This genus was thought to belong to the *Aspergillaceae*, and was named *Penicillago* (referring to the close phylogenetic relationship with *Penicillium*) (Guevara-Suarez *et al.* 2020). However, this genus forms a unique lineage in the *Eurotiales* and is rather distantly related to *Penicillium* in the *Aspergillaceae* (Fig. 1).  

***Thermoascaceae*** Apinis, Trans. Brit. Mycol. Soc. 50: 581. 1967. MycoBank MB81467.  

*Type*: *Thermoascus* Miehe  

*Description*: See (Apinis 1967) (morphology); Houbraken & Samson (2011) (morphology, phylogeny); Fig. 1, this study (phylogeny).  

***Trichocomaceae*** E. Fisch., in Engler & Prantl, Nat. Pflanzenfam. 1(1): 310. 1897. MycoBank MB81485.  

*Type*: *Trichocoma* Jungh.  

*Description*: See Houbraken & Samson (2011) (morphology, phylogeny); Fig. 1, this study (phylogeny).

### Genera

Based on our phylogenetic analysis (Fig. 1), the *Aspergillaceae* includes 15 genera (*Aspergillago*, *Aspergillus*, *Dichlaena*, *Evansstolkia*, *Hamigera*, *Leiothecium*, *Monascus*, *Penicilliopsis*, *Penicillium*, *Phialomyces*, *Pseudohamigera*, *Pseudopenicillium*, *Sclerocleista*, *Warcupiella*, *Xerochrysium* and *Xeromyces*), the *Thermoascaceae* two (*Paecilomyces*, *Thermoascus*), the *Trichocomaceae* eight (*Acidotalaromyces*, *Ascospirella*, *Dendrosphaera*, *Rasamsonia*, *Sagenomella*, *Talaromyces*, *Thermomyces*, *Trichocoma* and the *Penicillaginaceae* one (*Penicillago*). No material of *Dendrosphaera eberhardtii* (type of genus *Dendrosphaera*) and *Dichlaena lentisci* (type of genus *Dichlaena*) were available for examination. We follow Pitt *et al.* (2000) and tentatively accept these genera. A taxonomic review of the *Elaphomycetaceae* is not part of this study. According to Paz *et al.* (2017), this family includes two genera, *Elaphomyces* and *Pseudotulostoma*; however, the taxonomic status of the latter genus is questionable and molecular data indicate that it is congeneric with *Elaphomyces* (Castellano *et al.* 2016). An overview of genera belonging to the *Eurotiales* is given below.  

***Acidotalaromyces*** Houbraken, Frisvad & Samson, ***gen*. *nov*.** MycoBank MB832551.  

*Etymology*: This species requires a low pH for its growth and it was previously classified (as *Penicillium lignorum*) in *Penicillium* subgen. *Biverticillium*, which contains species that are nowadays mostly classified in *Talaromyces*.  

*Type*: *Penicillium lignorum* Stolk  

**Fig. 3:**
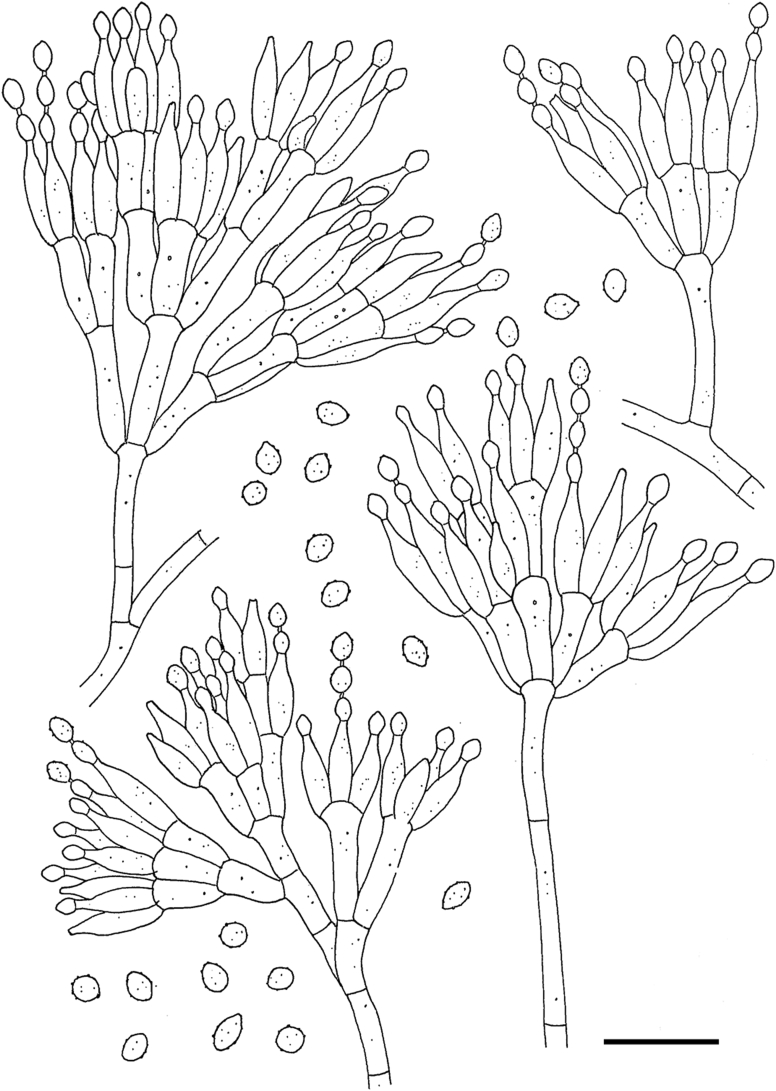
Drawing of *Acidotalaromyces lignorum* CBS 709.68 showing conidiophores and conidia; from Stolk (1969). Scale bar = 10 μm.

*Notes*: *Acidotalaromyces* is a monotypic genus and forms a unique lineage in the *Trichocomaceae*, phylogenetically related to *Sagenomella*. It requires acidified agar media (pH 3.5) for growth, as no or very limited growth occurs on regular media of slightly acidic or neutral pH. *Acidotalaromyces* is known from rotting wood in Europe and potentially produce biotechnologically interesting enzymes.  

*Diagnosis*: Phylogenetically distinct (Fig. 1). Conidiophores talaromyces-like (Fig. 3). No or very slow growth on regular agar media (*e.g*., CYA, MEA) and moderate growth on acidified media (pH 3.5).

***Ascospirella*** Houbraken, Frisvad & Samson, ***gen*. *nov*.** MycoBank MB832552.  

*Etymology*: Named after the typical transverse to spiral ridges on the ascospores of the type species.  

*Type*: *Penicillium luteum* Zukal  

**Fig. 4:**
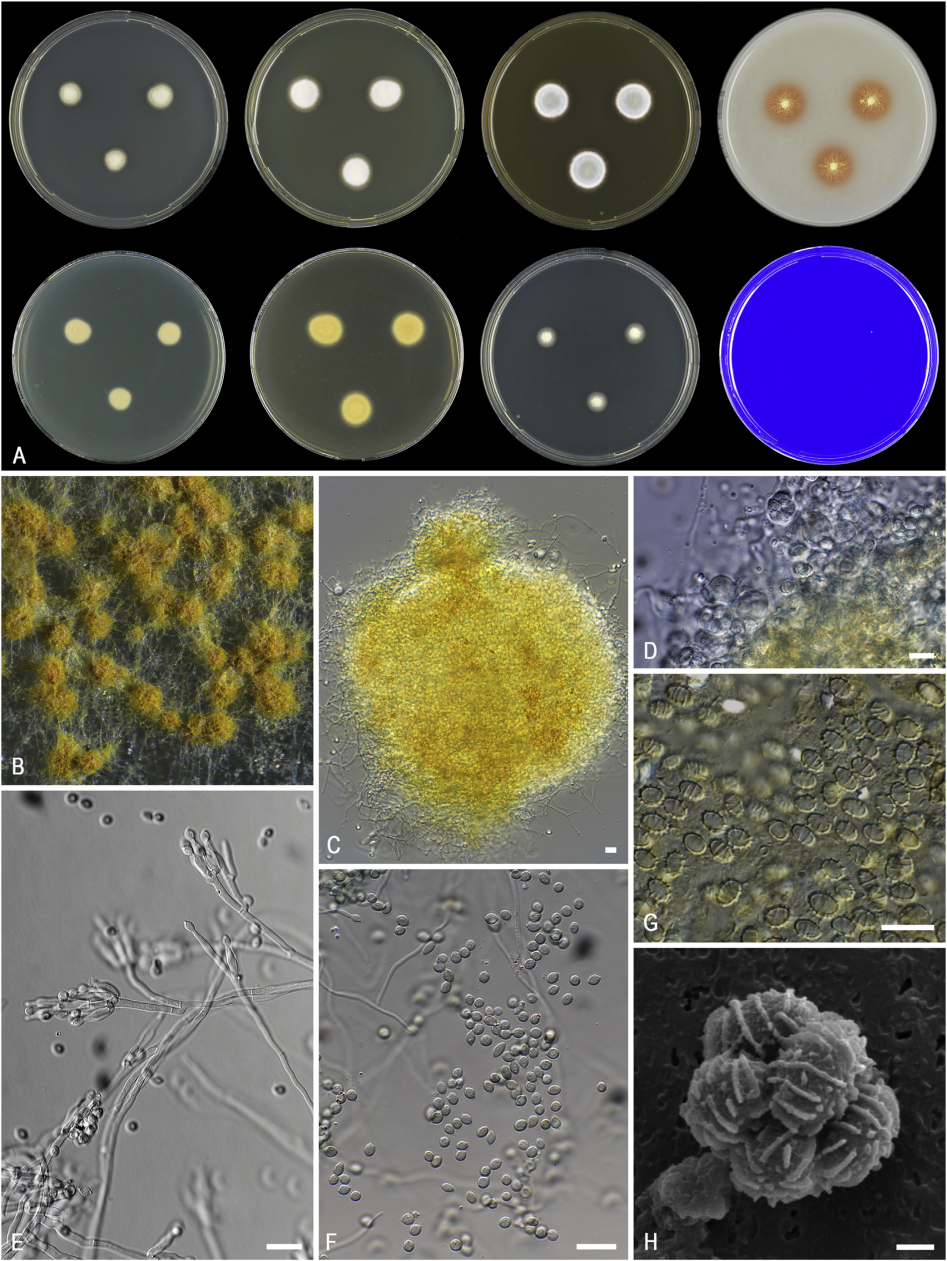
Morphological characters of *Ascospirella lutea*. **A.** Colonies from left to right, after 7 d at 25 °C (top row) CYA, YES, MEA, OA; (bottom row) CYA reverse, YES reverse, DG18, CREA. **B.** Ascomata on OA after 8 wk at 25 °C. **C.** Ascoma. **D.** Asci and ascospores. **E.** Conidiophores. **F.** Conidia. **G.** Ascospores. **H.** SEM micrograph of ascus with ascospores. Scale bars: C–G = 10 μm; H = 1 μm.

*Notes*: *Ascospirella* is a monotypic genus in the *Trichocomaceae* and is phylogenetically most closely related to *Thermomyces*. *Thermomyces* contains thermophilic species (*Tm*. *lanuginosus*, *Tm*. *dupontii*), while the sole member in *Ascospirella* (*i.e*. *Ascospirella lutea*) is a mesophile. *Ascospirella* can be further distinguished from *Thermomyces* by the production of penicillium-like conidiophores and yellow to orange ascomata (Fig. 4). The production of ascospores with conspicuous transverse or spiral ridges or striations is a striking feature for *Ascospirella*. Similarly, ornamented ascospores are also produced in *Trichocoma paradoxa* and *Talaromyces udagawae*. Both species also belong to the *Trichocomaceae*, but are phylogenetically distinct. *Ascospirella* readily produces ascomata on agar media (*e.g*., OA, MEA), while ascoma formation by *Trichocoma* is only observed on the natural substrate. The ascospores of *Ascospirella lutea* resemble *Talaromyces udagawae* and these species were therefore thought to be closely related (Stolk & Samson 1972). These species differ in ascospores size and ornamentation, and ascomatal initials.  

*Diagnosis*: Phylogenetically distinct; conidiophores typically biverticillate, but monoverticillate and irregular forms usually also present; ascomata yellow to orange; ascospores bearing 3–5 conspicuous transverse or spiral ridges or striations (Fig. 4).

***Aspergillago*** Samson *et al.*, Stud. Mycol. 85: 211. 2016. MycoBank MB819186.  

*Type*: *Aspergillago clavatoflava* (Raper & Fennell) Samson *et al.*  

*Notes*: *Aspergillago* was introduced to accommodate *Aspergillus clavatoflavus* (Kocsubé *et al.* 2016). The phylogenetic relationship of *Aspergillago* with other genera was unresolved (Houbraken and Samson, 2011, Kocsubé et al., 2016); however, Fig. 1 shows that this is a sister genus of *Penicilliopsis*.  

***Aspergillus*** P. Micheli ex Haller, Hist. stirp. indig. Helv. inch.: 113. 1768. MycoBank MB7248.  

*Type*: *Aspergillus glaucus* (L.) Link  

*Notes*: The typical conidiophore structure in *Aspergillus* is the aspergillum, with a foot cell, non-septate stipe ending in a vesicle on which the metulae and/or phialides are borne. Houbraken & Samson (2011) demonstrated that the type species of *Polypaecilum* and *Phialosimplex* with a simpler structure are related to members of sections *Cremei* and *Aspergillus*, phylogenetically placing those genera within the classical concept of *Aspergillus*. Furthermore, *Aspergillus paradoxus*, *A*. *malodoratus* and *A*. *crystallinus* characterised by aspergillus-like structures were shown to belong to *Penicillium* sect. *Paradoxa*. With these new taxonomic insights based on phylogenetic relationships, the generic boundaries of *Aspergillus* are now well defined. Samson *et al.* (2014) recommended methods for the identification and characterization of *Aspergillus* creating the basis for a stable taxonomy of the genus.

 The genus contains sexual morphs with different structures. In the dual nomenclature era, these structures were recognised as separate sexual genera; however various studies have demonstrated that they are all within the monophyly of *Aspergillus* (Kocsubé et al., 2016, Steenwyk et al., 2019). The teleomorphic generic name (sexual morphs) are nowadays indicated as morphotypes: eurotium-type, neosartorya-type, emericella-type, petromyces-type, chaetosartorya-type, fennellia-type and neopetromyces-type (Houbraken & Samson 2017). The sexual morph found in sect. *Nigri* can be regarded as the saitoa-type and studies on the genus *Dichlaena* are underway to elucidate the relationship with *Aspergillus*.  

***Dendrosphaera*** Pat., Bull. Soc. Mycol. France 23: 69. 1907. MycoBank MB1455.  

*Type*: *Dendrosphaera eberhardtii* Pat.  

*Notes*: *Dendrosphaera* (Patouillard 1907) is typified with *Dendrosphaera eberhardtii*, the sole species in the genus. The genus is phenotypically related to *Trichocoma* and produces very small brushes of soft hyphae bearing asci and ascospores (Malloch 1985). Kobayasi & Yokoyama (1981) reported that the asexual morph is talaromyces-like (*Penicillium* subgen. *Biverticillium*), similar as in *Trichocoma*. The ascospores of *Dendrosphaera* germinate poorly or not at all on agar media and no cultures or sequences were available for this study (hence not included in our phylogenetic analysis). The exact taxonomic position of this genus needs to be elucidated, but until that time, we follow Pitt *et al.* (2000) and (tentatively) accept it in the *Trichocomaceae*.  

***Dichlaena*** Durieu & Mont., Expl. Sci. l'Algérie 1: 405. 1849. MycoBank MB1514.  

*Type*: *Dichlaena lentisci* Durieu & Mont.  

*Notes*: *Dichlaena lentisci*, the type of the genus, was described in 1849 and found as mature fruiting bodies on decayed leaves. Malloch & Cain (1972) isolated and studied a strain of this species (TRTC 45715), which produces uniseriate *Aspergillus* conidiophores and a close relationship with this genus is therefore likely. No material was available for the current study, but studies are underway to elucidate the relationship between *Dichlaena* and *Aspergillus*. At this moment, it is too premature to combine *D*. *lentisci* in *Aspergillus* and we therefore tentatively retain *Dichlaena*. Three other species are described in *Dichlaena*: *D*. *bovina*, *D*. *indica* and *D*. *pterodontis*. The asexual morph of *D*. *pterodontis* is not aspergillus-like, but a hyphomycete producing conidia in slimy heads (Ram 1971). The classification of this species in *Dichlaena* is doubtful and it is therefore not included in our list of species. *Thielavia bovina* was combined by Booth (1961) in *Dichlaena* as *D*. *bovina*. The original description of this species (Scalia 1900) is insufficient to support the classification in *Dichlaena* and the taxonomic status of this species is therefore doubtful. *Dichlaena indica* was reported to produce an aspergillus asexual morph (Index Fungorum, RecordID 127024) and we therefore tentatively accept this species in *Dichlaena*.  

***Evansstolkia*** Houbraken, Frisvad & Samson, ***gen*. *nov*.** MycoBank MB832553.  

*Etymology*: Named after H.C. Evans and A.C. Stolk, the authors of the sole species in this genus.  

*Type*: *Penicillium leycettanum* H.C. Evans & Stolk  

**Fig. 5:**
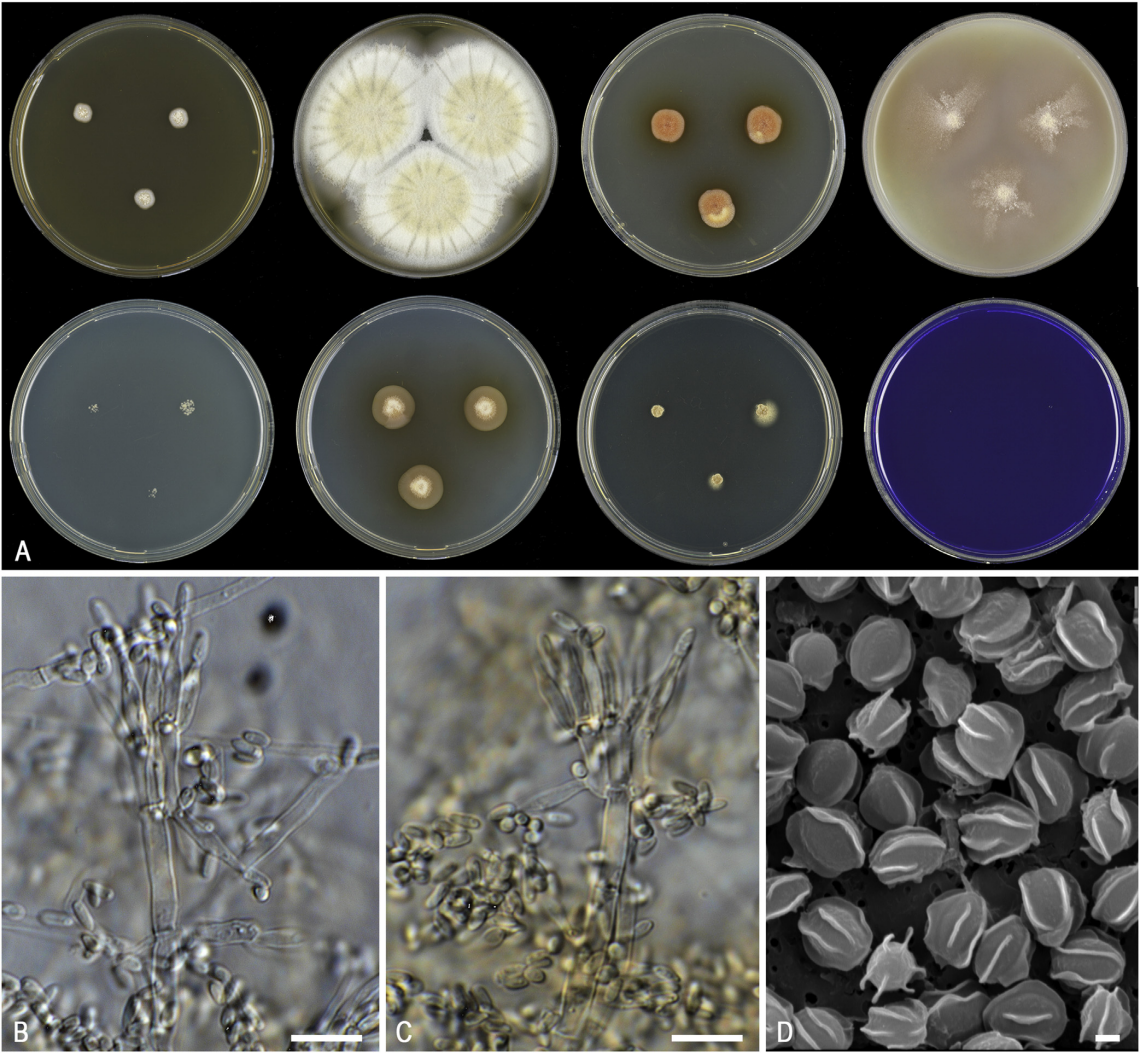
Morphological characters of *Evansstolkia leycettana* (CBS 398.68^T^). **A.** Colonies from left to right, after 7 d at 37 °C unless stated otherwise (top row) MEA 25 °C, MEA, YES, OA; (bottom row) CYA 25 °C, CYA, DG18, CREA. **B–C.** Conidiophores and conidia. **D.** SEM micrograph of ascospores. Scale bars: B–C = 10 μm; D = 1 μm.

*Notes*: *Evansstolkia* forms a single lineage and is a monotypic genus, containing the species *Talaromyces leycettanus* (basionym: *Penicillium* *leycettanum*). *Talaromyces leycettanus* was originally classified in *Talaromyces* because of the production of ascomata that are surrounded by a definite network of pale yellow hyphae and the production of asci in chains. In contrast to these observations, Houbraken & Samson (2011) noted that this species phylogenetically belongs to the *Aspergillaceae*, instead of the *Trichocomaceae*, and this observation is confirmed in this study. *Talaromyces leycettanus* is phylogenetically most closely related to *Hamigera striata* (88 % BS, Fig. 1). This species produces paecilomyces-like conidiophores, brown coloured conidia, thick-walled chlamydospores and ascospores that are decorated with somewhat jagged, irregular, mostly longitudinal ridges of different length. Furthermore, *Tal*. *leycettanus* is thermotolerant to thermophilic. This combination of characters is unique in the *Aspergillaceae* and we therefore decided to accommodate this species in a novel genus.  

*Diagnosis*: Phylogenetically distinct. Conidiophores paecilomyces-like; conidia brown; chlamydospores present, thick-walled; ascospores decorated with somewhat jagged, irregular, mostly longitudinal ridges of different length. Thermotolerant to thermophilic. Fig. 5.

***Hamigera*** Stolk & Samson, Persoonia 6: 342. 1971. MycoBank MB2215.  

*Type*: *Hamigera avellanea* (Thom & Turesson) Stolk & Samson  

*Notes*: The taxonomy of *Hamigera* and related genera has been subject of various studies. The genus *Hamigera* was erected for two *Talaromyces* species (*Tal*. *avellaneus*, *Tal*. *striatus*) that produce asci singly instead of in chains (Stolk & Samson 1971). Later, the genus *Merimbla* was introduced for the asexual morph of *H*. *avellanea* (Pitt 1979). von Arx (1986) transferred *Warcupiella* to *Hamigera* and treated *Merimbla* and *Raperia* as congeneric, giving priority to the latter. Peterson *et al.* (2010) revised the genus *Hamigera* using a multigene sequence-based approach and accepted seven species. They showed that *Warcupiella* (and the related asexual genus *Raperia*) and *Hamigera striata* (here combined to *Pseudohamigera*) do not belong to this genus. *Merimbla* and *Hamigera* resided in the same lineage, and after the introduction of a single name nomenclature, *Merimbla ingelheimensis* was transferred to *Hamigera ingelheimensis* (Igarashi *et al.* 2014). *Talaromyces brevicompactus* (asexual morph *Merimbla brevicompacta*, simultaneously published, identical holotype) is phenotypically similar to *Hamigera avellanea* (reported as *Tal*. *avellaneus*), but differs in their initials, ascospore ornamentation and conidiophore branching (Kong 1999). Samson *et al.* (2011c) indicated that *Talaromyces brevicompactus* represents a distinct species in *Hamigera*. The new combination, *Hamigera brevicompacta*, is made below. *Hamigera avellanea* var. *alba* is phylogenetically related to *H*. *pallida* (Supplementary Fig. S2), but the taxonomic status of both species needs further study. In total, nine species are currently accepted: *Hamigera avellanea*, *H*. *brevicompacta*, *H*. *fusca*, *H*. *inflata*, *H*. *ingelheimensis*, *H*. *insecticola*, *H*. *pallida*, *H*. *paravellanea* and *H*. *terricola*.  

***Leiothecium*** Samson & Mouch., Canad. J. Bot. 53: 1634. 1975. MycoBank MB2719.  

*Type*: *Leiothecium ellipsoideum* Samson & Mouch.  

*Notes*: *Leiothecium* is characterised by dark, glabrous ascomata with thin pseudoparenchymatous walls and by ellipsoidal, reticulate, hyaline ascospores (Samson & Mouchacca 1975a). *Leiothecium* is phylogenetically a sister of a cluster containing the genera *Monascus*, *Xerochrysium* and *Xeromyces* (Fig. 1). *Monascus* is also phenotypically related to *Leiothecium*, but differs in producing a thin, plectenchymatous ascomatal wall and smooth-walled ascospores (Samson and Mouchacca, 1975a, Barbosa et al., 2017). Furthermore, *Monascus* produces a basipetospora-type asexual morph, while an asexual morph is not observed in *Leiothecium*. *Leiothecium* also shows some similarity with *Ascorhiza* (considered a doubtful taxon below) and *Hapsidospora* (*Hypocreales*) (Samson & Mouchacca 1975a) because of the presence of cleistothecial ascomata and reticulate ascospores (Marin-Felix *et al.* 2014). The genus includes two species, *Leiothecium ellipsoideum* and *L*. *cristatum*.  

***Monascus*** Tiegh., Bull. Soc. Bot. France 31: 226. 1884. MycoBank MB3247.  

*Type*: *Monascus ruber* Tiegh.  

*Notes*: The genus *Monascus* was described by van Tieghem (1884) to accommodate the sexually reproducing species *M*. *ruber* and *M*. *mucoroides*. The genus is characterised by the production of colourless to light brown cleistothecia, in some species becoming dark brown in time, and smooth-walled ascospores. In the abandoned dual name nomenclature system, the genus *Basipetospora* was found to be the asexual morph of *Monascus* and was characterised by the production of aleurioconidia in a basipetal manner from undifferentiated conidiogenous cells that progressively shorten (retrogression, Cole & Samson 1979). The name *Monascus* was recommended over *Basipetospora* (Rossman *et al.* 2016). *Monascus* is phylogenetically sister to a clade containing the xerophilic genera *Xerochrysium* and *Xeromyces* (Fig. 1). Barbosa *et al.* (2017) conducted a phylogenetic analysis of this genus and resolved *Monascus* in nine species (*Monascus argentinensis*, *M*. *flavipigmentosus*, *M*. *floridanus*, *M*. *lunisporas*, *M*. *mellicola*, *M*. *pallens*, *M*. *purpureus*, *M*. *recifensis* and *M*. *ruber*) and two sections (sections *Floridani* and *Rubri*).  

***Paecilomyces*** Bainier, Bull. Soc. Mycol. France 23: 27. 1907. MycoBank MB9196.  

*Type*: *Paecilomyces variotii* Bainier  

*Notes*: Phylogenetic analysis of the 18S rDNA demonstrated that *Paecilomyces sensu*Samson (1974) is polyphyletic across two subclasses, *Sordariomycetidae* and *Eurotiomycetidae*. The type species of this genus, *Paecilomyces variotii*, and its thermophilic relatives belong in the *Eurotiales* (Luangsa-Ard *et al.* 2004). The ascomycete genus *Byssochlamys* is linked to *Paecilomyces sensu stricto*. The taxonomy of these genera was studied by Samson *et al.* (2009) and five *Byssochlamys* and four *Paecilomyces* species were accepted. One of their accepted species was *Paec*. *saturatus*. This species was based on the variety name *Paecilomyces mandshuricus* var. *saturatus*, while species names were also available (*Penicillium viniferum*, *Paec*. *dactylethromorphus*). *Penicillium viniferum* (1939) would have priority, but was invalidly described (without Latin diagnosis), and the correct name for *Paec*. *saturatus* is therefore *Paec*. *dactylethromorphus*. With the introduction of a single name nomenclature system, *Paecilomyces* got priority over *Byssochlamys* (Rossman *et al.* 2016). *Paecilomyces* is nowadays characterised by producing irregularly branched conidiophores bearing phialides with an inflated base and abruptly narrowing to a thin neck and producing olive-brown conidia in chains. They are thermotolerant and some species are able to produce a byssochlamys sexual morph and smooth-walled ellipsoidal ascospores. *Rasamsonia* is phenotypically most closely related and differs in having more regularly branched conidiophores with distinctly rough-walled structures. *Penicillium* and *Talaromyces* are phenotypically similar genera, but generally produce more regularly branched conidiophores, flask-shaped (*Penicillium*, *Talaromyces*) or lanceolate (*Talaromyces*) phialides and conidia in shades of green. *Paecilomyces* names are available for most *Byssochlamys* species, except *Byssochlamys lagunculariae*, and a new combination is proposed below (*Paec*. *lagunculariae*). After the taxonomic treatment of Samson *et al.* (2009), only one new species was described in *Paecilomyces*, *Paec*. *tabacinus* (Crous *et al.* 2016). The genus currently includes 10 species: *Paecilomyces brunneolus*, *Paec*. *formosus*, *Paec*. *lagunculariae*, *Paec*. *dactylethromorphus*, *Paec*. *divaricatus*, *Paec*. *fulvus*, *Paec*. *niveus*, *Paec*. *tabacinus*, *Paec*. *variotii* and *Paec*. *zollerniae*. *Paecilomyces formosus* is invalid, because it was based on the invalidly described species *Monilia formosa* (*nom*. *inval*., Art. 36.1). *Paecilomyces maximus* might be the correct name to use; however, we wait with this taxonomic change because this species (and therefore also *Paec*. *formosus*) might be a complex of at least three species.  

***Penicillago*** Guevara-Suarez *et al.*, Fungal Syst. Evol. 5: 64. 2020. MycoBank MB822073.  

*Type*: *Penicillago nodositata* (Valla) Guevara-Suarez *et al.*  

**Fig. 6:**
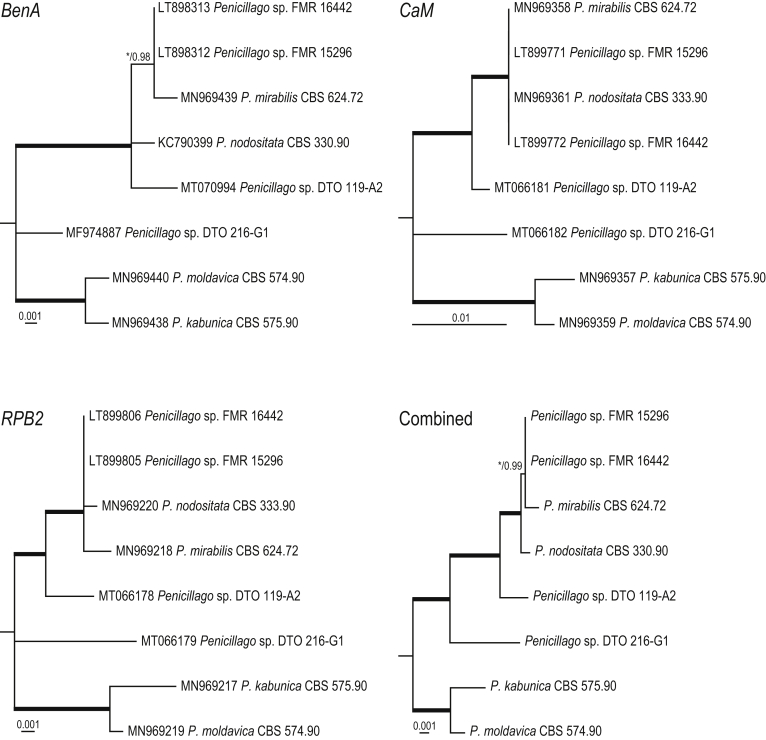
Phylogenetic trees based on single and a combined data set of *BenA*, *CaM* and *RPB2* sequences showing the relationship between *Penicillago* species. The BI posterior probability (pp) values and bootstrap percentages of the maximum likelihood (ML) analysis are presented at the nodes; fully supported branches are thickened. Values less than 70 % bootstrap support (ML) or less than 0.95 posterior probability (Bayesian analysis) are indicated with a hyphen or not shown. The bar indicates the number of substitutions per site.

**Fig. 7:**
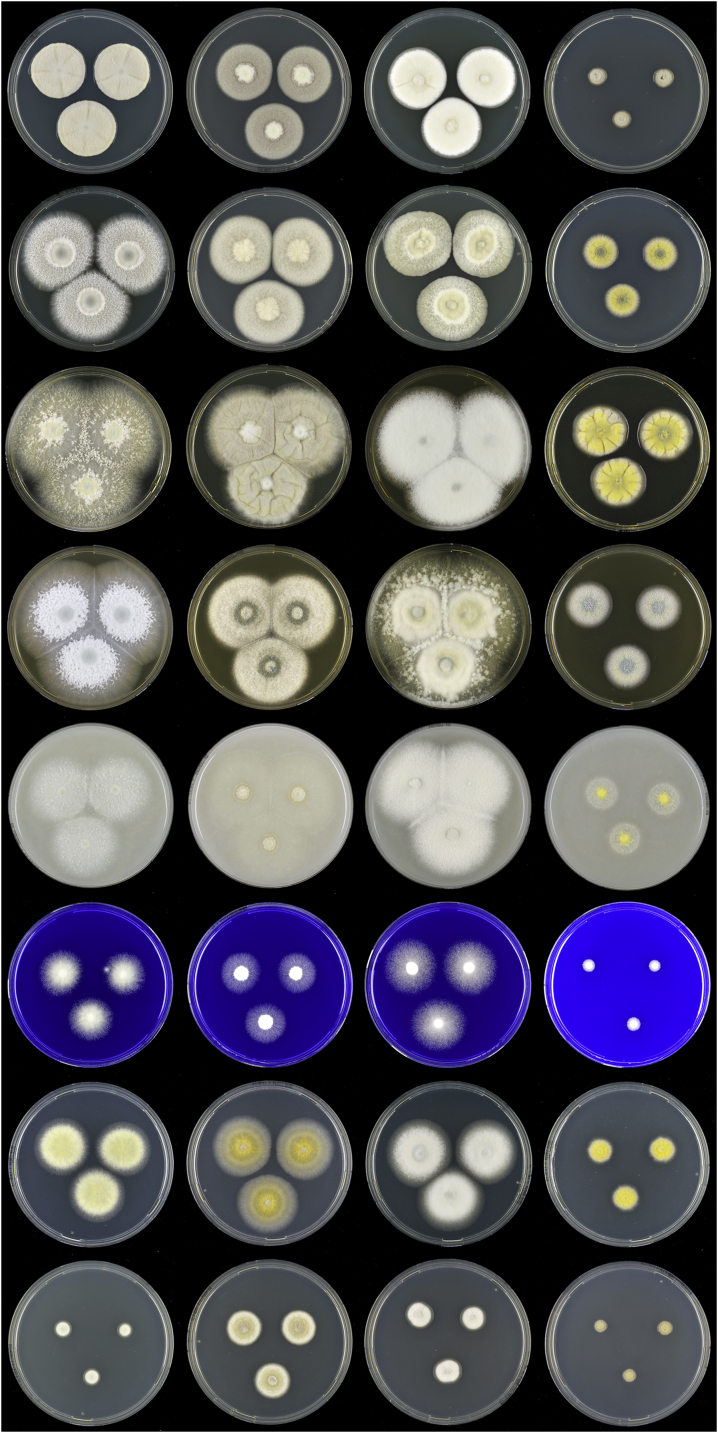
Overview of macromorphology of species classified in *Penicillago* (*Penicillaginaceae*), 7-d-old cultures at 25 °C (unless mentioned otherwise). Columns, left to right: *P*. *kabunica* CBS 575.90, *P*. *mirabilis* CBS 624.72, *P*. *moldavica* CBS 574.90, *P*. *nodositata* CBS 333.90; rows, top to bottom: CYA 30 °C, CYA, YES, MEA, OA, CREA, DG18, CYAS.

**Fig. 8:**
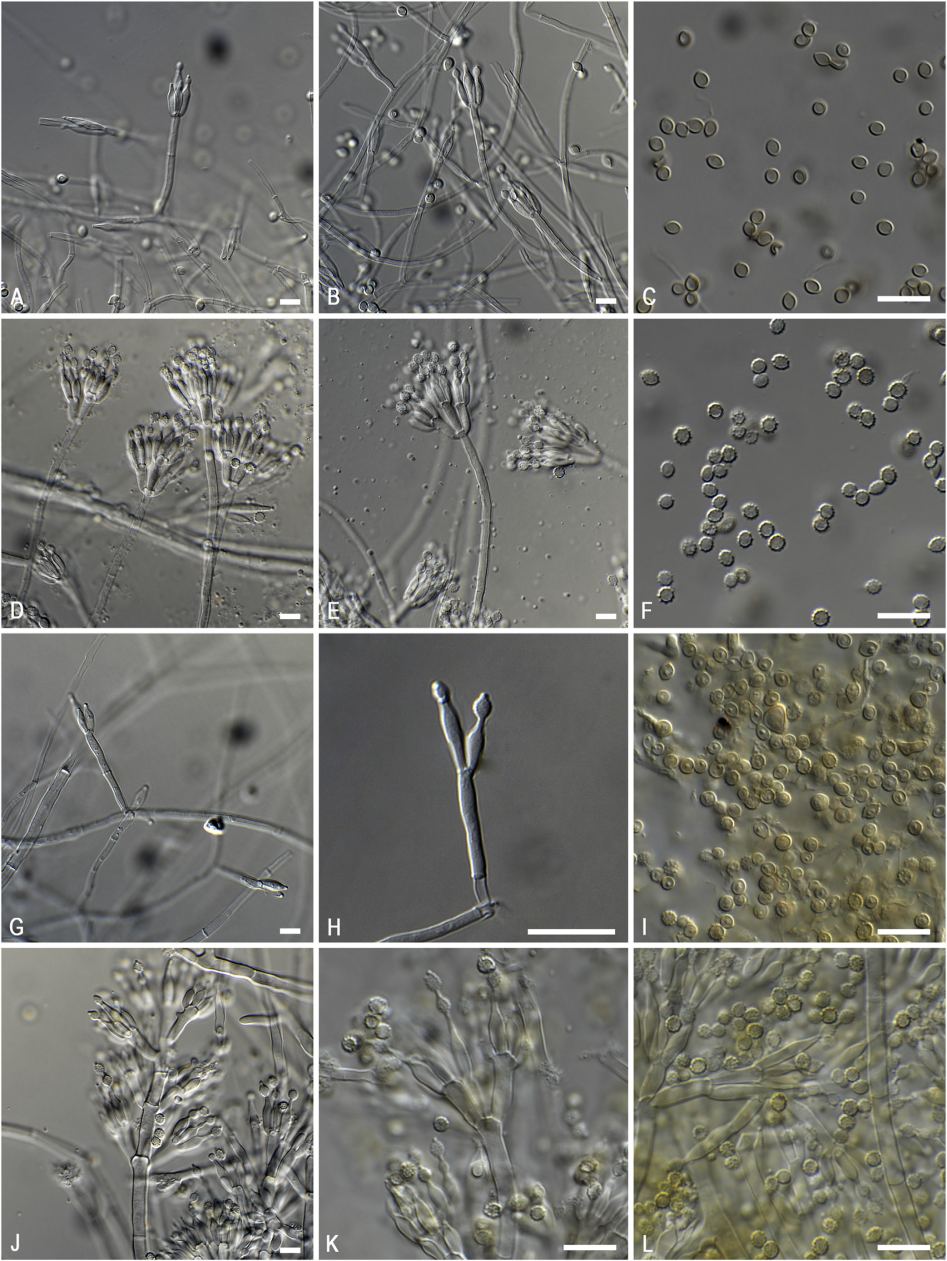
Overview of micromorphology of species classified in *Penicillago* (*Penicillaginaceae*). **A–C.***P*. *kabunica* CBS 575.90, conidiophores and conidia. **D–F.***P*. *mirabilis* CBS 624.72, conidiophores and conidia. **G–I.***P*. *moldavica* CBS 574.90, conidiophores and conidia. **J–L.***P*. *nodositata* CBS 333.90, conidiophores and conidia. Scale bar = 10 μm.

*Notes*: The phylogenetic analysis (Fig. 6) shows that four species can be recognised in *Penicillago*. These species were all originally described in *Penicillium* (*P*. *kabunicum*, *P*. *mirabile*, *P*. *moldavicum* and *P*. *nodositatum*). Only *Penicillium nodositatum* was recently combined to *Penicillago* (Guevara-Suarez *et al.* 2020) as *Penicillago nodositata*. The others are transferred to *Penicillago* below. Morphologically, these species seem unrelated and the most striking unifying character is the production of ampulliform phialides that have a relatively long, narrowed neck. They also share the production of penicillium-like conidiophores, produce conidia in green shades, are unable to grow on CYA incubated at 37 °C and have moderate growth on CREA without acid production. A comparison of the macro- and micromorphology of these species is shown in Fig. 7, Fig. 8 and summarised in Table 3. *Penicillago nodositata* is macroscopically distinct. This species produces relatively smaller and more colourful colonies than the other three members of the genus. *Penicillago mirabile* (= *Penicillium mirabile*) grows better on CYAS than the other species in the genus. *Penicillago kabunica* (= *Penicillium kabunicum*) and *P*. *moldavica* (= *Penicillium moldavicum*) are phylogenetically closely related and can be differentiated on their conidium ornamentation (Ramírez 1982). The former produces smooth-walled conidia, and the conidia of the latter are echinulate.

**Table 3:** Overview of colony diameters on various agar media and microscopic characters of *Penicillago* species.

Species	Colony diameter, 7 d, in mm								Conidiophore branching^1^	Shape conidia	Ornamentation conidia	Shape phialide
	CYA	CYAS	YES	MEA	CREA	DG18	CYA30°C	CYA37°C				
*P. kabunica*	36–40	8–12	>60	>60	26–30	30–34	30–36	No growth	Mono- or biverticillate, divaricate	Globose to subglobose, with a pointed end	Smooth walled	Ampulliform, with a long, narrowed neck
*P. mirabilis*	38–42	18–22	51–59	47–53	23–27	34–38	31–35	No growth	Mono- or biverticillate, occasional monophialidic	Globose to subglobose	Echinulate	Ampulliform, with a conspicuously long, narrowed neck
*P. nodositata*	19–23	6–9	29–33	26–30	6–8	15–19	8–12	No growth	Predominantly biverticillate	Subglobose to ellipsoidal	Echinulate	Ampulliform, with a conspicuously long, narrowed neck
*P. moldavica*	32–36	16–19	46–52	60–70	15–19	17–21	23–27	No growth	Mono- or biverticillate, divaricate	Globose to subglobose	Echinulate	Ampulliform, with a long, narrowed neck

1 The examined *P. kabunica* and *P. moldavica* strains were degenerated and part of presented data are also based on Ramirez (1982).

***Penicilliopsis*** Solms, Ann. Jard. Bot. Buitenzorg 6: 53. 1887. MycoBank MB3806.  

*Type*: *Penicilliopsis clavariiformis* Solms  

*Notes*: *Penicilliopsis* was described by Solms-Laubach (1887) on seeds of *Diospyros macrophylla* collected in the Botanical Garden of Bogor in Indonesia. This genus was originally introduced for sexually reproducing species and the genera *Sarophorum*, *Stilbothamnium*, *Stilbodendron* and *Pseudocordyceps* are associated asexual genera. The aforementioned genera were critically re-examined using morphological characters by Samson & Seifert (1985), but this group of fungi is to date not yet studied using molecular techniques. The type of *Sarophorum*, *S*. *ledermannii*, is considered to be conspecific with *S*. *palmicola*, and the latter species is regarded to be the asexual morph of *Penicilliopsis clavariiformis* (Samson & Seifert 1985). In a single name nomenclature system, *Penicilliopsis* (1887) will have priority over *Sarophorum* (1916). We therefore consider *Sarophorum* a synonym of *Penicilliopsis*, and *Sarophorum ledermannii* and *S*. *palmicola* synonyms of *Penicilliopsis clavariiformis*. *Stilbodendron* is typified with *S*. *camerunense* (= *S*. *cervinum*), and this species is referred to as the asexual morph of *Penicilliopsis africana*. Based on these data, *Stilbodendron* is also considered a synonym of *Penicilliopsis*. Furthermore, *Stilbodendron camerunense*, *S*. *congoense* and *S*. *cervinum* are conspecific (Samson & Seifert 1985) and treated there as synonyms of *Penicilliopsis africana*. *Stilbothamnium* (type *S*. *togoensis* (= *Aspergillus togoensis*)) was also thought to be related to *Penicilliopsis*, but *Stilbothamnium* is currently considered a synonym of *Aspergillus* (Samson and Seifert, 1985, Samson et al., 2014, Frisvad et al., 2019). Hsieh & Ju (2002) studied the taxonomic position of *Pseudocordyceps* and the sole species in this genus, *Pseudocordyceps seminicola*. Cultures obtained from ascospores of the undescribed *Penicilliopsis* yielded a culture of *Pseudocordyceps seminicola*. Based on this observation, the name *Penicilliopsis pseudocordyceps* was introduced for the sexual morph of *Pseudocordyceps seminicola*. *Penicilliopsis*, described in the year 1887, has priority over *Pseudocoryceps* (described in 1936).

 No (ex-)type material of *Penicilliopsis africana* and *P*. *clavariiformis* was included in our study, but CBS 257.33, a representative strain of *Penicilliopsis clavariiformis* (Samson & Seifert 1985), was used in our phylogenetic study (Fig. 1). *Aspergillus zonatus* clustered most closely with *P*. *clavariiformis* (Kocsubé *et al.* 2016), and this species was therefore combined in *Penicilliopsis*. Following Samson and Seifert, 1985, Hsieh and Ju, 2002 and Kocsubé *et al.* (2016), we include *Penicilliopsis africana*, *P*. *clavariiformis*, *P*. *pseudocordyceps*, and *P*. *zonata* in the species list below.  

***Penicillium*** Link, Mag. Ges. Naturf. Freunde Berlin 3: 16. 1809. MycoBank MB9257.  

*Type*: *Penicillium expansum* Link  

*Notes*: The generic concept of *Penicillium* is now well-defined following studies of Houbraken & Samson (2011) and Visagie *et al.* (2014b). The main conidiophore structure is the penicillus which can be mono- (simple), bi-, ter- or quarterverticillate. Houbraken & Samson (2011) synonymised the genera *Torulomyces* and *Thysanophora* with *Penicillium*. The sexual morphs which were formerly known as *Eupenicillium*, *Chromocleista* and *Hemicarpenteles* are regarded as synonyms on the basis of the single nomenclature. Visagie *et al.* (2014b) recommended methods for the identification and characterization of *Penicillium* creating the basis for a stable taxonomy of the genus.  

***Phialomyces*** P.C. Misra & P.H.B. Talbot, Canad. J. Bot. 42: 1287. 1964. MycoBank MB9341.  

*Type*: *Phialomyces macrosporus* P.C. Misra & P.H.B. Talbot  

*Notes*: The genus *Phialomyces* was described for species that produce large, dark, warted conidia on phialides whose apices are neither prolonged nor divergent (Misra & Talbot 1964). This genus phenotypically resembles *Paecilomyces*; however, species in this genus produce generally smaller, hyaline or slightly pigmented conidia. Furthermore, the phialides of *Paecilomyces* have a broad base and a long narrow neck. Based on a 4-gene phylogeny, Houbraken & Samson (2011) classified *Phialomyces* in the *Aspergillaceae*, with *Sclerocleista* being its most closely related sister. This relationship is confirmed in our multigene phylogenetic analysis (Fig. 1). Five species were described in this genus: *Phialomyces fusiformis* (Rodriguez & Decock 2003), *Ph*. *macrosporus* (Misra & Talbot 1964), *Ph*. *microsporus* (Zhang *et al.* 2010), *Ph*. *striatus* (Castañeda & Gams 1991) and *Ph*. *taiwanensis* (Matsushima 1985).

 *Phialomyces striatus* was, after *Ph*. *macrosporus*, the second species that was described in this genus. Sequences derived from the ex-type strain of this species (CBS 550.89^T^) indicate a relationship with taxa in *Talaromyces* sect. *Talaromyces*. A new name for this species is given below (as *Talaromyces striatoconidius*). The taxonomic position of *Ph*. *taiwanensis* is uncertain. Mercado-Sierra *et al.* (1998) transferred this species to *Thysanophora*. Later, *Thysanophora* was synonymised with *Penicillium* and therefore Houbraken & Samson (2011) renamed this species *Penicillium taiwanense*. Until new information becomes available, we follow Houbraken & Samson (2011) and retain this species in *Penicillium*. The type culture of *Ph*. *fusiformis* (MUCL 43747^T^) was not included in our study and no sequence data is present in public databases. Based on the description, this species is phenotypically similar to *Ph*. *macrosporus*, the generic type, and we therefore accept this species in the genus. In 2010, a small-spored *Phialomyces* species was described as *Ph*. *microsporus* (Zhang *et al.* 2010). Sequences obtained from the ex-type strain (DTO 413-G5^T^) show that this species belongs to *Penicillium* sect. *Canescentia*, and it is most closely related to *P*. *arizonense* and *P*. *yarmokense*. An overview taxonomic study of this section is lacking, and we therefore wait with combining this species in *Penicillium*. Besides the two currently accepted species, our phylogenetic analysis (Supplementary Fig. S1) also shows that *Penicillium arenicola* and *Merimbla humicoloides* belong to *Phialomyces*. These species are combined in *Phialomyces* below and in total four species are treated in this genus: *Ph*. *arenicola*, *Ph*. *fusiformis*, *Ph*. *humicoloides* and *Ph*. *macrosporus*.  

***Pseudohamigera*** Houbraken, Frisvad & Samson, ***gen*. *nov*.** MycoBank MB832554.  

*Etymology*: Named after *Hamigera*.  

*Type*: *Penicillium striatum* Raper & Fennell  

*Diagnosis*: Phylogenetically distinct. Conidiophores penicillium-like, monoverticillate or biverticillate; conidia pale grey; ascospores elliptical with walls bearing a series of wavy, longitudinal flanges or frills. Mesophilic.  

*Notes*: *Pseudohamigera* is phylogenetically most closely related to *Evansstolkia* (Fig. 1), a newly erected genus to accommodate *Talaromyces leycettanus* (see above). The ascospores of *Pseudohamigera* somewhat resemble those of *Evansstolkia* (Fig. 5, Fig. 6). The main difference between those genera is their optimum and maximum growth temperature. *Pseudohamigera* is a mesophile with an optimum growth temperature between 25 and 30 °C and a maximum around 37 °C; *Evansstolkia* is thermotolerant to thermophilic (optimum around 40 °C; maximum around 55 °C). *Hamigera* and *Pseudohamigera* differ markedly from each other in the ornamentation of their ascospores and in their conidial state. The ascospores of *Pseudohamigera* have 8–12 longitudinal striations while those of *Hamigera* are pitted. Furthermore, the asexual morph of *Pseudohamigera* is penicillium-like, and *Hamigera* has a merimbla-type conidiophore. Stolk & Samson (1971) already mentioned that these differences would justify classifying *H*. *striata* (= *P*. *striatum*) and *H*. *avellanea* in separate genera, but this was deferred at that time. *Warcupiella* differs from *Pseudohamigera* by the production of conspicuously spiny ascospores, without a trace of a furrow or equatorial ridge. Furthermore, *Warcupiella* produces a raperia-type (aspergillus-like) asexual morph.  

***Pseudopenicillium*** Guevara-Suarez *et al.*, Fungal Syst. Evol. 5: 66. 2020. MycoBank MB822076.  

*Type*: *Pseudopenicillium megasporum* (Orpurt & Fennell) Guevara-Suarez, Cano & Gené  

*Notes*: The genus *Pseudopenicillium* was recently introduced and two species previously classified in *Penicillium*, *Penicillium megasporum* and *Penicillium giganteum*, were combined in this genus. In the same article, *Pseudopenicillium cervifimosum* was newly described. *Pseudopenicillium* is characterised by its penicillium-like conidiophores and brown globose conidia with conspicuous disjunctors (Fig. 10). The genus is phylogenetically most closely related to *Hamigera* (Fig. 1, Guevara-Suarez *et al.* 2020). *Pseudopenicillium* differs morphologically from *Hamigera* by the production of large (6–9 μm), globose, dark or dull green conidia; the conidia of *Hamigera* are smaller (3–5 μm in length), (sub)ellipsoidal and in shades of brown (avellaneous). A sexual morph in *Pseudopenicillium* is unknown. *Pseudopenicillium* produces penicillium-like conidiophores, but can morphologically be differentiated from *Penicillium* and *Penicillago* by its short and often irregularly branched conidiophores producing large conidia in short chains. Furthermore, the colonies of *Pseudopenicillium* species on YES agar have a yeast-like appearance.

 Pitt (1980) introduced series *Megaspora* to accommodate *Penicillium megasporum* and *P*. *asperosporum*. These species could be differentiated based on conidium size: the conidia of *P*. *megasporum* are 6–7 μm, and those of *P*. *asperosporum* measure 4–5 μm. Pitt (1980) further noted that both species show little affinity with most other *Penicillium* species, with *P*. *montanense* being one of the exceptions. In a more recent taxonomic study by Houbraken *et al.* (2014b), they treated *P*. *asperosporum* as a synonym of *P*. *montanense* in section *Aspergilloides*. Based on sequence data of six loci, Peterson *et al.* (2010) depicted *P*. *giganteum* and *P*. *megasporum* as sibling species and accepted both species. CBS 144.69 (ex-type of *P*. *giganteum*) differs from CBS 256.55 (ex-type *P*. *megasporum*) in producing smaller colonies (CYA 8 mm; YES 15 mm; MEA 15 mm; DG18 10 mm *vs* CYA 18 mm; YES 21 mm; MEA 19 mm; DG18 17 mm). Furthermore, CBS 144.69 did not grow on CREA, while CBS 256.55 shows weak to moderate growth (7 mm) with weak acid production. We accept both species based on our observations and the phylogenetic data reported in Peterson *et al.* (2010).

**Fig. 9:**
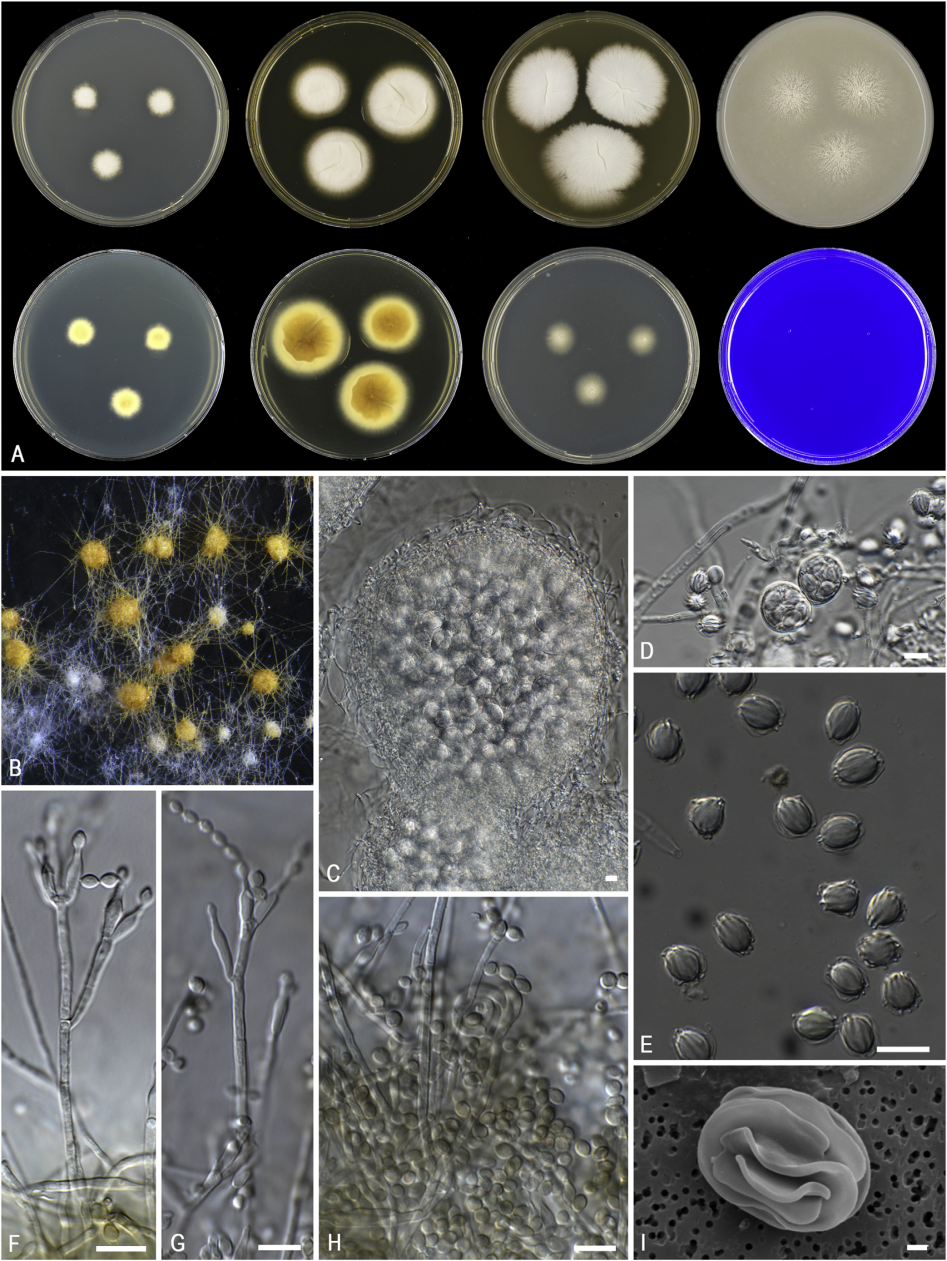
Morphological characters of *Pseudohamigera striata*. **A.** Colonies from left to right, after 7 d at 25 °C (top row) CYA, YES, MEA, OA; (bottom row) CYA reverse, YES reverse, DG18, CREA. **B.** Ascomata on OA after 8 wk at 25 °C. **C.** Ascoma and asci. **D.** Asci and ascospores. **E.** Ascospores. **F–G.** Conidiophores. **H.** Conidia. **I.** SEM micrograph of an ascospore. Scale bars: C–H = 10 μm; I = 1 μm.

**Fig. 10:**
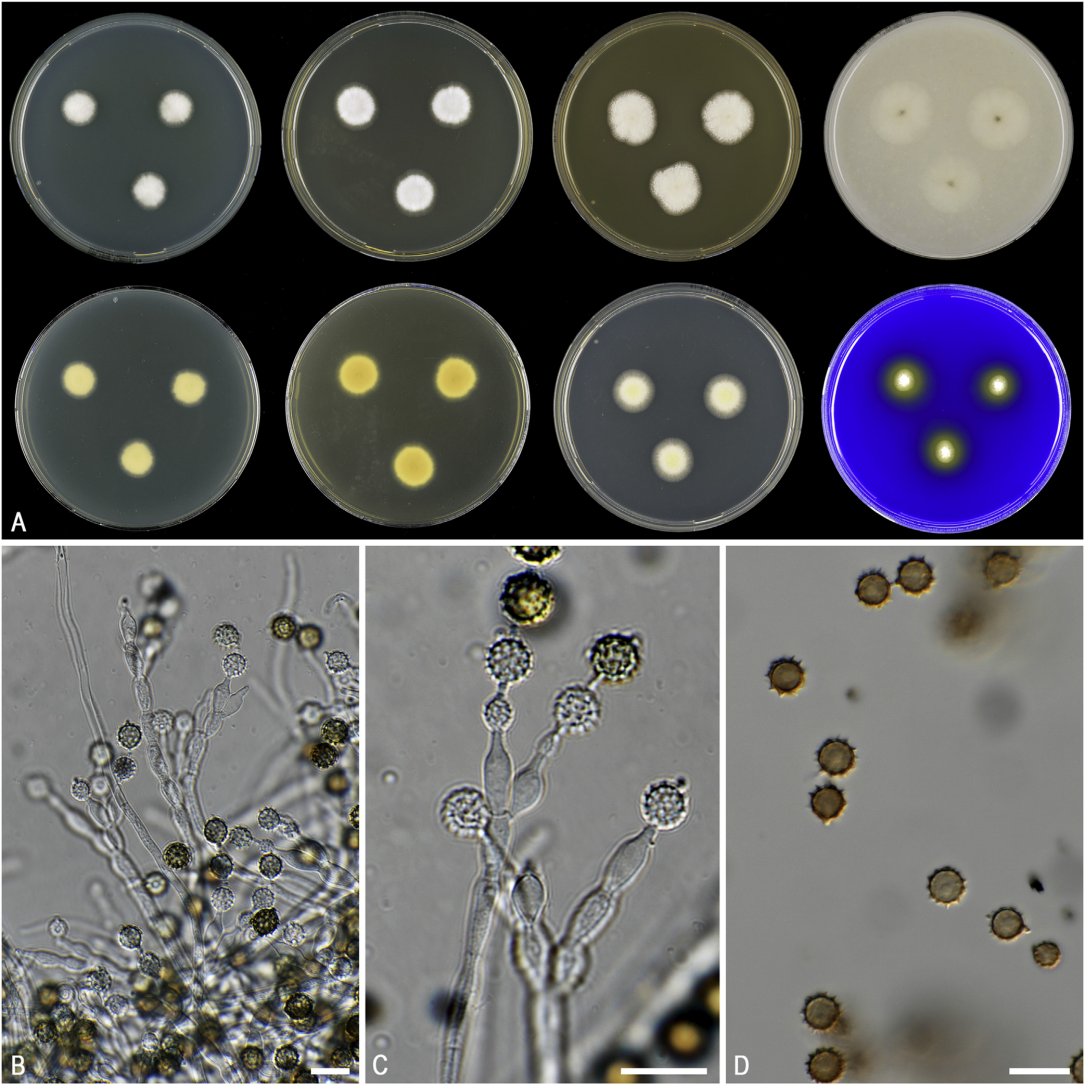
Morphological characters of *Pseudopenicillium megasporum* (CBS 256.55^NT^). **A.** Colonies from left to right, after 7 d at 25 °C (top row) CYA, YES, MEA, OA; (bottom row) CYA reverse, YES reverse, DG18, CREA. **B–C.** Conidiophores and conidia. **D.** Conidia. Scale bars: B–D = 10 μm.

***Rasamsonia*** Houbraken & Frisvad, Antonie van Leeuwenhoek 101: 411. 2011 [2012]. MycoBank MB519868.  

*Type*: *Rasamsonia emersonii* (Stolk) Houbraken & Frisvad  

*Notes*: Houbraken *et al.* (2012b) erected the genus *Rasamsonia* for the eurotialean *Geosmithia* species *G*. *argillacea*, *G*. *cylindrospora*, and *G*. *eburnea* (sexual morph, *Talaromyces eburneus*). The type of *Geosmithia*, *Penicillium lavendulum*, is a member of the *Hypocreales* and this name could therefore not be used for these eurotialean *Geosmithia* species. In the same publication, also the thermophiles *Talaromyces emersonii* and *Tal*. *byssochlamydoides* were transferred to *Rasamsonia*. *Rasamsonia* is characterised by producing olive-brown conidia, cylindrical phialides that usually gradually taper towards the apices and conidiophores with distinctly rough-walled stipes; ascomata, if present, have a scanty covering. *Rasamsonia* is phylogenetically a sister genus of *Trichocoma* (Houbraken and Samson, 2011, Houbraken et al., 2012b, Fig. 1). *Rasamsonia* phenotypically resembles *Paecilomyces*, and both genera contain thermotolerant species, produce olive brown conidia, and form ascomata with no or scarce ascomatal covering, but *Rasamsonia* differs from *Paecilomyces* in having more regularly branched conidiophores with distinctly rough-walled structures (Houbraken *et al.* 2012b). With the introduction of the genus, Houbraken *et al.* (2012b) combined five species in the genus (*Rasamsonia argillacea*, *R*. *eburnea*, *R*. *cylindrospora*, *R*. *byssochlamydoides*, *R*. *emersonii*) and described one new species (*R*. *brevistipitata*). Six new species were described afterwards, and all are accepted here: *Rasamsonia composticola* (Su & Cai 2013), *Rasamsonia aegroticola*, *R*. *piperina* (Houbraken *et al.* 2013), *Rasamsonia pulvericola* (Tanney & Seifert 2013), *Rasamsonia columbiensis* (Crous *et al.* 2016) and *Rasamsonia frigidotolerans* (Rodríguez-Andrade *et al.* 2020). This genus currently consists of 12 species. The genus originally only included thermotolerant or thermophilic species that have optimum growth temperatures above 30 °C and maximum growth temperatures above 45 °C (as defined by Cooney and Emerson, 1964, Crisan, 1973, Maheshwari et al., 2000). Interestingly, *R*. *frigidotolerans* and *R*. *pulvericola* are mesophiles, which means that the generic description should be expanded. The presence of a mesophilic species within this otherwise thermotolerant/thermophilic genus is an interesting subject for investigation of the evolution of thermophily.  

***Sagenomella*** W. Gams, Persoonia 10: 100. 1978. MycoBank MB9773.  

*Type*: *Sagenomella diversispora* (J.F.H. Beyma) W. Gams  

*Notes*: The genus *Sagenomella* was introduced by Gams (1978) to accommodate acremonium-like fungi and is characterised by the production of connected conidial chains and sympodially proliferating, often centrally swollen phialides. This genus is typified with *Sagenomella diversispora*, and Houbraken & Samson (2011) showed that this genus is phylogenetically unrelated to *Penicillium s*. *str*. Phylogenetically, this genus is most closely related to the new genus *Acidotalaromyces* (Fig. 1). In contrast to *Sagenomella*, the sole species in *Acidotalaromyces* produces penicillium-like conidiophores and has a preference for acidic substrates.

Gams (1978) described three species (*Sagenomella alba*, *S*. *oligospora*, *S*. *verticillata*) and combined six in *Sagenomella* (*S*. *diversispora*, *S*. *griseoviridis*, *S*. *humicola*, *S*. *sagenomatis*, *S*. *sclerotialis*, *S*. *striatispora*). Another six species and one variety were included in the genus afterwards: *S*. *alba* var. *synnematosa*, *S*. *bohemica*, *S*. *chlamydospora*, *S*. *dimorphica*, *S*. *keratitidis*, *S*. *ocotl* and *S*. *ryukyuensis* (Ueda and Udagawa, 1984, Fassatiová and Pecková, 1990, Gené et al., 2003, Seixas et al., 2005, Samson et al., 2011c). A taxonomic revision of the genus using molecular data is lacking.

 *Sagenomella alba* and *S. oligospora* do not belong to the *Trichocomaceae* and should be transferred to another genus. An ITS sequence (MH860843) of the type strain of *S*. *alba* (CBS 167.74^T^) indicates a relationship with *Crocicreas* in *Helotiales* (*Leotiomycetes*). Comparison of LT633929, an ITS sequence of the type of *S*. *oligospora* (CBS 168.74^T^) shows that this species probably represents a new genus or species in *Sordariomycetes* (near *Phialemonium*). Various species that were originally described in *Sagenomella* are combined in other genera. *Sagenomella bohemica*, *S*. *ryukyuensis* and *S*. *sagenomatis* belong to *Talaromyces* and the accepted names of those species are *Talaromyces bohemicus*, *Tal*. *ryukyuensis* and *Tal*. *viride*, respectively (Yilmaz *et al.* 2014). *Sagenomella chlamydospora*, *S*. *keratitidis* and *S*. *sclerotialis* are classified in *Aspergillus* as *Aspergillus chlamydospora*, *A*. *keratitidis* and *A*. *sclerotialis*, respectively (Samson *et al.* 2014). *Sagenomella dimorphica* was invalidly described (without type, Art. 40.1). Based on the data presented above, the following six species are accepted in *Sagenomella*: *S*. *diversispora*, *S*. *griseoviridis*, *S*. *humicola*, *S*. *ocotl*, *S*. *striatispora* and *S*. *verticillata*. The taxonomic position of *S*. *alba* var. *synnematosa* is unknown and it is unclear whether this variety should be raised to species level or should be considered a synonym of “*Sagenomella alba*”.  

***Sclerocleista*** Subram., Curr. Sci. 41: 757. 1972. MycoBank MB4928.  

*Type*: *Sclerocleista ornata* (Raper, Fennell & Tresner) Subram.  

*Notes*: The genus *Sclerocleista* was introduced to accommodate the sexually reproducing species *Aspergillus ornatus* (Subramanian 1972). Raper *et al.* (1953) noted that this species produces parenchymatous, purplish cleistothecia that differed markedly from all other Aspergilli. Based on these observations, they suggested that this species should be placed in a novel group of potentially ascosporic species. Another phenotypically closely related species is *Aspergillus citrisporus* (Raper *et al.* 1953). The name *Sclerocleista thaxteri* was introduced for this sexually reproducing *Aspergillus* species (Subramanian 1972). These two phenotypically similar species are also phylogenetically closely related and the only members of *Sclerocleista* to date. They form a distinct lineage in the *Aspergillaceae*, distantly related to *Aspergillus*, and sister to *Phialomyces* (Fig. 1).  

***Talaromyces*** C.R. Benj., Mycologia 47: 681. 1955. MycoBank MB5347.  

*Type*: *Talaromyces flavus* (Klöcker) Stolk & Samson  

*Notes*: Benjamin (1955) established the genus *Talaromyces* for sexual reproducing *Penicillium* species that produce soft-walled ascomata covered with interwoven hyphae. Several phylogenetic studies (*e.g.*, LoBuglio et al., 1993, Berbee et al., 1995, Ogawa et al., 1997, Ogawa and Sugiyama, 2000, Wang et al., 2007a, Houbraken and Samson, 2011, Samson et al., 2011c) have shown the majority of species belonging to *Penicillium* subgen. *Biverticillium* reside in a clade with species producing a talaromyces sexual morph. This clade is distantly related to *Penicillium* and following the single nomenclature, these taxa were accommodated in *Talaromyces* (Samson *et al.* 2011c). Yilmaz *et al.* (2014) provided a monograph of *Talaromyces* and accepted 88 species placed in seven sections: *Bacillispori*, *Helici*, *Islandici*, *Purpurei*, *Subinflati*, *Talaromyces* and *Trachyspermi*. This monograph was the basis of the description of many new taxa and in recent years many new species were described all over the world. The number of species grew rapidly and have now reached more than 170 species. Interestingly, Rodríguez-Andrade *et al.* (2019) described new *Talaromyces* species with basipetospora-like conidiophores, which have not been previously described in *Talaromyces*. The phylogenetic relationships within *Talaromyces* is given in Supplementary Fig. S3 and an overview of the species within the genus is given below in the list.  

***Thermoascus*** Miehe, Selbsterhitz. Heus: 70. 1907. MycoBank MB5446.  

*Type*: *Thermoascus aurantiacus* Miehe  

*Notes*: The taxonomy of *Thermoascus* has changed over time and *Dactylomyces* and *Coonemeria* are currently considered synonyms (Apinis, 1967, Mouchacca, 1997). *Thermoascus* is characterised by the production of orange-yellow, brown or red-brown, soft cleistothecia formed in a more or less continuous crust-like layer with a pseudoparenchymatous wall. The species of the genus are thermophilic. The asexual morphs of *Thermoascus* differ significantly and can be absent, or paecilomyces-, or polypaecilum-like. *Thermoascus* is phylogenetically related to *Paecilomyces* (Fig. 1, Houbraken & Samson 2011). This link is illustrated by *Byssochlamys verrucosa (=* *Paecilomyces verrucosus*). This species was, based on phenotypic characters, described in *Paecilomyces*, but it phylogenetically belongs in *Thermoascus*. A new combination for this species as *Th*. *verrucosus* is proposed below. The addition of this species to *Thermoascus* further expands the phenotypic diversity of the genus.

 Six species (*Th*. *aegyptiacus*, *Th*. *aurantiacus*, *Th*. *crustaceus*, *Th*. *isatschenkoi*, *Th*. *taitungiacus* and *Th*. *thermophilus*) and two varieties (*Th*. *crustaceus* var. *verrucosus* and *Th*. *aurantiacus* var. *levisporus*) are described in *Thermoascus*. A taxonomic study using sequence data and dealing with all currently described species is lacking. *Thermoascus isatschenkoi* Malchevskaya (1939) is regarded as a doubtful species of which no satisfactory description exists and no material is available for examination. An LSU sequence from *Thermoascus aurantiacus* var. *levisporus* ATCC 46197 (obtained directly from the ATCC website, www.lgcstandards-atcc.org/) is identical to *Thermoascus aurantiacus* ATCC 204492 and NRRL 5861, and similar to CBS 398.64 (1 bp difference) and CBS 257.34 (2 bp difference). We therefore tentatively treat this species as a synonym of *Thermoascus aurantiacus*. *Thermoascus crustaceus* var. *verrucosus* is a distinct species (see below) and the name *Th*. *yaguchii* is introduced for this variety. Sequences show that *Th*. *aegyptiacus* is a distinct species, related to *Th*. *crustaceus* and *Th*. *yaguchii*; the relationship of *Th*. *thermophilus* is unresolved. The taxonomic status of *Th*. *taitungiacus* remains unknown. In total, eight *Thermoascus* species are included in the list (including the new combination *Th*. *verrucosus* and the new name *Th*. *yaguchii*).  

***Thermomyces*** Tsikl., Ann. Inst. Pasteur 13: 500. 1899. MycoBank MB10209.  

*Type*: *Thermomyces lanuginosus* Tsikl.  

*Notes*: *Thermomyces* is phylogenetically most closely related to *Ascospirella*, but is phenotypically distinct (see under *Ascospirella*) (Houbraken & Samson 2011, Fig. 1). *Thermomyces* is a thermophilic genus and species of the genus are commercially used for the production of various (thermostable) enzymes. The genera have different sexual morphs and five species are described since the introduction of *Thermomyces*: *Th*. *dupontii*, *Th*. *ibadanensis*, *Th*. *lanuginosus* (generic type), *Th*. *stellatus* and *Th*. *verrucosus*. *Thermomyces stellatus* and *Th*. *verrucosus* are classified in the *Microascaceae* and *Chaetomiaceae*, respectively, and *Th*. *ibadanensis* is a synonym of *Th*. *lanuginosus* (Houbraken et al., 2014a, Wang et al., 2019b).  

***Trichocoma*** Jungh., Praem. Fl. Crypt. Java: 9. 1838. MycoBank MB5551.  

*Type*: *Trichocoma paradoxa* Jungh.  

*Notes*: The monotypic genus *Trichocoma* is characterised by asci in hyphal masses or tufts that can measure up to 10–20 mm (Kominami et al., 1952, Malloch, 1985). The sexual morph is only observed on natural substrates and not seen on agar media; a talaromyces-like asexual morph can be present on agar media. *Trichocoma* is phylogenetically most closely related to *Rasamsonia* (Fig. 1, Houbraken and Samson, 2011, Houbraken et al., 2012b). The majority of *Rasamsonia* species are thermotolerant or thermophilic, while *Trichocoma paradoxa* is mesophilic. Furthermore, *Rasamsonia* produces scanty ascomatal coverings and distinctly ornamented conidiophore stipes.  

***Warcupiella*** Subram., Curr. Sci. 41: 757. 1972. MycoBank MB5762.  

*Type*: *Warcupiella spinulosa* (Warcup) Subram.  

*Notes*: *Warcupiella* is a monotypic genus (Subramanian 1972) that was introduced to accommodate the sexually reproducing *Aspergillus spinosus* (Raper & Fennell 1965). Raper & Fennell (1965) mentioned that the classification of *A*. *spinulosus* in *Aspergillus* was difficult because the ascosporic stage differed from all other ascosporic species known at that time in having large, spiny ascospores without any a trace of equatorial ridges or furrows. Besides the unique sexual morph, *A*. *spinosus* also produces an asexual morph that is not typical for *Aspergillus*. A typical *Aspergillus* conidiophore terminates in a vesicle on which several metulae or phialides develop synchronously. In contrast, the conidiophores of *Warcupiella spinulosa* (*A*. *spinulosus*) terminate in a subvesicle. This subvesicle is cut off by a septum into a shorter apical cell and a larger basal portion. The apical cell develops into a phialide, and several smaller phialides arising later from the part of the subvesicle below the septum (Subramanian & Rajendran 1979). This observation was sufficient evidence to introduce the new genus *Raperia* for the asexual morph of *Warcupiella* (type *Raperia spinulosa*) (Subramanian & Rajendran 1979). Our phylogenetic analysis confirms the unique position of this species outside *Aspergillus*. Based on the publication date, *Warcupiella* has priority over *Raperia*. This species is phylogenetically sister of a clade containing *Pseudopenicillium* and *Hamigera* (Fig. 1). One species is classified in this genus, *Warcupiella spinulosa*.  

***Xerochrysium*** Pitt, IMA Fungus 4: 236. 2013. MycoBank MB807003.  

*Type*: *Xerochrysium dermatitidis* (A. Agostini) Pitt  

*Notes*: *Xerochrysium* was erected for xerophilic species belonging to the *Eurotiales* that produce a chrysosporium-like asexual morph. Species are characterised by the production of aleurioconidia, but also by the formation of chlamydoconidia and arthroconidia (Pitt *et al.* 2013). *Xeromyces*, a genus containing the extreme xerophile *X*. *bisporus*, is phylogenetically related (Fig. 1) and the xerophilic nature of both indicates a close relationship. *Xeromyces* is distinguished from *Xerochrysium* because it is primarily a sexual genus, in which fresh isolates readily produce characteristic asci containing two D-shaped ascospores; unlike *Xerochrysium*, it does not produce chlamydo- or aleurioconidia, instead producing a rare fraseriella-type asexual morph (Pitt *et al.* 2013). Two species are described in this genus: *Xerochrysium dermatitidis* and *X*. *xerophilum* (Pitt *et al.* 2013).  

***Xeromyces*** L.R. Fraser, Proc. Linn. Soc. New South Wales 78: 245. 1954. MycoBank MB5830.  

*Type*: *Xeromyces bisporus* L.R. Fraser  

*Notes*: See also *Xerochrysium*. The genus *Xeromyces* has a single species, *X*. *bisporus*. This is an extreme xerophile and growth will not occur on media with a high water activity. *Xeromyces bisporus* produces colourless cleistothecia, with evanescent asci containing two ”D”-shaped ascospores.

#### Doubtful genera

***Ascorhiza*** Lecht.-Trinka, Compt. Rend. Hebd. Séances Acad. Sci., 192: 499. 1931. MycoBank MB372.  

*Type*: *Ascorhiza leguminosarum* Lecht.-Trinka  

*Notes*: *Ascorhiza* is considered a doubtful genus. It was introduced by Lechtova-Trinka (1931) for a cleistothecial ascomycete parasitic in root tubers of *Astragalus alopecuroides*. No type or other material was available for this study, but from the description and illustrations in Lechtova-Trinka (1931), it is apparent that this fungus produces ascomata, subglobose asci and ellipsoidal, reticulate ascospores that measure 9–10 × 6 μm in size. The fungus was not cultivated, but was observed developing in root tubers, with the ascomata surrounded by the tissue of the tuber. Because of the incomplete description and absence of type material, the genus *Ascorhiza* is regarded as doubtful (Samson & Mouchacca 1975b).

### Infrageneric classification (subgenera, sections and series) in *Aspergillus* and *Penicillium*

An updated infrageneric classification system is presented below for *Aspergillus* and *Penicillium*. Besides these two genera, an infrageneric classification is also present in *Monascus* and *Talaromyces* (Yilmaz et al., 2014, Barbosa et al., 2017). *Monascus* includes two sections (*Floridani* and *Rubri*; Fig. 1) and *Talaromyces* seven (*Bacillispori*, *Helici*, *Islandici*, *Purpurei*, *Subinflati*, *Talaromyces* and *Trachyspermi*; Supplementary Fig. S3). *Monascus* is relatively small with nine accepted species, and a further subdivision in series is not useful or needed. On the other hand, *Talaromyces* includes 170 species and a series classification of this species-rich genus could be useful. However, the phylogenetic relationships below section level are less resolved (see Supplementary Fig. S3) than for *Aspergillus* and *Penicillium* and we therefore decided to wait with the introduction of a series classification until more data becomes available, and/or more species are being described. The infrageneric classification proposed below is mainly based on phylogenetic data and supplemented with phenotype, physiology and extrolite data. As discussed above, these latter characters can be present in most, but not all members of the class (polythetic classes). Descriptions are given for all accepted series and the sections without a formal series classification. None of the series (and subseries) in Thom and Church, 1926, Thom, 1930, Thom and Raper, 1945, Raper and Thom, 1949 and Ramírez (1982) are validly published due to the form in which they are presented (*Art*. 21.1., 36.1). Only sections and series names are considered and compete; those from different ranks are not included in this study. *Aspergillus* is subdivided 75 series (73 new, one new combination and the autonym *Aspergillus*), and *Penicillium* in 89 series (57 new, six new combinations) (excl. the informally introduced series names, see Table 4, Table 5).

**Table 4:** Overview of current infrageneric classification system in *Aspergillus*; the number of accepted species per section and series are mentioned between brackets^2^.

Subgenus	Section	Series
*Aspergillus*	*Aspergillus* (32)	*Aspergillus* (11)
		*Chevalierorum* (7)
		*Leucocarpi* (1)
		*Rubri* (9)
		*Tamarindosolorum* (1)
		*Teporium* (1)
		*Xerophili* (2)
	*Restricti* (22, incl. *A. tapirirae*)	*Halophilici* (1)
		*Penicillioides* (9)
		*Restricti* (9)
		*Vitricolarum* (2)
*Circumdati*	*Candidi* (7)	*Candidi*^1^ (7)
	*Circumdati* (27)	*Circumdati* (11)
		*Sclerotiorum* (9)
		*Steyniorum* (7)
	*Flavi* (35)	*Alliacei* (5)
		*Avenacei* (1)
		*Bertholletiarum* (1)
		*Coremiiformes* (2)
		*Flavi* (16)
		*Kitamyces* (4)
		*Leporum* (3)
		*Nomiarum* (3)
	*Flavipedes* (15)	*Flavipedes* (9)
		*Neonivei* (1)
		*Olivimuriarum* (1)
		*Spelaei* (4)
	*Janorum* (4)	*Janorum*^1^ (4)
	*Nigri* (29)	*Carbonarii* (4)
		*Heteromorphi* (2)
		*Homomorphi* (1)
		*Japonici* (12)
		*Nigri* (10)
	*Petersoniorum* (4)	*Petersoniorum*^1^ (4)
	*Robusti* (1)	*Robusti*^1^ (1)
	*Tannerorum* (1)	*Tannerorum*^1^ (1)
	*Terrei* (17)	*Ambigui* (2)
		*Nivei* (6)
		*Terrei* (9)
*Cremei*	*Cremei* (17)	*Arxiorum* (1)
		*Brunneouniseriati* (2)
		*Cremei* (5)
		*Inflati* (3)
		*Pulvini* (1)
		*Wentiorum* (5)
*Fumigati*	*Cervini* (10)	*Acidohumorum* (1)
		*Cervini* (9)
	*Clavati* (8)	*Clavati*^1^ (8)
	*Fumigati* (59)	*Brevipedes* (4)
		*Fennelliarum* (5)
		*Fumigati* (10)
		*Neoglabri* (11)
		*Spathulati* (2)
		*Thermomutati* (3)
		*Unilaterales* (12)
		*Viridinutantes* (12)
	*Vargarum* (1)	*Vargarum*^1^ (1)
*Nidulantes*	*Aenei* (10)	*Aenei*^1^ (10)
	*Bispori* (1)	*Bispori*^1^ (1)
	*Cavernicolarum* (5)	*Cavernicolarum* (4)
		*Egyptiaci* (1)
	*Nidulantes* (74)	*Aurantiobrunnei* (2)
		*Multicolores* (6)
		*Nidulantes* (25)
		*Speluncei* (6)
		*Stellati* (13)
		*Unguium* (5)
		*Versicolores* (17)
	*Ochraceorosei* (3)	*Funiculosi* (1)
		*Ochraceorosei* (2)
	*Raperorum* (2)	*Raperorum*^1^ (2)
	*Silvatici* (1)	*Silvatici*^1^ (1)
	*Sparsi* (9)	*Biplani* (2)
		*Conjuncti* (4)
		*Implicati* (1)
		*Sparsi* (2)
	*Usti* (25)	*Calidousti* (12)
		*Deflecti* (5)
		*Monodiorum* (1)
		*Usti* (7)
*Polypaecilum*	*Polypaecilum* (16)	*Canini* (2)
		*Kalimarum* (2)
		*Noonimiarum* (5)
		*Polypaecilum* (2)
		*Salinarum* (4)
		*Whitfieldiorum* (1)

1 Informally introduced series (for details, see Notes).

2 The taxonomic position of *Aspergillus argenteus*, *A. beijingensis*, *A. collembolorum*, *A. crassihyphae*, *A. ellipsoideus*, *A. maritimus*, *A. qizutongii*, *A. raianus*, *A. subunguis*, *A. vinosobubalinus* and *A. wangduanlii* is uncertain and are therefore not included in this Table.

**Table 5:** Overview of current infrageneric classification system in *Penicillium*; the number of accepted species per section and series are mentioned between brackets.

Subgenus	Section	Series
*Aspergilloides*	*Alfrediorum* (1)	*Alfrediorum*^1^ (1)
	*Aspergilloides* (53)	*Fortuita* (1)
		*Glabra* (7)
		*Hoeksiorum* (2)
		*Improvisa* (1)
		*Kiamaensia* (1)
		*Livida* (3)
		*Longicatenata* (2)
		*Pinetorum* (9)
		*Quercetorum* (1)
		*Saturniformia* (1)
		*Spinulosa* (7)
		*Sublectatica* (3)
		*Thiersiorum* (1)
		*Thomiorum* (12)
		*Verhageniorum* (2)
	*Charlesia* (9)	*Costaricensia* (1)
		*Fellutana* (2)
		*Indica* (4)
		*Phoenicea* (2)
	*Cinnamopurpurea* (20)	*Cinnamopurpurea* (3)
		*Idahoensia* (12)
		*Jiangxiensia* (2)
		*Nodula* (3)
	*Citrina* (42)	*Citrina* (7)
		*Copticolarum* (3)
		*Euglauca* (4)
		*Gallaica* (1)
		*Paxillorum* (1)
		*Roseopurpurea* (2)
		*Sheariorum* (1)
		*Sumatraensia* (1)
		*Westlingiorum* (22)
	*Crypta* (1)	*Crypta*^1^ (1)
	*Eremophila* (1)	*Eremophila*^1^ (1)
	*Exilicaulis* (58)	*Alutacea* (2)
		*Citreonigra* (4)
		*Corylophila* (11)
		*Erubescentia* (18)
		*Lapidosa* (14)
		*Restricta* (9)
	*Gracilenta* (6)	*Angustiporcata* (1)
		*Estinogena* (1)
		*Gracilenta* (1)
		*Macrosclerotiorum* (3)
	*Griseola* (1)	*Griseola*^1^ (1)
	*Inusitata* (2)	*Inusitata*^1^ (2)
	*Lanata-Divaricata* (76)	*Dalearum* (13)
		*Janthinella* (24)
		*Oxalica* (3)
		*Rolfsiorum* (16)
		*Simplicissima* (20)
	*Lasseniorum* (1)	*Lasseniorum*^1^ (1)
	*Ochrosalmonea* (2)	*Ochrosalmonea* (2)
	*Ramigena* (6)	*Georgiensia* (1)
		*Ramigena* (5)
	*Sclerotiorum* (35)	*Adametziorum* (13)
		*Herqueorum* (5)
		*Sclerotiorum* (17)
	*Stolkia* (7)	*Stolkia*^1^ (7)
	*Thysanophora* (8)	*Thysanophora*^1^ (8)
	*Torulomyces* (15)	*Torulomyces*^1^ (15)
*Penicillium*	*Brevicompacta* (11)	*Brevicompacta* (5)
		*Buchwaldiorum* (2)
		*Olsoniorum* (3)
		*Tularensia* (1)
	*Canescentia* (16)	*Atroveneta* (5)
		*Canescentia* (11)
	*Chrysogena* (18)	*Aethiopica* (1)
		*Chrysogena* (12)
		*Crustacea* (3)
		*Goetziorum* (1)
		*Persicina* (1)
	*Eladia* (2)	*Eladia*^1^ (2)
	*Fasciculata* (32)	*Camembertiorum* (11)
		*Corymbifera* (9)
		*Gladioli* (1)
		*Verrucosa* (3)
		*Viridicata* (8)
	*Formosana* (1)	*Formosana*^1^ (1)
	*Osmophila* (2)	*Osmophila* (1)
		*Samsoniorum* (1)
	*Paradoxa* (9)	*Atramentosa* (6)
		*Paradoxa* (3)
	*Penicillium* (8)	*Clavigera* (2)
		*Digitata* (1)
		*Italica* (2)
		*Penicillium* (2)
		*Sclerotigena* (1)
	*Ramosum* (17)	*Lanosa* (6)
		*Raistrickiorum* (3)
		*Scabrosa* (1)
		*Soppiorum* (6)
		*Virgata* (1)
	*Robsamsonia* (14)	*Claviformia* (1)
		*Glandicolarum* (4)
		*Robsamsonia* (7)
		*Urticicolae* (2)
	*Roquefortorum* (5)	*Roquefortorum* (5)
	*Turbata* (4)	*Turbata*^1^ (4)

1 Informally introduced series (for details, see Notes).

***Aspergillus* subgen. *Aspergillus*** [autonym], MycoBank MB701330.  

*Type*: *Aspergillus glaucus* (L.) Link, Mag. Ges. Naturf. Freunde Berlin 3: 16. 1809.  

*Description*: See Gams *et al.* (1985) (morphology), Houbraken and Samson, 2011, Kocsubé et al., 2016, this study (Fig. 2) (phylogeny).  

**Section *Aspergillus*** [autonym], MycoBank MB548676.  

*Type*: *Aspergillus glaucus* (L.) Link, Mag. Ges. Naturf. Freunde Berlin 3: 16. 1809.  

*Description*: See Gams *et al.* (1985) (morphology), Kocsubé *et al.* (2016), this study (Fig. 2) (phylogeny), Chen et al., 2017, Sklenář et al., 2017 (morphology, phylogeny).  

Series ***Aspergillus*** [autonym], MycoBank MB834209.  

*Type*: *Aspergillus glaucus* (L.) Link, Mag. Ges. Naturf. Freunde Berlin 3: 16. 1809.  

**Fig. 11:**
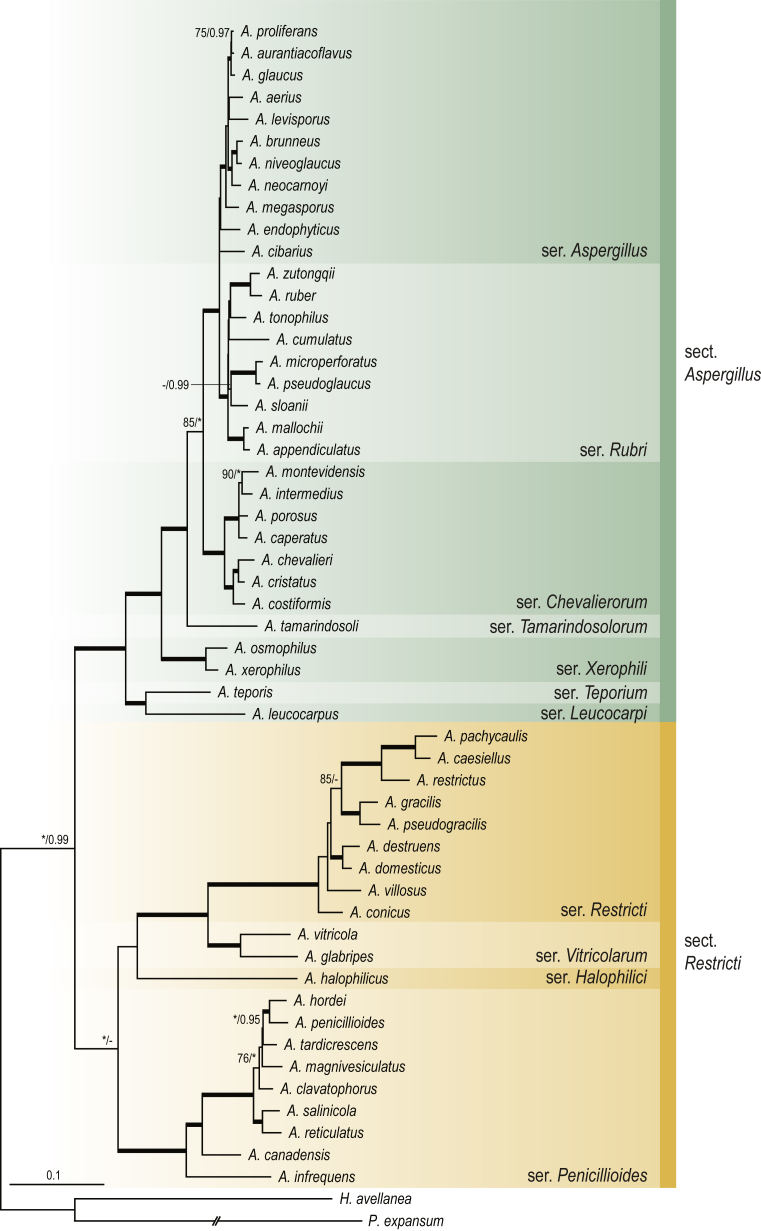
Combined phylogeny for *BenA*, *CaM* and *RPB2* data sets showing the phylogenetic relation of species, series and sections within *Aspergillus* subgen. *Aspergillus*. The BI posterior probability (pp) values and bootstrap percentages of the maximum likelihood (ML) analysis are presented at the nodes; fully supported branches are thickened. Values less than 70 % bootstrap support (ML) or less than 0.95 posterior probability (Bayesian analysis) are indicated with a hyphen or not shown. The bar indicates the number of substitutions per site. The phylogram is rooted with *Hamigera avellanea* and *Penicillium expansum*.

*Diagnosis*: *Phylogeny*: Series *Aspergillus* belongs to sect. *Aspergillus*, subgen. *Aspergillus*; this series forms a well-supported clade together with members of ser. *Rubri* (Fig. 11); the phylogenetic relationship of *A*. *cibarius* and *A*. *endophyticus* is unresolved and these species are tentatively classified in ser. *Aspergillus*. *Morphology & physiology*: Colonies restricted on MEA, spreading on low water activity media (*e.g*., M40Y), yellow or orange; conidiophores uniseriate; no growth on CY20S and M60Y at 37 °C, most species grow moderately on CY20S; rapid growth on M60Y at 25 °C, except *A*. *neocarnoyi* (restrictedly CY20S at 25 °C, 3–5 mm after 7 d). *Sexual morph* eurotium-type, homothallic, yellow; ascospores non-crested, with low crests (<0.5 μm) or irregular crests measuring 0.5–1 μm. Series description based on Chen et al., 2017, Visagie et al., 2017.

*Included species*: *Aspergillus aerius*, *A*. *aurantiacoflavus*, *A*. *brunneus*, *A*. *cibarius*, *A*. *endophyticus*, *A*. *glaucus*, *A*. *levisporus*, *A*. *megasporus*, *A*. *neocarnoyi*, *A*. *niveoglaucus*, *A*. *proliferans*.  

*Extrolites*: All species produce echinulins, neoechinulins, isoechinulins, auriglaucins and flavoglaucins in common with most other species in section *Aspergillus*. Mycophenolic acid and its precursor 5,7-dihydroxy-4-methylphthalide (Grove 1972) has been found in *A*. *brunneus* and *A*. *niveoglaucus* in series *Aspergillus* and in *A*. *pseudoglaucus* (identified as *A*. *flavus* by Grove (1972)) in ser. *Rubri*, but also in sect. *Restricti* (in six species classified in all four series of the section (*Halophilici*, *Penicillioides*, *Restricti* and *Vitricolarum*; Sklenář *et al.* (2017)).  

Series ***Chevalierorum*** Houbraken & Frisvad, ***ser*. *nov*.** MycoBank MB832995.  

*Etymology*: Named after the type species of this series, *A*. *chevalieri*.  

*Type*: *Aspergillus chevalieri* (L. Mangin) Thom & Church, The Aspergilli: 111. 1926.  

*Diagnosis*: *Phylogeny*: Series *Chevalierorum* belongs to sect. *Aspergillus*, subgen. *Aspergillus*; this series is phylogenetically sister to series *Aspergillus* and *Rubri* (Fig. 11). *Morphology & physiology*: Colonies restricted on MEA, spreading on low water activity media (*e.g.*, M40Y), yellow or orange; conidiophores uniseriate; all species grow rapid on M60Y at 37 °C, and CY20S and M60Y at 25 °C; all except *A*. *caperatus* and *A*. *costiformis* grow on CY20S at 37 °C. *Sexual morph* eurotium-type, homothallic, yellow; ascospores with high crests (0.5 μm). Series description based on Chen *et al.* (2017).  

*Included species*: *Aspergillus caperatus*, *A*. *chevalieri*, *A*. *costiformis*, *A*. *cristatus*, *A*. *intermedius*, *A*. *montevidensis*, *A*. *porosus*.  

*Extrolites*: All species produce echinulins, neoechinulins, isoechinulins, auroglaucins and flavoglaucins in common with most other species in sect. *Aspergillus*.  

Series ***Leucocarpi*** Houbraken & Frisvad, ***ser*. *nov*.** MycoBank MB832996.  

*Etymology*: Named after the type species of this series, *A*. *leucocarpus*.  

*Type*: *Aspergillus leucocarpus* Hadlok & Stolk, Antonie van Leeuwenhoek 35: 9. 1969.  

*Diagnosis*: *Phylogeny*: Series *Leucocarpi* belongs to sect. *Aspergillus*, subgen. *Aspergillus* and is an early branch in sect. *Aspergillus* (together with ser. *Teporium*) (Fig. 11). *Morphology & physiology*: Colonies restricted; conidial colour *en masse* greyish green or dark green; conidiophores uniseriate; no growth on CY20S at 37 °C, moderate growth on M60Y at 37 °C. S*exual morph* eurotium-type, homothallic, white (without the characteristic yellow colour seen in other series of sect. *Aspergillus*); ascospores in surface view globose to subglobose, spore bodies slightly verruculose, in side view lenticular, furrow pronounced, with scattered protuberances, crests 0.5 μm. Series description based on Chen *et al.* (2017).  

*Included species*: *Aspergillus leucocarpus*.  

*Extrolites*: Echinulins, epiheveadrides, neoechinulins (Chen *et al.* 2017). *Aspergillus leucocarpus* is the only species in sect. *Aspergillus* that does not produce auroglaucins and flavoglaucins.  

Series ***Rubri*** Houbraken & Frisvad, ***ser*. *nov*.** MycoBank MB832997.  

*Etymology*: Named after the type species of this series, *A*. *ruber*.  

*Type*: *Aspergillus ruber* (Jos. König *et al.*) Thom & Church, Aspergillus: 112. 1926.  

*Diagnosis*: *Phylogeny*: Series *Rubri* belongs to sect. *Aspergillus*, subgen. *Aspergillus*; this series forms a well-supported clade together with members of ser. *Aspergillus* (Fig. 11). *Morphology & physiology*: Colonies restricted on MEA, spreading on low water activity media (*e.g.*, M40Y), yellow or orange; conidiophores uniseriate; no growth on CY20S at 37 °C, four species (*A*. *appendiculatus*, *A*. *cumulatus*, *A*. *mallochii*, *A*. *sloanii*) cannot grow on M60Y at 37 °C, rapid growth on M60Y at 25 °C, growth on CY20S at 25 °C (except *A*. *appendiculatus*). *Sexual morph* eurotium-type, homothallic; ascospores non-crested or with reduced crests, the only exception is *A*. *cumulatus*, which produces irregular, low (<0.5 μm) crests. Series description based on Chen *et al.* (2017).  

*Included species*: *Aspergillus appendiculatus*, *A*. *cumulatus*, *A*. *mallochii*, *A*. *microperforatus*, *A*. *pseudoglaucus*, *A*. *ruber*, *A*. *sloanii*, *A*. *tonophilus*, *A*. *zutongqii*.  

*Extrolites*: Like most other species in sect. *Aspergillus*, all species produce echinulins, isoechinulins, neoechinulins, auroglaucins and flavoglaucin (ascomatal metabolites) (Chen *et al.* 2017). *Aspergillus pseudoglaucus* in addition produces mycophenolic acid and asperentins (Séguin et al., 2014, Chen et al., 2017, Mouhamadou et al., 2017). *Aspergillus ruber* produces large amounts of rubrocristin and erythroglaucin, giving the species its red colour (Arai et al., 1989a, Wang et al., 2007b (misidentified as *A*. *variecolor*), Li *et al.* 2017) in addition to asperinines, asperflavin, emodin, physcion, but the latter three are also produced by many other species in sect. *Aspergillus*.  

Series ***Tamarindosolorum*** Houbraken & Frisvad, ***ser*. *nov*.** MycoBank MB832998.  

*Etymology*: Named after the type species of this series, *A*. *tamarindosoli*.  

*Type*: *Aspergillus tamarindosoli* A.J. Chen *et al.*, Stud. Mycol. 88: 123. 2017.  

*Diagnosis*: *Phylogeny*: Series *Tamarindosolorum* belongs to sect. *Aspergillus*, subgen. *Aspergillus*; this series is sister to series *Aspergillus*, *Chevalierorum* and *Rubri* (Fig. 11). *Morphology & physiology*: Colonies restricted on MEA, spreading on low water activity media (*e.g.*, M40Y); conidial colour *en masse* pale green to greyish green; conidiophores uniseriate; no growth on CY20S at 37 °C. S*exual morph* eurotium-type, homothallic, yellow; ascospores hyaline, in surface view globose to subglobose, spore bodies verruculose, in side view lenticular, furrow present, crests irregular, 0.5–1.5 μm. Series description based on Chen *et al.* (2017).  

*Included species*: *Aspergillus tamarindosoli*.  

*Extrolites*: Asperflavin, auroglaucin, bisanthrons, echinulins, emodin, dihydroauroglaucin, epiheveadrides, flavoglaucin, isoechinulins, neoechinulins, physcion, tetrahydroauroglaucin (Chen *et al.* 2017).  

Series ***Teporium*** Houbraken & Frisvad, ***ser*. *nov*.** MycoBank MB832999.  

*Etymology*: Named after the type species of this series, *A*. *teporis*.  

*Type*: *Aspergillus teporis* A.J. Chen *et al.*, Stud. Mycol. 88: 123. 2017.  

*Diagnosis*: *Phylogeny*: Series *Teporium* belongs to sect. *Aspergillus*, subgen. *Aspergillus* and is an early diverging lineage in sect. *Aspergillus* (together with ser. *Leucocarpi*) (Fig. 11). *Morphology & physiology*: Colonies on MEA restricted, spreading on media with low water activity; conidial colour *en masse* greyish green to dark green; conidiophores uniseriate; fast growth on CY20S and M60Y at 37 °C. *Sexual morph* eurotium-type, homothallic, cream yellow; ascospores in surface view globose to subglobose, spore bodies slightly verruculose, in side view lenticular, furrow pronounced, with scattered protuberances, crests 0.5 μm. Series description based on Chen *et al.* (2017).  

*Included species*: *Aspergillus teporis*.  

*Extrolites*: Echinulins, epiheveadrides, isoechinulins, neoechinulins.  

Series ***Xerophili*** Houbraken & Frisvad, ***ser*. *nov*.** MycoBank MB833000.  

*Etymology*: Named after the type species of this series, *A*. *xerophilus*.  

*Type*: *Aspergillus xerophilus* Samson & Mouch., Antonie van Leeuwenhoek 41: 348. 1975.  

*Diagnosis*: *Phylogeny*: Series *Xerophili* belongs to sect. *Aspergillus*, subgen. *Aspergillus*; this series contains early diverging species sister to series *Aspergillus*, *Chevaleriorum*, *Rubri* and *Tamarindosolorum* (Fig. 11). *Morphology & physiology*: No growth on CYA and MEA, colonies spreading on low water activity media; conidial colour undetermined, sporulation absent; conidiophores uniseriate; no growth on CY20S, rapid growth on M60Y; *Aspergillus osmophilus* grows rapidly on M60Y at 37 °C, while *A*. *xerophilus* does not grow under this condition. S*exual morph* eurotium-type, homothallic, yellow; ascospores with low crests (<0.5 μm). Series description based on Chen *et al.* (2017).  

*Included species*: *Aspergillus osmophilus*, *A*. *xerophilus*.  

*Extrolites*: Asperflavin, auroglaucins, flavoglaucin, echinulins and neoechinulins are shared with most other series in sect. *Aspergillus*. Sulochrin is only produced by *A*. *xerophilus* in sect. *Aspergillus* but is also produced by other Aspergilli such as *A*. *terreus* and *A*. *wentii* (Curtis et al., 1970, Assante et al., 1980).  

*Notes on series in sect*. *Aspergillus*: The production of yellow cleistothecia (except in ser. *Leucocarpi*), lenticular ascospores and uniseriate conidiophore heads are characters shared by members of section *Aspergillus*. The species in this section are osmo-, xero- or halotolerant, have a worldwide distribution and are common in indoor air, house dust, cereals, and food products with low water activity (Chen et al., 2017, Visagie et al., 2017). Hubka *et al.* (2013a) recognised three clades in sect. *Aspergillus* (*A*. *chevalieri*-clade, *A*. *glaucus*-clade, *A*. *ruber*-clade) and Chen *et al.* (2017) also included the “*A*. *xerophilus*-clade”. These four clades are treated as series in our study. *Aspergillus cibarius* and *A*. *endophyticus* belong to a large well-supported clade that includes series *Aspergillus* and *Rubri*. The exact relationship of these species remains unresolved because of weakly supported branching within this clade; however, we tentatively classify them in ser. *Aspergillus*. *Aspergillus leucocarpus*, *A*. *tamarindosoli*, *A*. *teporis* each form single-species clades and are here treated as separate series (Fig. 11). The features of the asexual morph and macromorphology of colonies were of less importance to distinguish the various series. Red hyphae were uniformly absent from species of the ser. *Chevaleriorum* but can be absent in members of other series as well (Hubka *et al.* 2013a).

### Section *Restricti*

*Type*: *Aspergillus restrictus* G. Sm., J. Textile Inst. 22: 115. 1931.  

*Description*: See Gams *et al.* (1985) (morphology), Kocsubé *et al.* (2016) (phylogeny), Sklenář *et al.* (2017) (morphology, phylogeny).  

Series ***Halophilici*** Houbraken & Frisvad, ***ser*. *nov*.** MycoBank MB833001.  

*Etymology*: Named after the type species of this series, *A*. *halophilicus*.  

*Type*: *Aspergillus halophilicus* C.M. Chr. *et al.*, Mycologia 51: 636. 1961.  

*Diagnosis*: *Phylogeny*: Series *Halophilici* belongs to sect. *Restricti*, subgen. *Aspergillus*; the phylogenetic position of ser. *Halophilici* is unresolved in Fig. 11, but is closely related to series *Restricti* and *Vitricolarum* in Supplementary Fig. S1. *Morphology & physiology*: No growth on MEA, CYA and M40Y; conidiophores uniseriate, radiate conidial heads (sparsely produced); ser. *Halophilici* are not able to grow on agar media, including those with high sugar (M60Y) or salt (MEA + 10 % NaCl), growth is present on Czapek agar supplemented with 70 % sucrose. *Sexual morph* eurotium-type, homothallic, hyaline to pale yellow, globose to subglobose; ascospores hyaline, lenticular with two equatorial crests. Series description based on Sklenář *et al.* (2017).  

*Included species*: *Aspergillus halophilicus*.  

*Extrolites*: Asperphenamate, cristatin A, echinulin, mycophenolic acid (trace), preechinulin (Micheluz et al., 2016, Sklenář et al., 2017). The white ascomata of *A*. *halophilicus* do not contain auroglaucins, flavoglaucin or anthraquinones, explaining the absence of yellow colours in the ascomata.  

Series ***Penicillioides*** Houbraken & Frisvad, ***ser*. *nov*.** MycoBank MB833002.  

*Etymology*: Named after the type species of this series, *A*. *penicillioides*.  

*Type*: *Aspergillus penicillioides* Speg., Revista Fac. Agron. Univ. Nac. La Plata 2: 246. 1896.  

*Diagnosis*: *Phylogeny*: Series *Penicillioides* belongs to sect. *Restricti*, subgen. *Aspergillus*; the series is sister to the other series in this section, though with strong statistical support in ML (>95 % BS) and poor support in Bayesian analysis (<0.95 pp) (Fig. 11). *Morphology & physiology*: Colonies restricted on MEA and CYA or growth absent, moderate on M40Y; conidiophores uniseriate, with globose conidial heads and sometimes later becoming radiate, stipe surface (SEM) with hairs; no growth on CY20S at 37 °C, members of the series *Penicillioides* are the most xerophilic of the section. *Sexual morph* unknown. Series description based on Sklenář *et al.* (2017).  

*Included species*: *Aspergillus canadensis*, *A*. *clavatophorus*, *A*. *hordei*, *A*. *infrequens*, *A*. *magnivesiculatus*, *A*. *penicillioides*, *A*. *reticulatus*, *A*. *salinicola*, *A*. *tardicrescens*.  

*Extrolites*: Most species produce asperglaucide, while two species produce asperphenamate. One species produces mycophenolic acid and another species produces chrysogine. Three species produce echinulin and two species produce antarone A (Sklenář *et al.* 2017).  

Series ***Restricti*** Houbraken & Frisvad, ***ser*. *nov*.** MycoBank MB833003.  

*Etymology*: Named after the type species of this series, *A*. *restrictus*.  

*Type*: *Aspergillus restrictus* G. Sm., J. Textile Inst. 22: 115. 1931.  

*Diagnosis*: *Phylogeny*: Series *Restricti* belongs to sect. *Restricti*, subgen. *Aspergillus* and is a sister of series *Vitricolarum* (Fig. 11). *Morphology & physiology*: Colonies restricted on MEA and CYA or growth absent, moderate or spreading on M40Y; conidiophores uniseriate, with compact or loosely columnar heads, stipe surface (SEM) with hairs; no growth on CY20S at 37 °C (except *A*. *pachycaulis*). *Sexual morph* unknown. Series description based on Sklenář *et al.* (2017).  

*Included species*: *Aspergillus caesiellus*, *A*. *conicus*, *A*. *destruens*, *A*. *domesticus*, *A*. *gracilis*, *A*. *pachycaulis*, *A*. *pseudogracilis*, *A*. *restrictus*, *A*. *villosus*.  

*Extrolites*: All species produce asperphenamate, while two species produce asperglaucide. Four species produce clavatol, one orthosporins and one fulvic acid analog PI-4. Two species can produce mycophenolic acid (Sklenář *et al.* 2017).  

Series ***Vitricolarum*** Houbraken & Frisvad, ***ser*. *nov*.** MycoBank MB833004.  

*Etymology*: Named after the type species of this series, *A*. *vitricola*.  

*Type*: *Aspergillus vitricola* [as “*vitricolae*”] Ohtsuki, Bot. Mag. (Tokyo) 75: 436. 1962.  

*Diagnosis*: *Phylogeny*: Series *Vitricolarum* belongs to subgen. *Aspergillus*, sect. *Restricti* and is a sister of ser. *Restricti* (Fig. 11). *Morphology & physiology*: Colonies restricted on MEA and CYA or growth absent, moderate on M40Y; conidiophores uniseriate, with radiate heads, stipe surface (SEM) smooth; no growth on CY20S at 37 °C. *Sexual morph* unknown. Series description based on Sklenář *et al.* (2017).  

*Included species*: *Aspergillus glabripes*, *A*. *vitricola*.  

*Extrolites*: *Aspergillus glabripes* produces asperphenamate; *A*. *vitricola* produces asperglaucide, and one isolate produces an orthosporin (Sklenář *et al.* 2017).  

*Notes on series in sect*. *Restricti*: Section *Restricti* members are xerophilic and grow optimally on low water activity substrates (containing high concentrations of sugar or salt). Sklenář *et al.* (2017) recognised five clades (*A*. *conicus*-, *A*. *halophilicus*-, *A*. *penicillioides*-, *A*. *restrictus*-, *A*. *vitricola*-clade) in their monographic study on sect. *Restricti*. In our study, we introduce four series for these five clades. The *A*. *restrictus*- and *A*. *conicus*-clades are combined in ser. *Restricti* because of their unresolved phylogenetic relationship (Fig. 11, Supplementary Fig. S1). The shape of conidial heads can be used to distinguish the series in sect. *Restricti*. Conidial heads of ser. *Restricti* form compact or loose columns, those of ser. *Vitricolarum* are radiate and ser. *Penicillioides* mainly have globose conidial heads, which might become radiate after prolonged incubation (Sklenář *et al.* 2017). Ascomata are only produced in ser. *Halophilici*. Furthermore, ser. *Restricti* species (*e.g*., *A*. *caesiellus*, *A*. *pachycaulis*, *A*. *restrictus*) are less xerophilic compared to ser. *Penicillioides*, which contains the most xerophilic species of the section (*e.g*., *A*. *penicillioides*).  

***Aspergillus* subgen. *Circumdati*** W. Gams *et al.*, Adv. Pen. Asp. Syst.: 59. 1986 [1985]. MycoBank MB832507.  

*Type*: *Aspergillus alutaceus* Berk. & M.A. Curtis, Grevillea 3: 108. 1875 (= *Aspergillus ochraceus*).  

*Description*: See Gams *et al.* (1985) (morphology), Houbraken and Samson, 2011, Kocsubé et al., 2016, this study (Fig. 2) (phylogeny).  

**Section *Candidi*** W. Gams *et al.*, Adv. Pen. Asp. Syst.: 61. 1986 [1985]. MB832512.  

*Type*: *Aspergillus candidus* Link, Mag. Ges. Naturf. Freunde Berlin 3: 16. 1809.  

**Fig. 12:**
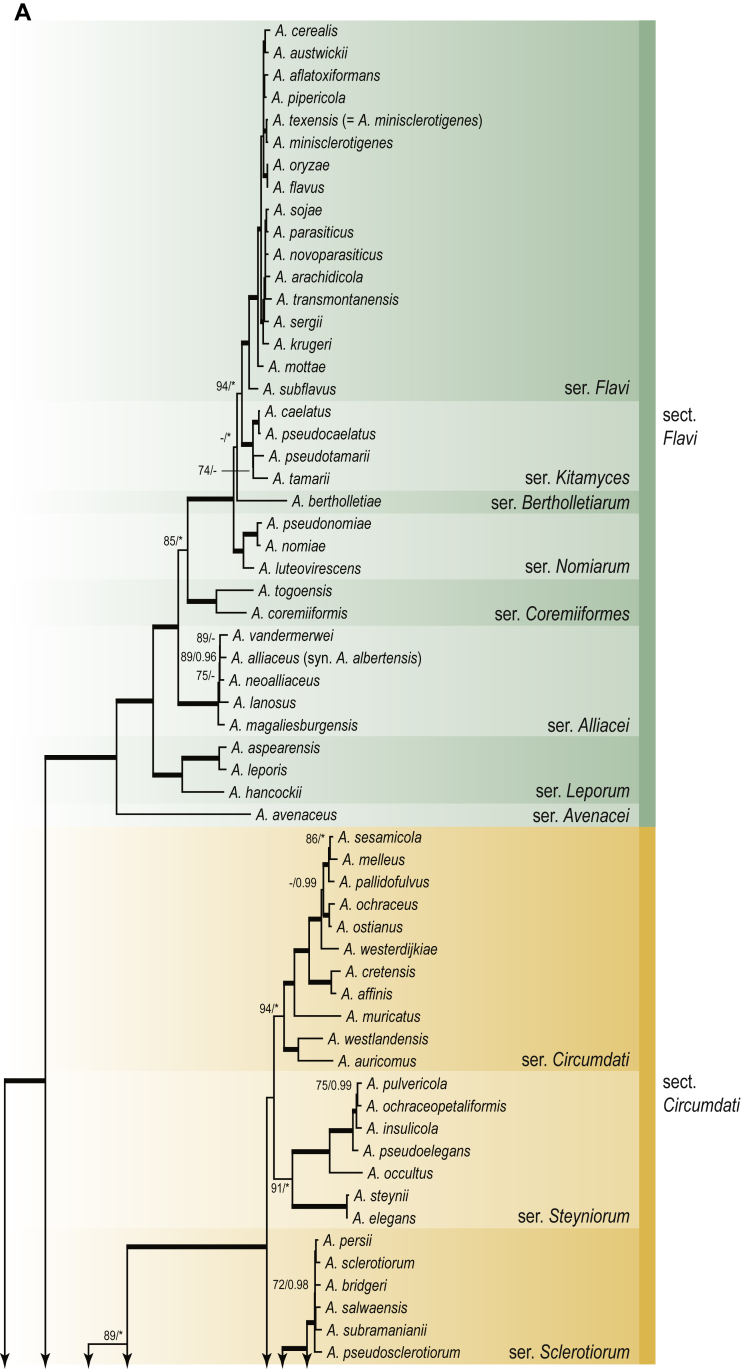

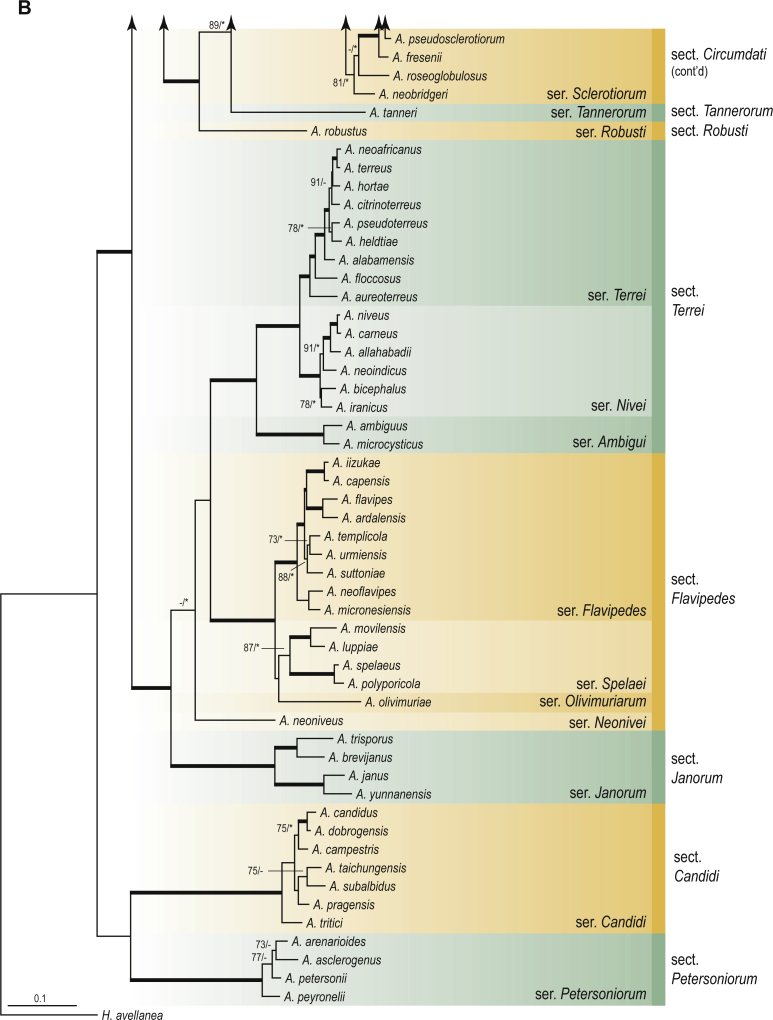
Combined phylogeny for *BenA*, *CaM* and *RPB2* data sets showing the phylogenetic relation of species, series and sections within *Aspergillus* subgen. *Circumdati* (excl. sect. *Nigri*, see Fig. 13). The BI posterior probability (pp) values and bootstrap percentages of the maximum likelihood (ML) analysis are presented at the nodes; fully supported branches are thickened. Values less than 70 % bootstrap support (ML) or less than 0.95 posterior probability (Bayesian analysis) are indicated with a hyphen or not shown. The bar indicates the number of substitutions per site. The phylogram is rooted with *Hamigera avellanea*.

*Description*: *Phylogeny*: Series *Candidi* belongs to subgen. *Circumdati*, sect. *Candidi* and is sister to sect. *Petersoniorum* (Supplementary Fig. S1, Fig. 12). *Morphology & physiology*: Colonies restricted or moderate; conidial colour *en masse* white or yellow; conidiophores biseriate. *Sexual morph* unknown; sclerotia produced by some species, black or purple-black. Also see Peterson et al., 2008, Kocsubé et al., 2016 (phylogeny), Varga et al., 2007b, Hubka et al., 2018b (morphology, phylogeny).

*Included species*: *Aspergillus campestris*, *A*. *candidus*, *A*. *dobrogensis*, *A*. *pragensis*, *A*. *subalbidus*, *A*. *taichungensis*, *A*. *tritici*.  

*Extrolites*: Most species in sect. *Candidi* produce the shikimic acid derived secondary metabolites chloroflavonins, terphenyllins, candidusins and xanthoascins (Hubka *et al.* 2018b), in addition to the terpene-derived taichunins (Kato *et al.* 2018) and the amino acid derived bicyclo [2.2.2]diazaoctane ring containing taichunamides (Kagiyama *et al.* 2016).  

*Notes on sect*. *Candidi*: No subdivision of sect. *Candidi* is proposed, and ser. *Candidi* is only informally introduced here (see Table 4). Section *Petersoniorum* is phylogenetically distant but is the most closely related section (Fig. 2, Jurjević *et al.* 2015), and some species in this section also produce conidia in white or yellow shades. Section *Candidi* species produce predominantly globose vesicles commonly reaching or exceeding a diameter of 20 μm, while the vesicles in sect. *Petersoniorum* species do not exceed 20 μm in diam and are variable in shape (pyriform, subglobose, elongate near angular or penicillium-like). Furthermore, sect. *Candidi* species can produce black or purple-black sclerotia, while the sclerotia in sect. *Petersoniorum* are pale yellow to brown (Jurjević *et al.* 2015).  

**Section *Circumdati*** W. Gams *et al.*, Adv. Pen. Asp. Syst.: 59. 1986 [1985]. MycoBank MB832508.  

*Type*: *Aspergillus alutaceus* Berk. & M.A. Curtis, Grevillea 3: 108. 1875 (= *Aspergillus ochraceus*).  

*Description*: See Gams *et al.* (1985) (morphology), Visagie *et al.* (2014c) (morphology, phylogeny), Steenwyk *et al.* (2019) (genome).  

Series ***Circumdati*** Houbraken & Frisvad, ***ser*. *nov*.** MycoBank MB832987.  

*Etymology*: This series is based on the same type as sect. *Circumdati*, and therefore also named after this section.  

*Type*: *Aspergillus alutaceus* Berk. & M.A. Curtis, Grevillea 3: 108. 1875 (= *Aspergillus ochraceus*).  

*Diagnosis*: *Phylogeny*: Series *Circumdati* belongs to subgen. *Circumdati*, sect. *Circumdati* and is phylogenetically most closely related to ser. *Sclerotiorum*; the node is fully supported in the Bayesian analysis (1.00 pp, data not shown), but bootstrap support is lacking (<70 %, Fig. 12). *Morphology & physiology*: Colonies spreading; conidial colour *en masse* mostly light yellow to ochre; conidiophores biseriate; generally no growth at 37 °C or restricted (<20 mm, 7 d, CYA), some species grow more rapid (*A*. *pallidofulvus*, *A*. *muricatus*). *Sexual morph* generally not observed in culture, except in *A*. *muricatus* (homothallic, neopetromyces-type); sclerotia production common, white, cream or yellow. Series description based on Visagie *et al.* (2014c).  

*Included species*: *Aspergillus affinis*, *A*. *auricomus*, *A*. *cretensis*, *A*. *melleus*, *A*. *muricatus*, *A*. *ochraceus*, *A*. *ostianus*, *A*. *pallidofulvus*, *A*. *sesamicola*, *A*. *westerdijkiae*, *A*. *westlandensis*.  

*Extrolites*: Circumdatins and destruxins are only produced in ser. *Circumdati*.  

Series ***Sclerotiorum*** Houbraken & Frisvad, ***ser*. *nov*.** MycoBank MB832581.  

*Etymology*: Named after the type species of the series, *Aspergillus sclerotiorum*.  

*Type*: *Aspergillus sclerotiorum* G. A. Huber, Phytopathology 23: 306. 1933.  

*Diagnosis*: *Phylogeny*: Series *Sclerotiorum* belongs to subgen. *Circumdati*, sect. *Circumdati* and is phylogenetically sister to series *Circumdati* and *Steyniorum*, though statistical support is lacking (<70 % BS, Fig. 12); The Bayesian analysis posterior probability (pp) positions this series as sister to ser. *Circumdati* (1.00 pp, data not shown). *Morphology & physiology*: Colonies spreading; conidial colour *en masse* mostly light yellow to ochre; conidiophores biseriate; good growth at 37 °C > 20 mm (7 d, CYA), with exception of *A*. *roseoglobulosus* (Visagie *et al.* 2014c). *Sexual morph* unknown; sclerotia production common, white, cream or yellow.  

*Included species*: *Aspergillus bridgeri*, *A*. *fresenii*, *A*. *neobridgeri*, *A*. *persii*, *A*. *pseudosclerotiorum*, *A*. *roseoglobulosus*, *A*. *salwaensis*, *A*. *sclerotiorum*, *A*. *subramanianii*.  

*Extrolites*: This series includes a species, *A*. *persii*, producing aspernidines, cyclopenins, mevinolins (= lovastatins), and sclerotiumins (= aspersclerotiorones) (reported as *A*. *sclerotiorum*) (Phainuphong et al., 2016, Bao et al., 2017, Phainuphong et al., 2017b, Phainuphong et al., 2018b, Lebar et al., 2019). Cyclopenin, radarins, secalonic acid A, secopenitrem D and sulphinines is only found in species in ser. *Sclerotiorum* (Visagie *et al.* 2014c). No species in ser. *Sclerotiorum* produce mellein.  

Series ***Steyniorum*** Houbraken & Frisvad, ***ser*. *nov*.** MycoBank MB832582.  

*Etymology*: Named after the type species of the series, *Aspergillus steynii*.  

*Type*: *Aspergillus steynii* Frisvad & Samson, Stud. Mycol. 50: 39. 2004.  

*Diagnosis*: *Phylogeny*: Series *Steyniorum* belongs to subgen. *Circumdati*, sect. *Circumdati* and is sister to a clade containing ser. *Circumdati*, but this relationship is lacking statistical support (Fig. 12); Bayesian analysis places this series confidently (1.00 pp) as a sister to series *Circumdati* and *Sclerotiorum* (phylogram not shown). *Morphology & physiology*: Colonies spreading; conidial colour *en masse* mostly light yellow to ochre; conidiophores biseriate; generally, no growth at 37 °C or sometimes restricted growth (<20 mm, 7 d, CYA). *Sexual morph* unknown; sclerotia production common, white, cream or yellow. Series description based on Visagie *et al.* (2014c).  

*Included species*: *Aspergillus elegans*, *A*. *insulicola*, *A*. *occultus*, *A*. *ochraceopetaliformis*, *A*. *pseudoelegans*, *A*. *pulvericola*, *A*. *steynii*.  

*Extrolites*: Metabolites only produced by species in ser. *Steyniorum* include antibiotic Y, asteltoxins, cycloechinulin, insulicolides, N-methylepiamauuromine, ochrindols, quinolactacin, and verruculogen TR-2 (Visagie *et al.* 2014c). No species in ser. *Steyniorum* produce aspergamides, mellamides, neohydroxyaspergillic acids or petromurins (Visagie *et al.* 2014c).  

*Notes on series in sect*. *Circumdati*: The extrolites produced in the three series of sect. *Circumdati* are similar and the mycotoxin ochratoxin A is produced in all series of the section. Extrolites include aspergamides (= stephacidins = sclerotiamides) (not yet found in species in ser. *Steyniorum*), aspochracins / sclerotiotides, aspyrones, circumdatins, mellamides, melleins, ochratoxins, orthosporins, penicillic acids, and xanthomegnins (Visagie *et al.* 2014c). The proposed series classification is based on the results of the multigene phylogeny (Fig. 12). Based on a phylogenetic analysis, Visagie *et al.* (2014c) recognised seven main clades in sect. *Circumdati*. One clade included *A*. *robustus* and this clade is raised to section level (sect. *Robusti*; see below) (Jurjević *et al.* 2015). The *A*. *auricomus*-, *A*. *muricatus*-, and A. *ochraceus*-clades *fide* Visagie *et al.* (2014c) are treated here as ser. *Circumdati*. Even though *A*. *auricomus* and *A*. *westlandensis* (together forming the *A*. *auricomus*-clade; Visagie *et al.* 2014c) form a sister clade to the other members of ser. *Circumdati* (Fig. 12), we did not find any additional evidence to treat these two species as a separate series. Series *Circumdati* includes species that produce aspochracins, mellamides, circumdatins and aspergamides; this series apparently lost the ability to produce aspochracins (Visagie *et al.* 2014c). Series *Sclerotiorum* represents the *A*. *fresenii*-clade *fide* Visagie *et al.* (2014c); ser. *Steyniorum* includes the *A*. *ochraceopetaliformis*-clade *fide* Visagie *et al.* (2014c), *A*. *steynii* and *A*. *elegans*. The latter two species were treated as a separate clade (Visagie *et al.* 2014c), and produce TR-2 and cycloechinulin, two extrolites not produced by any other species of sect. *Circumdati*.  

**Section *Flavi*** W. Gams *et al.*, Adv. Pen. Asp. Syst.: 60. 1986 [1985]. MycoBank MB832510.  

*Type*: *Aspergillus flavus* Link, Mag. Ges. Naturf. Freunde Berlin 3: 16. 1809.  

*Description*: See Gams *et al.* (1985) (morphology), Kocsubé *et al.* (2016), this study (Fig. 2) (phylogeny), Frisvad *et al.* (2019) (morphology, phylogeny).  

Series ***Alliacei*** Houbraken & Frisvad, ***ser*. *nov*.** MycoBank MB832583.  

*Etymology*: Named after the type species of the series, *Aspergillus alliaceus*.  

*Type*: *Aspergillus alliaceus* Thom & Church, Aspergilli: 163. 1926.  

*Diagnosis*: *Phylogeny*: Series *Alliacei* belongs to subgen. *Circumdati*, sect. *Flavi* and is sister to a large clade containing series *Bertholletiarum*, *Coremiiformes*, *Flavi*, *Kitamyces* and *Nomiarum* (Fig. 12). *Morphology & physiology*: Colonies spreading; conidial colour *en masse* yellow; conidiophores biseriate; growth at 37 °C, no or poor growth at 42 °C; reverse on AFPA (Aspergillus Flavus and Parasiticus Agar) cream. *Sexual morph* generally not observed in culture, present in *A*. *alliaceus*, petromyces-type, homothallic; sclerotia often present, black. Series description based on Frisvad *et al.* (2019).  

*Included species*: *Aspergillus alliaceus*, *A*. *lanosus*, *A*. *magaliesburgensis*, *A*. *neoalliaceus*, *A*. *vandermerwei*.  

*Extrolites*: Certain secondary metabolites, such as altersolanols, asperlicins, burnettienes / phaeospelides, burnettramic acid, griseofulvins, mevinolins, nalgiovensins / allianthrones, and ochratoxins are only found in ser. *Alliacei* species and not in any other species of sect. *Flavi* (Goetz et al., 1985, Liesch et al., 1985, Liesch et al., 1988, Mandelare et al., 2018, Frisvad et al., 2019, Li et al., 2019a, Morishita et al., 2019).  

Series ***Avenacei*** Houbraken & Frisvad, ***ser*. *nov*.** MycoBank MB832584.  

*Etymology*: Named after the type species of the series, *Aspergillus avenaceus*.  

*Type*: *Aspergillus avenaceus* G. Sm., Trans. Brit. Mycol. Soc. 26: 24. 1943.  

*Diagnosis*: *Phylogeny*: Series *Avenacei* belongs to subgen. *Circumdati*, sect. *Flavi*, encompassing the earlier diverging species to all other series of sect. *Flavi* (Fig. 12). *Morphology & physiology*: Colonies spreading; conidial colour *en masse* beige; conidiophores biseriate; growth at 37 °C, no growth at 42 °C; reverse on AFPA cream. *Sexual morph* unknown; sclerotia often present, large, black. Series description based on Frisvad *et al.* (2019).  

*Included species*: *Aspergillus avenaceus*.  

*Extrolites*: *Aspergillus avenaceus* is the only species in sect. *Flavi* that does not produce kojic acid, but isolates in the species can produce avenaciolides, and in common with other species in sect. *Flavi* altersolanols, aspirochlorins, pseurotin A and 3-nitropropionic acid (Frisvad *et al.* 2019).  

Series ***Bertholletiarum*** Houbraken & Frisvad, ***ser*. *nov*.** MycoBank MB832988.  

*Etymology*: Named after the type species of the series, *Aspergillus bertholletiae*.  

*Type*: *Aspergillus bertholletiae* [as “*bertholletius*”] Taniwaki *et al.*, PLoS ONE 7: e42480, 6. 2012.  

*Diagnosis*: *Phylogeny*: Series *Bertholletiarum* belongs to subgen. *Circumdati*, sect. *Flavi* and is sister to a clade containing series *Flavi*, *Kitamyces* and *Nomiarum* (Fig. 12). *Morphology & physiology*: Colonies spreading; conidial colour *en masse* brown; conidiophores biseriate; good growth at 37 °C, no growth at 42 °C; reverse on AFPA cream. *Sexual morph* unknown; sclerotia not observed in culture. Species associated with coconut trees. Series description based on Frisvad *et al.* (2019).  

*Included species*: *Aspergillus bertholletiae*.  

*Extrolites*: In common with species from other series in sect. *Flavi*, *A*. *bertholletiae* produces cyclopiazonic acid, kojic acid, 3-O-methylsterigmatocystin, parasiticolides, tenuazonic acid and ustilaginoidin C (Frisvad *et al.* 2019).  

Series ***Coremiiformes*** Houbraken & Frisvad, ***ser*. *nov*.** MycoBank MB832585.  

*Etymology*: Named after the type species of the series, *Aspergillus coremiiformis*.  

*Type*: *Aspergillus coremiiformis* Bartoli & Maggi, Trans. Brit. Mycol. Soc. 71: 386. 1979.  

*Diagnosis*: *Phylogeny*: Series *Coremiiformes* belongs to subgen. *Circumdati*, sect. *Flavi* and is sister to a clade containing series *Bertholletiarum*, *Flavi*, *Kitamyces* and *Nomiarum* (Fig. 12). *Morphology & physiology*: Colonies spreading; conidial colour *en masse* orange-brown; conidiophores biseriate; No growth at 37 °C, synnemata present; reverse on AFPA cream. *Sexual morph* unknown; sclerotia not observed in culture. Series description based on Frisvad *et al.* (2019).  

*Included species*: *Aspergillus coremiiformis*, *A*. *togoensis*.  

*Extrolites*: In common with species in other series in sect. *Flavi*, *A*. *togoensis* produces aflatoxin B_1_, a bisiderin, paspaline, paspalinine, paxillin and sterigmatocystin, while the other species in the series (*A*. *coremiiformis*) is quite different and only produces unknown indole alkaloids (Frisvad *et al.* 2019).  

Series ***Flavi*** Houbraken & Frisvad, ***ser*. *nov*.** MycoBank MB832989.  

*Etymology*: Named after the type species of the series, *Aspergillus flavus*.  

*Type*: *Aspergillus flavus* Link, Mag. Ges. Naturf. Freunde Berlin 3: 16. 1809.  

*Diagnosis*: *Phylogeny*: Series *Flavi* belongs to subgen. *Circumdati*, sect. *Flavi* and is sister to ser. *Kitamyces* (Fig. 12). *Morphology & physiology*: Colonies spreading; conidial colour *en masse* mostly yellow-green, occasionally brown (*A*. *oryzae*), or brownish green (*A*. *parasiticus*); conidiophores uni- and/or biseriate; good growth at 37 °C, generally growth at 42 °C, except *A*. *mottae* and *A*. *subflavus*; reverse on AFPA orange, except in *A*. *oryzae*. *Sexual morph* generally not observed in culture, except in *A*. *flavus* and *A*. *parasiticus*, petromyces-type; heterothallic; sclerotia often present, black. Most species are primarily associated with nuts and oil-seeds, but will also grow on foods from domesticated plants such as cereals (*e.g*. maize) and dry fruits. *Aspergillus oryzae* is the domesticated form of *A*. *flavus*, and *A*. *sojae* of *A*. *parasiticus*; both are used in food fermentations. Series description based on Frisvad *et al.* (2019).  

*Included species*: *Aspergillus aflatoxiformans*, *A*. *arachidicola*, *A*. *austwickii*, *A*. *cerealis*, *A*. *flavus*, *A*. *krugeri*, *A*. *minisclerotigenes*, *A*. *mottae*, *A*. *novoparasiticus*, *A*. *oryzae*, *A*. *parasiticus*, *A*. *pipericola*, *A*. *sergii*, *A*. *sojae*, *A*. *subflavus*, *A*. *transmontanensis*.  

*Extrolites*: Most species produces aflatoxins (B and G types) (and precursors such as versicolorins and sterigmatocystins), aflatrems, aflavarins, aflavazols, aflaviniones, asparasones, asperfurans, aspergillic acids, aspergillomarasmins, aspirochlorins, chrysogines, citreoisocoumarins, cyclopiamides, cyclopiazonic acids (and the related speradins), ditryptophenalines, kojic acids, kojistatins, leporins, miyakamides (= oryzamides), 3-nitropropionic acid, parasitenone, parasiticolides, parasiticols, parasperones, penicillins, sporogens, and ustilaginoidins (Frisvad *et al.* 2019).  

Series ***Kitamyces*** Houbraken & Frisvad, ***ser*. *nov*.** MycoBank MB832586.  

*Etymology*: Named after G. Kita, who described *A*. *tamarii*, the type species of this series.  

*Type*: *Aspergillus tamarii* Kita, Centralbl. Bakteriol. 2. Abth. 37: 433. 1913.  

*Diagnosis*: *Phylogeny*: Series *Kitamyces* belongs to subgen. *Circumdati*, sect. *Flavi* and is sister to ser. *Flavi* (Fig. 12). *Morphology & physiology*: Colonies spreading; conidial colour *en masse* in shades of brown; conidiophores biseriate; good growth at 37 °C, no growth at 42 °C; reverse on AFPA dark brown. *Sexual morph* not observed in culture; sclerotia occasionally present, black. Series description based on Frisvad *et al.* (2019).  

*Included species*: *Aspergillus caelatus*, *A*. *pseudocaelatus*, *A*. *pseudotamarii*, *A*. *tamarii*.  

Series ***Leporum*** Houbraken & Frisvad, ***ser*. *nov*.** MycoBank MB832587.  

*Etymology*: Named after the type species of the series, *Aspergillus leporis*.  

*Type*: *Aspergillus leporis* States & M. Chr., Mycologia 58: 738. 1966.  

*Diagnosis*: *Phylogeny*: Series *Leporum* belongs to subgen. *Circumdati*, sect. *Flavi* and is sister to a large clade containing series *Alliacei*, *Bertholletiarum*, *Coremiiformes*, *Flavi*, *Kitamyces* and *Nomiarum*. *Morphology & physiology*: Colonies spreading; conidial colour *en masse* yellow-green with a shade of beige, beige or olive; conidiophores biseriate; growth at 37 °C, no growth at 42 °C; reverse on AFPA cream. *Sexual morph* not observed in culture; sclerotia often present, large, black. *Aspergillus leporis* is dung-associated, while *A*. *aspearensis* and *A*. *hancockii* have been reported to be soil-borne. It is not known whether the two latter species are actually dung-associated. Series description based on Frisvad *et al.* (2019).  

*Included species*: *Aspergillus aspearensis*, *A*. *hancockii*, *A*. *leporis*.  

*Extrolites*: Aflavarins, aflavinines, antibiotic Y, clavatols, dehydroterrestric acid, eupenifeldin, fumitremorgins, hancockiamides, 7-hydroxytrichothecolone, kojic acid, leporines, leporizines, mevinolins, onychocins, paspalines, pseurotins, speradins (Frisvad *et al.* 2019). The aflavarins, aflavinines, paspalinines, pseurotins, speradins and kojic acid have been found in other series in sect. *Flavi*, but antibiotic Y, dehydroterrestric acid, eupenifeldin, fumitremorgins, hancockiamides, 7- hydroxytrichothecolone, leporizines, mevinolins and onychocins have only been found in ser. *Leporum*.  

Series ***Nomiarum*** Houbraken & Frisvad, ***ser*. *nov*.** MycoBank MB832588.  

*Etymology*: Named after the type species of the series, *Aspergillus nomiae*.  

*Type*: *Aspergillus nomiae* Kurtzman *et al.*, Antonie van Leeuwenhoek 53: 151. 1987.  

*Diagnosis*: *Phylogeny*: Series *Nomiarum* belongs to subgen. *Circumdati*, sect. *Flavi* and is sister to a large clade containing series *Alliacei*, *Bertholletiarum*, *Coremiiformes*, *Flavi*, *Kitamyces* and *Nomiarum* (Fig. 12). *Morphology & physiology*: Colonies spreading; conidial colour *en masse* (dark) yellow-green; conidiophores biseriate; good growth at 37 °C, no growth at 42 °C; reverse on AFPA cream orange. *Sexual morph* generally not observed in culture, present in *A*. *nomiae*, petromyces-type, heterothallic; sclerotia often present, bullet-shaped, black. Species primarily associated to bees. Series description based on Frisvad *et al.* (2019).  

*Included species*: *Aspergillus luteovirescens*, *A*. *nomiae*, *A*. *pseudonomiae*.  

*Extrolites*: Aflatoxins (B and G type) (and precursors), altersolanols, anominine, aspernomine, aspergillic acids, chrysogines, kojic acid, miyakamides, paspaline, paspalinine, pseurotins, sporogens, and tenuazonic acid (Frisvad et al., 2018, Frisvad et al., 2019).  

*Notes on series in sect*. *Flavi*: Using a multigene phylogenetic analysis, Frisvad *et al.* (2019) recognised eight clades in sect. *Flavi*. These clades are treated here as separate series. The majority of species belonging to the phylogenetically related series *Flavi*, *Nomiarum* and *Kitamyces* produce aflatoxin B and G. Non-aflatoxin producers are *A*. *oryzae*, *A*. *sojae* and *A*. *subflavus* in ser. *Flavi*, *A*. *caelatus* and *A*. *tamarii* in ser. *Kitamyces*, and *A*. *pseudotamarii* only produces aflatoxin B. Series *Bertholletiarum* can produce the aflatoxin precursor O-methylsterigmatocystin, but not aflatoxins. Series *Coremiiformes* is phylogenetically a sister to series *Flavi*, *Kitamyces*, *Bertholletiarum* (Fig. 12) and includes a species (*A*. *togoensis*) that produces aflatoxin B (Frisvad *et al.* 2019). Species in ser. *Alliacei* are able to produce ochratoxins, unlike species in other series of sect. *Flavi*. Other extrolites only found in sect. *Circumdati* ser. *Alliacei* are altersolanols, asperlicins, burnettiene, burnettramic acid, griseofulvin. Series *Avenacei* and *Leporum* are early branching clades in sect. *Flavi* (Fig. 7, Frisvad *et al.* 2019), but do not have the ability to produce aflatoxins or ochratoxins. The sole species in ser. *Avenacei* (*A*. *avenaceus*) does not produce kojic acid, an extrolite produced by the majority of species in sect. *Flavi* (Frisvad *et al.* 2019).  

**Section *Flavipedes*** W. Gams *et al.*, Adv. Pen. Asp. Syst.: 59. 1986 [1985]. MycoBank MB832506.  

*Type*: *Aspergillus flavipes* (Bainier & Sartory) Thom & Church, Aspergilli: 155. 1926.  

*Description*: See Hubka *et al.* (2015) (morphology, phylogeny), Kocsubé *et al.* (2016) (phylogeny).  

Series ***Flavipedes*** Houbraken & Frisvad, ***ser*. *nov*.** MycoBank MB832990.  

*Etymology*: Named after the type species of the series, *Aspergillus flavipes*.  

*Type*: *Aspergillus flavipes* (Bainier & Sartory) Thom & Church, Aspergilli: 155. 1926.  

*Diagnosis*: *Phylogeny*: Series *Flavipedes* belongs to subgen. *Circumdati*, sect. *Flavipedes* and is phylogenetically sister to series *Olivimuriarum* and *Spelaei* (Fig. 12). *Morphology & physiology*: Colonies growing moderately fast; conidial colour *en masse* white, pale brown, or yellow-brown; conidiophores biseriate; good growth at 37 °C, some species grow on CYA at 40 °C (*e.g*., *A*. *ardalensis*, *A*. *neoflavipes*, *A*. *templicola* (reported under *A*. *mangaliensis*), all grow on M40Y at 40 °C. *Sexual morph* generally not observed in culture, except in *A*. *neoflavipes*, fennellia-type; sclerotia not observed in culture. Series description based on Hubka *et al.* (2015).  

*Included species*: *Aspergillus ardalensis*, *A*. *capensis*, *A*. *flavipes*, *A*. *iizukae*, *A*. *micronesiensis*, *A*. *neoflavipes*, *A*. *suttoniae*, *A*. *templicola*, *A*. *urmiensis*.  

*Extrolites*: Aspochalasins, cytochalasins, flavipins, flaviphenalenones and geodins.  

Series ***Neonivei*** Houbraken & Frisvad, ***ser*. *nov*.** MycoBank MB832589.  

*Etymology*: Named after the type species of the series, *Aspergillus neoniveus*.  

*Type*: *Aspergillus neoniveus* Samson *et al.*, Stud. Mycol. 69: 53. 2011.  

*Diagnosis*: *Phylogeny*: Series *Neonivei* belongs to subgen. *Circumdati*, sect. *Flavipedes*; the phylogenetic relationship of this series is unresolved (more information, see Notes section *Flavipedes*). *Morphology & physiology*: Colonies growing moderately fast; conidial colour *en masse* white; conidiophores biseriate. *Sexual morph* fennellia-type, yellow, orange-yellow. Series description based on Samson *et al.* (2011a) and Hubka *et al.* (2015).  

*Included species*: *Aspergillus neoniveus*.  

*Extrolites*: Aspochalamins, citreoviridin and paspalinine are produced by *A. neoniveus*, the sole species in the series (Samson *et al.* 2011a).  

Series ***Olivimuriarum*** Houbraken & Frisvad, ***ser*. *nov*.** MycoBank MB835555.  

*Etymology*: Named after the type species of the series, *Aspergillus olivimuriae*.  

*Type*: *Aspergillus olivimuriae* S.W. Peterson & S. Crognale, Int. J. Syst. Evol. Microbiol. 69: 2901. 2019.  

*Diagnosis*: *Phylogeny*: Series *Olivimuriarum* belongs to subgen. *Circumdati*, sect. *Flavipedes* and is phylogenetically sister to ser. *Spelaei*, though statistical support is lacking (Fig. 12); Bayesian analysis confidently shows that this series is sister to series *Flavipedes* and *Spelaei* (1.00 pp, data not shown). *Morphology & physiology*: Colonies growing moderately fast; conidial colour *en masse* avellaneous; conidiophores biseriate; moderate growth at 37 °C, no growth on CYA at 40 °C. *Sexual morph* unknown; sclerotia not observed in culture. Series description based on*et al.* Crognale *et al.* (2019).  

*Included species*: *Aspergillus olivimuriae*.  

*Extrolites*: Extrolite production by the sole species in the series has not been performed.  

Series ***Spelaei*** Houbraken & Frisvad, ***ser*. *nov*.** MycoBank MB832590.  

*Etymology*: Named after the type species of the series, *Aspergillus spelaeus*.  

*Type*: *Aspergillus spelaeus* A. Nováková *et al.*, Mycologia 107: 194. 2015.  

*Diagnosis*: *Phylogeny*: Series *Spelaei* belongs to subgen. *Circumdati*, sect. *Flavipedes* and is phylogenetically sister to series *Olivimuriarum* and *Flavipedes* (Fig. 12). *Morphology & physiology*: Colonies growing moderately fast; conidial colour *en masse* white, pale brown, or yellow-brown; conidiophores biseriate; no or moderate growth at 37 °C, generally no growth on CYA and M40Y at 40 °C. *Sexual morph* unknown; sclerotia not observed in culture. Series description based on Hubka *et al.* (2015).  

*Included species*: *Aspergillus luppiae*, *A*. *movilensis*, *A*. *polyporicola*, *A*. *spelaeus*.  

*Extrolites*: Aspochalasins and curvularins can be produced by ser. *Spelaei* taxa.  

*Notes on series in sect*. *Flavipedes*: Hubka *et al.* (2015) recognised two main clades in sect. *Flavipedes* and named them the *A*. *flavipedes*- and *A*. *spelaeus*-clade. These results are confirmed here and the series names *Flavipedes* and *Spelaei* are introduced for those phylogenetically related clades (Fig. 12). These two series in sect. *Flavipedes* can be differentiated based on growth rates on CYA incubated at 37 and 40 °C. Furthermore, extrolites from species in ser. *Flavipedes* include aspochalasins, cytochalasins, flavipins, geodins and flaviphenalenones and ser. *Spelaei* species can produce aspochalasins and curvularins. The phylogenetic position of ser. *Neonivei* is uncertain. Our phylogenetic analysis based on *BenA*, *CaM* and *RPB2* sequences (Fig. 12) shows that this series is basal in sections *Terrei* and *Flavipedes*; however, this relationship lacks bootstrap support, but is fully supported in the Bayesian analysis. Our nine-gene phylogeny (Supplementary Fig. S1) positions ser. *Neonivei* in sect. *Flavipedes* (89 % BS). In the past, *A*. *neoniveus* (syn. *Fennellia nivea*) was classified in sections *Janorum*, *Terrei* and *Flavipedes* (Peterson, 2008, Peterson et al., 2008, Hubka et al., 2015, Jurjević et al., 2015), but never with high statistical support. We decided to include ser. *Neonivei* in sect. *Flavipedes* based on the result of our 9-gene phylogeny and future research using genome sequence data will probably resolve the exact position of this series.  

**Section *Janorum*** [as “*Jani*”] Hubka *et al.*, Mycologia 107: 197. 2015. MycoBank MB832532.  

*Type*: *Aspergillus janus* Raper & Thom, Mycologia 36: 556. 1944.  

*Description*: *Phylogeny*: Section *Janorum* belongs to subgen. *Circumdati*. *Morphology & physiology*: Colonies growing restricted; conidial colour *en masse* in shades of green and white; conidiophores variable, 1) tall, white sporulating, biseriate conidiophores with pyriform to clavate vesicles, producing smooth, uncoloured conidia; 2) lower, green sporulating, biseriate conidiophores with commonly pyriform vesicles producing green, echinulate conidia; 3) micro- to semimacronematous conidiophores producing globose, elliptical or clavate conidia, that are commonly truncate; no (*A*. *brevijanus*) or restricted (*A*. *janus*) growth at 37 °C, no growth at 40 °C. *Sexual morph* unknown. Also see Hubka *et al.* (2015) (morphology, phylogeny), Kocsubé *et al.* (2016) (phylogeny).  

*Included species*: *Aspergillus brevijanus*, *A*. *janus*, *A*. *trisporus*, *A*. *yunnanensis*.  

*Extrolites*: Asperphenamate, brevicompanins and janoxepin are found in sect. *Janorum*.  

*Notes*: Because no subdivision of sect. *Janorum* is proposed, ser. *Janorum* is only informally introduced here. Section *Janorum* is phylogenetically related to sections *Terrei* and *Flavipedes* (Fig. 2). Species belonging to this section produce three types of conidiophores and conidia, and colonies have green and white sectors making them distinctive (Hubka *et al.* 2015).  

**Section *Nigri*** W. Gams *et al.*, Adv. Pen. Asp. Syst.: 60. 1986 [1985]. MycoBank MB832511.  

*Type*: *Aspergillus niger* Tiegh., Ann. Sci. Nat., Bot., ser. 5, 8: 240. 1867; *nom*. *cons*. (Kozakiewicz *et al.* 1992).  

*Description*: See Gams *et al.* (1985) (morphology), Samson et al., 2007b, Varga et al., 2011 (morphology, phylogeny), Kocsubé *et al.* (2016) (phylogeny), Vesth et al., 2018, Steenwyk et al., 2019 (genome analysis).  

*Notes*: The phylogenomic analysis of Steenwyk *et al.* (2019) shows that section *Nigri* does not belong to subgen. *Circumdati* and that it is more closely related to subgen. *Nidulantes*. The species in subgen. *Nidulantes* are phenotypically distinct from sect. *Nigri* species, indicating that this section represents a separate subgenus. Based on phenotypic and extrolite data, and our phylogenetic analysis (Fig. 1, Fig. 2), we decided to maintain sect. *Nigri* in subgen. *Circumdati* until more (genome) data supporting the analysis of Steenwyk *et al.* (2019) becomes available.  

Series ***Carbonarii*** Houbraken & Frisvad, ***ser*. *nov*.** MycoBank MB832591.  

*Etymology*: Named after the type species of the series, *Aspergillus carbonarius*.  

*Type*: *Aspergillus carbonarius* (Bainier) Thom, J. Agric. Res. 7: 12. 1916.  

**Fig. 13:**
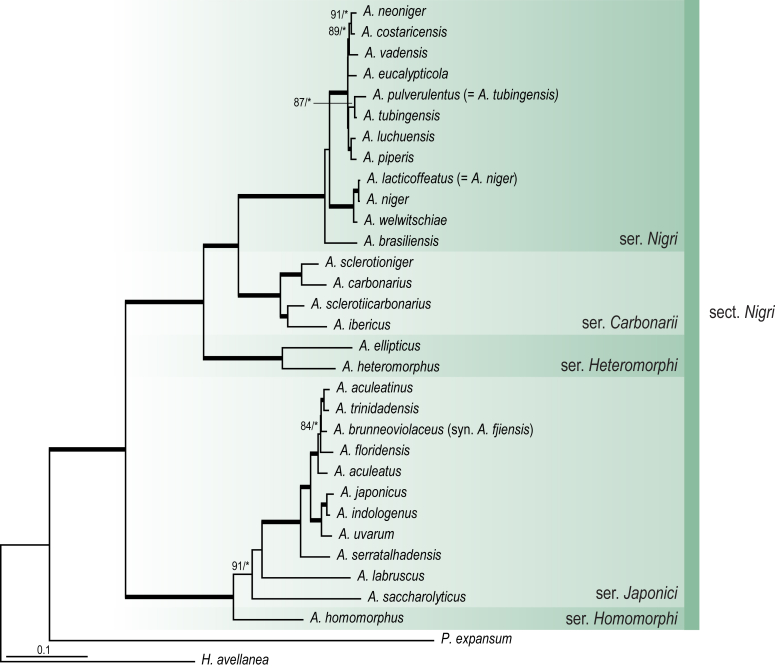
Combined phylogeny for *BenA*, *CaM* and *RPB2* data sets showing the phylogenetic relation of species and series within *Aspergillus* sect. *Nigri*. The BI posterior probability (pp) values and bootstrap percentages of the maximum likelihood (ML) analysis are presented at the nodes; fully supported branches are thickened. Values less than 70 % bootstrap support (ML) or less than 0.95 posterior probability (Bayesian analysis) are indicated with a hyphen or not shown. The bar indicates the number of substitutions per site. The phylogram is rooted with *Hamigera avellanea* and *Penicillium expansum*.

*Diagnosis*: *Phylogeny*: Series *Carbonarii* belongs to subgen. *Circumdati*, sect. *Nigri* and is phylogenetically sister to ser. *Nigri* (Fig. 13). *Morphology & physiology*: Colonies spreading; conidial colour *en masse* black or blackish brown; conidiophores biseriate; generally weak or no growth at 37 °C (except *A*. *ibericus*); conidia rough-walled, large, 6–9 μm. *Sexual morph* generally not produced in culture, except in *A*. *sclerotiicarbonarius*, saitoa-type, heterothallic, orange to red-brown. Series description based on Samson *et al.* (2007b) and Varga *et al.* (2011).

*Included species*: *Aspergillus carbonarius*, *A*. *ibericus*, *A*. *sclerotiicarbonarius*, *A*. *sclerotioniger*.  

*Extrolites*: Citric acid, oxalic acid, gluconic acid, atromentins, ochratoxins, funalenone, naphtho-γ-pyrones and pyranonigrins are shared by two or more species in ser. *Carbonarii*.  

Series ***Heteromorphi*** Houbraken & Frisvad, ***ser*. *nov*.** MycoBank MB832592.  

*Etymology*: Named after the type species of the series, *Aspergillus heteromorphus*.  

*Type*: *Aspergillus heteromorphus* Bat. & H. Maia, Anais Soc. Biol. Pernambuco 15: 200. 1957.  

*Diagnosis*: *Phylogeny*: Series *Heteromorphi* belongs to subgen. *Circumdati*, sect. *Nigri* and is phylogenetically sister to a clade containing series *Carbonarii* and *Nigri* (Fig. 13). *Morphology & physiology*: Colonies spreading; conidial colour *en masse* black or blackish brown; conidiophores biseriate; no growth at 37 °C; conidia 3–5 μm. *Sexual morph* unknown; sclerotia can be induced. Series description based on Samson *et al.* (2007b) and Varga *et al.* (2011).  

*Included species*: *Aspergillus ellipticus*, *A*. *heteromorphus*.  

*Extrolites*: Atromentins, austdiol, candidusins, terphenyllins, and xanthoascin.  

Series ***Homomorphi*** Houbraken & Frisvad, ***ser*. *nov*.** MycoBank MB832593.  

*Etymology*: Named after the type species of the series, *Aspergillus homomorphus*.  

*Type*: *Aspergillus homomorphus* Steiman *et al.* ex Samson & Frisvad, Stud. Mycol. 50: 58. 2004.  

*Diagnosis*: *Phylogeny*: Series *Homomorphi* belongs to subgen. *Circumdati*, sect. *Nigri* and is phylogenetically sister to ser. *Japonici* (Fig. 13). *Morphology & physiology*: Colonies spreading; conidial colour *en masse* black or blackish brown; conidiophores biseriate; growth at 37 °C, no growth at 40 °C; conidia 5–7 μm, with spiny ornamentation. *Sexual morph* unknown; sclerotia not observed in culture. Series description based on Steiman et al., 1995, Samson et al., 2007b and Varga *et al.* (2011).  

*Included species*: *Aspergillus homomorphus*.  

*Extrolites*: Asperflavin, atromentins, decaturins, dehydrocarolic acid, homomorphosins, styrylpyrones.  

Series ***Japonici*** Houbraken & Frisvad, ***ser*. *nov*.** MycoBank MB834295.  

*Etymology*: Named after the type species of the series, *Aspergillus japonicus*.  

*Type*: *Aspergillus japonicus* Saito, Bot. Mag. (Tokyo) 20: 61. 1906.  

*Diagnosis*: *Phylogeny*: Series *Japonici* belongs to subgen. *Circumdati*, sect. *Nigri* and is phylogenetically sister to ser. *Homomorphi* (Fig. 13). *Morphology & physiology*: Colonies spreading; conidial colour *en masse* black or blackish brown; conidiophores uniseriate; no growth at 37 °C; conidia 3–6 μm. *Sexual morph* generally not produced in culture, except in a species described as “*Saitoa japonica*” (Rajendran & Muthappa 1980); sclerotia often present. Series description based on Samson et al., 2007b, Varga et al., 2011 and Hubka & Kolarik (2012).  

*Included species*: *Aspergillus aculeatinus*, *A*. *aculeatus*, *A*. *assiutensis* (accepted species, unpubl. data, XC Wang), *A*. *brunneoviolaceus*, *A*. *floridensis*, *A*. *indologenus*, *A*. *japonicus*, *A*. *labruscus*, *A*. *saccharolyticus*, *A*. *serratalhadensis*, *A*. *trinidadensis*, *A*. *uvarum*.  

*Extrolites*: Aculene A, aflavinins, asperflavin, aspergillimide, calbistrin C, emodin, neopyranopnigrin, neoxaline, okaramin X, pre-aurantiamin, secalonic acid D.  

Series ***Nigri*** Houbraken & Frisvad, ***ser*. *nov*.** MycoBank MB832991.  

*Etymology*: Named after the type species of the series, *Aspergillus niger*.  

*Type*: *Aspergillus niger* Tiegh., Ann. Sci. Nat., Bot., ser. 5, 8: 240. 1867; *nom*. *cons*. (Kozakiewicz *et al.* 1992).  

*Diagnosis*: *Phylogeny*: Series *Nigri* belongs to subgen. *Circumdati*, sect. *Nigri* and is phylogenetically sister to ser. *Carbonarii* (Fig. 13). *Morphology & physiology*: Colonies spreading; conidial colour *en masse* black or blackish brown; conidiophores biseriate; good growth at 37 and 40 °C; conidia (2.5–)3–5 μm. *Sexual morph* generally not produced in culture, except in *A*. *tubingensis*, saitoa-type, heterothallic (Horn *et al.* 2013); sclerotia produced in most species. Series description based on Samson *et al.* (2007b) and Varga *et al.* (2011).  

*Included species*: *Aspergillus brasiliensis*, *A*. *costaricensis*, *A*. *eucalypticola*, *A*. *luchuensis*, *A*. *neoniger*, *A*. *niger*, *A*. *piperis*, *A*. *tubingensis*, *A*. *vadensis*, *A*. *welwitschiae*.  

*Extrolites*: Citric acid, oxalic acid, gluconic acid, tensyuic acids, atromentins, ochratoxins, funalenone, fumonisins, kotanins, yanuthones, naphtho-γ-pyrones, tensidols, malformins, nigragillins, pyranonigrins, asperazines, aflavinins (only in sclerotia) are shared by two or more species in ser. *Nigri*.  

*Notes on series in sect*. *Nigri*: Frisvad *et al.* (2007) suggested a series (and subseries) classification for section *Nigri* using morphological, chemical and physiological features. Their suggested provisional series classification fits with our suggested phylogenetic-based classification. Fig. 13 shows the phylogenetic relationship among the species of section *Nigri* based on partial *BenA*, *CaM* and *RPB2* gene sequencing. These relationships are similar to those presented in Vesth *et al.* (2018) using genome sequence data. In their manuscript, they distinguished the *A*. *niger*- and the *A*. *tubingensis*-clade. In contrast, Varga *et al.* (2011) treated these two clades as one, named the *A*. *niger*-clade. We follow Varga *et al.* (2011) in our concept of ser. *Nigri*, because species of this series share the ability to grow well at 37 and 40 °C and form biseriate conidiophores and similar-sized conidia measuring (2.5–)3–5 μm. Furthermore, the extrolites shared by two or more species in series *Nigri* are citric acid, oxalic acid, gluconic acid, tensyuic acids, atromentins, ochratoxins, funalenone, fumonisins, kotanins, yanuthones, naphtho-γ-pyrones, tensidols, malformins, nigragillins, pyranonigrins, asperazines, aflavinins (only in sclerotia) (Nielsen et al., 2009, Varga et al., 2011, Frisvad et al., 2018, Vesth et al., 2018). Series *Carbonarii* is characterised by a generally weak or no growth at 37 °C (except *A*. *ibericus*) and production of large conidia measuring 6–9 μm. Extrolites shared by two or more species in ser. *Carbonarii* include citric acid, oxalic acid, gluconic acid, atromentins, ochratoxins, funalenone, naphtho-γ-pyrones and pyranonigrins (Nielsen et al., 2009, Varga et al., 2011). Series *Japonici* is a sister series of ser. *Homomorphi*. Series *Homomorphi* include species that have biseriate conidiophores and produce the extrolites homomorphosins and decaturins, while ser. *Japonici* species have uniseriate conidiophores and produce aculenes, asperparalines, calbistrins, neoxalines, okaramins, pre-aurantiamine, and/or secalonic acids. The phylogenetic relationship of *A*. *labruscus* and *A*. *saccharolyticus* with the other species of ser. *Japonici* is unclear (Fig. 13). In contrast to our results, Fungaro *et al.* (2017) classified *A*. *labruscus*, *A*. *homomorphus* and *A*. *saccharolyticus* in the *A*. *homomorphus*-clade and a similar result was shown in Crous *et al.* (2018b, Fungal Planet 720). Based on genome sequence analysis, *A*. *saccharolyticus* is sister to the other ser. *Japonici* species, confirming the result of our 3-gene phylogeny. Unfortunately, *A*. *labruscus* and *A*. *serratalhadensis* were not included in the genome study of Vesth *et al.* (2018) and genome sequencing of these species might reveal the correct classification of these species in the future.  

**Section *Petersoniorum*** [as “*Petersonii*”] Jurjević & Hubka, Pl. Syst. Evol. 301: 2449. 2015. MycoBank MB832533.  

*Type*: *Aspergillus petersonii* Jurjević & Hubka, Pl. Syst. Evol. 301: 2454. 2015.  

*Description*: *Phylogeny*: Section *Petersoniorum* belongs to subgen. *Circumdati* and the phylogenetic relationship with other *Aspergillus* sections needs to be resolved (see Notes below). *Morphology & physiology*: Colonies restricted; conidial colour *en masse* in shades of green; conidiophores biseriate; no growth 40 °C. *Sexual morph* unknown; sclerotia produced in most species (except *A*. *asclerogenus*), globose to ellipsoidal, pale yellow to brown. Also see Jurjević *et al.* (2015) (morphology, phylogeny).  

*Included species*: *Aspergillus arenarioides*, *A*. *asclerogenus*, *A*. *petersonii*, *A*. *peyronelii*.  

*Notes on sect*. *Petersoniorum*: Because no subdivision of sect. *Petersoniorum* is proposed, ser. *Petersoniorum* is only informally introduced here. In Fig. 12, this section is an early diverging clade in subgen. *Circumdati*; however, a more thorough analysis places this section most close to sect. *Candidi* (Fig. 2) confirming the results of Jurjević *et al.* (2015). Sections *Petersoniorum* and *Candidi* can be differentiated by their differences in conidial and sclerotial colour (for more details, see sect. *Candidi*).  

**Section *Robusti*** Jurjević & Hubka, Pl. Syst. Evol. 301: 2460. 2015. MycoBank MB814443.  

*Type*: *Aspergillus robustus* M. Chr. & Raper, Mycologia 70: 200. 1978.  

*Description*: *Phylogeny*: Section *Robusti* belongs to subgen. *Circumdati* and is phylogenetically sister to sections *Tannerorum* and *Circumdati*. *Morphology & physiology*: Colonies restricted; conidial colour *en masse* in shades of yellow; conidiophores biseriate; no growth 37 °C. *Sexual morph* unknown; sclerotia produced, black (Christensen & Raper 1978). See also: Jurjević *et al.* (2015) (morphology, phylogeny).  

*Included species*: *Aspergillus robustus*.  

*Notes*: Because no subdivision of sect. *Robusti* is proposed, ser. *Robusti* is only informally introduced here. Section *Robusti* is a single species section. This section is phylogenetically sister to sections *Tannerorum* and *Circumdati* (Fig. 2, Fig. 12). It differs from both sections by the production of black coloured sclerotia and phototropic conidiophores (Visagie *et al.* 2014c).  

**Section *Tannerorum*** [as “*Tanneri*”] Jurjević & Hubka, Pl. Syst. Evol. 301: 2460. 2015. MycoBank MB832534.  

*Type*: *Aspergillus tanneri* Kwon-Chung *et al.*, J. Clin. Microbiol. 50: 3312. 2012.  

*Description*: *Phylogeny*: Section *Tannerorum* belongs to subgen. *Circumdati* and is sister to sect. *Circumdati*. *Morphology & physiology*: Colonies restricted; sporulation sparse; conidiophores biseriate; good growth 37 °C. *Sexual morph* unknown; sclerotia not observed in culture. Also see Jurjević *et al.* (2015) (morphology, phylogeny).  

*Included species*: *Aspergillus tanneri*.  

*Extrolites*: No extrolites are reported for *A*. *tanneri*, the sole species in this section.  

*Notes*: No subdivision of sect. *Tannerorum* is proposed and ser. *Tannerorum* is only informally introduced here (Table 4). Section *Tannerorum* is a single species section. This section is phylogenetically sister to sect. *Circumdati* (Fig. 2, Fig. 12). It differs from this series by its small pyriform vesicles, lack of sclerotia, very poor sporulation, uncoloured reverse of colonies without production of soluble pigments and better growth at 37 °C than at 25 °C (Jurjević *et al.* 2015).  

**Section *Terrei*** W. Gams *et al.*, Adv. Pen. Asp. Syst.: 59. 1986 [1985]. MycoBank MB832505.  

*Type*: *Aspergillus terreus* Thom, Amer. J. Bot. 5: 85. 1918.  

*Description*: See Gams *et al.* (1985) (morphology), Samson *et al.* (2011a) (morphology, phylogeny), Kocsubé *et al.* (2016) (phylogeny).  

Series ***Ambigui*** Houbraken & Frisvad, ***ser*. *nov*.** MycoBank MB832594.  

*Etymology*: Named after the type species of the series, *Aspergillus ambiguus*.  

*Type*: *Aspergillus ambiguus* Sappa, Allionia 2: 254. 1955.  

*Diagnosis*: *Phylogeny*: Series *Ambigui* belongs to subgen. *Circumdati*, sect. *Terrei* and is phylogenetically sister to series *Nivei* and *Terrei*. *Morphology & physiology*: Colonies restricted; conidial colour *en masse* white, cream, dull yellow or grey-green; conidiophores biseriate. *Sexual morph* unknown. Series description based on Raper & Fennell (1965).  

*Included species*: *Aspergillus ambiguus*, *A*. *microcysticus*.  

*Extrolites*: Butryolactone, terrequinone A.  

Series ***Nivei*** Houbraken & Frisvad, ***ser*. *nov*.** MycoBank MB832595.  

*Etymology*: Named after the type species of the series, *Aspergillus niveus*.  

*Type*: *Aspergillus niveus* Blochwitz, Ann. Mycol. 27: 205. 1929.  

*Diagnosis*: *Phylogeny*: Series *Nivei* belongs to subgen. *Circumdati*, sect. *Terrei* and is phylogenetically most closely related to ser. *Terrei*. *Morphology & physiology*: Colonies growing moderately fast; conidial colour *en masse* white, vinaceous fawn, blue-green; conidiophores biseriate; moderate growth at 37 °C. *Sexual morph* unknown. Series description based on Raper & Fennell (1965) and Samson *et al.* (2011a).  

*Included species*: *Aspergillus allahabadii*, *A*. *bicephalus*, *A*. *carneus*, *A*. *iranicus*, *A*. *neoindicus*, *A*. *niveus*.  

*Extrolites*: Aszonalenins, citrinins are shared by ser. *Nivei* species.  

Series ***Terrei*** Houbraken & Frisvad, ***ser*. *nov*.** MycoBank MB832992.  

*Etymology*: Named after the type species of the series, *Aspergillus terreus*.  

*Type*: *Aspergillus terreus* Thom, Amer. J. Bot. 5: 85. 1918.  

*Diagnosis*: *Phylogeny*: Series *Terrei* belongs to subgen. *Circumdati*, sect. *Terrei* and is phylogenetically most closely related to ser. *Nivei*. *Morphology & physiology*: Colonies spreading; conidial colour *en masse* (light) olive-brown; conidiophores biseriate; good growth at 37 °C. *Sexual morph* generally not produced in culture, except in *A*. *terreus*, heterothallic, fennellia-type. Series description based on Samson *et al.* (2011a).  

*Included species*: *Aspergillus alabamensis*, *A*. *aureoterreus*, *A*. *citrinoterreus*, *A*. *floccosus*, *A*. *heldtiae*, *A*. *hortae*, *A*. *neoafricanus*, *A*. *pseudoterreus*, *A*. *terreus*.  

*Extrolites*: Series *Terrei* extrolites include acetylaranotins, ardeemins, aspergillamides, aspergillicins, aspulvinones, asterriquinones, aszonalenins, butyrolactones, citreoviridins, citrinins, cytochalasins, geodins, gregatins, mevinolins, terrecyclic acids, terreic acid, terreins, terremides, terrequinones, terretonins and territrems (Samson *et al.* 2011a).  

*Notes on series in sect*. *Terrei*: Three series are introduced in sect. *Terrei*: series *Ambigui*, *Nivei and Terrei*. Series *Terrei* and *Nivei* are sister series and the species in those series differ in their conidial colour. Species in ser. *Terrei* generally produce conidia in brown shades, while ser. *Nivei* are in shades of yellow, vinaceous fawn or white. The two species of ser. *Ambigui* grow slower than the taxa of series *Terrei* and *Nivei*.  

***Aspergillus* subgen. *Cremei*** Samson *et al.*, Stud. Mycol. 85: 210. 2016. MycoBank MB819182.  

*Type*: *Aspergillus cremeus* Kwon-Chung & Fennell, Gen. Aspergillus: 418. 1965.  

*Description*: See Kocsubé *et al.* (2016) (morphology, phylogeny).  

**Section *Cremei*** W. Gams *et al.*, Adv. Pen. Asp. Syst.: 61. 1986 [1985]. MycoBank MB832513.  

*Type*: *Aspergillus cremeoflavus* Samson & W. Gams, Adv. Pen. Asp. Syst.: 37. 1986 [1985]. MycoBank MB114701 (= *Aspergillus cremeus*).  

*Description*: See Gams *et al.* (1985) (morphology, and partial sect. *Wentii*, see Notes below), Kocsubé *et al.* (2016), Fig. 2, this study (phylogeny).  

Series ***Arxiorum*** Houbraken & Frisvad, ***ser*. *nov*.** MycoBank MB833044.  

*Etymology*: Named after the type species of this series, *A*. *arxii*.  

*Type*: *Aspergillus arxii* (Fort & Guarro) Houbraken *et al.*, Stud. Mycol. 78: 154. 2014.  

**Fig. 14:**
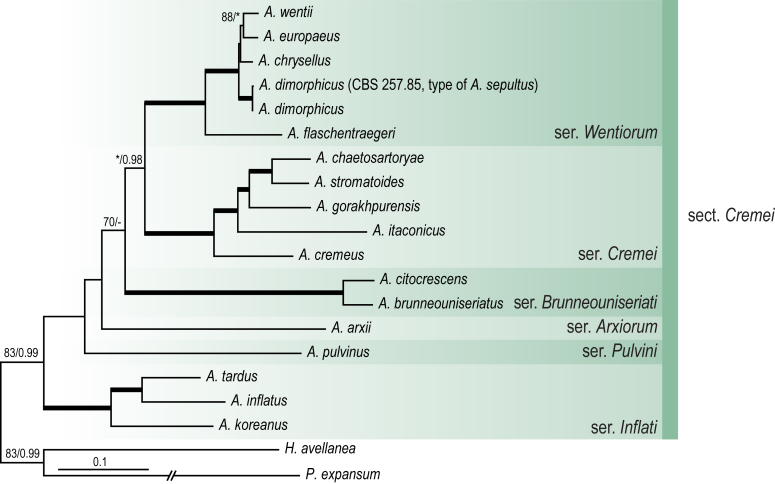
Combined phylogeny for *BenA*, *CaM* and *RPB2* data sets showing the phylogenetic relation of species and series within *Aspergillus* subgen. *Cremei*. The BI posterior probability (pp) values and bootstrap percentages of the maximum likelihood (ML) analysis are presented at the nodes; fully supported branches are thickened. Values less than 70 % bootstrap support (ML) or less than 0.95 posterior probability (Bayesian analysis) are indicated with a hyphen or not shown. The bar indicates the number of substitutions per site. The phylogram is rooted with *Hamigera avellanea* and *Penicillium expansum*.

*Diagnosis*: *Phylogeny*: Series *Arxiorum* belongs to sect. *Cremei*, subgen. *Cremei* and is phylogenetically sister to a clade containing series *Brunneouniseriati*, *Cremei* and *Wentiorum*, though without statistical support (Fig. 14). *Morphology & physiology*: Colonies restricted; conidiophores and conidia not observed in culture. *Sexual morph* chaetosartorya-type, homothallic; ascospores hyaline, ellipsoidal, with two equatorial ridges, convex surfaces finely rugose. Series description based on Fort & Guarro (1984).

*Included species*: *Aspergillus arxii*.  

*Extrolites*: No known extrolites have been found in *Aspergillus arxii*.  

Series ***Brunneouniseriati*** Houbraken & Frisvad, ***ser*. *nov*.** MycoBank MB833045.  

*Etymology*: Named after the type species of this series, *A*. *brunneouniseriatus*.  

*Type*: *Aspergillus brunneouniseriatus* Suj. Singh & B.K. Bakshi, Trans. Brit. Mycol. Soc. 44: 160. 1961.  

*Diagnosis*: *Phylogeny*: Series *Brunneouniseriati* belongs to sect. *Cremei*, subgen. *Cremei* and is phylogenetically sister to a clade containing series *Cremei* and *Wentiorum*, though with moderate (BS = 70 %) or poor (pp < 0.95) statistical support (Fig. 14). *Morphology & physiology*: Colonies growing rapidly; conidia *en masse* grey, (dark) olive-brown or grey-green; conidiophores uniseriate, stipes hyaline, smooth. *Sexual morph* unknown. Series description based on Raper & Fennell (1965) and Crous *et al.* (2015).  

*Included species*: *Aspergillus brunneouniseriatus*, *A*. *citocrescens*.  

*Extrolites*: No known extrolites have been found in series *Brunneouniseriati*.  

Series ***Cremei*** Houbraken & Frisvad, ***ser*. *nov*.** MycoBank MB833046.  

*Etymology*: Named after the type species of this series, *A*. *cremeus*.  

*Type*: *Aspergillus cremeus* Kwon-Chung & Fennell, Gen. Aspergillus: 418. 1965.  

*Diagnosis*: *Phylogeny*: Series *Cremei* belongs to sect. *Cremei*, subgen. *Cremei* and is phylogenetically sister to ser. *Wentiorum* (Fig. 14). *Morphology & physiology*: Colonies varying from restricted to spreading, more rapid growth on agar media with reduced water activity; conidia *en masse* in shades of green; conidiophores biseriate (*A*. *cremeus*, *A*. *gorakhpurensis*), uni- and biseriate (*A*. *stromatoides*) or strictly uniseriate (*A*. *itaconicus*), stipes hyaline, smooth, long. *Sexual morph* unknown (*A*. *gorakhpurensis*, *A*. *itaconicus*, *A*. *stromatoides*) or observed in culture (*A*. *chaetosartoryae*, *A*. *cremeus*), ascomata consisting of several layers of thick-walled hyphae, the outer layer becoming dematiaceous, homothallic; ascospores with prominent equatorial ridges, convex surface with spines. Series description based on Raper and Fennell, 1965, Kamal and Bhargava, 1969 and Wiley & Simmons (1973).  

*Included species*: *Aspergillus chaetosartoryae*, *A*. *cremeus*, *A*. *gorakhpurensis*, *A*. *itaconicus*, *A*. *stromatoides*.  

*Extrolites*: Only *A*. *itaconicus* has been examined for secondary metabolites according to the literature. This species can produce chrysogine (reported here), itaconic acid (Kinoshita, 1932, Steiger et al., 2013), itaconitin (Nakajima *et al.* 1964) and sorbicillins (reported here).  

Series ***Inflati*** (Stolk & Samson) Houbraken & Frisvad, ***comb*. *nov*.** MycoBank MB833047.

*Basionym*: *Penicillium* ser. *Inflata* Stolk & Samson, Adv. Pen. Asp. Syst.: 174. 1986 [1985].  

*Type*: *Penicillium inflatum* Stolk & Malla, Persoonia 6: 197. 1971. (syn. *Aspergillus inflatus*).  

*Diagnosis*: *Phylogeny*: Series *Inflati* belongs to sect. *Cremei*, subgen. *Cremei* and is phylogenetically sister to all other series in sect. *Cremei*. *Morphology & physiology*: Colonies restricted (*A*. *inflatus*) or spreading (*A*. *koreanus*), conidia *en masse* grey-green, greyish olive or grey-brown; conidiophores bi- or triseriate, or penicillium-like and biverticillate divaricate branched, stipe hyaline or pale reddish, smooth or finely roughened. *Sexual morph* unknown. Series description based on (Stolk and Malla, 1971, Bissett and Widden, 1984, Hyde et al., 2016).  

*Extrolites*: *Aspergillus inflatus* and a putative new species tentatively named *A*. *oregonensis* (CBS 576.95A&B) in ser. *Inflati* can produce sterigmatocystin (Rank *et al.* 2011). Otherwise sterigmatocystin (and aflatoxins) has only been found in the subgenera *Circumdati* (sect. *Flavi*) and *Nidulantes* in the genus *Aspergillus* (Chen et al., 2016a, Hubka et al., 2016a, Frisvad et al., 2019).  

*Included species*: *Aspergillus inflatus*, *A*. *koreanus*, *A*. *tardus*.  

Series ***Pulvini*** Houbraken & Frisvad, ***ser*. *nov*.** MycoBank MB833048.  

*Etymology*: Named after the type species of this series, *A*. *pulvinus*.  

*Type*: *Aspergillus pulvinus* Kwon-Chung & Fennell, Gen. Aspergillus: 455. 1965.  

*Diagnosis*: *Phylogeny*: Series *Pulvini* belongs to sect. *Cremei*, subgen. *Cremei* and is phylogenetically sister to a clade containing series *Arxiorum*, *Brunneouniseriati*, *Cremei* and *Wentiorum*, though without statistical support (Fig. 14). *Morphology & physiology*: Colonies spreading, conidia *en masse* blue-green; conidiophores biseriate, stipes brownish pigmented, roughened, long, thick-walled. *Sexual morph* unknown. Series description based on Raper & Fennell (1965).  

*Included species*: *Aspergillus pulvinus*.  

*Extrolites*: No known extrolites have been found in *Aspergillus pulvinus*.  

Series ***Wentiorum*** Houbraken & Frisvad, ***ser*. *nov*.** MycoBank MB833049.  

*Etymology*: Named after the type species of this series, *A*. *wentii*.  

*Type*: *Aspergillus wentii* Wehmer, Centralbl. Bakteriol., 2. Abth., 2: 149. 1896.  

*Diagnosis*: *Phylogeny*: Series *Wentiorum* belongs to sect. *Cremei*, subgen. *Cremei* and is phylogenetically sister to ser. *Cremei*. *Morphology & physiology*: Colonies growing restrictedly on MEA and CYA, growing moderately or rapidly on agar media with reduced water activity; conidia *en masse* in shades of yellow-brown, olive-brown or greyish yellow; conidiophores biseriate, sometimes uniseriate (*A*. *flaschentraegeri*), stipes hyaline, smooth or at most slightly roughened, often long and thick-walled; no growth at 37 °C. *Sexual morph* unknown (*A*. *europaeus*, *A*. *dimorphicus*, *A*. *flaschentraegeri*, *A*. *wentii*) or present (*A*. *chrysellus*), chaetosartorya-type, homothallic; ascospores hyaline, lenticular, with two equatorial ridges, convex surfaces with spines. Series description based on Raper and Fennell, 1965, Tuthill and Christensen, 1986 and Hubka *et al.* (2016b).  

*Included species*: *Aspergillus chrysellus*, *A*. *dimorphicus*, *A*. *europaeus*, *A*. *flaschentraegeri*, *A*. *wentii*.  

*Extrolites*: All species in ser. *Wentiorum* produce asperflavin, emodin, physcion, emodin bianthrone, physcion bianthrone (and other bianthrons), sulochrin and other sulochrins, and wentilacton A and B, except *A*. *flaschentraegeri* which only produces asperflavin, physcion and physcion bisanthron. These and many related extrolites have been reported from *A*. *wentii* and *A*. *europaeus* (Wells et al., 1975, Assante et al., 1979, Assante et al., 1980, Dorner et al., 1980, Xu et al., 2015, Hubka et al., 2016b, Du et al., 2018, Li et al., 2018b, Form et al., 2019). The metabolites have not been detected yet in any species in the other series in subgen. *Cremei*.  

*Notes on sect*. *Cremei and included series*: Raper & Fennell (1965) introduced the *A*. *wentii* and the *A*. *cremeus* group and later Gams *et al.* (1985) formally introduced these as sections *Wentii* and *Cremei*. All species classified by Raper & Fennell (1965) in sect. *Cremei* (*A*. *cremeus*, *A*. *chrysellus*, *A*. *flaschentraegeri*, *A*. *itaconicus*, *A*. *stromatoides*) are still here accepted in this section. Phylogenetic data demonstrated that sect. *Wentii* was superfluous (Peterson, 1995, Peterson, 2008). *Aspergillus wentii* is included in sect. *Cremei* and the other members of the *A*. *wentii* group (*fide* Raper & Fennell 1965) belong to sect. *Flavi*: *A*. *terricola* (= *A*. *tamarii*), *A*. *terricola* var. *americana* (= *A*. *parasiticus*), *A*. *terricola* var. *indicus* (= *A*. *tamarii*), *A*. *thomii* (= *A*. *flavus*) (Frisvad *et al.* 2019). Mainly based on molecular data, sect. *Cremei* expanded to 17 species, which are classified in six series (*Arxiorum*, *Brunneouniseriati*, *Cremei*, *Inflati*, *Pulvini*, *Wentiorum*). Series *Inflati* includes three species and two of those produce penicillium-like conidiophores. *Aspergillus inflatus* was originally described in *Penicillium*. Another species of this series, *A*. *tardus*, was assigned to the *A*. *versicolor* group (Bissett & Widden 1984), but the original description mentioned the non-synchronously production of metulae, a character frequently observed in *Penicillium* and not common in *Aspergillus*. Series *Pulvini* includes a species that produces blue-green coloured conidia, a unique feature in sect. *Cremei*. Series *Cremei* and *Wentiorum* are phylogenetically and phenotypically related. The conidia in ser. *Cremei* are more often in shades of green, while those of ser. *Wentiorum* are more often in shades of yellow-brown. Both series contain species that reproduce sexually. The sole taxon classified in series *Arxiorum* also reproduces sexually, but an asexual morph is not described. Series *Brunneouniseriati* includes two species that both have uniseriate conidial heads. Uniseriate heads are also produced by two species in other series: *A*. *flaschentraegeri* (ser. *Wentiorum*) and *A*. *itaconicus* (series *Cremei*). Regarding extrolites the six series in subgenus and section *Cremei* are remarkably different.  

***Aspergillus* subgen. *Fumigati*** W. Gams, M. Chr., Onions, Pitt & Samson, Adv. Pen. Asp. Syst.: 56. 1986 [1985]. MycoBank MB832495.  

*Type*: *Aspergillus fumigatus* Fresen., Beitr. Mykol. 3: 81. 1863.  

*Description*: See Gams *et al.* (1985) (morphology); Samson *et al.* (2007a) (morphology, phylogeny); Peterson et al., 2008, Kocsubé et al., 2016, Fig. 1, this study (phylogeny).  

**Section *Clavati*** W. Gams *et al.*, Adv. Pen. Asp. Syst.: 57. 1986 [1985]. MycoBank MB832500.  

*Type*: *Aspergillus clavatus* Desm., Ann. Sci. Nat., Bot., ser. 2, 2: 71. 1834.  

*Description*: *Phylogeny*: Series *Clavati* belongs to subgen. *Fumigati*. *Morphology & physiology*: *Colonies* growing restrictedly or moderately rapid; conidial colour *en masse* blue-green; *conidiophores* uniseriate, often with clavate vesicles; restricted growth at 37 °C, except *A*. *acanthosporus* (no growth). *Sexual morph* not observed in culture, or present, neosartorya-type, homothallic (*A*. *acanthosporus*) or heterothallic (*A*. *clavatus*). Section description based on Varga *et al.* (2007a) and Ojeda-López *et al.* (2018).  

**Fig. 15:**
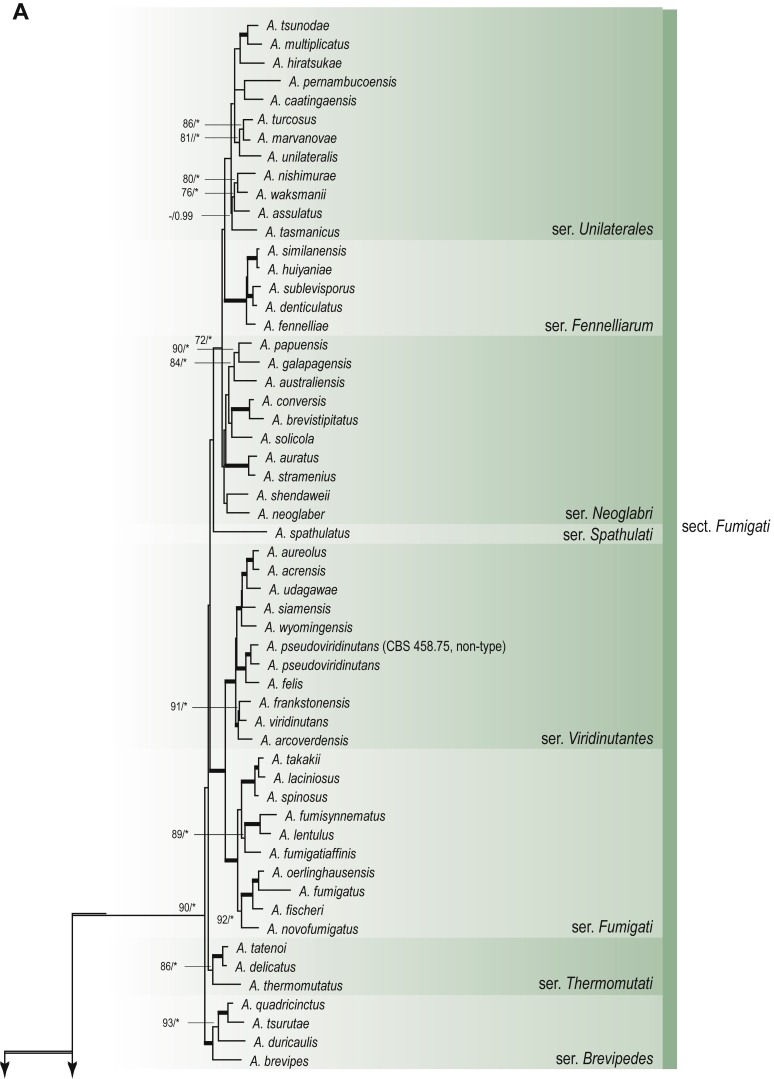

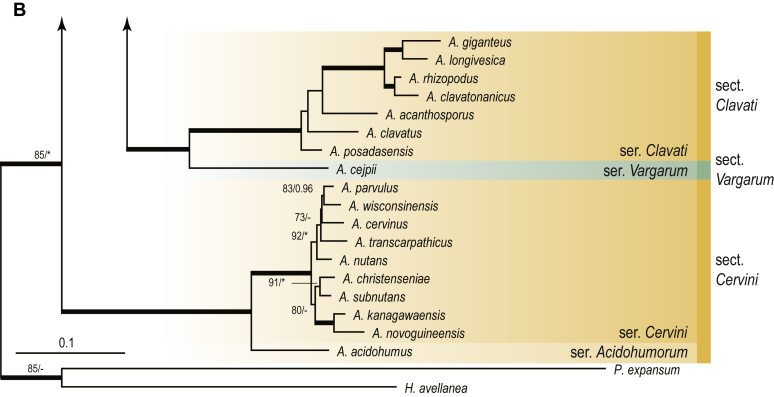
Combined phylogeny for *BenA*, *CaM* and *RPB2* data sets showing the phylogenetic relation of species, series and sections within *Aspergillus* subgen. *Fumigati*. The BI posterior probability (pp) values and bootstrap percentages of the maximum likelihood (ML) analysis are presented at the nodes; fully supported branches are thickened. Values less than 70 % bootstrap support (ML) or less than 0.95 posterior probability (Bayesian analysis) are indicated with a hyphen or not shown. The bar indicates the number of substitutions per site. The phylogram is rooted with *Hamigera avellanea*.

*Included species*: *Aspergillus acanthosporus*, *A*. *clavatonanicus*, *A*. *clavatus*, *A*. *giganteus*, *A*. *longivesica*, *A*. *posadasensis*, *A*. *rhizopodus*, *A*. *seifertii*∗ [∗ not included in Fig. 15; for more details, see Visagie & Houbraken (2020)].

*Extrolites*: At least six species (no data for *A*. *posadasensis* and *A*. *seifertii*) in the section produce ribotoxins (Varga & Samson 2008) and tryptoquivalines / tryptoquivalones (Varga *et al.* 2007a). Kotaninins are produced by four of six species (*A*. *acanthosporus*, *A*. *clavatonanicus*, *A*. *clavatus*, *A*. *rhizopodus*), patulin by three (*A*. *clavatus*, *A*. *giganteus*, *A*. *longivesica*) and antafumicins by four (*A*. *clavatonanicus*, *A*. *clavatus*, *A*. *giganteus*, *A*. *longivesica*). Pyripyropens are produced by two species (*A. longivesica* and *A. giganteus*). Ribotoxins are shared with several species in sect. *Fumigati*, including *A*. *fischeri*, *A*. *fumigatus*, *A*. *neoglaber* and *A viridinutans* (Martínez-Ruiz et al., 1999, Olombrada et al., 2014), while the isolate of *A*. *restrictus* producing restrictocin and regulin was an *A*. *fumigatus* (NRRL 3050 = ATCC 34475). Except for a claim that *A*. *oryzae* can produce ribotoxins (Machida *et al.* 2005), producers of these small insecticidal and antifungal proteins (Olombrada et al., 2014, Citores et al., 2018) are concentrated in subgen. *Fumigati*, sect. *Clavati* and ser. *Fumigati*.  

*Notes*: No subdivision of sect. *Clavati* is proposed, and ser. *Clavati* is only informally introduced here (Table 4).  

**Section *Cervini*** W. Gams *et al.*, Adv. Pen. Asp. Syst.: 56. 1986 [1985]. MycoBank MB832497.  

*Type*: *Aspergillus cervinus* Massee, Bull. Misc. Inform. Kew 1914: 158. 1914.  

*Description*: See Gams *et al.* (1985) (morphology), Kocsubé *et al.* (2016) (phylogeny), Chen *et al.* (2016c) (morphology, phylogeny).  

Series ***Acidohumorum*** Houbraken & Frisvad, ***ser*. *nov*.** MycoBank MB832596.  

*Etymology*: Named after the type species of the series, *Aspergillus acidohumus*.  

*Type*: *Aspergillus acidohumus* A.J. Chen *et al.*, Stud. Mycol. 85: 71. 2016.  

*Diagnosis*: *Phylogeny*: Series *Acidohumorum* belongs to sect. *Cervini*, subgen. *Fumigati*. *Morphology & physiology*: Colonies restricted; conidial colour *en masse* dark fawn; conidiophores uniseriate; no growth at 37 °C. *Sexual morph* unknown. Series description based on Chen *et al.* (2016c).  

*Included species*: *Aspergillus acidohumus*.  

*Extrolites*: No extrolites have been found in the species in ser. *Acidohumorum*.  

Series ***Cervini*** Houbraken & Frisvad, ***ser*. *nov*.** MycoBank MB832993.  

*Etymology*: Named after the type species of the series, *Aspergillus cervinus*.  

*Type*: *Aspergillus cervinus* Massee, Bull. Misc. Inform. Kew 1914: 158. 1914.  

*Diagnosis*: *Phylogeny*: Series *Cervini* belongs to sect. *Cervini*, subgen. *Fumigati*. *Morphology & physiology*: Colonies growing moderately fast; conidial colour *en masse* fawn; conidiophores uniseriate; generally no or very poor growth at 37 °C. *Sexual morph* unknown. Series description based on Chen *et al.* (2016c).  

*Included species*: *Aspergillus cervinus*, *A*. *christenseniae*, *A*. *kanagawaensis*, *A*. *novoguineensis*, *A*. *nutans*, *A*. *parvulus*, *A*. *subnutans*, *A*. *transcarpaticus*, *A*. *wisconsinensis*.  

*Extrolites*: Six of nine species produce terremutin, four of nine produce aspervenone, and three of nine produce 4-hydroxymellein (Chen *et al.* 2016c).  

*Notes*: *Aspergillus acidohumus* is the sole species in ser. *Acidohumorum*. This series clearly belongs to section *Cervini*, but is phylogenetically distant from ser. *Cervini*. Series *Acidohumorum* is phenotypically distinct from other species by its very slow growth rate.  

**Section *Fumigati*** W. Gams *et al.*, Adv. Pen. Asp. Syst.: 56. 1986 [1985]. MycoBank MB832496.  

*Type*: *Aspergillus fumigatus* Fresen., Beitr. Mykol. 3: 81. 1863.  

*Description*: See Gams *et al.* (1985) (morphology); Peterson et al., 2008, Kocsubé et al., 2016, Fig. 1, Fig. 2, this study (phylogeny); Samson *et al.* (2007a) (morphology, phylogeny).  

Series ***Brevipedes*** Houbraken & Frisvad, ***ser*. *nov*.** MycoBank MB832597.  

*Etymology*: Named after the type species of the series, *Aspergillus brevipes*.  

*Type*: *Aspergillus brevipes* G. Sm., Trans. Brit. Mycol. Soc. 35: 241. 1952.  

*Diagnosis*: *Phylogeny*: Series *Brevipedes* belongs to sect. *Fumigati*, subgen. *Fumigati*; the series is well-supported; however, the phylogenetic relationship of the series with other series of the section remains unresolved (Fig. 15). *Morphology & physiology*: Colonies growing moderately fast or spreading; conidial colour *en masse* blue-green; conidiophores uniseriate; good growth at 37 °C. *Sexual morph* not observed in culture, or present, neosartorya-type, homothallic, white, yellowish white or pale yellow. Series description based on Samson *et al.* (2007a) and Hubka *et al.* (2017).  

*Included species*: *Aspergillus brevipes*, *A*. *duricaulis*, *A*. *quadricinctus*, *A*. *tsurutae*.  

*Extrolites*: Asperdurin, asperpentyn, cyclopaldic acids, duricaulic acid, fumagillin, meleagrin, pseurotins, roquefortine C, viriditoxin. Asperdurin, asperpentyn, cyclopaldic acids, duricaulic acid and meleagrin has only been found in ser. *Brevipedes* in sect. *Fumigati*.  

Series ***Fennelliarum*** Houbraken & Frisvad, ***ser*. *nov*.** MycoBank MB832598.  

*Etymology*: Named after the type species of the series, *Aspergillus fennelliae*.  

*Type*: *Aspergillus fennelliae* Kwon-Chung & S.J. Kim, Mycologia 66: 629. 1974.  

*Diagnosis*: *Phylogeny*: Series *Fennelliarum* belongs to sect. *Fumigati*, subgen. *Fumigati*; Fig. 15 shows a close phylogenetic relationship with ser. *Unilaterales*, though statistical support for this relationship is lacking. *Morphology & physiology*: Colonies spreading; conidial colour *en masse* blue-green; conidiophores uniseriate; good growth at 37 °C. *Sexual morph* neosarotya-type, homo- or heterothallic, white, yellowish white or pale yellow. Series description based on Samson *et al.* (2007a) and Hubka *et al.* (2017).  

*Included species*: *Aspergillus denticulatus*, *A*. *fennelliae*, *A*. *huiyaniae*, *A*. *similanensis*, *A*. *sublevisporus*.  

*Extrolites*: Antafumicins, asperfuran, aszonalenins, aszonapyrones, chevalones, fumigaclavines, fumigatins, gliotoxin, isocoumarins, pyripyropenes, reticulol, similanamide, similanpyrones, viridicatumtoxin, viriditoxin. Asperfuran, the isocoumarins, reticulol and viridicatumtoxin are only found in this series in sect. *Fumigati*.  

Series ***Fumigati*** Houbraken & Frisvad, ***ser*. *nov*.** MycoBank MB832994.  

*Etymology*: Named after the type species of the series, *Aspergillus fumigatus*.  

*Type*: *Aspergillus fumigatus* Fresen., Beitr. Mykol. 3: 81. 1863.  

*Diagnosis*: *Phylogeny*: Series *Fumigati* belongs to sect. *Fumigati*, subgen. *Fumigati*, and is phylogenetically most closely related to ser. *Viridinutantes* (Fig. 15). *Morphology & physiology*: Colonies spreading; conidial colour *en masse* blue-green; conidiophores uniseriate; growth at 37 and 50 °C. *Sexual morph* not observed in culture or present, neosartorya-type, homo- or heterothallic, white or yellowish white. Series description based on Samson *et al.* (2007a) and Hubka *et al.* (2017).  

*Included species*: *Aspergillus fischeri*, *A*. *fumigatiaffinis*, *A*. *fumigatus*, *A*. *fumisynnematus*, *A*. *laciniosus*, *A*. *lentulus*, *A*. *novofumigatus*, *A*. *oerlinghausensis*, *A*. *spinosus*, *A*. *takakii*.  

*Extrolites*: Extrolite families found in species of ser. *Fumigati* include ardeemins, asnovolins, asperfumigatin, asperfumin (asperfumoid), aszonalenins, aszonapyrones, aurantines, avenaciolides, cephalimycins, chevalones, chloroanthraquinones, chrysogine, cottoquinazolines, cycloechinuline, cyclopiazonic acids, cyclotryprostatins, fiscalins, fischerins, fumagillins, fumicyclines, fumigachlorin, fumigaclavines, fumigatins, fumimycins, fumiquinazolines, fumitremorgins, gliotoxins, helvolic acids, lentulins, neosartoricins, novoamauromins, novobenzomalvins, novofumigatamide, palitantins, pseurotins, pyripyropenes, sartorenol, sartorypyrones, sesterfischeric acids, setosusin, sphingofungins, takakiamide, terreins, (territrems), trypacidins, tryprostatins, tryptoquivalines, wortmannins, xanthocillins. Even though ser. *Fumigati* species produce the largest diversity of different secondary metabolites, certain secondary metabolites are only found in other series of sect. *Fumigati*, for example anishidiol, antafumicins, asperdurin, asperfurans, asperpentyns, avenaciolides, cyclopaldic acids, cytochalasins, gancidin, glabramycins, kotanins, meleagrin, monochaetin, mycophenolic acid, phomaligins, reticulol, sartoryglabrins, sartorymensin, tatenoic acid, viridicatumtoxins, viriditins, viriditoxins, wasabidienones, wortmannins.  

Series ***Neoglabri*** Houbraken & Frisvad, ***ser*. *nov*.** MycoBank MB832599.  

*Etymology*: Named after the type species of the series, *Aspergillus neoglaber*.  

*Type*: *Aspergillus neoglaber* Kozak., Mycol. Pap. 161: 56. 1989.  

*Diagnosis*: *Phylogeny*: Series *Neoglabri* belongs to sect. *Fumigati*, subgen. *Fumigati*; Fig. 15 shows a phylogenetic relationship with series *Fennelliarum* and *Unilaterales*, though statistical support for this relationship is lacking. *Morphology & physiology*: Colonies spreading; conidial colour *en masse* blue-green; conidiophores uniseriate; good growth at 37 °C. *Sexual morph* not observed in culture or present, neosartorya-type, homothallic, white, yellowish white or pale yellow. Series description based on Samson *et al.* (2007a) and Hubka *et al.* (2017).  

*Included species*: *Aspergillus auratus*, *A*. *australensis*, *A*. *brevistipitatus*, *A*. *conversis*, *A*. *elsenburgensis*∗, *A*. *galapagensis*, *A*. *neoglaber*, *A*. *papuensis*, *A*. *shendaweii*, *A*. *solicola*, *A*. *stramenius* (∗ not included in Fig. 15).  

*Extrolites*: Extrolite families found in ser. *Neoglabri* include antafumicins, asperpentyns, aszonalenins, avenaciolides, chrysogines, clavatols, fellutanines, fumigatin, glabramycins, gregatins, helvolic acid, kotanins, sartoryglabramides, sartoryglabrin, takakiamide, tryptoquivalines, wortmannins. The asperpentyns, chrysogines, fellutanins, glabramycins, gregatins, sartoryglabramides, sartoryglabrin, takakiamide have only been found in ser. *Neoglabri*.  

Series ***Spathulati*** Houbraken & Frisvad, ***ser*. *nov*.** MycoBank MB832600.  

*Etymology*: Named after the type species of the series, *Aspergillus spathulatus*.  

*Type*: *Aspergillus spathulatus* Takada & Udagawa, Mycotaxon 24: 396. 1985.  

*Diagnosis*: *Phylogeny*: Series *Spathulati* belongs to sect. *Fumigati*, subgen. *Fumigati*; this section is phylogenetically unique and Fig. 15 shows a relationship with series *Fennelliarum*, *Neoglabri* and *Unilaterales*, though statistical support for this relationship is lacking. *Morphology & physiology*: Colonies spreading; conidial colour *en masse* blue-green; conidiophores uniseriate; good growth at 37 °C. *Sexual morph* neosartorya-type, homothallic, pale yellow; ascospores with large equatorial crests, convex surface smooth. Series description based on Samson *et al.* (2007a) and Hubka *et al.* (2017).  

*Included species*: *Aspergillus spathulatus*, *A*. *takadae*∗ (∗ not included in Fig. 15).  

*Extrolites*: Aszonalenins, xanthocillins.  

Series ***Thermomutati*** Houbraken & Frisvad, ***ser*. *nov*.** MycoBank MB832601.  

*Etymology*: Named after the type species of the series, *Aspergillus thermomutatus*.  

*Type*: *Aspergillus thermomutatus* (Paden) S.W. Peterson, Mycol. Res. 96: 549. 1992.  

*Diagnosis*: *Phylogeny*: Series *Thermomutati* belongs to sect. *Fumigati*, subgen. *Fumigati*; the phylogenetic relationship with other series of the section remains unresolved (Fig. 15). *Morphology & physiology*: Colonies spreading; conidial colour *en masse* blue-green; conidiophores uniseriate; good growth at 37 °C. *Sexual morph* neosartorya-type, homothallic, white, yellowish white. Series description based on Samson *et al.* (2007a) and Hubka *et al.* (2017).  

*Included species*: *Aspergillus delicatus*, *A*. *tatenoi*, *A*. *thermomutatus*.  

*Extrolites*: Extrolite families found in ser. *Thermomutati* include aszonalenins, aszonapyrones, brasilianamides, eurochevalierine, fischerindoline, gliotoxins, helvolic acids, pseudofischerine, pyripyropenes, tatenoic acid, tryptoquivalines (R & S). The brasilianamides, eurochevelierine, fischerindole, pseudofischerine and tatenoic acid has only been found in species in ser. *Thermomutati* in sect. *Fumigati*.  

Series ***Unilaterales*** Houbraken & Frisvad, ***ser*. *nov*.** MycoBank MB832602.  

*Etymology*: Named after the type species of the series, *Aspergillus unilateralis*.  

*Type*: *Aspergillus unilateralis* Thrower, Austral. J. Bot. 2: 355. 1954.  

*Diagnosis*: *Phylogeny*: Series *Unillateralis* belongs to sect. *Fumigati*, subgen. *Fumigati*; Fig. 15 shows a close phylogenetic relationship with ser. *Fennelliarum*, though statistical support for this relationship is lacking. *Morphology & physiology*: Colonies spreading; conidial colour *en masse* blue-green; conidiophores uniseriate, sometimes with nodding heads; maximum growth temperature around 42 to 45 °C, sometimes lower (*A*. *tasmanicus*) or higher (*A*. *assulatus*, *A*. *marvanovae*, *A*. *nishimurae*, *A*. *turcosus*, and *A*. *waksmanii*, 47 °C). *Sexual morph* not observed in culture, or present, neosartorya-type, homo- or heterothallic, white, yellowish white or pale yellow. Series description based on Samson *et al.* (2007a) and Hubka *et al.* (2017).  

*Included species*: *Aspergillus assulatus*, *A caatingaensis*, *A*. *hiratsukae*, *A*. *marvanovae*, *A*. *multiplicatus*, *A*. *nishimurae*, *A*. *pernambucoensis*, *A*. *tasmanicus*, *A*. *tsunodae*, *A*. *turcosus*, *A*. *unilateralis*, *A*. *waksmannii*.  

*Extrolites*: Anishidiol, aszonalenins, aszonapyrones, avenaciolides, gliotoxins, helvolic acid, kotanins, monochaetin, mycophenolic acid and sartorypyrones. Anishidiol has only been found in series *Unilaterales*, but the kotanins have also been found in ser. *Neoglabri*.  

Series ***Viridinutantes*** Houbraken & Frisvad, ***ser*. *nov*.** MycoBank MB832603.  

*Etymology*: Named after the type species of the series, *Aspergillus viridinutans*.  

*Type*: *Aspergillus viridinutans* Ducker & Thrower, Austal. J. Bot. 2: 355. 1954.  

*Diagnosis*: *Phylogeny*: Series *Viridinutantes* belongs to sect. *Fumigati*, subgen. *Fumigati* and is phylogenetically most closely related to ser. *Fumigati* (Fig. 15). *Morphology & physiology*: Colonies spreading, some species growing moderately fast (*A*. *viridinutans*, *A*. *frankstonensis*); conidial colour *en masse* blue-green; conidiophores uniseriate, generally with nodding heads; good growth at 37 °C, maximum growth temperature of 42 or 45 °C. *Sexual morph* not observed in culture, or present, neosartorya-type, homo- or heterothallic, white, yellowish white. Series description based on Talbot *et al.* (2017) and Hubka *et al.* (2018a).  

*Included species*: *Aspergillus acrensis*, *A*. *arcoverdensis*, *A*. *aureolus*, *A*. *bezerrae*, *A*. *curviformis*, *A*. *felis*, *A*. *frankstonensis*, *A*. *pseudoviridinutans*, *A*. *siamensis*, *A*. *udagawae*, *A*. *viridinutans*, *A*. *wyomingensis*. *Aspergillus curviformis* is tentatively included in this series based on its original description; no sequence data or material was available for this study.  

*Extrolites*: Extrolite families found in ser. *Viridinutantes* include ardeemins, aszonapyrones, chevalones, clavatols, cytochalasins, fiscalins, fumigaclavines, fumagillins, fumigatins, fumiquinazolins, gancidin, helvolic acid, neosartoryadines, neosartoryones, phomaligins, pseurotins, pyripyropenes, trypacidins, sartorymensin, tryptoquivalines, viriditin, viriditoxin, wasabidienones. The cytochalasins, gancidin, neosartoryadines, neosartoryones, phomaligins, sartorymensin, and viriditin have only been found in ser. *Viridinutantes* in sect. *Fumigati*.  

*Notes on series in sect*. *Fumigati*: Hubka *et al.* (2017) studied the phylogenetic relationship of sect. *Fumigati* species and recognised eight clades (*A*. *brevipes-*, *A*. *fennelliae-*, *A*. *fumigatus-*, *A*. *neoglaber-*, *A*. *spinosus-*, *A*. *tatenoi-*, *A*. *unilateralis*- and *A*. *viridinutans*-clade). More recently, Hubka *et al.* (2018a) recognised three additional clades: the *A*. *thermomutatus*-, *A*. *spathulatus*- and *A*. *auratus*-clade. Samson *et al.* (2007a) were the last who studied the taxonomy of the whole section. Based on this information, and data in more recent publications, it is difficult to find good characters to delimit series in this section. The series classification is therefore mainly based on (published) phylogenetic data (Hubka et al., 2017, Hubka et al., 2018a, this study). Extrolites in sect. *Fumigati* (with its eight families): ardeemins, asnovolins, aszonalenins, avenaciolides, cephalimycins, chaetominines, chevalones, chrysogines, clavatols, cycloechinulins, cyclopiazonic acids, cytochalasins, expansolides, fiscalins, fischerins, fumagillins,fumicyclins, fumigaclavines, fumigatins, fumigatonins, fumiquinazolines, fumitremorgins, gangicins, glabramycins, gliotoxins, helvolic acids, lentulins, neosartorins, novoamaauromins, novobenzomalvins, pseurotins, pyripyropenes, sartorypyrones, sphingofungins terreins trypacidins, tryprostatins, tryptoquivalines, viridicatumtoxins, viriditoxins, wortmannins and several more (see above) (Hong et al., 2005, Larsen et al., 2007, Samson et al., 2007a, Hong et al., 2008, Hubka et al., 2013b, Hubka et al., 2017, Hubka et al., 2018a, Frisvad and Larsen, 2015, Tamiya et al., 2015, Bessa et al., 2016, May Zin et al., 2016, Rajachan et al., 2016, Yu et al., 2016, Bang et al., 2019, Xu et al., 2019a, Yu et al., 2019). Among these, the aszonalenin biosynthetic family (BF) is shared by six species, the pyrones (aszonapyrone, chavalones, sartorypyrones) are shared by five species, the helvolic acid biosynthetic family is shared by five species, the pyripyropene BF is shared by four species, the gliotoxin BF is shared by four species, the fumigatin BF is shared by 4 species and the tryptoquivaline BF is shared by four species. Since the secondary metabolite biosynthetic family members are distributed as polythetic characters, not every member of the species series may produce these extrolites.  

**Section *Vargarum*** Houbraken & Frisvad, ***sect*. *nov*.** MycoBank MB832604.  

*Etymology*: In honour of Janos Varga, a prominent *Aspergillus* researcher and advocate of a broad monophyletic *Aspergillus* including polypaecilum-type morphs.  

*Type*: *Aspergillus cejpii* (Milko) Samson *et al.*, Stud. Mycol. 78: 155. 2014.  

*Diagnosis*: *Phylogeny*: Section *Vargarum* belongs to subgen. *Fumigati* and is phylogenetically most closely related to sect. *Clavati* (Fig. 2, Fig. 15) *Morphology & physiology*: Colonies growing moderately fast; conidiophores polypaecilum-like; growth at 37 °C. *Sexual morph* neosartorya-type, homothallic, yellowish white.  

*Extrolites*: Isolates in ser. *Vargarum* can produce gliotoxin, rubratoxins, tryptoquivalones and xanthocillins (Varga *et al.* 2007a). These extrolites are also produced by other series in subgen. *Fumigati*, except rubratoxins, which have been found only in this series in *Aspergillus*, but is also produced by *Talaromyces purpurogenus* (Yilmaz *et al.* 2012) outside *Aspergillus*.  

*Included species*: *Aspergillus cejpii*.  

*Notes*: The polypaecilum-like asexual morph present in ser. *Vargarum* is unique in subgen. *Fumigati*. This morphology type is also found in species belonging to *Aspergillus* subgen. *Polypaecilum*.  

***Aspergillus* subgen. *Nidulantes*** W. Gams *et al.*, Adv. Pen. Asp. Syst: 57. 1986 [1985]. MycoBank MB832501.  

*Type*: *Aspergillus nidulellus* Samson & W. Gams, Adv. Pen. Asp. Syst.: 44. 1986 [1985] (= *Aspergillus nidulans*).  

*Description*: See Gams *et al.* (1985) (morphology); Kocsubé *et al.* (2016), Fig. 1, Fig. 2, this study (phylogeny); Chen *et al.* (2016a) (morphology, phylogeny).  

**Section *Aenei*** Varga & Samson, IMA Fungus 1: 203. 2010. MycoBank MB517672.  

*Type*: *Aspergillus aeneus* Sappa, Allionia 2: 84. 1954.  

**Fig. 16:**
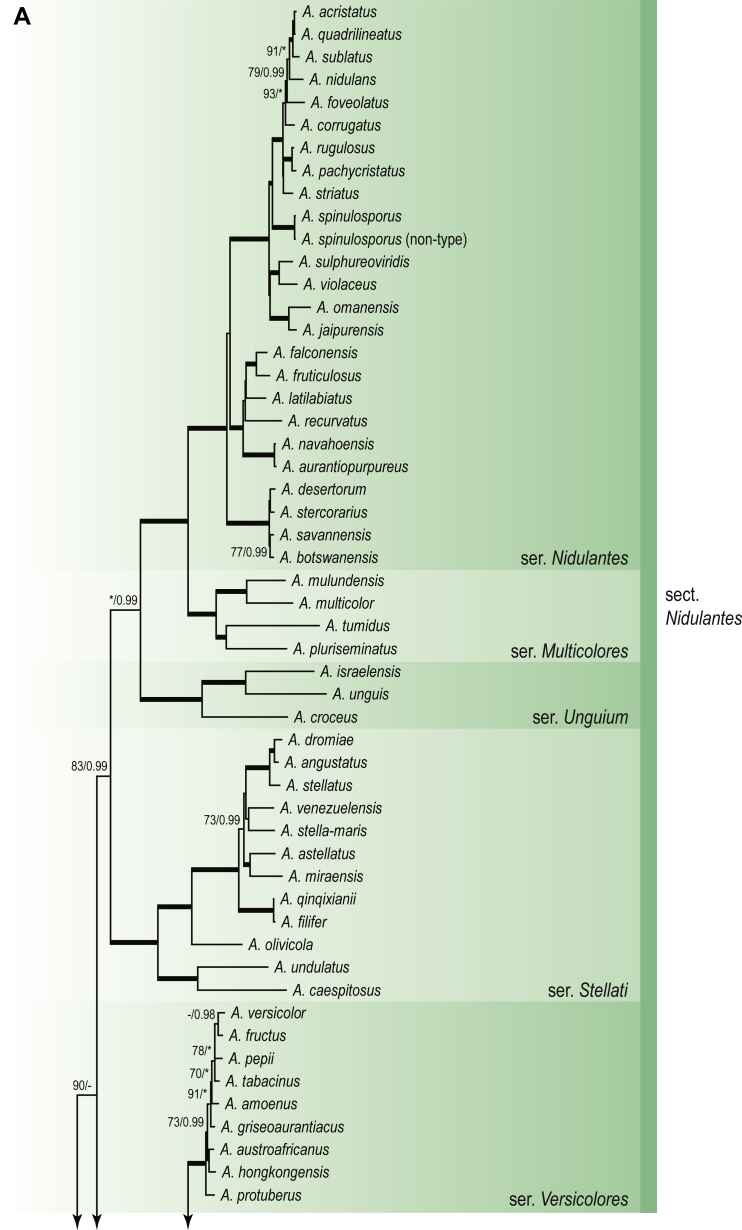

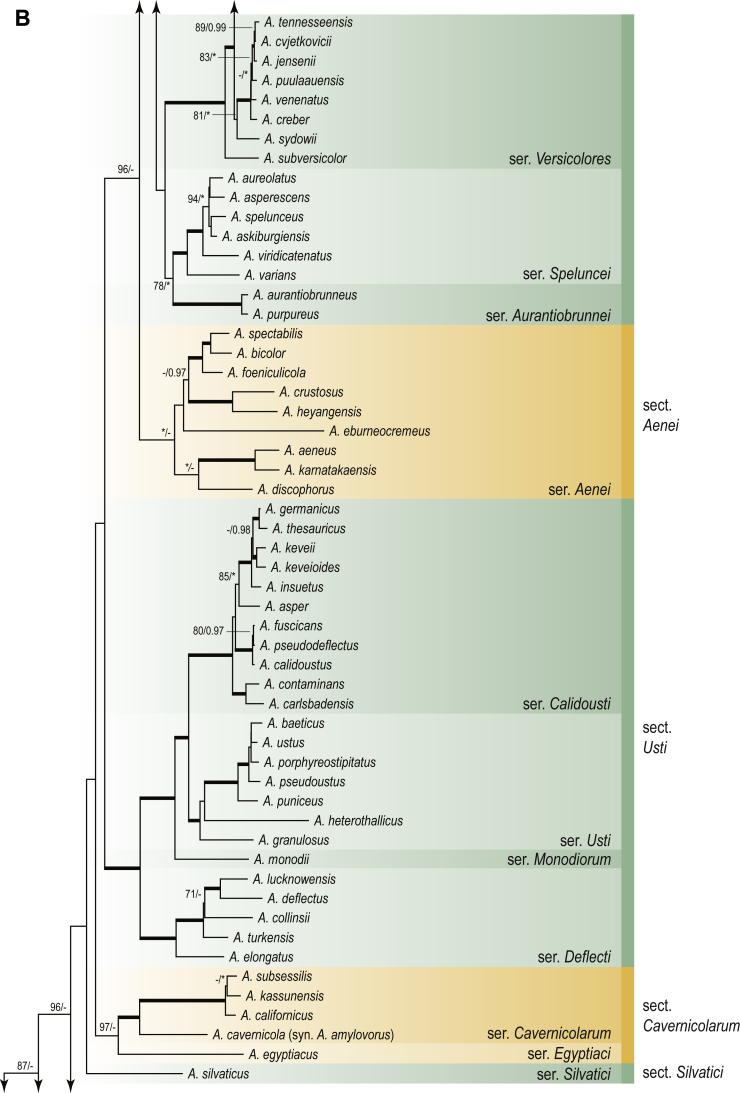

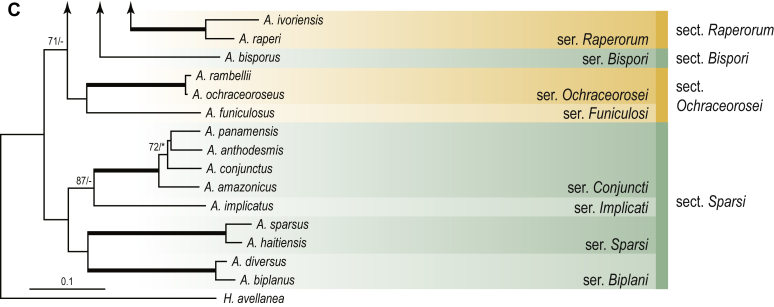
Combined phylogeny for *BenA*, *CaM* and *RPB2* data sets showing the phylogenetic relation of species, series and sections within *Aspergillus* subgen. *Nidulantes*. The BI posterior probability (pp) values and bootstrap percentages of the maximum likelihood (ML) analysis are presented at the nodes; fully supported branches are thickened. Values less than 70 % bootstrap support (ML) or less than 0.95 posterior probability (Bayesian analysis) are indicated with a hyphen or not shown. The bar indicates the number of substitutions per site. The phylogram is rooted with *Hamigera avellanea* and *Penicillium expansum*.

*Description*: *Phylogeny*: Section *Aenei* belongs to subgen. *Nidulantes* and is phylogenetically sister to sect. *Nidulantes* (Fig. 2, Fig. 16) *Morphology & physiology*: Colonies growing moderately or fast, conidia *en masse* in shades of green or olive-brown; conidiophores biseriate, stipes brown pigmented, Hülle cells abundant (except in *A*. *heyangensis*), often in crusts, globose, subglobose or pyriform; no growth at or above 40 °C. *Sexual morph* not observed in culture, or present, emericella-type, homothallic; ascospore convex smooth or delicately roughened, with two equatorial crests. Section description based on Varga *et al.* (2010a).

*Included species*: *Aspergillus aeneus*, *A*. *bicolor*, *A*. *coloradensis*∗, *A*. *crustosus*, *A*. *discophorus*, *A*. *eburneocremeus*, *A*. *foeniculicola*, *A*. *heyangensis*, *A*. *karnatakaensis*, *A*. *spectabilis* [∗ not included in Fig. 16; details on classification, see Sklenář *et al.* (2020)].  

*Extrolites*: Sterigmatocystin (5/9), decaturins (3/9), asperugins (2/9), asteltoxin (2/9), karnatakafurans (2/9), Mer-NF8054X (2/9), emeheteron (1/9), fumitremorgins (1/9), physcion (1/9), PR-toxin (1/9), quinolactacin (1/9), stellatin (1/9), terrein (1/9) (Manniche et al., 2004, Varga et al., 2010a).  

*Notes*: Hubka *et al.* (2016a) included the current sect. *Aenei* in sect. *Nidulantes* as the *Aspergillus aeneus*-clade. Our nine-gene phylogenetic analysis locates representatives of sect. *Aenei* with statistical confidence outside sect. *Nidulantes*, confirming the results of previous reports (Varga et al., 2010a, Chen et al., 2016a). Phenotypically, the sexual species in sect. *Aenei* (*A*. *bicolor*, *A*. *discophorus*, *A*. *foeniculicola*, *A*. *spectabilis*) produce similar ascospores as taxa in the *A*. *nidulans*-clade, but none of them are able to grow at 40 °C (Chen *et al.* 2016a). There are no data indicating a subdivision of sect. *Aenei* into more than one series, and ser. *Aenei* is therefore only informally introduced here (see Table 4).  

**Section *Bispori*** S.W. Peterson *et al.* ex Houbraken, ***sect*. *nov*.** MycoBank MB833244.

*Synonym*: *Aspergillus* sect. *Bispori* Peterson *et al.*, Aspergillus in the genomic era: 42. 2008; *sect*. *inval*.  

*Etymology*: Named after the type species of this section, *A*. *bisporus*.  

*Type*: *Aspergillus bisporus* Kwon-Chung & Fennell, Mycologia 63: 479. 1971.  

*Diagnosis*: *Phylogeny*: Section *Bispori* belongs to subgen. *Nidulantes* and is phylogenetically sister to a large clade containing sections *Nidulantes*, *Aenei*, *Usti*, *Cavernicolarum*, *Raperorum* and *Silvatici*. *Morphology & physiology*: Colonies restricted or growing moderately fast, conidia *en masse* olive to dark brown; conidiophores uniseriate, stipes slightly brown pigmented, smooth, occasionally showing granular pigment deposits; colonies growing more rapid at 37 °C than at 25 °C. *Sexual morph* unknown. Section description based on Kwon-Chung & Fennell (1971) (under *A*. *bisporus*).  

*Included species*: *Aspergillus bisporus*.  

*Extrolites*: There are no known extrolites from *A*. *bisporus*.  

*Notes*: *Aspergillus bisporus* could not be satisfactorily fitted in any of the groups delineated by Raper & Fennell (1965). Based on morphological similarities, Samson (1979) classified the species in sect. *Cervini*. Molecular studies revealed that *A*. *bisporus* belongs to subgen. *Nidulantes* (Peterson, 2000, Peterson, 2008, Peterson et al., 2008, Chen et al., 2016a). Section *Bispori* was informally introduced (Peterson *et al.* 2008), and this section is validated here. This section includes one species, *A*. *bisporus*, and introducing ser. *Bispori* would therefore be superfluous; ser. *Bispori* is only informally introduced here.  

**Section *Cavernicolarum*** [as “*Cavernicolus*”*’*] A.J. Chen *et al.*, Stud. Mycol. 84: 112. 2016. MycoBank MB832535.  

*Type*: *Aspergillus cavernicola* Lörinczi, Contrtii bot. Univ. Babes-Bolyai, Cluj, Grad. bot.: 341. 1969.  

*Description*: See Chen *et al.* (2016a) (morphology, phylogeny).  

Series ***Cavernicolarum*** Houbraken & Frisvad, ***ser*. *nov*.** MycoBank MB833245.  

*Etymology*: Named after the type species of this series, *A*. *cavernicola*.  

*Type*: *Aspergillus cavernicola* Lörinczi, Contrtii bot. Univ. Babes-Bolyai, Cluj, Grad. bot.: 341. 1969.  

*Diagnosis*: *Phylogeny*: Series *Cavernicolarum* belongs to sect. *Cavernicolarum*, subgen. *Nidulantes* and is phylogenetically sister to a clade containing ser. *Egyptiaci*. *Morphology & physiology*: Colonies growing restrictedly or moderately rapid, conidia *en masse* in shades of green or brown; conidiophores biseriate, short, occasionally long (in *A*. *californicus*), stipes uncoloured or in brown shades, smooth, Hülle cells often present, usually globose or subglobose; no growth at 37 °C. *Sexual morph* unknown. Series description based on Raper & Fennell (1965) and Chen *et al.* (2016a).  

*Included species*: *Aspergillus californicus*, *A*. *cavernicola*, *A*. *kassunensis*, *A*. *subsessilis*.  

*Extrolites*: Red azaphilones of the same constitution as monascorubramin and N-glutarylrubropunctamin and similar extrolites found in *Talaromyces albobiverticillius*, *Tal*. *atroroseus* and *Tal*. *purpurogenus* (Yilmaz et al., 2012, Frisvad et al., 2013b, Venkatachalam et al., 2018) have been detected in *A*. *cavernicola*, including monasnicotinic acid (Antipova *et al.* 2018a). *Aspergillus kassunensis* produces asperugins and Mer-NF8054X and *A*. *californicus* arugosins (Samson *et al.* 2011b). No extrolites are common for the four species.  

Series ***Egyptiaci*** Houbraken & Frisvad, ***ser*. *nov*.** MycoBank MB833246.  

*Etymology*: Named after the type species of this series, *A*. *egyptiacus*.  

*Type*: *Aspergillus egyptiacus* Moub. & Mustafa, Egypt. J. Bot. 15: 153. 1972.  

*Diagnosis*: *Phylogeny*: Series *Egyptiaci* belongs to sect. *Cavernicolarum*, subgen. *Nidulantes* and is phylogenetically sister to a clade containing ser. *Cavernicolarum*. *Morphology & physiology*: Colonies restricted, conidia *en masse* in shades of green; conidiophores, mostly not arranged in typical *Aspergillus* heads when grown at 25 °C, biseriate, short, smooth; often also poorly developed conidiophores present: phialides solitary or occurring in little groups along the conidiophores resembling penicillium-like structures; Hülle cells produced, varying from globose to almost cylindrical; good growth on malt extract agar with 20 % or 40 % sucrose at 35 °C, minimum growth temperature 15 °C, optimum 35 °C and maximum 45 °C. *Sexual morph* unknown. Series description based on Samson & Mouchacca (1974).  

*Included species*: *Aspergillus egyptiacus*.  

*Extrolites*: Fumitremorgin A & B, verruculogen and biosynthetically related indole-terpene-alkaloids are produced by *A*. *egyptiacus* (Samson *et al.* 2011b). These compounds are produced by species in both *Aspergillus* (different sections) and *Penicillium* (also different sections), but also by one species in sect. *Nidulantes*, ser. *Stellati*, namely *A*. *caespitosus* (Schroeder et al., 1975, Steyn et al., 1981).  

*Notes*: Section *Cavernicolarum* contains five species previously assigned to sect. *Usti* and share the production of short stipes. Series *Egyptiaci* is phylogenetically distinct and the sole species in this section that grows well at 37 °C; ser. *Cavernicolarum* species do not grow at this temperature.  

**Section *Nidulantes*** W. Gams *et al.*, Adv. Pen. Asp. Syst.: 57. 1986 [1985]. MycoBank MB832502.  

*Type*: *Aspergillus nidulellus* Samson & W. Gams, Adv. Pen. Asp. Syst.: 44. 1986 [1985]. MycoBank MB114711 (= *Aspergillus nidulans*).  

*Description*: See Gams et al., 1985, Chen et al., 2016a (morphology, phylogeny), Kocsubé *et al.* (2016) (phylogeny).  

Series ***Aurantiobrunnei*** Houbraken & Frisvad, ***ser*. *nov*.** MycoBank MB833247.  

*Etymology*: Named after the type species of this series, *A*. *aurantiobrunneus*.  

*Type*: *Aspergillus aurantiobrunneus* (G.A. Atkins *et al.*) Raper & Fennell, Gen. Aspergillus: 511. 1965.  

*Diagnosis*: *Phylogeny*: Series *Aurantiobrunnei* belongs to sect. *Nidulantes*, subgen. *Nidulantes* and is phylogenetically sister of ser. *Speluncei*. *Morphology & physiology*: Colonies restricted, sporulation absent or sparse; conidiophores biseriate, smooth, stipes hyaline to pale brown; Hülle cells present, hyaline to pale brown, globose, subglobose or ovoid; no growth at 37 °C. *Sexual morph* emericella-type, homothallic; ascospores globose to subglobose, with two equatorial crests, smooth convex. Series description based on Chen *et al.* (2016a).  

*Included species*: *Aspergillus aurantiobrunneus*, *A*. *purpureus*.  

*Extrolites*: Epurpurins (including emerin), variecolactones (including emericolins, stellatic acid, variecoacetal, variecolin, variecolol), and sterigmatocystin (including versicolorins) are shared by the two species in the ser. *Aurantiobrunnei*. Eremophiline has been found in *A*. *aurantiobrunneus* and calbistrins and shamixanthones has been found in *A*. *purpureus* (Chen *et al.* 2016a).  

Series ***Multicolores*** Houbraken & Frisvad, ***ser*. *nov*.** MycoBank MB833248.  

*Etymology*: Named after the type species of this series, *A*. *multicolor*.  

*Type*: *Aspergillus multicolor* Sappa, Allionia 2: 87. 1954.  

*Diagnosis*: *Phylogeny*: Series *Multicolores* belongs to sect. *Nidulantes*, subgen. *Nidulantes* and is phylogenetically sister of ser. *Nidulantes*. *Morphology & physiology*: Colonies growing moderately fast or spreading, conidia *en masse* in shades of green; conidiophores biseriate, smooth, stipes hyaline to yellowish brown; Hülle cells absent (*A*. *mulundensis*, *A*. *purpureocrustaceus*) or present (*A*. *incahuasiensis*, *A*. *multicolor*, *A*. *pluriseminatus*, *A*. *tumidus*), pale yellowish brown, orange, brown to pink, globose, subglobose or ovoid. *Sexual morph* not observed in culture, or present (*A*. *pluriseminatus*), emericella-type, homothallic; ascospores lenticular, convex surface tuberculate under SEM, with two conspicuously pleated, stellate and striate equatorial crests. Series description based on Stchigel and Guarro, 1997, Chen et al., 2016a, Crous et al., 2018b, Piontelli et al., 2019.  

*Included species*: *Aspergillus incahuasiensis*∗, *A*. *multicolor*, *A*. *mulundensis*, *A*. *pluriseminatus*, *A*. *purpureocrustaceus*∗, *A*. *tumidus* [∗ not included in Fig. 16; details on classification, see Sklenář *et al.* (2020)].  

*Extrolites*: *Aspergillus multicolor*, *A*. *mulundensis* and *A*. *pluriseminatus* all produce asticolorins or closely related dibenzofurans. *Aspergillus mulundensis* produces mulundocandins and emericellamide, and azaphilones in common with *A*. *pluriseminatus* (Bills et al., 2016, Chen et al., 2016a), while *A*. *multicolor* is the only species in the series that has been reported to produce sterigmatocystins (Hamasaki *et al.* 1980).  

Series ***Nidulantes*** Houbraken & Frisvad, ***ser*. *nov*.** MycoBank MB833249.  

*Etymology*: Named after the type species of this series, *A*. *nidulellus*.  

*Type*: *Aspergillus nidulellus* Samson & W. Gams, Adv. Pen. Asp. Syst.: 44. 1986 [1985]. MycoBank MB114711 (= *Aspergillus nidulans*).  

*Diagnosis*: *Phylogeny*: Series *Nidulantes* belongs to sect. *Nidulantes*, subgen. *Nidulantes* and is phylogenetically sister of ser. *Multicolores*. *Morphology & physiology*: Colonies spreading, conidia *en masse* green; conidiophores biseriate, stipes hyaline to yellowish brown pigmented, smooth, occasionally with surface protuberances; Hülle cells present, globose; good growth at 37 °C and 40 °C, growth at 45 °C, except for *A*. *botswanensis*, *A*. *fruticulosus*, *A*. *latilabiatus* and *A*. *recurvatus*. *Sexual morph* generally present (only not observed in *A*. *recurvatus*), emericella-type, homothallic; ascospores irregularly wrinkled, finely pitted, rugulose or echinulate, with two equatorial crests (except for four crests in *A*. *quadrilineatus*). Series description based on Chen *et al.* (2016a).  

*Included species*: *Aspergillus amethystinus∗*, *A*. *aurantiopurpureus*, *A*. *botswanensis*, *A*. *corrugatus*, *A*. *desertorum*, *A*. *dipodomyus*, *A*. *falconensis*, *A*. *foveolatus*, *A*. *fruticulosus*, *A*. *jaipurensis*, *A*. *latilabiatus*, *A*. *navahoensis*, *A*. *nidulans*, *A*. *omanensis*, *A*. *pachycristatus*, *A*. *quadrilineatus*, *A*. *recurvatus*, *A*. *rugulosus*, *A*. *savannensis*, *A*. *spinulosporus*, *A*. *stercorarius*, *A*. *striatus*, *A*. *sublatus*, *A*. *sulphureoviridis*, *A*. *violaceus* [∗ not included in Fig. 16; details on classification, see Sklen]? et al. (2020)].  

*Extrolites* (number of species producing compound / total species in series): Asperthecin (14/22), asperugins (10/22), austinols (2/22), austalides (5/22), calbistrins (2/22), cordycepin (1/22), cyclopaldic acid (1/22), desertorins (6/22), echinocandins (3/22), emericellin (9/22), emerin/epurpurins (2/22), emestrin (6/22), emindols (7/22), falconensins (6/22), falconensons (5/22), gregartins (2/22), 2-ω-hydroxyemodin (4/22), isocoumarins (1/22), paxillin (6/22), quadrilineatin (and the related nidulol & silvaticol) (2/22), shamixanthones (10/22), sterigmatocystin (14/22, *A*. *latilabiatus* producing only versicolorins), terrequinone A (4/22), violaceols (16/22), viridicatumtoxin (1/22) (Chen *et al.* 2016a).  

Series ***Speluncei*** Houbraken & Frisvad, ***ser*. *nov*.** MycoBank MB833250.  

*Etymology*: Named after the type species of this series, *A*. *spelunceus*.  

*Type*: *Aspergillus spelunceus* Raper & Fennell [as “*speluneus*”], Gen. Aspergillus: 457. 1965.  

*Diagnosis*: *Phylogeny*: Series *Speluncei* belongs to sect. *Nidulantes*, subgen. *Nidulantes* and is phylogenetically sister of ser. *Aurantiobrunnei*. *Morphology & physiology*: Colonies restricted or moderately fast, sporulation sparse or moderate, *en masse* in shades of green; conidiophores biseriate, stipes hyaline or yellowish brown, smooth; Hülle cells absent or present (*A*. *askiburgiensis*, *A*. *asperescens*, *A*. *spelunceus*), hyaline, globose, subglobose or ovoid; no growth at 37 °C (except *A*. *asperescens*). *Sexual morph* unknown. Series description based on Chen *et al.* (2016a).  

*Included species*: *Aspergillus askiburgiensis*, *A*. *asperescens*, *A*. *aureolatus*, *A*. *spelunceus*, *A*. *varians*, *A*. *viridicatenatus*.  

*Extrolites* (number of species producing compound / total species in series): Azaphilones (1/6), austinols (1/6), calbistrins (1/6), desertorins (1/6), emerin / epurpurins (2/6), 2-ω-hydroxyemodin (1/6), a phthalide (1/6), shamixanthones (1/6), sterigmatocystin (5/6, *A*. *varians* only produces the versicolorin precursors), violaceols (1/6), viridicatins and cyclopenols (1/6) (Chen *et al.* 2016a).  

Series ***Stellati*** Houbraken & Frisvad, ***ser*. *nov*.** MycoBank MB833251.  

*Etymology*: Named after the type species of this series, *A*. *stellatus*.  

*Type*: *Aspergillus stellatus* Curzi, Atti Reale Accad. Naz. Lincei, Rendiconti Cl. Sci. Fis. 19: 428. 1934.  

*Diagnosis*: *Phylogeny*: Series *Stellati* belongs to sect. *Nidulantes*, subgen. *Nidulantes* and is phylogenetically sister of a clade including series *Multicolores*, *Nidulantes* and *Unguium*. *Morphology & physiology*: Colonies spreading, conidia *en masse* green, conidiophores biseriate, stipes hyaline to yellowish brown, smooth; Hülle cells present, globose; no growth at 40 °C. *Sexual morph* present (except in *A*. *caespitosus*), emericella-type, homothallic; ascospores globose, stellate or appendaged. Series description based on Chen *et al.* (2016a).  

*Included species*: *Aspergillus angustatus*, *A*. *astellatus*, *A*. *caespitosus*, *A*. *dromiae*, *A*. *filifer*, *A*. *miraensis*, *A*. *olivicola*, *A*. *qinqixianii*, *A*. *stella-maris*, *A*. *stellatus*, *A*. *stelliformis*∗, *A*. *undulatus*, *A*. *venezuelensis* [∗ not included in Fig. 16; details on classification, see Sklenář *et al.* (2020)].  

*Extrolites* (number of species producing compound / total species in series): Aflatoxin B_1_ (4/12), asperlicine (1/12), asperthecin (6/12), asperugines (3/12), astellolide (1/12), asteltoxin (4/12), austinol (1/12), curvularins (1/12), desertorins (6/12), emericellins (8/12), emerin / epurpurin (1/12), fischerin (1/12), gregatins (1/12), 2-ω-hydroxyemodin (8/12), Mer-NF-8054X (1/12), secalonic acid D 781/12), shamixanthones (10/12), sterigmatocystin (5/12), terrein (3/12), varitriol (4/12), verruculogens and fumitremorgins (1/12), violaceols (1/12) (Chen *et al.* 2016a).  

Series ***Unguium*** Houbraken & Frisvad, ***ser*. *nov*.** MycoBank MB833253.  

*Etymology*: Named after the type species of this series, *A*. *unguis*.  

*Type*: *Aspergillus unguis* (Émile-Weill & L. Gaudin) Thom & Raper, Mycologia 31: 667. 1939.  

*Diagnosis*: *Phylogeny*: Series *Unguium* belongs to sect. *Nidulantes*, subgen. *Nidulantes* and is phylogenetically sister of series *Multicolores* and *Nidulantes*. *Morphology & physiology*: Colonies growing restrictedly or moderately rapid; conidia *en masse* in shades of green (yellow-green, olive-green); conidiophores biseriate; stipes hyaline to yellowish brown, smooth; Hülle cells absent (*A*. *israelensis*, *A*. *unguis*) or present (*A*. *croceus*), globose, subglobose or pyriform; growth at 37 °C absent (*A*. *croceus*, *A*. *israelensis*) or restricted (*A*. *unguis*), some *A*. *unguis* strains grow restrictedly at 40 °C). *Sexual morph* unknown, *A*. *unguis* NRRL 2393 was reported to tardily produce ascospores. Series description based on Fennell and Raper, 1955, Chen et al., 2016a, Hubka et al., 2016a.  

*Included species*: *Aspergillus croceiaffinis*∗, *A*. *croceus*, *A*. *israelensis*, *A*. *longistipitatus*∗, *A*. *unguis* [∗ not included in Fig. 16; details on classification, see Sklenář *et al.* (2020)].  

*Extrolites*: *Aspergillus unguis* produces asperunguisones (and 3-ethyl-5,7-dihydroxy-3,6-dimethyl phthalide), penicillin G, unguinols (= nidulins), aspergillusidones, aspergicides, agonodepsides, emeguisins, folipastatins, haiderin, nasrin, rubidin, shirin, yasimin (= unguinol = tridechloronidulin), unguisins, unguispyrones, ustilaginoidin C and violaceols / orcinols (Chen et al., 2016a, Phainuphong et al., 2017a, Morshed et al., 2018, Phainuphong et al., 2018a), while *A*. *croceus* produces desertorins / kotanins and sterigmatocystin and *A*. *israelensis* produce emindols (Chen *et al.* 2016a), showing there is no extrolites in common between the three species in series *Unguium*.  

Series ***Versicolores*** Houbraken & Frisvad, ***ser*. *nov*.** MycoBank MB833254.  

*Etymology*: Named after the type species of this series, *A*. *versicolor*.  

*Type*: *Aspergillus versicolor* (Vuill.) Tirab., Ann. Bot. (Roma) 7: 9. 1908.  

*Diagnosis*: *Phylogeny*: Series *Versicolores* belongs to sect. *Nidulantes*, subgen. *Nidulantes*; the phylogenetic relationship with other series in the section is unresolved. *Morphology & physiology*: Colonies restricted or moderately fast, conidia *en masse* in shades or green or brown; conidiophores biseriate, stipes hyaline or pale brown, smooth or with tubercles; Hülle cells absent or present, hyaline, globose, subglobose, ellipsoidal or pyriform; no or poor growth at 37 °C. *Sexual morph* unknown. Series description based on Jurjević *et al.* (2012) and Chen *et al.* (2016a).  

*Included species*: *Aspergillus amoenus*, *A*. *austroafricanus*, *A*. *creber*, *A*. *cvjetkovicii*, *A*. *fructus*, *A*. *griseoaurantiacus*, *A*. *hongkongensis*, *A*. *jensenii*, *A*. *pepii*, *A*. *protuberus*, *A*. *puulaauensis*, *A*. *subversicolor*, *A*. *sydowii*, *A*. *tabacinus*, *A*. *tennesseensis*, *A*. *venenatus*, *A*. *versicolor*.  

*Extrolites*: Most species produce sterigmatocystin (14/16) and precursors such as versicolorins, averufin, averufanin, and norsolorinic acid ((Jurjević et al., 2013, Chen et al., 2016a, Jakšić Despot et al., 2017). Other extrolites from series *Versicolores* include aniduquinolones (2/14), brevianamides F, J, K, Q, R, T, U (1/14), calbistrins (3/14), deoxybrevianamides (2/14), isocoumarins (2/14), cyclopenols / viridicatols (2/14), emericellin / arugosins (1/14), shamixanthones (4/14), insulicolides (2/14), mangrovamides (1/14), psychrophilin E-H (1/14), sydowic acids (5/14), sydowinins (1/14), versicolamides (and notoamides, stephacidins) (7/14), versiols (2/14), violaceols (13/14), WIN64745 (1/14) (Chen *et al.* 2016a).  

*Notes on sect*. *Nidulantes and included series*: Section *Nidulantes* species share the production of more or less brown-pigmented, smooth conidiophores (occasionally with surface protuberances) with globose, subglobose or subclavate vesicles. The conidia are generally globose and echinulate and are *en masse* green coloured. Chen *et al.* (2016a) recognised seven clades in sect. *Nidulantes* and these were named the *A*. *aurantiobrunneus*-, *A*. *multicolor*-, *A*. *nidulans*-, *A*. *spelunceus*-, *A*. *stellatus*-, *A*. *unguis*- and *A*. *versicolor*-clade. These seven clades are here treated as series. These series were primarily based on multigene phylogenetic analysis and certain shared characters, such as growth rates at 25, 37 and 40 °C and ascospore ornamentation (if produced).  

**Section *Ochraceorosei*** Frisvad & Samson, Syst. Appl. Microbiol. 28: 451. 2005. MycoBank MB500165.  

*Type*: *Aspergillus ochraceoroseus* Bartoli & Maggi, Trans. Brit. Mycol. Soc. 71: 393. 1978.  

*Description*: See Frisvad *et al.* (2005) (morphology), Kocsubé *et al.* (2016) (phylogeny).  

Series ***Funiculosi*** Houbraken & Frisvad, ***ser*. *nov*.** MycoBank MB833255.  

*Etymology*: Named after the type species of this series, *A*. *funiculosus*.  

*Type*: *Aspergillus funiculosus* G. Sm., Trans. Brit. Mycol. Soc. 39: 111. 1956.  

*Diagnosis*: *Phylogeny*: Series *Funiculosi* belongs to sect. *Ochraceorosei*, subgen. *Nidulantes* and is phylogenetically sister ser. *Ochraceorosei* (Fig. 16). *Morphology & physiology*: Colonies growing moderately; conidia *en masse* yellow-green, olive-brown to deep brownish purple; conidiophores uniseriate, stipes hyaline or faintly coloured just below the vesicle, smooth; Hülle cells absent. *Sexual morph* unknown. Series description based on Smith (1956) and Raper & Fennell (1965).  

*Included species*: *Aspergillus funiculosus*.  

*Extrolites*: *Aspergillus funiculosus* produces kojic acid (Siddhardha *et al.* 2010) and funicin = ethericin B (König et al., 1978, Hamasaki et al., 1980, König et al., 1980, Nakamura et al., 1984) and is chemically different from *A*. *ochraceoroseus* and *A*. *rambellii* in series *Ochraceorosei*. Chemically these diphenylether antibiotic extrolites are shared with *Aspergillus sydowii* and other species in series *Versicolores* (Li *et al.* 2015a) and kojic acid is shared with some species in section *Nidulantes* (Frisvad and Samson, 2004a, Frisvad et al., 2005, Chen et al., 2016a) and *Flavi* (Frisvad *et al.* 2019).  

Series ***Ochraceorosei*** Houbraken & Frisvad, ***ser*. *nov*.** MycoBank MB833256.  

*Etymology*: Named after the type species of this series, *A*. *ochraceoroseus*.  

*Type*: *Aspergillus ochraceoroseus* Bartoli & Maggi, Trans. Brit. Mycol. Soc. 71: 393. 1979 [1978].  

*Diagnosis*: *Phylogeny*: Series *Ochraceorosei* belongs to sect. *Ochraceorosei*, subgen. *Nidulantes* and is phylogenetically sister ser. *Funiculosi*. *Morphology & physiology*: Colonies growing moderately or fast on MEA, conidia *en masse* in shades of yellow; conidiophores biseriate, stipes hyaline, smooth, long; Hülle cells absent; no growth at 37 °C. *Sexual morph* unknown. Series description based on Bartoli & Maggi (1978) and Frisvad *et al.* (2005).  

*Included species*: *Aspergillus ochraceoroseus*, *A*. *rambellii*.  

*Extrolites*: Aflatoxin B_1_, B_2_, sterigmatocystin, 3-O-methylsterigmatocystin (Frisvad *et al.* 2005). Many other species produce aflatoxin and the related sterigmatocystins, but species in ser. *Ochraceorosei* do not produce kojic acid like species in sect. *Flavi* (Frisvad *et al.* 2019), but produce unique secondary metabolites, not as yet structure elucidated, that only occur in this section. No extrolites in common with the other species in sect. *Nidulantes* (Chen *et al.* 2016a).  

*Notes on series in sect*. *Ochraceorosei*: The taxonomic position of *A*. *funiculosus* was discussed several times in the past (Smith, 1956, Raper and Fennell, 1965, Peterson, 2008, Peterson et al., 2008, Hubka et al., 2016a). Smith (1956) could not satisfactorily classify this species and mentioned a possible relationship with *A*. *versicolor*, *A*. *flavus* and *A*. *glaucus*, and Raper & Fennell (1965) accepted this species in sect. *Sparsi* (as *A*. *sparsus* group). Using sequence data, *A*. *funiculosus* took a basal position in sect. *Sparsi* in the phylogenetic analysis of Peterson *et al.* (2008), while this species was more similar to *A*. *ochraceoroseus* in other analyses (Houbraken and Samson, 2011, Chen et al., 2016a). Based on our nine-gene phylogeny, *A*. *funiculosus* is sister to *Aspergillus ochraceoroseus*, *A*. *rambellii* of ser. *Ochraceorosei* (Fig. 2). The sole species in ser. *Funiculosi* is uniseriate, in contrast to the biseriate species in ser. *Ochraceorosei*. *Aspergillus funiculosus* does not produce aflatoxins or sterigmatocystins, but kojic acid and funicin (= ethericin B) (König et al., 1978, Hamasaki et al., 1980, König et al., 1980, Nakamura et al., 1984, Siddhardha et al., 2010).  

**Section *Raperorum*** S.W. Peterson, Varga, Frisvad, Samson ex Houbraken, ***sect*. *nov*.** MycoBank MB833258.

*Synonym*: *Aspergillus* sect. *Raperi* Peterson *et al.*, Aspergillus in the genomic era: 42. 2008; *sect*. *inval*.  

*Etymology*: Named after the type species of this section, *A*. *raperi*.  

*Type*: *Aspergillus raperi* Stolk & J.A. Meyer, Trans. Brit. Mycol. Soc. 40: 190. 1957.  

*Diagnosis*: *Phylogeny*: Section *Raperi* belongs to subgen. *Nidulantes* and is phylogenetically sister to a clade containing sections *Nidulantes*, *Aenei*, *Usti* and *Cavernicolarum* (Fig. 16). *Morphology & physiology*: Colonies growing restrictedly, moderately or fast, conidia *en masse* in shades of yellow or green; conidiophores uniseriate (*A*. *raperi*) or biseriate (*A*. *ivorensis*), stipes hyaline, smooth (*A*. *raperi*) or rough (*A*. *ivorensis*), long; Hülle cells present, globose to subglobose, pyriform or elongate. *Sexual morph* unknown. Section description based on Bartoli & Maggi (1978) and Stolk & Meyer (1957).  

*Included species*: *Aspergillus ivoriensis*, *A*. *raperi*.  

*Extrolites*: No known extrolites have been reported.  

*Notes*: Section *Raperorum* is introduced to accommodate *A*. *ivoriensis* and *A*. *raperi*. These species differ in growth rates and conidiophore structure. However, we tentatively keep both species in one series. Based on phenotypic characters, *Aspergillus raperi* was first placed in the *A*. *versicolor* group (= ser. *Versicolores*) (Stolk & Meyer 1957) and later in the *A*. *ornatus* group (Raper & Fennell 1965). This species forms, together with *A*. *ivoriensis*, a unique lineage in subgen. *Nidulantes*, and sect. *Raperi* was informally introduced to accommodate these species (Peterson *et al.* 2008). This section is formally introduced here as sect. *Raperorum*. Section *Raperorum* is not subdivided in series and therefore ser. *Raperorum* is only informally introduced here.  

**Section *Silvatici*** S.W. Peterson, Varga, Frisvad, Samson ex Houbraken, ***sect*. *nov*.** MycoBank MB833259.

*Synonym*: *Aspergillus* sect. *Silvati* Peterson *et al.*, Aspergillus in the genomic era: 44. 2008; *sect*. *inval*.  

*Etymology*: Named after the type species of this section, *A*. *silvaticus*.  

*Type*: *Aspergillus silvaticus* Fennell & Raper, Mycologia 47: 83. 1955.  

*Diagnosis*: *Phylogeny*: Section *Silvatici* belongs to subgen. *Nidulantes* and is phylogenetically sister to a large clade containing sections *Nidulantes*, *Aenei*, *Usti*, *Cavernicolarum* and *Raperorum* (Fig. 16). *Morphology & physiology*: Colonies growing moderate to fast, conidia *en masse* in shades of green; conidiophores biseriate, stipes brownish pigmented, smooth, seldom exceeding 300 μm; Hülle cells present, globose to subglobose. *Sexual morph* unknown. Section description based on Raper & Fennell (1965).  

*Included species*: *Aspergillus silvaticus*.  

*Extrolites*: Naphthalic anhydride, phthalides (silvaticol, O-methylsilvaticol, ethyl 3-methylorsellinate, 6-hydroxy-4-methoxy-5-methylphtalimidine, 3,6-dimethyl-4-hydroxy-2-methoxybenzaldehyde, nidulol, quadrilineatin), shamixanthones (arugosin A, B & E, silvaticamide), silvathione and dithiosilvatin (Homma et al., 1980, Yamazaki et al., 1981a, Fujita et al., 1985, Maebayashi and Yamazaki, 1985, Kawahara et al., 1986, Kawahara et al., 1987, Kawahara et al., 1988). Naphthalic anhydride and the silvatins are unique to this section.  

*Notes*: Section *Silvatici* (as *Silvati*) was informally introduced to accommodate *A*. *silvaticus* (Peterson *et al.* 2008) and this section is formally introduced here. Only one species is accommodated in sect. *Silvatici*. Introducing ser. *Silvatici* would therefore be superfluous, and the series name *Silvatici* is therefore only informally introduced.  

**Section *Sparsi*** W. Gams, M. Chr., Onions, Pitt & Samson, Adv. Pen. Asp. Syst.: 61. 1986 [1985]. MycoBank MB832514.  

*Type*: *Aspergillus sparsus* Raper & Thom, Mycologia 36: 572. 1944.  

*Description*: See Gams *et al.* (1985) (morphology), Kocsubé *et al.* (2016) (phylogeny).  

Series ***Biplani*** Houbraken & Frisvad, ***ser*. *nov*.** MycoBank MB833260.  

*Etymology*: Named after the type species of this series, *A*. *biplanus*.  

*Type*: *Aspergillus biplanus* Raper & Fennell, Gen. Aspergillus: 434. 1965.  

*Diagnosis*: *Phylogeny*: Series *Biplani* belongs to sect. *Sparsi*, subgen. *Nidulantes* and is phylogenetically sister to ser. *Sparsi* (Fig. 16). *Morphology & physiology*: Colonies growing restrictedly or moderately rapid, conidia *en masse* in shades of green (dark green, blueish green); conidiophores biseriate, stipes brown, smooth, long; fragmentary conidial structures of varying size and without definite pattern borne near the agar surface; Hülle cells absent. *Sexual morph* unknown. Series description based on Raper & Fennell (1965).  

*Included species*: *Aspergillus biplanus*, *A*. *diversus*.  

*Extrolites*: Auroglaucin is shared with sections *Aspergillus* and *Restricti*, also with *A*. *conjuctus* in ser. *Conjuncti* (Varga *et al.* 2010b).  

Series ***Conjuncti*** Houbraken & Frisvad, ***ser*. *nov*.** MycoBank MB833261.  

*Etymology*: Named after the type species of this series, *A*. *conjunctus*.  

*Type*: *Aspergillus conjunctus* Kwon-Chung & Fennell, Gen. Aspergillus: 552. 1965.  

*Diagnosis*: *Phylogeny*: Series *Conjuncti* belongs to sect. *Sparsi*, subgen. *Nidulantes* and is phylogenetically sister to ser. *Implicati* (Fig. 16). *Morphology & physiology*: Colonies grow restrictedly or moderately rapid; conidia *en masse* in shades of brown (brown, red-brown, olive brown); conidiophores biseriate, stipes hyaline or (pale) brown pigmented, smooth; reduced conidial structures produced near colony surface absent; Hülle cells absent (*A*. *amazonicus*, *A*. *anthodesmis*) or present (*A*. *conjunctus*, *A*. *panamensis*), elongate and/or variously curved or twisted. *Sexual morph* unknown. Series description based on Raper and Fennell, 1965, Bartoli and Maggi, 1978.  

*Included species*: *Aspergillus amazonicus*, *A*. *anthodesmis*, *A*. *conjunctus*, *A*. *panamensis*.  

*Extrolites* (number of species producing compound / total number of species in series): An aszonalenin (1/4), auroglaucin (1/4), gregatins (2/4), and siderin (3/4) have been found in species in ser. *Conjuncti*.  

Series ***Implicati*** Houbraken & Frisvad, ***ser*. *nov*.** MycoBank MB833262.  

*Etymology*: Named after the type species of this series, *A*. *implicatus*.  

*Type*: *Aspergillus implicatus* Persiani & Maggi, Mycol. Res. 98: 871. 1994.  

*Diagnosis*: *Phylogeny*: Series *Implicati* belongs to sect. *Sparsi*, subgen. *Nidulantes* and is phylogenetically sister to ser. *Conjuncti* (Fig. 16). *Morphology & physiology*: Colonies growing moderately, conidia *en masse* white or pale yellow; conidiophores biseriate, stipes hyaline, smooth, long, surrounded by parallel sterile hyphae, slightly echinulate, originating from the base growing up to the vesicle and later then branching to build the hyphal tangle; reduced, penicillate conidiophores present; Hülle cells absent. *Sexual morph* unknown. Series description based on Maggi & Persiani (1994).  

*Included species*: *Aspergillus implicatus*.  

*Extrolites*: Only a versicolorin has been detected in *A*. *implicatus* (Varga *et al.* 2010b), indicating that this species may produce either sterigmatocystin and aflatoxins or austocystins, as versicolorins are precursors for both biosynthetic families; however, this has not been confirmed. Since species in sect. *Sparsi* are not known for producing aflatoxins or austocystins, and since *A*. *implicatus* was found in the same habitat as *A*. *ochraceoroseus*, that actually produce aflatoxins.  

Series ***Sparsi*** Houbraken & Frisvad, ***ser*. *nov*.** MycoBank MB833263.  

*Etymology*: Named after the type species of this series, *A*. *sparsus*.  

*Type*: *Aspergillus sparsus* Raper & Thom, Mycologia 36: 572. 1944.  

*Diagnosis*: *Phylogeny*: Series *Sparsi* belongs to sect. *Sparsi*, subgenus *Nidulantes* and is phylogenetically sister to ser. *Biplani* (Fig. 16). *Morphology & physiology*: Colonies spreading, conidia *en masse* reddish brown; conidiophores biseriate, stipes dark brown pigmented, smooth (*A*. *haitiensis*) or rough-walled (*A*. *sparsus*), long; small, malformed, proliferating conidial structures produced near colony surface; Hülle cells absent. *Sexual morph* unknown. Series description based on Raper & Fennell (1965) and Varga *et al.* (2010b).  

*Included species*: *Aspergillus haitiensis*, *A*. *sparsus*.  

*Extrolites*: Some extrolites of unknown structure are common for the two species in ser. *Sparsi*. The only known extrolites detected in this series are gregatins and siderin (found in *A*. *haitiensis* NRRL 4569), also found in species in ser. *Conjuncti* (Varga *et al.* 2010b).  

*Notes on sect*. *Sparsi and included series*: Fennell & Raper (1955) created the *Aspergillus sparsus* group to accommodate four species that possess certain characters, such as pigmented conidiophore stipes and production of smaller conidium forming structures (besides typical aspergillus-like conidiophores). Gams *et al.* (1985) formally introduced sect. *Sparsi* for this group of species. Peterson *et al.* (2008) included seven species in this section (*A*. *anthodesmis*, *A*. *biplanus*, *A*. *conjunctus*, *A*. *diversus*, *A*. *funiculosus*, *A*. *panamensis*, *A*. *sparsus*). Later, the taxonomy of this section was studied using a polyphasic approach and the section was expanded with *A*. *amazonicus*, *A*. *haitiensis*, *A*. *implicatus* (Varga *et al.* 2010b). All species except *A*. *funiculosus* (sect. *Ochraceorosei*, ser. *Funiculosi*) are accepted in this section in the current study (Fig. 16). The species in ser. *Implicati* produce white or pale-yellow conidia; a unique feature in sect. *Sparsi*. Hülle cells are present in some species of ser. *Conjuncti* and those are predominantly elongate. These resemble the Hülle cells produced by sect. *Usti* members, rather than the (sub)globose or pyriform Hülle cells in sections *Aenei*, *Cavernicolarum*, *Nidulantes*, *Raperorum* and *Silvatici*. Species in series *Biplani*, *Implicati* and *Sparsi* produce reduced, sometimes penicillate, conidiophores.  

**Section *Usti*** W. Gams *et al.*, Adv. Pen. Asp. Syst.: 58. 1986 [1985]. MycoBank MB832504.  

*Type*: *Aspergillus ustus* (Bainier) Thom & Church, Aspergilli: 152. 1926.  

*Description*: See Gams *et al.* (1985) (morphology), Houbraken et al., 2007, Samson et al., 2011b (morphology, phylogeny), Kocsubé *et al.* (2016) (phylogeny).  

Series ***Calidousti*** Houbraken & Frisvad, ***ser*. *nov*.** MycoBank MB833264.  

*Etymology*: Named after the type species of this series, *A*. *calidoustus*.  

*Type*: *Aspergillus calidoustus* Varga, Houbraken & Samson, Eukaryot. Cell 7: 636. 2008.  

*Diagnosis*: *Phylogeny*: Series *Calidousti* belongs to sect. *Usti*, subgen. *Nidulantes* and is phylogenetically sister of ser. *Usti* (Fig. 16). *Morphology & physiology*: Colonies growing moderately, conidia *en masse* greyish yellow, brownish grey, greyish brown or greyish green; conidiophores biseriate, stipes hyaline, (yellow-)brown, smooth; Hülle cells absent (*A*. *germanicus*, *A*. *keveioides*, *A*. *pseudodeflectus*) or present (*A*. *asper*, *A*. *calidoustus*, *A*. *carlsbadensis*, *A*. *contaminans*, *A*. *fuscicans*, *A*. *keveii*, *A*. *thesauricus*), generally irregularly elongate, ovoid, curved to coiled, sometimes globose (*A*. *carlsbadensis*). *Sexual morph* unknown. Series description based on Raper and Fennell, 1965, Samson et al., 2011b, Nováková et al., 2012, Visagie et al., 2014a, Jurjević and Peterson, 2016.  

*Included species*: *Aspergillus asper*, *A*. *calidoustus*, *A*. *carlsbadensis*, *A*. *contaminans*, *A*. *fuscicans*, *A*. *germanicus*, *A*. *insuetus*∗, *A*. *keveii*, *A*. *keveioides*, *A*. *pseudodeflectus*, *A*. *sigurros*, *A*. *thesauricus* (∗ not included in Fig. 16).  

*Extrolites*: Of the five species examined for extrolites, four produce drimans, two produce asperugins, two produce ophiobolins (G & H), one produces brevianamide A, one produces an arugosin, one produces nidulol and one produces TMC120-B (Samson et al., 2011b, Kozlovsky et al., 2017).  

Series ***Deflecti*** Houbraken & Frisvad, ***ser*. *nov*.** MycoBank MB833266.  

*Etymology*: Named after the type species of this series, *A*. *deflectus*.  

*Type*: *Aspergillus deflectus* Fennell & Raper, Mycologia 47: 83. 1955.  

*Diagnosis*: *Phylogeny*: Series *Deflecti* belongs to sect. *Usti*, subgen. *Nidulantes* and is phylogenetically sister to a clade containing series *Caldidousti*, *Monodiorum* and *Usti* (Fig. 16). *Morphology & physiology*: Colonies grow restrictedly, conidia *en masse* orange-yellow to grey-green, mycelium on CYA pinkish or orange-shaded; conidiophores biseriate, stipes hyaline or in shades of (reddish) brown or yellowish orange, smooth; Hülle cells absent (*A*. *collinsii*, *A*. *lucknowensis*) or present (*A*. *deflectus*, *A*. *elongatus*, *A*. *turkensis*), predominantly (irregularly) elongate, straight or curved. *Sexual morph* unknown. Series description based on Raper and Fennell, 1965, Samson et al., 2011b, Jurjević and Peterson, 2016.  

*Included species*: *Aspergillus collinsii*, *A*. *deflectus*, *A*. *elongatus*, *A*. *lucknowensis*, *A*. *turkensis*.  

*Extrolites*: Of the four species examined for extrolites in this series, two produce emerin, two produce deflectins, two produce shamixanthones, one produces fumitremorgin C, one produces notoamide E and one produces desacetylferriacetylfusigen B (Anke, 1977, Anke et al., 1981, Samson et al., 2011b, Kozlovsky et al., 2017).  

Series ***Monodiorum*** Houbraken & Frisvad, ***ser*. *nov*.** MycoBank MB833267.  

*Etymology*: Named after the type species of this series, *A*. *monodii*.  

*Type*: *Aspergillus monodii* (Locq.-Lin.) Varga *et al.*, Stud. Mycol. 69: 91. 2011.  

*Diagnosis*: *Phylogeny*: Series *Monodiorum* belongs to sect. *Usti*, subgen. *Nidulantes* and is phylogenetically sister to a clade containing series *Caldidousti* and *Usti* (Fig. 16). *Morphology & physiology*: Colonies restricted; conidiophores not observed on various media after cultivation at different temperatures; Hülle cells present, surrounding the ascomata, globose to ellipsoidal. *Sexual morph* fennellia-type, homothallic; ascospores hyaline, with two equatorial ridges, convex smooth. Series description based on Samson *et al.* (2011b).  

*Included species*: *Aspergillus monodii*.  

*Extrolites*: Terrein has been detected in *A*. *monodii* (Samson *et al.* 2011b).  

Series ***Usti*** Houbraken & Frisvad, ***ser*. *nov*.** MycoBank MB833268.  

*Etymology*: Named after the type species of this series, *A*. *ustus*.  

*Type*: *Aspergillus ustus* (Bainier) Thom & Church, Aspergilli: 152. 1926.  

*Diagnosis*: *Phylogeny*: Series *Usti* belongs to sect. *Usti*, subgen. *Nidulantes* and is phylogenetically sister to a clade containing ser. *Caldidousti* (Fig. 16). *Morphology & physiology*: Colonies growing moderately or fast, conidia *en masse* in shades of brown; conidiophores biseriate, stipes (pale) brown or reddish brown pigmented, smooth and occasionally having warts; Hülle cells generally present (absent in *A*. *pseudoustus*), predominantly elongate, occasionally irregularly globose or ovoid (*A*. *granulosus*). *Sexual morph* unknown, except in *A*. *heterothallicus*, emericella-type, heterothallic; ascospores orange-brown, with two equatorial ridges, convex smooth. Series description based on Raper and Fennell, 1965, Samson et al., 2011b, Nováková et al., 2012, Visagie et al., 2014a.  

*Included species*: *Aspergillus baeticus*, *A*. *granulosus*, *A*. *heterothallicus*, *A*. *porphyreostipitatus*, *A*. *pseudoustus*, *A*. *puniceus*, *A*. *ustus*.  

*Extrolites*: Of the six species examined chemically in this series four produce asperugins, three produce ustic acid, three produce nidulol, two produce austocystins, two produce drimans, one produces austamide, one produces austdiol, one produces austocystins (and versicolorins), one produces deoxybrevianamides, one produces emethallicin, one produces emeheterone, one produces emesterone, one produces Mer-NF8054X, one produces ophiobolin G & H, one produces stellatin, and one produces sterigmatocystin (Samson *et al.* 2011b, and references therein).  

*Notes on sect*. *Usti and included series*: No asexual morph is observed in ser. *Monodiorum* and only the sexual morph is produced (*A*. *monodii* is homothallic). Sexual reproduction is also present in one member in ser. *Usti* (*A*. *heterothallicus*, heterothallic); all taxa in series *Calidousti* and *Deflecti* reproduce strictly asexually. Species classified in series *Deflecti* and *Monodiorum* grow more restricted than those in series *Calidousti* and *Usti*. Series *Calidousti* and *Usti* cannot be differentiated using phenotypic characters and are primary separated based on phylogenetic data. Members of these series grow moderately fast, produce conidia in shades of brown and have biseriate conidiophores. Some species produce Hülle cells or are able to grow at 37 °C, though these characters cannot be linked to a specific series. Future studies might give more insight whether certain phenotypic characters are shared by the taxa of these series.  

***Aspergillus* subgen. *Polypaecilum*** Samson *et al.*, Stud. Mycol. 85: 211. 2016. MycoBank MB819184.  

*Type*: *Polypaecilum insolitum* G. Sm., Trans. Brit. Mycol. Soc. 44: 437. 1961 (= *Aspergillus insolitus*).  

*Description*: See Kocsubé et al., 2016, Tanney et al., 2017(morphology, phylogeny),  

**Section *Polypaecilum*** Houbraken & Frisvad, ***sect*. *nov*.** MycoBank MB833038.  

*Etymology*: Named after the genus *Polypaecilum*.  

*Type*: *Polypaecilum insolitum* G. Sm., Trans. Brit. Mycol. Soc. 44: 437. 1961 (= *Aspergillus insolitus*).  

*Diagnosis*: *fide*Kocsubé *et al.* (2016): Conidia formed on reduced phialides (as in *Phialosimplex salinarum*; Greiner *et al.* 2014, appearing as phialide collula only), small phialides with long collula often with a thickened centre part (like in *Phialosimplex caninus*; Sigler *et al.* (2010)) or on polyphialides (as in *Polypaecilum insolitum*; Smith (1961)), with the common theme of a thin, long collulum producing chains of conidia that are large compared to the diameter of the collulum. *Aspergillus* conidiophores not produced. The species are halophilic or osmophilic (Wheeler et al., 1988, Wheeler and Hocking, 1993, Greiner et al., 2014, Piñar et al., 2015, Piñar et al., 2016). The sect. *Polypaecilum* includes species of the previously known genera *Polypaecilum* and *Phialosimplex*.  

Series ***Canini*** Houbraken & Frisvad, ***ser*. *nov*.** MycoBank MB833039.  

*Etymology*: Named after the type of the series, *Phialosimplex caninus*.  

*Type*: *Phialosimplex caninus* Sigler *et al.*, Med. Mycol. 48: 338. 2010 (= *Aspergillus caninus*).  

**Fig. 17:**
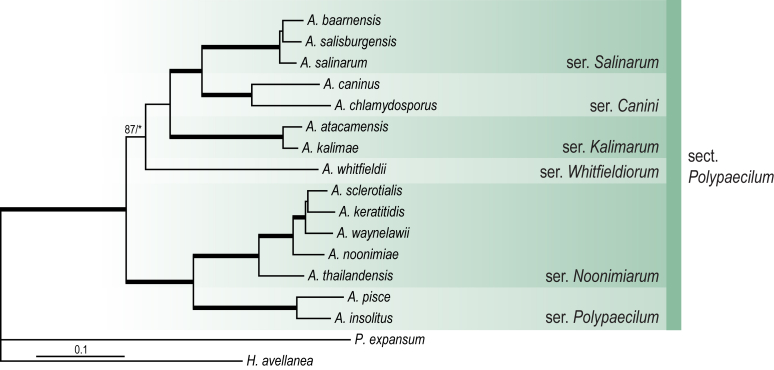
Combined phylogeny for *BenA*, *CaM* and *RPB2* data sets showing the phylogenetic relation of species and series within *Aspergillus* subgen. *Polypaecilum*. The BI posterior probability (pp) values and bootstrap percentages of the maximum likelihood (ML) analysis are presented at the nodes; fully supported branches are thickened. Values less than 70 % bootstrap support (ML) or less than 0.95 posterior probability (Bayesian analysis) are indicated with a hyphen or not shown. The bar indicates the number of substitutions per site. The phylogram is rooted with *Hamigera avellanea* and *Penicillium expansum*.

*Diagnosis*: *Phylogeny*: Series *Canini* belongs to sect. *Polypaecilum*, subgen. *Polypaecilum* and is phylogenetically sister to ser. *Salinarum* (Fig. 17). *Morphology & physiology*: Colonies on CYA restricted, growth on yeast malt extract agar (YMA) moderate, growth on YMA with 20 % NaCl absent, conidia *en masse* yellowish white to orange grey; conidiogenous cells phialides (monophialidic; sometimes proliferating form a second opening, polyphialidic), simple, borne laterally on the vegetative hyphae or occasionally on short, unbranched conidiophores, 3–16 μm long; conidia in heads or chains; chlamydospores absent (*A*. *caninus*) or present (*A*. *chlamydosporus*), on short unbranched or branched stalks; growth on MEA at 37 °C. *Sexual morph* unknown, sclerotia not observed in culture. Xerotolerant and having potential to cause opportunistic disseminated mycoses in dogs. Series description based on Sigler et al., 2010, Martinelli et al., 2017, Tanney et al., 2017.

*Included species*: *Aspergillus caninus*, *A*. *chlamydosporus*.  

*Extrolites*: No extrolites are reported for the species in this series.  

Series ***Kalimarum*** Houbraken & Frisvad, ***ser*. *nov*.** MycoBank MB833040.  

*Etymology*: Named after the type of the series, *A*. *kalimae*.  

*Type*: *Aspergillus kalimae* Tanney *et al.*, Stud. Mycol. 88: 249. 2017.  

*Diagnosis*: *Phylogeny*: Series *Kalimarum* belongs to sect. *Polypaecilum*, subgen. *Polypaecilum* and is phylogenetically sister to series *Canini* and *Salinarum* (Fig. 17). *Morphology & physiology*: No growth or at most germination on CYA or MEA, growth on YMA absent or very restricted, optimal growth on YMA with 15 % NaCl, growth present on YMA with 25 % NaCl; conidia *en masse* white, typical *Aspergillus* conidiophores lacking; conidiogenous cells monophialidic, polyphialides sometimes present, simple, solitary, borne laterally or terminally on hyphae, 3–30 μm long; conidia solitary, in chains or (small) heads; chlamydospores absent (*A*. *kalimae*) or present (*A*. *atacamensis*); no growth at 37 °C, halotolerant. *Sexual morph* unknown; sclerotia absent. Series description based on Martinelli et al., 2017, Tanney et al., 2017.  

*Included species*: *Aspergillus atacamensis*, *A*. *kalimae*.  

*Extrolites*: No extrolites are reported for the species in this series.  

Series ***Noonimiarum*** Houbraken & Frisvad, ***ser*. *nov*.** MycoBank MB833041.  

*Etymology*: Named after the type of the series, *A*. *noonimiae*.  

*Type*: *Aspergillus noonimiae* Tanney *et al.*, Stud. Mycol. 88: 252. 2017.  

*Diagnosis*: *Phylogeny*: Series *Noonimiarum* belongs to sect. *Polypaecilum*, subgen. *Polypaecilum* and is phylogenetically sister to ser. *Polypaecilum* (Fig. 17). *Morphology & physiology*: Colonies restricted on CYA and MEA, growth absent or only germination on MEA with 20 % NaCl; growth at 37 °C; conidia, when produced, white *en masse*; typical *Aspergillus* conidiophores lacking; conidiogenous cells monophialidic, polyphialides sometimes present, simple, solitary, borne laterally or terminally on hyphae, 2.5–25 μm long; conidia solitary or in chains, with persistent membranous sheath. *Sexual morph* unknown; sclerotia absent or present in culture (*A*. *sclerotialis*, *A*. *keratitidis*). Series description based on Sigler et al., 2010, Tanney et al., 2017.  

*Included species*: *Aspergillus keratitidis*, *A*. *noonimiae*, *A*. *sclerotialis*, *A*. *thailandensis*, *A*. *waynelawii*.  

*Extrolites*: No extrolites are reported for the species in this series.  

Series ***Polypaecilum*** Houbraken & Frisvad, ***ser*. *nov*.** MycoBank MB833037.  

*Etymology*: Named after the genus *Polypaecilum*.  

*Type*: *Polypaecilum insolitum* G. Sm., Trans. Brit. Mycol. Soc. 44: 437. 1961 (= *Aspergillus insolitus*).  

*Diagnosis*: *Phylogeny*: Series *Polypaecilum* belongs to sect. *Polypaecilum*, subgen. *Polypaecilum* and is phylogenetically sister to ser. *Noonimiarum* (Fig. 17). *Morphology & physiology*: Colonies restricted on CYA and MEA, growth absent or only germination on MEA with 20 % NaCl; conidia *en masse* white; typical *Aspergillus* conidiophores lacking, reproductive structures polyphialides, born solitary on short conidiophores, polyphialides large and complex, 15–60 μm long; chlamydospores absent (*A*. *pisce*) or present (*A*. *insolitus*). *Sexual morph* unknown; sclerotia absent (*A*. *insolitus*) or present (*A*. *pisce*). Isolated from hypersaline habitats. Series description based on Smith, 1961, Pitt and Hocking, 2009, Tanney et al., 2017.  

*Included species*: *Aspergillus insolitus*, *A*. *pisce*.  

*Extrolites*: No extrolites are reported for the species in this series.  

Series ***Salinarum*** Houbraken & Frisvad, ***ser*. *nov*.** MycoBank MB833042.  

*Etymology*: Named after the type of the series, *A*. *salinarum*.  

*Type*: *Aspergillus salinarum* (Greiner *et al.*) Zalar & Greiner, Extremophiles 21: 762. 2017.  

*Diagnosis*: *Phylogeny*: Series *Salinarum* belongs to sect. *Polypaecilum*, subgen. *Polypaecilum* and is phylogenetically sister to ser. *Canini* (Fig. 17). *Morphology & physiology*: No growth or only germination on CYA, growth on YMA absent or very restricted, optimal growth on YMA with 15 % NaCl, growth present on YMA with 25 % NaCl; conidia *en masse* white, typical *Aspergillus* conidiophores lacking, conidiogenous cells monophialidic, polyphialides absent, simple, solitary, borne laterally or terminally on hyphae, 3–30 μm long; conidia solitary, in chains or (small) heads; chlamydospores absent (*A*. *baarnensis*, *A*. *loretoensis*) or present (*A*. *salinarum*, *A*. *salisburgensis*); halotolerant, or (obligate) halophilic. *Sexual morph* unknown; sclerotia absent. Series description based on Martinelli et al., 2017, Tanney et al., 2017, González-Martínez et al., 2019.  

*Included species*: *Aspergillus baarnensis*, *A*. *loretoensis*∗, *A*. *salinarum*, *A*. *salisburgensis* [∗not included in Fig. 17; details on classification, see González-Martínez *et al.* (2019)].  

*Extrolites*: No extrolites are reported for the species in this series.  

Series ***Whitfieldiorum*** Houbraken & Frisvad, ***ser*. *nov*.** MycoBank MB833043.  

*Etymology*: Named after the type of the series, *A*. *whitfieldii*.  

*Type*: *Aspergillus whitfieldii* Tanney *et al.*, Stud. Mycol. 88: 258. 2017.  

*Diagnosis*: *Phylogeny*: Series *Whitfieldiorum* belongs to sect. *Polypaecilum*, subgen. *Polypaecilum* and is phylogenetically related to series *Canini*, *Kalimarum* and *Salinarum*; the exact phylogenetic relationship with those series is unresolved (Fig. 17). *Morphology & physiology*: Colonies restricted, no growth on MEA with 20 % NaCl, no growth at 37 °C; sporulation sparse; typical *Aspergillus* conidiophores lacking, conidiogenous cells mono- to polyphialidic, solitary, borne laterally or terminally on vegetative hyphae, sometimes occurring in hyphal networks resembling branched conidiophores, 4–36 μm; conidia solitary or in chains, with persistent membranous sheath; chlamydospores absent. *Sexual morph* unknown; sclerotia not observed in culture. Series description based on Tanney *et al.* (2017).  

*Included species*: *Aspergillus whitfieldii*.  

*Extrolites*: No extrolites are reported for the species in this series.  

*Notes on sect*. *Polypaecilum and included series*: We did not find significant differences within subgen. *Polypaecilum* that would warrant it to split the subgenus in sections. Section *Polypaecilum* is therefore the sole section in subgen. *Polypaecilum*, hence, both have the same description. However, there are few differences within sect. *Polypaecilum* and we choose to introduce six series (series *Canini*, *Kalimarum*, *Noonimiarum*, *Polypaecilum*, *Salinarum* and *Whitfieldiorum*). Series *Polypaecilum* forms a strongly supported clade and can be distinguished from the other series by their large and more complexly branched polyphialides. Sclerotia are produced by species in series *Noonimiarum* (*A*. *sclerotialis*, *A*. *keratitidis*) and *Polypaecilum* (*A*. *pisce*), and were not observed in the other series. The conidial chains of some species of subgen. *Polypaecilum* adhered within a persistent membranous sheath visible by SEM and light microscopy. This sheath is present in series *Noonimiarum* (*A*. *keratitidis*, *A*. *noonimiae*, *A*. *sclerotialis*, *A*. *waynelawii*) and *Kalimarum* (*A*. *kalimae*). The production of chlamydospores is distributed over subgen. *Polypaecilum* and not restricted to any specific series. The two species accommodated in ser. *Canini* were previously classified in the genus *Phialosimplex*. These species share the ability to grow at 37 °C and have the potential to cause opportunistic disseminated mycoses in dogs. Furthermore, ser. *Canini* species are unable to grow on MY10-12, and all other species in subgen. *Polypaecilum* can (Tanney *et al.* 2017). These species are xerotolerant, while the members of the phylogenetically related series *Salinarum* and *Kalimarum* are considered halotolerant (Sigler et al., 2010, Martinelli et al., 2017, Tanney et al., 2017). The phylogenetic position of *A*. *whitfieldii* (the sole species in ser. *Whitfieldiorum*) is unresolved and more research is needed to determine its exact phylogenetic relationship with the other series of subgen. *Polypaecilum* (Fig. 17, Tanney *et al.* 2017).

 Like in many other extremophilic fungi, secondary metabolite production is not common in species of subgen. (and sect.) *Polypaecilum*. For example, *Xeromyces bisporus* does not produce a single family of secondary metabolites (Leong *et al.* 2015). On the other hand, species in sections *Aspergillus* and *Restricti* produce a large number of secondary metabolites, as do other species in most of the sections of *Aspergillus*.  

***Penicillium* subgen. *Aspergilloides*** Dierckx, Ann. Soc. Sci. Bruxelles. 25: 85. 1901. MycoBank MB833420.  

*Type*: *Penicillium aurantiobrunneum* Dierckx, Ann. Soc. Sci. Bruxelles 25: 86. 1901 (= *Penicillium glabrum*).  

*Description*: See Houbraken & Samson (2011) (morphology, phylogeny); Fig. 1, this study (phylogeny).  

**Section *Alfrediorum*** Houbraken & Frisvad, ***sect*. *nov*.** MycoBank MB834239.  

*Etymology*: Named after the type species of the series, *Penicillium alfredii*.  

*Type*: *Penicillium alfredii* Visagie *et al.*, Stud. Mycol. 78: 116. 2014.  

**Fig. 18:**
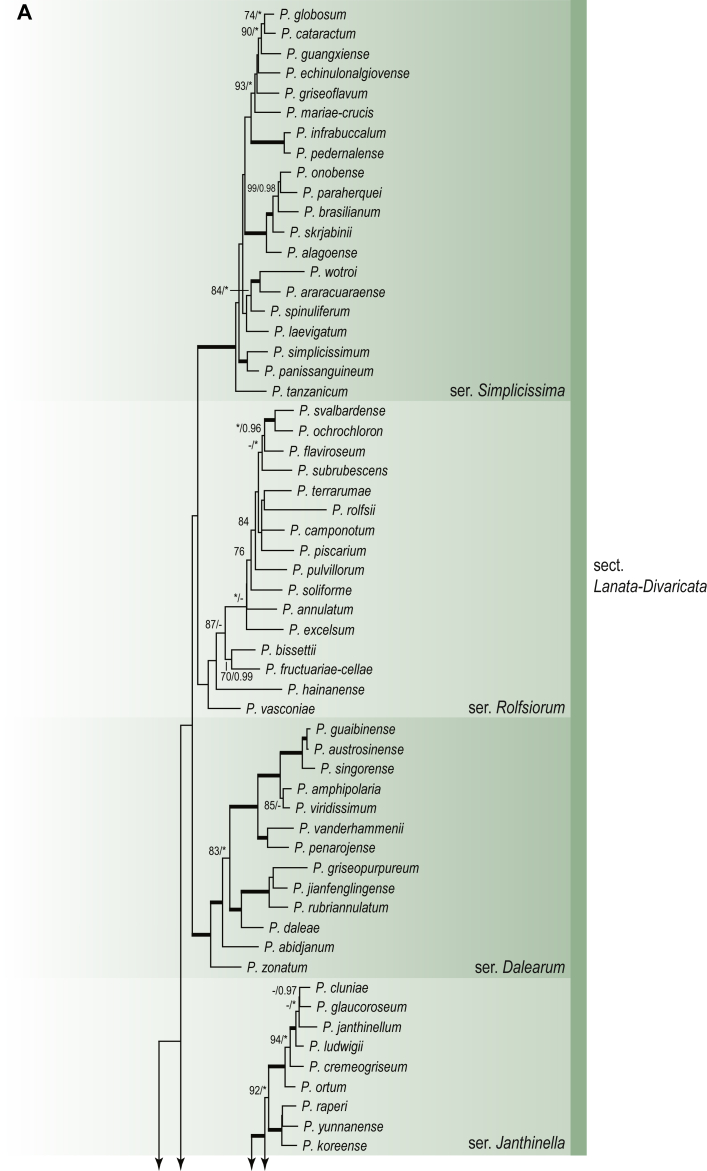

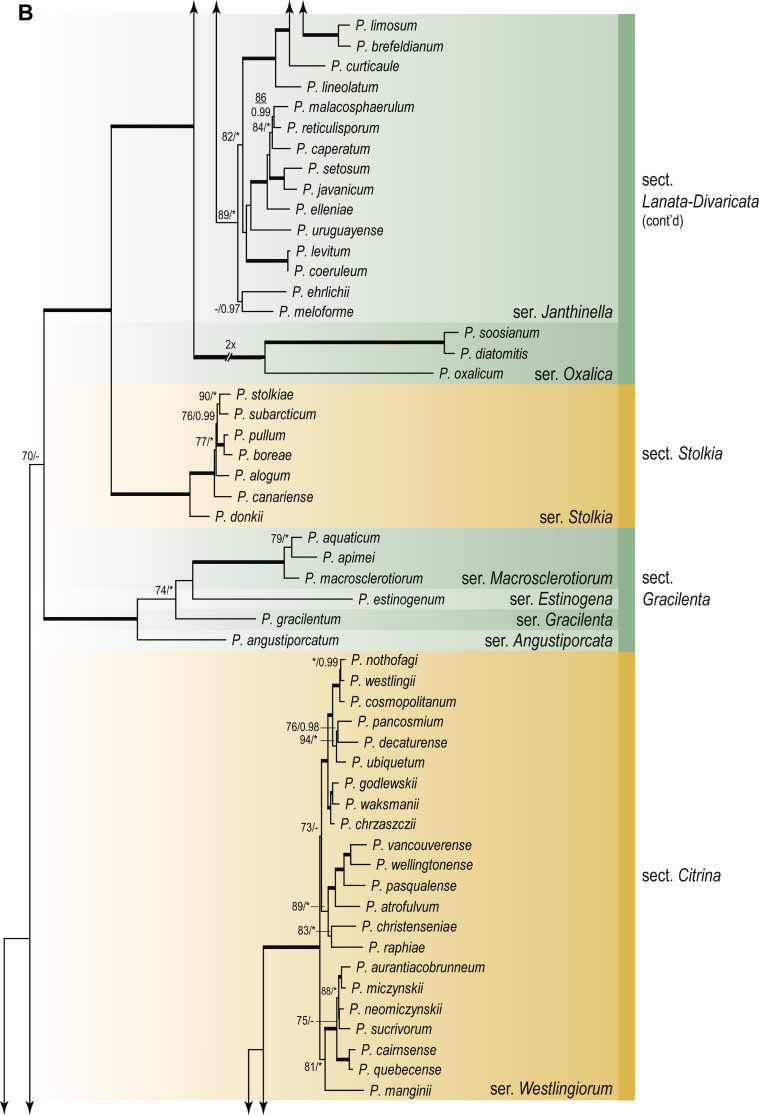

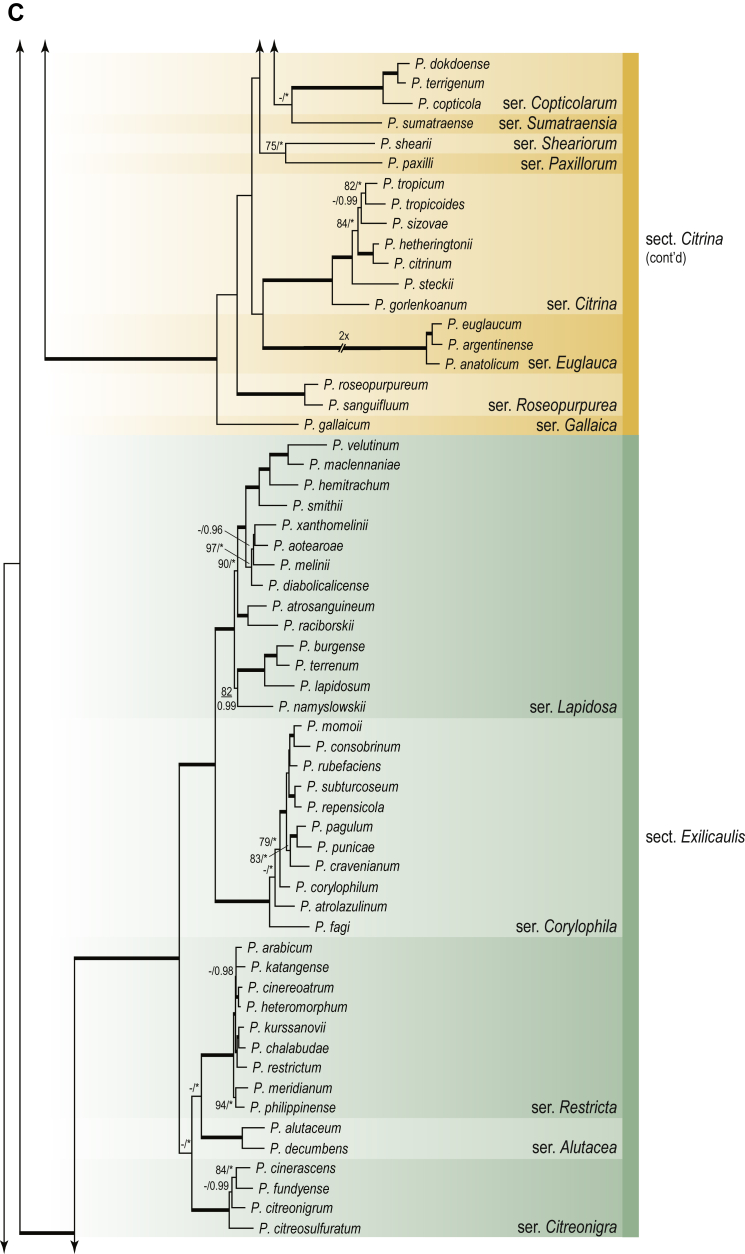

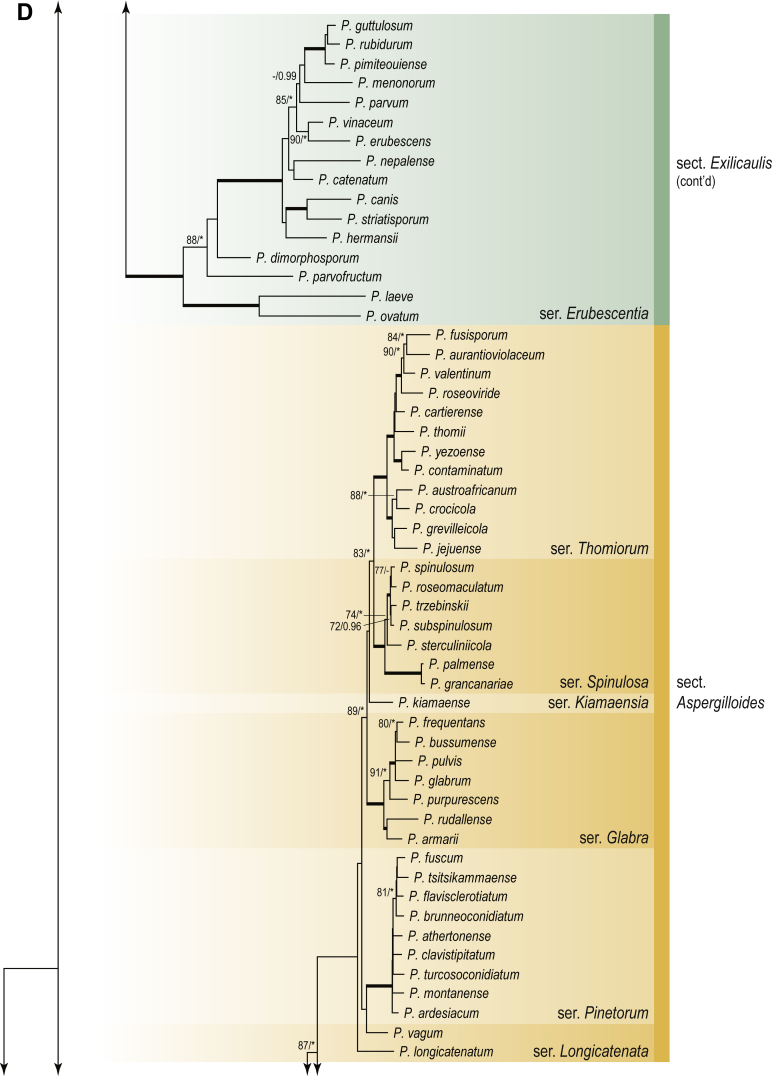

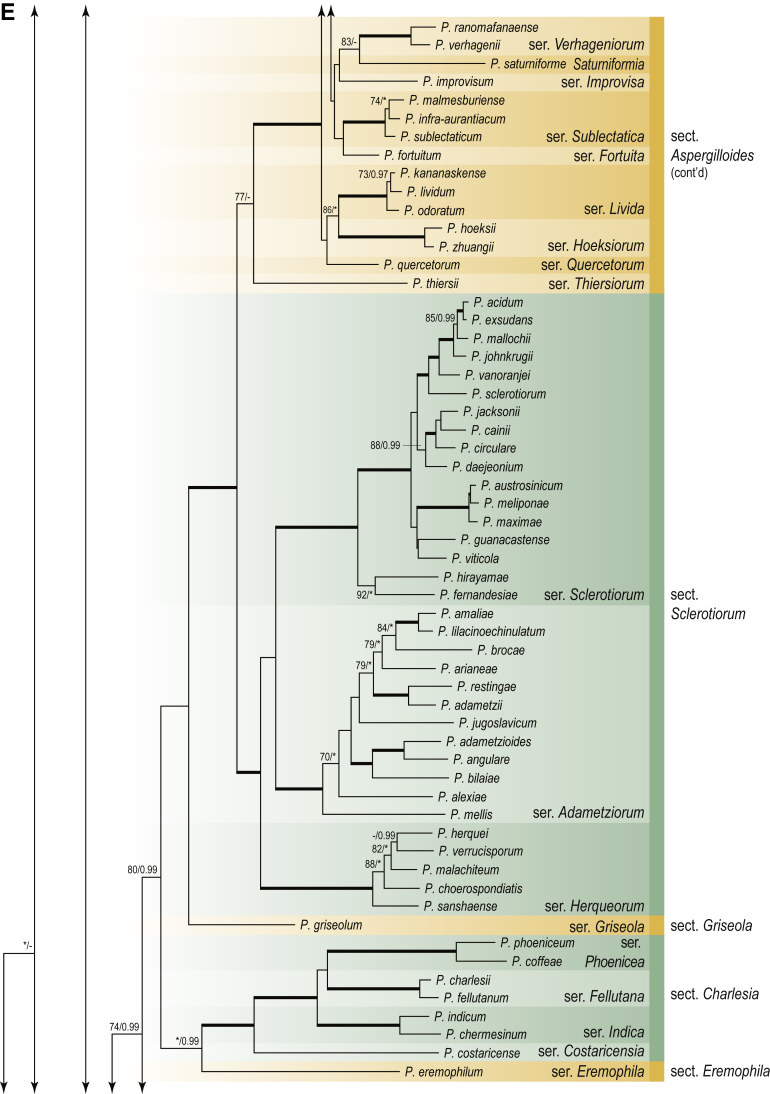

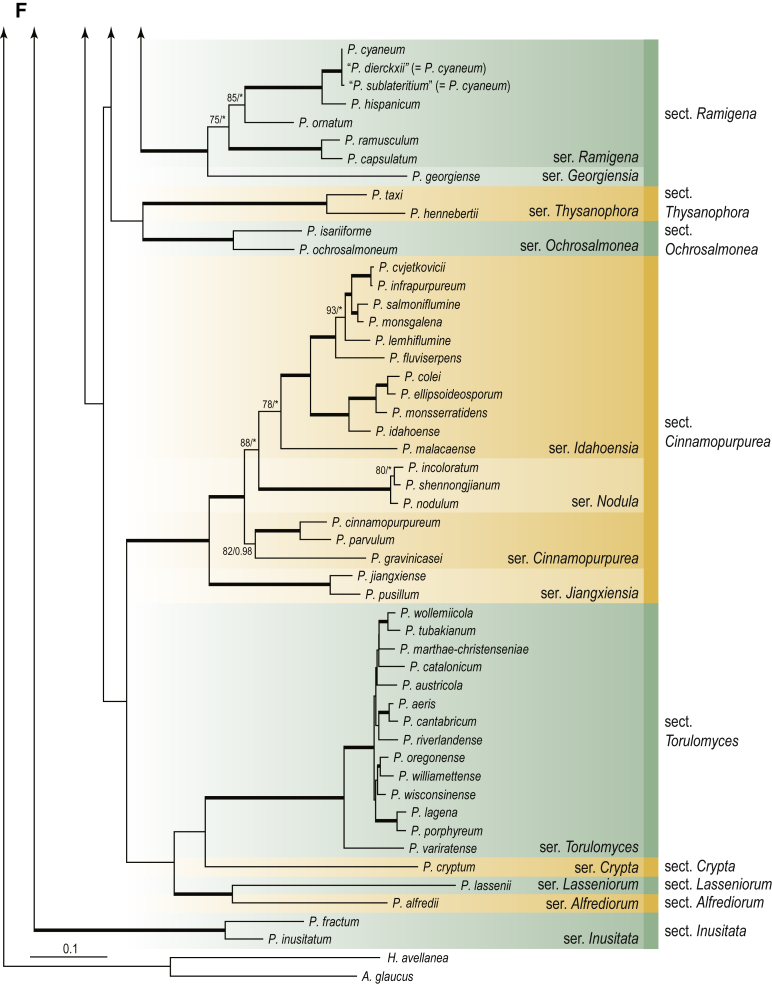
Combined phylogeny for *BenA*, *CaM* and *RPB2* data sets showing the phylogenetic relation of species, series and sections within *Penicillium* subgen. *Aspergilloides*. The BI posterior probability (pp) values and bootstrap percentages of the maximum likelihood (ML) analysis are presented at the nodes; fully supported branches are thickened. Values less than 70 % bootstrap support (ML) or less than 0.95 posterior probability (Bayesian analysis) are indicated with a hyphen or not shown. The bar indicates the number of substitutions per site. The phylogram is rooted with *Aspergillus glaucus* and *Hamigera avellanea*.

*Diagnosis*: *Phylogeny*: Section *Alfrediorum* belongs to subgen. *Aspergilloides* and is phylogenetically most closely related to sect. *Lasseniorum* (Fig. 2, Fig. 18). *Morphology & physiology*: Colonies restricted; conidial colour *en masse* greyish green; conidiophores monoverticillate, smooth; no growth at 37 °C. *Sexual morph* unknown; sclerotia not produced in culture. Section description based on Visagie *et al.* (2014a).

*Included species*: *Penicillium alfredii*.  

*Extrolites*: No known extrolites detected.  

*Notes*: *Penicillium alfredii* is the sole species in sect. *Alfredii*. The phylogenetic position of this species remained unresolved and it could previously not be classified properly in any of the known sections (Visagie *et al.* 2014a). *Penicillium alfredii* colonies resemble those of species in sect. *Torulomyces*; however, sect. *Torulomyces* species generally produce conidiophores that have solitary phialides and these are not observed in *P*. *alfredii* (Visagie *et al.* 2014a). A subdivision of the section cannot be made and therefore ser. *Alfrediorum* is only informally introduced here.  

**Section *Aspergilloides*** Pitt, The Genus Penicillium: 169. 1980 [1979]. MycoBank MB832951.

*Synonym*: *Eupenicillium* sect. *Pinetorum* (Pitt) Stolk & Samson, Stud. Mycol. 23: 88. 1983.  

*Type*: *Penicillium aurantiobrunneum* Dierckx, Ann. Soc. Sci. Bruxelles 25: 86. 1901 (= *Penicillium glabrum*).  

*Description*: See Houbraken and Samson, 2011, Houbraken et al., 2014b (phylogeny, morphology).  

Series ***Glabra*** Pitt, The Genus Penicillium: 169. 1980 [1979]. MycoBank MB832952.  

*Type*: *Penicillium glabrum* (Wehmer) Westling, Ark. Bot. 11: 131. 1911.  

*Diagnosis*: *Phylogeny*: Series *Glabra* belongs to subgen. *Aspergilloides*, sect. *Aspergilloides* and forms a unique lineage sister to series *Kiamaensia*, *Spinulosa* and *Thomiorum* (Fig. 18). *Morphology & physiology*: Colonies spreading on CYA, MEA and YES, texture velvety; conidial colour *en masse* dark green; conidiophores monoverticillate with vesiculate apex, smooth; conidia ornamented, finely to distinctly rough-walled, globose to subglobose; on CREA often weak growth (except *P*. *armarii*) and moderate acid production. *Sexual morph* unknown; sclerotia not observed in culture. Series description based on Houbraken *et al.* (2014b).  

*Included species*: *Penicillium armarii*, *P*. *bussumense*, *P*. *frequentans*, *P*. *glabrum*, *P*. *pulvis*, *P*. *purpurescens*, *P*. *rudallense*.  

*Extrolites*: The very common species *P*. *glabrum* (as *Citromyces glaber*) has been reported to produce citric acid (Wehmer, 1893, Raper and Thom, 1949) and members of the geodin biosynthetic family, including asterric acid, bis-dechlororogeodin, questin, questinol, and sulochrin (Mahmoodian & Stickings 1964). We did not detect any members of the sulochrin biosynthetic family in *P*. *frequentans*, but a series of extrolites with unique UV spectra. *Penicillium rudallense* has been reported to produce a large number of austalides (Wang *et al.* 2019a), but we detected nigragillin and pyranonigrins in this species. Asterric acid, sulochrin and pyranonigrin A was detected in *P*. *bussumense*; *P*. *pulvis* produced asterric acid, some red anthraquionones, sulochrin, pyranonigrins and spinulosin.

 Frequentin production was reported from a fungus identified as an atypical strain of *P*. *frequentans* CBS 345.51 (Curtis *et al.* 1951), but this strain is correctly identified as *P*. *subspinulosum* (Houbraken *et al.* 2014b). Species in this series have not been systematically studied chemotaxonomically. Austalides have also been reported in *P*. *thomii* in ser. *Thomiorum* and geodins were reported from *P*. *lividum* from ser. *Livida* (Sobolevskaya *et al.* 2014), but the identity of those isolates has not been confirmed.  

Series ***Fortuita*** Houbraken & Frisvad, ***ser*. *nov*.** MycoBank MB834240.  

*Etymology*: Named after the type species of the series, *Penicillium fortuitum*.  

*Type*: *Penicillium fortuitum* Visagie & Seifert, Persoonia 41: 387. 2018.  

*Diagnosis*: *Phylogeny*: Series *Fortuita* belongs to subgen. *Aspergilloides*, sect. *Aspergilloides* and forms a single species lineage sister to ser. *Sublectatica*, though statistical support for this relationship is lacking. *Morphology & physiology*: Colonies restricted; conidial colour *en masse* greyish green; conidiophores predominantly monoverticillate, occasionally divaricate, smooth; no growth at 37 °C. *Sexual morph* unknown; sclerotia not observed in culture. Series description based on Crous *et al.* (2018a).  

*Included species*: *Penicillium fortuitum*.  

*Extrolites*: No chemotaxonomic data on *Penicillium fortuitum* is known.  

Series ***Hoeksiorum*** Houbraken & Frisvad, ***ser*. *nov*.** MycoBank MB834241.  

*Etymology*: Named after the type species of the series, *Penicillium hoeksii*.  

*Type*: *Penicillium hoeksii* Houbraken, Stud. Mycol. 78: 423. 2014.  

*Diagnosis*: *Phylogeny*: Series *Hoeksiorum* belongs to subgen. *Aspergilloides*, sect. *Aspergilloides* and is sister to ser. *Livida* (Fig. 18). *Morphology & physiology*: Colonies growing moderately; conidial colour *en masse* grey or blue green; conidiophores monoverticillate, in older parts divaricate, smooth, generally shorter than 250 μm; conidia ellipsoidal, finely rough-walled; no growth at 30 and 37 °C. *Sexual morph* unknown; sclerotia not observed in culture. Series description based on Houbraken *et al.* (2014b).  

*Included species*: *Penicillium hoeksii*, *P*. *zhuangii*.  

*Extrolites*: *Penicillium hoeksii* produces fulvic acids and haenamindole, but no chemotaxonomic data is available for *P*. *zhuangii*.  

Series ***Improvisa*** Houbraken & Frisvad, ***ser*. *nov*.** MycoBank MB834242.  

*Etymology*: Named after the type species of the series, *Penicillium improvisum*.  

*Type*: *Penicillium improvisum* Visagie *et al.*, Persoonia 36: 256. 2016.  

*Diagnosis*: *Phylogeny*: Series *Improvisa* belongs to subgen. *Aspergilloides* sect. *Aspergilloides* and the phylogenetic relationship of this single species is unresolved (Fig. 18). *Morphology & physiology*: Colonies growing moderately; conidial colour *en masse* greyish turquoise to greyish green; conidiophores predominantly monoverticillate, sometimes divaricate, smooth; conidia globose to subglobose, smooth; growth at 37 °C absent. *Sexual morph* unknown; sclerotia not observed in culture. Series description based on Crous *et al.* (2016).  

*Included species*: *Penicillium improvisum*.  

*Extrolites*: No chemotaxonomical data on *P*. *improvisum* is available.  

Series ***Kiamaensia*** Houbraken & Frisvad, ***ser*. *nov*.** MycoBank MB834243.  

*Etymology*: Named after the type species of the series, *Penicillium kiamaense*.  

*Type*: *Penicillium kiamaense* Houbraken & Pitt, Stud. Mycol. 78: 426. 2014.  

*Diagnosis*: *Phylogeny*: Series *Kiamaensia* belongs to subgen. *Aspergilloides*, sect. *Aspergilloides* and is, with weak statistical support, sister to series *Spinulosa* and *Thomiorum* (Fig. 18). *Morphology & physiology*: Colonies moderate to spreading; conidial colour *en masse* dark green; conidiophores predominantly monoverticillate, occasionally with a short branch, finely rough-walled; conidia globose or subglobose, ornamented with striations; growth at 30 °C, absent at 37 °C. *Sexual morph* unknown; sclerotia not observed in culture. Series description based on Houbraken *et al.* (2014b).  

*Included species*: *Penicillium kiamaense*.  

*Extrolites*: *Penicillium kiamense* produces some anthraquinones and some extrolites that are not yet structure elucidated.  

Series ***Livida*** Houbraken & Frisvad, ***ser*. *nov*.** MycoBank MB834244.  

*Etymology*: Named after the type species of the series, *Penicillium lividum*.  

*Type*: *Penicillium lividum* Westling, Ark. Bot. 11: 134. 1911.  

*Diagnosis*: *Phylogeny*: Series *Livida* belongs to subgen. *Aspergilloides*, sect. *Aspergilloides* and is sister to ser. *Hoeksiorum* (Fig. 18). *Morphology & physiology*: Colonies growing moderately; conidial colour *en masse* (dark) blue-green; conidiophores monoverticillate, rough-walled, longer than 250 μm; conidia broadly ellipsoidal or ellipsoidal, distinctly roughened, often striate; growth at 30 °C, absent at 37 °C. *Sexual morph* unknown; sclerotia not produced in culture. Series description based on Pitt (1980) and Houbraken *et al.* (2014b).  

*Included species*: *Penicillium kananaskense*, *P*. *lividum*, *P*. *odoratum*.  

*Extrolites*: *Penicillium odoratum* has been reported to produce citrinin (Nakajima & Nozawa 2004), and this has been confirmed here. *Penicillium kananaskense* produces citreoisocoumarins and a large number of unique extrolites that are not yet structure elucidated. *Penicillium lividum* has been reported to produce austalides, daldinin D, peniciraistin C, questin and sulochrin (Sobolevskaya et al., 2014, Zhuravleva et al., 2014a, Zhuravleva et al., 2014b, Sobolevskaya et al., 2016b). We could not confirm this, but found some unique not yet structure elucidated extrolites for *P*. *lividum*. The three species in ser. *Livida* have no extrolites in common.  

Series ***Longicatenata*** Houbraken & Frisvad, ***ser*. *nov*.** MycoBank MB834245.  

*Etymology*: Named after the type species of the series, *Penicillium longicatenatum*.  

*Type*: *Penicillium longicatenatum* Visagie *et al.*, Stud. Mycol. 78: 429. 2014.  

*Diagnosis*: *Phylogeny*: Series *Longicatenata* belongs to subgen. *Aspergilloides*, sect. *Aspergilloides*; the two species in the series are related in the phylogeny of Houbraken *et al.* (2014b) with moderate statistical support (81 % BS, 0.96 pp); however, their relationship is unresolved in this study (Fig. 18). *Morphology & physiology*: Colonies growing moderate or spreading; conidial colour *en masse* in shades of green (greyish green, dull green or dark green); colony texture velvety or floccose; conidiophores monoverticillate, smooth; conidia (sub)globose, finely or distinctly rough-walled; generally no growth at 37 °C or sometimes restricted growth (<4 mm, 7 d, CYA). *Sexual morph* unknown, sclerotia absent (*P*. *vagum*) or present (*P*. *longicatenatum*). Series description based on Houbraken *et al.* (2014b).  

*Included species*: *Penicillium longicatenatum*, *P*. *vagum*.  

*Extrolites*: *Penicillium longicatenatum* produced kotanins, palitantin, spinulosin and a versicolorin, while the ex-type culture of *P*. *vagum* produces citrinin. Other strains supposed to be *P*. *vagum* were chemotaxonomically very different: DTO 038-E7 and DTO 056-I6 produced spinulosin and daldinins, while DTO 120-B1 and DTO 120-B4 produced asperfuran, dehydrocarolic acid, frequentin and palitantin. This indicates that *P*. *vagum* may be split into three species.  

Series ***Pinetorum*** (Pitt) Houbraken & Frisvad, ***comb*. *nov*.** MycoBank MB834246.

*Basionym*: *Eupenicillium* ser. *Pinetorum* Pitt, The Genus Penicillium: 105. 1980 [1979]. MycoBank MB832947.  

*Type*: *Eupenicillium pinetorum* Stolk, Antonie van Leeuwenhoek 34: 37. 1968 (= *Penicillium fuscum*).  

*Diagnosis*: *Phylogeny*: Series *Pinetorum* belongs to subgen. *Aspergilloides*, sect. *Aspergilloides* and is unresolved, but forms a sister lineage to series *Glabra*, *Kiamaensia*, *Spinulosa* and *Thomiorum* (Fig. 18). *Morphology & physiology*: Colonies growing restrictedly or moderately rapid; conidial colour *en masse* variable (in shades of green); conidiophores monoverticillate, generally smooth, sometimes rough-walled (*P*. *clavistipitatum*), short; conidia globose to subglobose, distinctly rough-walled, thick; no growth at 37 °C. *Sexual morph* generally not produced in culture, except for *P*. *fuscum*, eupenicillium-type, homothallic, greyish yellow; ascospores ellipsoidal, with two longitudinal flanges, convex smooth-walled; sclerotia generally absent, except in *P*. *flavisclerotiatum* (yellow) and *P*. *tsitsikammaense* (white). Series description based on Pitt (1980) and Houbraken *et al.* (2014b).  

*Included species*: *Penicillium ardesiacum*, *P*. *athertonense*, *P*. *brunneoconidiatum*, *P*. *clavistipitatum*, *P*. *flavisclerotiatum*, *P*. *fuscum*, *P*. *montanense*, *P*. *tsitsikammaense*, *P*. *turcosoconidiatum*.  

*Extrolites*: The species in ser. *Pinetorum* are chemotaxonomically quite different. *Penicillium athertonense* produces viridicatins, *P*. *flavisclerotiatum* DTO 184-D8 produces burnettienes, while the isolates DTO 180-I1 and DTO 181-I9 produce asperfuran. *Penicillium fuscum* produces asperfuran, while isolates allocated to *P*. *lapatayae* produce kotanins, lapatins and spinulosins indicating *P*. *lapatayae* is a separate species. However, we follow Houbraken *et al.* (2014b) and consider this species as a synonym of *P*. *fuscum* based on molecular data. *Penicillium montanense* produces unique extrolites, while *P*. *turcosoconidiatum* (DTO 181-A3) produces citreoviridin and haenamindole.  

Series ***Quercetorum*** Houbraken & Frisvad, ***ser*. *nov*.** MycoBank MB834247.  

*Etymology*: Named after the type species of the series, *Penicillium quercetorum*.  

*Type*: *Penicillium quercetorum* Baghd., Novosti Sist. Nizsh. Rast. 5: 110. 1968.  

*Diagnosis*: *Phylogeny*: Series *Quercetorum* belongs to subgen. *Aspergilloides*, sect. *Aspergilloides* and is sister to series *Hoeksiorum* and *Livida* (Fig. 18). *Morphology & physiology*: Colonies growing moderately; conidial colour *en masse* dull green; conidiophores monoverticillate, smooth-walled; conidia globose, smooth; growth at 37 °C absent. *Sexual morph* not observed in culture; sclerotia orange-brown. Series description based on Houbraken *et al.* (2014b).  

*Included species*: *Penicillium quercetorum*.  

*Extrolites*: No chemotaxonomic data available for *P*. *quercetorum*.  

Series ***Saturniformia*** Houbraken & Frisvad, ***ser*. *nov*.** MycoBank MB834248.  

*Etymology*: Named after the type species of the series, *Penicillium saturniforme*.  

*Type*: *Penicillium saturniforme* (L. Wang & W.Y. Zhuang) Houbraken & Samson, Stud. Mycol. 70: 48. 2011.  

*Diagnosis*: *Phylogeny*: Series *Saturniformia* belongs to subgen. *Aspergilloides*, sect. *Aspergilloides* and is sister to ser. *Verhageniorum* (Fig. 18). *Morphology & physiology*: Colonies growing moderate; conidial colour *en masse* green to greyish olive; conidiophores biverticillate; no growth on CYA incubated at 30 °C; conidia (broadly) ellipsoidal, finely rough-walled. *Sexual morph* eupenicillium-type, homothallic, pinkish brown; ascospores ellipsoidal, with two very closely appressed equatorial ridges, convex smooth with sparsely scattered fine warts or irregular ribs along the outer areas. Series description based on Wang & Zhuang (2009) and Houbraken *et al.* (2014b).  

*Included species*: *Penicillium saturniforme*.  

*Extrolites*: No chemotaxonomic data available for *P*. *saturniforme*.  

Series ***Spinulosa*** Houbraken & Frisvad, ***ser*. *nov*.** MycoBank MB834249.  

*Etymology*: Named after the type species of the series, *Penicillium spinulosum*.  

*Type*: *Penicillium spinulosum* Thom, U.S.D.A. Bur. Animal Industr. Bull. 118: 76. 1910.  

*Diagnosis*: *Phylogeny*: Series *Spinulosa* belongs to subgen. *Aspergilloides*, sect. *Aspergilloides* and is sister to ser. *Thomiorum* (Fig. 18). Colonies spreading on CYA, MEA and YES, texture (slightly) floccose; conidial colour *en masse* mostly pure or dull green; conidiophores monoverticillate with vesiculate apex, smooth; conidia ornamented, finely to distinctly rough-walled, globose to subglobose; on CREA poor or good growth, acid production often absent or poor. *Sexual morph* unknown; sclerotia not observed in culture. Series description based on Houbraken *et al.* (2014b).  

*Included species*: *Penicillium grancanariae*, *P*. *palmense*, *P*. *roseomaculatum*, *P*. *spinulosum*, *P*. *sterculiniicola*, *P*. *subspinulosum*, *P*. *trzebinskii*.  

*Extrolites*: *Penicillium spinulosum* has been reported to produce spinulosin (Birkinshaw and Raistrick, 1931, Anslow and Raistrick, 1938, Pettersson, 1965). The original producing strain, IMI 091950, did not match the description of *P*. *spinulosum* (results reported here). *Penicillium subspinulosum* produces frequentin and palitantin (Houbraken *et al.* 2014b), in common with *P*. *trzebinskii*; *P*. *spinulosum* produces asperfuran, while *P*. *sterculiniicola* produces asperfuran and 12,13-deoxybrevianamide E.  

Series ***Sublectatica*** Houbraken & Frisvad, ***ser*. *nov*.** MycoBank MB834250.  

*Etymology*: Named after the type species of the series, *Penicillium sublectaticum*.  

*Type*: *Penicillium sublectaticum* Houbraken *et al.*, Stud. Mycol. 78: 436. 2014.  

*Diagnosis*: *Phylogeny*: Series *Sublectatica* belongs to subgen. *Aspergilloides*, sect. *Aspergilloides* and is sister to ser. *Fortuita*, though statistical support for this relationship is lacking (Fig. 18). *Morphology & physiology*: Colonies growing moderately fast or spreading; conidial colour *en masse* in various shades of green (greyish green, dark green, dull green); conidiophores monoverticillate; growth on CYA incubated at 30 °C, no growth at 37 °C. *Sexual morph* unknown; sclerotia not observed in culture. Series description based on Houbraken *et al.* (2014b).  

*Included species*: *Penicillium infra-aurantiacum*, *P*. *malmesburiense*, *P*. *sublectaticum*.  

*Extrolites*: *Penicillium infra-aurantiacum* produces citrinin, while *P*. *malmesburiense* produces unique extrolites that have not been structure elucidated yet.  

Series ***Thiersiorum*** Houbraken & Frisvad, ***ser*. *nov*.** MycoBank MB834251.  

*Etymology*: Named after the type species of the series, *Penicillium thiersii*.  

*Type*: *Penicillium thiersii* S.W. Peterson *et al.*, Mycologia 96: 1283. 2004.  

*Diagnosis*: *Phylogeny*: Series *Thiersiorum* belongs to subgen. *Aspergilloides*, sect. *Aspergilloides* and is sister to all other series in the section (Fig. 18). *Morphology & physiology*: Colonies spreading; conidial colour *en masse* dark bluish grey; conidiophores monoverticillate, smooth or slightly roughened; conidia ellipsoidal, smooth; growth absent at 37 °C. *Sexual morph* eupenicillium-type, homothallic, pale brown; ascospores ellipsoidal, with equatorial ridge, smooth or finely roughened convex. Series description based on Peterson *et al.* (2004) and Houbraken *et al.* (2014b).  

*Included species*: *Penicillium thiersii*.  

*Extrolites*: *Penicillium thiersii* produces thiersinines and their precursors such as 1’-O-acetylpaxilline, dehydroxypaxilline, paxilline, paspaline, PC-M5’ and PC-M6 (Li *et al.* 2002), decaturin B, C and D, 15-deoxyoxalicine A, oxalicine A and B (Zhang et al., 2003, Li et al., 2005) and emindole SB and thiersindole A-C (Li et al., 2002, Li et al., 2003).  

Series ***Thomiorum*** Houbraken & Frisvad, ***ser*. *nov*.** MycoBank MB834252.  

*Etymology*: Named after the type species of the series, *Penicillium thomii*.  

*Type*: *Penicillium thomii* Maire, Bull. Soc. Hist. Nat. Afrique N. 8: 189. 1917.  

*Diagnosis*: *Phylogeny*: Series *Thomiorum* belongs to subgen. *Aspergilloides*, sect. *Aspergilloides* and is sister to ser. *Spinulosa* (Fig. 18). *Morphology & physiology*: Colonies spreading on CYA, MEA and YES; conidial colour *en masse* dull green; conidiophores monoverticillate with a vesiculate apex, rough-walled, conidia ellipsoidal or fusiform; growth on CYA incubated at 30 °C, (5–)15–35(–45), no growth at 37 °C. *Sexual morph* unknown; sclerotia commonly produced, in shades of pink (orange-pink, brownish pink). Series description based on Houbraken *et al.* (2014b).  

*Included species*: *Penicillium aurantioviolaceum*, *P*. *austroafricanum*, *P*. *cartierense*, *P*. *contaminatum*, *P*. *crocicola*, *P*. *fusisporum*, *P*. *grevilleicola*, *P*. *jejuense*, *P*. *roseoviride*, *P*. *thomii*, *P*. *valentinum*, *P*. *yezoense*.  

*Extrolites*: *Penicillium thomii* has been reported to produce N-acetylphenylalaninol and the related 2(S)-acetamido-3-phenylpropylacetate, austalides, furan-2-carboxylic acid derivatives, guaidiol A and 4,10,11-trihydroxyguaiane, pallidopenillines, penistinraistin C and the related daldinin D, sargassopenillines, thomimarides, thomimarines, VM55599 and zesteropenillines (Sobolevskaya et al., 2014, Zhuravleva et al., 2014a, Zhuravleva et al., 2014b, Sobolevskaya et al., 2016a, Sobolevskaya et al., 2016b, Sobolevskaya et al., 2018, Afiyatullov et al., 2017a, Afiyatullov et al., 2017b, Afiyatullov et al., 2018). *Penicillium austroafricanum* produces fumagillin and *P*. *auirantioviolaceum* produces spinulosins. Five species in series *Thomiorum* produce haenamindole: *P*. *cartierense*, *P*. *contaminatum*, *P*. *crocicola*, *P*. *roseoviride* and *P*. *yezoense*; *P*. *contaminatum* also produces palitantin.  

Series ***Verhageniorum*** Houbraken & Frisvad, ***ser*. *nov*.** MycoBank MB834253.  

*Etymology*: Named after the type species of the series, *Penicillium verhagenii*.  

*Type*: *Penicillium verhagenii* Houbraken, Stud. Mycol. 78: 443. 2014.  

*Diagnosis*: Series *Verhageniorum* belongs to subgen. *Aspergilloides*, sect. *Aspergilloides* and is sister to ser. *Saturniformia* (Fig. 18). *Morphology & physiology*: Colonies growing moderately; conidial colour *en masse* variable, in green shades with a blue element; conidiophores biverticillate, sometimes becoming divaricate by sympodial branching of the stipe at the apex, smooth or finely roughened; conidial shape variable, rough-walled; no growth on CYA incubated at 30 °C *Sexual morph* unknown; sclerotia not observed in culture. Series description based on Houbraken *et al.* (2014b).  

*Included species*: *Penicillium ranomafanaense*, *P*. *verhagenii*.  

*Extrolites*: *Penicillium ranomafanaense* produces andrastin A, asterric acid, fulvic acids, gregatins and geodin; *P*. *verhagenii* produces a quinone of unknown structure.  

*Notes on sect*. *Aspergilloides and included series*: Section *Aspergilloides* was introduced by Pitt (1980) to accommodate *Penicillium* species that predominantly produce monoverticillate conidiophores in which at least a portion of the stipes terminate in vesicular swellings. The phenotype-based infrageneric classification systems proposed in *Penicillium* are generally loosely corresponding with those based on phylogenetic inference using sequence data. Houbraken & Samson (2011) proposed a sectional classification system based on the phylogenetic analysis of a combined four-gene dataset and re-circumscribed section *Aspergilloides*. The majority of species belonging to this re-circumscribed section grow moderately or fast on agar media and are predominantly monoverticillate. The section was subsequently revised, and the 51 accepted species were distributed over 12 clades (Houbraken *et al.* 2014b). These clades are here treated as series. The relationship of *P*. *kiamaense* was unresolved and was therefore not accommodated in a clade; we introduced ser. *Kiamaensia* for this species. After 2014, three new species were described in sect. *Aspergilloides* (*P*. *fortuitum*, *P*. *improvisum*, and *P*. *jejuense*). The former two species form unique lineages in the section and are therefore accommodated in unique, separate series (*Fortuita*, *Improvisa*).

 The series classification is primarily based on the phylogenetic relationships of the species within the section, and this is often supported by morphology and physiology data. Growth rate, the ability to grow at 30 °C, conidiophore branching pattern and conidial shape and ornamentation were useful characters to differentiate the series of sect. *Aspergilloides*. The phylogenetic support was low or absent for series *Fortuita*, *Improvisa* and *Kiamaensia*. The phylogenetic distance of the former two series was sufficient to accommodate them in separate series. In addition, ser. *Fortuita* grows restrictedly, a feature shared with the distantly related ser. *Pinetorum*. Series *Kiamaensia* is a sister series of series *Spinulosa* and *Thomiorum*, though statistical support for this is weak. Series *Spinulosa* and *Thomiorum* are phylogenetically and phenotypically distinct. Series *Kiamaensia* is introduced in order to maintain monophyletic series. The relationship between the two species in ser. *Longicatenata* is moderately supported in the phylogram of Houbraken *et al.* (2014b). These species are phenotypically unrelated and this suggests that they might belong to two separate series. The discovery of more species related to this clade might show that they are actually more than one series; however, we prefer at this moment a conservative approach and maintain both species in one series.  

**Section *Charlesia*** Houbraken & Samson, Stud. Mycol. 70: 33. 2011. MycoBank MB563125.  

*Type*: *Penicillium charlesii* G. Sm., Trans. Brit. Mycol. Soc. 18: 90. 1933.  

*Description*: See Peterson *et al.* (2005) and Houbraken & Samson (2011) (morphology, phylogeny); a modern taxonomic study on this section is lacking.  

Series ***Costaricensia*** Houbraken & Frisvad, ***ser*. *nov*.** MycoBank MB834254.  

*Etymology*: Named after the type species of the series, *Penicillium costaricense*.  

*Type*: *Penicillium costaricense* Visagie *et al.*, Persoonia 36: 263. 2016.  

*Diagnosis*: Series *Costaricensia* belongs to subgen. *Aspergilloides*, sect. *Charlesia* and is sister to series *Fellutana*, *Indica* and *Phoenicea*. *Morphology & physiology*: Colonies restricted; conidial colour *en masse* turquoise to dull green; conidiophores monoverticillate, smooth; conidia subglobose, smooth-walled; growth at 37 °C absent. *Sexual morph* unknown; sclerotia not observed in culture. Series description based on Visagie *et al.* (2016b).  

*Included species*: *Penicillium costaricense*.  

*Extrolites*: Andrastin A & C (Visagie *et al.* 2016b).  

Series ***Fellutana*** Pitt, The Genus Penicillium: 263. 1980 [1979]. MycoBank MB832961.  

*Type*: *Penicillium fellutanum* Biourge, Cellule 33: 262. 1923.  

*Diagnosis*: Series *Fellutana* belongs to subgen. *Aspergilloides*, sect. *Charlesia* and is sister to ser. *Indica* (Fig. 18). *Morphology & physiology*: Colonies growing restricted; conidial colour *en masse* dark green; conidiophores monoverticillate or furcate, smooth; conidia globose or ellipsoidal, finely or distinctly rough-walled; growth at 37 °C absent. *Sexual morph* unknown; sclerotia not observed in culture. Series description based on Pitt (1980) and Peterson *et al.* (2005).  

*Included species*: *Penicillium charlesii*, *P*. *fellutanum*.  

*Extrolites*: *Penicillium charlesii* in ser. *Fellutana* produces carolic acids (Clutterbuck et al., 1934, Clutterbuck et al., 1935a, Clutterbuck et al., 1935b, Clutterbuck et al., 1935c), an uracil nucleoside (Maynard & Gander 1966) and exopolysaccharides (Haworth *et al.* 1935). *Penicillium fellutanum* has been reported to produce different secondary metabolites such as fellutamides (Shigemori *et al.* 1991), fellutanine A-E and isofellutanine B & C (Kozlovsky et al., 1997a, Kozlovsky et al., 1997b, Kozlovsky et al., 2000), cyclosporine (Anjum *et al.* 2012) and peniphenylanes (Zhang *et al.* 2016).  

Series ***Indica*** Houbraken & Frisvad, ***ser*. *nov*.** MycoBank MB834255.  

*Etymology*: Named after the type species of the series, *Penicillium indicum*.  

*Type*: *Penicillium indicum* D.K. Sandhu & R.S. Sandhu, Canad. J. Bot. 41: 1273. 1963.  

*Diagnosis*: Series *Indica* belongs to subgen. *Aspergilloides*, sect. *Charlesia* and is sister to ser. *Fellutana* (Fig. 18). *Morphology & physiology*: Colonies growing moderately fast or spreading; conidial colour *en masse* dull green or grey green; conidiophores predominantly monoverticillate, conspicuously vesiculate, smooth; conidia subglobose to ellipsoidal, smooth-walled; growth at 37 °C present (*P*. *chermesinum*, *P*. *indicum*) or absent (*P*. *lunae*). *Sexual morph* unknown; sclerotia produced in *P*. *indicum*, white to cream. Series description based on Pitt (1980) and Crous *et al.* (2019).  

*Included species*: *Penicillium chermesinum*, *P*. *cuddlyae*∗, *P*. *indicum*, *P*. *lunae*∗ [∗not included in Fig. 18; more info on their phylogenetic relationship, see Crous *et al.* (2019)].  

*Extrolites*: *Penicillium chermesinum* is reported to produce chermesins (Liu *et al.* 2016b), penicilliumolides (Darsih *et al.* 2015), PR-toxins (Darsih *et al.* 2015), chermesinones and terphenyllins (Huang *et al.* 2011) and costaclavin (Agurell 1964), and also to secrete the ribotoxins proteins (Hwu *et al.* 2001).  

Series ***Phoenicea*** Houbraken & Frisvad, ***ser*. *nov*.** MycoBank MB834256.  

*Etymology*: Named after the type species of the series, *Penicillium phoeniceum*.  

*Type*: *Penicillium phoeniceum* J.F.H. Beyma, Zentralbl. Bakteriol. Parasitenk., Abt. 2 88: 136. 1933.  

*Diagnosis*: Series *Phoenicea* belongs to subgen. *Aspergilloides*, sect. *Charlesia* and is sister to series *Fellutana* and *Indica* (Fig. 18). *Morphology & physiology*: Colonies growing restrictedly or moderately rapid; conidial colour *en masse* dull green or dull greyish blue; conidiophores monoverticillate, vesiculate, smooth; conidia globose, smooth-walled; growth at 37 °C present (*P*. *phoeniceum*) or absent (*P*. *coffeae*). *Sexual morph* unknown; sclerotia not observed in culture. Series description based on Pitt (1980) and Peterson *et al.* (2005).  

*Included species*: *Penicillium coffeae*, *P*. *phoeniceum*.  

*Extrolites*: *Penicillium phoenicum* has been reported to produce phoenicin (Friedheim, 1938, Posternak, 1938, Curtin et al., 1940, Posternak et al., 1943, Steiner et al., 1974).  

*Notes on series of sect*. *Charlesia*: Peterson *et al.* (2005) studied the phylogenetic relationship of *P*. *coffeae* within the genus *Penicillium*. They showed that this species is related to *P*. *charlesii*, *P*. *chermesinum*, *P*. *coffeae*, *P*. *fellutanum*, *P*. *indicum* and *P*. *phoeniceum*; all species currently classified in sect. *Charlesia*. The phenotypic similarity between *P*. *charlesii*, *P*. *fellutanum* (ser. *Fellutana*), *P*. *chermesinum*, *P*. *indicum* (ser. *Indica*), and *P*. *coffeae* and *P*. *phoeniceum* (ser. *Phoenicea*) was also indicated and these groups of species (here treated as series) could be distinguished using colony growth rates and conidiophore complexity. *Penicillium lunae* and *P*. *costaricense* were described after Peterson *et al.* (2005); the former species belongs to ser. *Indica* and the latter represents a single species series.  

**Section *Cinnamopurpurea*** Houbraken & Samson, Stud. Mycol. 70: 34. 2011. MycoBank MB563128.  

*Type*: *Penicillium cinnamopurpureum* Udagawa, J. Agric. Food Sci., Tokyo 5: 1. 1959.  

*Description*: See Houbraken and Samson, 2011, Peterson et al., 2015 (morphology, phylogeny).  

Series ***Cinnamopurpurea*** Houbraken & Frisvad, ***ser*. *nov*.** MycoBank MB834257.  

*Etymology*: Named after the type species of the series, *Penicillium cinnamopurpureum*.  

*Type*: *Penicillium cinnamopurpureum* Udagawa, J. Agric. Food Sci., Tokyo 5: 1. 1959.  

*Diagnosis*: *Phylogeny*: Series *Cinnamopurpurea* belongs to subgen. *Aspergilloides*, sect. *Cinnamopurpurea* and is a sister series of *Idahoensia* and *Nodula* (Fig. 18). *Morphology & physiology*: Colonies restricted; conidial colour *en masse* blue-green, grey-green or pale green; conidiophores monoverticillate; stipes smooth, short, often less than 50 μm in length; conidia globose to subglobose, sometimes (broadly) ellipsoidal, smooth; growth at 37 °C absent (*P*. *gravinicasei*, *P*. *parvulum*) or present (*P*. *cinnamopurpureum*). *Sexual morph* not observed in culture (*P*. *gravinicasei*, *P*. *parvulum*) or present (*P*. *cinnamopurpureum*), eupenicillium-type, homothallic, pinkish cinnamon to brown; ascospores ellipsoidal, with two close equatorial ridges, valves (finely) rough-walled, warted viewed by SEM; sclerotia not observed in culture. Series description based on Pitt, 1980, Stolk and Samson, 1983, Peterson and Horn, 2009 and Anelli *et al.* (2018).  

*Included species*: *Penicillium cinnamopurpureum*, *P*. *gravinicasei*, *P*. *parvulum*.  

*Extrolites*: *Penicillium cinnamopurpureum* and *P*. *parvulum* produce some red anthraquinones of unknown structure.  

Series ***Idahoensia*** Houbraken & Frisvad, ***ser*. *nov*.** MycoBank MB834258.  

*Etymology*: Named after the type species of the series, *Penicillium idahoense*.  

*Type*: *Penicillium idahoense* Paden, Mycopathol. Mycol. Appl. 43: 259. 1971.  

*Diagnosis*: *Phylogeny*: Series *Idahoensia* belongs to subgen. *Aspergilloides*, sect. *Cinnamopurpurea* and is sister to ser. *Nodula* (Fig. 18). *Morphology & physiology*: Colonies restricted; conidial colour *en masse* in shades of green, grey-green, blue-green or pale green; conidiophores monoverticillate, occasionally with an additional branch; stipes short, smooth, often vesiculate; conidia varying from globose to ellipsoidal, mostly smooth or finely roughened, sometimes conspicuously spinulose (*P*. *malacaense*); growth at 37 °C generally absent, sometimes present (*P*. *idahoense*, (Visagie *et al.* 2014a). *Sexual morph* generally not observed in culture, only present in *P*. *idahoense*, eupenicillium-type, homothallic, (dark) brown; ascospores ellipsoidal, with two close equatorial ridges, valves (finely) smooth-walled under light microscope, warted viewed by SEM; sclerotia absent or present (*P*. *fluviserpens*, *P*. *lemhiflumine*), brown. Series description based on Paden, 1971, Ramírez, 1982, Stolk and Samson, 1983, Visagie et al., 2014a and Peterson *et al.* (2015).  

*Included species*: *Penicillium colei*, *P*. *cvjetkovicii*, *P*. *ellipsoideosporum*, *P*. *fluviserpens*, *P*. *idahoense*, *P*. *infrapurpureum*, *P*. *lemhiflumine*, *P*. *malacaense*, *P*. *minnesotense*∗, *P*. *monsgalena*, *P*. *monsserratidens*, *P*. *salmoniflumine* [∗ not included in Fig. 18, for details on their phylogenetic relationship, see Crous *et al.* (2019)].  

*Extrolites*: Red anthraquinones possibly related to roseopurpurin (Peterson *et al.* 2015); *P*. *colei* and *P*. *monsserratidens* produce citreoviridin (Peterson *et al.* 2015).  

Series ***Jiangxiensia*** Houbraken & Frisvad, ***ser*. *nov*.** MycoBank MB834259.  

*Etymology*: Named after the type species of the series, *Penicillium jiangxiense*.  

*Type*: *Penicillium jiangxiense* H.Z. Kong & Z.Q. Liang, Mycosystema 22: 4. 2003.  

*Diagnosis*: *Phylogeny*: Series *Jiangxiensia* belongs to subgen. *Aspergilloides*, sect. *Cinnamopurpurea* and is sister to the other series of sect. *Cinnamopurpurea*. *Morphology & physiology*: Colonies growing slowly; sporulation poor, conidial colour *en masse* grey-green, blueish grey; conidiophores predominantly monoverticillate, occasionally with an additional branch, stipes smooth; conidia globose to subglobose or ellipsoidal, smooth; growth at 37 °C reported in *P*. *jiangxiense*. *Sexual morph* unknown; sclerotia absent (*P*. *jiangxiense*) or present (*P*. *pusillum*), brownish. Series description based on Smith, 1939, Pitt, 1980 and Kong & Liang (2003).  

*Included species*: *Penicillium jiangxiense*, *P*. *pusillum*.  

*Extrolites*: No chemotaxonomic data available for these species.  

Series ***Nodula*** Houbraken & Frisvad, ***ser*. *nov*.** MycoBank MB834260.  

*Etymology*: Named after the type species of the series, *Penicillium nodulum*.  

*Type*: *Penicillium nodulum* H.Z. Kong & Z.T. Qi, Mycosystema 1: 108. 1988.  

*Diagnosis*: *Phylogeny*: Series *Nodula* belongs to subgen. *Aspergilloides*, sect. *Cinnamopurpurea* and is phylogenetically sister to ser. *Idahoensia* (Fig. 18). *Morphology & physiology*: Colonies growing restricted; conidial colour *en masse* dull green to olive green (*P*. *nodulum*, *P*. *shennongjianum*) or uncoloured (*P*. *incoloratum*); conidiophores predominantly monoverticillate, occasionally branched; stipes smooth, short, less than 50 μm in length; conidia globose to subglobose (*P*. *incoloratum*, *P*. *shennongjianum*) or ellipsoidal (*P*. *nodulum*), smooth-walled; growth at 37 °C absent. *Sexual morph* unknown; sclerotia not produced in culture. These species are to date only reported from China. Series description based on Kong & Qi (1988) and Huang & Qi (1994).  

*Included species*: *Penicillium incoloratum*, *P*. *nodulum*, *P*. *shennongjianum*.  

*Extrolites*: *Penicillium nodulum* produces griseofulvin, but there are no chemotaxonomic data available for the other species in ser. *Nodula*.  

*Notes on series of sect*. *Cinnamopurpurea*: Peterson *et al.* (2015) studied the species within sect. *Cinnamopurpurea*. They noted that this group of species are morphologically quite similar, all producing subglobose to ellipsoidal smooth to finely roughened spores, monoverticillate to divaricate biverticillate smooth-walled conidiophores and quite slow-growing colonies, often with a brown reverse on some media (Peterson *et al.* 2015). It is difficult to find good characters to delimit series in this section and the current series classification is therefore based on phylogenetic data.  

**Section *Citrina*** Houbraken & Samson, Stud. Mycol. 70: 40. 2011. MycoBank MB563132.  

*Type*: *Penicillium citrinum* Thom, U.S.D.A. Bur. Animal Industr. Bull. 118: 61. 1910.  

*Description*: See Houbraken & Samson (2011) and Houbraken *et al.* (2011a) (morphology, phylogeny).  

Series ***Citrina*** Raper & Thom ex Pitt, The Genus Penicillium: 290. 1980 [1979]. MycoBank MB832965.

*Synonym*: *Penicillium* ser. *Implicata* Raper & Thom ex Pitt, The Genus Penicillium: 191. 1980 [1979].  

*Type*: *Penicillium citrinum* Thom, U.S.D.A. Bur. Animal Industr. Bull. 118: 61. 1910.  

*Diagnosis*: *Phylogeny*: Series *Citrina* belongs to subgen. *Aspergilloides*, sect. *Citrina*, and the phylogenetic relationship with other series is unknown (Fig. 18). *Morphology & physiology*: Colonies growing moderately or fast; conidial colour *en masse* variable, (blueish) grey-green, dull green or pure green; conidiophores biverticillate, smooth; conidia globose, subglobose or broadly ellipsoidal, smooth or finely roughened; growth at 37 °C variable (absent: *P*. *gorlenkoanum*, *P*. *steckii*, *P*. *tropicoides*, *P*. *tropicum*; variable: *P*. *hetheringtonii*, *P*. *sizovae*; present: *P*. *citrinum*). *Sexual morph* unknown, or present (*P*. *tropicoides*, *P*. *tropicum*), eupenicillium-type, orange-tan, becoming (brownish) grey; ascospores ellipsoidal, with two narrow closely appressed equatorial ridges, convex smooth or finely roughened; sclerotia absent. Series description based on Houbraken et al., 2010b, Houbraken et al., 2011a.  

*Included species*: *Penicillium citrinum*, *P*. *gorlenkoanum*, *P*. *hetheringtonii*, *P*. *sizovae*, *P*. *steckii*, *P*. *tropicoides*, *P*. *tropicum*.  

*Extrolites*: Quinolactacin is produced by 4/7 species in series *Citrina* (*P*. *citrinum*, *P*. *heteringtonii*, *P*. *sizovae*, *P*. *steckii*), citrinin is produced by 3/7 species (*P citrinum*, *P*. *gorlenkoanum*, *P*. *heteringtonii*), citriquinones by 3/7 species (*P*. *gorlenkoanum*, *P*. *citrinum*, *P*. *steckii*), citrinalin by 2/7 species (*P*. *hetheringtonii*, *P*. *tropicoides*), isochromantoxins by 2/7 species (*P*. *steckii*, *P*. *tropicoides*), tanzawaic acid by 2/7 species (*P*. *sizovae*, *P*. *steckii*), and chanoclasvin by 1/7 species (*P*. *gorlenkoanum*) (Houbraken et al., 2011a, El-Neketi et al., 2013, Lai et al., 2013, Ranji et al., 2013).  

Series ***Copticolarum*** Houbraken & Frisvad, ***ser*. *nov*.** MycoBank MB834261.  

*Etymology*: Named after the type species of the series, *Penicillium copticola*.  

*Type*: *Penicillium copticola* Houbraken *et al.*, Stud. Mycol. 70: 88. 2011.  

*Diagnosis*: *Phylogeny*: Series *Copticolarum* belongs to subgen. *Aspergilloides*, sect. *Citrina*, and is phylogenetically related to ser. *Sumatraensia*, though without statistical support (Fig. 18). *Morphology & physiology*: Colonies growing moderately; conidial colour *en masse* dull (grey-)green or pure green; conidiophores symmetrically biverticillate, smooth, finely or distinctly rough-walled; conidia broadly ellipsoidal, smooth; growth present at 30 °C (up to 23 mm), absent at 37 °C. *Sexual morph* unknown; sclerotia not observed in culture. Series description based on Houbraken *et al.* (2011a).  

*Included species*: *Penicillium copticola*, *P*. *dokdoense*, *P*. *terrigenum*.  

*Extrolites*: *Penicillium copticola* produces sporogen AO1 and related terpenes in addition to penicillimides / penicillithiophenols and chimeric products of those families of secondary metabolites (Bu et al., 2015, Daengrot et al., 2015).  

Series ***Euglauca*** Houbraken & Frisvad, ***ser*. *nov*.** MycoBank MB834262.  

*Etymology*: Named after the type species of the series, *Penicillium euglaucum*.  

*Type*: *Penicillium euglaucum* J.F.H. Beyma, Antonie van Leeuwenhoek 6: 269. 1940.  

*Diagnosis*: *Phylogeny*: Series *Euglauca* belongs to subgen. *Aspergilloides*, sect. *Citrina*; however, the phylogenetic relationship within this section is unresolved (Fig. 18). *Morphology & physiology*: Colonies growing restrict or moderately fast; conidial colour *en masse* grey-green; conidiophores predominant monoverticillate or biverticillate, smooth; conidia globose to subglobose, smooth or finely roughened; growth at 37 °C absent or present. *Sexual morph* eupenicillium-type, brown or brownish grey; ascospores ellipsoidal, with two appressed distinct ridges, convex slightly roughened with warts and small ridges (*P*. *anatolicum*) or reticulate (*P*. *argentinense*, *P*. *euglaucum*). Series description based on Houbraken *et al.* (2011a).  

*Included species*: *Penicillium anatolicum*, *P*. *argentinense*, *P*. *euglaucum*, *P*. *vascosobrinhous* (recently described species, not included in Fig. 18).  

*Extrolites*: Curvularin and dehydrocurvularin are produced by *P*. *anatolicum* and *P*. *argentinense*. Furthermore, *P*. *anatolicum* produces anthraquinones, bisanthrons and sorbicillins, and *P*. *euglaucum* produces terrain (Houbraken *et al.* 2011a).  

Series ***Gallaica*** Houbraken & Frisvad, ***ser*. *nov*.** MycoBank MB834263.  

*Etymology*: Named after the type species of the series, *Penicillium gallaicum*.  

*Type*: *Penicillium gallaicum* Ramírez *et al.*, Mycopathol. 72: 30. 1980.  

*Diagnosis*: *Phylogeny*: Series *Gallaica* belongs to subgen. *Aspergilloides*, sect. *Citrina* and is phylogenetically basal to other series of sect. *Citrina* (Fig. 18). *Morphology & physiology*: Colonies growing moderately fast; conidial colour *en masse* dull or pale grey-green; conidiophores monoverticillate, occasionally with additional branch, smooth, short; conidia globose or subglobose, smooth; growth at 37 °C absent or very restricted (5 mm). *Sexual morph* unknown; sclerotia produced by *P*. *gallaicum*, orange-brown. Series description based on Houbraken *et al.* (2011a).  

*Included species*: *Penicillium gallaicum*.  

*Extrolites*: Citreoviridin, and several uncharacterised compounds unique for this series in sect. *Citrina* (“KOKSO”, “3-S”, “VYL”) (Houbraken *et al.* 2011a).  

Series ***Paxillorum*** Houbraken & Frisvad, ***ser*. *nov*.** MycoBank MB834264.  

*Etymology*: Named after the type species of the series, *Penicillium paxilli*.  

*Type*: *Penicillium paxilli* Bainier, Bull. Soc. Mycol. France 23: 95. 1907.  

*Diagnosis*: *Phylogeny*: Series *Paxillorum* belongs to subgen. *Aspergilloides*, sect. *Citrina*, and is phylogenetically related to ser. *Sheariorum*, though without statistical support (Fig. 18). *Morphology & physiology*: Colonies growing moderately to fast; conidial colour *en masse* dull (blue-)green; conidiophores predominantly symmetrically biverticillate, rough-walled; conidia subglobose, smooth; good growth at 30 °C, no growth at 37 °C. *Sexual morph* unknown; sclerotia not observed in culture. Series description based on Houbraken *et al.* (2011a).  

*Included species*: *Penicillium paxilli*.  

*Extrolites*: *Penicillium paxilli* produces paxilline (Cole *et al.* 1974, Springer et al., 1975, Fan et al., 2018a) and pyrenocins (Fan et al., 2018a, Fan et al., 2018b, under the name *P*. *camemberti*).  

Series ***Roseopurpurea*** Houbraken & Frisvad, ***ser*. *nov*.** MycoBank MB834265.  

*Etymology*: Named after the type species of the series, *Penicillium roseopurpureum*.  

*Type*: *Penicillium roseopurpureum* Dierckx, Ann. Soc. Sci. Bruxelles 25: 86. 1901.  

*Diagnosis*: *Phylogeny*: Series *Roseopurpurea* belongs to subgen. *Aspergilloides*, sect. *Citrina*; the series is well-supported, but phylogenetic relationship with other series within sect. *Citrina* is undetermined (Fig. 18). *Morphology & physiology*: Colonies restricted; conidial colour *en masse* pale grey-green; conidiophores monoverticillate or furcate, smooth, generally short; conidia globose to subglobose, smooth or finely roughened; growth at 30 °C absent or restricted (13 mm), at 37 °C absent. *Sexual morph* unknown; sclerotia not observed in culture. Series description based on Houbraken *et al.* (2011a).  

*Included species*: *Penicillium roseopurpureum*, *P*. *sanguifluum*.  

*Extrolites*: The species in ser. *Roseopurpurea* produce the anthraquionone carviolin (= roseopurpurin) and related anthraquinones (Hind, 1940a, Hind, 1940b, Posternak, 1940). In addition, *P*. *sanguifluum* produces aculeatusquinones, citreofuran, citridones, curvularins, neobrugarones, penilactone, roseopurpurins A-I (not related to roseopurpurin), sulfimarin, and trichodimerol (Aly et al., 2011, Shang et al., 2016).  

Series ***Sheariorum*** Houbraken & Frisvad, ***ser*. *nov*.** MycoBank MB834266.  

*Etymology*: Named after the type species of the series, *Penicillium shearii*.  

*Type*: *Penicillium shearii* Stolk & D.B. Scott, Persoonia 4: 396. 1967.  

*Diagnosis*: *Phylogeny*: Series *Sheariorum* belongs to subgen. *Aspergilloides*, sect. *Citrina*, and is phylogenetically related to ser. *Paxillorum*, though without statistical support (Fig. 18). *Morphology & physiology*: Colonies growing moderately fast; conidial colour *en masse* grey-green; conidiophores predominantly biverticillate, smooth; conidia subglobose to broadly ellipsoidal, smooth; growth at 37 °C. *Sexual morph* eupenicillium-type, dark grey; ascospores ellipsoidal with two equatorial ridges, convex roughened, warted. Series description based on Houbraken *et al.* (2011a).  

*Included species*: *Penicillium shearii*.  

*Extrolites*: Paxillin, paspalinine, shearinins (Belofsky et al., 1995, Houbraken et al., 2011a, Ariantari et al., 2019). The production of paxillin indicates a relationship to *P*. *paxilli* (ser. *Paxillorum*) and *P*. *thiersii* (ser. *Thiersiorum*).  

Series ***Sumatraensia*** Houbraken & Frisvad, ***ser*. *nov*.** MycoBank MB834267.  

*Etymology*: Named after the type species of the series, *Penicillium sumatraense*.  

*Type*: *Penicillium sumatraense* Szilvinyi, Archiv. Hydrobiol.14 Suppl. 6: 535. 1936.  

*Diagnosis*: *Phylogeny*: Series *Sumatraensia* belongs to subgen. *Aspergilloides*, sect. *Citrina* and is phylogenetically related to ser. *Copticolarum*, though without statistical support (Fig. 18). *Morphology & physiology*: Colonies growing moderately or fast; conidial colour *en masse* blue-green, dull green or dark green; conidiophores predominantly biverticillate, smooth; conidia subglobose to broadly ellipsoidal, finely roughened; growth at 37 °C absent. *Sexual morph* unknown; sclerotia not observed in culture. Series description based on Houbraken *et al.* (2011a).  

*Included species*: *Penicillium sumatraense*.  

*Extrolites*: *Penicillium sumatraense* produces curvularins such as curvularin, dehydrocurvularin, sumalactone A-D, sumalarins and citridones E-G (Vesonder et al., 1976, Malmstrom et al., 2000, Meng et al., 2013, de Castro et al., 2016, Ha et al., 2017, Wu et al., 2017, Xu et al., 2019b). This species has been reported to produce a blue mould rot of *Vitus vinifera* and *Sparassis crispa* (Mahdian and Zafari, 2016, Liu et al., 2018b).  

Series ***Westlingiorum*** Houbraken & Frisvad, ***ser*. *nov*.** MycoBank MB834268.  

*Etymology*: Named after the type species of the series, *Penicillium westlingii*.  

*Type*: *Penicillium westlingii* K.W. Zaleski, Bull. Int. Acad. Polon. Sci., Sér. B., Sci. Nat. 1927: 473. 1927.  

*Diagnosis*: *Phylogeny*: Series *Westlingiorum* belongs to subgen. *Aspergilloides*, sect. *Citrina*; the phylogenetic position of this series in sect. *Citrina* remains unknown (Fig. 18). *Morphology & physiology*: Colonies growing variable, restrictly (*e.g*., *P*. *wellingtonense*, *P*. *nothofagi*), moderately or rapidly (*P*. *decaturense*, *P*. *quebecense*); conidial colour *en masse* blue-green to greyish green; conidiophores predominantly biverticillate, generally smooth, except in certain strains of *P*. *manginii* and *P*. *atrofulvum*; conidia globose, subglobose or (broadly) ellipsoidal; growth at 37 °C absent. *Sexual morph* unknown; sclerotia absent or present, mostly in shades of orange-brown (*P*. *aurantiacobrunneum*, *P*. *cairnsense*, *P*. *manginii*, *P*. *miczynskii*, *P*. *pasqualense*, *P*. *quebecense*), sometimes black (*P*. *atrofulvum*). Series description based on Houbraken *et al.* (2011a).  

*Included species*: *Penicillium atrofulvum*, *P*. *aurantiacobrunneum*, *P*. *cairnsense*, *P*. *christenseniae*, *P*. *chrzaszczii*, *P*. *cosmopolitanum*, *P*. *decaturense*, *P*. *godlewskii*, *P*. *manginii*, *P*. *miczynskii*, *P*. *neomiczynskii*, *P*. *nothofagi*, *P*. *pancosmium*, *P*. *pasqualense*, *P*. *quebecense*, *P*. *raphiae*, *P*. *sucrivorum*, *P*. *ubiquetum*, *P*. *vancouverense*, *P*. *waksmanii*, *P*. *wellingtonense*, *P*. *westlingii*.  

*Extrolites*: Citrinin is produced by 13/21 species, terrein is produced by 9/21 species, citreoviridin is produced by 8/21 species, decaturins are produced by 5/21 species, okaramins are produced by 3/21 species, phoenicin is produced by 3/21 species, quinolactacin is produced by species, quinolactacin is produced by 2/21 species, benzomalvins are produced by 2/21 species, territrems are produced by 2/21 species, perinadins are produced by 2/21 species, daldinins are produced by 2/21 species, citrinalins are produced by 1/21 species, curvularins are produced by 1/21 species, cyclopiazonic acid is produced by 1/21 species, meleagrin is produced by 1/21 species, and pyrenocin is produced by 1/21 species (Houbraken *et al.* 2011a).  

*Notes on series of sect*. *Citrina*: The phylogenetic relationships within sect. *Citrina* were studied in detail with partial β-tubulin and calmodulin sequences (Houbraken *et al.* 2011a). Nine lineages were recognised and are treated here as series. The deeper nodes in sect. *Citrina* are mostly without any statistical support and therefore the phylogenetic relationship between the various series remains uncertain (Fig. 18). Six sect. *Citrina* species form cleistothecia: all taxa of series *Euglauca* (three species) and *Sheariorum* (one species), and two species of ser. *Citrina* (*P*. *tropicum* and *P*. *tropicoides*). Sclerotium production is present in ser. *Gallaica* and seven species of ser. *Westlingiorum*. The growth rate at 30 and 37 °C is also informative at series level. Series *Westlingiorum* species generally have maximum growth temperatures at or below 30 °C (with exception of *P*. *pasqualense*, *P*. *quebecense* and *P*. *decaturense*; growth at 30 °C or 33 °C). In contrast, ser. *Citrina* species have higher optimum and maximum growth temperatures. With exception of *P*. *tropicoides*, all species were able to grow at 33 °C. Series *Sheariorum* species grow well at 37 °C. The majority of sect. *Citrina* taxa produce symmetrically branched biverticillate conidiophores. Exceptions are all species classified in series *Roseopurpurea* (*P*. *roseopurpureum*, *P*. *sanguifluum*) and *Gallaica* (*P*. *gallaicum*) that predominantly produce monoverticillate conidiophores. Conidiophore stipes are generally smooth, with exception of ser. *Sheariorum* species and certain isolates of *P*. *manginii* and *P*. *atrofulvum* of ser. *Westlingiorum* (Houbraken *et al.* 2011a).  

**Section *Crypta*** Houbraken & Frisvad, ***sect*. *nov*.** MycoBank MB834269.  

*Etymology*: Named after the type species of the series, *Penicillium cryptum*.  

*Type*: *Penicillium cryptum* Goch., Mycotaxon 26: 349. 1986.  

*Diagnosis*: *Phylogeny*: Section *Crypta* belongs to subgen. *Aspergilloides* and is phylogenetically related to sect. *Torulomyces*. *Morphology & physiology*: Colonies growing very restrictedly; conidial colour *en masse* pale yellow-green; conidiophores biverticillate, occasionally terverticillate; stipes short (8–50(–90) μm), smooth; conidia globose to subglobose, smooth to finely roughened. *Sexual morph* eupenicillium-type, pale beige; ascospores ellipsoidal, with two well separated ridges, convex smooth-walled. Series description based on Gochenaur & Cochrane (1986).  

*Included species*: *Penicillium cryptum*.  

*Extrolites*: No chemotaxonomic data available.  

*Notes*: Houbraken & Samson (2011) accommodated *P*. *cryptum* in sect. *Torulomyces*. More recently, Visagie *et al.* (2016a) showed that this species is actually distantly related to the other sect. *Torulomyces* taxa and suggested that it might not belong to this section. This observation was confirmed by phenotypic characters: *P*. *cryptum* predominately produces biverticillate conidiophores, while sect. *Torulomyces* members produce solitary phialides. Furthermore, *P*. *cryptum* produces smooth-walled conidia, in contrast to the ornamented conidia in sect. *Torulomyces*. *Penicillium cryptum* is the sole species in sect. *Crypta* and a subdivision of the section can therefore not be made. Series *Crypta* is only informally introduced here.  

**Section *Eremophila*** Houbraken & Frisvad, ***sect*. *nov*.** MycoBank MB834270.  

*Etymology*: Named after the type species of the series, *Penicillium eremophilum*.  

*Type*: *Penicillium eremophilum* (A.D. Hocking & Pitt) Houbraken *et al.*, Stud. Mycol. 86: 47. 2017.  

*Diagnosis*: *Phylogeny*: Section *Eremophila* belongs to subgen. *Aspergilloides* and is most closely related to sect. *Charlesia*. *Morphology & physiology*: Xerophilic, no growth on high water activity media; asexual morph not produced; growth at 37 °C absent. *Sexual morph* monascus-like, brownish orange; ascospores subglobose to ellipsoidal, formed in pairs, smooth-walled. Series description based on Hocking and Pitt, 1988, Leong et al., 2011 and Barbosa *et al.* (2017).  

*Included species*: *Penicillium eremophilum*.  

*Extrolites*: This species has not been examined for extrolites.  

*Notes*: *Penicillium eremophilum* is the sole species in sect. *Eremophila* and is unlike any other *Penicillium* species, an obligate xerophile. The asexual morph of this species is not known. In addition, the formation of two-spored asci is also not shared with other *Penicillium* species and this feature, together with its xerophily, is shared with the phylogenetically distant species *Xeromyces bisporus* (Barbosa *et al.* 2017). Earlier studies (Park et al., 2004, Pettersson et al., 2011, Houbraken et al., 2014a, Barbosa et al., 2017) repeatedly position *P*. *eremophilum* (as *Monascus eremophilus*) in *Penicillium*, supporting the results of our phylogenetic analysis. *Penicillium eremophilum* was confidently positioned as a sister lineage of a clade containing *P*. *charlesii* and *P*. *fellutanum* (both sect. *Charlesia*) (Houbraken *et al.* 2014a), and this result is confirmed in our study (Fig. 2, Fig. 18).  

**Section *Exilicaulis*** Pitt, The Genus Penicillium: 205. 1980 [1979]. MycoBank MB832954.

*Synonym*: *Eupenicillium* sect. *Lapidosa* (Pitt) Stolk & Samson, Stud. Mycol. 23: 55. 1983.  

*Type*: *Penicillium restrictum* J.C. Gilman & E.V. Abbott, Iowa State Coll. J. Sci. 1: 297. 1927.  

*Description*: See Houbraken and Samson, 2011, Visagie et al., 2016c (morphology, phylogeny).  

Series ***Alutacea*** (Pitt) Houbraken & Frisvad ***comb*. *nov*.** MycoBank MB834271.

*Basionym*: *Eupenicillium* ser. *Alutacea* Pitt, The Genus Penicillium: 54. 1980 [1979].  

*Type*: *Eupenicillium alutaceum* D.B. Scott, Mycopathol. Mycol. Appl. 36: 17. 1968.  

*Diagnosis*: *Phylogeny*: Series *Alutacea* belongs to subgen. *Aspergilloides*, sect. *Exilicaulis* and is phylogenetically related to series *Citreonigra*, *Corylophila*, *Lapidosa* and *Restricta*; the most closely related series could not be determined (Fig. 18). *Morphology & physiology*: Colonies restricted or growing moderately fast; conidial colour *en masse* grey-green or dull green; conidiophores monoverticillate, short and generally 20–60 μm; conidia (broadly) ellipsoidal, smooth-walled; growth at 37 °C generally present. *Sexual morph* not produced in culture (*P*. *decumbens*) or present (*P*. *alutaceum*), eupenicillium-type, pale brown; ascospores ellipsoidal, yellow, with two, sometimes four, longitudinal flanges, convex smooth to finely roughened. Series description based on Pitt (1980).  

*Included species*: *Penicillium alutaceum*, *Penicillium decumbens*.  

*Extrolites*: *Penicillium alutaceum* produces andrastin A and fulvic acid. *Penicillium decumbens* produces the volatiles thujopsene, nerolidol (both terpenes), 1-octen-3-ol, 3-octanone and phenylethylalcohol (Halim et al., 1975, Polizzi et al., 2011), and decumbenones and calbistrins (Fujii *et al.* 2002, results reported here). *Penicillium striatisporum* also produces calbistrins (Brill et al., 1993, Jackson et al., 1993) (both as *P*. *restrictum,* Stewart *et al.* 2005) and citromycins and citromycetins (Capon *et al.* 2007). Other extrolites reported include cyclocitrinols (Lin *et al.* 2014), cyclopenicillone (Lin *et al.* 2011) trichopyrone, sorbicillin, penicillone A and 3,11-dihydroxy-6,8-dimethyldodecanoic acid (Lin *et al.* 2018), and diisooctylphthalate (Amer *et al.* 2019). We also detected andrastin A and C in *P*. *decumbens*. Decumbin, reported from *P*. *decumbens* was shown to be brefeldin A (and produced by *Penicillium brefeldianum*) (Singleton et al., 1958, Betina, 1992). Peniproline A and chrysotriazoles and related compounds could not be detected in *P*. *decumbens* by us (see Wang *et al.* 2017b, the ITS sequence indicates the producer is *Penicillium limosum*), but such compounds have been found in *Penicillium paneum* (Li *et al.* 2011). In ser. *Alutacea* only andrastin A seems to be in common between the two species. A full genome sequenced isolate, with 28 predicted gene clusters for secondary metabolites was first identified as *P*. *decumbens*, but was later shown to be *P*. *oxalicum* (Liu et al., 2013, Houbraken et al., 2014a). This was confirmed by the fact that the isolate of *P*. *oxalicum* produced roquefortine C and meleagrin as other isolates of *P*. *oxalicum* (Steyn & Vleggaar 1983).  

Series ***Citreonigra*** Pitt, The Genus Penicillium: 218. 1980 [1979]. MycoBank MB832956.  

*Type*: *Penicillium citreonigrum* Dierckx, Ann. Soc. Sci. Bruxelles 25: 86. 1901.  

*Diagnosis*: *Phylogeny*: Series *Citreonigra* belongs to subgen. *Aspergilloides*, sect. *Exilicaulis* and is phylogenetically related to series *Alutacea*, *Corylophila*, *Lapidosa* and *Restricta*; the most closely related series could not be determined (Fig. 18). *Morphology & physiology*: Colonies growing restrictedly to moderately rapid; conidial colour *en masse* grey-green, dull green or dark green; conidiophores monoverticillate, occasionally biverticillate; stipe smooth-walled; conidia smooth, globose; growth at 37 °C absent (*P*. *cinerascens*, *P*. *citreonigrum*, *P*. *fundyense*) or restricted (*P*. *citreosulfuratum*). *Sexual morph* unknown. Series description based on Visagie *et al.* (2016c).  

*Included species*: *Penicillium cinerascens*, *P*. *citreonigrum*, *P*. *citreosulfuratum*, *P*. *fundyense*.  

*Extrolites*: Three species in the series *Citreonigra* produce citreoviridin, *P*. *cinerascens*, *P*. *citreonigrum* (Sakabe *et al.* 1964, as *P*. *citreoviride*) and *P*. *citreosulphuratum*. *Penicillium citreonigrum* also produces citreoindol (closely related to haemindole) (Matsunaga et al., 1991, Song et al., 2016) and dipicolinic acid (Kalle & Khandekar 1983). *Penicillium cinerascens* was also reported to produce gliotoxin and dehydrocarolic acid (Bracken & Raistrick 1947). An atlantinone, sclerotiorin (several sclerotiorins) and pencolide were reported from *P*. *citreonigrum*; however, this fungus is correctly identified as *P*. *hirayamae* (Wang *et al.* 2010). These metabolites are typical for members of *Penicillium* series *Sclerotiorum*. A strain identified as *P*. *citreonigrum* (XN 10) was reported to produce sclerotiamine, three chromones, several eremophilans such as PR-amide, citreopenin and 3-epi-isopetasol, and mycophenolic acid (Yuan et al., 2014, Yuan et al., 2015, Yuan et al., 2017). We have not been able to detect those extrolites in *P*. *citreonigrum*. The isolate SP-6 of *P*. *citreonigrum* was reported to produce (-)-dichlorodiaportal in addition to an unusual diketopiperazine and N-(3-acetamidopropyl)-3-hydroxy-4-methoxybenzamide and a related compound (Huang *et al.* 2018). We have not been able to confirm production of those extrolites in *P*. *citreonigrum* either.  

Series ***Corylophila*** Houbraken & Frisvad, ***ser*. *nov*.** MycoBank MB834272.  

*Type*: *Penicillium corylophilum* Dierckx, Ann. Soc. Sci. Bruxelles 25: 86. 1901.  

*Etymology*: Named after the type species of the series, *Penicillium corylophilum*.  

*Diagnosis*: *Phylogeny*: Series *Corylophila* belongs to subgen. *Aspergilloides*, sect. *Exilicaulis* and is phylogenetically related to ser. *Lapidosa* (Fig. 18). *Morphology & physiology*: Colonies generally spreading, sometimes growing moderately rapid or restrictedly (*P*. *cravenianum*, *P*. *pagulum*); conidial colour *en masse* in shades of green (greyish, dull or dark green); conidiophores predominantly biverticillate, sometimes mono- or terverticillate; stipe smooth or rough-walled; conidia globose to broadly ellipsoidal, smooth, finely or distinctly roughened; growth at 37 °C absent or present and often restricted (*P*. *momoii*, *P*. *pagulum*, *P*. *repensicola*, *P*. *subturcoseum*). *Sexual morph* unknown. Series description based on Visagie *et al.* (2016c).  

*Included species*: *Penicillium atrolazulinum*, *P*. *consobrinum*, *P*. *corylophilum*, *P*. *cravenianum*, *P*. *fagi*, *P*. *momoii*, *P*. *pagulum*, *P*. *punicae*, *P*. *repensicola*, *P*. *rubefaciens*, *P*. *subturcoseum*.  

*Extrolites*: *Penicillium corylophilum* produces andrastins and the related citreohybridinol, citreoisocoumarin and the related (+)-orthosporin, phomenone, isochromans and α-pyrones (Lai *et al.* 1991 (reported under the synonym *P*. *citreovirens*), Malmstrom et al., 2000, McMullin et al., 2014a, McMullin et al., 2014b, Yadav et al., 2014). Production of decarestrictins and epoxyagroclavine-I has also been reported from *P*. *corylophilum* (Grabley *et al.* 1992), but the identity of the strain has not been confirmed. Furan-2-carboxylic acid has been reported from *P*. *corylophilum* (Turner & Aldridge 1983). *Penicillium fagi* produces andrastin A, citrinalin, mycophenolic acid, a paraherquamide and pulvilloric acid. *Penicillium rubefaciens* produces a curvulic acid related extrolite. The other species have not yet been examined for extrolites, but there seem to be few extrolites in common between the species in ser. *Corylophila*.  

Series ***Erubescentia*** (Pitt) Houbraken & Frisvad, ***comb*. *nov*.** MycoBank MB834273.

*Basionym*: *Eupenicillium* ser. *Erubescentia* Pitt, The Genus Penicillium: 70. 1980 [1979].  

*Type*: *Eupenicillium erubescens* D.B. Scott, Mycopathol. Mycol. Appl. 36: 14. 1968. MycoBank MB330727.  

*Diagnosis*: *Phylogeny*: Series *Erubescentia* belongs to subgen. *Aspergilloides* sect. *Exilicaulis* and is phylogenetically sister to all other series of this section (Fig. 18). *Morphology & physiology*: Colonies generally restricted, sometimes moderately fast; conidial colour *en masse* variable, olive grey, blue grey, grey-green or dull green; conidiophores monoverticillate; stipes short, smooth; conidial shape variable, globose, subglobose, ovoid, or (broadly) ellipsoidal, smooth or rough-walled; growth at 37 °C generally present, sometimes absent (*e.g*., *P*. *hermansii*, *P*. *nepalense*). *Sexual morph* unknown (12 species) or present (six species) (see list of accepted species), eupenicillium-type, brown, orange-brown or pinkish brown; ascospores (broadly) ellipsoidal, with one or two pairs of longitudinal flanges, convex smooth, roughened or spinose. Series description based on Pitt, 1980, Peterson et al., 1999, Visagie et al., 2016c and Houbraken *et al.* (2019).  

*Included species*: *Penicillium canis*, *P*. *catenatum*, *P*. *dimorphosporum*, *P*. *dravuni*, *P*. *erubescens*, *P*. *guttulosum*, *P*. *hermansii*, *P*. *labradorum*∗, *P*. *laeve*, *P*. *menonorum*, *P*. *nepalense*, *P*. *ovatum*, *P*. *parvofructum*, *P*. *parvum*, *P*. *pimiteouiense*, *P*. *rubidurum*, *P*. *striatisporum*, *P*. *vinaceum* [∗ not included in Fig. 18; see Rothacker *et al.* (2020)].  

*Extrolites*: *Penicillium striatisporum* produces calbistrins, striatisporins, hexylitaconic acids and striatisporolide A (Brill et al., 1993, Jackson et al., 1993, Stewart et al., 2005). *Penicillium erubescens* has been reported to produce anhydrofulvic acid and the related myxotrichin B and citromycins, GKK1032B, penialidin D, pyranochromones, secalonic acid A and SPF-3059-30 and related chromones including erubescensoic acid (Kumla et al., 2018, Kumla et al., 2019). *Penicillium parvum* has been reported to produce mycophenolic acid and many derivatives and precursors (including euparvic acid and euparvilactone), citromycetin, euparvione, 7-hydroxy-2,5-dimethyl-4H-chromen-4-one and azadirachtin (Habib et al., 2008, Kusari et al., 2012, León et al., 2013) and *P*. *dravuni* produces dictyosphaeric acid A & B and carviolin = roseopurpurin (Bugni et al., 2004, Burns et al., 2010). *Penicillium vinaceum* has been reported to produce vinaxanthones (Aoki et al., 1991, Řezanka et al., 2008) and (-)-(1R,4R)-1,4-(2,3)indolmethane-1-methyl-2,4-dihydro-1H-pyrazino-[2,1-b]-quinazoline.3,6-dione (Zheng *et al.* 2012), and penicillivinacine, citreoisocoumarin, indol-3-carbaldehyde, α-cyclopiazonic acid, terretrione A, brevianamide F and its diastereomer cyclo-D-Trp-L-Pro (Asiri *et al.* 2015). The latter seven metabolites are apparently produced by *Penicillium rubens* (or closely related species) according to the reported ITS sequence. Other metabolites reported from *P*. *vinaceum* was based on the strain DQ25 (Wei *et al.* 2009), which, based on the reported ITS sequence, is also a *P*. *rubens*. *Penicillium parvum* and *P*. *erubescens* share secondary metabolites from the citromycetin biosynthetic family, but else species in ser. *Erubescentia* shares coloured polyketide metabolites, that are somewhat different.  

Series ***Lapidosa*** (Pitt) Houbraken & Frisvad, ***comb*. *nov*.** MycoBank MB834517.

*Basionym*: *Eupenicillium* ser. *Lapidosa* Pitt, The Genus Penicillium: 129. 1980 [1979].  

*Type*: *Eupenicillium lapidosum* D.B. Scott & Stolk, Antonie van Leeuwenhoek 33: 298. 1967. MycoBank MB330733.  

*Diagnosis*: *Phylogeny*: Series *Lapidosa* belongs to subgen. *Aspergilloides*, sect. *Exilicaulis* and is phylogenetically related to ser. *Corylophila* (Fig. 18). *Morphology & physiology*: Colonies growing moderate, sometimes fast; conidial colour *en masse* variable, in shades of green, dark green, greyish green or dull green; conidiophores biverticillate, sometimes monoverticillate; conidia variable shaped (globose to ellipsoidal), generally smooth-walled; growth at 37 °C variable. *Sexual morph* generally not observed in culture, present in *P*. *lapidosum* and *P*. *terrenum*, eupenicillium-type, orange-brown or light brownish yellow; ascospores hyaline to pale yellow, ellipsoidal, with two prominent longitudinal flanges, convex smooth or spinose. Series description based on Pitt (1980) and Visagie *et al.* (2016c).  

*Included species*: *Penicillium aotearoae*, *P*. *atrosanguineum*, *P*. *burgense*, *P*. *diabolicalicense*, *P*. *hemitrachum*, *P*. *lapidosum*, *P*. *maclennaniae*, *P*. *melinii*, *P*. *namyslowskii*, *P*. *raciborskii*, *P*. *smithii*, *P*. *terrenum*, *P*. *velutinum*, *P*. *xanthomelinii*.  

*Extrolites*: *Penicillium smithii* produces a metabolite related to phoenicin, in addition to citreoviridin, citreoisocoumarins and paxillin, and *P*. *atrosanguineum* and *P*. *maclennaniae* share quinone secondary metabolites (phoenicin and spinulosin, respectively) (Christensen *et al.* 1999). Furthermore, *P*. *atrosanguineum* produces some tryptoquivalines. *Penicillium lapidosum* was reported to produce patulin (Myrchink 1967) and lapidosin (Turner 1978). We detected pulvilloric acid in *P*. *raciborskii* and *P*. *melinii*. In addition, *P*. *melinii* produces andrastin A and daldinin D. *Penicillium namyslowskii* produces a polyene not yet structure elucidated and haenamindole. Most species in ser. *Lapidosa* have not yet been studied in any detail chemically.  

Series ***Restricta*** Raper & Thom ex Pitt, The Genus Penicillium: 205. 1980 [1979]. MycoBank MB832955.  

*Type*: *Penicillium restrictum* J.C. Gilman & E.V. Abbott, Iowa State Coll. J. Sci. 1: 297. 1927.  

*Diagnosis*: *Phylogeny*: Series *Restricta* belongs to subgen. *Aspergilloides*, sect. *Exilicaulis* and is phylogenetically related to series *Alutacea*, *Corylophila*, *Lapidosa* and *Citreonigra*; the most closely related series could not be determined (Fig. 18). *Morphology & physiology*: Colonies growing restrictedly or moderately rapid; conidial colour *en masse* grey-green, blue-green or dull green; conidiophores generally monoverticillate; stipe smooth or finely roughened, generally short; conidia globose, subglobose, or (broadly) ellipsoidal, smooth or roughened; growth at 37 °C generally present. *Sexual morph* generally not observed in culture (15 species), sometimes present (*P*. *katangense*, *P*. *meridianum*, *P*. *philippinense*), eupenicillium-type, greyish orange or light brownish orange; ascospores ellipsoidal, yellow, with two small appressed longitudinal flanges, sometimes with a second smaller pair, convex smooth-walled or finely roughened. Series description based on Pitt, 1980, Stolk and Samson, 1983 and Visagie *et al.* (2016c).  

*Included species*: *Penicillium arabicum*, *P*. *chalabudae*, *P*. *cinereoatrum*, *P*. *heteromorphum*, *P*. *katangense*, *P*. *kurssanovii*, *P*. *meridianum*, *P*. *philippinense*, *P*. *restrictum*.  

*Extrolites*: *Penicillium restrictum* has been reported to produce pestalotin, hydroxypestalotin, LLP880γ and 5,6-dihydro-4-methoxy-6-(1-oxopentyl)-2H-pyran-2-one (Geiger *et al.* 2013), emodin, emodic acid and other anthraquinones (Figueroa *et al.* 2014), andrastin A and phomenone (Antipova *et al.* 2018b), gliotoxin and dehydrocarolic acid (Sankhala 1968), 2,3-dihydro-3,6-dihydroxy-2-methyl-4-pyrone, curvularin and dehydrocurvularin (as *P*. *gilmanii*, Raistrick and Rice, 1971, Rice and Chen, 1984), and restricticins (Hensens et al., 1991, Schwartz et al., 1991). Production of patulin and penicillic acid by *P*. *restrictum* (Martín *et al.* 2004) could not be confirmed.  

*Notes*: Visagie *et al.* (2016c) studied the phylogenetic relationship between sect. *Exilicaulis* species and recognised six main lineages: the *P*. *citreonigrum*-, *P*. *corylophilum*-, *P*. *decumbens*-, *P*. *melinii*-, *P*. *parvum*- and *P*. *restrictum*-clades. These clades are treated here as series: *Citreonigra, Corylophila*, *Alutacea*, *Lapidosa*, *Erubescentia* and *Restricta*, respectively. These series are primary based on phylogenetic data. Visagie *et al.* (2016c) indicated that the branching pattern is informative. The phylogenetically related series *Corylophila* and *Lapidosa* contain species with biverticillate conidiophores, and monoverticillate species were present in the four other series (*Alutacea*, *Citreonigra*, *Erubescentia* and *Restricta*). Species belonging to sect. *Exilicaulis* have different growth rates and species belonging to series *Alutacea*, *Citreonigra*, *Erubescentia* and *Restricta* grow restrictedly or moderately fast, while species of series *Lapidosa* and *Corylophila* tend to be more spreading. Growth at 37 °C is generally present in species of series *Alutacea*, *Erubescentia* and *Restricta* and absent in *Citreonigra* and *Corylophila*, though exceptions are present in most series; species of ser. *Lapidosa* show variable growth at 37 °C. At this moment, it is not possible to distinguish the series on phenotypic characters and this could be subject of future studies.  

**Section *Inusitata*** Houbraken & Frisvad, ***sect*. *nov*.** MycoBank MB834274.  

*Type*: *Penicillium inusitatum* D.B. Scott, Mycopathol. Mycol. Appl. 36: 20. 1968.  

*Etymology*: Named after the type species of the section, *P*. *inusitatum*.  

*Diagnosis*: *Phylogeny*: Section *Inusitata* belongs to subgen. *Aspergilloides* and is phylogenetically sister to sect. *Exilicaulis* (Fig. 18). *Morphology & physiology*: Colonies restricted; conidial colour *en masse* dull green or glaucous grey; conidiophores monoverticillate (*P*. *fractum*) or biverticillate and divaricate (*P*. *inusitatum*); conidia ellipsoidal or cylindrical, smooth; growth at 37 °C present. *Sexual morph* eupenicillium-type, yellow, yellowish brown or orange-brown; ascospores globose, spinose (without flanges or furrows). Section description based on Pitt (1980) and Houbraken & Samson (2011).  

*Included species*: *Penicillium fractum*, *P*. *inusitatum*.  

*Extrolites*: No extrolites have been detected in the two species in ser. *Inusitata* yet.  

*Notes*: Taxa belonging to this section were previously classified in *Penicillium* sect. *Fracta*. Section *Fracta* was (incorrectly) typified with *Penicillium ornatum*, a member of sect. *Ramigena*. Section *Fracta* is therefore considered a synonym of sect. *Ramigena* and the new section *Inusitata* is here proposed. A subdivision of sect. *Inusitata* is not made here and *Penicillium* ser. *Inusitata* is only informally introduced here. *Eupenicillium* ser. *Fracta* (MycoBank MB832945) (Pitt 1980) was formally introduced and is the basionym of this tentative series.  

**Section *Gracilenta*** Houbraken & Samson, Stud. Mycol. 70: 40. 2011. MycoBank MB563131.  

*Type*: *Penicillium gracilentum* Udagawa & Y. Horie, Trans. Mycol. Soc. Japan 14: 373. 1973.  

*Description*: See Houbraken & Samson (2011), this study (phylogeny); a modern overview of this section is lacking.  

Series ***Angustiporcata*** Houbraken & Frisvad, ***ser*. *nov*.** MycoBank MB834275.  

*Type*: *Penicillium angustiporcatum* Takada & Udagawa, Trans. Mycol. Soc. Japan 24: 143. 1983.  

*Etymology*: Named after the type species of the series, *Penicillium angustiporcatum*.  

*Diagnosis*: *Phylogeny*: Series *Angustiporcata* belongs to subgen. *Aspergilloides*, sect. *Gracilenta* and is phylogenetically sister to series *Estinogena*, *Gracilenta* and *Macrosclerotiorum* (Fig. 18). *Morphology & physiology*: Colonies restricted to moderately fast; conidial colour *en masse* undetermined; conidiophores predominantly biverticillate, occasionally monoverticillate, smooth; conidia broadly ellipsoidal, smooth; growth at 37 °C absent. *Sexual morph* eupenicillium-type, flesh-coloured or pale yellowish brown, small (50–150 μm); ascospores broadly ellipsoidal, with two prominent equatorial ridges, convex rugose, with several low ribs. Series description based on Takada & Udagawa (1983).  

*Included species*: *Penicillium angustiporcatum*.  

*Extrolites*: No extrolites have been found in *P*. *angustiporcatum*.  

Series ***Estinogena*** Houbraken & Frisvad, ***ser*. *nov*.** MycoBank MB834276.  

*Type*: *Penicillium estinogenum* A. Komatsu & S. Abe ex G. Sm., Trans. Brit. Mycol. Soc. 46: 335. 1963.  

*Etymology*: Named after the type species of the series, *Penicillium estinogenum*.  

*Diagnosis*: *Phylogeny*: Series *Estinogena* belongs to subgen. *Aspergilloides*, sect. *Gracilenta* and is phylogenetically sister to ser. *Macrosclerotiorum* (Fig. 18). *Morphology & physiology*: Colonies spreading; reverse dark olive; conidial colour *en masse* blue-green; conidiophores symmetrically biverticillate, rough-walled; conidia globose or ellipsoidal; smooth or finely roughened. *Sexual morph* unknown; sclerotia not observed in culture. Series description based on Ramírez (1982).  

*Included species*: *Penicillium estinogenum*.  

*Extrolites*: *Penicillium estinogenum* produces asterric acid, erdin, estin, geodin geodin hydrate in addition to dehydocurvularin and patulin. Production of verruculogen (Day *et al.* 1980) could not be confirmed, and the original strain producing this secondary metabolite was found to be a new species (not yet described).  

Series ***Gracilenta*** Houbraken & Frisvad, ***ser*. *nov*.** MycoBank MB834277.  

*Type*: *Penicillium gracilentum* Udagawa & Y. Horie, Trans. Mycol. Soc. Japan 14: 373. 1973.  

*Etymology*: Named after the type species of the series, *Penicillium gracilentum*.  

*Diagnosis*: *Phylogeny*: Series *Gracilenta* belongs to subgen. *Aspergilloides*, sect. *Gracilenta* and is phylogenetically sister to series *Estinogena* and *Macrosclerotiorum* (Fig. 18). *Morphology & physiology*: Colonies growing moderately fast; conidial colour *en masse* dull blue-green to greenish grey; conidiophores predominantly monoverticillate, sometimes divaricate; conidia (broadly) ellipsoidal, smooth or finely roughened; growth at 37 °C absent. *Sexual morph* eupenicillium-type, greyish yellow-brown; ascospores ellipsoidal, with two equatorial sinuous ridges, convex scattered with small spines. Series description based on Udagawa & Horie (1973) and Pitt (1980).  

*Included species*: *Penicillium gracilentum*.  

*Extrolites*: No known extrolites were found in *P*. *gracilentum*.  

Series ***Macrosclerotiorum*** Houbraken & Frisvad, ***ser*. *nov*.** MycoBank MB834278.  

*Type*: *Penicillium macrosclerotiorum* L. Wang *et al.*, Mycol. Res. 111: 1244. 2007.  

*Etymology*: Named after the type species of the series, *Penicillium macrosclerotiorum*.  

*Diagnosis*: *Phylogeny*: Series *Macrosclerotiorum* belongs to subgen. *Aspergilloides*, sect. *Gracilenta* and is phylogenetically sister to ser. *Estinogena* (Fig. 18). *Morphology & physiology*: Colonies growing moderately to fast; conidial colour *en masse* variable, grey-green to olive-grey; conidiophores monoverticillate, smooth; conidia globose, smooth or finely roughened; growth at 37 °C absent (*P*. *macrosclerotiorum*) or present (*P*. *apimei*, *P*. *aquaticum*). *Sexual morph* unknown; sclerotia absent (*P*. *apimei*) or present (*P*. *aquaticum, P*. *macrosclerotiorum*), white when young, becoming ivory in age. Series description based on Wang et al., 2007a, Barbosa et al., 2018 and Wanasinghe *et al.* (2018).  

*Included species*: *Penicillium apimei*, *P*. *aquaticum*, *P*. *macrosclerotiorum*.  

*Extrolites*: *Penicillium macrosclerotium* produces asterric acid, erdin, estin, geodin geodin hydrate like *P*. *estinogenum* in the sister ser. *Estinogena*.  

*Notes*: Comparison of the phenotypic characters at section level did not reveal many significant similarities, except that all produce an olive-brown to brown reverse on agar media (Houbraken and Samson, 2011, Barbosa et al., 2018). The inability to grow at 37 °C was also mentioned as a shared character of the species belonging to this section. However, *P*. *apimei* and *P*. *aquaticum* were recently added to sect. *Gracilenta* and both are able to grow at this temperature (Barbosa et al., 2018, Wanasinghe et al., 2018). Section *Gracilenta* has not yet been subject of a taxonomic study; however, the phylogenetic data shows there are four main lineages within the section. These lineages are here treated as series. Species of ser. *Macrosclerotiorum* share the production of monoverticillate conidiophores and globose conidia, and this combination of characters is not observed in the other sect. *Gracilenta* series. Series *Angustiporcata* and *Gracilenta* are monotypic series. Species in these series are the only ones in the section that reproduce sexually. They differ in their conidiophore branching complexity (monoverticillate *vs* biverticillate) and convex ornamentation of the ascospores (bars *vs* spines). *Penicillium estinogenum* is the sole member of ser. *Estinogena*. This strictly asexual reproducing species grows rapidly and produces rough-walled, biverticillate conidiophores. These features are not observed in the other taxa of sect. *Gracilenta*.  

**Section *Griseola*** Houbraken & Frisvad, ***sect*. *nov*.** MycoBank MB834279.  

*Type*: *Penicillium griseolum* G. Sm., Trans. Brit. Mycol. Soc. 40: 485. 1957.  

*Etymology*: Named after the type species of the section, *Penicillium griseolum*.  

*Diagnosis*: *Phylogeny*: Section *Griseola* belongs to subgen. *Aspergilloides* and is phylogenetically sister of sections *Aspergilloides*, *Charlesia*, *Eremophila* and *Sclerotiorum* (Fig. 2, Fig. 18). *Morphology & physiology*: Colonies growing moderately quickly, thin and transparent on Czapek agar; texture funiculose; conidial colour *en masse* grey; conidiophores monoverticillate, smooth, short; conidia globose, conspicuously echinulate, with conspicuous connectives. *Sexual morph* unknown; sclerotia not observed in culture. Section description based on Smith (1957).  

*Included species*: *Penicillium griseolum*.  

*Extrolites*: No known extrolites have been reported from *P*. *griseolum*.  

*Notes*: *Penicillium griseolum* was considered to be closely related to *P*. *terlikowskii* (= *P*. *glabrum*), from which it differs in its very poor growth on Czapek agar, its lack of green colour on malt extract agar, its different reverse colour, and in the curious tendency to form oidium-like chains of very slow ripening conidia (Smith 1957). Later, Pitt (1980) assigned this species to synonymy with *P*. *restrictum* based on phenotypic similarity, while Ramírez (1982) and Pitt *et al.* (2000) considered the species distinct. Using sequence data, Peterson & Horn (2009) showed that *P*. *griseolum* is a distinct species, though the phylogenetic position in the genus remained unresolved. In the phylogenetic analysis of the genus, Houbraken & Samson (2011) placed this species sister to sections *Aspergilloides*, *Charlesia* and *Sclerotiorum*; however, statistical support was generally poor. In this study, this relationship is confidently supported (Fig. 2). In order to maintain monophyly, sect. *Griseola* is introduced for this species. A subdivision of the section cannot be made and therefore ser. *Griseola* is only informally introduced here (Table 5).  

**Section *Lanata-Divaricata*** Thom, The Penicillia: 157, 328. 1930. MycoBank MB834002.

*Synonyms*: *Penicillium* sect. *Funiculosa* Thom, The Penicillia: 157, 358. 1930.

*Penicillium* sect. *Divaricata* [as “*Divaricatum*”] Raper & Thom ex Pitt, The Genus Penicillium: 238. 1980 [1979].

*Penicillium* sect. *Furcata* [as “*Furcatum*”] Pitt, The Genus Penicillium: 272. 1980 [1979].

*Eupenicillium* sect. *Javanica* (Pitt) Stolk & Samson, Stud. Mycol. 23: 55. 1983.  

*Type*: *Penicillium janthinellum* Biourge, Cellule 33: 258. 1923.  

*Description*: See Houbraken and Samson, 2011, Visagie et al., 2015 (phylogeny, morphology).  

Series ***Dalearum*** Houbraken & Frisvad, ***ser*. *nov*.** MycoBank MB834280.  

*Type*: *Penicillium daleae* K.W. Zaleski, Bull. Int. Acad. Polon. Sci., Sér. B., Sci. Nat. 1927: 495. 1927.  

*Etymology*: Named after the type species of the series, *Penicillium daleae*.  

*Diagnosis*: *Phylogeny*: Series *Dalearum* belongs to subgen. *Aspergilloides*, sect. *Lanata-Divaricata* and is phylogenetically sister to series *Rolfsiorum* and *Simplicissima* (represented by *P*. *subrubescens* and *P*. *simplicissimum* in Supplementary Fig. S1, resp.). *Morphology & physiology*: Colonies growing moderately fast or spreading; conidial colour *en masse* dull green, blueish green, grey-green; conidiophores monoverticillate, biverticillate and divaricate, occasionally terverticillate, generally smooth, sometimes finely rough-walled; conidia variable shaped (globose, subglobose, (broadly) ellipsoidal), smooth or rough-walled, spinose; growth at 37 °C variable. *Sexual morph* generally not observed in culture, sometimes present, eupenicillium-type, homothallic, yellow-brown, ochre or yellowish cream; ascospores ellipsoidal, with two (shallow) longitudinal flanges (*P*. *abidjanum*, *P*. *zonatum*) or traces of an inconspicuous equatorial ridge (*P*. *vanderhammenii*), valves (finely) spinose).  

*Included species*: *Penicillium abidjanum*, *P*. *amphipolaria*, *P*. *austrosinense*, *P*. *daleae*, *P*. *griseopurpureum*, *P*. *guaibinense*, *P*. *jianfenglingense*, *P*. *penarojense*, *P*. *rubriannulatum*, *P*. *singorense*, *P*. *vanderhammenii*, *P*. *viridissimum*, *P*. *zonatum*.  

*Extrolites*: *Penicillium abidjanum* produces N-methylgliovirin. *Penicillium daleae* produces 3-(dimethylaminobenzyl)-N-(1,1-ddimethyl-2-propenyl)indol (Lam *et al.* 1994), JBIR-54 (Mukai *et al.* 2009), penicilliols (Kimura *et al.* 2009), isopenicins (Tang *et al.* 2019b), andrastin A, antarone A, curvulic acid, gregatins, penicillifuranone A, viomellein, vioxanthin, and xanthomegnin. *Penicillium griseopurpureum* produces andrastin A, curvulic acid and xanthoepocin. *Penicillium penarojense* produces paxillin, paspaline, and shearinins/janthitrems (Houbraken *et al.* 2011b), *P*. *vanderhammenii* produces paxillin, paspaline, penicilic acid and shearinins/janthitrems (Houbraken *et al.* 2011b), *P*. *zonatum* produces brefeldin A, curvulic acids, shearinins/janthitrems, viomellein, vioxanthin and xanthomegnin. The species in the series thus share several extrolites. Some of these are also produced by other species in sect. *Lanata-Divaricata*.  

Series ***Janthinella*** Thom ex Pitt, The Genus Penicillium: 239. 1980 [1979]. MycoBank MB832959.

*Synonym*: *Eupenicillium* ser. *Javanica* Pitt, The Genus Penicillium: 113. 1980 [1979].  

*Type*: *Penicillium janthinellum* Biourge, Cellule 33: 258. 1923.  

*Diagnosis*: *Phylogeny*: Series *Janthinella* belongs to subgen. *Aspergilloides*, sect. *Lanata-Divaricata* and is phylogenetically sister to series *Dalearum*, *Rolfsiorum* and *Simplicissima* (represented by *P*. *abidjanum*, *P*. *subrubescens* and *P*. *simplicissimum* in Supplementary Fig. S1, resp.). *Morphology & physiology*: Colonies spreading; conidial colour *en masse* in shades of green, often grey-green; conidiophores biverticillate or divaricate, sometimes monoverticillate; stipes smooth-walled, sometimes roughened (*P*. *javanicum*); conidia variable in shape and ornamentation: globose, subglobose or (broadly) ellipsoidal and smooth, finely rough or rough-walled; growth at 37 °C generally present, sometimes absent (*P*. *meloforme*, *P*. *yunnanense*). *Sexual morph* not observed in culture (11 species) or present (13 species) (for details, see below in list of accepted species), eupenicillium-type, homothallic, creamish, yellow, yellow-brown or brown; ascospores ellipsoidal, with two distinct or inconspicuous longitudinal flanges, sometimes lacking equatorial ridges, convex ornamented or spinose. Series description based on Pitt, 1980, Stolk and Samson, 1983, Houbraken et al., 2011b and Visagie *et al.* (2015).  

*Included species*: *Penicillium brefeldianum*, *P*. *caperatum*, *P*. *cluniae*, *P*. *coeruleum*, *P*. *cremeogriseum*, *P*. *curticaule*, *P*. *ehrlichii*, *P*. *elleniae*, *P*. *glaucoroseum*, *P*. *janthinellum*, *P*. *javanicum*, *P*. *koreense*, *P*. *levitum*, *P*. *limosum*, *P*. *lineolatum*, *P*. *ludwigii*, *P*. *malacosphaerulum*, *P*. *meloforme*, *P*. *ortum*, *P*. *raperi*, *P*. *reticulisporum*, *P*. *setosum*, *P*. *uruguayense*, *P*. *yunnanense*.  

*Extrolites*: *Penicillium brefeldianum* produces bredinin (Mizuno *et al.* 1974), brefeldin A (Härri et al., 1963, Jouda et al., 2016), citromycetin and fulvic acid (Jouda et al., 2014, Jouda et al., 2016), eupenifeldin (Mayerl *et al.* 1993), hydroxyphenylglyoxaladoxime (Jouda *et al.* 2016), palitantin (Demetriadou *et al.* 1985) and penialidins (Jouda et al., 2014, Jouda et al., 2016, Cheng et al., 2018). *Penicillium caperatum* produces viridicatumtoxin and aplora indoloterpenes, and *P*. *cluniae* produces brefeldin A, janthitrems/sharinins, paraherquamides and cyclic dipeptides (diketopiperazines) (López-Gresa *et al.* 2006). *Penicillium cremeogriseum* produces brefeldin A, janthitrems/shearinins and fulvic acid; *P*. *elleniae* was reported to produce paxillin and sorbicillins (Houbraken *et al.* 2011b); *P*. *glaucoroseum* produces brefeldin A, fulvic acid and viomellein, vioxanthin and xanthomegnin; *P*. *janthinellum* and *P*. *levitum* produces viomellein, vioxanthin and xanthomegnin. Brefeldin A, fulvic acid, paspaline and palitantin are produced by *P*. *ludwigii* and *P*. *reticulisporum* produces xanthoepocin. These data show that the species in ser. *Janthinella* have many extrolites in common.  

Series ***Oxalica*** Raper & Thom ex Pitt, The Genus Penicillium: 273. 1980 [1979]. MycoBank MB832963.  

*Type*: *Penicillium oxalicum* Currie & Thom, J. Biol. Chem. 22: 289. 1915.  

*Diagnosis*: *Phylogeny*: Series *Oxalica* belongs to subgen. *Aspergilloides*, sect. *Lanata-Divaricata* and is phylogenetically sister to all other series of sect. *Lanata-Divaricata* (Fig. 2, Fig. 18). *Morphology & physiology*: Colonies spreading; conidial colour *en masse* yellowish green, grey-green or dull green; texture strictly velutinous and crustose; conidiophores biverticillate, occasionally mono- or terverticillate, smooth; conidia ellipsoidal, smooth or finely roughened; growth at 37 °C present. *Sexual morph* unknown; sclerotia not observed in culture.  

*Included species*: *Penicillium diatomitis*, *P*. *oxalicum*, *P*. *soosanum*.  

*Extrolites*: *Penicillium oxalicum* produces oxaline, meleagrin, glandicolin A & B, and roquefortine C (Nagel et al., 1974, Nagel et al., 1976, Steyn and Vleggaar, 1983, Chen et al., 2015), secalonic acids, paecilin C and penicillixanthone (Steyn, 1970, Li et al., 2010, Kim et al., 2012, Bao et al., 2013, Wang et al., 2013b, Chen et al., 2015, Liu et al., 2015, Chen et al., 2019), oxalic acid (Currie & Thom 1915), benzenedicarboxylic acid and benzimidazole (Ahmad *et al.* 2019), (Z)-3-(3,4-dihydroxyphenyl)-2-foramidoacrylate and decaturins and oxalicins (Ubillas et al., 1989, Wang et al., 2013a, Li et al., 2015b, Zhang et al., 2015a), 2,2’,4,4’-tetrahydroxy-8’-methyl-6-methoxy-acyl-ethyl-diphenylmethanone (Liu et al., 2015, Liu et al., 2016a), hydroxyscytalone (Ji *et al.* 2014), coniochaetones and penicillones (Bao *et al.* 2014), gymnemagenin (Parthasarathy & Sathiyabama 2014), 2-(4-hydroxybenzoyl) quinazolin-4(3H)-one, 2-(4-hydroxybenzyl) quinazolin-4(3H)-one, methyl 4-hydroxyphenylacetate (= penipanoid C), rubinaphthin, citreorosein, emodin, isorhodoptilometrin and endocrocin (Li et al., 2010, Bao et al., 2013, Shen et al., 2013, Chen et al., 2015), oxalicumones (Sun et al., 2012, Sun et al., 2013, Zhang et al., 2013), 6,8-dihydroxy-3-methyl-9-oxo-9H-xanthene-1-carboxylic acid, 5-hydroxy-2-methoxybenzoic acid and 2-phenylacetic acid (Li *et al.* 2010),and vermiculidiol (Kim *et al.* 2012). *Penicillium soosanum* and *P*. *diatomitis* appear to be the producers of penioxalamine, as the ITS sequence (KJ101590) reported by Hu *et al.* (2014) is different from that of *P*. *oxalicum sensu stricto* (Kubátová *et al.* 2019).  

Series ***Rolfsiorum*** Houbraken & Frisvad, ***ser*. *nov*.** MycoBank MB834281.  

*Type*: *Penicillium rolfsii* Thom, Penicillia: 489. 1930.  

*Etymology*: Named after the type species of the series, *Penicillium rolfsii*.  

*Diagnosis*: *Phylogeny*: Series *Rolfsiorum* belongs to subgen. *Aspergilloides*, sect. *Lanata-Divaricata* and is phylogenetically sister to ser. *Simplicissima* (represented by *P*. *simplicissimum* in Supplementary Fig. S1). *Morphology & physiology*: Colonies spreading; conidial colour *en masse* grey-green, dull green; conidiophores terminally biverticillate, sometimes terverticillate or divaricate, rough-walled; conidia generally globose to subglobose, occasionally ellipsoidal or fusiform, smooth, finely roughened or rough; growth at 37 °C variable. *Sexual morph* unknown; sclerotia generally not observed in culture, present in *P*. *rolfsii* (white or pink) and *P*. *soliforme* (light yellow).  

*Included species*: *Penicillium annulatum*, *P*. *bissettii*, *P*. *camponotum*, *P*. *excelsum*, *P*. *flaviroseum*, *P*. *fructuariae-cellae*, *P*. *hainanense*, *P*. *ochrochloron*, *P*. *piscarium*, *P*. *pulvillorum*, *P*. *rolfsii*, *P*. *soliforme*, *P*. *subrubescens*, *P*. *svalbardense*, *P*. *terrarumae*, *P*. *vasconiae*.  

*Extrolites*: *Penicillium bissettii* produces aurantioclavine, neooxaline, meleagrin, roquefortine C and other precursors, oxalicine B and penicillic acid (Visagie *et al.* 2016b), *P*. *camponotum* produces andrastin A-D, citrinalin, mangrovamides, marcfortine A and B and patulin (Visagie *et al.* 2016b, and results reported here), *P*. *excelsum* produces andrastin A, curvulic acid, penicillic acid and xanthoepocin (Taniwaki *et al.* 2015), *P*. *ochrochloron* produces andrastin A, okaramine A-R, penicillic acid, penitrems including penitrem A, viridicatumtoxin and xanthoepocin (Hayashi et al., 1988, Hayashi et al., 1989, Hayashi et al., 1991a, Hayashi et al., 1995, Murao et al., 1988, Hayashi and Sakaguchi, 1998, Shiono et al., 1999, Shiono et al., 2000, Tuthill et al., 2001, Nielsen and Smedsgaard, 2003), *P*. *piscarium* produces haenamindole, janthitrems/shearinins, pulvilloric acid and xanthoepocin (Tuthill *et al.* 2001 and results reported here) and results reported here), *P*. *pulvillorum* produces andrastin A, curvulic acids, penicilic acid, pulvilloric acid, and xanthoepocin (Brian et al., 1957, Barber et al., 1986, Tuthill et al., 2001), *P*. *rolfsii* produces decaturins/oxalicins, paraherquamides, patulin, penicillic acid, viridicatumtoxin and xanthoepocin (data reported here), *P*. *subrubescens* and *P*. *svalbardense* produce xanthoepocin (Sonjak et al., 2007, Mansouri et al., 2013), and *P*. *vasconiae* produces curvulic acid, janthitrems/shearinins and pyripyropenes (results reported here). Species in ser. *Rolfsiorum* share many secondary metabolites, including andrastins, penicillic acid, curvulic acid, pulvilloric acid, viridicatumtoxin, janthitrems/shearinins, decaturins/oxalicins. However, not all species in the series produce these metabolites and several metabolites are found in other series in sect. *Lanata-Divaricata* as well.  

Series ***Simplicissima*** Houbraken & Frisvad, ***ser*. *nov*.** MycoBank MB834282.  

*Type*: *Penicillium simplicissimum* (Oudem.) Thom, Penicillia: 335. 1930.  

*Etymology*: Named after the type species of the series, *Penicillium simplicissimum*.  

*Diagnosis*: *Phylogeny*: Series *Simplicissima* belongs to subgen. *Aspergilloides*, sect. *Lanata-Divaricata* and is phylogenetically sister to ser. *Rolfsiorum* (represented by *P*. *subrubescens* in Supplementary Fig. S1). *Morphology & physiology*: Colonies spreading; conidial colour *en masse* grey-green or dull green; conidiophores biverticillate or divaricate, sometimes terverticillate; stipes smooth or rough-walled; conidia variable in size and shape, globose, subglobose, (broadly) ellipsoidal, ornamentation smooth, finely rough or rough-walled, sometimes in spiral pattern; growth at 37 °C variable. *Sexual morph* unknown; sclerotia generally not observed in culture, sometimes present, *e.g*., in *P*. *mariae-cruscis*, *P*. *tanzanicum* (yellowish), *P*. *araracuaraense* (yellow-brown), *P*. *griseoflavum* (greyish orange).  

*Included species*: *Penicillium alagoense*, *P*. *araracuaraense*, *P*. *brasilianum*, *P*. *cataractarum*, *P*. *echinulonalgiovense*, *P*. *globosum*, *P*. *griseoflavum*, *P*. *guangxiense*, *P*. *infrabuccalum*, *P*. *laevigatum*, *P*. *mariae-crucis*, *P*. *onobense*, *P*. *panissanguineum*, *P*. *paraherquei*, *P*. *pedernalense*, *P*. *simplicissimum*, *P*. *skrjabinii*, *P*. *spinuliferum*, *P*. *tanzanicum*, *P*. *wotroi*.  

*Extrolites*: *Penicillium araracuarense* produces pulvilloric acid, *P*. *brasilianum* produces aspterric acid, austins, brasilianoids and berkeleytrione (Hayashi et al., 1994, Matsuda et al., 2016, Zhang et al., 2018, Zhang et al., 2019), brasiliamides (Fujita et al., 2002, Fujita and Hayashi, 2004, Fill Taicia et al., 2009), fischerin, fumitremorgsins, verruculogen, spirotryprostatins and cyclotryprostatins (Frisvad, 1989, Zhang et al., 2019), JBIR 113, 114 & 115 (Koyama *et al.* 2012), neosartorin, penicillic acid (Frisvad et al., 1989, Kang and Kim, 2004, Schürmann et al., 2010), viridicatumtoxin and spirohexalines (Frisvad et al., 1989, Hayashi et al., 1994, Inokoshi et al., 2013, Inokoshi et al., 2016), and xanthoepocin (Tuthill et al., 2001, Bazioli et al., 2017), *P*. *cataractum* produces andrastin A-D, pulvilloric acid and trichodermamide A and C (Visagie *et al.* 2016b), *P*. *echinulonalgiovense* produces andrastin A and pulvilloric acid, *P*. *mariae-crucis* produces xanthomegnin and viomellein, *P*. *onobense* produces brefeldin A, janthitrems/shearinins, and 2-(4-hydroxyphenyl)-2-oxo acetaldehyde oxime, *P*. *paraherquei* produces fumitremorgins (Yoshizawa *et al.* 1976), paraherquonin (Okuyama *et al.* 1983), and paraherquamides (Yamazaki *et al.* 1981b), *P*. *panissanguineum* produces andrastins and pulvilloric acid (Visagie *et al.* 2016b), *P*. *simplicissimum* produces andrastin A, austalides (referred to as chromanols), paraherquamides, and xanthoepocin (Tuthill *et al.* 2001), *P*. *tanzanicum* produces bisdechlorogeodin, fiscalin C and pulvilloric acid (Visagie *et al.* 2016b) and *P*. *wotroi* produces pulvilloric acid (Houbraken *et al.* 2011b). In ser. *Simplicissima* many extrolites are in common, but they are also common in series *Rolfsiorum*, *Dalearum* and *Janthinella*. Series *Oxalica* species generally have least extrolites in common with the other series in sect. *Lanata-Divaricata*.  

*Notes on series of sect*. *Lanata-Divaricata*: The deeper nodes within sect. *Lanata-Divaricata* are often poorly supported in Fig. 18, and the relationships between the series are therefore based on representative species in Supplementary Fig. S1. Using a multigene phylogenetic approach, Visagie *et al.* (2015) studied the relationship within sect. *Lanata-Divaricata*. Their new species resolved in three consistent, well-supported clades, named the *P*. *janthinellum*-, *P*. *javanicum*- and *P*. *rolfsii*-clade. In this study, sect. *Lanata-Divaricata* is subdidived in five series: *Janthinella*, *Dalearum*, *Oxalica*, *Rolfsiorum* and *Simplicissima*. The proposed *P*. *janthinellum*- and *P*. *javanicum*-clades in Visagie *et al.* (2015) are combined and equivalent to ser. *Janthinella*, and ser. *Rolfsiorum* includes the taxa of the *P*. *rolfsii*-clade. The majority of species outside the three clades of Visagie *et al.* (2015) belong to series *Dalearum*, *Oxalica* and *Simplicissima*, and exceptions are *P*. *ehrlichii*, *P*. *meloforme*, *P*. *coeruleum*, *P*. *levitum* (all ser. *Janthinella*), and *P*. *vasconiae* (ser. *Rolfsiorum*). The series classification in sect. *Lanata-Divaricata* is primarily based on the presented phylogeny and the series are difficult to distinguish phenotypically. Series *Oxalica* is distinct: species in this series produce spreading colonies, which are strictly velutinous and crustose and the conidia are ellipsoidal. None of the species in series *Oxalica* (three species), *Rolfsiorum* (16 species) and *Simplicissima* (21 species) are known to produce a sexual morph. In contrast, 13 out of the 24 ser. *Janthinella* species and three out of 13 ser. *Dalearum* species produce a sexual morph. The majority of species of series *Oxalica* and *Janthinella* are able to grow at 37 °C, while growth is more variable in the species of the section. Furthermore, ser. *Rolfsiorum* species produce rough-walled conidiophores and mostly globose conidia; however, these characters are shared with species in other series.  

**Section *Lasseniorum*** Houbraken & Frisvad, ***sect*. *nov*.** MycoBank MB834283.  

*Type*: *Penicillium lassenii* Paden, Mycopathol. Mycol. Appl. 43: 266. 1971.  

*Etymology*: Named after the type species of the section, *Penicillium lassenii*.  

*Diagnosis*: *Phylogeny*: Section *Lasseniorum* belongs to subgen. *Aspergilloides* and is phylogenetically most closely related to sect. *Alfrediorum*. *Morphology & physiology*: Colonies growing very restricted; conidial colour *en masse* pale grey-green; conidiophores monoverticillate or terminal biverticillate, smooth; conidia subglobose to ellipsoidal, smooth; growth at 37 °C absent. *Sexual morph* eupenicillium-type, homothallic, yellow-brown or tan; ascospores ellipsoidal, with two closely spaced ridges, convex smooth. Section description based on Paden (1971) and Pitt (1980).  

*Included species*: *Penicillium lassenii*.  

*Extrolites*: *Penicillium lassenii* produce extrolites which have not yet been structure elucidated.  

*Notes*: Based on a multigene phylogenetic analysis, Houbraken & Samson (2011) accommodated *P*. *lassenii*, together with *P*. *cryptum*, in sect. *Torulomyces*. Visagie *et al.* (2016a) questioned the placement of *P*. *lassenii* (and *P*. *cryptum*) in sect. *Torulomyces* and this observation is confirmed here. *Penicillium lassenii* produces monoverticillate or terminal biverticillate conidiophores and smooth-walled conidia, while sect. *Torulomyces* species produce conidiophores with solitary phialides and typically roughened conidia (Paden, 1971, Visagie et al., 2016a). A series subdivision of the section is not proposed and therefore ser. *Lasseniorum* is only informally introduced here (Table 5).  

**Section *Ochrosalmonea*** Houbraken & Samson, Stud. Mycol. 70: 33. 2011. MycoBank MB563127.  

*Type*: *Penicillium ochrosalmoneum* Udagawa, J. Agric. Sci. Tokyo Nogyo Daig. 5: 10. 1959.  

*Description*: See Houbraken & Samson (2011) (morphology, phylogeny), and here (under ser. *Ochrosalmonea*).  

Series ***Ochrosalmonea*** Stolk & Samson, Adv. Pen. Asp. Syst.: 177. 1986 [1985]. MycoBank MB832728.  

*Type*: *Penicillium ochrosalmoneum* Udagawa, J. Agric. Sci. Tokyo Nogyo Daig. 5: 10. 1959.  

*Diagnosis*: *Phylogeny*: Series *Ochrosalmonea* belongs to subgen. *Aspergilloides*, sect. *Ochrosalmonea* and the series (and section) are phylogenetically related to *Thysanophora*. *Morphology & physiology*: Colonies growing restrictedly (*P*. *isariiforme*) or rapidly (*P*. *ochrosalmoneum*); conidial colour *en masse* greyish green or dull green; conidiophores predominantly biverticillate, occasionally with additional branches, smooth; conidia subglobose to apiculate (*P*. *ochrosalmoneum*) or ellipsoidal (*P*. *isariiforme*), smooth; growth at 37 °C absent (*P*. *isariiforme*) or present (*P*. *ochrosalmoneum*). *Sexual morph* produced in *P*. *ochrosalmoneum*, eupenicillium-type, homothallic, bright yellow or orange; ascospores ellipsoidal, with two longitudinal ridges, convex smooth to roughened or spinulose; sclerotia absent. Description based on Pitt (1980) and Houbraken & Samson (2011).  

*Included species*: *Penicillium isariiforme*, *P*. *ochrosalmoneum*.  

*Extrolites*: Both species in the series produce citreoviridin (Wicklow & Cole 1984).  

*Notes on series and section Ochrosalmonea*: *Penicillium ochrosalmoneum* and *P*. *isariifrome* are phenotypically unrelated, but both are accommodated in sect. *Ochrasalmonea* based on the result of our phylogenetic analysis. For more details, see Houbraken & Samson (2011).  

**Section *Ramigena*** Thom, The Penicillia: 156, 225. 1930. MycoBank MB834004.

*Synonym*: *Penicillium* sect. *Fracta* Houbraken & Samson, Stud. Mycol. 70: 35. 2011.  

*Type*: *Penicillium cyaneum* (Bainier & Sartory) Biourge, Cellule 33: 102. 1923.  

*Description*: See Houbraken & Samson (2011) (morphology, phylogeny); a taxonomic study on the section is lacking.  

Series ***Georgiensia*** Houbraken & Frisvad, ***ser*. *nov*.** MycoBank MB834284.  

*Type*: *Penicillium georgiense* S.W. Peterson & B.W. Horn, Mycologia 101: 79. 2009.  

*Etymology*: Named after the type species of the series, *Penicillium georgiense*.  

*Diagnosis*: *Phylogeny*: Series *Georgiensia* belongs to subgen. *Aspergilloides*, sect. *Ramigena* and is phylogenetically sister to ser. *Ramigena*. *Morphology & physiology*: Colonies restricted; conidial colour *en masse* pale to greyish green; conidiophores divaricate; stipes smooth; conidia ellipsoidal or subglobose, smooth-walled; growth at 37 °C present. *Sexual morph* unknown. Series description based on Peterson & Horn (2009).  

*Included species*: *Penicillium georgiense*.  

*Extrolites*: This species has not been examined for extrolites.  

Series ***Ramigena*** Houbraken & Frisvad, ***ser*. *nov*.** MycoBank MB834285.  

*Type*: *Penicillium cyaneum* (Bainier & Sartory) Biourge, Cellule 33: 102. 1923.  

*Etymology*: Named after the type species of the series, *Penicillium cyaneum*.  

*Diagnosis*: *Phylogeny*: Series *Ramigena* belongs to subgen. *Aspergilloides*, sect. *Ramigena* and is phylogenetically sister to ser. *Georgiensia*. *Morphology & physiology*: Colonies restricted; conidial colour *en masse* in shades of green (blue-green, dull green or grey-green); conidiophores strictly or predominantly monoverticillate, stipes smooth-walled; conidia generally ellipsoidal or pyriform, sometimes globose to subglobose (*P*. *ornatum*), smooth-walled; growth at 37 °C present or absent. *Sexual morph* generally not observed in culture, present in *P*. *ornatum*, eupenicillium-type, homothallic, buff to brown; ascospores broadly ellipsoidal, with two and sometimes four longitudinal flanges, convex smooth-walled. Series description based on Pitt (1980) and Houbraken & Samson (2011).  

*Included species*: *Penicillium capsulatum*, *P*. *cyaneum*, *P*. *hispanicum*, *P*. *ornatum*, *P*. *ramusculum*.  

*Extrolites*: *Penicillium capsulatum*, *P*. *ornatum* and *P*. *ramusculum* produce many unknown secondary metabolites, while *P*. *cyaneum* and *P*. *hispanicum* share production of deoxybrevianamide E. *Penicillium hispanicum* also produces asperflavin and bisanthrons.  

*Notes*: *Penicillium* sect. *Fracta* is typified with *Penicillium ornatum*, a member sect. *Ramigena*. Section *Fracta* is therefore considered synonym of sect. *Ramigena*. Section *Ramigena* was introduced by Thom (1930) and the section was accepted in the classification proposed by Houbraken & Samson (2011). A taxonomic study of this section is lacking and the taxonomic status of *P*. *cyaneum*, *P*. *dierckxii* and *P*. *sublateritium* is unknown. Following Pitt, 1980, Houbraken and Samson, 2011 accepted these three species, even though *RPB2* sequence data showed that these species are very closely related. This close relationship is supported by *BenA* and *CaM* sequences (>99.5 % homology) and we therefore treat *P*. *dierckxii* and *P*. *sublateritium* as synonyms of *P*. *cyaneum*. *Penicillium georgiense* is the sole member of ser. *Georgiensia*. Using sequence data, Peterson & Horn (2009) suggested a relationship of *P*. *georgiense* with *P*. *thiersii* (sect. *Aspergilloides*) and Houbraken & Samson (2011) classified this species in sect. *Aspergilloides*. While studying the taxonomy of sect. *Aspergilloides*, Houbraken *et al.* (2014b) showed that it is not part of this section and we here confidently classify *P*. *georgiense* in sect. *Ramigena*. *Penicillium georgiense* (ser. *Georgiensia*) differs from the species in the related ser. *Ramigena* by the production of divaricate branched conidiophores, while ser. *Ramigena* members are strictly or predominantly monoverticillate.  

**Section *Sclerotiorum*** [as “*Sclerotiora*”] Houbraken & Samson, Stud. Mycol. 70: 32. 2011. MycoBank MB585167.  

*Type*: *Penicillium sclerotiorum* J.F.H. Beyma, Zentralbl. Bakteriol. Parasitenk., Abt. 2 96: 418. 1937.  

*Description*: See Houbraken and Samson, 2011, Visagie et al., 2013 (morphology, phylogeny).  

Series ***Adametziorum*** Houbraken & Frisvad, ***ser*. *nov*.** MycoBank MB834286.  

*Type*: *Penicillium adametzii* K.W. Zaleski, Bull. Int. Acad. Polon. Sci., Sér. B., Sci. Nat. 1927: 507. 1927.  

*Etymology*: Named after the type species of the series, *P*. *adametzii*.  

*Diagnosis*: *Phylogeny*: Series *Adametziorum* belongs to subgen. *Aspergilloides*, sect. *Sclerotiorum* and is phylogenetically sister to the other series of sect. *Sclerotiorum*. *Morphology & physiology*: Colonies restricted or moderately; conidial colour *en masse* dull green, grey-green; conidiophores monoverticillate, smooth-walled; conidia globose, subglobose or ellipsoidal, smooth or finely roughened; growth at 37 °C variable, absent or present. *Sexual morph* unknown; sclerotia absent or present, white (*P*. *alexiae*). Series description based on Visagie *et al.* (2013).  

*Included species*: *Penicillium adametzii*, *P*. *adametzioides*, *P*. *alexiae*, *P*. *amaliae*, *P*. *angulare*, *P*. *arianeae*, *P*. *bilaiae*, *P*. *brocae*, *P*. *jugoslavicum*, *P*. *lilacinoechinulatum*, *P*. *mellis*, *P*. *reconvexovelosoi*∗, *P*. *restingae* [∗ not included in Fig. 18; for more information on the phylogenetic relationship, see Crous *et al.* (2019)].  

*Extrolites*: Series *Adametziorum* species general produce dithiodiketopirazines such as brocazines, gliotoxins, epicoccins, phomazines, penicibrocazines, and spirobrocazines. *Penicillium adametzioides* produces glyanthrypine and lapatins, kotanins and spinulosins (Larsen et al., 2005, Liu et al., 2014b), *Penicillium bilaiae* produces citromycins, citromycetins, bilains, pistillarin and cyclic dipeptides (diketopiperazines) (Capon *et al.* 2007), hyalodendrins, 2-hydroxy-3,6-dimethylbenzaldehyde, dibutylphthalate, and 4-hydroxy-3,6-dimethyl-2H-pyran-2-one (Savard *et al.* 1994), penipratynolene and picolinic acids (Nakahara *et al.* 2004) and citric acid & oxalic acid (Cunningham & Kuiack 1992). *Penicillium brocae* produces brocazines, spirobrocazines, penibrocazines, phenopyrrozines, brocaeloids, brocaenols, pyranonigrin A, and brocapyrones (Bugni et al., 2003, Meng et al., 2014, Meng et al., 2015a, Meng et al., 2015b, Meng et al., 2016, Meng et al., 2017, Zhang et al., 2015b). *Penicillium lilacinoechinulatum* produces gliotoxins, phenopyrrozin, spinulosin and dehydrocarolic acid.  

Series ***Herqueorum*** Houbraken & Frisvad, ***ser*. *nov*.** MycoBank MB834287.  

*Type*: *Penicillium herquei* Bainier & Sartory, Bull. Soc. Mycol. France 28: 121. 1912.  

*Etymology*: Named after the type species of the series, *Penicillium herquei*.  

*Diagnosis*: *Phylogeny*: Series *Herqueorum* belongs to subgen. *Aspergilloides*, sect. *Sclerotiorum* and phylogenetically sister to ser. *Sclerotiorum*. *Morphology & physiology*: Colonies growing moderately fast; conidial colour *en masse* dull green, grey-green; conidiophores generally biverticillate, mono- and terverticillate also occasionally produced, smooth or rough-walled; conidia ellipsoidal, smooth or rough-walled; growth at 37 °C absent. *Sexual morph* generally not observed in culture, present in *P*. *malachiteum*, eupenicillium-type, homothallic, malachite-green; ascospores ellipsoidal, with two-equatorial ridges; sclerotia absent or present (*P*. *herquei*), cream. Series description based on Yaguchi et al., 1993, Visagie et al., 2013 and Wang *et al.* (2017a).  

*Included species*: *Penicillium choerospondiatis*, *P*. *herquei*, *P*. *malachiteum*, *P*. *sanshaense*, *P*. *verrucisporum*.  

*Extrolites*: Species in ser. *Herqueorum* produce atrovenetin, herqueinone, herqueichrysin, peniciherqueinone, peniciphenalines, sclerodin, scleroderolide, sclerodione, sculenonone A & B, and xanthoherquein (Stodola et al., 1951, Galarraga et al., 1955, Harman et al., 1955, Barton et al., 1959, Narasimhachari et al., 1963, Narasimhachari and Vining, 1963, Narasimhachari and Ramaswami, 1966, Narasimhachari and Vining, 1972, Simpson, 1976, Robinson et al., 1992, Trotter, 1992, Tansakul et al., 2014, Nishikori et al., 2016, Li et al., 2018a), herqulines (Furusaki et al., 1980, Enomoto et al., 1996, Chiba et al., 2017, Yang et al., 2018), neocyclocitrinol (Marinho *et al.* 2009), peniciherquamides (Nishikori *et al.* 2016), hualyzin (Ding *et al.* 2008), penicilquei A-D (Zhou *et al.* 2019), penicillic acid, and these metabolites have been found in both *P*. *herquei* and *P*. *malachiteum*. There are no secondary metabolites in common with series *Adametziorum* or *Sclerotiorum*.  

Series ***Sclerotiorum*** Houbraken & Frisvad, ***ser*. *nov*.** MycoBank MB834288.  

*Type*: *Penicillium sclerotiorum* J.F.H. Beyma, Zentralbl. Bakteriol. Parasitenk., Abt. 2 96: 418. 1937.  

*Etymology*: Named after the type species of the series, *Penicillium sclerotiorum*.  

*Diagnosis*: *Phylogeny*: Series *Sclerotiorum* belongs to subgen. *Aspergilloides*, sect. *Sclerotiorum* and is phylogenetically sister to ser. *Herqueorum*. *Morphology & physiology*: Colonies growing moderately fast; conidial colour *en masse* dull green or grey-green; conidiophores monoverticillate, smooth to rough; conidia globose, subglobose or ellipsoidal, smooth to finely rough; growth at 37 °C variable, absent or present. *Sexual morph* produced in *P*. *hirayamae*, eupenicillium-type, homothallic, orange or brown; ascospores ellipsoidal, with two small closely appressed longitudinal flanges, convex rough-walled; sclerotia produced in *P*. *acidum*, *P*. *austrosinicum*, *P*. *johnkrugii*, *P*. *sclerotiorum*, *P*. *vanoranjei*, orange, white/grey, cream to yellow. Series description based on Rivera and Seifert, 2011, Visagie et al., 2013 and Wang *et al.* (2017a)  

*Included species*: *Penicillium acidum*, *P*. *austrosinicum*, *P*. *cainii*, *P*. *circulare*, *P*. *daejeonium*, *P*. *exsudans*, *P*. *fernandesiae*, *P*. *guanacastense*, *P*. *hirayamae*, *P*. *jacksonii*, *P*. *johnkrugii*, *P*. *mallochii*, *P*. *maximae*, *P*. *meliponae*, *P*. *sclerotiorum*, *P*. *vanoranjei*, *P*. *viticola*.  

*Extrolites*: All species examined in ser. *Sclerotiorum* produce sclerotiorins, rotiorin, penazaphilones, penicilazaphilones (Curtin and Reilly, 1940, Maccurin and Reilly, 1940, Birkinshaw, 1952, Jackman et al., 1958, Arai et al., 1995, Matsuzaki et al., 1995a, Matsuzaki et al., 1995b, Pairet et al., 1995, Nam et al., 2000, Lucas et al., 2007, Arunpanichlert et al., 2010, Hemtasin et al., 2016, Zhou et al., 2016, Wang et al., 2018, Jia et al., 2019, Tang et al., 2019a, Wu et al., 2019), penicilisorin, (+)-4,6-dimetylocta-2,4-dienoic acid and 5,6-dihydro-3,5,6-trimethylpyran-2-one (Arunpanichlert *et al.* 2010), pencolide (Birkinshaw et al., 1963, Lucas et al., 2007), carotenes (Mase *et al.* 1957), multicolic acid, multicolosic acid and multicolanic acid (Gudgeon et al., 1974, Gedge and Pattenden, 1979, Gudgeon et al., 1979, Holker et al., 1987) and atlantinone A. In general, all species with a reddish reverse in ser. *Sclerotiorum* produce azaphilones such as sclerotiorin.  

*Notes on series of sect*. *Sclerotiorum*: Visagie *et al.* (2013) divided sect. *Sclerotiorum* in three main clades. These clades are also observed in our phylogenetic study (Fig. 18), and we treat those clades here as series. Specific phenotypic characters that define the three series of the section could not be identified, but there are some polythetic features for each series (Visagie *et al.* 2013). Generally, species in ser. *Herqueorum* produce biverticillate conidiophores, in contrast with species of series *Sclerotiorum* and *Adametziorum*, which are monoverticillate. Taxa in ser. *Sclerotiorum* have colonies in orange colours and lack the strongly coloured, soluble pigments such as those generally observed in ser. *Adametziorum* species.  

**Section *Stolkia*** Houbraken & Samson, Stud. Mycol. 70: 38. 2011. MycoBank MB563130.  

*Type*: *Penicillium stolkiae* D.B. Scott, Mycopathol. Mycol. Appl. 36: 8. 1968.  

*Diagnosis*: *Phylogeny*: Section *Stolkia* belongs to subgen. *Aspergilloides* and is phylogenetically related sister to sect. *Lanata-Divaricata*. *Morphology & physiology*: Colonies restricted or growing moderately fast; conidial colour *en masse* variable (dark green, greyish green, grey, blue green); conidiophores monoverticillate (*P*. *alogum*, *P*. *boreae*, *P*. *canariense*, *P*. *donkii*, *P*. *pullum*, *P*. *stolkiae*, *P*. *subarcticum*) or biverticillate (*P*. *boreae*, *P*. *canariense*), smooth, with some brown pigmentation; conidia globose, smooth or (finely) roughened; growth at 37 °C absent. *Sexual morph* not observed in culture, or present (*P*. *stolkiae*), eupenicillium-type, homothallic, orange-brown; ascospores ellipsoidal, with two well-defined longitudinal ridges, convex conspicuously roughened; sclerotia not produced. Section description based on Pitt, 1980, Peterson and Sigler, 2002, Houbraken and Samson, 2011 and Visagie *et al.* (2016b).  

*Included species*: *Penicillium alogum*, *P*. *boreae*, *P*. *canariense*, *P*. *donkii*, *P*. *pullum*, *P*. *stolkiae*, *P*. *subarcticum*.  

*Extrolites*: The are no chemotaxonomic data available for species in sect. *Stolkia*.  

*Notes*: See Houbraken & Samson (2011). A series subdivision of the section is not proposed and therefore ser. *Stolkia* is only informally introduced here (see Table 5).  

**Section *Thysanophora*** Houbraken & Samson, Stud. Mycol. 70: 33. 2011. MycoBank MB563126.  

*Type*: *Sclerotium glaucoalbidum* Desm., Ann. Sci. Nat. Bot. ser. 3, 16: 329. 1851. MycoBank MB212120 (= *Penicillium glaucoalbidum*).  

*Diagnosis*: *Phylogeny*: Section *Thysanophora* belongs to subgenus *Aspergilloides* and is phylogenetically sister to sect. *Ochrosalmonea*. *Morphology & physiology*: Colonies growing restrictedly; conidial colour *en masse* dark brown; conidiophores mono-, bi- or terverticillate, often with secondary, subapical formation of a penicillius (resulting in a chain of sympodial penicilli), dark brown pigmented, smooth or distinctly ornamented; conidia subglobose, ellipsoidal, fusiform or ovoidal, hyaline or in shades or brown, smooth or rough-walled. *Sexual morph* unknown; sclerotia pale brown, dark brown. Species are often associated with leaves of various trees (pine needles). Section description based on Ellis, 1971, Mercado-Sierra et al., 1998 and Houbraken & Samson (2011).  

*Included species*: *Penicillium asymmetricum*∗, *P*. *coniferophilum*∗, *P*. *glaucoalbidum*∗, *P*. *hennebertii*, *P*. *longisporum*∗, *P*. *melanostipe*∗, *P*. *taiwanense*∗, *P*. *taxi* (∗ not included in Fig. 18, no sequence data available for these species).  

*Extrolites*: There are no data for secondary metabolites in section *Thysanophora*.  

*Notes*: A modern taxonomic study of this section is lacking and needed, and we therefore follow Houbraken & Samson (2011). A series subdivision of the section is not proposed and therefore ser. *Thysanophora* is only informally introduced here (Table 5).  

**Section *Torulomyces*** (Delitsch) Stolk & Samson, Adv. Pen. Asp. Syst.: 169. 1986 [1985]. MycoBank MB832720.  

*Type*: *Penicillium lagena* (Delitsch) Stolk & Samson, Stud. Mycol. 23: 100. 1983.  

*Diagnosis*: *Phylogeny*: Section *Torulomyces* belongs to subgen. *Aspergilloides* and is phylogenetically sister to sect. *Crypta*. *Morphology & physiology*: Colonies restricted; conidial colour *en masse* greyish green, greenish white; conidiophores as solitary phialides, smooth; conidia globose or subglobose, occasionally broadly ellipsoidal (*P*. *catalonicum*, *P*. *variratense*), rough-walled; growth at 37 °C generally absent, present in *P*. *aeris*, *P*. *cantabricum* and *P*. *riverlandense*. *Sexual morph* generally not observed in culture, present in *P*. *wollemiicola*, eupenicillium-type, homothallic, colourless; ascospores ellipsoidal with two widely separated ridges, convex smooth-walled; sclerotia generally absent. Section description based on Visagie *et al.* (2016a).  

*Included species*: *Penicillium aeris*, *P*. *austricola*, *P*. *cantabricum*, *P*. *catalonicum*, *P*. *lagena*, *P*. *marthae-christenseniae*, *P*. *oregonense*, *P*. *parviverrucosum*∗, *P*. *porphyreum*, *P*. *riverlandense*, *P*. *tubakianum*, *P*. *variratense*, *P*. *williamettense*, *P*. *wisconsinense*, *P*. *wollemiicola* (∗ not included in Fig. 18, no sequence data available for this species).  

*Extrolites*: There are no chemotaxonomic data available for section *Torulomyces*.  

*Notes*: Visagie *et al.* (2016a) studied the taxonomy of sect. *Torulomyces* and accepted 18 species. *Penicillium cryptum* (sect. *Crypta*), *P*. *lassenii* (sect. *Lasseniorum*), *P*. *laeve* and *P*. *ovatum* (sect. *Exilicaulis*) are transferred here to other sections, and *P*. *parviverrucosum* is tentatively accepted. By excluding *P*. *cryptum*, *P*. *lassenii*, *P*. *laeve* and *P*. *ovatum*, sect. *Torulomyces* becomes more homogenous and all species produce conidiophores with solitary phialides and rough-walled conidia. A series subdivision of the section is not proposed and therefore ser. *Torulomyces* is only informally introduced here (Table 5).  

***Penicillium* subgen. *Penicillium*** [autonym]. MycoBank MB92187.

*Synonym*: *Penicillium* subgen. *Eupenicillium* Dierckx, Ann. Soc. Sci. Bruxelles 25: 86. 1901.  

*Type*: *Penicillium expansum* Link, Mag. Ges. Naturf. Freunde Berlin 3: 16. 1809.  

*Description*: See Frisvad & Samson (2004b) (extrolites, morphology), Houbraken & Samson (2011) (morphology, phylogeny), Houbraken *et al.* (2016).  

**Section *Brevicompacta*** Thom, The Penicillia: 157, 289. 1930. MycoBank MB834006.

*Synonym*: *Penicillium* sect. *Coronata* Pitt, The Genus Penicillium: 392. 1980 [1979].  

*Type*: *Penicillium brevicompactum* Dierckx, Ann. Soc. Sci. Bruxelles 25: 88. 1901.  

*Description*: See Frisvad & Samson (2004b) (as sect. “*Coronata*”; extrolites, morphology), Houbraken & Samson (2011) (morphology, phylogeny).  

Series ***Brevicompacta*** Houbraken & Frisvad, ***ser*. *nov***. MycoBank MB834482.  

*Etymology*: Named after the type species of the series, *Penicillium brevicompactum*.  

*Type*: *Penicillium brevicompactum* Dierckx, Ann. Soc. Sci. Bruxelles 25: 88. 1901.  

*Diagnosis*: *Phylogeny*: Series *Brevicompacta* belongs to subgen. *Penicillium*, sect. *Brevicompacta* and is phylogenetically most closely related to ser. *Olsoniorum*. *Morphology & physiology*: Colonies restricted, colony texture velvety; conidiophores terverticillate, sometimes biverticillate or multiramulate, smooth-walled, wide; conidia subglobose or ellipsoidal, smooth or finely roughened. *Sexual morph* unknown; sclerotia present in *P*. *neocrassum*, brown. Series description based on Frisvad & Samson (2004b) and Serra & Peterson (2007).  

*Included species*: *Penicillium bialowiezense*, *P*. *brevicompactum*, *P*. *fennelliae*, *P*. *kongii*, *P*. *neocrassum*.  

*Extrolites*: The Raistrick phenols and mycophenolic acid are only found in ser. *Brevicompacta* within sect. *Brevicompacta*. Botryodiploidin is found in one species in ser. *Brevicompacta* (*P*. *kongii*). Xanthoepocin is produced by three out of five species in ser. *Brevicompacta* and by all species in ser. *Olsoniorum*. The breviones have been found in two species in ser. *Brevicompacta*, one species in ser. *Spathulata* and one species in ser. *Olsoniorum*. Quinolactacin is produced by one species in ser. *Brevicompacta* (*P*. *bialowiezense*) and one species in ser. *Spathulata*.  

Series ***Buchwaldiorum*** Houbraken & Frisvad, ***ser*. *nov*.** MycoBank MB833972  

*Etymology*: Named after the type species of the series, *Penicillium buchwaldii*.  

*Type*: *Penicillium buchwaldii* Frisvad & Samson, FEMS Microbiol. Lett. 339: 86. 2013.  

**Fig. 19:**
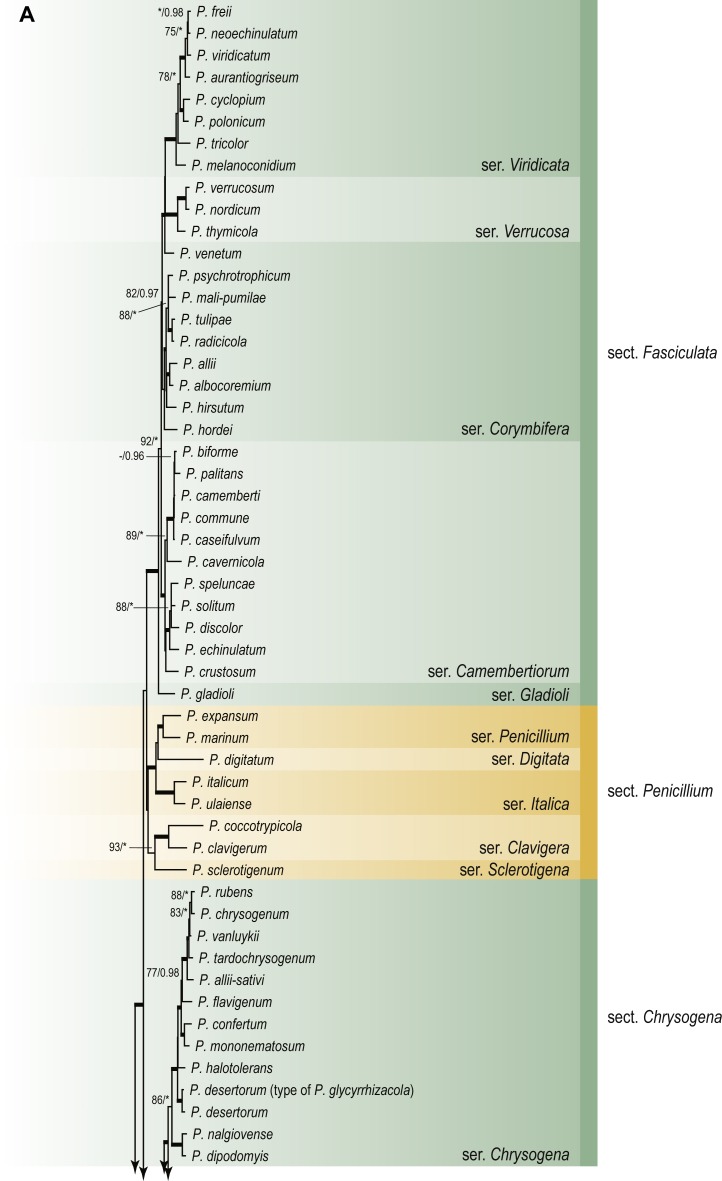

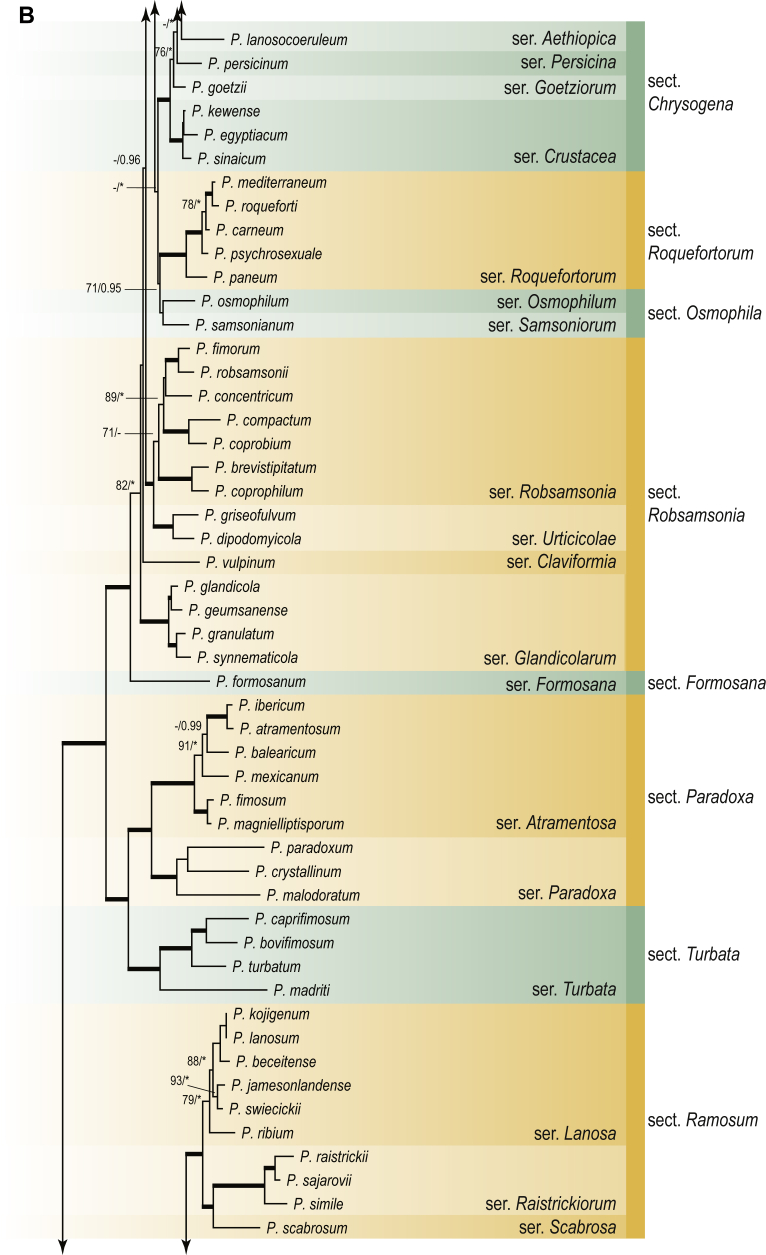

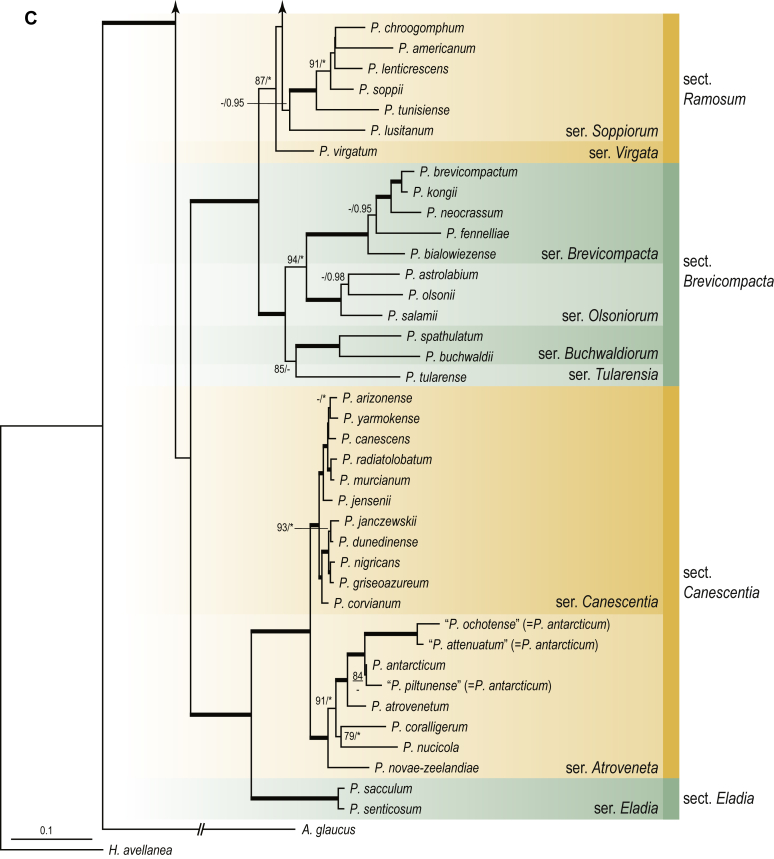
Combined phylogeny for *BenA*, *CaM* and *RPB2* data sets showing the phylogenetic relation of species, series and sections within *Penicillium* subgen. *Penicillium*. The BI posterior probability (pp) values and bootstrap percentages of the maximum likelihood (ML) analysis are presented at the nodes; fully supported branches are thickened. Values less than 70 % bootstrap support (ML) or less than 0.95 posterior probability (Bayesian analysis) are indicated with a hyphen or not shown. The bar indicates the number of substitutions per site. The phylogram is rooted with *Aspergillus glaucus* and *Hamigera avellanea*.

*Diagnosis*: *Phylogeny*: Series *Buchwaldiorum* belongs to subgen. *Penicillium*, sect. *Brevicompacta* and is phylogenetically most closely related to ser. *Tularensia*, though statistical support for this relationship is lacking (Fig. 19). *Morphology & physiology*: Colonies growing restrictedly (*P*. *spathulatum*) or moderately rapid (*P*. *buchwaldii*), colony texture velvety; conidiophores terverticillate, sometimes bi- or quarterverticillate, smooth to finely roughened; conidia globose or subglobose, rough-walled. *Sexual morph* unknown; sclerotia not observed in culture. Series description based on Frisvad *et al.* (2013a).

*Included species*: *Penicillium buchwaldii*, *P*. *spathulatum*.  

*Extrolites*: The extrolites only found in this series (in sect. *Brevicompacta*) include asperentins, communesins, paraherquamide, benzomalvins, cyclopenol and 2-chloro-6-[2’(S)-hydroxypropyl]-1,3,8-trihydroxy-anthraquinone (Frisvad *et al.* 2013a, Del Valle *et al.* 2016).  

Series ***Olsoniorum*** [as “*Olsonii*” Pitt, The Genus Penicillium (London): 392. 1980 [1979]. MycoBank MB833052.  

*Type*: *Penicillium olsonii* Bainier & Sartory, Ann. Mycol. 10: 398. 1912.  

*Diagnosis*: *Phylogeny*: Series *Olsoniorum* belongs to subgen. *Penicillium*, sect. *Brevicompacta* and is phylogenetically related to ser. *Brevicompacta*. *Morphology & physiology*: Colonies growing restrictedly or moderately rapid, colony texture velvety; conidiophores multiramulate, smooth-walled, long, wide; conidia ellipsoidal, smooth-walled or finely roughened. *Sexual morph* unknown; sclerotia observed in some strains of *P*. *olsonii* and *P*. *salami*, pale to light yellow or greyish brown. Series description based on Frisvad and Samson, 2004b, Serra and Peterson, 2007 and Perrone *et al.* (2015).  

*Included species*: *Penicillium astrolabium*, *P*. *olsonii*, *P*. *salamii*.  

*Extrolites*: Extrolites in ser. *Olsoniorum* include some that are only found in this series in sect. *Brevicompacta* (chrysogine, 2-(4-hydroxyphenyl)-2-oxo acetaldehyde oxime, meleagrin, siderin and verruculone (Perrone *et al.* 2015), showing a close relationship between those two series.  

Series ***Tularensia*** (Pitt) Houbraken & Frisvad, ***comb*. *nov*.** MycoBank MB833973.

*Basionym*: *Eupenicillium* ser. *Tularensia* Pitt, The Genus Penicillium: 98. 1980 [1979]. MycoBank MB832946.  

*Type*: *Eupenicillium tularense* Paden, Mycopathol. Mycol. Appl. 43: 262. 1971 (= *Penicillium tularense*).  

*Diagnosis*: *Phylogeny*: Series *Tularensia* belongs to subgen. *Penicillium*, sect. *Brevicompacta* and is phylogenetically related to ser. *Buchwaldiorum*, though statistical support is lacking (Fig. 19). *Morphology & physiology*: Colonies restricted, colony texture velvety; conidiophores predominant terverticillate, occasionally biverticillate, smooth-walled; conidia globose to broadly ellipsoidal, smooth. *Sexual morph* eupenicillium-type, homothallic, light brown to pale tan; ascospores ellipsoidal, with two longitudinal flanges and rugose walls. Series description based on Paden, 1971, Pitt, 1980, Stolk and Samson, 1983 and Frisvad *et al.* (2013a).  

*Included species*: *Penicillium tularense*.  

*Extrolites*: Apart from asperphenamate, shared with nearly all other species in sect. *Brevicompacta*, *P*. *tularense* produces a series of indoloterpenes unique for this species, including paxillins, and the related paspalines and janthitrems / shearinins, in addition to emindoles (Andersen and Frisvad, 2004, Frisvad et al., 2013a).  

*Notes*: Section *Brevicompacta* species produce terverticillate or multiramulate branched conidiophores that superficially resemble *Aspergillus* in the stereomicroscope. The position of *Penicillium fennelliae* in sect. (and ser.) *Brevicompacta* is remarkable, because this species produces penicillic acid, an extrolite not shared by any other species in the section. Furthermore, it does not produce asperphenamate, a compound shared by all other sect. *Brevicompacta* species (Frisvad *et al.* 2013a). The section is split in four series: *Brevicompacta*, *Buchwaldiorum*, *Olsoniorum* and *Tularensia*. Series *Brevicompacta* and *Olsoniorum* can be differentiated by their conidiophore branching pattern: the former has terverticillate conidiophores; the latter multiramulate ones. Species in ser. *Tularensia* produce a sexual morph and this is not observed in the series *Brevicompacta*, *Buchwaldiorum* and *Olsoniorum*. Sclerotia have been found in some strains of *P*. *olsonii* and *P*. *salami* (of ser. *Olsoniorum*).  

**Section *Canescentia*** Houbraken & Samson, Stud. Mycol. 70: 46. 2011. MycoBank MB563135.  

*Type*: *Penicillium canescens* Sopp, Skr. Vidensk.-Selsk. Christiana Math.-Nat. Kl. 11: 181. 1912.  

*Description*: See Houbraken & Samson (2011), this study (phylogeny); a modern overview including phenotypic data is not yet published.  

Series ***Atroveneta*** Stolk & Samson, Adv. Pen. Asp. Syst.: 175. 1986 [1985]. MycoBank MB832726.  

*Type*: *Penicillium atrovenetum* G. Sm., Trans. Brit. Mycol. Soc. 39: 112. 1956.  

*Diagnosis*: *Phylogeny*: Series *Atroveneta* belongs to subgen. *Penicillium*, sect. *Canescentia* and is phylogenetically related to ser. *Canescentia*. *Morphology & physiology*: Colonies growing moderately fast to fast, colony texture velutinous, exudate often formed on CYA; conidia *en masse* from dark green to turquoise; conidiophores biverticillate and appressed with an occasional additional branch, smooth- or rough-walled; conidia globose to ellipsoidal, smooth-walled or roughened; conidia 2.5–3.5 μm; no growth at 37 °C. *Sexual morph* unknown; sclerotia produced by some species.  

*Included species*: *Penicillium antarcticum*, *P*. *atrovenetum*, *P*. *coralligerum*, *P*. *novae-zeelandiae*, *P*. *nucicola*.  

*Extrolites*: Species in ser. *Atroveneta* produce different extrolites than those in ser. *Canescentia*: Asperentins, atrovenetins, benzomalvins, citreoviridin, cycloaspeptides, naphthalic anhydride, 3-nitropropionic acid, patulin, while chrysogines, communesins, penitrems and xanthoepocin are shared with some species in ser. *Canescentia* (Turner and Aldridge, 1971, Frisvad and Filtenborg, 1990, Shiono et al., 2008, Vansteelandt et al., 2012, Geiger et al., 2013, Takahashi et al., 2017).  

Series ***Canescentia*** Raper & Thom ex Pitt, The Genus Penicillium: 251. 1980 [1979]. MycoBank MB832960.  

*Type*: *Penicillium canescens* Sopp, Skr. Vidensk.-Selsk. Christiana Math.-Nat. Kl. 11: 181. 1912.  

*Diagnosis*: *Phylogeny*: Series *Canescentia* belongs to subgen. *Penicillium*, sect. *Canescentia* and is phylogenetically related to ser. *Atroveneta*. *Morphology & physiology*: Colonies growing moderately fast, colony texture floccose; conidiophores formed mostly on aerial mycelium, biverticillate with an occasional additional branch, divaricate, smooth- or rough-walled; conidia globose to ellipsoidal, smooth-walled or roughened; no growth at 37 °C. *Sexual morph* unknown. All species are soil- and litter-borne.  

*Included species*: *Penicillium arizonense*, *P*. *canescens*, *P*. *corvianum*, *P*. *dunedinense*, *P*. *griseoazureum*, *P*. *janczewskii*, *P*. *jensenii*, *P*. *murcianum*, *P*. *nigricans*, *P*. *radiatolobatum*, *P*. *yarmokense*.  

*Extrolites*: Acetylaranotins, austalides, curvulinic acids, fumagillins, griseofulvin, pseurotins, pyripyropens, trichodermamides and tryptoquivalines (Turner and Aldridge, 1971, Turner and Aldridge, 1983, Grijseels et al., 2016) are produced by some species in ser. *Canescentia*, but not by any species in ser. *Atroveneta*.  

*Notes*: Two phylogenetically related series are accepted in sect. *Canescentia*: *Canescentia* and *Atroveneta*. These series can be differentiated by different extrolite profiles and by their colony texture, which is typically floccose in species in ser. *Canescentia* and velutinous in ser. *Atroveneta*.  

**Section *Chrysogena*** Frisvad & Samson, Stud. Mycol. 49: 17. 2004. MycoBank MB700796.  

*Type*: *Penicillium chrysogenum* Thom, U.S.D.A. Bur. Animal Industr. Bull. 118: 58. 1910.  

*Description*: See Frisvad *et al.* (2004) (extrolites, morphology), Houbraken & Samson (2011) (phylogeny), Houbraken *et al.* (2012a) (morphology, phylogeny), Houbraken *et al.* (2016) (extrolites, morphology, phylogeny).  

Series ***Aethiopica*** Frisvad & Samson, Stud. Mycol. 49: 19. 2004. MycoBank MB700800.  

*Type*: *Penicillium aethiopicum* Frisvad, Mycologia 81: 848. 1989 (current name: *Penicillium lanosocoeruleum*).  

*Diagnosis*: *Phylogeny*: Series *Aethiopica* belongs to subgen. *Penicillium*, sect. *Chrysogena* and is phylogenetically most closely related to ser. *Chrysogena*. *Morphology & physiology*: Colonies growing moderately fast, colony texture fasciculate, copious production of exudate droplets on CYA; conidiophores terverticillate, rough-walled; conidia ellipsoidal, smooth-walled; growth at 37 °C, slower growth on CYA with 5 % NaCl (CYAS) than on CYA. *Sexual morph* unknown; sclerotia not observed in culture. Series description based on Frisvad & Samson (2004b) and Houbraken *et al.* (2012a).  

*Included species*: *Penicillium lanosocoeruleum*.  

*Extrolites*: The only species in ser. *Aethiopica*, *P*. *lanosocoeruleum* (= *P*. *aethiopicum*), produces the extrolites griseofulvin, tryptoquialanines and viridicatumtoxin, and these extrolites are not produced by other species of sect. *Chrysogena* (Frisvad & Samson 2004b).  

Series ***Chrysogena*** Raper & Thom ex Stolk & Samson, Adv. Pen. Asp. Syst.: 180. 1986 [1985]. MycoBank MB832730.

*Synonym*: *Penicillium* ser. *Mononematosa* Frisvad, Int. Mod. Meth. Pen. Asp. Clas. 269. 2000.  

*Type*: *Penicillium chrysogenum* Thom, U.S.D.A. Bur. Animal Industr. Bull. 118: 58. 1910.  

*Diagnosis*: *Phylogeny*: Series *Chrysogena* belongs to subgen. *Penicillium*, sect. *Chrysogena* and is phylogenetically most closely related to ser. *Aethiopica*. *Morphology & physiology*: Colonies growing moderately fast, colony texture velutinous, exudate droplets often present on CYA; conidiophores ter- to quarterverticillate, divergent, smooth; conidia globose to ellipsoidal, smooth; no or poor growth at 37 °C; growth on CYAS similar or more rapid than on CYA. *Sexual morph* generally not observed in culture, present under specific conditions in *P*. *rubens* (reported as *P*. *chrysogenum* by Böhm *et al.* (2013)), eupenicillium-type, heterothallic. Series description based on Frisvad & Samson (2004b) and Houbraken *et al.* (2012a).  

*Included species*: *Penicillium allii-sativi*, *P*. *chrysogenum*, *P*. *confertum*, *P*. *desertorum*, *P*. *dipodomyis*, *P*. *flavigenum*, *P*. *halotolerans*, *P*. *mononematosum*, *P*. *nalgiovense*, *P*. *rubens*, *P*. *tardochrysogenum*, *P*. *vanluykii*.  

*Extrolites*: Most species in ser. *Chrysogena* produce andrastins, chrysogine, meleagrin and roquefortine C, penicillin, sorbicillins (species with yellow exudate), while few species produce bioxanthracenes, citreoisocoumarins, chrysogenamide, cyclopaldic acids, dipodazin, haenamindole, nalgiovensins, penitrems, PR-toxins, secalonic acids, verrucosidins, verruculogen (and fumitremorgins), viriditoxins, xanthocillins, yanuthones Frisvad and Samson, 2004b, Frisvad et al., 2004 and Houbraken *et al.* (2012a).  

Series ***Goetziorum*** Houbraken & Frisvad, ***ser*. *nov*.** MycoBank MB833974.  

*Etymology*: Named after the type species of the series, *Penicillium goetzii*.  

*Type*: *Penicillium goetzii* J. Rogers *et al.*, Persoonia 29: 92. 2012.  

*Diagnosis*: *Phylogeny*: Series *Goetziorum* belongs to subgen. *Penicillium*, sect. *Chrysogena* and is phylogenetically sister to a clade containing series *Aethiopica*, *Chrysogena* and *Persicina*. *Morphology & physiology*: Colonies growing moderately fast, colony texture velvety to slightly floccose; conidiophores ter- to quarterverticillate, smooth-walled; conidia broadly ellipsoidal, smooth; no growth at 37 °C; growth on CYAS similar to CYA. *Sexual morph* eupenicillium-type, homothallic, creamish brown; ascospores ellipsoidal, with two distinct equatorial ridges, valves reticulate. Series description based on Houbraken *et al.* (2012a).  

*Included species*: *Penicillium goetzii*.  

*Extrolites*: Aflavinines, andrastin A, citreoisocoumarins (Wang et al., 1995, Houbraken et al., 2012a).  

Series ***Crustacea*** (Pitt) Houbraken & Frisvad, ***comb*. *nov*.** MycoBank MB833975.

*Basionym*: *Eupenicillium* ser. *Crustacea* Pitt, The Genus Penicillium: 139. 1980 [1979]. MycoBank MB832950.  

*Type*: *Eupenicillium crustaceum* F. Ludw., Lehrb. Nied. Krypt.: 263. 1892 (current name: *Penicillium kewense*).  

*Diagnosis*: *Phylogeny*: Series *Crustacea* belongs to subgen. *Penicillium*, sect. *Chrysogena* and is phylogenetically sister to a clade containing series *Aethiopica*, *Chrysogena*, *Goetziorum* and *Persicina*. *Morphology & physiology*: Colonies growing restrictedly or moderately fast, colony texture velvety or floccose; colony morphology dominated by sclerotia or ascomata, conidiophores predominantly terverticillate, sometimes bi- or quarterverticillate, appressed, smooth-walled; conidia globose to subglobose, smooth-walled. *Sexual morph* eupenicillium-type, homothallic, creamish, avellaneous or ochraceous; ascospores broadly ellipsoidal, with two distinct equatorial flanges, valves smooth or roughened. Series description based on Stolk & Samson (1983).  

*Included species*: *Penicillium egyptiacum*, *P*. *kewense*, *P*. *sinaicum*.  

*Extrolites*: Macrophorins, penicillic acid, secalonic acid D, xanthocillin X (Wang et al., 1995, Fujimoto et al., 2001).  

Series ***Persicina*** Frisvad & Samson, Stud. Mycol. 49: 19. 2004. MycoBank MB700803.  

*Type*: *Penicillium persicinum* L. Wang *et al.*, Antonie van Leeuwenhoek 86: 177. 2004.  

*Diagnosis*: *Phylogeny*: Series *Persicina* belongs to subgen. *Penicillium*, sect. *Chrysogena* and is phylogenetically sister to a clade containing series *Aethiopica* and *Chrysogena*. *Morphology & physiology*: Colonies growing moderately fast, colony texture velvety; conidiophores terverticillate, sometimes quarterverticillate; conidia ellipsoidal or cylindrical, smooth; growth at 37 °C, growth CYAS > CYA. *Sexual morph* unknown; sclerotia not observed in culture. Series description based on Frisvad & Samson (2004b).  

*Included species*: *Penicillium persicinum*.  

*Extrolites*: *Penicillium persicinum*, included in ser. *Persicina*, produces chrysogines, griseofulvins and roquefortines (Wang *et al.* 2004).  

*Notes*: The majority of sect. *Chrysogena* species are characterised by the formation of smooth-walled, divergent, ter- to quarterverticillate conidiophores with relatively small phialides. The species in this section are tolerant to salt and the majority produce colonies with a velvety texture. This section is divided in five series: *Aethiopica*, *Chrysogena*, *Crustacea*, *Goetziorum* and *Persicina*. All species in series *Goetziorum* and *Crustacea* produce a sexual morph in a homothallic manner and this is not observed in the other series. Penicillin production is restricted to ser. *Chrysogena*. Series *Aethiopica* and *Persicina* are monotypic series and both share the ability to produce griseofulvin, an extrolite not produced by other sect. *Chrysogena* species. Series *Aethiopica* is characterised by the production of fasciculate colonies and rough-walled conidiophores; ser. *Persicina* produces appressed, smooth-walled conidiophores and ellipsoidal to cylindrical conidia, a combination of features not observed in series *Chrysogena* and *Aethiopica* (Frisvad & Samson 2004b).  

**Section *Eladia*** (G. Smith) Stolk & Samson, Adv. Pen. Asp. Syst.: 171. 1986 [1985]. MycoBank MB832721.  

*Type*: *Penicillium sacculum* E. Dale, Ann. Mycol. 24: 137. 1926.  

*Diagnosis*: *Phylogeny*: Section *Eladia* belongs to subgen. *Penicillium* and is phylogenetically most closely related to sect. *Canescentia*. *Morphology & physiology*: Colonies restricted or growing moderately fast, colony texture velvety; conidiophores monoverticillate, occasionally biverticillate; conidia globose to subglobose, rough-walled, often with prominent connectives; phialides with a swollen base and very short neck, also born irregularly on the stipe. *Sexual morph* absent (*P*. *sacculum*) or present (*P*. *senticosum*), eupenicillium-type, homothallic, pale brown; ascospores ellipsoidal with two longitudinal flanges and echinulate walls. Section description based on Pitt, 1980, Stolk and Samson, 1983, Stolk and Samson, 1985 (morphology), Houbraken & Samson (2011) (morphology, phylogeny).  

*Included species*: *Penicillium sacculum*, *P*. *senticosum*.  

*Extrolites*: *Penicillium senticosum* produces curvularins and sorbicillins.  

*Notes*: This section is phylogenetically most closely related to sect. *Canescentia*. The production of ornamented conidia (often with prominent connectives), phialides with very short necks and the irregularly position of these phialides on the conidiophore stipes confirms the distinct phylogenetic position of this series in *Penicillium* (Pitt, 1980, Stolk and Samson, 1983, Stolk and Samson, 1985). Section *Eladia* is not subdivided in series and therefore ser. *Eladia* is informally introduced in this article.  

**Section *Fasciculata*** Thom, The Penicillia: 158, 374. 1930. MycoBank MB834008.

*Synonyms*: *Penicillium* sect. *Lanata-typica* Thom, The Penicillia: 157, 305. 1930.

*Penicillium* sect. *Viridicata* Frisvad & Samson, Stud. Mycol. 49: 27. 2004.  

*Type*: *Penicillium hirsutum* Dierckx, Ann. Soc. Sci. Bruxelles 25: 89. 1901.  

*Description*: See Frisvad & Samson (2004b) (as sect. *Viridicata*; morphology, extrolites), Houbraken & Samson (2011) (phylogeny), Houbraken *et al.* (2016) (morphology, phylogeny).  

Series ***Camembertiorum*** [as “*Camembertii*”] Raper & Thom ex Pitt, The Genus Penicillium: 358. 1980 [1979]. MycoBank MB833061.

*Synonym*: *Penicillium* series *Solita* Frisvad, Int. Mod. Meth. Pen. Asp. Clas.: 279. 2000.  

*Type*: *Penicillium camemberti* Thom, U.S.D.A. Bur. Animal Industr. Bull. 82: 33. 1906.  

*Diagnosis*: *Phylogeny*: Series *Camembertiorum* belongs to subgen. *Penicillium*, sect. *Fasciculata* and forms a clade with series *Corymbifera*, *Verrucosa* and *Viridicata*. *Morphology & physiology*: Colonies growing moderately fast, colony texture floccose, granular or crustose, but not distinctly fasciculate; conidiophores terverticillate, rough-walled; conidia ellipsoidal or globose to subglobose, smooth or rough-walled (*P*. *cavernicola*, *P*. *discolor*, *P*. *echinulatum, P*. *solitum*); good growth and (delayed) base production on creatine agar (CREA). *Sexual morph* unknown; sclerotia not observed in culture. Species typically occur on proteinaceous and lipid-containing foods. Series description based on Frisvad & Samson (2004b).  

*Included species*: *Penicillium biforme*, *P*. *camemberti*, *P*. *caseifulvum*, *P*. *cavernicola*, *P*. *commune*, *P*. *crustosum*, *P*. *discolor*, *P*. *echinulatum*, *P*. *palitans*, *P*. *solitum*, *P*. *speluncae*.  

*Extrolites*: Most of the species are able to produce viridicatins, cyclopenins and palitantin, while some species produce cyclopiazonic acids, cyclopaldic acid, territrems and arisugacins, compactins, penitrems, andrastins and atlantinones, asteltoxin, aurantiamine, dipodazine, glyanthrypine, roquefortine, clavatols, penitrems and terrestric acids (the latter four only by *P*. *crustosum*), burnettienes, geosmin, fumigaclavines, chaetoglobosins, daldinin D, and rugulovasines (Frisvad & Samson 2004b).  

Series ***Corymbifera*** Frisvad, Int. Mod. Meth. Pen. Asp. Clas.: 275. 2000. MycoBank MB700801.  

*Type*: *Penicillium hirsutum* var. *albocoremium* Frisvad, Mycologia 81: 856. 1990 (current name: *Penicillium albocoremium*).  

*Diagnosis*: *Phylogeny*: Series *Corymbifera* belongs to subgen. *Penicillium*, sect. *Fasciculata* and forms a clade with series *Camembertiorum*, *Verrucosa* and *Viridicata*; the position of *P*. *venetum* in this series is uncertain. *Morphology & physiology*: Colonies growing moderately fast, colony texture fasciculate or coremiform; most species produce coloured exudate droplets on CYA and MEA and grows moderately well on CREA, and acid is produced on CREA; conidiophores terverticillate, conspicuously roughened (except *P*. *hordei*, smooth to finely roughened), appressed; conidia globose or subglobose, smooth-walled (roughened in *P*. *hordei*). *Sexual morph* unknown; sclerotia not produced in culture. Species are mainly associated with flower bulbs and occasionally other plant roots. Series description based on Frisvad & Samson (2004b).  

*Included species*: *Penicillium albocoremium*, *P. allii*, *P*. *hirsutum*, *P*. *hordei*, *P*. *mali-pumilae*, *P*. *psychrotrophicum*, *P*. *radicicola*, *P*. *tulipae*, *P*. *venetum*.  

*Extrolites*: Roquefortine C and D are present in most species and meleagrin in some species. Cyclopenins and viridicatins are also present in several species in ser. *Corymbifera*. Atrovenetins and terrestic acids are also common in the series. Barceloneic acids, chrysogine, citrinin, compactins, daldinin D, ergometrine, fulvic acid, penicillic acid, penitrems are produced by some species in ser. *Corymbifera* (Overy and Frisvad, 2003, Frisvad and Samson, 2004b, Frisvad et al., 2004, Overy et al., 2005a, Overy et al., 2005b, Overy et al., 2005c, Hallas-Moller et al., 2018).  

Series ***Gladioli*** Raper & Thom ex Stolk & Samson, Adv. Pen. Asp. Syst.: 183. 1986 [1985]. MycoBank MB832732.  

*Type*: *Penicillium gladioli* L. McCulloch & Thom, Science 67: 217. 1928.  

*Diagnosis*: *Phylogeny*: Series *Gladioli* belongs to subgen. *Penicillium*, sect. *Fasciculata* and is phylogenetically basal to the other series of the section. *Morphology & physiology*: Colonies growing restrictedly to moderately rapid, colony texture floccose to slightly fasciculate; conidiophores terverticillate, occasionally more complexly branched, rough-walled; conidia subglobose, smooth-walled; growth on CREA moderate to good. *Sexual morph* not observed in culture; sclerotia produced, avellaneous, pale brown. Series *Gladioli* is associated with gladiolus bulb rot (Frisvad & Samson 2004b).  

*Included species*: *Penicillium gladioli*.  

*Extrolites*: The only species in the series produce atrovenetins, gladiolic acids, glyanthrypine and patulin (Frisvad & Samson 2004b).  

Series ***Verrucosa*** Frisvad, Int. Mod. Meth. Pen. Asp. Clas.: 269. 2000. MycoBank MB700806.  

*Type*: *Penicillium verrucosum* Dierckx, Ann. Soc. Sci. Bruxelles 25: 88. 1901.  

*Diagnosis*: *Phylogeny*: Series *Verrucosa* belongs to subgen. *Penicillium*, sect. *Fasciculata* and forms a clade with series *Camembertiorum*, *Corymbifera* and *Viridicata*. *Morphology & physiology*: Colonies restricted, colony texture velvety, floccose, or weakly fasciculate; conidiophores terverticillate; conidia globose to subglobose, rough-walled; weak growth on CREA, and no acid production; the three species in ser. *Verrucosa* grow well on nitrite-sucrose agar. *Sexual morph* unknown; sclerotia not observed in culture. Species are associated with stored cereal grains (*P*. *verrucosum*) and dried or salted meat products; they can also grow on cheese (Frisvad & Samson 2004b).  

*Included species*: *Penicillium nordicum*, *P*. *thymicola*, *P*. *verrucosum*.  

*Extrolites*: All species in *Verrucosa* produce verruculones and ochratoxins (Frisvad and Samson, 2004b, Nguyen et al., 2016). Some species produce alantrypinones, anacines, burnettienes, citrinin, daldinin D, fumiquinazolines, lumpidin, pyranonigrins, sclerotigenin, verrucins and viridic acid (Larsen et al., 1998, Ariza et al., 2001, Rahbæk et al., 2003, Frisvad and Samson, 2004b, Zhelifonova et al., 2012, Ma et al., 2017a, Tang et al., 2018).  

Series ***Viridicata*** Raper & Thom ex Pitt, The Genus Penicillium: 334. 1980 [1979]. MycoBank MB832967.  

*Type*: *Penicillium viridicatum* Westling, Ark. Bot. 11: 88. 1911.  

*Diagnosis*: *Phylogeny*: Series *Viridicata* belongs to subgen. *Penicillium*, sect. *Fasciculata* and forms a clade with series *Camembertiorum*, *Corymbifera* and *Verrucosa*. *Morphology & physiology*: Colonies growing moderately fast, colony texture granular or velvety; conidiophores terverticillate; rough-walled, growth on CREA weak to moderate, no base production; conidia ellipsoidal or globose to subglobose, smooth or very finely roughened (except echinulate in *P*. *neoechinulatum*). *Sexual morph* unknown; sclerotia not observed in culture. Many species in ser. *Viridicata* are associated with stored cereal grains. Series *Viridicata* is a typical example of a polythetic series regarding extrolites as no metabolite is shared among all species (Frisvad and Samson, 2004b, Hallas-Moller et al., 2018). Most species produce a dark brown halo on Raulin-Thom agar (Frisvad & Samson 2004b).  

*Included species*: *Penicillium aurantiogriseum*, *P*. *cyclopium*, *P*. *freii*, *P*. *melanoconidium*, *P*. *neoechinulatum*, *P*. *polonicum*, *P*. *tricolor*, *P*. *viridicatum*.  

*Extrolites*: Several species in ser. *Viridicata* produce 4-hydroxy-3,6-dimethyl-2H-pyran-2-one, penicillic acids, cyclopenins / viridicatins, verrucofortines / fructigenines / puberulines, verrucosidins, xanthomegnins, while rather few species produce anacine, aspermytin A / peaurantiogriseols, aspterric acid, asteltoxin, auranthine, aurantiomides, aurantiamine / viridamine, brevianamide A, 4-(9-hydroxy-10-butynoxy)benzoic acid / penipratynolenes, chrysogine, moniliformin, penicyrones, penitrem A, pseurotins, puberulonic acid, terrestric acids, viridic acid (Frisvad and Samson, 2004b, Ma et al., 2015, Bu et al., 2016, Hallas-Moller et al., 2018, Hamed et al., 2019).  

*Notes*: Species in sect. *Fasciculata* are able to grow well at 15 °C and low water activities, and they produce rough-walled conidiophores. This section is phylogenetically most closely related to sect. *Penicillium* (Fig. 19, Steenwyk *et al.* 2019). Section *Fasciculata* is divided in five series: *Camembertiorum*, *Corymbifera*, *Verrucosa*, *Viridicata* and *Gladioli*. The former four series are phylogenetically very closely related, and ser. *Gladioli* takes a sister position to those (Fig. 19). We tentatively place *P*. *venetum* in ser. *Corymbifera*. This species shares features with taxa in ser. *Corymbifera* but is phylogenetically closer to series *Viridicata* and *Verrucosa*. *Penicillium cavernicola*, *P*. *discolor*, *P*. *echinulatum* and *P*. *solitum* share the production of rough-walled conidia and these species were therefore previously placed in ser. *Solita* (Frisvad & Samson 2004b). Phylogenetic analysis shows that *P*. *cavernicola* is closely related to *P*. *camemberti* and related species. The other three species are phylogenetically related and these form a clade close to ser. *Camembertiorum*. The species previously classified in ser. *Solita* grow well on CREA, can use creatine as the sole N-source, and typically occur on proteinaceous and lipid-containing foods and these features are shared with ser. *Camembertiorum*. We therefore decided to expand ser. *Camembertiorum* with *P*. *cavernicola*, *P*. *discolor*, *P*. *echinulatum* and *P*. *solitum*, and treat ser. *Solita* (Frisvad & Samson 2004b) as a synonym of ser. *Camembertiorum*.  

**Section *Formosana*** Houbraken & Frisvad, ***sect*. *nov*.** MycoBank MB833976.  

*Etymology*: Named after the type species of the section, *Penicillium formosanum*.  

*Type*: *Penicillium formosanum* H.M. Hsieh *et al.*, Trans. Mycol. Soc. Rep. China 2: 159. 1987.  

*Diagnosis*: *Phylogeny*: Section *Formosana* belongs to subgen. *Penicillium* and is phylogenetically basal to sections *Chrysogena*, *Fasciculata*, *Osmophila*, *Penicillium*, *Samsoniorum* and *Roquefortorum*. *Morphology & physiology*: Colonies growing moderately fast, colony texture fasciculate, producing yellow synnemata; conidiophores terverticillate, smooth-walled; conidia subglobose to broadly ellipsoidal, smooth-walled; no growth at 37 °C, weak growth and no acid production on CREA. *Sexual morph* unknown; sclerotia not observed in culture. Series description on Hsieh *et al.* (1987) and Frisvad & Samson (2004b).  

*Included species*: *Penicillium formosanum*.  

*Extrolites*: Asteltoxin and patulin (Frisvad & Samson 2004b).  

*Notes*: *Penicillium formosanum* is the only species in sect. and ser. *Formosana*. This species forms a unique lineage in subgen. *Penicillium* and therefore treated as a separate section and series (Fig. 2, Fig. 19). *Penicillium formosanum* produces yellow synnemata on MEA and oatmeal agar (Frisvad & Samson 2004b), a feature not observed in species of related sections. A series classification is lacking in sect. and ser. *Formosana* is therefore only informally introduced in this article.  

**Section *Osmophila*** Houbraken & Frisvad, Persoonia 36: 309. 2016. MycoBank MB815869.  

*Type*: *Penicillium osmophilum* Stolk & Veenb.-Rijks, Antonie van Leeuwenhoek 40: 1. 1974.  

*Description*: See Houbraken *et al.* (2016) (morphology, phylogeny).  

Series ***Osmophila*** Houbraken & Frisvad, ***ser*. *nov*.** MycoBank MB833977.  

*Etymology*: Named after the type species of the series, *Penicillium osmophilum*.  

*Type*: *Penicillium osmophilum* Stolk & Veenb.-Rijks, Antonie van Leeuwenhoek 40: 1. 1974.  

*Diagnosis*: *Phylogeny*: Series *Osmophila* belongs to subgen. *Penicillium*, sect. *Osmophila* and is phylogenetically most closely related to ser. *Samsoniorum*. *Morphology & physiology*: Colonies restricted, colony texture floccose; conidiophores bi- and terverticillate, smooth-walled; conidia broadly ellipsoidal or subglobose, smooth-walled; osmophilic. *Sexual morph* eupenicillium-type, homothallic, avellaneous; ascospores ellipsoidal, with two equatorial flanges, valves rough-walled. Series description based on Pitt (1980) and Houbraken *et al.* (2016).  

*Included species*: *Penicillium osmophilum*.  

*Extrolites*: Andrastin A and meleagrin.  

Series ***Samsoniorum*** Houbraken & Frisvad, ***ser*. *nov*.** MycoBank MB833978.  

*Etymology*: Named after R.A. Samson, a Dutch mycologist studying *Penicillium* taxonomy.  

*Type*: *Penicillium samsonianum* L. Wang *et al.*, Persoonia 36: 313. 2016.  

*Diagnosis*: *Phylogeny*: Series *Samsoniorum* belongs to subgen. *Penicillium*, sect. *Osmophila* and is most closely related to ser. *Osmophila*. *Morphology & physiology*: Colonies restricted, colony texture lanose; conidiophores terverticillate, occasionally bi- or quarterverticillate, smooth-walled; conidia globose, smooth-walled; poor growth and no acid production on CREA, psychrotrophic. *Sexual morph* unknown; sclerotia not produced in culture. Series description based on Houbraken *et al.* (2016).  

*Included species*: *Penicillium samsonianum*.  

*Extrolites*: Berkbenzofuranthioester, dimethylphthalides (3,5-dimethyl-6-hydroxyphthalide and 3,5-dimethyl-6-methoxyphthalide), haenamindole, mycophenolic acid, patulin, penitrems, penitremones, phomfuranone, phomopsolides, phompyrone, roquefortine C, sclerotigenin (Stierle et al., 1997, Stierle et al., 2014 (strain misidentified as *P*. *clavigerum*); (Houbraken *et al.* 2016).  

*Notes*: Section *Osmophila* was introduced to accommodate *P*. *osmophilum* and *P*. *samsonianum*. These two species are phylogenetically related in our three-gene phylogenetic analysis (Fig. 19), confirming the results of Houbraken *et al.* (2016). In contrast, these species did not form a well-supported clade in our nine-gene phylogenetic analysis (Fig. 2). The two species included in sect. *Osmophila* are phenotypically different and share a few characters (Houbraken *et al.* 2016). We therefore decided to treat these as separate series (*Osmophila* and *Samsoniorum*).  

**Section *Paradoxa*** Houbraken & Samson, Stud. Mycol. 70: 43. 2011. MycoBank MB563134.  

*Type*: *Aspergillus paradoxus* Fennell & Raper, Mycologia 47: 69. 1955.  

*Description*: See Houbraken & Samson (2011) (morphology, phylogeny), Kocsubé *et al.* (2016) (phylogeny).  

Series ***Atramentosa*** Houbraken & Frisvad, ***ser*. *nov*.** MycoBank MB833979.  

*Etymology*: Named after the type species of the series, *Penicillium atramentosum*.  

*Type*: *Penicillium atramentosum* Thom, U.S.D.A. Bur. Animal Industr. Bull. 118: 65. 1910.  

*Diagnosis*: *Phylogeny*: Series *Atramentosa* belongs to subgen. *Penicillium* sect. *Paradoxa* and is phylogenetically most closely related to ser. *Paradoxa*. *Morphology & physiology*: Colonies growing moderately fast, brown reverse colour on CYA and YES, colony texture velvety; conidiophores predominantly terverticillate, sometimes bi- or quarterverticillate, thin, smooth-walled, good growth on CREA, but no acid production; conidia globose to subglobose or (broadly) ellipsoidal, smooth-walled. *Sexual morph* unknown. Series description based on Frisvad and Samson, 2004b, Visagie et al., 2014b and Guevara-Suarez *et al.* (2020).  

*Included species*: *Penicillium atramentosum*, *P*. *balearicum*, *P*. *fimosum*, *P*. *ibericum*, *P*. *magnielliptisporum*, *P*. *mexicanum*.  

*Extrolites*: Andrastin A, haenamindole, meleagrin, oxaline, roquefortine C and D, rugulovasine A and B.  

Series ***Paradoxa*** Houbraken & Frisvad, ***ser*. *nov*.** MycoBank MB833980.  

*Etymology*: Named after the type species of the series, *Aspergillus paradoxus*.  

*Type*: *Aspergillus paradoxus* Fennell & Raper, Mycologia 47: 69. 1955 (current name: *Penicillium paradoxum*).  

*Diagnosis*: *Phylogeny*: Series *Paradoxa* belongs to subgen. *Penicillium*, sect. *Paradoxa* is phylogenetically most closely related to ser. *Atramentosa*. *Morphology & physiology*: Colonies growing moderately fast or fast; conidiophores aspergillus-like, smooth-walled or roughened; biseriate, occasionally “triseriate”; conidia globose, subglobose or (broadly) ellipsoidal, smooth-walled or roughened. *Sexual morph* not observed in culture (*P*. *crystallinum*, *P*. *malodoratum*) or present (*P*. *paradoxum*), eupenicillium-type, homothallic, buff to vinaceous buff; ascospores ellipsoidal, with two equatorial flanges, valves smooth-walled; sclerotia present (*P*. *malodoratus*), buff to brown coloured. Series *Paradoxa* species produce an unpleasant smell and the species are mostly found on dung. Series description based on Raper and Fennell, 1965, Sarbhoy and Elphick, 1968 and Samson *et al.* (2014).  

*Included species*: *Penicillium crystallinum*, *P*. *malodoratum*, *P*. *paradoxum*.  

*Extrolites*: Andrastin A, chrysogine, chrysophanic acid, pachybasin, meleagrin, oxaline, sorbicillins (Frisvad and Samson, 2004b, Visagie et al., 2014b). Andrastin A, chrysogine, meleagrin and sorbicillins are shared with several species in ser. *Chrysogena*.  

*Notes*: Section *Paradoxa* is phylogenetically related to sect. *Turbata* (Fig. 2, Fig. 19). Both sections include species that are associated with dung (Tuthill and Frisvad, 2002, Samson et al., 2014, Guevara-Suarez et al., 2020). Two series are recognised in sect. *Paradoxa*: *Atramentosa* and *Paradoxa*. These series are phylogenetically and phenotypically distinct. Series *Paradoxa* includes species that have an aspergillus-type asexual morph, while ser. *Atramentosa* species produce typical penicillium-type conidiophores. *Penicillium atramentosum* was previously classified in ser. *Camembertiorum*, due to its ability to grow on creatine as sole N-source. This species differs from other series in this section by the production of smooth-walled conidiophores and its alkali-tolerant nature (Frisvad & Samson 2004b).  

**Section *Penicillium*** [autonym]. MycoBank MB549140.

*Synonyms*: *Penicillium* sect. *Bulliardium* Dierckx, Ann. Soc. Sci. Bruxelles 25: 85. 1901.

*Penicillium* sect. *Cylindrosporum* Pitt, The Genus Penicillium: 381. 1980 [1979].

*Penicillium* sect. *Digitata* Frisvad & Samson, Stud. Mycol. 49: 26. 2004.  

*Type*: *Penicillium expansum* Link, Mag. Ges. Naturf. Freunde Berlin 3: 16. 1809.  

*Description*: See Houbraken & Samson (2011) (phylogeny), Houbraken *et al.* (2016) (morphology, phylogeny).  

Series ***Clavigera*** Houbraken & Frisvad, ***ser*. *nov*.** MycoBank MB833981.  

*Etymology*: Named after the type species of the series, *Penicillium clavigerum*.  

*Type*: *Penicillium clavigerum* Demelius, Verh. Zool.-Bot. Ges. Wien 72: 74. 1923.  

*Diagnosis*: *Phylogeny*: Series *Clavigera* belongs to subgen. *Penicillium*, sect. *Penicillium* and is phylogenetically most closely related to ser. *Sclerotigena*. *Morphology & physiology*: Colonies restricted, colony texture coremiform, indeterminate synnemata (synnemata covered over nearly the entire length with conidiophores); conidiophores terverticillate, appressed, smooth-walled; conidia ellipsoidal, smooth-walled; poor growth on CYA supplemented with 5 % NaCl (CYAS). *Sexual morph* unknown; sclerotia not observed in culture. Series description based on Frisvad & Samson (2004b) and Crous *et al.* (2014).  

*Included species*: *Penicillium clavigerum*, *P*. *coccotrypicola*.  

*Extrolites*: Ascladiol, asperfuran, bioxanthracenes, cyclopiazonic acids, glandicolin A, isofumigaclavine A, norlichexanthone, patulin, penitrems, Raistrick phenols, roquefortin C, sclerotigenin, TAN-1612, viomellein, vioxanthin, xanthomegnin are all produced by *P*. *clavigerum* (Frisvad & Samson 2004b).  

Series ***Digitata*** Raper & Thom ex Stolk & Samson, Adv. Pen. Asp. Syst.: 183. 1986 [1985]. MycoBank MB832731.  

*Type*: *Penicillium digitatum* (Pers.) Sacc., Fungi. Italica Autogr. Delin: tab. 894. 1881.  

*Diagnosis*: *Phylogeny*: Series *Digitata* belongs to subgen. *Penicillium*, sect. *Penicillium* and is phylogenetically related to series *Italica* and *Penicillium*; but the relationship between these three series remains unresolved. *Morphology & physiology*: Colonies spreading, colony texture velvety, conidial colour *en masse* olive; conidiophores terverticillate, appressed, smooth-walled; conidia ellipsoidal or cylindrical, large, measuring 6–9(–14) μm in length, smooth-walled; no growth on Czapek agar, poor growth and no acid production on CREA. *Sexual morph* unknown; sclerotia not observed in culture. Causing rot in citrus (Frisvad & Samson 2004b).  

*Included species*: *Penicillium digitatum*.  

*Extrolites*: *Penicillium digitatum* produces tryptoquialanines and tryptoquialanones (Ariza et al., 2002, Frisvad and Samson, 2004b).  

Series ***Italica*** Fassatiová, Acta Univ. Carol., Biol. 12: 324. 1977. MycoBank MB832970.

*Synonym*: *Penicillium* series *Italica* Raper & Thom ex Pitt, The Genus Penicillium: 381. 1980 [1979].  

*Type*: *Penicillium italicum* Wehmer, Hedwigia 33: 211. 1894.  

*Diagnosis*: *Phylogeny*: Series *Italica* belongs to subgen. *Penicillium*, sect. *Penicillium* and is phylogenetically related to series *Digitata* and *Penicillium*; but the relationship between these three series remains unresolved. *Morphology & physiology*: Colonies restricted (*P*. *ulaiense*) or fast (*P*. *italicum*), colony texture velvety or fasciculate; conidiophores terverticillate, appressed, smooth-walled; conidia ellipsoidal or cylindrical, smooth-walled. *Sexual morph* not observed in culture; sclerotia can be present, colourless to light brown. Causing rot in citrus fruits. The extrolite deoxybrevianamide E is produced by both species in series *Italica* (Frisvad & Samson 2004b).  

*Included species*: *Penicillium italicum*, *P*. *ulaiense*.  

*Extrolites*: Italicic acids, PI-3, PI-4, dehydrofulvic acid, deoxybrevianamides, formylxanthocillin X, verrucolones including 5,6-dihydro-4-methoxy-2H-pyran-2-one, 4-methoxy-6-n-propenyl-2-pyrone, 5-hydroxymethylfuroic acid. Of these, *P*. *ulaiense* has only been shown to produce the deoxybrevanamides (deoxybrevianamide E, 12,13-dehydrodeoxybrevianamide E, 10,20-dehydro[12,13-dehydropropyl]-2-(1’,1’-dimethylallyltryptophanyl)diketo-piperazine) (Arai et al., 1989b, Frisvad and Samson, 2004b).  

Series ***Penicillium*** [autonym]. MycoBank MB549141.

*Synonym*: *Penicillium* ser. *Expansa* Raper & Thom ex Fassatiová, Acta Univ. Carol., Biol. 12: 324. 1977.  

*Type*: *Penicillium expansum* Link, Mag. Ges. Naturf. Freunde Berlin 3: 16. 1809.  

*Diagnosis*: *Phylogeny*: Series *Penicillium* belongs to subgen. *Penicillium*, sect. *Penicillium* and is phylogenetically related to series *Digitata* and *Italica*; but the relationship between these three series remains unresolved. *Morphology & physiology*: Colonies growing restrictedly or moderately rapid, colony texture velvety to fasciculate; conidiophores terverticillate, divergent, smooth; conidia globose, subglobose or ellipsoidal, smooth; good growth and acid production (and base production) on CREA. *Sexual morph* unknown; sclerotia not observed in culture.  

*Included species*: *Penicillium expansum*, *P*. *marinum*.  

*Extrolites*: Andrastins, aurantioclavine∗, chaetoglobosins, citrinin, communesins∗, expansolides, 3,5-dimethyl-6-hydroxyphthalide and 3,5-dimethyl-6-methoxyphthalide, patulin∗, penochalasins, penostatins, pyrrolocins∗, and roquefortine C∗ are produced by ser. *Penicillium* species (Andersen et al., 2004, Frisvad and Samson, 2004b). The extrolites marked with an asterisk are produced by both species in ser. *Penicillium*.  

Series ***Sclerotigena*** Houbraken & Frisvad, ***ser*. *nov*.** MycoBank MB833982.  

*Etymology*: Named after the type species of the series, *Penicillium sclerotigenum*.  

*Type*: *Penicillium sclerotigenum* W. Yamam., Sci. Rep. Hyogo Univ. Agric. 1: 69. 1955.  

*Diagnosis*: *Phylogeny*: Series *Sclerotigena* belongs to subgen. *Penicillium*, sect. *Penicillium* and is phylogenetically most closely related to ser. *Clavigera*. *Morphology & physiology*: Colonies spreading, colony texture velvety; conidiophores bi- and terverticillate, appressed, smooth; conidia ellipsoidal, smooth. *Sexual morph* not observed in culture; sclerotia orange-brown. Causing rot in yam tubers (Frisvad & Samson 2004b).  

*Included species*: *Penicillium sclerotigenum*.  

*Extrolites*: The extrolites griseofulvins, gregatins, patulin and sclerotigenin are produced by the sole species in ser. *Sclerotigena*.  

*Notes*: The species belonging to sect. *Penicillium* have a strongly fasciculate or synnematous colony texture, produce smooth-walled conidiophore stipes and conidia. Section *Penicillium* is poorly supported (ML < 70 % BS; BI < 0.95 pp) (Fig. 19), and a similar result was obtained by Houbraken *et al.* (2016). Five series are accepted in the phenotypically diverse sect. *Penicillium*: *Clavigera*, *Digitata*, *Italica*, *Penicillium* and *Sclerotigena*. Series *Digitata*, *Italica* and *Penicillium* are phylogenetically related and form a well-supported clade (Fig. 19); series *Clavigerum* and *Sclerotigena* form a related sister clade, but statistical support is lacking. Series *Clavigerum* and *Sclerotigena* are related but differ phenotypically. Series *Sclerotigena* species grow rapidly and form sclerotia, while ser. *Clavigera* species grow restrictedly and do not produce sclerotia. Series *Italica* and *Digitata* include species causing rot of citrus fruits, but these series are phenotypically distinct: ser. *Italica* species produce blue-green conidia, while olive-green conidia are produced in ser. *Digitata*.  

**Section *Ramosum*** Stolk & Samson, Adv. Pen. Asp. Syst.: 179. 1986 [1985]. MycoBank MB832722.  

*Type*: *Penicillium lanosum* Westling, Ark. Bot. 11: 97. 1911.  

*Description*: See Stolk & Samson (1985) (morphology), Houbraken & Samson (2011) (phylogeny); a modern taxonomic overview of the section using phenotypic data is lacking.  

Series ***Lanosa*** Stolk & Samson, Adv. Pen. Asp. Syst.: 180. 1986 [1985]. MycoBank MB834032.  

*Type*: *Penicillium lanosum* Westling, Ark. Bot. 11: 97. 1911.  

*Diagnosis*: *Phylogeny*: Series *Lanosa* belongs to subgen. *Penicillium*, sect. *Ramosum* and is phylogenetically most closely related to series *Raistrickiorum* and *Scabrosa*. *Morphology & physiology*: Colonies restricted, colony texture floccose; conidiophores biverticillate, twice biverticillate, or terverticillate, stipes smooth-walled or finely roughened; conidia globose to subglobose, smooth-, or rough-walled. *Sexual morph* unknown; sclerotia not observed in culture. All species are psychrotolerant.  

*Included species*: *Penicillium beceitense*, *P*. *jamesonlandense*, *P*. *kojigenum*, *P*. *lanosum*, *P*. *ribium*, *P*. *swiecickii*.  

*Extrolites*: Asperfuran, chrysogine, compactins, cycloaspeptides, griseofulvins,2-(4-hydroxyphenyl)-2-oxoacetaldehyde oxime, kojic acid, penicillic acid, pseurotins, psychrophilins, pyripyropenes, sclerotigenin, tryptoquivalines and viridicatumtoxin are produced by ser. *Lanosa* species (Frisvad *et al.* 2006).  

Series ***Raistrickiorum*** Houbraken & Frisvad, ***ser*. *nov*.** MycoBank MB833983.  

*Etymology*: Named after the type species of the series, *Penicillium raistrickii*.  

*Type*: *Penicillium raistrickii* G. Sm., Trans. Brit. Mycol. Soc. 18: 90. 1933.  

*Diagnosis*: *Phylogeny*: Series *Raistrickiorum* belongs to subgen. *Penicillium*, sect. *Ramosum* and is phylogenetically most closely related to ser. *Scabrosa*. *Morphology & physiology*: Colonies spreading, colony texture velutinous; conidiophores bi- or terverticillate, stipes rough-walled, inflated at the apex; conidia globose, smooth-walled; no growth at 37 °C, poor growth on CREA. *Sexual morph* not observed in culture; sclerotia produced in *P*. *raistrickii* and *P*. *simile*.  

*Included species*: *Penicillium raistrickii*, *P*. *sajarovii*, *P*. *simile*.  

*Extrolites*: Atrovenetins, chrysogine, griseofulvins, penicillic acid, peniciketals, peniciraistrins, pestafolide and desmethylcandidusins are produced by ser. *Raistrickiorum* species (Belofsky et al., 1998, Ma et al., 2012, Ma et al., 2017b, Liu et al., 2014a, Liu et al., 2018a, Li et al., 2019b).  

Series ***Scabrosa*** Houbraken & Frisvad, ***ser*. *nov*.** MycoBank MB833984.  

*Etymology*: Named after the type species of the series, *Penicillium scabrosum*.  

*Type*: *Penicillium scabrosum* Frisvad, Samson & Stolk, Persoonia 14: 177. 1990.  

*Diagnosis*: *Phylogeny*: Series *Scabrosa* belongs to subgen. *Penicillium*, sect. *Ramosum* and is phylogenetically most closely related to ser. *Raistrickiorum*. *Morphology & physiology*: Colonies growing restrictedly to moderately rapid, colony texture velvety; conidiophores biverticillate, sometimes with additional branch, rough-walled; conidia globose to subglobose, rough-walled. *Sexual morph* unknown; sclerotia not observed in culture.  

*Included species*: *Penicillium scabrosum*.  

*Extrolites*: The only species in ser. *Scabrosa* produces aurantiamine, asperpentyns, cyclopenins & viridicatins, gliovictins, penigequinolones, peniprequinolones, fumagillin, penicillic acid, pseurotins (Frisvad and Filtenborg, 1990, Larsen et al., 1998).  

Series ***Soppiorum*** Houbraken & Frisvad, ***ser*. *nov*.** MycoBank MB833985.  

*Etymology*: Named after the type species of the series, *Penicillium soppii*.  

*Type*: *Penicillium soppii* K.W. Zaleski, Bull. Int. Acad. Polon. Sci., Cl. Sci. Math., Sér. B., Sci. Nat. 1927: 476. 1927.  

*Diagnosis*: *Phylogeny*: Series *Soppiorum* belongs to subgen. *Penicillium*, sect. *Ramosum* and is phylogenetically sister to a clade with series *Lanosa*, *Raistrickiorum* and *Scabrosa*, though statistical support for this relationship is lacking; we place *P*. *lusitanum* in ser. *Soppiorum*, though statistical support for this position is poor (<70 % BS, 0.95 pp). *Morphology & physiology*: Colonies growing slow to moderately fast, colony texture velutinous, conidiophores on aerial hyphae or from substrate; conidiophores 100–500 μm in length; smooth-walled or finely roughened, conidia globose, subglobose or broadly ellipsoidal, smooth-walled to finely roughened; no growth at 30 °C and 37 °C, poor growth and no acid production on CREA. *Sexual morph* not observed in culture; sclerotia produced by *P*. *soppii*.  

*Included species*: *Penicillium americanum*, *P*. *chroogomphum*, *P*. *lenticrescens*, *P*. *lusitanum*, *P*. *soppii*, *P*. *tunisiense*.  

*Extrolites*: Asperentins, asperphenamate, benzomalvins, cycloaspeptides, fumagillin, griseofulvin, pseurotins and terrein are extrolites found in *P*. *soppii* (Frisvad *et al.* 2006).  

Series ***Virgata*** Houbraken & Frisvad, ***ser*. *nov*.** MycoBank MB833986.  

*Etymology*: Named after the type species of the series, *Penicillium virgatum*.  

*Type*: *Penicillium virgatum* Nirenberg & Kwasna, Mycol. Res. 109: 977. 2005.  

*Diagnosis*: *Phylogeny*: Series *Virgata* belongs to subgen. *Penicillium*, sect. *Ramosum* and is phylogenetically basal to the other series of sect. *Ramosum*. *Morphology & physiology*: Colonies restricted, colony texture velvety to floccose; conidiophores biverticillate, with additional branches, smooth; conidia globose to subglobose, rough-walled; poor growth and no acid production on CREA. *Sexual morph* unknown; sclerotia not observed in culture. Series description based on Kwaśna & Nirenberg (2005).  

*Included species*: *Penicillium virgatum*.  

*Extrolites*: Andrastin A.  

*Notes*: Five well-supported lineages, representing series *Lanosa*, *Raistrickiorum*, *Scabrosa*, *Soppiorum* and *Virgata* are recognised in sect. *Ramosum*. This series classification is mainly based on the phylogenetic analysis presented in Fig. 19. Series *Scabrosa* is phylogenetically most close to ser. *Raistrickiorum* and these two series from a clade together with ser. *Lanosa*. Series *Soppiorum* is sister to these series, but the statistical support for this absent (ML = <75 % BS) or low (BI < 0.95 pp). Series *Virgata* takes a basal position in sect. *Ramosum*. A taxonomic study of sect. *Ramosum* is not yet performed and might give more data supporting the proposed series classification.  

**Section *Robsamsonia*** Houbraken & Frisvad, Persoonia 36: 309. 2016. MycoBank MB815870.  

*Type*: *Penicillium robsamsonii* Frisvad & Houbraken, Persoonia 36: 313. 2016.  

*Description*: See Houbraken *et al.* (2016) (morphology, phylogeny).  

Series ***Claviformia*** Raper & Thom ex Stolk *et al.*, Mod. Conc. Pen. Asp. Clas.: 132. 1990. MycoBank MB834533.  

*Type*: *Penicillium vulpinum* (Cooke & Massee) Seifert & Samson, Adv. Pen. Asp. Syst.: 144. 1986 [1985].  

*Diagnosis*: *Phylogeny*: Series *Claviformia* is here tentatively classified in subgen. *Penicilium*, sect. *Robsamsonia*; statistical support for this classification is lacking (Fig. 19, see also Notes on series in section below). *Morphology & physiology*: Colonies restricted, sometimes growing moderately fast, having capitulate synnemata in concentric zones; conidiophores terverticillate, sinoid, smooth-walled; conidia ellipsoidal, smooth. *Sexual morph* unknown; sclerotia not observed in culture.  

*Included species*: *Penicillium vulpinum*.  

*Extrolites*: Andrastins, (-)-3-butyl-7-hydroxyphthalides, cyclopenins, cyclopiamine, meleagrin, 2-methoxymethyl-3-pentylphenol, 2-methyl-hydroquinone, oxalicin, oxalin, pachybasin, patulin, pintulin, roquefortine C & D viridicatins (Frisvad & Samson 2004b).  

Series ***Glandicolarum*** Houbraken & Frisvad, ***ser*. *nov*.** MycoBank MB834600.  

*Etymology*: Named after the type species of the series, *Coremium glandicola*.  

*Type*: *Coremium glandicola* Oudem. Ned. Kruidk. Arch. 2: 918. 1903 (current name: *Penicillium glandicola*).  

*Diagnosis*: *Phylogeny*: Series *Glandicolarum* belongs to subgen. *Penicillium*, sect. *Robsamsonia* and is phylogenetically basal to the other series of sect. *Robsamsonia* (except ser. *Claviformia*). *Morphology & physiology*: *Colonies* restricted; colony texture strongly fasciculate; conidiophores terverticillate, tuberculate stipes; conidia ellipsoidal, smooth-walled; good growth and acid production on CREA. *Sexual morph* unknown; sclerotia not observed in culture. *Penicillium glandicola* has been found on acorns and in dung.  

*Included species*: *Penicillium geumsanense*, *P*. *glandicola*, *P*. *granulatum*, *P*. *synnematicola*.  

*Extrolites*: Andrastin A, asperfuran, meleagrins, roquefortine C & D, patulidin, penitrems are produced by *P*. *glandicola* (Frisvad *et al.* 2004).  

Series ***Robsamsonia*** Houbraken & Frisvad, ***ser*. *nov*.** MycoBank MB833987.  

*Etymology*: Named after the type species of the series, *Penicillium robsamsonii*.  

*Type*: *Penicillium robsamsonii* Frisvad & Houbraken, Persoonia 36: 313. 2016.  

*Diagnosis*: *Phylogeny*: Series *Robsamsonia* belongs to subgen. *Penicillium*, sect. *Robsamsonia* and is phylogenetically related to ser. *Urticicola*, though statistical support is poor (Fig. 19). *Morphology & physiology*: Colonies restricted, sometimes growing moderately fast, colony texture fasciculate or synnematous; conidiophores terverticillate, smooth- or rough-walled; conidia ellipsoidal, smooth-walled; good growth on CREA and no or some acid production. *Sexual morph* unknown.  

*Included species*: *Penicillium brevistipitatum*, *P*. *compactum*, *P*. *concentricum*, *P*. *coprobium*, *P*. *coprophilum*, *P*. *fimorum*, *P*. *robsamsonii*.  

*Extrolites*: Alternariol, andrastins, chaetoglobosins, citreoisocoumarins, clavatols, cyclopiamins, griseofulvins, meleagrin, palitantin, patulin, patulodin, pyripyropenes, quinolactacins, roquefortine C, xanthoepocin. Pyripyropens are shared by ser. *Robsamsonia* species (Houbraken *et al.* 2016).  

Series ***Urticicola*** Fassatiová, Acta Univ. Carol., Biol. 12: 324. 1977. MycoBank MB834534.  

*Type*: *Penicillium urticae* Bainier, Bull. Soc. Mycol. France 23: 15. 1907. (current name: *Penicillium griseofulvum*).  

*Diagnosis*: *Phylogeny*: Series *Urticicola* belongs to subgen. *Penicillium*, sect. *Robsamsonia* and is phylogenetically related to ser. *Robsamsonia*, though statistical support is poor (Fig. 19). *Morphology & physiology*: Colonies growing restrictedly or moderately rapid, colony texture weakly fasciculate; conidiophores bi-, ter-, or quarterverticillate, divergent, smooth-walled; phialides very short, less than 6 μm in length; conidia broadly ellipsoidal, smooth-walled. Poor growth and no acid production on CREA. *Sexual morph* unknown.  

*Included species*: *Penicillium dipodomyicola*, *P*. *griseofulvum*.  

*Extrolites*: Cyclopiamins, cyclopiazonic acids∗, fulvic acids, griseofulvins∗, mycelianamide, patulin∗, patulodin, roquefortine C. The extrolites with an asterisk are produced by both species in ser. *Urticicola*.  

*Notes*: Species in sect. *Robsamsonia* grow moderately fast on CYA incubated at 25 °C and no or slow growth is present on CYA incubated at 30 °C. Section *Robsamsonia* includes four series: *Claviformia*, *Glandicolarum*, *Robsamsonia* and *Urticicola*. Series *Robsamsonia* and *Urticicola* are phylogenetically related, but the relationship of the series *Claviformia* (*Penicillium vulpinum*) and *Glandicolarum* (*P*. *glandicola*) is unresolved. In Houbraken *et al.* (2016), series *Claviformia* and *Glandicolarum* taxa took a basal position in sect. *Robsamsonia*, but lacked support in the ML and Bayesian analysis. A similar result was obtained in this study (Fig. 19). *Penicillium vulpinum* was included in the genome scale phylogenetic study of Steenwyk *et al.* (2019) and based on this analysis, this species is sister to *P*. *coprophilum* (ser. *Robsamsonia*) and *P*. *griseofulvum* (ser. *Urticicola*). Series *Claviformia*, *Glandicolarum* and *Robsamsonia* include species that occur on dung, and ser. *Urticicola* occurs mainly on dry cereals and seeds. Furthermore, series *Claviformia* and *Glandicolarum* include species that produce synnematous structures and these structures are also produced by certain members of ser. *Robsamsonia* (*e.g*., *P*. *coprophilum*). Based on similarity in habitat (dung) and morphology and the phylogenetic relationship using genome data, we decided to accommodate series *Claviformia* and *Glandicolarum* in sect. *Robsamsonia*. Stolk *et al.* (1990) listed *Penicillium* ser. *Granulata* Raper & Thom ex Fassatiová in their classification of the terverticillate Penicillia (type *P*. *granulatum*). We could not find Fassatiová’s original description of this series and regard this series as doubtful. Series *Glandicolarum* is introduced for *P*. *glandicola* and related species.  

**Section *Roquefortorum*** [as “*Roqueforti*”] Frisvad & Samson, Stud. Mycol. 49: 16. 2004. MycoBank MB701527.  

*Description*: See series *Roquefortorum* below.  

Series ***Roquefortorum*** [as “*Roqueforti*”] Raper & Thom ex Frisvad, Int. Mod. Meth. Pen. Asp. Clas.: 277. 2000. MycoBank MB701528.  

*Type*: *Penicillium roqueforti* Thom, U.S.D.A. Bur. Animal Industr. Bull. 82: 35. 1906.  

*Diagnosis*: *Phylogeny*: Section *Roquefortorum* belongs to subgen. *Penicillium*; the phylogenetic relationship with other sections is unresolved (Fig. 2, Fig. 19). *Morphology & physiology*: Colonies spreading; texture velutinous; conidiophores terverticillate, with tuberculate stipes; conidia globose to subglobose, smooth-walled; growth at low pH (*e.g*., media containing 0.5 % acetic acid), high alcohol concentrations and elevated CO_2_ levels. Good growth but no acid production on CREA. *Sexual morph* not observed in culture (*P*. *carneum*, *P*. *paneum*) or present, eupenicillium-type, homothallic (*P*. *psychrosexuale*) or heterothallic (*P*. *roqueforti*, Ropars *et al.* (2014)), pale orange-brown. Section description based on Frisvad & Samson (2004b) (extrolites, phylogeny), Houbraken *et al.* (2010a) (morphology, extrolites), Houbraken & Samson (2011) (phylogeny).  

*Included species*: *Penicillium carneum*, *P*. *mediterraneum*, *P*. *paneum*, *P*. *psychrosexuale*, *P*. *roqueforti*.  

*Extrolites*: Andrastins, botryodiplidin, chryzothiazoles, citreoisocoumarins, isofumigaclavines, marcfortines, mycophenolic acid, patulin, penipacids, penipanoids, PR-toxins, roquefortine C & D∗ (Frisvad and Samson, 2004b, Nielsen et al., 2005, Li et al., 2011, Li et al., 2013, Li et al., 2014, An et al., 2013 (*P*. *paneum* misidentified as *P*. *chrysogenum*), Xu *et al.* 2014; *P*. *paneum* misidentified as *P*. *oxalicum*).  

*Notes*: Even though ser. *Roquefortorum* is the sole series in the section, we accept this series because it was formally introduced (Frisvad *et al.* 2000). The phylogenetic relationship of this section with other sections is unresolved (Fig. 2, Fig. 19). The extrolites marcfortines, botryodiploidin and isofumigaclavine are produced by members of this series, and are not detected in the related sections *Chrysogena*, *Fasciculata*, *Penicillium* and *Robsamsonia* (Houbraken *et al.* 2016).  

**Section *Turbata*** Houbraken & Samson, Stud. Mycol. 70: 43. 2011. MycoBank MB563133.  

*Type*: *Penicillium turbatum* Westling, Ark. Bot. 11: 128. 1911.  

*Diagnosis*: *Phylogeny*: Section *Turbata* belongs to subgen. *Penicillium* and is phylogenetically related to sect. *Paradoxa*. *Morphology & physiology*: Colonies growing moderately fast, texture velutinous; conidiophores monoverticillate, (symmetrically biverticillate), or terverticillate; conidia globose, subglobose or ellipsoidal, smooth or rough-walled in older cultures. *Sexual morph* not observed in culture (*P*. *madriti*) or present (*P*. *bovifimosum*, *P*. *turbatum* (syn. *Eup*. *baarnense*)), eupenicillium-type, homothallic, in brown shades. Series description based on Houbraken & Samson (2011) (phylogeny).  

*Included species*: *Penicillium bovifimosum*, *P*. *caprifimosum*, *P*. *madriti*, *P*. *turbatum*.  

*Extrolites*: Species belonging to this section can produce penicillic acid and a fumagillin-like compound (Tuthill & Frisvad 2002).  

*Notes*: No series classification is proposed for sect. *Turbata*, and ser. *Turbata* is therefore only informally introduced in this article.

### New combinations and names for species in *Eurotiales*

Four new genera are introduced above (*Acidotalaromyces*, *Ascospirella*, *Evansstolkia* and *Pseudohamigera*). New combinations are made below for the species belonging to these new genera. Furthermore, new combinations (or names) are introduced for the following incorrectly classified species: *Byssochlamys verrucosa*, *Chaetosartorya stromatoides*, *Merimbla humicoloides*, *Penicillium arenicola*, *P*. *kabunicum*, *P*. *mirabile*, *P*. *moldavicum*, *P*. *resinae*, *Phialomyces striatus*, *Talaromyces brevicompactus* and *Thermoascus crustaceus* var. *verrucosus*. No *Paecilomyces* name was available for *Byssochlamys lagunculariae* and this is introduced below.  

***Acidotalaromyces lignorum*** (Stolk) Houbraken, Frisvad & Samson, ***comb*. *nov*.** MycoBank MB832555.

*Basionym*: *Penicillium lignorum* Stolk, Antonie van Leeuwenhoek 35: 264. 1969.  

*Notes*: Stolk (1969) classified this species in sect. *Biverticillata-Symmetrica* (subgen. *Biverticillium*). Many of the species previously belonging to subgen. *Biverticillium* were transferred to *Talaromyces* (Samson *et al.* 2011c). Phylogenetically, this species clearly does not belong to *Talaromyces sensu stricto* and is the sole species in the newly introduced genus *Acidotalaromyces*.  

***Ascospirella lutea*** (Zukal) Houbraken, Frisvad & Samson, ***comb*. *nov*.** MycoBank MB832556.

*Basionym*: *Penicillium luteum* Zukal, Sitzungsber. Kaiserl. Akad. Wiss. Wien, Math.-Naturwiss. Cl., Abt. 1, 98: 561. 1890.

*Synonyms*: *Gymnoascus luteus* (Zukal) Sacc., Syll. Fung. 11: 437. 1895.

*Talaromyces luteus* (Zukal) C.R. Benj., Mycologia 47: 681. 1955.

*Talaromyces luteus* (Zukal) Stolk & Samson, Stud. Mycol. 2: 23. 1972.  

*Notes*: Pitt (1980) treated *Tal*. *udagawae* as a synonym of *Ascospirella luteum* (= *Tal*. *luteus*). These species produce similarly ornamented ascospores, but are phylogenetically distinct (Yilmaz *et al.* 2014). *Ascospirella lutea* produces luteusins A-E (Fujimoto et al., 1990, Yoshida et al., 1996a, Yoshida et al., 1996b), which are different from the azaphilones produced by *Penicillium*, *Aspergillus* and *Talaromyces* (Gao *et al.* 2013).  

***Aspergillus chaetosartoryae*** Hubka, Kocsubé & Houbraken, ***nom*. *nov*.** MycoBank MB832557.

*Replaced synonym*: *Chaetosartorya stromatoides* B.J. Wiley & E.G. Simmons, Mycologia 65: 935. 1973, *non Aspergillus stromatoides* Raper & Fennell.  

*Etymology*: Referring to *Chaetosartorya*, the teleomorph genus in which this species was originally described.  

*Notes*: *Chaetosartorya stromatoides* was described as the sexual morph of *Aspergillus stromatoides*. This species was typified with QM 8944 (= CBS 265.73 = ATCC 24480 = IMI 171880 = NRRL 5501). Molecular analysis showed that *Aspergillus stromatoides* (IMI 123750^T^ = CBS 500.65 = NRRL 4519) and *Chaetosartorya stromatoides* are related, but distinct species (Peterson, 1995, Peterson, 2008). The name *Aspergillus stromatoides* is already occupied and therefore the name *Aspergillus* *chaetosartoryae* is introduced.  

***Evansstolkia leycettana*** (H.C. Evans & Stolk) Houbraken, Frisvad & Samson, ***comb*. *nov*.** MycoBank MB832558.

*Basionym*: *Talaromyces leycettanus* H.C. Evans & Stolk, Trans. Brit. Mycol. Soc. 56: 45. 1971.

*Synonyms*: *Penicillium leycettanum* H.C. Evans & Stolk, Trans. Brit. Mycol. Soc. 56: 45. 1971.

*Paecilomyces leycettanus* (H.C. Evans & Stolk) Stolk *et al.*, Stud. Mycol. 2: 51. 1972.  

*Note*: See above, under generic description of *Evansstolkia*.  

***Hamigera brevicompacta*** (H.Z. Kong) Houbraken, Frisvad & Samson, ***comb*. *nov*.** MycoBank MB832579.

*Basionym*: *Talaromyces brevicompactus* H.Z. Kong, Mycosystema 18: 9. 1999.  

*Notes*: The taxonomy of *Hamigera* has been subject of various studies (Stolk and Samson, 1971, Pitt, 1980, Peterson et al., 2010). The genus has been molecularly revised (Peterson *et al.* 2010); however, *Tal*. *brevicompactus* was not included. The original description mentioned that this species is phenotypically similar to *Hamigera avellanea* (reported as *Tal*. *avellaneus*), but differs in ascomatal initials, ascospore ornamentation and conidiophore branching (Kong 1999). Samson *et al.* (2011c) indicated that *Tal*. *brevicompactus* represents a distinct species in *Hamigera* and our phylogenetic analysis (Supplementary Fig. S2) supports this observation. The new combination *H*. *brevicompacta* is proposed here.  

***Paecilomyces lagunculariae*** (C. Ram) Houbraken, Frisvad & Samson, ***comb*. *nov*.** MycoBank MB832559.

*Basionym*: *Byssochlamys nivea* var. *lagunculariae* C. Ram, Nova Hedwigia 16: 311. 1968.

*Synonym*: *Byssochlamys lagunculariae* (C. Ram) Samson *et al.*, Persoonia 22: 18. 2009.  

*Notes*: Ram (1968) described *Byssochlamys nivea* var. *lagunculariae*, without the description of the *Paecilomyces* asexual morph. In a later study on the taxonomy of *Byssochlamys* and its *Paecilomyces* asexual morphs, Samson *et al.* (2009) elevated this variety to species level, but also did not formally describe the *Paecilomyces* morph. The type species of *Paecilomyces*, *Paec*. *variotii*, and the type species of *Byssochlamys*, *B*. *nivea*, were shown to be congeneric through molecular sequence analyses (Luangsa-Ard et al., 2004, Samson et al., 2009). Anticipating on the change to one scientific name for fungi (McNeill *et al.* 2012), Rossman *et al.* (2016) recommended the use of *Paecilomyces* over *Byssochlamys*, and we therefore formally introduce *Paec*. *lagunculariae* here.  

***Penicillago kabunica*** (Baghd.) Houbraken, Frisvad & Samson, ***comb*. *nov*.** MycoBank MB832560.

*Basionym*: *Penicillium kabunicum* Baghd., Novosti Sist. Nizsh. Rast. 5: 98. 1968.  

*Notes*: See under *Penicillago*. *Penicillium kabunicum* (and *P*. *moldavicum*) were treated by Ramírez (1982) in the “*P*. *brasilianum*-series” and Pitt (1980) treated this species as a synonym of *P*. *janthinellum*. These observations indicate a relationship with *Penicillium* sect. *Lanata-Divaricata*; however, phylogenetic analysis does not support this placement. *Penicillago kabunica* produces cycloaspeptide A and D.  

***Penicillago mirabilis*** (Beliakova & Milko) Houbraken, Frisvad & Samson, ***comb*. *nov*.** MycoBank MB832561.

*Basionym*: *Penicillium mirabile* Beliakova & Milko, Mikol. Fitopatol. 6: 145. 1972.  

*Notes*: See also above, under *Penicillago* (under genera). The identity of this species is controversial and needs further study. Samson *et al.* (2011c) and Yilmaz *et al.* (2014) showed in their phylogenetic analyses that *P*. *mirabile* belongs to *Talaromyces* sect. *Trachyspermi*. However, Samson *et al.* (2011c) had doubts about these results and they therefore did not combine this species in *Talaromyces*. Furthermore, they noted that the type strain is in poor condition. Two sequences obtained from IMI 167383 (ex-type of *P*. *mirabile*) (KC992257, *BenA*; KC962096, ITS) are present in GenBank and comparison of those sequences indicate that this strain is a *Penicillium corylophilum*. In the original description of *P*. *mirabile*, it is mentioned that the species produces echinulate conidia with conspicuous connectives. This feature does not fit with *P*. *corylophilum* and the sequences derived from IMI 167383 are incorrect. Pitt (1980) placed *P*. *mirabile* in *Penicillium* subgen. *Biverticillium* (nowadays *Talaromyces pro parte*). However, he also reported production of smooth-walled conidia and the strain he examined therefore also does not fit with the original description. Re-examination of CBS 624.72, the ex-type of *P*. *mirabile*, shows that this strain fits well with the description of Ramírez (1982). Similar to the observation of Ramírez (1982), deep brown to black coloured sclerotia were not observed in CBS 624.72. Phylogenetic analysis using *BenA*, *CaM*, ITS and *RPB2* sequences shows that this strain is related to *P*. *kabunicum*, *P*. *moldavicum* and *P*. *nodositatum* in the genus *Penicillago* (Fig. 6).  

***Penicillago moldavica*** (Milko & Beliakova) Houbraken, Frisvad & Samson, ***comb*. *nov*.** MycoBank MB832562.

*Basionym*: *Penicillium moldavicum* Milko & Beliakova, Novosti Sist. Nizsh. Rast. 4: 255. 1967.  

*Notes*: See above, under *Penicillago*, and in Guevara-Suarez *et al.* (2020). *Penicillago moldavica* produces alternatriol.  

***Penicillago nodositata*** (Valla) Guevara-Suarez *et al.*, Fungal Syst. Evol. 5: 64. 2020. MycoBank MB822074.

*Basionym*: *Penicillium nodositatum* Valla, Pl. & Soil 114: 146. 1989.  

*Notes*: Based on sequence data, *P*. *nodositatum* was tentatively placed in synonymy with *P*. *bilaiae* by Houbraken & Samson (2011). This conclusion was drawn based on incorrect accession numbers in the CBS culture collection (Visagie *et al.* 2013). Visagie *et al.* (2013) subsequently re-examined CBS 333.90, the ex-type of *P*. *nodositatum*, and reported that this strain is related to *P*. *kabunicum*, outside *Penicillium sensu stricto*. *Penicillago nodositata* is unique in that it forms myconodules in roots of *Alnus* trees (Valla *et al.* 1989). We detected an altersolanol, cycloaspeptide A and griseofulvin in this species.  

***Phialomyces arenicola*** (Chalab.) Houbraken, Frisvad & Samson, ***comb*. *nov*.** MycoBank MB832563.

*Basionym*: *Penicillium arenicola* Chalab., Not. Syst. Crypt. Inst. bot. Acad. Sci. USSR: 162. 1950.

*Synonym*: *Penicillium canadense* G. Sm., Trans. Brit. Mycol. Soc. 39: 113. 1956.  

*Notes*: *Merimbla humicoloides*, *P*. *arenicola* and *P*. *canadense* are phylogenetically related to *Phialomyces macrosporus*, the type species of *Phialomyces* (Fig. 1) (Misra & Talbot 1964). Based on a multigene phylogenetic analysis, *M*. *humicoloides* proved not to belong to the *Hamigera*-clade. To maintain a monophyletic genus, Peterson *et al.* (2010) decided to combine this species in the genus *Penicillium*, as *P*. *humicoloides*. On the other hand, they also noted a close relationship of this species with *P*. *arenicola* and *P*. *canadensis*, two species outside *Penicillium sensu stricto* (Houbraken & Samson 2011). The species belonging to the *Phialomyces* clade produce penicillium-like conidiophores and gold-brown conidia, a feature not observed in *Penicillium sensu stricto*. Based on phylogenetic data and morphology, we combine *Merimbla* (= *Penicillium*) *humicoloides*, and *P*. *arenicola* in *Phialomyces*. Pitt (1980) treated *P*. *canadense* as a synonym of *P*. *arenicola*. A phylogenetic analysis of six loci resolved *P*. *canadense* as a close relative of *P*. *arenicola* (Peterson *et al.* 2010) but since no phenotypic differences exist between these species we regard *Penicillium canadense* as a synonym of *Phialomyces arenicola*. *Phialomyces arenicola* has been reported to produce canadensolides (McCorkindale *et al.* 1968), chlorogentisylalcohol (McCorkindale *et al.* 1972) and asperphenamate (McCorkindale *et al.* 1978).  

***Phialomyces humicoloides*** (Bills & Heredia) Houbraken, Frisvad & Samson, ***comb*. *nov*.** MycoBank MB832564.

*Basionym*: *Merimbla humicoloides* Bills & Heredia, Mycol. Res.105: 1276. 2001.

*Synonym*: *Penicillium humicoloides* (Bills & Heredia) S.W. Peterson *et al.*, Mycologia 102: 858. 2010.  

*Note*: See *Ph*. *arenicola*.  

***Pseudohamigera striata*** (Raper & Fennell) Houbraken, Frisvad & Samson, ***comb*. *nov*.** MycoBank MB832565.

*Basionym*: *Penicillium striatum* Raper & Fennell, Mycologia 40: 521. 1948.

*Synonyms*: *Talaromyces striatus* (Raper & Fennell) C.R. Benj., Mycologia 47: 682. 1955.

*Hamigera striata* (Raper & Fennell) Stolk & Samson, Persoonia 6: 347. 1971.

*Byssochlamys striata* (Raper & Fennell) Arx, Mycotaxon 26: 120. 1986.

*Penicillium lineatum* Pitt, The Genus Penicillium: 485. 1980.

*Paecilomyces lineatus* (Pitt) Arx, Mycotaxon 26: 120. 1986.  

*Notes*: The taxonomic position of *Pseudohamigera striata* was discussed several times in history. The sexual morph of *Pseudohamigera striata* was previously classified in *Talaromyces*, *Hamigera* and *Byssochlamys* and the asexual morph in *Penicillium* and *Paecilomyces* (Raper and Fennell, 1948, Stolk and Samson, 1971, Pitt, 1980, von Arx, 1986), showing the difficulty in classifying this species. Phylogenetic data (Supplementary Fig. S1) show that this species is distinct from *Byssochlamys*, *Hamigera*, *Paecilomyces*, *Penicillium* and *Talaromyces*.  

***Talaromyces resinae*** (Z.T. Qi & H.Z. Kong) Houbraken & X.C. Wang, ***comb*. *nov*.** MycoBank MB833989.

*Basionym*: *Penicillium resinae* Z.T. Qi & H.Z. Kong, Acta Mycol. Sin. 1: 103. 1982.  

*Notes*: Based on an incorrect *RPB2* sequence, Houbraken *et al.* (2014b) considered *P*. *resinae* a synonym of *Penicillium purpurescens* (sect. *Aspergilloides*). *Penicillium purpurescens* predominantly produced simple, monoverticillate conidiophores, while *P*. *resinae* was originally described to predominantly produce symmetrical biverticillate conidiophores. Qi & Kong (1982) classified *P*. *resinae* in the *Penicillium funiculosum* series, near *Penicillium piceum*). Both *P*. *funiculosum* and *P*. *piceum* are currently classified in *Talaromyces* and it is likely that this species also belongs to this genus. The ex-type strain of *P*. *resinae* (CBS 324.83) was re-examined and new sequence data was generated. Comparison of these sequences show a relationship with *Tal*. *brasiliensis and Tal*. *subericola* in sect. *Trachyspermi* (Supplementary Fig. S3).  

***Talaromyces striatoconidius*** Houbraken, Frisvad & Samson, ***nom*. *nov*.** MycoBank MB832566.

*Replaced synonym*: *Phialomyces striatus* R.F. Castañeda & W. Gams, Mycotaxon 42: 239. 1991, *non Talaromyces striatus* (Raper & Fennell) C.R. Benj. 1955.  

*Etymology*: The epithet refers to the typical striate conidia produced by the species.  

*Notes*: This species was originally described as *Phialomyces striatus*. A connection with *Penicillium* was made and it was suggested that the species takes an intermediate position between *Phialomyces* and *Penicillium* (Castañeda & Gams 1991). Molecular data shows that the species belongs to *Talaromyces* sect. *Talaromyces* and it is phylogenetically most closely related to *Tal*. *galapagensis*, *Tal*. *indigoticus* and *Tal*. *rubicundus* (Supplementary Fig. S3). *Talaromyces striatoconidius* can be easily differentiated from these related species by its production of striate conidia, a rare feature for most other *Talaromyces* species. The name *Talaromyces striatus* (= *Pseudohamigera striata*) is already occupied and therefore the name *T*. *striatoconidius* is proposed.  

***Thermoascus verrucosus*** (Samson & Tansey) Houbraken, Frisvad & Samson, ***comb*. *nov*.** MycoBank MB832567.

*Basionym*: *Byssochlamys verrucosa* Samson & Tansey, Trans. Brit. Mycol. Soc. 65: 512. 1975.

*Synonym*: *Paecilomyces verrucosus* Samson & Tansey, Trans. Brit. Mycol. Soc. 65: 512. 1975.  

*Notes*: Samson & Tansey (1975) described *B*. *verrucosa* in *Byssochlamys* based on phenotypic characters. The conidial state of this species resembles *Paecilomyces fulvus* and both produce asci from croziers. However, based on an ITS phylogeny, Samson *et al.* (2009) showed that this species is related to *Thermoascus*. This observation is confirmed in our phylogenetic analysis (Supplementary Fig. S1) and we therefore combine this species in *Thermoascus*. Samson & Tansey (1975) already noted that *B*. *verrucosa* produced simple coiled initials, similar to those of *Tal*. *leycettanus* and *Tal*. *purpureus*, indicating a position outside *Byssochlamys*. *Thermoascus crustaceus* produces, like *Th*. *verrucosus*, a paecilomyces-type asexual morph and asci from croziers, and this also indicates a relationship with the genus *Thermoascus*.  

***Thermoascus yaguchii*** Houbraken, Frisvad & Samson, ***stat*. *et nom*. *nov*.** MycoBank MB833988.

*Replaced synonym*: *Thermoascus crustaceus* var. *verrucosus* Yaguchi, Someya & Udagawa, Mycoscience 36: 151. 1995, *non Thermoascus verrucosus* (Samson & Tansey) Houbraken, Frisvad & Samson (this study).  

*Etymology*: In honour of Takashi Yaguchi, a prominent taxonomist studying various genera of *Eurotiales* and one of the authors that described *Thermoascus crustaceus* var. *verrucosus*.

*Synonym*: *Coonemeria verrucosa* (Yaguchi *et al.*) Mouch., Cryptog. Mycol. 18: 32. 1997.  

*Notes*: *Therrnoascus crustaceus* var. *verrucosus* was introduced as a variety because of its verrucose ascospore ornamentation, in contrast to the echinulate ornamentation in *Th*. *crustaceus* var. *crustaceus*. Sequence data show that this variety represents a distinct species in *Thermoascus*. Comparison of partial *BenA* and *CaM* sequences of CBS 181.67 (a representative strain of *Th*. *crustaceus*) and PF-1160^T^ (= CBS 146343 = IFM 66000), the type of *Th*. *crustaceus* var. *verrucosus*, shows a homology of 95.6 and 96.9 %, respectively. The name *Thermoascus verrucosus* is already occupied and therefore the name *Thermoascus yaguchii* is proposed.

### Invalid and not accepted species described after 2013

Since the “2014 *Aspergillus*/*Penicillium*/*Talaromyces* lists”, around 300 new names have been described in *Penicillium*, *Aspergillus* and *Talaromyces*. Re-evaluation of these names showed that some of those recently described species were not new to science, incorrectly described or described in the wrong genus. An overview of the not accepted species described after 2013 is given below.  

***Aspergillus capsici*** (J.F.H. Beyma) Houbraken *et al.*, Stud. Mycol. 78: 154. 2014.  

*Notes*: The genus *Polypaecilum* was synonymised with *Aspergillus* and therefore *Polypaecilum capsici* was incorrectly combined in *Aspergillus*. The correct name of *Aspergillus capsici* (basionym *Scopulariopsis capsici* J.F.H. Beyma) is *Leuconeurospora capsici* (J.F.H. Beyma) Malloch *et al.*  

***Aspergillus chinensis*** Samson *et al.*, Stud. Mycol. 78: 155. 2014.  

*Notes*: The name *Aspergillus appendiculatus* was already occupied and therefore the new name *Aspergillus chinensis* was introduced for *Emericella appendiculata* (Samson *et al.* 2014). Phylogenetic and morphologic examination showed that *Aspergillus chinensis* is identical with *Aspergillus filifer*. We follow Matsuzawa et al., 2012, Hubka et al., 2016a and Chen *et al.* (2016a) in correcting the name *A*. *chinensis* to *Aspergillus filifer* Zalar *et al.*  

***Aspergillus cicutus*** P. Singh & P.J. Cotty, Int. J. Food Microbiol. 289: 148. 2018, *nom*. *inval*.  

*Notes*: This species is invalidly described: Arts 38.1 (without a description or diagnosis of the taxon), 40.1 (without designation of type specimen), F.5.1 (no identifier cited). None of the markers commonly used in *Aspergilllus* taxonomy (*BenA*, *CaM* or *RPB2*) were available for the representative isolates of *A*. *cicutus* (NRRL 66829, NRRL 66830, NRRL 66831) and only *niaD* (nitrate reductase) and *aflR* (aflatoxin pathway transcription factor) sequences were studied (Singh & Cotty 2019). This species could represent a novel species or it is a synonym of a sect. *Flavi* member (*e.g*., *Aspergillus austwickii*).  

***Aspergillus delacroxii*** Samson *et al.*, Stud. Mycol. 78: 155. 2014.  

*Notes*: From Hubka *et al.* (2016a): The name *Aspergillus echinulatus* was already occupied and therefore the name *Aspergillus delacroxii* was introduced for *Aspergillus nidulans* var. *echinulatus* Fennell & Raper. The epithet ‘‘delacroxii’’ was derived from the name of E.G. Delacroix and thus the correct Latin form is “delacroixii”. The spelling ‘‘delacroxii’’ could be treated as a correctable orthographical error [Art. 60.1; McNeill *et al.* (2012)], but the name *A*. *delacroixii* was validly proposed twice before, making the name *A*. *delacroxii* Samson *et al.* illegitimate. Even if the epithet ‘‘delacroxii’’ was grammatically correct, its similarity to the epithet “delacroixii” would likely cause confusion [(Art. 53.3; McNeill *et al.* (2012)]. We follow Hubka *et al.* (2016a) and the correct name for *A*. *nidulans* var. *echinulatus* is *Aspergillus spinulosporus* Hubka *et al.*  

***Aspergillus ferenczii*** (Varga & Samson) Samson *et al.*, Stud. Mycol. 78: 155. 2014.  

*Notes*: Samson *et al.* (2007a) described *Neosartorya ferenczii* as a new species in their monograph on *Aspergillus* sect. *Fumigati* and due to the introduction of the single name nomenclature, the combination in *Aspergillus* was subsequently made in 2014 (Samson *et al.* 2014). In their monograph, Samson *et al.* (2007a) were unable to obtain the ex-type cultures of *Neosartorya indohii*, *N. nishimurae*, *N*. *sublevispora*, *N*. *takakii*, and *N*. *tsurutae*, and these species were therefore treated as doubtful. However, comparison of the *BenA*, *CaM* and *RPB2* sequences of the ex-type strains on *A*. *ferenczii* (CBS 121594) and *A*. *sublevisporus* (CBS 128796 = IFM 53598) showed that these are similar (*BenA*, EF669833 *vs* AB488759: 100 %; *CaM*, EF669903 *vs* AB488767: 99.6 %; ITS, EF669977 *vs* MN431376: 100 %; *RPB2*, EF669764 *vs* MN969095: 99.8 %). *Aspergillus ferenczii* is regarded to be conspecific with *Aspergillus sublevisporus* Someya *et al.*  

***Aspergillus frequens*** Hubka *et al.*, Mycologia 107: 183. 2015.  

*Notes*: The type strains of *A*. *frequens* (NRRL 4578) and *A*. *micronesiensis* (CBS 138183) have highly similar *BenA*, *CaM* and *RPB2* sequences. Based on these data, Arzanlou *et al.* (2016) reduced *A*. *frequens* (Hubka *et al.* 2015) in synonymy with *A*. *micronesiensis* Visagie *et al.* (Visagie *et al.* 2014a).  

***Aspergillus korhogoensis*** A. Carvajal-Campos *et al.*, Toxins 9, 353: 11. 2017. *nom*. *inval*.  

*Notes*: An identifier issued by a recognised repository for *A*. *korhogoensis* was not cited in the protologue and this species is therefore invalidly described [Art. 42.1, McNeill *et al.* (2012)]. Frisvad *et al.* (2019) studied the taxonomy of *Aspergillus* sect. *Flavi* and based on their studied specimens, they introduced *Aspergillus cerealis*. Based on sequence similarity, *Aspergillus korhogoensis* is treated as a synonym of *A*. *cerealis* Houbraken *et al.*  

***Aspergillus latus*** (Thom & Raper) A.J. Chen *et al.*, Stud. Mycol. 84: 69. 2016.  

*Notes*: Chen *et al.* (2016a) treated *Aspergillus sublatus* as a synonym of *A*. *latus*. Even though *A*. *nidulans* var. *latus* Thom & Raper (1939) is the oldest name of this species, the new combination/status proposed by Chen *et al.* (2016a) cannot have priority over *A*. *sublatus* (oldest name in the species rank) (Hubka *et al.* 2016a). The correct name for this species is *Aspergillus sublatus* Y. Horie.  

***Aspergillus mangaliensis*** A. Nováková *et al.*, Mycologia 107: 187. 2015.  

*Notes*: Based on gene concordance and a high sequence homology, Arzanlou *et al.* (2016) treated *Aspergillus mangaliensis* as a synonym of *A*. *templicola* Visagie *et al.* Their conclusion is followed here.  

***Aspergillus occiafricanus*** P. Singh & P.J. Cotty, Int. J. Food Microbiol. 289: 148. 2018, *nom*. *inval*.  

*Notes*: This species was invalidly described (without an identifier, Art. F.5.1). NRRL A-11612 was designated as the type. The *BenA* (MK119746) and *CaM* (MK119712) sequences derived from the type strain have high homology (99.8 %) with the type of *A*. *aflatoxiformans* (CBS 143679). *Aspergillus occiafricanus* can therefore be considered an invalidly described synonym of *A*. *aflatoxiformans* Frisvad *et al.*  

***Aspergillus parafelis*** Sugui *et al.*, J. Clin. Microbiol. 52: 3709. 2014.  

*Notes*: Hubka *et al.* (2018a) re-evaluated the species boundaries in the *Aspergillus viridinutans* species complex using a large set of clinical and environmental strains. In their analyses, they showed that *A*. *parafelis* and *A*. *pseudofelis* are included in the genetically diverse *A*. *felis* lineage. This is in contrary to the results of Sugui *et al.* (2014); however, only two isolates of each species were included in their analysis and these isolates did not sufficiently cover the genetic diversity within *A*. *felis* (Hubka *et al.* 2018a).  

***Aspergillus pseudofelis*** Sugui *et al.*, J. Clin. Microbiol. 52: 3709. 2014.  

*Notes*: This species is a synonym of *A*. *felis* Barrs *et al.;* see *A*. *parafelis*.  

***Aspergillus similis*** (Y. Horie *et al.*) Samson *et al.*, Stud. Mycol. 78: 157. 2014.  

*Notes*: Samson *et al.* (2014) combined *Emericella similis* in *Aspergillus*. Chen *et al.* (2016a) noted that the ascospore morphology of the ex-type culture (CBS 293.93) is identical to that of *Aspergillus violaceus*. This close relationship is confirmed by sequence data and we follow the conclusion of Chen *et al.* (2016a) and treat *A*. *similis* as a synonym of *A*. *violaceus* Fennell & Raper.  

***Aspergillus texensis*** P. Singh *et al.*, Toxins 10, 513: 6. 2018.  

**Fig. 20:**
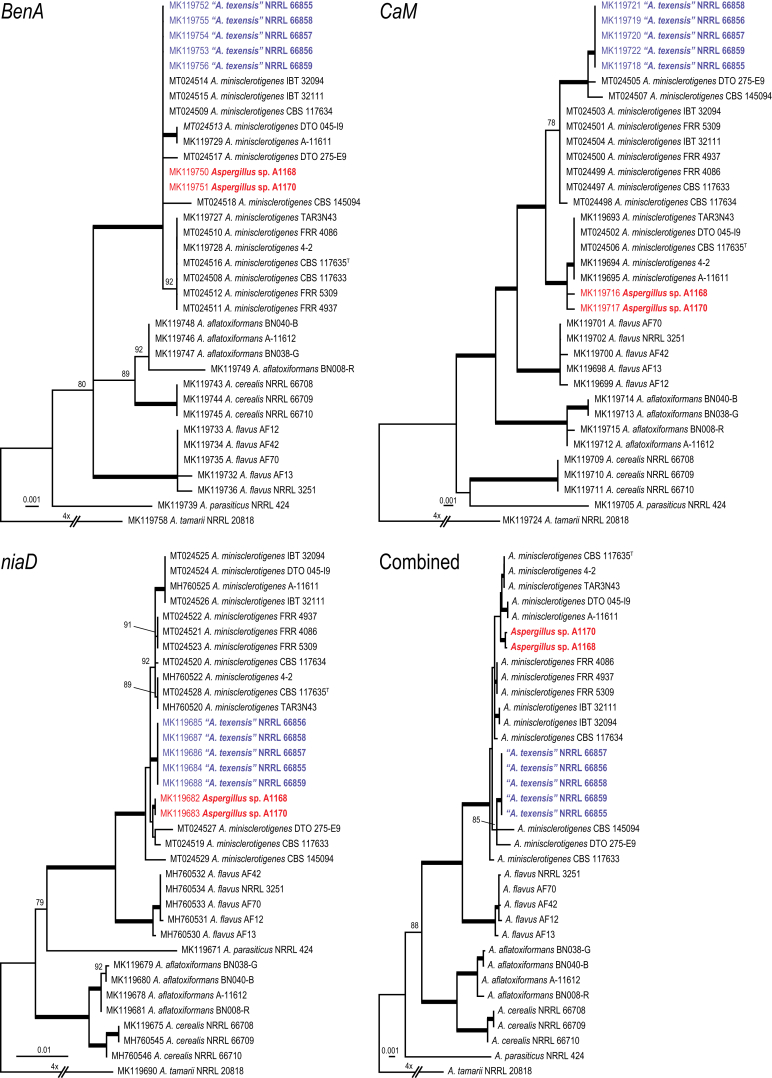
Phylogenetic trees based on single and a combined data set of *BenA*, *CaM* and *niaD* sequences showing the relationship between *Aspergillus texensis* and related species. The BI posterior probability (pp) values and bootstrap percentages of the maximum likelihood (ML) analysis are presented at the nodes; fully supported branches are thickened. Values less than 70 % bootstrap support (ML) or less than 0.95 posterior probability (Bayesian analysis) are indicated with a hyphen or not shown. The bar indicates the number of substitutions per site. The phylogram is rooted with *Aspergillus tamarii* NRRL 20818.

*Notes*: Molecular phylogenetic analyses of *Aspergillus flavus*-like isolates producing small-sized sclerotia (average sclerotium size < 400 μm) collected from across the United States resulted in the discovery of a novel aflatoxin-producing species, named *Aspergillus texensis*. The phylogenetic position of this species was studied using partial β-tubulin (0.9 kb), calmodulin (1.2 kb), and nitrate reductase (*niaD*, 2.1 kb) gene sequences (Singh *et al.* 2018). The former two gene regions are commonly used in *Aspergillus* taxonomy; however, the sequenced fragments are longer than those recommended by Samson *et al.* (2014). On the other hand, the nitrate reductase gene is part of the aflatoxin biosynthetic pathway and not commonly used in phylogenetic studies. Based on the results of Singh *et al.* (2018), *A*. *texensis* is closely related to *A*. *minisclerotigenes* and a previously reported unnamed lineage designated “Lethal Aflatoxicosis Fungus” (LAF). In this study, we re-evaluated the species boundaries of *A*. *minisclerotigenes* using a larger set of strains. Single gene phylogenies using *BenA*, *CaM*, and *niaD* sequences were constructed (using the longer fragments as described by Singh *et al.* (2018)) (Fig. 20). The five representative *A*. *texensis* strains clustered together in all analyses, indicating their close genetic relationship. The two included LAF strains (A1168, A1170) causing lethal aflatoxicosis in Kenya (ex maize), clustered together in the *BenA* and *niaD* phylogenies, and were closely related in the *CaM* phylogram. In the *BenA* phylogram, all *A*. *minisclerotigenes*, *A*. *texensis* and LAF strains clustered together with high statistical support and there were four minor deviating lineages present. Three main clades are present in the *CaM* phylogram. One clade contains the *A*. *texensis* strains and *A*. *minisclerotigenes* strains DTO 275-E9 and CBS 145094, one clade includes the two LAF strains, CBS 117635^T^ and four other *A*. *minisclerotigenes* strains and one clade with strains only identified as *A*. *minisclerotigenes*. There are also three main clades present in the *niaD* phylogram; however, the clade containing the LAF strains does not have statistical support. The position of *A*. *minisclerotigenes* CBS 145094 is basal to the other investigated strains. The *A*. *texensis* and the LAF strains clustered together in the combined analysis of the three genes, confirming the results of Singh *et al.* (2018). However, with the addition of *A*. *minisclerotigenes* strains and therefore increasing the genetic diversity within the analysis, it becomes clear that *A*. *texensis* and the LAF strains fall within the genetic diversity of *A*. *minisclerotigenes*. We therefore consider both as synonyms of *A*. *minisclerotigenes* Vaamonde *et al.*

***Penicillium attenuatum*** Kirichuk & Pivkin, Mycol. Prog. 16: 21. 2017.  

*Notes*: Kirichuk *et al.* (2017) introduced *Penicillium attenuatum*, *P*. *ochotense* and *P*. *piltunense* as new species closely related to *P*. *antarcticum* (sect. *Canescentia*, ser. *Atroveneta*) based on DNA sequences of the ITS, *BenA* and *CaM* regions. *Penicillium* sect. *Canescentia* is currently being reviewed (Visagie *et al.*, unpublished) and the authors found that the published sequences for those species are of poor quality. Alignment of them against reference sequences revealed numerous suspicious regions towards read ends (*e.g*., gaps within coding regions of *BenA* and *CaM*, as well as conserved regions of the 28S rDNA region of ITS). Removing suspected low quality read ends, resulted in phylograms where all three species resolve with *Penicillium antarcticum* A.D. Hocking & C.F. McRae. Unfortunately, these strains are not available for study and are thus reduced to synonyms.  

***Penicillium cellarum*** Strausbaugh & Dugan, Pl. Dis. 101: 1783. 2017.  

*Notes*: Strausbaugh & Dugan (2017) described *P*. *cellarum* from sugar beet. Based on *BenA* sequencing, this species is most closely related to *P*. *aurantiogriseum* in *Penicillium* sect. *Fasciculata*. Seven unique sequence types were found among the studied *P*. *cellarum* strains (F727^T^, F759, F761, F769, F776, F785, F790) (Strausbaugh and Dugan, 2017, Strausbaugh, 2018). Those *BenA*, *CaM* and *RPB2* sequences were re-analysed and compared with a set of nine *P*. *aurantiogriseum* strains from the CBS culture collection (Supplementary Fig. S4). The *BenA* analysis shows that all *P*. *cellarum* strains reside in a clade with *P*. *aurantiogriseum* with 88 % bootstrap support (no statistical support in the Bayesian analysis). The statistical support of the *P*. *aurantiogriseum* clade, including the *P*. *cellarum* sequences, is poor in the *CaM* analysis (<70 % BS, <0.95 pp). Strains F727^T^, F769 and F785 cluster together within the *P*. *aurantiogriseum* clade (81 % BS, 0.99 pp), while the other *P*. *cellarum* strains cluster with strains previously identified as *P*. *aurantiogriseum* (Frisvad & Samson 2004b). The phylogenetic analysis of the *RPB2* data set resulted in a less well-supported phylogram. The majority of the *RPB2* sequences obtained from the CBS strains (including CBS 324.89^T^) cluster with F769, F785, F761 and F790. A smaller clade with strains F727^T^, F759 and F776 is present in the *RPB2* phylogram (full support). The strains within this cluster differ from those in the cluster present in the *CaM* phylogram, showing that the phylograms are not congruent. These data indicate that *P*. *cellarum* is a synonym of *P*. *aurantiogriseum* Dierckx. This observation is also supported by the *BenA* phylogram.  

***Penicillium imranianum*** Imran Ali, Pakistan J. Bot. 50: 2055. 2018.  

*Notes*: The deposited ITS sequence in GenBank (KP780293) cannot be reliably aligned in a dataset that contains the ITS barcodes of the accepted species in the families *Aspergillaceae*, *Trichocomaceae* and *Thermoascaceae*. This is probably due to a sequencing error, because also the conserved 5.8S nrDNA region is difficult to align. The taxonomic position of *Penicillium imranianum* remains unresolved and is considered a doubtful species. Other genes need to be sequenced to get insight in the taxonomic position and status of this species.  

***Penicillium ochotense*** Kirichuk & Pivkin, Mycol. Prog. 16: 21. 2017.  

*Notes*: Synonym of *Penicillium antarcticum* A.D. Hocking & C.F. McRae; see notes for *P. attenuatum* above.  

***Penicillium pedernalense*** Laich & J. Andrade, Int. J. Syst. Evol. Microbiol. 66: 4386. 2016. *nom*. *inval*.  

*Notes*: *Penicillium pedernalense* was invalidly described and validated in 2018. In the original description, two strain numbers (CBS 140770; CECT 20949) were indicated as the type (invalid under Art. 40.7, Melbourne). The name was later validated with the CBS strain designated as holotype (Index Fungorum 361: 1).  

***Penicillium piltunense*** Kirichuk & Pivkin, Mycol. Prog. 16: 19 2017.  

*Notes*: Synonym of *Penicillium antarcticum* A.D. Hocking & C.F. McRae; see notes for *P. attenuatum* above.  

***Penicillium wandoense*** Hyang B. Lee *et al.*, Fungal Diversity 96: 105. 2019.  

*Notes*: Comparison of the *BenA*, *CaM* and *RPB2* sequences deposited in GenBank shows that *Penicillium laevigatum* and *P*. *wandoense* are conspecific. *Penicillium laevigatum* was effectively published online on 19 December 2018 (online version of record) whereas the other article was only published on 24 June 2019. *Penicillium laevigatum* L. Cai *et al.* has priority over *P*. *wandoense*.  

***Phialosimplex halophilus*** [as “*halophila*”] (F.J.H. Beyma) Greiner *et al.*, IMA Fungus 5: 170. 2014.  

*Notes*: Samson *et al.* (2014) combined *Basipetospora halophila* (basionym *Oospora halophila* J.F.H. Beyma) in *Aspergillus* as *Aspergillus baarnensis*. In the same year, Greiner *et al.* (2014) combined this species in the genus *Phialosimplex*. Even though *Phialosimplex* species (*e.g*., *Ph*. *caninus*, the generic type) produce reduced conidiophores or solitary phialides (and lack typical aspergillus-type conidiophores), they phylogenetically belong to *Aspergillus* subgen. *Polypaecilum* (Fig. 17). We therefore accept *A*. *baarnensis* Samson *et al.*, which is in agreement with Tanney *et al.* (2017).  

***Phialosimplex salinarum*** (Greiner *et al.*) Zalar & Greiner, Extremophiles 21: 762. 2017.  

*Notes*: Martinelli *et al.* (2017) combined *Ph*. *salinarum* in *Aspergillus* (as *Aspergillus salinarus*) and Tanney *et al.* (2017) accepted this combination in their overview paper on *Aspergillus* subgen. *Polypaecilum*. Based on the multigene phylogenetic study performed here, we treat *Phialosimplex* as a synonym of *Aspergillus* and accept *Aspergillus salinarum* [as “*salinarus*”] (Greiner *et al.*) Zalar & Greiner over *Ph*. *salinarum*.  

***Talaromyces omanensis*** Halo *et al.*, Phytotaxa 404: 192. 2019.  

*Notes*: *Penicillium resedanum* belongs to *Talaromyces* and will be combined in this genus as *Talaromyces resedanus* elsewhere. Based on sequence and phenotypic similarities, *Tal*. *omanensis* as a synonym of *Tal*. *resedanus* (pers. communication, A. J. Chen).  

***Talaromyces rubrifaciens*** W.W. Gao, Mycologia 108: 775. 2016.  

*Notes*: During a study on new *Talaromyces* species from indoor environments in China, Chen *et al.* (2016b) synonymised *Tal*. *rubrifaciens* with *Tal*. *albobiverticillius* based on GCPSR approach and phenotypic characters. Similarly as with *A*. *parafelis* and *A*. *pseudofelis*, a limited number of *Tal*. *albobiverticillius* sequences were included and the full sequence diversity was not represented in the phylogenetic analyses. We follow Chen *et al.* (2016b) and treat *Tal. rubrifaciens* as a synonym of *Talaromyces albobiverticillius* (H.-M. Hsieh *et al.*) Samson *et al.*

### List of species in *Eurotiales*

A list of “Names in Current Use” (NCU) for the family *Trichocomaceae* was prepared in 1993 and was of great value for anyone working with these fungi (Pitt & Samson 1993). The list was updated in 2000, and this version included the accepted species and their synonyms in this family (Pitt *et al.* 2000). In total, 29 genera and 617 species were accepted. The genera *Penicillium* and *Aspergillus* made up for the majority of the included species. New overviews of accepted species in *Aspergillus* (Samson *et al.* 2014), *Penicillium* (Visagie *et al.* 2014b) and *Talaromyces* (Yilmaz *et al.* 2014) were published. These lists were urgently needed, due to many changes since the list of 2000. The majority of changes were caused by the move from a dual to a single name nomenclature system (McNeill *et al.* 2012), with the result that many teleomorphic genera were synonymised with *Penicillium* or *Aspergillus* (Houbraken and Samson, 2011, Samson et al., 2014). Furthermore, new names were introduced due to descriptions of new species, and old names were resurrected because taxonomic studies using molecular data showed that the phenotype-based species were actually consisting of complexes of multiple species (*e.g*., sibling species such as *Aspergillus niger* and *A*. *welwitschiae*; Hong *et al.* (2013). Another advantage of the “2014 *Aspergillus*/*Penicillium*/*Talaromyces* lists” are the inclusion of MycoBank numbers, type and ex-type culture (accession number) information and GenBank accession numbers for *BenA*, *CaM*, ITS and *RPB2* sequences.

 Compared with the “2014 *Aspergillus*/*Penicillium*/*Talaromyces* lists”, the list of species presented below is expanded with the species described after publication in 2014. In the “2014 *Aspergillus*/*Penicillium*/*Talaromyces* lists”, 339 *Aspergillus*, 354 *Penicillium* and 88 *Talaromyces* species were accepted. These numbers increased significantly, and now includes 446 *Aspergillus* (32 % increase), 483 *Penicillium* (36 % increase) and 171 *Talaromyces* (94 % increase) species. An overview of the number of accepted species in the 2000, 2014 and the current list is given in Table 6. We decided that if no sequence data is available for examination of the type or representative strain, then we only included the species that were described since 1970. Also, species such as *A*. *amazonensis* were excluded because it was described in 1904, but combined later (in 1985) (Samson & Seifert 1985). Additional *BenA*, *CaM*, ITS and *RPB2* sequences were generated for the species lacking this data in GenBank or for those that were linked to poor or short sequences. Sequence data are now available for the majority of species. Of the 446 *Aspergillus* taxa, 3.1 % (n = 14), 3.1 % (n = 14), 4.5 % (n = 20) and 5.1 % (n = 23) of the species are missing *BenA*, *CaM*, ITS or *RPB2* sequences, respectively. No sequence data are available for 13 species: *Aspergillus argenteus*, *A*. *beijingensis*, *A*. *collembolorum*, *A*. *crassihyphae*, *A*. *curviformis*, *A*. *ellipsoideus*, *A*. *maritimus*, *A*. *qizutongii*, *A*. *raianus*, *A*. *subunguis*, *A*. *tapirirae*, *A*. *vinosobubalinus*, *A*. *wangduanlii*, and only an ITS sequence is available for *Aspergillus assiutensis*. The taxonomic position of these species needs to be confirmed. The list contains 483 *Penicillium* species and *BenA*, *CaM*, ITS and *RPB2* sequences are lacking for 1.9 % (n = 8), 2.3 % (n = 11), 2.3 %, (n = 11) and 5.4 % (n = 26) of the taxa, respectively. No sequence data are available *Penicillium asymmetricum*, *P*. *coniferophilum*, *P*. *glaucoalbidum*, *P*. *longisporum*, *P*. *melanostipe*, *P*. *parviverrucosum* and *P*. *taiwanense*. Besides these species, only *P*. *dravunii* is not represented by a *BenA* sequence (an ITS sequence is known though, which is distinct from all other accepted species). Out of the 171 *Talaromyces* species, three (1.8 %) taxa do not have a *BenA* sequence, five (2.9 %) are lacking *CaM* sequences, one (0.6 %) is lacking an ITS sequence and nine (5.3 %) *RPB2* sequences. New in the list is the inclusion of other genera and species belonging to the *Eurotiales* (*e.g*., *Monascus*, *Paecilomyces*, *Rasamsonia*), with exception of taxa classified in the *Elaphomycetaceae*. The list includes 1 187 species, distributed over 27 genera (Table 6). Due to the change to a single name nomenclature system, a name does not reflect the ability of a species to produce a sexual morph anymore. In order to make this information readily available, we also included information on the mode of reproduction. Species could be asexual, homothallic, heterothallic or protoheterothallic. The latter term is used in those species where both idiomorphs are known in the species populations; if only one of the idiomorphs is known, then the species is listed as asexual (Houbraken & Dyer 2015). Information on several type specimens are not available or has been lost for various reasons. Where ex-type strains were available, new dried specimens were prepared and the typifications fixed below.

**Table 6:** Genera and number of species in current and earlier lists.

Genus	Number of accepted species			Number of species producing sexual morph
	Pitt *et al.* (2000)	2014-lists^3^	This study^4^	
*Acidotalaromyces*	–	–	1	0
*Ascospirella*	–	–	1	1
*Aspergillago*	–	–	1	0
*Aspergillus*^1^	184	339	446	139 (incl. 19 heterothallic spp.)
*Byssochlamys*	4	–	*Paecilomyces*	n/a
*Chaetosartorya*	3	–	*Aspergillus*	n/a
*Cristaspora*	1	–	*Aspergillus*	n/a
*Dendrosphaera*	1	–	1	1
*Dichlaena*	1	–	2	2
*Dichotomomyces*	1	–	*Aspergillus*	n/a
*Emericella*	27	–	*Aspergillus*	n/a
*Eupenicillium*	43	–	*Penicillium*	n/a
*Eurotium*	19	–	*Aspergillus*	n/a
*Evansstolkia*	–	–	1	1
*Fennellia*	3	–	*Aspergillus*	n/a
*Geosmithia*^1^	8	–	*Hypocreales*	n/a
*Hamigera*	1	–	9	8
*Hemicarpenteles*	2	–	*Penicillium*	n/a
*Leiothecium*	–	–	2	2
*Merimbla*^1^	1	–	*Hamigera*	n/a
*Monascus*	222	–	9	7
*Neosartorya*	12	–	*Aspergillus*	n/a
*Paecilomyces*^1^	41	–	10	5 (incl. 1 heterothallic spp.)
*Penicillago*	–	–	4	0
*Penicilliopsis*	2	–	4	3
*Penicillium*^1^	225	354	483	64 (incl. 2 heterothallic spp.)
*Petromyces*	2	–	*Aspergillus*	n/a
*Phialomyces*	–	–	4	0
*Pseudohamigera*	–	–	1	1
*Pseudopenicillium*	–	–	3	3
*Raperia*^1^	1	–	*Warcupiella*	n/a
*Rasamsonia*	–	–	12	4
*Sagenomella*	–	–	6	1
*Sarophorum*^1^	1	–	*Penicilliopsis*	n/a
*Sclerocleista*	2	–	2	2
*Stilbodendron*^1^	1	–	*Penicilliopsis*	n/a
*Talaromyces*	24	88	171	40 (incl. 3 heterothallic spp.)
*Thermoascus*	4	–	7	7
*Thermomyces*	–	–	2	1
*Torulomyces*^1^	1	–	*Penicillium*	n/a
*Trichocoma*	1	–	1	1
*Warcupiella*	1	–	1	1
*Xerochrysium*	–	–	2	0
*Xeromyces*	–	–	1	1
**Total**	**617**^2^	**781**^3^	**1** **187**	**295 (incl. 25 heterothallic spp.)**

1 Genera originally introduced for strictly asexual reproducing species.

2 Species producing a sexual and asexual morph are counted two times in this list due to the at that time dual nomenclature rules.

3 Species of genera other than *Aspergillus*, *Penicillium* and *Talaromyces* were not included in the 2014-lists.

4 If applicable, then the current generic name is provided.

#### List of accepted species

***Acidotalaromyces***

***Acidotalaromyces lignorum*** (Stolk) Houbraken, Frisvad & Samson, this study. 2020. [MB832555]. Basionym: *Penicillium lignorum* Stolk, Antonie van Leeuwenhoek 35: 264. 1969. [MB335743]. — Type: CBS 709.68. Ex-type: CBS 709.68 = ATCC 22051 = FRR 804 = IMI 151899 = UPSC 3184. Reproduction: asexual. ITS barcode: JF910285 (alternative markers: *BenA* = HQ156946; *CaM* = JX140680; *RPB2* = MN969215).  

***Ascospirella***

***Ascospirella lutea*** (Zukal) Houbraken *et al.*, this study. 2020. [MB832556]. Basionym: *Penicillium luteum* Zukal, Sitzungsber. Kaiserl. Akad. Wiss. Math.-Naturwiss. Cl., Abt. 1 98: 561. 1890. [MB306716]. — Type: IMI 89305. Ex-type: CBS 348.51 = DTO 165-C7 = CECT 2950 = IFO 31753 = IMI 089305 = LSHB BB228. Reproduction: homothallic. ITS barcode: MN431414 (alternative markers: *BenA* = MN969437; *CaM* = MN969356; *RPB2* = MN969216).  

***Aspergillago***

***Aspergillago clavatoflava*** (Raper & Fennell) Samson *et al.*, Stud. Mycol. 85: 211. 2016. [MB819187]. Basionym: *Aspergillus clavatoflavus* Raper & Fennell, Gen. Aspergillus: 378. 1965. [MB326619]. — Type: WB5113. Ex-type: DTO 022-B2 = CBS 473.65 = NRRL 5113 = ATCC 16866 = IMI 124937 = LCP 89.2589. Reproduction: asexual. ITS barcode: EF669713 (alternative markers: *BenA* = EF669686; *CaM* = EF669700; *RPB2* = EF669668).  

***Aspergillus***

***Aspergillus acanthosporus*** Udagawa & Takada, Bull. Nat. Sci. Mus. Tokyo 14: 503. 1971. [MB309201]. — Type: NHL 22462. Ex-type: CBS 558.71 = NRRL 5293 = ATCC 22931 = IMI 164621 = NHL 2462. Infragen. class: subgen. *Fumigati*, sect. *Clavati*, ser. *Clavati*. Reproduction: homothallic. ITS barcode: EU078625 (alternative markers: *BenA* = MN969364; *CaM* = EU078676; *RPB2* = EF669779).

***Aspergillus acidohumus*** A.J. Chen *et al.*, Stud. Mycol. 85: 71. 2016. [MB817723]. — Type: CBS H-22730. Ex-type: CBS 141577 = CGMCC 3.18217 = DTO 340-H1 = IBT 34346. Infragen. class: subgen. *Fumigati*, sect. *Cervini*, ser. *Acidohumorum*. Reproduction: asexual. ITS barcode: KX423646 (alternative markers: *BenA* = KX423623; *CaM* = KX423634; *RPB2* = KX423663).

***Aspergillus acrensis*** Hubka *et al.*, Persoonia 41: 163. 2018. [MB822542]. — Type: IFM 57291H. Ex-type: IFM 57291 = CCF 4670. Infragen. class: subgen. *Fumigati*, sect. *Fumigati*, ser. *Viridinutantes*. Reproduction: protoheterothallic; both MAT idiomorphs detected (Hubka *et al.* 2018a). ITS barcode: n.a. (alternative markers: *BenA* = LT795980; *CaM* = LT795981; *RPB2* = LT795982).

***Aspergillus aculeatinus*** Noonim *et al.*, Int. J. Syst. Evol. Microbiol. 58: 1733. 2008. [MB505075]. — Type: unknown. Ex-type: CBS 121060 = DTO 202-G5 = IBT 29077. Infragen. class: subgen. *Circumdati*, sect. *Nigri*, ser. *Japonici*. Reproduction: protoheterothallic; unpublished (genome data; Vesth *et al.* 2018). ITS barcode: EU159211 (alternative markers: *BenA* = EU159220; *CaM* = EU159241; *RPB2* = HF559233).

***Aspergillus aculeatus*** Iizuka, J. Agric. Chem. Soc. Japan 27: 806. 1953. [MB292831]. — Type: IMI 211388. Ex-type: CBS 172.66 = NRRL 5094 = NRRL 20623 = IMI 211388 = ATCC 16872 = WB 5094. Infragen. class: subgen. *Circumdati*, sect. *Nigri*, ser. *Japonici*. Reproduction: protoheterothallic; MAT1-2-1 detected (de Vries *et al.* 2017). ITS barcode: EF661221 (alternative markers: *BenA* = HE577806; *CaM* = EF661148; *RPB2* = EF661046).

***Aspergillus aeneus*** Sappa, Allionia 2: 84. 1954. [MB292832]. — Type: CBS H-6735. Ex-type: CBS 128.54 = NRRL 4769 = ATCC 16803 = IMI 69855 = LSHBBB 355 = MUCL 13570 = QM 1945 = WB 4279 = WB 4769. Infragen. class: subgen. *Nidulantes*, sect. *Aenei*, ser. *Aenei*. Reproduction: asexual. ITS barcode: EF652474 (alternative markers: *BenA* = EF652298; *CaM* = EF652386; *RPB2* = EF652210).

***Aspergillus aerius*** A.J. Chen *et al.*, Stud. Mycol. 88: 79. 2017. [MB818731]. — Type: CBS H-22823. Ex-type: CBS 141771 = DTO 241-G7 = IBT 34446. Infragen. class: subgen. *Aspergillus*, sect. *Aspergillus*, ser. *Aspergillus*. Reproduction: homothallic. ITS barcode: LT670916 (alternative markers: *BenA* = LT670990; *CaM* = LT670991; *RPB2* = LT670992).

***Aspergillus affinis*** Davolos *et al.*, Int. J. Syst. Evol. Microbiol. 62: 1014. 2012. [MB517245]. — Type: ATCC MYA-4773. Ex-type: CBS 129190 = DTO 223-C6 = BT 32310 = TCC MYA-4773. Infragen. class: subgen. *Circumdati*, sect. *Circumdati*, ser. *Circumdati*. Reproduction: asexual. ITS barcode: MN431360 (alternative markers: *BenA* = GU721092; *CaM* = GU721091; *RPB2* = MN969063).

***Aspergillus aflatoxiformans*** Frisvad *et al.*, Stud. Mycol. 93: 32. 2019. [MB823770]. — Type: CBS H-23361. Ex-type: CBS 143679 = DTO 228-G2 = IBT 32085. Infragen. class: subgen. *Circumdati*, sect. *Flavi*, ser. *Flavi*. Reproduction: protoheterothallic; both MAT idiomorphs detected (Carvajal-Campos *et al.* 2017, referred to as *A*. *parvisclerotigenes*). ITS barcode: MG662388 (alternative markers: *BenA* = MG517706; *CaM* = MG518076; *RPB2* = MG517897).

***Aspergillus alabamensis*** Balajee *et al.*, Eukaryot. Cell 8: 720. 2009. [MB543648]. — Type: UAB20. Ex-type: CBS 125693 = UAB20 = DTO 045-C5. Infragen. class: subgen. *Circumdati*, sect. *Terrei*, ser. *Terrei*. Reproduction: asexual. ITS barcode: KP987071 (alternative markers: *BenA* = KP987049; *CaM* = EU147583; *RPB2* = KP987018).

***Aspergillus allahabadii*** B.S. Mehrotra & Agnihotri, Mycologia 54: 400. 1963. [MB326609]. — Type: CBS H-6736. Ex-type: CBS 164.63 = NRRL 4539 = ATCC 15055 = IMI 139273 = MUCL 13571 = WB 4539. Infragen. class: subgen. *Circumdati*, sect. *Terrei*, ser. *Nivei*. Reproduction: asexual. ITS barcode: EF669601 (alternative markers: *BenA* = EF669531; *CaM* = EF669559; *RPB2* = EF669643).

***Aspergillus alliaceus*** Thom & Church, Aspergilli: 163. 1926. [MB256402]. — Type: CBS H-7812. Ex-type: CBS 536.65 = DTO 034-B3 = DTO 046-B1 = ATCC 10060 = DSM 813 = IFO 7538 = IMI 051982 = IMI 051982ii = NRRL 315 = QM 1885 = WB 315. Infragen. class: subgen. *Circumdati*, sect. *Flavi*, ser. *Alliacei*. Reproduction: homothallic (Fennell & Warcup 1959). ITS barcode: EF661551 (alternative markers: *BenA* = EF661465; *CaM* = EF661534; *RPB2* = MG517825).

***Aspergillus amazonicus*** D. Mares, Curr. Microbiol. 57: 228. 2008. [MB531888]. — Type: E19D. Ex-type: CBS 124228 = DTO 092-D6 = DTO 411-B6. Infragen. class: subgen. *Nidulantes*, sect. *Sparsi*, ser. *Conjuncti*. Reproduction: asexual. ITS barcode: MN431399 (alternative markers: *BenA* = FJ943939; *CaM* = FJ943936; *RPB2* = KU866979).

***Aspergillus ambiguus*** Sappa, Allionia 2: 254. 1955. [MB292834]. — Type: CBS H-6737. Ex-type: CBS 117.58 = NRRL 4737 = ATCC 16827 = IMI 139274 = QM 8155 = WB 4737. Infragen. class: subgen. *Circumdati*, sect. *Terrei*, ser. *Ambigui*. Reproduction: asexual. ITS barcode: EF669606 (alternative markers: *BenA* = EF669534; *CaM* = EF669564; *RPB2* = EF669648).

***Aspergillus amethystinus*** F. Sklenář *et al.*, Mycologia 112: 356. 2020. [MB832712]. — Type: PRM 951579. Ex-type: NRRL 4178 = CCF 5261. Infragen. class: subgen. *Nidulantes*, sect. *Nidulantes*, ser. *Nidulantes*. Reproduction: homothallic. ITS barcode: EF652462 (alternative markers: *BenA* = EF652286; *CaM* = EF652374; *RPB2* = EF652198).

***Aspergillus amoenus*** M. Roberg, Hedwigia 70: 138. 1931. [MB250654]. — Type: Münster i.W., isol. ex Berberis sp. fruit, M. Roberg (type locality, this specimen was not deposited into herbarium). Ex-type: NRRL 4838 = CBS 111.32. Infragen. class: subgen. *Nidulantes*, sect. *Nidulantes*, ser. *Versicolores*. Reproduction: protoheterothallic; MAT 1-1-1 detected . ITS barcode: EF652480 (alternative markers: *BenA* = JN853946; *CaM* = JN854035; *RPB2* = JN853824).

***Aspergillus angustatus*** A.J. Chen *et al.*, Stud. Mycol. 84: 41. 2016. [MB816090]. — Type: CBS H-22487. Ex-type: CBS 273.65 = DTO 319-H8. Infragen. class: subgen. *Nidulantes*, sect. *Nidulantes*, ser. *Stellati*. Reproduction: homothallic. ITS barcode: EU448283 (alternative markers: *BenA* = AY339993; *CaM* = EU443984; *RPB2* = KU867013).

***Aspergillus anthodesmis*** Bartoli & Maggi, Trans. Brit. Mycol. Soc. 71: 386. 1979 [1978]. [MB309207]. — Type: RO 103 S. Ex-type: CBS 552.77 = NRRL 22884 = IMI 223070. Infragen. class: subgen. *Nidulantes*, sect. *Sparsi*, ser. *Conjuncti*. Reproduction: asexual. ITS barcode: FJ491662 (alternative markers: *BenA* = EF661108; *CaM* = FJ491648; *RPB2* = EF661039).

***Aspergillus appendiculatus*** Blaser, Sydowia 28: 38. 1975. [MB309209]. — Type: ZT 8286. Ex-type: CBS 374.75 = DTO 196-H3 = ETH8286 = IMI 278374 = KACC 45268. Infragen. class: subgen. *Aspergillus*, sect. *Aspergillus*, ser. *Rubri*. Reproduction: homothallic. ITS barcode: HE615132 (alternative markers: *BenA* = HE801333; *CaM* = HE801318; *RPB2* = HE801307).

***Aspergillus arachidicola*** Pildain *et al.*, Int. J. Syst. Evol. Microbiol. 58: 730. 2008. [MB505189]. — Type: Pildain *et al.* 2008, Int. J. Syst. Evol. Microbiol. 58: p. 731 Fig. 2 (– lectotype designated here, MBT392255; CBS H-24274 [dried culture] – epitype designated here, MBT392256). Ex-epitype: DTO 009-G3 = CBS 117610 = IBT 117610 = IBT 25020. Infragen. class: subgen. *Circumdati*, sect. *Flavi*, ser. *Flavi*. Reproduction: protoheterothallic; both MAT idiomorphs detected (Carvajal-Campos *et al.* 2017). ITS barcode: MF668184 (alternative markers: *BenA* = EF203158; *CaM* = EF202049; *RPB2* = MG517802).

***Aspergillus arcoverdensis*** Y. Horie *et al.*, Mycoscience 56: 130. 2015. [MB804028]. — Type: CBM-FA-39845. Ex-type: IFM 61334 = JCM 19878 = CCF 4695 = CBS 139187 = DTO 316-F7. Infragen. class: subgen. *Fumigati*, sect. *Fumigati*, ser. *Viridinutantes*. Reproduction: protoheterothallic; both MAT idiomorphs detected (Matsuzawa *et al.* 2015). ITS barcode: MN431385 (alternative markers: *BenA* = AB818845; *CaM* = AB818856; *RPB2* = MN969103).

***Aspergillus ardalensis*** A. Nováková *et al.*, Mycologia 107: 179. 2015. [MB808140]. — Type: PRM 923450. Ex-type: CCF 4031 = CCF 4426 = CMF ISB 1688 = CBS 134372 = NRRL 62824. Infragen. class: subgen. *Circumdati*, sect. *Flavipedes*, ser. *Flavipedes*. Reproduction: asexual. ITS barcode: FR733808 (alternative markers: *BenA* = HG916683; *CaM* = HG916725; *RPB2* = HG916704).

***Aspergillus arenarioides*** Visagie, Hirooka & Samson, Stud. Mycol. 78: 110. 2014. [MB809195]. — Type: CBS H-21812. Ex-type: CBS 138200 = DTO 268-E3 = CCF 4928. Infragen. class: subgen. *Circumdati*, sect. *Petersoniorum*, ser. *Petersoniorum*. Reproduction: asexual. ITS barcode: KJ775562 (alternative markers: *BenA* = KJ775091; *CaM* = KJ775390; *RPB2* = LN849430).

***Aspergillus argenteus*** J.N. Rai & H.J. Chowdhery, Kavaka 7: 19. 1979. [MB116063]. — Type: MLLU 104. Ex-type: Infragen. class: subgen. *Nidulantes*, sect.: unknown, ser.: unknown. Reproduction: asexual. ITS barcode: n.a. (alternative markers: *BenA* = n.a.; *CaM* = n.a.; *RPB2* = n.a.).

***Aspergillus arxii*** (Fort & Guarro) Houbraken, Visagie & Samson, Stud. Mycol. 78: 154. 2014. [MB809575]. Basionym: *Cristaspora arxii* Fort & Guarro, Mycologia 76: 1115. 1984. [MB106038]. — Type: CBS H-14047. Ex-type: CBS 525.83 = ATCC 52744 = FMR 416. Infragen. class: subgen. *Cremei*, sect. *Cremei*, ser. *Arxiorum*. Reproduction: homothallic; asexual morph unknown. ITS barcode: MN431361 (alternative markers: *BenA* = MN969365; *CaM* = MN969223; *RPB2* = JN121529).

***Aspergillus asclerogenus*** Jurjević & Hubka, Plant Syst. Evol. 301: 2451. 2015. [MB814441]. — Type: PRM 933843. Ex-type: CCF 4947 = NRRL 58502. Infragen. class: subgen. *Circumdati*, sect. *Petersoniorum*, ser. *Petersoniorum*. Reproduction: asexual. ITS barcode: LN849392 (alternative markers: *BenA* = LN849406; *CaM* = LN849421; *RPB2* = LN849437).

***Aspergillus askiburgiensis*** A. Nováková *et al.*, Plant Syst. Evol. 302: 1285. 2016. [MB816280]. — Type: PRM 924055. Ex-type: CCF 4716 = CCF 4428 = CBS 134374 = NRRL 62818 = IBT 33114 = IBT 32911. Infragen. class: subgen. *Nidulantes*, sect. *Nidulantes*, ser. *Speluncei*. Reproduction: asexual. ITS barcode: LN873939 (alternative markers: *BenA* = LN873952; *CaM* = LN873965; *RPB2* = LN873984).

***Aspergillus aspearensis*** Houbraken *et al.*, Stud. Mycol. 93: 32. 2019. [MB823771]. — Type: CBS H-23358. Ex-type: CBS 143672 = DTO 203-D9 = CCTU 758 = IBT 32590 = IBT 34544. Infragen. class: subgen. *Circumdati*, sect. *Flavi*, ser. *Leporum*. Reproduction: asexual. ITS barcode: MG662398 (alternative markers: *BenA* = MG517669; *CaM* = MG518040; *RPB2* = MG517857).

***Aspergillus asper*** Jurjević & S.W. Peterson, Int. J. Syst. Evol. Microbiol. 66: 2567. 2016. [MB814412]. — Type: BPI-893218. Ex-type: CBS 140842 = NRRL 35910 = CCF 5174. Infragen. class: subgen. *Nidulantes*, sect. *Usti*, ser. *Calidousti*. Reproduction: asexual. ITS barcode: KT698840 (alternative markers: *BenA* = KT698838; *CaM* = KT698839; *RPB2* = KT698842).

***Aspergillus asperescens*** Stolk, Antonie van Leeuwenhoek 20: 303. 1954. [MB292835]. — Type: IMI 46813. Ex-type: CBS 110.51 = NRRL 2252 = NRRL 4770 = ATCC 11079 = DSM 871 = IMI 46813 = QM 1946 = WB 2252 = WB 4770 = WB 5038. Infragen. class: subgen. *Nidulantes*, sect. *Nidulantes*, ser. *Speluncei*. Reproduction: asexual. ITS barcode: EF652475 (alternative markers: *BenA* = EF652299; *CaM* = EF652387; *RPB2* = EF652211).

***Aspergillus assiutensis*** Moub. & Soliman, J. Basic Appl. Mycol. 2: 84. 2011. [MB584202]. — Type: AUMC 5748. Ex-type: CBS 132773 = AUMC 5748. Infragen. class: subgen. *Circumdati*, sect. *Nigri*, ser. *Japonici*. Reproduction: asexual. ITS barcode: JN393254 (alternative markers: *BenA* = n.a.; *CaM* = n.a.; *RPB2* = n.a.).

***Aspergillus assulatus*** (S.B. Hong *et al.*) Houbraken *et al.*, Stud. Mycol. 78: 154. 2014. [MB809576]. Basionym: *Neosartorya assulata* S.B. Hong *et al.*, Antonie van Leeuwenhoek 93: 95. 2008. [MB506376]. — Type: KACC 41691. Ex-type: IBT 27911 = DTO 043-E8. Infragen. class: subgen. *Fumigati*, sect. *Fumigati*, ser. *Unilaterales*. Reproduction: homothallic. ITS barcode: HF545007 (alternative markers: *BenA* = DQ114123; *CaM* = MN969222; *RPB2* = HF545311).

***Aspergillus astellatus*** (Fennell & Raper) Houbraken *et al.*, Stud. Mycol. 78: 154. 2014. [MB809577]. Basionym: *Aspergillus variecolor* var. *astellatus* Fennell & Raper, Mycologia 47: 81. 1955. [MB346549]. — Type: IMI 061455. Ex-type: CBS 134.55 = CBS 261.93 = NRRL 2396 = ATCC 16817 = IMI 61455 = IMI 61455ii = NRRL A-1634 = QM 1910 = WB 2396. Infragen. class: subgen. *Nidulantes*, sect. *Nidulantes*, ser. *Stellati*. Reproduction: homothallic. ITS barcode: EF652446 (alternative markers: *BenA* = EF652270; *CaM* = EF652358; *RPB2* = EF652182).

***Aspergillus atacamensis*** Zalar *et al.*, Extremophiles 21:766. 2017. [MB818565]. — Type: CBS H-23062. Ex-type: EXF-6660 = CBS 142046 = DTO 411-B9. Infragen. class: subgen. *Polypaecilum*, sect. *Polypaecilum*, ser. *Kalimarum*. Reproduction: asexual. ITS barcode: KX900619 (alternative markers: *BenA* = MN969415; *CaM* = MN969325; *RPB2* = MN969192).

***Aspergillus aurantiacoflavus*** Hubka *et al.*, Stud. Mycol. 88: 82. 2017. [MB818732]. — Type: CBS H-22827. Ex-type: CBS 141930 = EMSL No. 2903 = CCF 5393 = DTO 355-I1 = IBT 34485. Infragen. class: subgen. *Aspergillus*, sect. *Aspergillus*, ser. *Aspergillus*. Reproduction: homothallic. ITS barcode: LT670917 (alternative markers: *BenA* = LT670993; *CaM* = LT670994; *RPB2* = LT670995).

***Aspergillus aurantiobrunneus*** (G.A. Atkins *et al.*) Raper & Fennell, Gen. Aspergillus: 511. 1965. [MB326612]. Basionym: *Emericella nidulans* var. *aurantiobrunnea* G.A. Atkins, Hindson & A.B. Russell, Trans. Brit. Mycol. Soc. 41: 504. 1958 [MB346743]. — Type: IMI 74897. Ex-type: CBS 465.65 = NRRL 4545 = NRRL 2775 = ATCC 16821 = IMI 074897. Infragen. class: subgen. *Nidulantes*, sect. *Nidulantes*, ser. *Aurantiobrunnei*. Reproduction: homothallic. ITS barcode: EF652465 (alternative markers: *BenA* = EF652289; *CaM* = EF652377; *RPB2* = EF652201).

***Aspergillus aurantiopurpureus*** A.J. Chen *et al.*, Stud. Mycol. 84: 46. 2016. [MB816087]. — Type: CBS H-22488. Ex-type: CBS 140608 = IBT 12601 = DTO 060-A7. Infragen. class: subgen. *Nidulantes*, sect. *Nidulantes*, ser. *Nidulantes*. Reproduction: homothallic. ITS barcode: KU866588 (alternative markers: *BenA* = KU866824; *CaM* = KU866711; *RPB2* = KU866966).

***Aspergillus auratus*** Warcup, Gen. Aspergillus: 263. 1965. [MB326613]. — Type: IMI 75886. Ex-type: CBS 466.65 = NRRL 4378 = ATCC 16894 = IFO 8783 = IMI 75886 = QM 7861 = WB 4378. Infragen. class: subgen. *Fumigati*, sect. *Fumigati*, ser. *Neoglabri*. Reproduction: homothallic. ITS barcode: EF669979 (alternative markers: *BenA* = EF669835; *CaM* = EF669905; *RPB2* = EF669766).

***Aspergillus aureolatus*** Munt.-Cvetk. & Bata, Bull. Inst. Jard. Bot. Univ. Beograd 1: 196. 1964. [MB326614]. — Type: CBS H-6738. Ex-type: CBS 190.65 = NRRL 5126 = ATCC 16810 = IMI 136527 = IMI 136527ii = WB 5126. Infragen. class: subgen. *Nidulantes*, sect. *Nidulantes*, ser. *Speluncei*. Reproduction: asexual. ITS barcode: EF652501 (alternative markers: *BenA* = EF652325; *CaM* = EF652413; *RPB2* = EF652237).

***Aspergillus aureolus*** Fennell & Raper, Mycologia 47: 71. 1955. [MB292836]. — Type: CBS 105.55. Ex-type: CBS 105.55 = NRRL 2244 = ATCC 16896 = IFO 8105 = IMI 61451 = IMI 061451ii = MUCL 13579 = QM 1906 = WB 2244 = DTO 052-C8 = DTO 331-G6. Infragen. class: subgen. *Fumigati*, sect. *Fumigati*, ser. *Viridinutantes*. Reproduction: homothallic. ITS barcode: EF669950 (alternative markers: *BenA* = EF669808; *CaM* = EF669877; *RPB2* = EF669738).

***Aspergillus aureoterreus*** Samson *et al.*, Stud. Mycol. 69: 45. 2011. [MB560392]. — Replaced synonym: *Aspergillus terreus* var. *aureus* Thom & Raper, A manual of the Aspergilli: 198. 1945. [MB351655]. — Type: Thom & Raper 1945, A Manual of the Aspergilli: p. 199 Fig. 57B (– lectotype designated here, MBT392257; CBS H-24275 [dried culture] – epitype designated here, MBT392258). Ex-epitype: CBS 503.65 = NRRL 1923 = ATCC 16793 = IFO 30536 = IMI 82431 = MUCL 38644 = QM 7472 = VKM F-2035 = WB 1923. Infragen. class: subgen. *Circumdati*, sect. *Terrei*, ser. *Terrei*. Reproduction: asexual. ITS barcode: EF669580 (alternative markers: *BenA* = EF669524; *CaM* = EF669538; *RPB2* = EF669622).

***Aspergillus auricomus*** (Guég.) Saito, J. Ferment. Technol. 17: 3. 1939. [MB119950]. Basionym: *Sterigmatocystis auricoma* Guég., Bull. Soc. Mycol. Fr. 15: 186. 1899. [MB209799]. — Type: CBS H-9173. Ex-type: CBS 467.65 = NRRL 391 = IBT 14581 = ATCC 16890 = IMI 172277 = LCP 89.2596 = LSHBA 41 = WB 391. Infragen. class: subgen. *Circumdati*, sect. *Circumdati*, ser. *Circumdati*. Reproduction: asexual. ITS barcode: EF661411 (alternative markers: *BenA* = EF661320; *CaM* = EF661379; *RPB2* = EF661300).

***Aspergillus australiensis*** [as “*australensis*”] (Samson *et al.*) Houbraken *et al.*, Stud. Mycol. 78: 154. 2014. [MB821660]. Basionym: *Neosartorya australensis* Samson, S.B. Hong & Varga, Stud. Mycol. 59: 174. 2007. [MB492203]. — Type: CBS 112.55. Ex-type: CBS 112.55 = NRRL 2392 = IMI 061450 = DTO 026-H3. Infragen. class: subgen. *Fumigati*, sect. *Fumigati*, ser. *Neoglabri*. Reproduction: homothallic. ITS barcode: EF669953 (alternative markers: *BenA* = EF669811; *CaM* = EF669880; *RPB2* = EF669741).

***Aspergillus austroafricanus*** Jurjević *et al.*, IMA Fungus 3: 67. 2012. [MB800597]. — Type: BPI 880914. Ex-type: CBS 145748 = NRRL 233 = DTO 225-D8. Infragen. class: subgen. *Nidulantes*, sect. *Nidulantes*, ser. *Versicolores*. Reproduction: asexual. ITS barcode: JQ301891 (alternative markers: *BenA* = JN853963; *CaM* = JN854025; *RPB2* = JN853814).

***Aspergillus austwickii*** Frisvad *et al.*, Stud. Mycol. 93: 38. 2019. [MB823772]. — Type: CBS H-23360. Ex-type: CBS 143677 = DTO 228-F7 = IBT 32590 = IBT 32076. Infragen. class: subgen. *Circumdati*, sect. *Flavi*, ser. *Flavi*. Reproduction: asexual. ITS barcode: MG662391 (alternative markers: *BenA* = MG517702; *CaM* = MG518072; *RPB2* = MG517893).

***Aspergillus avenaceus*** G. Sm., Trans. Brit. Mycol. Soc. 26: 24. 1943. [MB284296]. — Type: CBS H-6739. Ex-type: CBS 109.46 = NRRL 517 = ATCC 16861 = IMI 16140 = LCP 89.2592 = LSHBBB 155 = QM 6741 = WB 517. Infragen. class: subgen. *Circumdati*, sect. *Flavi*, ser. *Avenacei*. Reproduction: asexual. ITS barcode: AF104446 (alternative markers: *BenA* = FJ491481; *CaM* = FJ491496; *RPB2* = JN121424).

***Aspergillus baarnensis*** Samson *et al.*, Stud. Mycol. 78: 154. 2014. [MB809579]. Replaced synonym: *Oospora halophila* J.F.H. Beyma, Zentralbl. Bakteriol. Parasitenk., Abt. 2 88: 134. 1933. [MB266778]. — Type: van Beyma 1933, Zentralbl. Bakteriol. Parasitenk., Abt. 2 88: p 134 Fig. 2 (– lectotype designated here, MBT392360; CBS H-24276 [dried culture] – epitype designated here, MBT392361). Ex-epitype: CBS 232.32 = VKM F-204. Infragen. class: subgen. *Polypaecilum*, sect. *Polypaecilum*, ser. *Salinarum*. Reproduction: asexual. ITS barcode: KY980621 (alternative markers: *BenA* = KY980549; *CaM* = KY980585; *RPB2* = JN121509).

***Aspergillus baeticus*** A. Nováková & Hubka, Int. J. Syst. Evol. Microbiol. 62: 2783. 2012. [MB564188]. — Type: PRM 860609. Ex-type: NRRL 62501 = CCF 4226 = CMFISB 2153. Infragen. class: subgen. *Nidulantes*, sect. *Usti*, ser. *Usti*. Reproduction: asexual. ITS barcode: HE615086 (alternative markers: *BenA* = HE615092; *CaM* = HE615117; *RPB2* = HE615124).

***Aspergillus beijingensis*** D.M. Li *et al.*, Mycoscience 39: 299. 1998. [MB446575]. — Type: CBM FD-285. Ex-type: CBM FD-285. Infragen. class: subgen.: unknown, sect.: unknown, ser.: unknown. Reproduction: asexual. ITS barcode: n.a. (alternative markers: *BenA* = n.a.; *CaM* = n.a.; *RPB2* = n.a.).

***Aspergillus bertholletiae*** [as “*bertholletius*”] Taniwaki *et al.*, PLoS ONE 7: e42480, 6. 2012. [MB622229]. — Type: CCT 7615. Ex-type: DTO 223-D3 = ITAL 270/06 = IBT 29228. Infragen. class: subgen. *Circumdati*, sect. *Flavi*, ser. *Bertholletiarum*. Reproduction: protoheterothallic; MAT1-1-1 detected (Carvajal-Campos *et al.* 2017). ITS barcode: JX198673 (alternative markers: *BenA* = MG517689; *CaM* = MN969224; *RPB2* = MG517880).

***Aspergillus bezerrae*** J.P. Andrade *et al.*, Persoonia 42: 379. 2019. [MB830186]. — Type: HURB 22323 (holotype). Ex-type: CCDCA 11511 = 9EM2. Infragen. class: subgen. *Fumigati*, sect. *Fumigati*, ser. *Viridinutantes*. Reproduction: Heterothallic. ITS barcode: n.a. (alternative markers: *BenA* = MK597913; *CaM* = MK597915; *RPB2* = n.a.).

***Aspergillus bicephalus*** J.P.Z. Siqueira *et al.*, Persoonia 37: 283. 2016. [MB818290]. — Type: CBS H-22807. Ex-type: CBS 142900 = FMR 14918. Infragen. class: subgen. *Circumdati*, sect. *Terrei*, ser. *Nivei*. Reproduction: asexual. ITS barcode: LT601380 (alternative markers: *BenA* = LT601381; *CaM* = LT601382; *RPB2* = LT601383).

***Aspergillus bicolor*** M. Chr. & States, Mycologia 70: 337. 1978. [MB309212]. — Type: NY RMF 2058. Ex-type: CBS 425.77 = NRRL 6364 = ATCC 36104 = IMI 216612. Infragen. class: subgen. *Nidulantes*, sect. *Aenei*, ser. *Aenei*. Reproduction: homothallic. ITS barcode: EF652511 (alternative markers: *BenA* = EF652335; *CaM* = EF652423; *RPB2* = EF652247).

***Aspergillus biplanus*** Raper & Fennell, Gen. Aspergillus: 434. 1965. [MB326615]. — Type: IMI 235602. Ex-type: CBS 468.65 = NRRL 5071 = ATCC 16858 = IMI 235602 = QM 8873 = WB 5071. Infragen. class: subgen. *Nidulantes*, sect. *Sparsi*, ser. *Biplani*. Reproduction: asexual. ITS barcode: EF661210 (alternative markers: *BenA* = EF661116; *CaM* = EF661130; *RPB2* = EF661036).

***Aspergillus bisporus*** Kwon-Chung & Fennell, Mycologia 63: 479. 1971. [MB309213]. — Type: BPI NRRL 3693. Ex-type: CBS 707.71 = NRRL 3693 = ATCC 22527 = IMI 350350 = NRRL A-17271 = QM 9700. Infragen. class: subgen. *Nidulantes*, sect. *Bispori*, ser. *Bispori*. Reproduction: asexual. ITS barcode: EF661208 (alternative markers: *BenA* = EF661121; *CaM* = EF661139; *RPB2* = EF661077).

***Aspergillus botswanensis*** A.J. Chen *et al.*, Stud. Mycol. 84: 49. 2016. [MB816095]. — Type: CBS H-22494. Ex-type: CBS 314.89 = DTO 047-I4. Infragen. class: subgen. *Nidulantes*, sect. *Nidulantes*, ser. *Nidulantes*. Reproduction: homothallic; asexual morph unknown. ITS barcode: KU866572 (alternative markers: *BenA* = KU866812; *CaM* = KU866695; *RPB2* = KU866949).

***Aspergillus brasiliensis*** Varga *et al.*, Int. J. Syst. Evol. Microbiol. 57: 57. 2007. [MB510581]. — Type: CBS 101740. Ex-type: CBS 101740 = IMI 381727 = IBT 101740. Infragen. class: subgen. *Circumdati*, sect. *Nigri*, ser. *Nigri*. Reproduction: protoheterothallic; MAT1-2-1 detected (de Vries *et al.* 2017). ITS barcode: FJ629321 (alternative markers: *BenA* = FJ629272; *CaM* = FN594543; *RPB2* = EF661063).

***Aspergillus brevijanus*** S.W. Peterson, Mycologia 100: 217. 2008. [MB506751]. Replaced synonym: *Aspergillus janus* var. *brevis* Raper & Thom Mycologia 36: 561. 1944. [MB351654]. — Type: IMI 16066. Ex-type: CBS 111.46 = NRRL 1935 = ATCC 16828 = CBS 119.45 = IMI 016066ii = IMI 16066 = NCTC 6971 = QM 7417 = WB 1935. Infragen. class: subgen. *Circumdati*, sect. *Janorum*, ser. *Janorum*. Reproduction: asexual. ITS barcode: EF669582 (alternative markers: *BenA* = EU014078; *CaM* = EF669540; *RPB2* = EF669624).

***Aspergillus brevipes*** G. Sm., Trans. Brit. Mycol. Soc. 35: 241. 1952. [MB292837]. — Type: IMI 51494. Ex-type: CBS 118.53 = NRRL 2439 = NRRL 4078 = NRRL 4772 = NRRL A-5521 = ATCC 16899 = IFO 5821 = IMI 51494 = LSHBBB 263 = LSHBSm 242 = QM 1948 = WB 4078 = WB 4224 = WB 4772. Infragen. class: subgen. *Fumigati*, sect. *Fumigati*, ser. *Brevipedes*. Reproduction: asexual. ITS barcode: EF669954 (alternative markers: *BenA* = EF669812; *CaM* = EF669881; *RPB2* = EF669742).

***Aspergillus brevistipitatus*** A. Nováková & Hubka, Fungal Diversity 64: 260. 2014. [MB803934]. — Type: PRM 860543. Ex-type: CBS 135454 = CCF 4149 = CMF ISB 2152 = NRRL 62500 = IFM 60858 = DTO 311-F5. Infragen. class: subgen. *Fumigati*, sect. *Fumigati*, ser. *Neoglabri*. Reproduction: protoheterothallic (Nováková *et al.* 2014). ITS barcode: HF937386 (alternative markers: *BenA* = HF933364; *CaM* = HF933388; *RPB2* = HF937380).

***Aspergillus bridgeri*** M. Chr., Mycologia 74: 210. 1982. [MB110494]. — Type: NY JB 26-1-2. Ex-type: CBS 350.81 = NRRL 13000 = IBT 13380 = ATCC 44562 = IMI 259098. Infragen. class: subgen. *Circumdati*, sect. *Circumdati*, ser. *Sclerotiorum*. Reproduction: asexual. ITS barcode: EF661404 (alternative markers: *BenA* = EF661335; *CaM* = EF661358; *RPB2* = EF661290).

***Aspergillus brunneouniseriatus*** Suj. Singh & B.K. Bakshi, Trans. Brit. Mycol. Soc. 44: 160. 1961. [MB326616]. — Type: IMI 227677. Ex-type: CBS 127.61 = NRRL 4273 = ATCC 16916 = IFO 6993 = IMI 227677 = QM 6990 = WB 4273. Infragen. class: subgen. *Cremei*, sect. *Cremei*, ser. *Brunneouniseriati*. Reproduction: asexual. ITS barcode: EF652141 (alternative markers: *BenA* = EF652123; *CaM* = EF652138; *RPB2* = EF652089).

***Aspergillus brunneoviolaceus*** Bat. & H. Maia, Anais Soc. Biol. Pernambuco 13: 91. 1955. [MB292838]. — Type: IMI 312981. Ex-type: CBS 621.78 = NRRL 4912 = IMI 312981 = WB 4912. Infragen. class: subgen. *Circumdati*, sect. *Nigri*, ser. *Japonici*. Reproduction: asexual. ITS barcode: AJ280003 (alternative markers: *BenA* = EF661105; *CaM* = EF661147; *RPB2* = EF661045).

***Aspergillus brunneus*** Delacr., Bull. Soc. Mycol. Fr. 9: 185. 1893. [MB204832]. — Type: IMI 211378. Ex-type: CBS 112.26 = CBS 524.65 = NRRL 131 = NRRL 134 = ATCC 1021 = IFO 5862 = IMI 211378 = QM 7406 = Thom 4481 = Thom 5633.4 = WB 131. Infragen. class: subgen. *Aspergillus*, sect. *Aspergillus*, ser. *Aspergillus*. Reproduction: homothallic (Delacroix 1893, Chen *et al.* 2017). ITS barcode: EF652060 (alternative markers: *BenA* = EF651907; *CaM* = EF651998; *RPB2* = EF651939).

***Aspergillus caatingaensis*** Y. Horie *et al.*, Mycoscience 55: 84. 2014. [MB801323]. — Type: IFM 61335H. Ex-type: IFM 61335 = CBS 137446 = DTO 278-B3 = DTO 316-F8. Infragen. class: subgen. *Fumigati*, sect. *Fumigati*, ser. *Unilaterales*. Reproduction: homothallic. ITS barcode: MN431362 (alternative markers: *BenA* = AB743854; *CaM* = AB743860; *RPB2* = MN969064).

***Aspergillus caelatus*** B.W. Horn, Mycotaxon 61: 186. 1997. [MB436955]. — Type: BPI 737601. Ex-type: DTO 046-A8 = CBS 763.97 = NRRL 25528 = ATCC 201128. Infragen. class: subgen. *Circumdati*, sect. *Flavi*, ser. *Kitamyces*. Reproduction: protoheterothallic; both idiomorphs detected (Ramirez-Prado *et al.* 2008, unpublished data in GenBank). ITS barcode: AF004930 (alternative markers: *BenA* = EF661470; *CaM* = EF661522; *RPB2* = EF661436).

***Aspergillus caesiellus*** Saito, J. Coll. Sci. Imp. Univ. Tokyo 18: 49. 1904. [MB205025]. — Type: IMI 172278. Ex-type: CBS 470.65 = NRRL 5061 = ATCC 11905 = IFO 4882 = IMI 172278 = WB 5061. Infragen. class: subgen. *Aspergillus*, sect. *Restricti*, ser. *Restricti*. Reproduction: asexual. ITS barcode: EF652044 (alternative markers: *BenA* = EF651884; *CaM* = EF652030; *RPB2* = EF651981).

***Aspergillus caespitosus*** Raper & Thom, Mycologia 36: 563. 1944. [MB284298]. — Type: IMI 16034ii. Ex-type: CBS 103.45 = NRRL 1929 = ATCC 11256 = IMI 16034 = MUCL 13587 = NCTC 6972 = NCTC 6973 = QM 7399 = WB 1929. Infragen. class: subgen. *Nidulantes*, sect. *Nidulantes*, ser. *Stellati*. Reproduction: asexual. ITS barcode: EF652428 (alternative markers: *BenA* = EF652252; *CaM* = EF652340; *RPB2* = EF652164).

***Aspergillus calidoustus*** Varga *et al.*, Eukaryot. Cell 7: 636. 2008. [MB504846]. — Type: Varga *et al.* 2008, Eukaryot. Cell 7: p. 636 Fig. 3 (– lectotype designated here, MBT392259; CBS H-24277 [dried culture] – epitype designated here, MBT392260). Ex-epitype: CBS 121601. Infragen. class: subgen. *Nidulantes*, sect. *Usti*, ser. *Calidousti*. Reproduction: asexual. ITS barcode: HE616558 (alternative markers: *BenA* = FJ624456; *CaM* = HE616559; *RPB2* = MN969061).

***Aspergillus californicus*** Frisvad *et al.*, Stud. Mycol. 69: 91. 2011. [MB560400]. — Type: CBS H-20635. Ex-type: CBS 123895 = IBT 16748 = DTO 061-D4. Infragen. class: subgen. *Nidulantes*, sect. *Cavernicolarum*, ser. *Cavernicolarum*. Reproduction: asexual. ITS barcode: FJ531153 (alternative markers: *BenA* = FJ531180; *CaM* = FJ531128; *RPB2* = MN969065).

***Aspergillus campestris*** M. Chr., Mycologia 74: 212. 1982. [MB110495]. — Type: NY ST 2–3–1. Ex-type: CBS 348.81 = NRRL 13001 = ATCC 44563 = IMI 259099. Infragen. class: subgen. *Circumdati*, sect. *Candidi*, ser. *Candidi*. Reproduction: asexual. ITS barcode: EF669577 (alternative markers: *BenA* = EU014091; *CaM* = EF669535; *RPB2* = EF669619).

***Aspergillus canadensis*** Visagie *et al.*, Stud. Mycol. 88: 187. 2017. [MB818935]. — Type: DAOM 740109. Ex-type: CCF 5548 = KAS 6194 = DTO 356-H9 = IBT 34520 = IBT 34642 = NRRL 66614. Infragen. class: subgen. *Aspergillus*, sect. *Restricti*, ser. *Penicillioides*. Reproduction: asexual. ITS barcode: KY087667 (alternative markers: *BenA* = KY117731; *CaM* = KY068215; *RPB2* = KY117909).

***Aspergillus candidus*** Link, Mag. Ges. Naturf. Freunde Berlin 3: 16. 1809. [MB204868]. — Type: CBS 566.65. Ex-type: CBS 566.65 = NRRL 303 = ATCC 1002 = IMI 16264 = IMI 91889 = LSHBA c .27 = NCTC 595 = QM 1995 = Thom 106 = WB 303. Infragen. class: subgen. *Circumdati*, sect. *Candidi*, ser. *Candidi*. Reproduction: asexual. ITS barcode: EF669592 (alternative markers: *BenA* = EU014089; *CaM* = EF669550; *RPB2* = EF669634).

***Aspergillus caninus*** (Sigler *et al.*) Houbraken *et al.*, Stud. Mycol. 78: 154. 2014. [MB809580]. Basionym: *Phialosimplex caninus* Sigler *et al.*, Med. Mycol. 48: 338. 2010. [MB513393]. — Type: UAMH 10337. Ex-type: CBS 128032 = UAMH 10337 = DTO 139-A6. Infragen. class: subgen. *Polypaecilum*, sect. *Polypaecilum*, ser. *Canini*. Reproduction: asexual. ITS barcode: KY980618 (alternative markers: *BenA* = KY980546; *CaM* = MN969225; *RPB2* = JN121445).

***Aspergillus capensis*** Visagie *et al.*, Stud. Mycol. 78: 105. 2014. [MB809193]. — Type: CBS H-21810. Ex-type: CBS 138188 = DTO 179-E6. Infragen. class: subgen. *Circumdati*, sect. *Flavipedes*, ser. *Flavipedes*. Reproduction: asexual. ITS barcode: KJ775550 (alternative markers: *BenA* = KJ775072; *CaM* = KJ775279; *RPB2* = KP987020).

***Aspergillus caperatus*** A.J. Chen *et al.*, Stud. Mycol. 88: 85. 2017. [MB818733]. — Type: CBS H-22825. Ex-type: CBS 141774 = DTO 337-E6 = IBT 34451. Infragen. class: subgen. *Aspergillus*, sect. *Aspergillus*, ser. *Chevalierorum*. Reproduction: homothallic. ITS barcode: LT670922 (alternative markers: *BenA* = LT671008; *CaM* = LT671009; *RPB2* = LT671010).

***Aspergillus carbonarius*** (Bainier) Thom, J. Agric. Res. 7: 12. 1916. [MB100545]. Basionym: *Sterigmatocystis carbonaria* Bainier, Bull. Soc. Bot. France 27: 27. 1880. [MB195901]. — Type: CBS 556.65. Ex-type: CBS 111.26 = NRRL 369 = ATCC 1025 = IMI 16136 = LSHBA c .11 = NCTC 1325 = NRRL 1987 = QM 331 = Thom 4030.1 = WB 369. Infragen. class: subgen. *Circumdati*, sect. *Nigri*, ser. *Carbonarii*. Reproduction: protoheterothallic; MAT1-2-1 detected (de Vries *et al.* 2017). ITS barcode: EF661204 (alternative markers: *BenA* = EF661099; *CaM* = EF661167; *RPB2* = EF661068).

***Aspergillus carlsbadensis*** Frisvad *et al.*, Stud. Mycol. 69: 88. 2011. [MB560399]. — Type: CBS H-30634. Ex-type: CBS 123894 = IBT 14493 = DTO 061-C7. Infragen. class: subgen. *Nidulantes*, sect. *Usti*, ser. *Calidousti*. Reproduction: asexual. ITS barcode: FJ531151 (alternative markers: *BenA* = FJ531179; *CaM* = FJ531126; *RPB2* = MN969066).

***Aspergillus carneus*** Blochwitz, Ann. Mycol. 31: 81. 1933. [MB259903]. — Type: IMI 1358818. Ex-type: CBS 494.65 = NRRL 527 = ATCC 16798 = IMI 135818 = QM 7401 = Thom 5740.4 = WB 527. Infragen. class: subgen. *Circumdati*, sect. *Terrei*, ser. *Nivei*. Reproduction: asexual. ITS barcode: EF669611 (alternative markers: *BenA* = EF669529; *CaM* = EF669569; *RPB2* = EF669653).

***Aspergillus cavernicola*** Lörinczi, Contrtii bot. Univ. Babes-Bolyai, Cluj, Grad. bot.: 341. 1969. [MB326617]. — Type: CBS 117.76. Ex-type: CBS 117.76 = NRRL 6327. Infragen. class: subgen. *Nidulantes*, sect. *Cavernicolarum*, ser. *Cavernicolarum*. Reproduction: asexual. ITS barcode: EF652508 (alternative markers: *BenA* = EF652332; *CaM* = EF652420; *RPB2* = EF652244).

***Aspergillus cejpii*** (Milko) Samson *et al.*, Stud. Mycol. 78: 155. 2014. [MB809582]. Basionym: *Talaromyces cejpii* Milko, Novosti Sist. Nizsh. Rast. 1: 208. 1964. [MB339918]. — Type: CBS H-7011. Ex-type: CBS 157.66. Infragen. class: subgen. *Fumigati*, sect. *Vargarum*, ser. *Vargarum*. Reproduction: homothallic. ITS barcode: MN431363 (alternative markers: *BenA* = EU076314; *CaM* = MN969226; *RPB2* = JN121447).

***Aspergillus cerealis*** Houbraken *et al.*, Stud. Mycol. 93: 43. 2019. [MB823773]. — Type: CBS H-23359. Ex-type: CBS 143674 = DTO 228-E7 = IBT 32067. Infragen. class: subgen. *Circumdati*, sect. *Flavi*, ser. *Flavi*. Reproduction: protoheterothallic; both MAT idiomorphs detected (Carvajal-Campos *et al.* 2017; referred to as *A*. *korhogoensis*). ITS barcode: MG662394 (alternative markers: *BenA* = MG517693; *CaM* = MG518063; *RPB2* = MG517884).

***Aspergillus cervinus*** Massee, Bull. Misc. Inform. Kew 1914: 158. 1914. [MB211549]. — Type: WIS WISC WT 540. Ex-type: CBS 537.65 = NRRL 5025 = ATCC 16915 = IMI 126542 = QM 8875 = WB 5025. Infragen. class: subgen. *Fumigati*, sect. *Cervini*, ser. *Cervini*. Reproduction: asexual. ITS barcode: EF661268 (alternative markers: *BenA* = EF661251; *CaM* = EF661261; *RPB2* = EF661229).

***Aspergillus chaetosartoryae*** Hubka *et al.*, this study. [MB832557]. Replaced synonym: *Chaetosartorya stromatoides* B.J. Wiley & E.G. Simmons, Mycologia 65: 935. 1973. [MB310956]. — Type: QM 8944. Ex-type: CBS 265.73 = ATCC 24480 = IMI 171880 = NRRL 5501. Infragen. class: subgen. *Cremei*, sect. *Cremei*, ser. *Cremei*. Reproduction: homothallic. ITS barcode: EF652144 (alternative markers: *BenA* = EF652117; *CaM* = EF652129; *RPB2* = EF652099).

***Aspergillus chevalieri*** (L. Mangin) Thom & Church, Aspergilli: 111. 1926. [MB292839]. Basionym: *Eurotium chevalieri* L. Mangin, Ann. Sci. Nat., Bot., ser. 9, 10: 361. 1909. [MB238304]. — Type: IMI 211382. Ex-type: CBS 522.65 = NRRL 78 = ATCC 16443 = IMI 211382 = NRRL A-7803 = Thom 4125.3 = WB 78. Infragen. class: subgen. *Aspergillus*, sect. *Aspergillus*, ser. *Chevalierorum*. Reproduction: homothallic. ITS barcode: EF652068 (alternative markers: *BenA* = EF651911; *CaM* = EF652002; *RPB2* = EF651954).

***Aspergillus chlamydosporus*** (Gené & Guarro) Houbraken *et al.*, Stud. Mycol. 78: 155. 2014. [MB809584]. Basionym: *Sagenomella chlamydospora* Gené & Guarro, J. Clin. Microbiol. 41: 1723. 2003. [MB488173]. — Type: IMI 387422. Ex-type: CBS 109945 = IMI 387422 = FMR 7371 = DTO 138-C2 = UAMH 10961. Infragen. class: subgen. *Polypaecilum*, sect. *Polypaecilum*, ser. *Canini*. Reproduction: asexual. ITS barcode: KY980617 (alternative markers: *BenA* = KY980545; *CaM* = MN969227; *RPB2* = JN121425).

***Aspergillus christenseniae*** A.J. Chen *et al.*, Stud. Mycol. 85: 75. 2016. [MB817724]. — Type: CBS H-9217. Ex-type: CBS 122.56 = DTO 022-C8 = IBT 22043 = IBT 23735 = IMI 343732 = NRRL 4897 = WB 4897. Infragen. class: subgen. *Fumigati*, sect. *Cervini*, ser. *Cervini*. Reproduction: asexual. ITS barcode: FJ491613 (alternative markers: *BenA* = FJ491639; *CaM* = FJ491608; *RPB2* = EF661235).

***Aspergillus chrysellus*** Kwon-Chung & Fennell, Gen. Aspergillus: 424. 1965. [MB326618]. — Type: IMI 238612. Ex-type: CBS 472.65 = NRRL 5084 = ATCC 16852 = IMI 238612 = IMI 238612ii = QM 8876 = WB 5084. Infragen. class: subgen. *Cremei*, sect. *Cremei*, ser. *Wentiorum*. Reproduction: homothallic. ITS barcode: EF652155 (alternative markers: *BenA* = EF652109; *CaM* = EF652136; *RPB2* = EF652090).

***Aspergillus cibarius*** S.B. Hong & Samson, J. Microbiol 50: 713. 2012. [MB800861]. — Type: KACC 46346. Ex-type: DTO 197-D3 = KACC 46346. Infragen. class: subgen. *Aspergillus*, sect. *Aspergillus*, ser. *Aspergillus*. Reproduction: homothallic. ITS barcode: JQ918177 (alternative markers: *BenA* = JQ918180; *CaM* = JQ918183; *RPB2* = JQ918186).

***Aspergillus citocrescens*** Hubka *et al.*, Persoonia 35: 311. 2015. [MB814680]. — Type: PRM 934413. Ex-type: CCF 4001 = CBS 140566 = DTO 376-B3. Infragen. class: subgen. *Cremei*, sect. *Cremei*, ser. *Brunneouniseriati*. Reproduction: asexual. ITS barcode: FR727121 (alternative markers: *BenA* = FR775317; *CaM* = LN878969; *RPB2* = MN969163).

***Aspergillus citrinoterreus*** J. Guinea *et al.*, J. Clin. Microbiol. 53: 612. 2015. [MB810584]. — Type: CBS H-22005. Ex-type: CBS 138921 = GM 228 = DTO 331-H6. Infragen. class: subgen. *Circumdati*, sect. *Terrei*, ser. *Terrei*. Reproduction: asexual. ITS barcode: KP175260 (alternative markers: *BenA* = LN680657; *CaM* = LN680685; *RPB2* = MN969155).

***Aspergillus clavatonanicus*** Bat. *et al.*, Anais Fac. Med. Univ. Recife 15: 197. 1955. [MB292840]. — Type: IMI 235352. Ex-type: CBS 474.65 = NRRL 4741 = ATCC 12413 = DMUR 532 = IMI 235352 = JCM 10183 = QM 7059 = WB 4741. Infragen. class: subgen. *Fumigati*, sect. *Clavati*, ser. *Clavati*. Reproduction: asexual. ITS barcode: EF669986 (alternative markers: *BenA* = EF669842; *CaM* = EF669912; *RPB2* = EF669773).

***Aspergillus clavatophorus*** F. Sklenář *et al.*, Stud. Mycol. 88: 187. 2017. [MB818936]. — Type: PRM 944440. Ex-type: NRRL 25874 = CCF 5454 = IBT 34560 = IBT 34823 = DTO 356-D8. Infragen. class: subgen. *Aspergillus*, sect. *Restricti*, ser. *Penicillioides*. Reproduction: asexual. ITS barcode: KY087772 (alternative markers: *BenA* = KY117836; *CaM* = KY068323; *RPB2* = KY118014).

***Aspergillus clavatus*** Desm., Ann. Sci. Nat., Bot., ser. 2, 2: 71. 1834. [MB211530]. — Type: IMI 15949. Ex-type: CBS 513.65 = NRRL 1 = ATCC 1007 = ATCC 9598 = ATCC 9602 = CECT2674 = DSM 816 = IMI 15949 = LSHBA c .86 = LSHBA c .95 = MIT213 = NCTC 3887 = NCTC 9 = NCTC 978 = NRRL 1656 = QM 1276 = QM 7404 = Thom 107 = WB 1. Infragen. class: subgen. *Fumigati*, sect. *Clavati*, ser. *Clavati*. Reproduction: Heterothallic (Ojeda-López *et al.* 2018). ITS barcode: EF669942 (alternative markers: *BenA* = EF669802; *CaM* = EF669871; *RPB2* = EF669730).

***Aspergillus collembolorum*** Dörfelt & A.R. Schmidt, Mycol. Res. 109: 956, Figs 1–9. 2005. [MB344420]. — Type: Russia: Kaliningrad (Koenigsberg), in succinum Balticum, in exemplare subordines Entomobryomorpha (Collembola), C. & H. W. Hoffeins (coll. Hoffeins, Hamburg, no. 805, holotypus). Ex-type: n.a. Infragen. class: subgen.: unknown, sect.: unknown, ser.: unknown. Reproduction: asexual. ITS barcode: n.a. (alternative markers: *BenA* = n.a.; *CaM* = n.a.; *RPB2* = n.a.).

***Aspergillus collinsii*** Jurjević & S.W. Peterson, Int. J. Syst. Evol. Microbiol. 66: 2570. 2016. [MB814413]. — Type: BPI 893219. Ex-type: CBS 140843 = NRRL 66196 = CCF 5175. Infragen. class: subgen. *Nidulantes*, sect. *Usti*, ser. *Deflecti*. Reproduction: asexual. ITS barcode: KT698845 (alternative markers: *BenA* = KT698843; *CaM* = KT698844; *RPB2* = KT698848).

***Aspergillus coloradensis*** F. Sklenář *et al.*, Mycologia 112: 357. 2020. [MB832715]. — Type: PRM 951699. Ex-type: CCF 6118 = EMSL No. 2726 = NRRL 66888. Infragen. class: subgen. *Nidulantes*, sect. *Aenei*, ser. *Aenei*. Reproduction: homothallic. ITS barcode: MK713539 (alternative markers: *BenA* = MK695646; *CaM* = MK695657; *RPB2* = MK695668).

***Aspergillus conicus*** Blochwitz, Ann. Mycol. 12: 38. 1914. [MB120214]. — Type: IMI 172281. Ex-type: CBS 475.65 = NRRL 149 = ATCC 16908 = IMI 172281 = QM 7405 = Thom 4733.701 = WB 149. Infragen. class: subgen. *Aspergillus*, sect. *Restricti*, ser. *Restricti*. Reproduction: asexual. ITS barcode: EF652039 (alternative markers: *BenA* = EF651881; *CaM* = EF652033; *RPB2* = EF651975).

***Aspergillus conjunctus*** Kwon-Chung & Fennell, Gen. Aspergillus: 552. 1965. [MB326620]. — Type: IMI 135421. Ex-type: CBS 476.65 = NRRL 5080 = ATCC 16796 = IMI 135421 = QM 8878 = WB 5080. Infragen. class: subgen. *Nidulantes*, sect. *Sparsi*, ser. *Conjuncti*. Reproduction: asexual. ITS barcode: EF661179 (alternative markers: *BenA* = EF661111; *CaM* = EF661133; *RPB2* = EF661042).

***Aspergillus contaminans*** Hubka *et al.*, Persoonia 39: 285. 2017. [MB821684]. — Type: PRM 944503. Ex-type: CCF 4682 = CBS 142451 = NRRL 66666. Infragen. class: subgen. *Nidulantes*, sect. *Usti*, ser. *Calidousti*. Reproduction: asexual. ITS barcode: LT594451 (alternative markers: *BenA* = LT594443; *CaM* = LT594425; *RPB2* = LT594434).

***Aspergillus conversis*** Hubka & A. Nováková, Fungal Diversity 64: 262. 2014. [MB803935]. — Type: PRM 860541. Ex-type: CBS 135457 = NRRL 62496 = CCF 4190 = CMF ISB 2151 = IFM 60857. Infragen. class: subgen. *Fumigati*, sect. *Fumigati*, ser. *Neoglabri*. Reproduction: protoheterothallic; MAT 1-2-1 detected (Nováková *et al.* 2014). ITS barcode: HF937385 (alternative markers: *BenA* = HF933363; *CaM* = HF933387; *RPB2* = HF937379).

***Aspergillus coremiiformis*** Bartoli & Maggi, Trans. Brit. Mycol. Soc. 71: 386. 1979 [1978]. [MB309214]. — Type: RO 102 S. Ex-type: CBS 553.77 = NRRL 13603 = ATCC 38576 = IMI 223069 = NRRL 13756. Infragen. class: subgen. *Circumdati*, sect. *Flavi*, ser. *Coremiiformes*. Reproduction: asexual. ITS barcode: EF661544 (alternative markers: *BenA* = EU014104; *CaM* = EU014112; *RPB2* = EU021623).

***Aspergillus corrugatus*** Udagawa & Y. Horie, Mycotaxon 4: 535. 1976. [MB309216]. — Type: NHL 2763. Ex-type: CBS 191.77 = NHL 2763 = DTO 047-I9 = CBM-FA-73. Infragen. class: subgen. *Nidulantes*, sect. *Nidulantes*, ser. *Nidulantes*. Reproduction: homothallic. ITS barcode: KU866574 (alternative markers: *BenA* = KU866814; *CaM* = MN969228; *RPB2* = KU866951).

***Aspergillus costaricensis*** [as “*costaricaensis*”] Samson & Frisvad, Stud. Mycol. 50: 52. 2004. [MB369151]. — Type: CBS H-13437. Ex-type: CBS 115574 = IBT 23401 = CECT 20579 = ITEM 7555. Infragen. class: subgen. *Circumdati*, sect. *Nigri*, ser. *Nigri*. Reproduction: protoheterothallic; unpublished (genome data; Vesth *et al.* 2018). ITS barcode: DQ900602 (alternative markers: *BenA* = FJ629277; *CaM* = FN594545; *RPB2* = HE984361).

***Aspergillus costiformis*** H.Z. Kong & Z.T. Qi, Acta Mycol. Sin. 14: 10. 1995 [MB363444]. — Type: HMAS 62766. Ex-type: CBS 101749 = AS 3.4664. Infragen. class: subgen. *Aspergillus*, sect. *Aspergillus*, ser. *Chevalierorum*. Reproduction: homothallic. ITS barcode: HE615136 (alternative markers: *BenA* = HE801338; *CaM* = HE801320; *RPB2* = HE801309).

***Aspergillus crassihyphae*** Wadhwani & N. Mehrotra, Indian Bot. Reporter: 52. 1985. [MB105070]. — Type: unknown. Ex-type: unknown. Infragen. class: subgen. *Circumdati*, sect. *Nigri*, ser.: unknown. Reproduction: asexual. ITS barcode: n.a. (alternative markers: *BenA* = n.a.; *CaM* = n.a.; *RPB2* = n.a.).

***Aspergillus creber*** Jurjević, S.W. Peterson & B.W. Horn, IMA Fungus 3: 69. 2012. [MB800598]. — Type: BPI 800912. Ex-type: CBS 145749 = NRRL 58592 = DTO 225-G7. Infragen. class: subgen. *Nidulantes*, sect. *Nidulantes*, ser. *Versicolores*. Reproduction: asexual. ITS barcode: JQ301889 (alternative markers: *BenA* = JN853980; *CaM* = JN854043; *RPB2* = JN853832).

***Aspergillus cremeus*** Kwon-Chung & Fennell, Gen. Aspergillus: 418. 1965. [MB326621]. — Type: IMI 123749ii. Ex-type: CBS 477.65 = NRRL 5081 = ATCC 16857 = IMI 123749 = QM 8879 = QM 9191 = WB 5081. Infragen. class: subgen. *Cremei*, sect. *Cremei*, ser. *Cremei*. Reproduction: homothallic. ITS barcode: EF652149 (alternative markers: *BenA* = EF652120; *CaM* = EF652125; *RPB2* = EF652101).

***Aspergillus cretensis*** Frisvad & Samson, Stud. Mycol. 50: 33. 2004. [MB500002]. — Type: CBS H-13446. Ex-type: CBS 112802 = NRRL 35672 = IBT 17505. Infragen. class: subgen. *Circumdati*, sect. *Circumdati*, ser. *Circumdati*. Reproduction: asexual. ITS barcode: FJ491572 (alternative markers: *BenA* = EF661332; *CaM* = FJ491534; *RPB2* = EF661311).

***Aspergillus cristatus*** Raper & Fennell, Gen. Aspergillus: 169. 1965. [MB326622]. — Type: IMI 172280. Ex-type: CBS 123.53 = NRRL 4222 = ATCC 16468 = IMI 172280 = MUCL 15644 = WB 4222. Infragen. class: subgen. *Aspergillus*, sect. *Aspergillus*, ser. *Chevalierorum*. Reproduction: homothallic. ITS barcode: EF652078 (alternative markers: *BenA* = EF651914; *CaM* = EF652001; *RPB2* = EF651957).

***Aspergillus croceiaffinis*** F. Sklenář *et al.*, Mycologia 112: 359. 2020. [MB832713]. — Type: PRM 951576. Ex-type: CCF 6035 = EMSL No. 2282 = NRRL 66887. Infragen. class: subgen. *Nidulantes*, sect. *Nidulantes*, ser. *Unguium*. Reproduction: asexual. ITS barcode: MK713538 (alternative markers: *BenA* = MK695645; *CaM* = MK695656; *RPB2* = MK695667).

***Aspergillus croceus*** Hubka *et al.*, Plant Syst. Evol. 302: 1291. 2016. [MB816281]. — Type: PRM 924053. Ex-type: CCF 4405 = CBS 134396 = NRRL 62495 = IBT 33602. Infragen. class: subgen. *Nidulantes*, sect. *Nidulantes*, ser. *Unguium*. Reproduction: asexual. ITS barcode: LN873931 (alternative markers: *BenA* = LN873944; *CaM* = LN873957; *RPB2* = LN873976).

***Aspergillus crustosus*** Raper & Fennell, Gen. Aspergillus: 532. 1965. [MB326623]. — Type: IMI 135819. Ex-type: CBS 478.65 = NRRL 4988 = ATCC 16806 = IMI 135819 = NRRL A-3254 = QM 8910 = WB 4988. Infragen. class: subgen. *Nidulantes*, sect. *Aenei*, ser. *Aenei*. Reproduction: asexual. ITS barcode: EF652489 (alternative markers: *BenA* = EF652313; *CaM* = EF652401; *RPB2* = EF652225).

***Aspergillus cumulatus*** D.H. Kim & S.B. Hong, J. Microbiol. Biotechnol. 24: 335. 2014. [MB807118]. — Type: KACC 47316. Ex-type: KACC 47316. Infragen. class: subgen. *Aspergillus*, sect. *Aspergillus*, ser. *Rubri*. Reproduction: homothallic. ITS barcode: KF928303 (alternative markers: *BenA* = KF928297; *CaM* = KF928300; *RPB2* = KF928294).

***Aspergillus curviformis*** H.J. Chowdhery & J.N. Rai, Nova Hedwigia 32: 231. 1980. [MB118396]. — Type: unknown. Ex-type: unknown. Infragen. class: subgen. *Fumigati*, sect. *Fumigati*, ser. *Viridinutantes*. Reproduction: asexual. ITS barcode: n.a. (alternative markers: *BenA* = n.a.; *CaM* = n.a.; *RPB2* = n.a.).

***Aspergillus cvjetkovicii*** Jurjević *et al.*, IMA Fungus 3: 69. 2012. [MB800599]. — Type: BPI 880909. Ex-type: NRRL 227 = CBS 599.65. Infragen. class: subgen. *Nidulantes*, sect. *Nidulantes*, ser. *Versicolores*. Reproduction: asexual. ITS barcode: EF652440 (alternative markers: *BenA* = EF652264; *CaM* = EF652352; *RPB2* = EF652176).

***Aspergillus deflectus*** Fennell & Raper, Mycologia 47: 83. 1955. [MB292841]. — Type: IMI 61448. Ex-type: CBS 109.55 = NRRL 2206 = ATCC 16807 = IMI 61448 = NRRL A-2700A = QM 1904 = UC4638 = WB 2206. Infragen. class: subgen. *Nidulantes*, sect. *Usti*, ser. *Deflecti*. Reproduction: asexual. ITS barcode: EF652437 (alternative markers: *BenA* = EF652261; *CaM* = EF652349; *RPB2* = EF652173).

***Aspergillus delicatus*** H.Z. Kong, Mycotaxon 62: 429. 1997. [MB437509]. — Type: HMAS 71159. Ex-type: CBS 101754 = AS 3.4697 = DTO 050-E7. Infragen. class: subgen. *Fumigati*, sect. *Fumigati*, ser. *Thermomutati*. Reproduction: homothallic. ITS barcode: MN431364 (alternative markers: *BenA* = DQ114124; *CaM* = DQ114132; *RPB2* = MN969067).

***Aspergillus denticulatus*** (Samson *et al.*) Samson *et al.*, Stud. Mycol. 78: 155. 2014. [MB809586]. Basionym: *Neosartorya denticulata* Samson *et al.*, Antonie van Leeuwenhoek 93: 95. 2008. [MB506375]. — Type: CBS 652.73. Ex-type: CBS 652.73 = KACC 41183 = DTO 050-D8 = DTO 026-G9. Infragen. class: subgen. *Fumigati*, sect. *Fumigati*, ser. *Fennelliarum*. Reproduction: homothallic. ITS barcode: MN431365 (alternative markers: *BenA* = DQ114125; *CaM* = DQ114133; *RPB2* = MN969068).

***Aspergillus desertorum*** (Samson & Mouch.) Samson *et al.*, Stud. Mycol. 78: 155. 2014. [MB809587]. Basionym: *Emericella desertorum* Samson & Mouch., Antonie van Leeuwenhoek 40: 121. 1974. [MB313502]. — Type: CBS H-7045. Ex-type: CBS 653.73 = NRRL 5921 = IMI 343076. Infragen. class: subgen. *Nidulantes*, sect. *Nidulantes*, ser. *Nidulantes*. Reproduction: homothallic; asexual morph unknown. ITS barcode: EF652505 (alternative markers: *BenA* = EF652329; *CaM* = EF652417; *RPB2* = EF652241).

***Aspergillus destruens*** Zalar *et al.*, Stud. Mycol. 88: 191. 2017. [MB818930]. — Type: PRM 944428. Ex-type: NRRL 145 = IMI 358691 = CCF 5462 = CBS 593.91 = DTO 079-A8 = IBT 34818. Infragen. class: subgen. *Aspergillus*, sect. *Restricti*, ser. *Restricti*. Reproduction: asexual. ITS barcode: KY087748 (alternative markers: *BenA* = KY117811; *CaM* = KY068298; *RPB2* = KY117989).

***Aspergillus dimorphicus*** B.S. Mehrotra & R. Prasad, Trans. Brit. Mycol. Soc. 52: 331. 1969. [MB326625]. — Type: IMI 131553. Ex-type: CBS 649.74 = NRRL 3650 = IMI 131553 = QM 9190. Infragen. class: subgen. *Cremei*, sect. *Cremei*, ser. *Wentiorum*. Reproduction: asexual. ITS barcode: EF652154 (alternative markers: *BenA* = EF652111; *CaM* = EF652135; *RPB2* = EF652096).

***Aspergillus dipodomyus*** F. Sklenář *et al.*, Mycologia 112: 360. 2020. [MB832710]. — Type: PRM 951565. Ex-type: CCF 5265 = NRRL 66273. Infragen. class: subgen. *Nidulantes*, sect. *Nidulantes*, ser. *Nidulantes*. Reproduction: homothallic. ITS barcode: MK713535 (alternative markers: *BenA* = MK695642; *CaM* = MK695653; *RPB2* = MK695664).

***Aspergillus discophorus*** Samson *et al.*, Mycologia 100: 787. 2008. [MB507360]. — Type: CBS H-19889. Ex-type: CBS 469.88 = IBT 21910 = IMI 328717 = DTO 011-B1. Infragen. class: subgen. *Nidulantes*, sect. *Aenei*, ser. *Aenei*. Reproduction: homothallic. ITS barcode: EU448272 (alternative markers: *BenA* = AY339999; *CaM* = EU443970; *RPB2* = MN969069).

***Aspergillus diversus*** Raper & Fennell, Gen. Aspergillus: 437. 1965. [MB326626]. — Type: IMI 232882. Ex-type: CBS 480.65 = NRRL 5074 = ATCC 16849 = IMI 232882 = QM 8882 = WB 5074. Infragen. class: subgen. *Nidulantes*, sect. *Sparsi*, ser. *Biplani*. Reproduction: asexual. ITS barcode: EF661213 (alternative markers: *BenA* = EF661114; *CaM* = EF661128; *RPB2* = EF661034).

***Aspergillus dobrogensis*** A. Nováková *et al.*, Int. J. Syst. Evol. Microbiol. 68: 1004. 2018. [MB821313]. — Type: PRM 935751. Ex-type: CCF 4651 =CCF 4655 = NRRL 62821 = IBT 32697 = CBS 143370. Infragen. class: subgen. *Circumdati*, sect. *Candidi*, ser. *Candidi*. Reproduction: asexual. ITS barcode: LT626959 (alternative markers: *BenA* = LT627027; *CaM* = LT558722; *RPB2* = LT627028).

***Aspergillus domesticus*** F. Sklenář *et al.*, Stud. Mycol. 88: 194. 2017. [MB818931]. — Type: PRM 944426. Ex-type: DTO 079-F2 = CCF 5464 = NRRL 66616 = IBT 34814. Infragen. class: subgen. *Aspergillus*, sect. *Restricti*, ser. *Restricti*. Reproduction: asexual. ITS barcode: KY087688 (alternative markers: *BenA* = KY117752; *CaM* = KY068236; *RPB2* = KY117928).

***Aspergillus dromiae*** A.J. Chen *et al.*, Stud. Mycol. 84: 57. 2016. [MB816089]. — Type: CBS H-22489. Ex-type: CBS 140633 = IBT 25166 = DTO 059-H5. Infragen. class: subgen. *Nidulantes*, sect. *Nidulantes*, ser. *Stellati*. Reproduction: homothallic. ITS barcode: KU866580 (alternative markers: *BenA* = KU866885; *CaM* = KU866703; *RPB2* = KU866958).

***Aspergillus duricaulis*** Raper & Fennell, Gen. Aspergillus: 249. 1965. [MB326627]. — Type: IMI 172282. Ex-type: CBS 481.65 = NRRL 4021 = ATCC 16900 = IMI 172282 = IMI 367413 = NRRL A-5509 = QM 8884 = WB 4021. Infragen. class: subgen. *Fumigati*, sect. *Fumigati*, ser. *Brevipedes*. Reproduction: asexual. ITS barcode: EF669971 (alternative markers: *BenA* = EF669827; *CaM* = EF669897; *RPB2* = EF669758).

***Aspergillus eburneocremeus*** Sappa, Allionia 2: 87. 1954. [MB292842]. — Type: TMI 69856. Ex-type: CBS 130.54 = NRRL 4773 = ATCC 16802 = IMI 69856 = MUCL 13588 = QM 1949 = WB 4773. Infragen. class: subgen. *Nidulantes*, sect. *Aenei*, ser. *Aenei*. Reproduction: asexual. ITS barcode: EF652476 (alternative markers: *BenA* = EF652300; *CaM* = EF652388; *RPB2* = EF652212).

***Aspergillus egyptiacus*** Moub. & Mustafa, Egypt. J. Bot. 15: 153. 1972. [MB344341]. — Type: IMI 141415. Ex-type: CBS 656.73 = NRRL 5920 = ATCC 32114 = IMI 141415. Infragen. class: subgen. *Nidulantes*, sect. *Cavernicolarum*, ser. *Egyptiaci*. Reproduction: asexual. ITS barcode: EF652504 (alternative markers: *BenA* = EF652328; *CaM* = EF652416; *RPB2* = EF652240).

***Aspergillus elegans*** Gasperini, Atti Soc. Tosc. Sci. Nat. Pisa Mem. 8: 328. 1887. [MB212852]. — Type: CBS 102.14. Ex-type: CBS 102.14 = CBS 543.65 = NRRL 4850 = IBT 13505 = ATCC 13829 = ATCC 16886 = IFO 4286 = IMI 133962 = QM 8912 = QM 9373 = WB 4850. Infragen. class: subgen. *Circumdati*, sect. *Circumdati*, ser. *Steyniorum*. Reproduction: asexual. ITS barcode: EF661414 (alternative markers: *BenA* = EF661349; *CaM* = EF661390; *RPB2* = EF661316).

***Aspergillus ellipsoideus*** J.N. Rai & H.J. Chowdhery, Kavaka 7: 17. 1979 [MB116064]. — Type: MLLU 107. Ex-type: unknown. Infragen. class: subgen. *Circumdati*, sect. *Nigri*, ser.: unknown. Reproduction: asexual. ITS barcode: n.a. (alternative markers: *BenA* = n.a.; *CaM* = n.a.; *RPB2* = n.a.).

***Aspergillus ellipticus*** Raper & Fennell, Gen. Aspergillus: 319. 1965. [MB326628]. — Type: CBS 707.79. Ex-type: CBS 482.65 = CBS 707.79 = DTO 035-B7 = NRRL 5120 = ATCC 16876 = IMI 172283 = NRRL 20624 = QM 8886 = WB 5120. Infragen. class: subgen. *Circumdati*, sect. *Nigri*, ser. *Heteromorphi*. Reproduction: asexual. ITS barcode: EF661194 (alternative markers: *BenA* = AY585530; *CaM* = EF661170; *RPB2* = EF661051).

***Aspergillus elongatus*** J.N. Rai & S.C. Agarwal, Canad. J. Bot. 48: 791. 1970. [MB309217]. — Type: CBS 387.75. Ex-type: CBS 387.75 = NRRL 5176 = QM 9702 = WB 5495. Infragen. class: subgen. *Nidulantes*, sect. *Usti*, ser. *Deflecti*. Reproduction: asexual. ITS barcode: EF652502 (alternative markers: *BenA* = EF652326; *CaM* = EF652414; *RPB2* = EF652238).

***Aspergillus elsenburgensis*** Visagie, Stud. Mycol., this issue. 2020. [MB834199]. — Type: PREM 62313. Ex-type: PPRI 2994 = CMV 011G4. Infragen. class: subgen. *Fumigati*, sect. *Fumigati*, ser. *Neoglabri*. Reproduction: homothallic. ITS barcode: MK450651 (alternative markers: *BenA* = MK451215; *CaM* = MK451513; *RPB2* = MK450804).

***Aspergillus endophyticus*** Hubka *et al.*, Stud. Mycol. 88: 95. 2017. [MB818734]. — Type: CBS H-22819. Ex-type: CBS 141766 = DTO 354-I2 = CCF 5345 = IBT 34511. Infragen. class: subgen. *Aspergillus*, sect. *Aspergillus*, ser. *Aspergillus*. Reproduction: homothallic. ITS barcode: LT670941 (alternative markers: *BenA* = LT671067; *CaM* = LT671068; *RPB2* = LT671069).

***Aspergillus eucalypticola*** Varga *et al.*, Stud. Mycol. 69: 9. 2011. [MB560387]. — Type: CBS H-20627. Ex-type: CBS 122712 = IBT 29274. Infragen. class: subgen. *Circumdati*, sect. *Nigri*, ser. *Nigri*. Reproduction: protoheterothallic (genome data, Vesth *et al.* 2018). ITS barcode: EU482439 (alternative markers: *BenA* = EU482435; *CaM* = EU482433; *RPB2* = MN969070).

***Aspergillus europaeus*** Hubka *et al.*, Plant Syst. Evol. 302: 645. 2016. [MB815574]. — Type: PRM 933832. Ex-type: CCF 4409 = CBS 134393 = IBT 32228 = NRRL 66252. Infragen. class: subgen. *Cremei*, sect. *Cremei*, ser. *Wentiorum*. Reproduction: asexual. ITS barcode: LN908996 (alternative markers: *BenA* = LN909006; *CaM* = LN909007; *RPB2* = LT548274).

***Aspergillus falconensis*** Y. Horie *et al.*, Trans. Mycol. Soc. Japan 30: 257. 1989. [MB127891]. — Type: CBM 10001. Ex-type: CBS 271.91 = IFM 4997 = NHL 2999 = ATCC 76117 = DTO 048-A2. Infragen. class: subgen. *Nidulantes*, sect. *Nidulantes*, ser. *Nidulantes*. Reproduction: homothallic. ITS barcode: KU866575 (alternative markers: *BenA* = KU866815; *CaM* = KU866697; *RPB2* = KU866952).

***Aspergillus felis*** Barrs *et al.*, PLoS ONE 8: e64871, 8. 2013. [MB560382]. — Type: CBS H-21125. Ex-type: CBS 130245 = DTO 131-F4 = CCF 5620. Infragen. class: subgen. *Fumigati*, sect. *Fumigati*, ser. *Viridinutantes*. Reproduction: Heterothallic. ITS barcode: MN431358 (alternative markers: *BenA* = MN969363; *CaM* = JX021715; *RPB2* = MN969062).

***Aspergillus fennelliae*** Kwon-Chung & S.J. Kim, Mycologia 66: 629. 1974. [MB309218]. — Type: IMI 278382. Ex-type: AF4 = CBS 599.74 = NRRL 5535 = ATCC 24326 = KACC 41150 (A); AF5 = CBS 598.74 = DTO 046-E8 = NRRL 5534 = ATCC 24325 = KACC 41125 (a). Infragen. class: subgen. *Fumigati*, sect. *Fumigati*, ser. *Fennelliarum*. Reproduction: Heterothallic. ITS barcode: EF669994 (alternative markers: *BenA* = AF057320; *CaM* = EF669920; *RPB2* = EF669781).

***Aspergillus filifer*** [as “*filifera*”] Zalar *et al.*, Mycologia 100: 787. 2008. [MB540309]. — Type: CBS H-19886. Ex-type: CBS 113636 = IBT 23443 = DTO 011-A5. Infragen. class: subgen. *Nidulantes*, sect. *Nidulantes*, ser. *Stellati*. Reproduction: homothallic. ITS barcode: EU448277 (alternative markers: *BenA* = EF428372; *CaM* = EU443973; *RPB2* = KU866932).

***Aspergillus fischeri*** Wehmer, Zentralbl. Bakteriol. Parasitenk, Abt. 2, 18: 390. 1907. [MB202877]. — Type: IMI 21139ii. Ex-type: CBS 544.65 = NRRL 181 = ATCC 1020 = DSM 3700 = IMI 211391 = QM 1983 = Thom 4651.2 = WB 181. Infragen. class: subgen. *Fumigati*, sect. *Fumigati*, ser. *Fumigati*. Reproduction: homothallic. ITS barcode: EF669936 (alternative markers: *BenA* = EF669796; *CaM* = EF669865; *RPB2* = EF669724).

***Aspergillus flaschentraegeri*** Stolk, Trans. Brit. Mycol. Soc. 47: 123. 1964. [MB326629]. — Type: CBS 108.63. Ex-type: CBS 108.63 = NRRL 5042 = ATCC 15535 = IMI 101651 = QM 8889 = WB 5042. Infragen. class: subgen. *Cremei*, sect. *Cremei*, ser. *Wentiorum*. Reproduction: asexual. ITS barcode: EF652150 (alternative markers: *BenA* = EF652113; *CaM* = EF652130; *RPB2* = EF652102).

***Aspergillus flavipes*** (Bainier & Sartory) Thom & Church, Aspergilli: 155. 1926. [MB265045]. Basionym: *Sterigmatocystis flavipes* Bainier & Sartory, Bull. Soc. Mycol. France 27: 90. 1911. [MB452855]. — Type: IMI 171885. Ex-type: NRRL 302 = ATCC 24487 = IMI 171885 = QM 9566 = Thom 4640.474 = WB 302. Infragen. class: subgen. *Circumdati*, sect. *Flavipedes*, ser. *Flavipedes*. Reproduction: asexual; the putative sexual morph of *A*. *flavipes*, *Fennellia flavipes* Wiley & Simmons, represents a different species - *A*. *neoflavipes* (Hubka *et al.* 2015). ITS barcode: EF669591 (alternative markers: *BenA* = EU014085; *CaM* = EF669549; *RPB2* = EF669633).

***Aspergillus flavus*** Link, Mag. Ges. Naturf. Freunde Berlin 3: 16. 1809. [MB209842]. — Type: IMI 124930. Ex-type: CBS 569.65 = NRRL 1957 = ATCC 16883 = IMI 124930 = QM 9947 = WB 1957. Infragen. class: subgen. *Circumdati*, sect. *Flavi*, ser. *Flavi*. Reproduction: Heterothallic (Horn *et al.* 2009a). ITS barcode: AF027863 (alternative markers: *BenA* = EF661485; *CaM* = EF661508; *RPB2* = EF661440).

***Aspergillus floccosus*** (Y.K. Shih) Samson *et al.*, Stud. Mycol. 69: 45. 2011. [MB560393]. Basionym: *Aspergillus terreus* var. *floccosus* Y.K. Shih, Lingnan Sci. J. 15: 372. 1936. [MB499550]. — Type: Unknown. Ex-type: CBS 116.37 = CBS H-24278 = IBT 10846 = IBT 22556 = WB 4872 = DTO 067-B7. Infragen. class: subgen. *Circumdati*, sect. *Terrei*, ser. *Terrei*. Reproduction: asexual. ITS barcode: KP987086 (alternative markers: *BenA* = FJ491714; *CaM* = KP987066; *RPB2* = KP987021).

***Aspergillus floridensis*** Jurjević *et al.*, IMA Fungus 3: 169. 2012. [MB802363]. — Type: BPI 883907. Ex-type: DTO 198-A8 = NRRL 62478 = ITEM 14783. Infragen. class: subgen. *Circumdati*, sect. *Nigri*, ser. *Japonici*. Reproduction: asexual. ITS barcode: MN431366 (alternative markers: *BenA* = HE984412; *CaM* = HE984429; *RPB2* = HE984376).

***Aspergillus foeniculicola*** Udagawa, Trans. Mycol. Soc. Japan 20: 13. 1979. [MB309220]. — Type: NHL 2777. Ex-type: CBS 156.80 = ATCC 42155 = IMI 334933 = LCP 84.2560 = NHL 2777. Infragen. class: subgen. *Nidulantes*, sect. *Aenei*, ser. *Aenei*. Reproduction: homothallic. ITS barcode: EU448274 (alternative markers: *BenA* = EU443990; *CaM* = EU443968; *RPB2* = MN969071).

***Aspergillus foveolatus*** Y. Horie, Trans. Mycol. Soc. Japan 19: 313. 1978. [MB309221]. — Type: IFM 4547. Ex-type: CBS 279.81 = IFM 4547 = NHL 2839 = NBRC 30559 = IFO 30559 = DTO 320-D2. Infragen. class: subgen. *Nidulantes*, sect. *Nidulantes*, ser. *Nidulantes*. Reproduction: homothallic. ITS barcode: KX423658 (alternative markers: *BenA* = KX423622; *CaM* = MN969229; *RPB2* = KU867034).

***Aspergillus frankstonensis*** Barrs *et al.*, PloS ONE 12: e0181660, 8. 2017. [MB819986]. — Type: CBS-H-22969. Ex-type: CBS 142233 = DTO 341-E7. Infragen. class: subgen. *Fumigati*, sect. *Fumigati*, ser. *Viridinutantes*. Reproduction: protoheterothallic; MAT1-2-1 detected (Talbot *et al.* 2017). ITS barcode: KY808756 (alternative markers: *BenA* = KY808594; *CaM* = KY808724; *RPB2* = KY808948).

***Aspergillus fresenii*** Subram., Hyphomycetes: 552. 1971. [MB309222]. Replaced synonym: *Sterigmatocystis sulphurea* Fresen., Beitr. Mykol. 3: 83. 1863. [MB231754]. — Type: IMI 211397. Ex-type: CBS 550.65 = NRRL 4077 = ATCC 16893 = IMI 211397 = NRRL A-5355 = NRRL A-5520 = WB 4077. Infragen. class: subgen. *Circumdati*, sect. *Circumdati*, ser. *Sclerotiorum*. Reproduction: asexual. ITS barcode: EF661409 (alternative markers: *BenA* = EF661341; *CaM* = EF661382; *RPB2* = EF661296).

***Aspergillus fructus*** Jurjević *et al.*, IMA Fungus 3: 70. 2012. [MB800600]. — Type: BPI 880915. Ex-type: NRRL 239 = CBS 584.65. Infragen. class: subgen. *Nidulantes*, sect. *Nidulantes*, ser. *Versicolores*. Reproduction: asexual. ITS barcode: EF652449 (alternative markers: *BenA* = EF652273; *CaM* = EF652361; *RPB2* = EF652185).

***Aspergillus fruticulosus*** Raper & Fennell, Gen. Aspergillus: 506. 1965. [MB326630]. — Type: IMI 139279. Ex-type: CBS 486.65 = NRRL 4903 = ATCC 16823 = IMI 139279 = O-1077 = QM 8033 = WB 4903. Infragen. class: subgen. *Nidulantes*, sect. *Nidulantes*, ser. *Nidulantes*. Reproduction: homothallic. ITS barcode: EF652483 (alternative markers: *BenA* = EF652307; *CaM* = EF652395; *RPB2* = EF652219).

***Aspergillus fumigatiaffinis*** S.B. Hong *et al.*, Mycologia 97: 1326. 2006. [MB500296]. — Type: CBS 117186. Ex-type: CBS 117186 = KACC 41148 = IBT 12703 = IFM 55214. Infragen. class: subgen. *Fumigati*, sect. *Fumigati*, ser. *Fumigati*. Reproduction: protoheterothallic; both MAT idiomorphs detected (Dudová 2014). ITS barcode: MN431367 (alternative markers: *BenA* = DQ094885; *CaM* = DQ094891; *RPB2* = MN969072).

***Aspergillus fumigatus*** Fresen., Beitr. Mykol. 3: 81. 1863. [MB211776]. — Type: IMI 16152. Ex-type: CBS 133.61 = NRRL 163 = ATCC 1022 = ATCC 4813 = IMI 16152 = LSHBA c .71 = NCTC 982 = QM 1981 = Thom 118 = WB 163 = DTO 001-D1. Infragen. class: subgen. *Fumigati*, sect. *Fumigati*, ser. *Fumigati*. Reproduction: Heterothallic (O’Gorman *et al.* 2009). ITS barcode: EF669931 (alternative markers: *BenA* = EF669791; *CaM* = EF669860; *RPB2* = EF669719).

***Aspergillus fumisynnematus*** Y. Horie *et al.*, Trans. Mycol. Soc. Japan 34: 3. 1993. [MB360061]. — Type: CBM FD-0001. Ex-type: DTO 354-A5 = CBS 141446 = IFM 42277. Infragen. class: subgen. *Fumigati*, sect. *Fumigati*, ser. *Fumigati*. Reproduction: protoheterothallic (unpublished data). ITS barcode: AB250779 (alternative markers: *BenA* = AB248076; *CaM* = AB259968; *RPB2* = MN969073).

***Aspergillus funiculosus*** G. Sm., Trans. Brit. Mycol. Soc. 39: 111. 1956. [MB292845]. — Type: IMI 44397. Ex-type: NRRL 4744 = NRRL 2550 = NRRL A-6752. Infragen. class: subgen. *Nidulantes*, sect. *Ochraceorosei*, ser. *Funiculosi*. Reproduction: asexual. ITS barcode: EF661223 (alternative markers: *BenA* = EF661112; *CaM* = EF661175; *RPB2* = EF661078).

***Aspergillus fuscicans*** S.M. Romero *et al.*, Phytotaxa 343: 69. 2018. [MB823159]. — Type: BAFC 52653. Ex-type: BAFCcult 4564!. Infragen. class: subgen. *Nidulantes*, sect. *Usti*, ser. *Calidousti*. Reproduction: asexual. ITS barcode: n.a. (alternative markers: *BenA* = KY853416; *CaM* = KY853415; *RPB2* = n.a.).

***Aspergillus galapagensis*** (Frisvad *et al.*) Samson *et al.*, Stud. Mycol. 78: 155. 2014. [MB809589]. Basionym: *Neosartorya galapagensis* Frisvad *et al.*, Antonie van Leeuwenhoek 93: 96. 2008. [MB506377]. — Type: CBS 117522. Ex-type: CBS 117522 = IBT 16756 = KACC 41935 = DTO 003-H5 = DTO 022-B6. Infragen. class: subgen. *Fumigati*, sect. *Fumigati*, ser. *Neoglabri*. Reproduction: homothallic. ITS barcode: MN431368 (alternative markers: *BenA* = DQ534145; *CaM* = DQ534151; *RPB2* = MN969074).

***Aspergillus germanicus*** Frisvad *et al.*, Stud. Mycol. 69: 91. 2011. [MB560401]. — Type: CBS H-20636. Ex-type: CBS 123887 = DTO 027-D9. Infragen. class: subgen. *Nidulantes*, sect. *Usti*, ser. *Calidousti*. Reproduction: asexual. ITS barcode: FJ531146 (alternative markers: *BenA* = FJ531172; *CaM* = FJ531141; *RPB2* = KU866944).

***Aspergillus giganteus*** Wehmer, Mem. Soc. Phys. Genève 33: 85. 1901. [MB206765]. — Type: IMI 227678. Ex-type: CBS 526.65 = NRRL 10 = ATCC 10059 = DSM 1146 = IFO 5818 = IMI 227678 = QM 1970 = Thom 5581.13A = WB 10. Infragen. class: subgen. *Fumigati*, sect. *Clavati*, ser. *Clavati*. Reproduction: asexual. ITS barcode: EF669928 (alternative markers: *BenA* = EF669789; *CaM* = EF669857; *RPB2* = EF669716).

***Aspergillus glabripes*** F. Sklenář *et al.*, Stud. Mycol. 88: 197. 2017. [MB818934]. — Type: PRM 944436. Ex-type: CCF 5474 = DTO 356-E8 = EMSL No. 2462 = NRRL 66618 = IBT 34820. Infragen. class: subgen. *Aspergillus*, sect. *Restricti*, ser. *Vitricolarum*. Reproduction: asexual. ITS barcode: KY087614 (alternative markers: *BenA* = KY117683; *CaM* = KY068166; *RPB2* = KY117859).

***Aspergillus glaucus*** (L.) Link, Mag. Ges. Naturf. Freunde Berlin 3: 16. 1809. [MB161735]. Basionym: *Mucor glaucus* L., Sp. Pl. 2: 1186. 1753. [MB185847]. — Type: IMI 211383. Ex-type: CBS 516.65 = NRRL 116 = ATCC 16469 = IMI 211383 = LCP 64.1859 = Thom 5629.C = WB 116. Infragen. class: subgen. *Aspergillus*, sect. *Aspergillus*, ser. *Aspergillus*. Reproduction: homothallic (Link 1809, Chen *et al.* 2017). ITS barcode: EF652052 (alternative markers: *BenA* = EF651887; *CaM* = EF651989; *RPB2* = EF651934).

***Aspergillus gorakhpurensis*** Kamal & Bhargava, Trans. Brit. Mycol. Soc. 52: 338. 1969. [MB326632]. — Type: IMI 130728. Ex-type: CBS 648.74 = NRRL 3649 = IMI 130728 = QM 9187 = WB 5346. Infragen. class: subgen. *Cremei*, sect. *Cremei*, ser. *Cremei*. Reproduction: asexual. ITS barcode: EF652145 (alternative markers: *BenA* = EF652114; *CaM* = EF652126; *RPB2* = EF652097).

***Aspergillus gracilis*** Bainier, Bull. Soc. Mycol. France 23: 90. 1907. [MB167554]. — Type: IMI 211393. Ex-type: CBS 539.65 = NRRL 4962 = ATCC 16906 = IMI 211393 = QM 8915 = WB 4962. Infragen. class: subgen. *Aspergillus*, sect. *Restricti*, ser. *Restricti*. Reproduction: asexual. ITS barcode: EF652045 (alternative markers: *BenA* = EF651883; *CaM* = EF652031; *RPB2* = EF651980).

***Aspergillus granulosus*** Raper & Thom, Mycologia 36: 565. 1944. [MB284302]. — Type: IMI 17278ii. Ex-type: NRRL 1932 = ATCC 16837 = IMI 17278 = QM 6846 = WB 1932. Infragen. class: subgen. *Nidulantes*, sect. *Usti*, ser. *Usti*. Reproduction: asexual. ITS barcode: EF652430 (alternative markers: *BenA* = EF652254; *CaM* = EF652342; *RPB2* = EF652166).

***Aspergillus griseoaurantiacus*** Visagie *et al.*, Stud. Mycol. 78: 112. 2014. [MB809197]. — Type: CBS H-21814. Ex-type: CBS 138191 = DTO 267-D8. Infragen. class: subgen. *Nidulantes*, sect. *Nidulantes*, ser. *Versicolores*. Reproduction: asexual. ITS barcode: KJ775553 (alternative markers: *BenA* = KJ775086; *CaM* = KJ775357; *RPB2* = KU866988).

***Aspergillus haitiensis*** Varga *et al.*, IMA Fungus 1: 194. 2010. [MB517384]. — Type: CBS H-20503. Ex-type: CBS 464.91. Infragen. class: subgen. *Nidulantes*, sect. *Sparsi*, ser. *Sparsi*. Reproduction: asexual. ITS barcode: FJ491657 (alternative markers: *BenA* = FJ491670; *CaM* = FJ491645; *RPB2* = KU866943).

***Aspergillus halophilicus*** C.M. Chr. *et al.*, Mycologia 51: 636. 1961. [MB326633]. — Type: BPI 566153. Ex-type: CBS 122.62 = NRRL 2739 = ATCC 16401 = IFO 7054 = IMI 211802 = NRRL 4679 = NRRL A-7206 = QM 8894 = WB 4679. Infragen. class: subgen. *Aspergillus*, sect. *Restricti*, ser. *Halophilici*. Reproduction: homothallic. ITS barcode: EF652088 (alternative markers: *BenA* = EF651926; *CaM* = EF652034; *RPB2* = EF651982).

***Aspergillus hancockii*** Pitt, PLoS ONE 12: e0170254, 16. 2017. [MB818219]. — Type: FRR 3425. Ex-type: CBS 142004 = DTO 360-G7. Infragen. class: subgen. *Circumdati*, sect. *Flavi*, ser. *Leporum*. Reproduction: asexual. ITS barcode: KX858342 (alternative markers: *BenA* = MBFL01001228.1:26000-28000; *CaM* = MBFL01000377.1:5000-7000; *RPB2* = MBFL01000137:9000-11000).

***Aspergillus heldtiae*** Visagie, Stud. Mycol., this issue. 2020. [MB834200]. — Type: PREM 50864. Ex-type: PPRI 4229 = CMV 004A2. Infragen. class: subgen. *Circumdati*, sect. *Terrei*, ser. *Terrei*. Reproduction: asexual. ITS barcode: MK450656 (alternative markers: *BenA* = MK450981; *CaM* = MK451518; *RPB2* = MK450809).

***Aspergillus heteromorphus*** Bat. & H. Maia, Anais Soc. Biol. Pernambuco 15: 200. 1957. [MB292846]. — Type: IMI 172288. Ex-type: CBS 117.55 = NRRL 4747 = ATCC 12064 = IMI 172288 = QM 6954 = WB 4747. Infragen. class: subgen. *Circumdati*, sect. *Nigri*, ser. *Heteromorphi*. Reproduction: protoheterothallic; unpublished (genome data, Vesth *et al.* 2018). ITS barcode: EU821305 (alternative markers: *BenA* = EF661103; *CaM* = EF661169; *RPB2* = EF661050).

***Aspergillus heterothallicus*** Kwon-Chung *et al.*, Gen. Aspergillus: 502. 1965. [MB326635]. — Type: CBS 488.65. Ex-type: CBS 488.65 = NRRL 5096 = ATCC 16847 = IMI 139277 = QM 8916 = WB 5096. Infragen. class: subgen. *Nidulantes*, sect. *Usti*, ser. *Usti*. Reproduction: Heterothallic. ITS barcode: EF652499 (alternative markers: *BenA* = EF652323; *CaM* = EF652411; *RPB2* = EF652235).

***Aspergillus heyangensis*** Z.T. Qi *et al.*, Acta Mycol. Sin. 13: 81. 1994. [MB414654]. — Type: HMAS 58982. Ex-type: CBS 101751 = AS 3.4630 = DTO 026-G6. Infragen. class: subgen. *Nidulantes*, sect. *Aenei*, ser. *Aenei*. Reproduction: asexual. ITS barcode: FJ491520 (alternative markers: *BenA* = FJ491521; *CaM* = FJ491522; *RPB2* = KX423659).

***Aspergillus hiratsukae*** Udagawa *et al.*, Trans. Mycol. Soc. Japan 32: 23. 1991. [MB354908]. — Type: NHL 3008. Ex-type: CBS 294.93 = NRRL 20820 = IMI 349859 = NHL 3008 = DTO 050-E5. Infragen. class: subgen. *Fumigati*, sect. *Fumigati*, ser. *Unilaterales*. Reproduction: homothallic. ITS barcode: MN431369 (alternative markers: *BenA* = AF057324; *CaM* = AY870699; *RPB2* = MN969075).

***Aspergillus homomorphus*** Steiman *et al.* ex Samson & Frisvad, Stud. Mycol. 50: 58. 2004. [MB500011]. — Type: CBS H-13440. Ex-type: CBS 101889 = ITEM 7556. Infragen. class: subgen. *Circumdati*, sect. *Nigri*, ser. *Homomorphi*. Reproduction: protoheterothallic; unpublished (genome data, Vesth *et al.* 2018). ITS barcode: EF166063 (alternative markers: *BenA* = AY820015; *CaM* = FN594549; *RPB2* = MN969076).

***Aspergillus hongkongensis*** C.C. Tsang *et al.*, Diagnostic Microbiology and Infectious Disease 84: 130. 2016. [MB810279]. — Type: NBRC H-13268. Ex-type: CBS 145671 = HKU49 = NBRC 110693 = NCPF 7870 = BCRC FU30360 = DTO 351-C3. Infragen. class: subgen. *Nidulantes*, sect. *Nidulantes*, ser. *Versicolores*. Reproduction: asexual. ITS barcode: AB987907 (alternative markers: *BenA* = LC000552; *CaM* = MN969320; *RPB2* = LC000578).

***Aspergillus hordei*** F. Sklenář *et al.*, Stud. Mycol. 88: 207. 2017. [MB818937]. — Type: PRM 944446. Ex-type: NRRL 25825 = CCF 5483 = DTO 356-D3 = IBT 34539. Infragen. class: subgen. *Aspergillus*, sect. *Restricti*, ser. *Penicillioides*. Reproduction: asexual. ITS barcode: KY087759 (alternative markers: *BenA* = KY117822; *CaM* = KY068309; *RPB2* = KY118000).

***Aspergillus hortae*** [as “*hortai*”] (Langeron) C.W. Dodge, Medic. Mycol.: 628. 1935. [MB252620]. Basionym: *Sterigmatocystis hortae* Langeron, Bull. Soc. Pathol. Exot.: 383. 1922. [MB252621]. — Type: Langeron 1922, Bull. Soc. Path. Exot. 15: p. 384, Fig. 1 (– lectotype designated here, MBT392292; CBS H-24279 [dried culture] – epitype designated here, MBT392293). Ex-epitype: CBS 124230 = NRRL 274 = ATCC 10070 = IBT 26384 = DTO 051-D6. Infragen. class: subgen. *Circumdati*, sect. *Terrei*, ser. *Terrei*. Reproduction: asexual. ITS barcode: KP987087 (alternative markers: *BenA* = FJ491706; *CaM* = KP987054; *RPB2* = KP987022).

***Aspergillus huiyaniae*** Y. Horie *et al.*, Mycoscience 55: 218. 2014. [MB803656]. — Type: IFM 57847H. Ex-type: IFM 57847 = JCM 19448 = CBS 139185 = DTO 316-F5. Infragen. class: subgen. *Fumigati*, sect. *Fumigati*, ser. *Fennelliarum*. Reproduction: homothallic. ITS barcode: MN431370 (alternative markers: *BenA* = AB787219; *CaM* = AB787564; *RPB2* = MN969077).

***Aspergillus ibericus*** R. Serra *et al.*, Mycologia 98: 298. 2006. [MB501326]. — Type: MUM-H 03.49. Ex-type: NRRL 35644. Infragen. class: subgen. *Circumdati*, sect. *Nigri*, ser. *Carbonarii*. Reproduction: protoheterothallic; unpublished (genome data, Vesth *et al.* 2018). ITS barcode: EF661200 (alternative markers: *BenA* = EF661102; *CaM* = EF661163; *RPB2* = EF661065).

***Aspergillus iizukae*** Sugiy., J. Fac. Sci. Univ. Tokyo, Sect. 3, Bot. 9: 390. 1967. [MB326636]. — Type: TI 0007. Ex-type: CBS 541.69 = NRRL 3750 = IMI 141552 = QM 9325. Infragen. class: subgen. *Circumdati*, sect. *Flavipedes*, ser. *Flavipedes*. Reproduction: asexual. ITS barcode: EF669597 (alternative markers: *BenA* = EU014086; *CaM* = EF669555; *RPB2* = EF669639).

***Aspergillus implicatus*** Persiani & Maggi, Mycol. Res. 98: 871. 1994. [MB362533]. — Type: ROHB 110 S. Ex-type: CBS 484.95. Infragen. class: subgen. *Nidulantes*, sect. *Sparsi*, ser. *Implicati*. Reproduction: asexual. ITS barcode: FJ491656 (alternative markers: *BenA* = FJ491667; *CaM* = FJ491650; *RPB2* = MN969078).

***Aspergillus incahuasiensis*** E. Piontelli *et al.*, Int. J. Syst. Evol. Microbiol. 69: 3354. 2019. [MB828145]. — Type: BPI 910732. Ex-type: NRRL 66825. Infragen. class: subgen. *Nidulantes*, sect. *Nidulantes*, ser. *Multicolores*. Reproduction: asexual. ITS barcode: MH473585 (alternative markers: *BenA* = MH476273; *CaM* = MH476276; *RPB2* = MH476279).

***Aspergillus indologenus*** Frisvad *et al.*, Stud. Mycol. 69: 9. 2011. [MB560389]. — Type: CBS H-20629. Ex-type: CBS 114.80 = IBT 3679 = ITEM 7038. Infragen. class: subgen. *Circumdati*, sect. *Nigri*, ser. *Japonici*. Reproduction: asexual. ITS barcode: AJ280005 (alternative markers: *BenA* = AY585539; *CaM* = AM419750; *RPB2* = HE984366).

***Aspergillus inflatus*** (Stolk & Malla) Samson *et al.*, Stud. Mycol. 78: 155. 2014. [MB809590]. Basionym: *Penicillium inflatum* Stolk & Malla, Persoonia 6: 197. 1971. [MB319276]. — Type: CBS H-7500. Ex-type: CBS 682.70 = FRR 1549 = IMI 191498. Infragen. class: subgen. *Cremei*, sect. *Cremei*, ser. *Inflati*. Reproduction: asexual. ITS barcode: FJ531054 (alternative markers: *BenA* = FJ531008; *CaM* = FJ531090; *RPB2* = JN406529).

***Aspergillus infrequens*** F. Sklenář *et al.*, Stud. Mycol. 88: 207. 2017. [MB818938]. — Type: PRM 944449. Ex-type: NRRL 25868 = CCF 5486 = DTO 356-D6 = IBT 34524. Infragen. class: subgen. *Aspergillus*, sect. *Restricti*, ser. *Penicillioides*. Reproduction: asexual. ITS barcode: KY087770 (alternative markers: *BenA* = KY117833; *CaM* = KY068320; *RPB2* = KY118011).

***Aspergillus insolitus*** (G. Sm.) Houbraken *et al.*, Stud. Mycol. 78: 155. 2014. [MB809591]. Basionym: *Polypaecilum insolitum* G. Sm., Trans. Brit. Mycol. Soc. 44: 437. 1961. [MB337467]. — Type: CBS 384.61. Ex-type: CBS 384.61 = ATCC 18164 = IFO 8788 = IMI 75202 = LSHB BB414 = MUCL 3078 = QM 7961 = DTO 049-I6. Infragen. class: subgen. *Polypaecilum*, sect. *Polypaecilum*, ser. *Polypaecilum*. Reproduction: asexual. ITS barcode: KY980622 (alternative markers: *BenA* = KY980550; *CaM* = MN969230; *RPB2* = JN121510).

***Aspergillus insuetus*** (Bainier) Thom & Church, Manual of the Aspergilli: 153. 1929. [MB267997]. Basionym: *Sterigmatocystis insueta* Bainier, Bull. Soc. Mycol. France 24: 85. 1908. [MB218947]. — Type: CBS 107.25. Ex-type: CBS 107.25 = NRRL 279 = NRRL 1726 = ATCC 1033 = IFO 4128. Infragen. class: subgen. *Nidulantes*, sect. *Usti*, ser. *Calidousti*. Reproduction: asexual. ITS barcode: EF652457 (alternative markers: *BenA* = EF652281; *CaM* = EF652369; *RPB2* = EF652193).

***Aspergillus insulicola*** Montem. & A.R. Santiago, Mycopathol. 55: 130. 1975. [MB309225]. — Type: CBS 382.75. Ex-type: CBS 382.75 = NRRL 6138 = ATCC 26220. Infragen. class: subgen. *Circumdati*, sect. *Circumdati*, ser. *Steyniorum*. Reproduction: asexual. ITS barcode: EF661430 (alternative markers: *BenA* = EF661353; *CaM* = EF661396; *RPB2* = EF661286).

***Aspergillus intermedius*** Blaser, Sydowia 28: 41. 1976. [MB309226]. — Type: IMI 89278. Ex-type: CBS 523.65 = NRRL 82 = ATCC 16444 = DSM 2830 = IMI 089278ii = IMI 89278 = LSHBBB 107 = LSHTM 107 = QM 7403 = Thom 5612.107 = WB 82. Infragen. class: subgen. *Aspergillus*, sect. *Aspergillus*, ser. *Chevalierorum*. Reproduction: homothallic. ITS barcode: EF652074 (alternative markers: *BenA* = EF651892; *CaM* = EF652012; *RPB2* = EF651958).

***Aspergillus iranicus*** Arzanlou *et al.*, Mycol. Prog. 15: 1085. 2016. [MB817473]. — Type: CBS H-22338. Ex-type: CCTU 756 = CBS 139561 = IBT 32596 = DTO 203-D7. Infragen. class: subgen. *Circumdati*, sect. *Terrei*, ser. *Nivei*. Reproduction: asexual. ITS barcode: KP987077 (alternative markers: *BenA* = KP987045; *CaM* = KP987060; *RPB2* = KP987034).

***Aspergillus israelensis*** A.J. Chen *et al.*, Stud. Mycol. 84: 63. 2016. [MB816091]. — Type: CBS H-22491. Ex-type: CBS 140627 = IBT 24293 = DTO 325-E2. Infragen. class: subgen. *Nidulantes*, sect. *Nidulantes*, ser. *Unguium*. Reproduction: asexual. ITS barcode: KU866677 (alternative markers: *BenA* = KU866915; *CaM* = KU866797; *RPB2* = KU867062).

***Aspergillus itaconicus*** Kinosh., Bot. Mag. (Tokyo) 45: 60. 1931. [MB268225]. — Type: IMI 16119. Ex-type: CBS 115.32 = NRRL 161 = ATCC 10021 = IHEM 4378 = IMI 16119 = LSHBA 48 = MUCL 31306 = QM 1980 = Thom 5344 = Thom 5660.48 = WB 161. Infragen. class: subgen. *Cremei*, sect. *Cremei*, ser. *Cremei*. Reproduction: asexual. ITS barcode: EF652147 (alternative markers: *BenA* = EF652118; *CaM* = EF652140; *RPB2* = EF652103).

***Aspergillus ivoriensis*** Bartoli & Maggi, Trans. Brit. Mycol. Soc. 71: 383. 1979 [1978]. [MB309228]. — Type: RO 101 S. Ex-type: CBS 551.77 = NRRL 22883. Infragen. class: subgen. *Nidulantes*, sect. *Raperorum*, ser. *Raperorum*. Reproduction: asexual. ITS barcode: EF652441 (alternative markers: *BenA* = EF652265; *CaM* = EF652353; *RPB2* = EF652177).

***Aspergillus jaipurensis*** Samson *et al.*, Stud. Mycol. 78: 155. 2014. [MB809592]. Replaced synonym: *Emericella indica* Stchigel & Guarro, Mycol. Res. 103: 1059. 1999. [MB460081]. — Type: IMI 378525. Ex-type: IMI 378525 = DTO 320-A9 = FMR 6232 = CBS 952.97. Infragen. class: subgen. *Nidulantes*, sect. *Nidulantes*, ser. *Nidulantes*. Reproduction: homothallic. ITS barcode: MN431371 (alternative markers: *BenA* = AY339988; *CaM* = KU866761; *RPB2* = KU867024).

***Aspergillus janus*** Raper & Thom, Mycologia 36: 556. 1944. [MB284303]. — Type: IMI 16065. Ex-type: CBS 118.45 = NRRL 1787 = IMI 16065 = NCTC 6970. Infragen. class: subgen. *Circumdati*, sect. *Janorum*, ser. *Janorum*. Reproduction: asexual. ITS barcode: EF669578 (alternative markers: *BenA* = EU014076; *CaM* = EF669536; *RPB2* = EF669620).

***Aspergillus japonicus*** Saito, Bot. Mag. (Tokyo) 20: 61. 1906. [MB160656]. — Type: CBS 114.51. Ex-type: CBS 114.51 = ITEM 7034. Infragen. class: subgen. *Circumdati*, sect. *Nigri*, ser. *Japonici*. Reproduction: protoheterothallic; unpublished (genome data, Vesth *et al.* 2018). ITS barcode: AJ279985 (alternative markers: *BenA* = HE577804; *CaM* = FN594551; *RPB2* = MN969079).

***Aspergillus jensenii*** Jurjević *et al.*, IMA Fungus 3: 70. 2012. [MB800601]. — Type: BPI 880910. Ex-type: NRRL 58600. Infragen. class: subgen. *Nidulantes*, sect. *Nidulantes*, ser. *Versicolores*. Reproduction: asexual. ITS barcode: JQ301892 (alternative markers: *BenA* = JN854007; *CaM* = JN854046; *RPB2* = JN853835).

***Aspergillus kalimae*** Tanney *et al.*, Stud. Mycol. 88: 249. 2017. [MB822732]. — Type: DAOM 745800. Ex-type: DAOMC 251762 = UAMH 11837 = CBS 143506 = KAS 8135 = SLOAN 4181 = PN08TH-526. Infragen. class: subgen. *Polypaecilum*, sect. *Polypaecilum*, ser. *Kalimarum*. Reproduction: asexual. ITS barcode: KY980650 (alternative markers: *BenA* = KY980578; *CaM* = KY980614; *RPB2* = KY980475).

***Aspergillus kanagawaensis*** Nehira, J. Jap. Bot. 26: 109. 1951. [MB292847]. — Type: IMI 126690. Ex-type: CBS 538.65 = NRRL 4774 = NRRL 2854 = NRRL 3156 = NRRL A-13499 = IMI 126690. Infragen. class: subgen. *Fumigati*, sect. *Cervini*, ser. *Cervini*. Reproduction: asexual. ITS barcode: EF661275 (alternative markers: *BenA* = EF661239; *CaM* = EF661263; *RPB2* = EF661236).

***Aspergillus karnatakaensis*** Varga *et al.*, IMA Fungus 1: 203. 2010. [MB517549]. — Type: CBS H-20502. Ex-type: CBS 102800 = IBT 22153. Infragen. class: subgen. *Nidulantes*, sect. *Aenei*, ser. *Aenei*. Reproduction: asexual. ITS barcode: EU482441 (alternative markers: *BenA* = EU482438; *CaM* = EU482431; *RPB2* = KU866956).

***Aspergillus kassunensis*** Baghd., Novosti. Sist. Nizsh. Rast., 1968 5: 113. 1968. [MB326639]. — D10 in Universitate Mosquensi (holotype); CBS H-24320 (isotype). Ex-type: CBS 419.69 = NRRL 3752 = IMI 334938. Infragen. class: subgen. *Nidulantes*, sect. *Cavernicolarum*, ser. *Cavernicolarum*. Reproduction: asexual. ITS barcode: EF652461 (alternative markers: *BenA* = EF652285; *CaM* = EF652373; *RPB2* = EF652197).

***Aspergillus keratitidis*** (W.L. Chen *et al.*) Zalar & W.L. Chen. Extremophiles 21: 762. 2017. [MB818566]. Basionym: *Sagenomella keratitidis* W.L. Chen *et al.*, Bot. Stud. 50: 332. 2009. [MB541734]. — Type: BCRC 34221. Ex-type: BCRC 34221 = DTO 198-E8. Infragen. class: subgen. *Polypaecilum*, sect. *Polypaecilum*, ser. *Noonimiarum*. Reproduction: asexual. ITS barcode: KY980616 (alternative markers: *BenA* = KY980544; *CaM* = KY980580; *RPB2* = KY980443).

***Aspergillus keveii*** Varga *et al.*, Stud. Mycol. 59: 120. 2007. [MB505570]. — Type: CBS 209.92. Ex-type: CBS 209.92 = DTO 013-G8. Infragen. class: subgen. *Nidulantes*, sect. *Usti*, ser. *Calidousti*. Reproduction: asexual. ITS barcode: EU076354 (alternative markers: *BenA* = EU076376; *CaM* = EU076365; *RPB2* = KU866938).

***Aspergillus keveioides*** L. Wang, Mycosystema 32 (suppl.): 139. 2013. [MB800250]. — Type: HMAS242394. Ex-type: AS 3.15305 = CBS 132737 = DTO 328-D7. Infragen. class: subgen. *Nidulantes*, sect. *Usti*, ser. *Calidousti*. Reproduction: asexual. ITS barcode: JN982704 (alternative markers: *BenA* = JN982694; *CaM* = JN982684; *RPB2* = MN969151).

***Aspergillus koreanus*** Hyang B. Lee *et al.*, Fungal Diversity 80: 142. 2016. [MB816938]. — Type: NIBR EML-GSNP1-1. Ex-type: JMRC:SF:012334. Infragen. class: subgen. *Cremei*, sect. *Cremei*, ser. *Inflati*. Reproduction: asexual. ITS barcode: KX216525 (alternative markers: *BenA* = KX216530; *CaM* = KX216528; *RPB2* = KX216531).

***Aspergillus krugeri*** Visagie, Stud. Mycol., this issue. 2020. [MB834203]. — Type: PREM 62309. Ex-type: PPRI 8986 = CMV 006G4. Infragen. class: subgen. *Circumdati*, sect. *Flavi*, ser. *Flavi*. Reproduction: asexual. ITS barcode: MK450655 (alternative markers: *BenA* = MK451098; *CaM* = MK451517; *RPB2* = MK450808).

***Aspergillus labruscus*** Fungaro *et al.*, Sci. Rep. 7: 6203, 4. 2017. [MB815746]. — Type: CCT 7800. Ex-type: DTO 357-D4 = ITAL 22.223 = IBT 33586. Infragen. class: subgen. *Circumdati*, sect. *Nigri*, ser. *Japonici*. Reproduction: asexual. ITS barcode: KU708544 (alternative markers: *BenA* = KT986014; *CaM* = KT986008; *RPB2* = MN969196).

***Aspergillus laciniosus*** S.B. Hong *et al.*, Int. J. Syst. Evol. Microbiol. 56: 484. 2006. [MB521269]. — Type: CBS 117721. Ex-type: CBS 117721 = NRRL 35589 = KACC 41657 = DTO 164-I2. Infragen. class: subgen. *Fumigati*, sect. *Fumigati*, ser. *Fumigati*. Reproduction: homothalliic. ITS barcode: AB299413 (alternative markers: *BenA* = AY870756; *CaM* = AY870716; *RPB2* = MN969080).

***Aspergillus lanosus*** Kamal & Bhargava, Trans. Brit. Mycol. Soc. 52: 336. 1969. [MB326640]. — Type: IMI 130727. Ex-type: CBS 650.74 = DTO 034-B7 = NRRL 3648 = IMI 130727 = QM 9183 = WB 5347. Infragen. class: subgen. *Circumdati*, sect. *Flavi*, ser. *Alliacei*. Reproduction: asexual. ITS barcode: EF661553 (alternative markers: *BenA* = MG517633; *CaM* = MG518017; *RPB2* = EU021642).

***Aspergillus latilabiatus*** A.J. Chen *et al.*, Stud. Mycol. 84: 66. 2016. [MB816093]. — Type: CBS H-22514. Ex-type: CBS 426.93 = IBT 33959 = DTO 320-B2. Infragen. class: subgen. *Nidulantes*, sect. *Nidulantes*, ser. *Nidulantes*. Reproduction: homothallic; asexual morph unknown. ITS barcode: KU866624 (alternative markers: *BenA* = KU866864; *CaM* = KU866762; *RPB2* = KU867025).

***Aspergillus lentulus*** Balajee & K.A. Marr, Eukaryot. Cell 4: 631. 2005. [MB356679]. — Type: BPI 863540. Ex-type: CBS 117885 = NRRL 35552 = IBT 27201 = KACC 41940 = DTO 004-E9. Infragen. class: subgen. *Fumigati*, sect. *Fumigati*, ser. *Fumigati*. Reproduction: Heterothallic. ITS barcode: EF669969 (alternative markers: *BenA* = EF669825; *CaM* = EF669895; *RPB2* = EF669756).

***Aspergillus leporis*** States & M. Chr., Mycologia 58: 738. 1966. [MB326641]. — Type: NY RMF 99. Ex-type: CBS 151.66 = NRRL 3216 = ATCC 16490 = NRRL A-14256 = NRRL A-15810 = QM 8995 = RMF99 = WB 5188. Infragen. class: subgen. *Circumdati*, sect. *Flavi*, ser. *Leporum*. Reproduction: asexual. ITS barcode: AF104443 (alternative markers: *BenA* = EF661499; *CaM* = EF661541; *RPB2* = EF661459).

***Aspergillus leucocarpus*** Hadlok & Stolk, Antonie van Leeuwenhoek 35: 9. 1969. [MB326642]. — Type: CBS 353.68. Ex-type: CBS 353.68 = NRRL 3497 = QM 9365 = QM 9707. Infragen. class: subgen. *Aspergillus*, sect. *Aspergillus*, ser. *Leucocarpi*. Reproduction: homothallic. ITS barcode: EF652087 (alternative markers: *BenA* = EF651925; *CaM* = EF652023; *RPB2* = EF651972).

***Aspergillus levisporus*** Hubka *et al.*, Stud. Mycol. 88: 99. 2017. [MB818735]. — Type: CBS H-22820. Ex-type: CBS 141767 = DTO 355-G4 = EMSL No.3211 = CCF 5378 = IBT 34512. Infragen. class: subgen. *Aspergillus*, sect. *Aspergillus*, ser. *Aspergillus*. Reproduction: homothallic. ITS barcode: LT670950 (alternative markers: *BenA* = LT671094; *CaM* = LT671095; *RPB2* = LT671096).

***Aspergillus longistipitatus*** F. Sklenář *et al.*, Mycologia 112: 363. 2020. [MB832714]. — Type: PRM 951573. Ex-type: CCF 5788 = EMSL No. 2705 = NRRL 66886. Infragen. class: subgen. *Nidulantes*, sect. *Nidulantes*, ser. *Unguium*. Reproduction: asexual. ITS barcode: MK713534 (alternative markers: *BenA* = MK695641; *CaM* = MK695652; *RPB2* = MK695663).

***Aspergillus longivesica*** L.H. Huang & Raper, Mycologia 63: 53. 1971. [MB309229]. — Type: WIS Nl l79. Ex-type: CBS 530.71 = NRRL 5215 = ATCC 22434 = IMI 156966 = JCM 10186 = QM 9698. Infragen. class: subgen. *Fumigati*, sect. *Clavati*, ser. *Clavati*. Reproduction: asexual. ITS barcode: EF669991 (alternative markers: *BenA* = EF669847; *CaM* = EF669917; *RPB2* = EF669778).

***Aspergillus loretoensis*** S. González-Martinez & A. Portillo-López, Extremophiles 23: 562. 2019. [MB830181]. — Type: CM-CNRG 624. Ex-type: BCMEX-UABC 6006. Infragen. class: subgen. *Polypaecilum*, sect. *Polypaecilum*, ser. *Salinarum*. Reproduction: asexual. ITS barcode: KX236325 (alternative markers: *BenA* = MK309399; *CaM* = MK309400; *RPB2* = MK312162).

***Aspergillus luchuensis*** Inui, J. Coll. Agric. Imp. Univ. Tokyo 13: 469. 1901. [MB151291]. — Type: Inui 1901, J. Coll. Agric. Imp. Univ. Tokyo 13: Tafel XXII, Figs 1–8 (– lectotype designated here, MBT392280; CBS H-24280 [dried culture] – epitype designated here, MBT392281). Ex-epitype: CBS 205.80 = NBRC 4281 = KACC 46772 = IFM 47726 = RIB 2642. Infragen. class: subgen. *Circumdati*, sect. *Nigri*, ser. *Nigri*. Reproduction: protoheterothallic; both MAT idiomorphs detected (Mageswari *et al.* 2016, Yamada *et al.* 2016, de Vries *et al.* 2017). ITS barcode: JX500081 (alternative markers: *BenA* = JX500062; *CaM* = JX500071; *RPB2* = MN969081).

***Aspergillus lucknowensis*** J.N. Rai *et al.*, Canad. J. Bot. 46: 1483. 1968. [MB326643]. — Type: CBS 449.75. Ex-type: CBS 449.75 = NRRL 3491 = ATCC 18607 = IMI 278379 = PIL623 = QM 9271 = WB 5377. Infragen. class: subgen. *Nidulantes*, sect. *Usti*, ser. *Deflecti*. Reproduction: asexual. ITS barcode: EF652459 (alternative markers: *BenA* = EF652283; *CaM* = EF652371; *RPB2* = EF652195).

***Aspergillus luppiae*** [as “*luppii*”] Hubka *et al.*, Mycologia 107: 187. 2015. [MB825372]. — Type: PRM 923447. Ex-type: NRRL 6326 = CBS 653.74 = CCF 4545. Infragen. class: subgen. *Circumdati*, sect. *Flavipedes*, ser. *Spelaei*. Reproduction: asexual. ITS barcode: EF669617 (alternative markers: *BenA* = EU014079; *CaM* = EF669575; *RPB2* = EF669659).

***Aspergillus luteovirescens*** Blochwitz, Ann. Mycol. 31: 80. 1933. [MB269992]. — Type: CBS H-23401. Ex-type: CBS 620.95 = DTO 010-H1 = CBS 116.32 (dead) = IMI 348034 = NRRL 4858 = WB 4858. Infragen. class: subgen. *Circumdati*, sect. *Flavi*, ser. *Nomiarum*. Reproduction: protoheterothallic; MAT 1-2-1 detected (Ramirez-Prado *et al.* 2008). ITS barcode: MG662406 (alternative markers: *BenA* = MG517625; *CaM* = MG517998; *RPB2* = MG517808).

***Aspergillus magaliesburgensis*** Visagie, Stud. Mycol., this issue. 2020. [MB834204]. — Type: PREM 62314. Ex-type: PPRI 6165 = CMV 007A3. Infragen. class: subgen. *Circumdati*, sect. *Flavi*, ser. *Alliacei*. Reproduction: asexual. ITS barcode: MK450649 (alternative markers: *BenA* = MK451116; *CaM* = MK451511; *RPB2* = MK450802).

***Aspergillus magnivesiculatus*** F. Sklenář *et al.*, Stud. Mycol. 88: 211. 2017. [MB818939]. — Type: PRM 944444. Ex-type: NRRL 25866 = CCF 5488 = IBT 34816. Infragen. class: subgen. *Aspergillus*, sect. *Restricti*, ser. *Penicillioides*. Reproduction: asexual. ITS barcode: KY087768 (alternative markers: *BenA* = KY117831; *CaM* = KY068318; *RPB2* = KY118009).

***Aspergillus mallochii*** Visagie *et al.*, MycoKeys 19: 16. 2017. [MB819025]. — Type: DAOM 740296. Ex-type: DAOMC 146054 = CBS 141928 = DTO 357-A5 = KAS 7618. Infragen. class: subgen. *Aspergillus*, sect. *Aspergillus*, ser. *Rubri*. Reproduction: homothallic. ITS barcode: KX450907 (alternative markers: *BenA* = KX450889; *CaM* = KX450902; *RPB2* = KX450894).

***Aspergillus maritimus*** Samson & W. Gams, Adv. Pen. Asp. Syst.: 43. 1986 [1985]. [MB114709]. — Type: CBS 186.77. Ex-type: CBS 186.77. Infragen. class: subgen.: unknown, sect.: unknown, ser.: unknown. Reproduction: homothallic. ITS barcode: n.a. (alternative markers: *BenA* = n.a.; *CaM* = n.a.; *RPB2* = n.a.).

***Aspergillus marvanovae*** Hubka *et al.*, Int. J. Syst. Evol. Microbiol. 63: 787. 2013. [MB801064]. — Type: PRM 860539. Ex-type: NRRL 62486 = IBT 31279 = CCM 8003 = CCF 4037 = IFM 60873 = DTO 303-A2. Infragen. class: subgen. *Fumigati*, sect. *Fumigati*, ser. *Unilaterales*. Reproduction: protoheterothallic; MAT 1-1-1 detected (Hubka *et al.* 2017). ITS barcode: HE974450 (alternative markers: *BenA* = HE974387; *CaM* = HE974389; *RPB2* = HE974396).

***Aspergillus megasporus*** Visagie *et al.*, MycoKeys 19: 17. 2017. [MB819028]. — Type: DAOM 741781. Ex-type: DAOMC 250799 = CBS 141929= DTO 356-H7 = KAS 6176. Infragen. class: subgen. *Aspergillus*, sect. *Aspergillus*, ser. *Aspergillus*. Reproduction: homothallic. ITS barcode: KX450910 (alternative markers: *BenA* = KX450892; *CaM* = KX450905; *RPB2* = KX450897).

***Aspergillus melleus*** Yukawa, J. Coll. Agric. Imp. Univ. Tokyo 1: 358. 1911. [MB164593]. — Type: CBS 546.65. Ex-type: CBS 546.65 = NRRL 5103 = IBT 13510 = IBT 13511 = IBT 13875 = ATCC 16889 = WB 5103. Infragen. class: subgen. *Circumdati*, sect. *Circumdati*, ser. *Circumdati*. Reproduction: asexual. ITS barcode: EF661425 (alternative markers: *BenA* = EF661326; *CaM* = EF661391; *RPB2* = EF661309).

***Aspergillus microcysticus*** Sappa, Allionia 2: 251. 1955. [MB292848]. — Type: IMI 139275. Ex-type: CBS 120.58 = NRRL 4749 = ATCC 16826 = IMI 139275 = QM 8158 = WB 4749. Infragen. class: subgen. *Circumdati*, sect. *Terrei*, ser. *Ambigui*. Reproduction: asexual. ITS barcode: EF669607 (alternative markers: *BenA* = EF669515; *CaM* = EF669565; *RPB2* = EF669649).

***Aspergillus micronesiensis*** Visagie *et al.*, Stud. Mycol. 78: 105. 2014. [MB809192]. — Type: CBS H-21809. Ex-type: CBS 138183 = DTO 267-D5. Infragen. class: subgen. *Circumdati*, sect. *Flavipedes*, ser. *Flavipedes*. Reproduction: asexual. ITS barcode: KJ775548 (alternative markers: *BenA* = KJ775085; *CaM* = KP987067; *RPB2* = KP987023).

***Aspergillus microperforatus*** J.P.Z. Siqueira *et al.*, Med. Mycol. 56: 545. 2018. [MB820080]. — Type: CBS H-22998. Ex-type: UTHSCSA DI16-407 = CBS 142376 = FMR 14071. Infragen. class: subgen. *Aspergillus*, sect. *Aspergillus*, ser. *Rubri*. Reproduction: homothallic. ITS barcode: LT627271 (alternative markers: *BenA* = LT627296; *CaM* = LT627321; *RPB2* = LT627346).

***Aspergillus minisclerotigenes*** Vaamonde *et al.*, Int. J. Syst. Evol. Microbiol. 58: 733. 2008. [MB505188]. — Type: Pildain *et al.* 2008, Int. J. Syst. Evol. Microbiol. 58: p. 732, Fig. 3 (– lectotype designated here, MBT392282; CBS H-24281 [dried culture] – epitype designated here, MBT392283). Ex-epitype: CBS 117635 = DTO 009-F7 = DTO 303-C6 = IBT 25032. Infragen. class: subgen. *Circumdati*, sect. *Flavi*, ser. *Flavi*. Reproduction: protoheterothallic; both MAT idiomorphs detected (Soares *et al.* 2012). ITS barcode: EF409239 (alternative markers: *BenA* = EF203148; *CaM* = MG518009; *RPB2* = MG517799).

***Aspergillus miraensis*** (L.C. Zhang *et al.*) Hubka *et al.*, Plant Syst. Evol. 302: 1288. 2016. [MB816283]. Basionym: *Emericella miraensis* L.C. Zhang *et al.*, Mycotaxon 125: 132. 2013. [MB800444]. — Type: CGMCC 3.14984. Ex-type: CGMCC 3.14984 = DTO 323-B2. Infragen. class: subgen. *Nidulantes*, sect. *Nidulantes*, ser. *Stellati*. Reproduction: homothallic. ITS barcode: KU866642 (alternative markers: *BenA* = KC342577; *CaM* = KU866780; *RPB2* = KU867045).

***Aspergillus monodii*** (Locq.-Lin.) Varga *et al.*, Stud. Mycol. 69: 91. 2011. [MB560402]. Basionym: *Fennellia monodii* Locq.-Lin., Mycotaxon 39: 10. 1990. [MB126894]. — Type: LCP 89-3570 (PC). Ex-type: DTO 69-A3 = CBS 435.93 = DTO 026-I4. Infragen. class: subgen. *Nidulantes*, sect. *Usti*, ser. *Monodiorum*. Reproduction: homothallic; asexual morph unknown. ITS barcode: FJ531150 (alternative markers: *BenA* = FJ531171; *CaM* = FJ531142; *RPB2* = MN969082).

***Aspergillus montevidensis*** Talice & Mackinnon, Compt. Rend. Soc. Biol. Fr. 108: 1007. 1931. [MB309231]. — Type: NRRL 108. Ex-type: CBS 491.65 = NRRL 108 = BPI 884202 = ATCC 10077 = IHEM 3337 = IMI 172290 = NRRL 109 = QM 7423 = Thom 5290 = Thom 5633.24 = WB 108. Infragen. class: subgen. *Aspergillus*, sect. *Aspergillus*, ser. *Chevalierorum*. Reproduction: homothallic. ITS barcode: EF652077 (alternative markers: *BenA* = EF651898; *CaM* = EF652020; *RPB2* = EF651964).

***Aspergillus mottae*** C. Soares *et al.*, Mycologia 104: 692. 2012. [MB561841]. — Type: MUM-H 10.231. Ex-type: CBS 130016 = DTO 223-C8. Infragen. class: subgen. *Circumdati*, sect. *Flavi*, ser. *Flavi*. Reproduction: protoheterothallic; both MAT idiomorphs detected (Soares *et al.* 2013). ITS barcode: JF412767 (alternative markers: *BenA* = HM803086; *CaM* = MG518058; *RPB2* = MG517878).

***Aspergillus movilensis*** A. Nováková *et al.*, Mycologia 107: 190. 2015. [MB808144]. — Type: PRM 923448. Ex-type: PRM 923449 = CCF 4410 = CMF ISB 2614 = NRRL 62819 = CBS 134395 = DTO 316-C6. Infragen. class: subgen. *Circumdati*, sect. *Flavipedes*, ser. *Spelaei*. Reproduction: asexual. ITS barcode: KP987089 (alternative markers: *BenA* = HG916697; *CaM* = HG916740; *RPB2* = HG916718).

***Aspergillus multicolor*** Sappa, Allionia 2: 87. 1954. [MB292849]. — Type: IMI 69875. Ex-type: CBS 133.54 = NRRL 4775 = ATCC 16804 = IFO 8133 = IMI 69857 = LSHBBB 356 = QM 1952 = WB 4281 = WB 4775. Infragen. class: subgen. *Nidulantes*, sect. *Nidulantes*, ser. *Multicolores*. Reproduction: asexual. ITS barcode: EF652477 (alternative markers: *BenA* = EF652301; *CaM* = EF652389; *RPB2* = EF652213).

***Aspergillus multiplicatus*** Yaguchi *et al.*, Mycoscience 35: 310. 1994. [MB412530]. — Type: CBM PF-1154. Ex-type: CBS 646.95 = IBT 17517 = DTO 050-E2. Infragen. class: subgen. *Fumigati*, sect. *Fumigati*, ser. *Unilaterales*. Reproduction: homothallic. ITS barcode: HE974445 (alternative markers: *BenA* = DQ114129; *CaM* = DQ114137; *RPB2* = HE974397).

***Aspergillus mulundensis*** Bills & Frisvad, J Antibiot. 69: 143. 2016. [MB813062]. — Type: DSMZ 5745. Ex-type: DTO 316-C9 = DSMZ 5745a = IBT 33104. Infragen. class: subgen. *Nidulantes*, sect. *Nidulantes*, ser. *Multicolores*. Reproduction: asexual. ITS barcode: KP985732 (alternative markers: *BenA* = KP985735; *CaM* = KP985734; *RPB2* = KU866989).

***Aspergillus muricatus*** Udagawa *et al.*, Mycotaxon 52: 210. 1994. [MB362530]. — Type: CBM BF-42515. Ex-type: CBS 112808 = NRRL 35674 = IBT 19374 = IMI 36852. Infragen. class: subgen. *Circumdati*, sect. *Circumdati*, ser. *Circumdati*. Reproduction: homothallic. ITS barcode: EF661434 (alternative markers: *BenA* = EF661356; *CaM* = EF661377; *RPB2* = EF661314).

***Aspergillus navahoensis*** M. Chr. & States, Mycologia 74: 226. 1982. [MB110496]. — Type: NY SD-5. Ex-type: CBS 351.81 = NRRL 13002 = ATCC 44663 = IMI 259971. Infragen. class: subgen. *Nidulantes*, sect. *Nidulantes*, ser. *Nidulantes*. Reproduction: homothallic. ITS barcode: EF652424 (alternative markers: *BenA* = EF652248; *CaM* = EF652336; *RPB2* = EF652160).

***Aspergillus neoafricanus*** Samson *et al.*, Stud. Mycol. 69: 53. 2011. [MB560391]. Replaced synonym: *Aspergillus terreus* var. *africanus* Fennell & Raper, Mycologia 47: 86. 1955. [MB351904]. — Type: Fennell & Raper 1955, Mycologia 47: p. 87, Fig. 8 (– lectotype designated here, MBT392284; CBS H-24282 [dried culture] – epitype designated here, MBT392285). Ex-epitype: CBS 130.55 = NRRL 2399 = ATCC 16792 = IHEM 4380 = IMI 61457 = MUCL 31316 = NRRL A-3175 = QM 1913 = VKMF-2037 = WB 2399. Infragen. class: subgen. *Circumdati*, sect. *Terrei*, ser. *Terrei*. Reproduction: asexual. ITS barcode: EF669585 (alternative markers: *BenA* = EF669516; *CaM* = EF669543; *RPB2* = EF669627).

***Aspergillus neoalliaceus*** A. Nováková *et al.*, Stud. Mycol. 93: 43. 2019. [MB823775]. — Type: CBS H-23363. Ex-type: CBS 143681 = DTO 326-D3 = S765 = CCF 5433 = IBT 33110 = IBT 33353. Infragen. class: subgen. *Circumdati*, sect. *Flavi*, ser. *Alliacei*. Reproduction: asexual. ITS barcode: MH279420 (alternative markers: *BenA* = MG517763; *CaM* = MG518133; *RPB2* = MG517954).

***Aspergillus neobridgeri*** Frisvad & Samson, Stud. Mycol. 50: 35. 2004. [MB500004]. — Type: CBS 559.82. Ex-type: CBS 559.82 = NRRL 13078 = IBT 14026. Infragen. class: subgen. *Circumdati*, sect. *Circumdati*, ser. *Sclerotiorum*. Reproduction: asexual. ITS barcode: EF661410 (alternative markers: *BenA* = EF661345; *CaM* = EF661359; *RPB2* = EF661298).

***Aspergillus neocarnoyi*** Kozak., Mycol. Pap. 161: 63. 1989. [MB127756]. Basionym: *Eurotium carnoyi* Malloch & Cain, Canad. J. Bot. 50: 63. 1972. [MB297362]. — Type: IMI 172279. Ex-type: CBS 471.65 = NRRL 126 = ATCC 16924 = IMI 172279 = LSHTM A32 = QM 7402 = Thom 5612.A32 = WB 126. Infragen. class: subgen. *Aspergillus*, sect. *Aspergillus*, ser. *Aspergillus*. Reproduction: homothallic. ITS barcode: EF652057 (alternative markers: *BenA* = EF651903; *CaM* = EF651985; *RPB2* = EF651942).

***Aspergillus neoflavipes*** Hubka *et al.*, Mycologia 107: 192. 2015. [MB808147]. Replaced synonym: *Fennellia flavipes* B.J. Wiley & E.G. Simmons, Mycologia 65: 937. 1973. [MB314109]. — Type: BPI 410858. Ex-type: CBS 260.73 = NNRL 5504 = ATCC 24484 = IMI 171883 = IFM 40894 = CCF 4552. Infragen. class: subgen. *Circumdati*, sect. *Flavipedes*, ser. *Flavipedes*. Reproduction: homothallic. ITS barcode: EF669614 (alternative markers: *BenA* = EU014084; *CaM* = EF669572; *RPB2* = EF669656).

***Aspergillus neoglaber*** Kozak., Mycol. Pap. 161: 56. 1989. [MB127762]. Replaced synonym: *Aspergillus fischeri* var. *glaber* Fennell & Raper, Mycologia 47: 74. 1955. [MB351897]. — Type: IMI 61447. Ex-type: CBS 111.55 = NRRL 2163 = ATCC 16909 = IFO 8789 = IMI 061447ii = IMI 367412 = IMI 61447 = NRRL A-2175 = QM 1903 = WB 2163 = DTO 050-D4 = DTO 164-H7. Infragen. class: subgen. *Fumigati*, sect. *Fumigati*, ser. *Neoglabri*. Reproduction: homothallic. ITS barcode: EF669948 (alternative markers: *BenA* = EU014107; *CaM* = EU014120; *RPB2* = EF669736).

***Aspergillus neoindicus*** Samson *et al.*, Stud. Mycol. 69: 53. 2011. [MB560394]. Replaced synonym: *Aspergillus niveus* var. *indicus* [as “*indica*”] Lal & A.K. Sarbhoy, Indian Phytopathol. 25: 311. 1972 [1973]. [MB252165]. — Type: ITCC 1575 (holotype); CBS H-24283 (isotype). Ex-type: CBS 444.75 = NRRL 6134 = IMI 334935. Infragen. class: subgen. *Circumdati*, sect. *Terrei*, ser. *Nivei*. Reproduction: asexual. ITS barcode: EF669616 (alternative markers: *BenA* = EF669532; *CaM* = EF669574; *RPB2* = EF669658).

***Aspergillus neoniger*** Varga *et al.*, Stud. Mycol. 69: 16. 2011. [MB560390]. — Type: CBS H-20630. Ex-type: CBS 115656 = NRRL 62634. Infragen. class: subgen. *Circumdati*, sect. *Nigri*, ser. *Nigri*. Reproduction: protoheterothallic; unpublished (genome data; Vesth *et al.* 2018). ITS barcode: FJ491682 (alternative markers: *BenA* = FJ491691; *CaM* = FJ491700; *RPB2* = KC796429).

***Aspergillus neoniveus*** Samson *et al.*, Stud. Mycol. 69: 53. 2011. [MB560395]. Replaced synonym: *Emericella nivea* B.J. Wiley & E.G. Simmons, Mycologia 65: 934. 1973. [MB313507]. — Type: QM 8942. Ex-type: CBS 261.73 = NRRL 5299 = ATCC 24482 = IMI 171878. Infragen. class: subgen. *Circumdati*, sect. *Flavipedes*, ser. *Neonivei*. Reproduction: homothallic. ITS barcode: EF669612 (alternative markers: *BenA* = EU014098; *CaM* = EF669570; *RPB2* = KP987024).

***Aspergillus nidulans*** (Eidam) G. Winter, Rabenh. Krypt.-Fl., ed. 2, 1: 62. 1884. [MB182069]. Basionym: *Sterigmatocystis nidulans* Eidam, Beitr. Biol. Pflanzen 3: 393. 1883. [MB221350]. — Type: IMI 86806. Ex-type: CBS 589.65 = NRRL 187 = ATCC 10074 = IHEM 3563 = IMI 126691 = IMI 86806 = QM 1985 = Thom 4640.5 = WB 187. Infragen. class: subgen. *Nidulantes*, sect. *Nidulantes*, ser. *Nidulantes*. Reproduction: homothallic. ITS barcode: EF652427 (alternative markers: *BenA* = EF652251; *CaM* = EF652339; *RPB2* = EF652163).

***Aspergillus niger*** Tiegh., Ann. Sci. Nat., Bot., ser. 5, 8: 240. 1867.; *nom. cons.* (Kozakiewicz *et al.* 1992). [MB284309]. — Type: CBS 554.65. Ex-type: CBS 554.65 = NRRL 326 = ATCC 16888 = IFO 33023 = IHEM 3415 = IMI 050566ii = IMI 50566 = JCM 10254 = QM 9270 = QM 9946 = Thom 2766 = WB 326. Infragen. class: subgen. *Circumdati*, sect. *Nigri*, ser. *Nigri*. Reproduction: protoheterothallic; both MAT idiomorphs detected (Pel et al., 2007, Mageswari et al, 2016). ITS barcode: EF661186 (alternative markers: *BenA* = EF661089; *CaM* = EF661154; *RPB2* = EF661058).

***Aspergillus nishimurae*** Takada *et al.*, Mycoscience 42: 362. 2001. [MB474712]. — Type: CBM-FA-919 (holotype); PRM 935217 (epitype). Ex-epitype: IFM 54133 = CBM-FA-0910 = CCF 4547 = IBT 29024. Infragen. class: subgen. *Fumigati*, sect. *Fumigati*, ser. *Unilaterales*. Reproduction: Heterothallic. ITS barcode: HE974449 (alternative markers: *BenA* = AB201360; *CaM* = HE974392; *RPB2* = HE974393).

***Aspergillus niveoglaucus*** Thom & Raper, U.S.D.A. Misc. Pub. 426: 35. 1941. [MB120985]. — Type: IMI 32050ii. Ex-type: CBS 114.27 = CBS 517.65 = NRRL 127 = ATCC 10075 = IMI 32050 = LSHBA 16 = NRRL 129 = NRRL 130 = QM 1977 = Thom 5612.A16 = Thom 5633 = Thom 5633.7 = Thom 7053.2 = WB 127 = WB 130. Infragen. class: subgen. *Aspergillus*, sect. *Aspergillus*, ser. *Aspergillus*. Reproduction: homothallic. ITS barcode: EF652058 (alternative markers: *BenA* = EF651905; *CaM* = EF651993; *RPB2* = EF651943).

***Aspergillus niveus*** Blochwitz, Ann. Mycol. 27: 205. 1929. [MB272402]. — Type: IMI 171878. Ex-type: CBS 115.27 = NRRL 5505. Infragen. class: subgen. *Circumdati*, sect. *Terrei*, ser. *Nivei*. Reproduction: asexual; the putative sexual morph of *A*. *niveus*, *Emericella nivea* Wiley & Simmons, represents a different species - *A. neoniveus* (Samson *et al.* 2011a). ITS barcode: EF669615 (alternative markers: *BenA* = EF669528; *CaM* = EF669573; *RPB2* = EF669657).

***Aspergillus nomiae*** [as “*nomius*”] Kurtzman *et al.*, Antonie van Leeuwenhoek 53: 151. 1987. [MB634998]. — Type: NRRL 13137. Ex-type: CBS 260.88 = NRRL 13137 = ATCC 15546 = FRR 3339 = IMI 331920 = LCP 89.3558 = NRRL 6108 = NRRL A-13671 = NRRL A-13794. Infragen. class: subgen. *Circumdati*, sect. *Flavi*, ser. *Nomiarum*. Reproduction: Heterothallic (Horn *et al.* 2011). ITS barcode: AF027860 (alternative markers: *BenA* = AF255067; *CaM* = AY017588; *RPB2* = EF661456).

***Aspergillus noonimiae*** Tanney *et al.*, Stud. Mycol. 88: 252. 2017. [MB822733]. — Type: DAOM 745797. Ex-type: DAOMC 251754 = UAMH 11836 = CBS 143382 = KAS 8125 = SLOAN 7955 = PN06TH-370. Infragen. class: subgen. *Polypaecilum*, sect. *Polypaecilum*, ser. *Noonimiarum*. Reproduction: asexual. ITS barcode: KY980641 (alternative markers: *BenA* = KY980569; *CaM* = KY980605; *RPB2* = KY980466).

***Aspergillus novofumigatus*** S.B. Hong *et al.*, Mycologia 97: 1368. 2006. [MB500297]. — Type: CBS 117520. Ex-type: CBS 117520 = IBT 16806 = KACC 41934 = IFM 55215 = CCF 4695 = DTO 022-B7 = DTO 003-H3. Infragen. class: subgen. *Fumigati*, sect. *Fumigati*, ser. *Fumigati*. Reproduction: protoheterothallic; both MAT idiomorphs detected (Dudová 2014). ITS barcode: MN431372 (alternative markers: *BenA* = DQ094886; *CaM* = DQ094893; *RPB2* = MN969083).

***Aspergillus novoguineensis*** A.J. Chen *et al.*, Stud. Mycol. 85: 75. 2016. [MB817725]. — Type: CBS H-22729. Ex-type: CBS 906.96 = DTO 021-G5 = IBT 29312. Infragen. class: subgen. *Fumigati*, sect. *Cervini*, ser. *Cervini*. Reproduction: asexual. ITS barcode: FJ491622 (alternative markers: *BenA* = FJ491641; *CaM* = FJ491605; *RPB2* = KX423681).

***Aspergillus novoparasiticus*** S.S. Gonçalves *et al.*, Med. Mycol. 50: 158. 2011. [MB516612]. — Type: CBS H-20401. Ex-type: CBS 126849 = DTO 223-C3 = LEMI 250 = FMR 10121. Infragen. class: subgen. *Circumdati*, sect. *Flavi*, ser. *Flavi*. Reproduction: protoheterothallic; MAT1-1-1 detected (Carvajal-Campos *et al.* 2017). ITS barcode: MG662397 (alternative markers: *BenA* = MG517684; *CaM* = MG518055; *RPB2* = MG517875).

***Aspergillus nutans*** McLennan & Ducker, Austral. J. Bot. 2: 355. 1954. [MB292850]. — Type: IMI 62874ii. Ex-type: CBS 121.56 = NRRL 575 = NRRL 4364 = NRRL A-6280 = ATCC 16914 = IFO 8134 = IMI 062874ii = IMI 62874 = QM 8159 = WB 4364 = WB 4546 = WB 4776. Infragen. class: subgen. *Fumigati*, sect. *Cervini*, ser. *Cervini*. Reproduction: asexual. ITS barcode: EF661272 (alternative markers: *BenA* = EF661249; *CaM* = EF661262; *RPB2* = EF661227).

***Aspergillus occultus*** Visagie *et al.*, Stud. Mycol. 78: 32. 2014. [MB809198]. — Type: CBS H-21794. Ex-type: CBS 137330 = IBT 32285 = DTO 231-A7. Infragen. class: subgen. *Circumdati*, sect. *Circumdati*, ser. *Steyniorum*. Reproduction: asexual. ITS barcode: KJ775443 (alternative markers: *BenA* = KJ775061; *CaM* = KJ775239; *RPB2* = MN969084).

***Aspergillus ochraceopetaliformis*** Bat. & Maia, Anais Soc. Biol. Pernambuco 15: 213. 1957. [MB292851]. — Type: no 270 Instituto de Micologia, Iniversidade do Recife. Ex-type: CBS 123.55 = NRRL 4752 = IBT 14347 = ATCC 12066 = IMI 211804 = QM 6955 = WB 4752. Infragen. class: subgen. *Circumdati*, sect. *Circumdati*, ser. *Steyniorum*. Reproduction: asexual. ITS barcode: EF661429 (alternative markers: *BenA* = EF661350; *CaM* = EF661388; *RPB2* = EF661283).

***Aspergillus ochraceoroseus*** Bartoli & Maggi, Trans. Brit. Mycol. Soc. 71: 393. 1979 [1978]. [MB309233]. — Type: RO 104 S. Ex-type: CBS 550.77 = NRRL 28622 = ATCC 38873 = SRRC1432. Infragen. class: subgen. *Nidulantes*, sect. *Ochraceorosei*, ser. *Ochraceorosei*. Reproduction: asexual. ITS barcode: EF661224 (alternative markers: *BenA* = EF661113; *CaM* = EF661137; *RPB2* = EF661074).

***Aspergillus ochraceus*** K. Wilh., Beitr. Kenntn. Aspergillus: 66. 1877. [MB190223]. — Type: IMI 16247iv. Ex-type: CBS 108.08 = NRRL 398 = IBT 11952 = ATCC 1008 = CECT2093 = DSM 824 = HARVARD296 = IMI 16247 = NCTC 3889 = NRRL 1642 = QM 6731 = Thom 112 = WB 398. Infragen. class: subgen. *Circumdati*, sect. *Circumdati*, ser. *Circumdati*. Reproduction: asexual. ITS barcode: EF661419 (alternative markers: *BenA* = EF661322; *CaM* = EF661381; *RPB2* = EF661302).

***Aspergillus oerlinghausenensis*** Bader & Houbraken, FEMS Microbiol. Letter 363 (3/fnv236): 4. 2016. [MB813868]. — Type: CBS H-22119. Ex-type: CBS 139183 = IBT 33878 = DTO 316-A3. Infragen. class: subgen. *Fumigati*, sect. *Fumigati*, ser. *Fumigati*. Reproduction: protoheterothallic; MAT 1-1-1 detected (Houbraken *et al.* 2016). ITS barcode: KT359601 (alternative markers: *BenA* = KT359603; *CaM* = KT359605; *RPB2* = MN969162).

***Aspergillus olivicola*** Frisvad *et al.*, Mycologia 100: 781. 2008. [MB507362]. — Type: CBS H-19888. Ex-type: CBS 119.37 = IBT 21903 = DTO 002-I2. Infragen. class: subgen. *Nidulantes*, sect. *Nidulantes*, ser. *Stellati*. Reproduction: homothallic. ITS barcode: EU448268 (alternative markers: *BenA* = AY339996; *CaM* = EU443986; *RPB2* = KU866923).

***Aspergillus olivimuriae*** S.W. Peterson & S. Crognale, Int. J. Syst. Evol. Microbiol. 69: 2901. 2019. [MB826866]. — Type: BPI 910647. Ex-type: NRRL 66783 = DIBAF 6C2. Infragen. class: subgen. *Circumdati*, sect. *Flavipedes*, ser. *Olivimuriarum*. Reproduction: asexual. ITS barcode: MH298877 (alternative markers: *BenA* = MH492010; *CaM* = MH492011; *RPB2* = MH492012).

***Aspergillus omanensis*** Y. Horie & Udagawa, Mycoscience 36: 391. 1995. [MB414655]. — Type: CBM FA-700. Ex-type: CBM FA-700 = IFM 54275. Infragen. class: subgen. *Nidulantes*, sect. *Nidulantes*, ser. *Nidulantes*. Reproduction: homothallic. ITS barcode: n.a. (alternative markers: *BenA* = AB248347; *CaM* = AB524047; *RPB2* = n.a.).

***Aspergillus oryzae*** (Ahlb.) Cohn, Jahresber. Schles. Ges. Vaterl. Cult. 61: 226. 1884. [MB184394]. Basionym: *Eurotium oryzae* Ahlb., Dingler's Polytechn. J. 230: 330. 1878. [MB225012]. — Type: IMI 16266. Ex-type: CBS 100925 = CBS 102.07 = NRRL 447 = ATCC 1011 = ATCC 12891 = ATCC 4814 = ATCC 7561 = ATCC 9102 = IAM13118 = IFO 4075 = IFO 537 = IFO 5375 = IMI 16266 = IMI 44242 = LSHBA c .19 = NCTC 598 = NRRL 692 = QM 6735 = Thom 113 = WB 447. Infragen. class: subgen. *Circumdati*, sect. *Flavi*, ser. *Flavi*. Reproduction: protoheterothallic; both MAT idiomorphs detected (Wada *et al.* 2012). ITS barcode: EF661560 (alternative markers: *BenA* = EF661483; *CaM* = EF661506; *RPB2* = EF661438).

***Aspergillus osmophilus*** Asgari & Zare, Mycoscience 55: 58. 2014. [MB803278]. — Type: IRAN 16110. Ex-type: IRAN 2090C = CBS 134258. Infragen. class: subgen. *Aspergillus*, sect. *Aspergillus*, ser. *Xerophili*. Reproduction: homothallic. ITS barcode: KC473921 (alternative markers: *BenA* = LT671127; *CaM* = LT671128; *RPB2* = LT671129).

***Aspergillus ostianus*** Wehmer, Bot. Centralbl. 80: 461. 1899. [MB179393]. — Type: IMI 15960. Ex-type: CBS 103.07 = CBS 548.65 = IBT 13386 = NRRL 420 = ATCC 16887 = IMI 015960iii = IMI 15960 = LCP 89.2584 = LSHBA c .35 = NCTC 3788 = QM 7460 = Thom 4724.35 = WB 420. Infragen. class: subgen. *Circumdati*, sect. *Circumdati*, ser. *Circumdati*. Reproduction: asexual. ITS barcode: EF661421 (alternative markers: *BenA* = EF661324; *CaM* = EF661385; *RPB2* = EF661304).

***Aspergillus pachycaulis*** F. Sklenář *et al.*, Stud. Mycol. 88: 211. 2017. [MB823048]. — Type: PRM 944432. Ex-type: NRRL 25824 = CCF 5492 = DTO 356-D2 = IBT 34521 = IBT 34812. Infragen. class: subgen. *Aspergillus*, sect. *Restricti*, ser. *Restricti*. Reproduction: asexual. ITS barcode: KY087758 (alternative markers: *BenA* = KY117821; *CaM* = KY068308; *RPB2* = KY117999).

***Aspergillus pachycristatus*** Matsuzawa *et al.*, Mycoscience 53: 439. 2012. [MB580944]. — Type: IFM 55265. Ex-type: IFM 55265 = NBRC 104790. Infragen. class: subgen. *Nidulantes*, sect. *Nidulantes*, ser. *Nidulantes*. Reproduction: homothallic. ITS barcode: n.a. (alternative markers: *BenA* = AB375875; *CaM* = AB524062; *RPB2* = n.a.).

***Aspergillus pallidofulvus*** Visagie *et al.*, Stud. Mycol. 78: 40. 2014. [MB809199]. — Type: CBS H-21796. Ex-type: CBS 640.78 = NRRL 4789 = IBT 13871 = IFO 4095 = WB 4789. Infragen. class: subgen. *Circumdati*, sect. *Circumdati*, ser. *Circumdati*. Reproduction: asexual. ITS barcode: EF661423 (alternative markers: *BenA* = EF661328; *CaM* = EF661389; *RPB2* = MN969085).

***Aspergillus panamensis*** Raper & Thom, Mycologia 36: 568. 1944. [MB284311]. — Type: IMI 19393iii. Ex-type: CBS 120.45 = NRRL 1785 = ATCC 16797 = IMI 019393ii = IMI 019393iii = IMI 19393 = LSHBA .61 = NCTC 6974 = QM 6829 = QM 8897 = WB 1785. Infragen. class: subgen. *Nidulantes*, sect. *Sparsi*, ser. *Conjuncti*. Reproduction: asexual. ITS barcode: EF661177 (alternative markers: *BenA* = EF661109; *CaM* = EF661135; *RPB2* = EF661040).

***Aspergillus papuensis*** (Samson *et al.*) Samson *et al.*, Stud. Mycol. 78: 155. 2014. [MB809593]. Basionym: *Neosartorya papuensis* Samson *et al.*, Stud. Mycol. 59: 190. 2007. [MB505571]. — Type: CBS H-6277. Ex-type: CBS 841.96 = IBT 27801 = DTO 050-D1. Infragen. class: subgen. *Fumigati*, sect. *Fumigati*, ser. *Neoglabri*. Reproduction: homothallic. ITS barcode: EU220280 (alternative markers: *BenA* = AY870738; *CaM* = AY870697; *RPB2* = MN969086).

***Aspergillus parasiticus*** Speare, Bull. Hawaiian Sugar Planters Assoc. Exp. Sta. Pathol. Physiol. Ser. 12: 38. 1912. [MB191085]. — Type: IMI 15957ix. Ex-type: CBS 100926 = CBS 103.13 = NRRL 502 = ATCC 1018 = ATCC 6474 = ATCC 7865 = IMI 15957 = IMI 15957ii = IMI 15957iv = IMI 15957ix = IMI 15957vi = IMI 15957vii = LCP 89.2566 = LSHBA c 14 = NCTC 975 = NRRL 1731 = NRRL 3315 = NRRL A-13360 = NRRL A-14693 = Thom 3509 = WB 502. Infragen. class: subgen. *Circumdati*, sect. *Flavi*, ser. *Flavi*. Reproduction: Heterothallic (Horn *et al.* 2009b). ITS barcode: AY373859 (alternative markers: *BenA* = EF661481; *CaM* = AY017584; *RPB2* = EF661449).

***Aspergillus parvulus*** G. Sm., Trans. Brit. Mycol. Soc. 44: 45. 1961. [MB121074]. — Type: IMI 86558. Ex-type: CBS 136.61 = NRRL 4753 = ATCC 16911 = IMI 86558 = LSHBBB 405 = NRRL 1846 = QM 7955 = UC4613 = WB 4753. Infragen. class: subgen. *Fumigati*, sect. *Cervini*, ser. *Cervini*. Reproduction: asexual. ITS barcode: EF661269 (alternative markers: *BenA* = EF661247; *CaM* = EF661259; *RPB2* = EF661233).

***Aspergillus penicillioides*** Speg., Revista Fac. Agron. Univ. Nac. La Plata 2: 246. 1896. [MB309234]. — Type: IMI 211342. Ex-type: CBS 540.65 = NRRL 4548 = ATCC 16910 = IMI 211342 = IMUR540 = QM 9370 = WB 4548. Infragen. class: subgen. *Aspergillus*, sect. *Restricti*, ser. *Penicillioides*. Reproduction: asexual. ITS barcode: EF652036 (alternative markers: *BenA* = EF651928; *CaM* = EF652024; *RPB2* = EF651930).

***Aspergillus pepii*** Despot *et al.*, Mycol. Prog. 16: 67. 2016 [2017]. [MB817073]. — Type: SZMC 23791. Ex-type: MFBF AV11051B IX = SZMC 22333 = CBS 142028. Infragen. class: subgen. *Nidulantes*, sect. *Nidulantes*, ser. *Versicolores*. Reproduction: asexual. ITS barcode: KU613368 (alternative markers: *BenA* = KU613371; *CaM* = KU613365; *RPB2* = n.a.).

***Aspergillus pernambucoensis*** Y. Horie *et al.*, Mycoscience 55: 86. 2014. [MB801324]. — Type: IFM 61342H. Ex-type: IFM 61342 = JCM 19244 = CBS 137449 = DTO 316-G1. Infragen. class: subgen. *Fumigati*, sect. *Fumigati*, ser. *Unilaterales*. Reproduction: homothallic. ITS barcode: MN431373 (alternative markers: *BenA* = AB743856; *CaM* = AB743862; *RPB2* = MN969087).

***Aspergillus persii*** A.M. Corte & Zotti, Mycotaxon 83: 276 2002. [MB374215]. — Type: MUCL 41970. Ex-type: CBS 112795 = NRRL 35669 = IBT 22660 = MUCL 41970. Infragen. class: subgen. *Circumdati*, sect. *Circumdati*, ser. *Sclerotiorum*. Reproduction: asexual. ITS barcode: FJ491580 (alternative markers: *BenA* = AY819988; *CaM* = FJ491559; *RPB2* = EF661295).

***Aspergillus petersonii*** Jurjević & Hubka, Plant Syst. Evol. 301: 2454. 2015. [MB814440]. — Type: PRM 933841. Ex-type: CCF 4999 = NRRL 66216. Infragen. class: subgen. *Circumdati*, sect. *Petersoniorum*, ser. *Petersoniorum*. Reproduction: asexual. ITS barcode: LN849393 (alternative markers: *BenA* = LN849407; *CaM* = LN849422; *RPB2* = LN849438).

***Aspergillus peyronelii*** Sappa, Allionia 2: 248. 1955. [MB292855]. — Type: Plate 1, subfigures 1–4, in Sappa 1955, Allionia 2: 249 (lectotype); a dried herbarium specimen derived from the culture IMI 139271 (epitype, PRM 933831) (designated in Jurjević *et al.* 2015). Ex-epitype: IMI 139271 = CCF 4942 = NRRL 4754 = ATCC 16840. Infragen. class: subgen. *Circumdati*, sect. *Petersoniorum*, ser. *Petersoniorum*. Reproduction: asexual. ITS barcode: LN849398 (alternative markers: *BenA* = LN849412; *CaM* = LN849428; *RPB2* = LN849443).

***Aspergillus pipericola*** Frisvad *et al.*, Stud. Mycol. 93: 46. 2019. [MB823774]. — Type: CBS H-23362. Ex-type: CBS 143680 = DTO 228-H4 = IBT 24628. Infragen. class: subgen. *Circumdati*, sect. *Flavi*, ser. *Flavi*. Reproduction: asexual. ITS barcode: MG662385 (alternative markers: *BenA* = MG517717; *CaM* = MG518087; *RPB2* = MG517908).

***Aspergillus piperis*** Samson & Frisvad, Stud. Mycol. 50: 57. 2004. [MB500009]. — Type: CBS H-13434. Ex-type: CBS 112811 = IBT 24630 = IBT 26239 = NRRL 62631. Infragen. class: subgen. *Circumdati*, sect. *Nigri*, ser. *Nigri*. Reproduction: protoheterothallic; unpublished (genome data, Vesth *et al.* 2018). ITS barcode: EU821316 (alternative markers: *BenA* = FJ629303; *CaM* = EU163267; *RPB2* = KC796427).

***Aspergillus pisce*** [as “*pisci*”] (A.D. Hocking & Pitt) Houbraken *et al.*, Stud. Mycol. 78: 155. 2014. [MB812441]. Basionym: *Polypaecilum pisce* A.D. Hocking & Pitt, Mycotaxon 22: 200. 1985. [MB536436]. — Type: FRR 2732. Ex-type: FRR 2732 = ATCC 56982 = IMI 288726. Infragen. class: subgen. *Polypaecilum*, sect. *Polypaecilum*, ser. *Polypaecilum*. Reproduction: asexual. ITS barcode: MF362690 (alternative markers: *BenA* = MF362691; *CaM* = MN969231; *RPB2* = JN121415).

***Aspergillus pluriseminatus*** (Stchigel & Guarro) Samson *et al.*, Stud. Mycol. 78: 155. 2014. [MB809595]. Basionym: *Emericella pluriseminata* Stchigel & Guarro, Mycologia 89: 937. 1997. [MB443124]. — Type: FMR 5588. Ex-type: CBS 100523 = FMR 5588 = IMI 370867 = DTO 011-H1. Infragen. class: subgen. *Nidulantes*, sect. *Nidulantes*, ser. *Multicolores*. Reproduction: homothallic; asexual morph unknown. ITS barcode: KU866566 (alternative markers: *BenA* = AY339989; *CaM* = EU443988; *RPB2* = KU866937).

***Aspergillus polyporicola*** Hubka *et al.*, Mycologia 107: 194. 2015. [MB808145]. — Type: PRM 923452. Ex-type: NRRL 32683 = CCF 4553. Infragen. class: subgen. *Circumdati*, sect. *Flavipedes*, ser. *Spelaei*. Reproduction: asexual. ITS barcode: EF669595 (alternative markers: *BenA* = EU014088; *CaM* = EF669553; *RPB2* = EF669637).

***Aspergillus porosus*** A.J. Chen *et al.*, Stud. Mycol. 88: 113. 2017. [MB818736]. — Type: CBS H-22822. Ex-type: CBS 141770 = DTO 262-D7 = IBT 34443. Infragen. class: subgen. *Aspergillus*, sect. *Aspergillus*, ser. *Chevalierorum*. Reproduction: homothallic. ITS barcode: LT670961 (alternative markers: *BenA* = LT671130; *CaM* = LT671131; *RPB2* = LT671132).

***Aspergillus porphyreostipitatus*** Visagie *et al.*, Stud. Mycol. 78: 112. 2014. [MB809196]. — Type: CBS H-21813. Ex-type: CBS 138203 = DTO 266-D9. Infragen. class: subgen. *Nidulantes*, sect. *Usti*, ser. *Usti*. Reproduction: asexual. ITS barcode: KJ775564 (alternative markers: *BenA* = KJ775080; *CaM* = KJ775338; *RPB2* = KU866987).

***Aspergillus posadasensis*** Y. Marín *et al.*, Int. J. Syst. Evol. Microbiol. 64: 2874. 2014. [MB803514]. — Type: CBS-H 21131. Ex-type: FMR 12168 = CBS 134259 = NBRC 109845. Infragen. class: subgen. *Fumigati*, sect. *Clavati*, ser. *Clavati*. Reproduction: homothallic; asexual morph unknown. ITS barcode: HG529483 (alternative markers: *BenA* = HG529481; *CaM* = HG529488; *RPB2* = HF954977).

***Aspergillus pragensis*** Hubka *et al.*, Med. Mycol. 52: 570. 2014. [MB800371]. — Type: PRM 922702. Ex-type: CCF 3962 = CBS 135591 = NRRL 62491 = IBT 32274. Infragen. class: subgen. *Circumdati*, sect. *Candidi*, ser. *Candidi*. Reproduction: asexual. ITS barcode: FR727138 (alternative markers: *BenA* = HE661604; *CaM* = FR751452; *RPB2* = LN849445).

***Aspergillus proliferans*** G. Sm., Trans. Brit. Mycol. Soc. 26: 26. 1943. [MB284312]. — Type: IMI 16105iii. Ex-type: CBS 121.45 = NRRL 1908 = IMI 016105ii = IMI 016105iii = IMI 16105 = LSHB BB.82 = MUCL 15625 = NCTC 6546 = QM 7462 = UC 4303 = WB 1908. Infragen. class: subgen. *Aspergillus*, sect. *Aspergillus*, ser. *Aspergillus*. Reproduction: homothallic (Hubka *et al.* 2013a). ITS barcode: EF652064 (alternative markers: *BenA* = EF651891; *CaM* = EF651988; *RPB2* = EF651941).

***Aspergillus protuberus*** Munt.-Cvetk., Mikrobiologia 5: 119. 1968. [MB326650]. — Type: CBS 602.74. Ex-type: CBS 602.74 = NRRL 3505 = ATCC 18990 = QM 9804. Infragen. class: subgen. *Nidulantes*, sect. *Nidulantes*, ser. *Versicolores*. Reproduction: asexual. ITS barcode: EF652460 (alternative markers: *BenA* = EF652284; *CaM* = EF652372; *RPB2* = EF652196).

***Aspergillus pseudocaelatus*** Varga *et al.*, Stud. Mycol. 69: 63. 2011. [MB560397]. — Type: CBS H-20632. Ex-type: CBS 117616 = DTO 010-H4. Infragen. class: subgen. *Circumdati*, sect. *Flavi*, ser. *Kitamyces*. Reproduction: protoheterothallic; MAT1-1-1 detected (Carvajal-Campos *et al.* 2017). ITS barcode: EF409242 (alternative markers: *BenA* = MG517626; *CaM* = MG517995; *RPB2* = MG517809).

***Aspergillus pseudodeflectus*** Samson & Mouch., Antonie van Leeuwenhoek 40: 345. 1975. [MB309236]. — Type: CBS 756.74. Ex-type: CBS 756.74 = NRRL 6135. Infragen. class: subgen. *Nidulantes*, sect. *Usti*, ser. *Calidousti*. Reproduction: asexual. ITS barcode: EF652507 (alternative markers: *BenA* = EF652331; *CaM* = EF652419; *RPB2* = EF652243).

***Aspergillus pseudoelegans*** Frisvad & Samson, Stud. Mycol. 50: 35. 2004. [MB500005]. — Type: CBS H-13439. Ex-type: CBS 112796 = DTO 077-F5 = NRRL 35670 = IBT 23402. Infragen. class: subgen. *Circumdati*, sect. *Circumdati*, ser. *Steyniorum*. Reproduction: asexual. ITS barcode: FJ491590 (alternative markers: *BenA* = EU014095; *CaM* = FJ491552; *RPB2* = EF661281).

***Aspergillus pseudoglaucus*** Blochwitz, Ann. Mycol. 27: 207. 1929. [MB275429]. — Type: IMI 016122ii. Ex-type: CBS 123.28 = NRRL 40 = ATCC 10066 = IMI 16122 = IMI 016122ii = LSHBA 19 = MUCL 15624 = QM 7463 = WB 40. Infragen. class: subgen. *Aspergillus*, sect. *Aspergillus*, ser. *Rubri*. Reproduction: homothallic. ITS barcode: EF652050 (alternative markers: *BenA* = EF651917; *CaM* = EF652007; *RPB2* = EF651952).

***Aspergillus pseudogracilis*** F. Sklenář *et al.*, Stud. Mycol. 88: 216. 2017. [MB818932]. — Type: PRM 944434. Ex-type: CCF 5505 = EMSL No. 2765 = DTO 356-F3 = NRRL 66620 = IBT 34813. Infragen. class: subgen. *Aspergillus*, sect. *Restricti*, ser. *Restricti*. Reproduction: asexual. ITS barcode: KY087634 (alternative markers: *BenA* = KY117702; *CaM* = KY068186; *RPB2* = KY117879).

***Aspergillus pseudonomiae*** [as “*pseudonomius*”] Varga *et al.*, Stud. Mycol. 69: 67. 2011. [MB833325]. — Type: CBS H-20633. Ex-type: CBS 119388 = DTO 009-F1 = NRRL 3353 = IBT 27864. Infragen. class: subgen. *Circumdati*, sect. *Flavi*, ser. *Nomiarum*. Reproduction: asexual. ITS barcode: AF338643 (alternative markers: *BenA* = EF661495; *CaM* = EF661529; *RPB2* = EF661454).

***Aspergillus pseudosclerotiorum*** J.P.Z. Siqueira *et al.*, J. Clin. Microbiol. 55: 950. 2017. [MB818572]. — Type: CBS H-22808. Ex-type: UTHSCSA DI15-13 = FMR 14449 = CBS 141845. Infragen. class: subgen. *Circumdati*, sect. *Circumdati*, ser. *Sclerotiorum*. Reproduction: asexual. ITS barcode: LT574713 (alternative markers: *BenA* = LT574748; *CaM* = LT574783; *RPB2* = LT574818).

***Aspergillus pseudotamarii*** Yoko Ito *et al.*, Mycol. Res. 105: 237. 2001. [MB466527]. — Type: BPI 746098. Ex-type: CBS 766.97 = DTO 046-C1 = NRRL 25517. Infragen. class: subgen. *Circumdati*, sect. *Flavi*, ser. *Kitamyces*. Reproduction: protoheterothallic; MAT 1-1-1 detected (Carvajal-Campos *et al.* 2017). ITS barcode: AF272574 (alternative markers: *BenA* = EF203125; *CaM* = EF202030; *RPB2* = EU021631).

***Aspergillus pseudoterreus*** S.W. Peterson *et al.*, Stud. Mycol. 69: 53. 2011. [MB560396]. — Type: CBS H-20631. Ex-type: CBS 123890 = NRRL 4017. Infragen. class: subgen. *Circumdati*, sect. *Terrei*, ser. *Terrei*. Reproduction: asexual. ITS barcode: EF669598 (alternative markers: *BenA* = EF669523; *CaM* = EF669556; *RPB2* = EF669640).

***Aspergillus pseudoustus*** Frisvad *et al.*, Stud. Mycol. 69: 91. 2011. [MB560403]. — Type: CBS H-20637. Ex-type: CBS 123904 = NRRL 5856 = IBT 28161 = DTO 083-G3. Infragen. class: subgen. *Nidulantes*, sect. *Usti*, ser. *Usti*. Reproduction: asexual. ITS barcode: FJ531147 (alternative markers: *BenA* = FJ531168; *CaM* = FJ531129; *RPB2* = KU866978).

***Aspergillus pseudoviridinutans*** Sugui *et al.*, J. Clin. Microbiol. 52: 3709. 2014. [MB808637]. — Type: NRRL 62904. Ex-type: NRRL 62904 = NIH AV1 = CCF 5631 = DTO 304-I5. Infragen. class: subgen. *Fumigati*, sect. *Fumigati*, ser. *Viridinutantes*. Reproduction: protoheterothallic; both MAT idiomorphs detected (Hubka *et al.* 2018a). ITS barcode: MN431384 (alternative markers: *BenA* = KJ914690; *CaM* = KJ914708; *RPB2* = MN969102).

***Aspergillus pulvericola*** Visagie *et al.*, Stud. Mycol. 78: 43. 2014. [MB809200]. — Type: CBS H-21793. Ex-type: CBS 137327 = DTO 267-C6. Infragen. class: subgen. *Circumdati*, sect. *Circumdati*, ser. *Steyniorum*. Reproduction: asexual. ITS barcode: KJ775440 (alternative markers: *BenA* = KJ775055; *CaM* = KJ775236; *RPB2* = MN969088).

***Aspergillus pulvinus*** Kwon-Chung & Fennell, Gen. Aspergillus: 455. 1965. [MB326651]. — Type: IMI 139628. Ex-type: CBS 578.65 = NRRL 5078 = ATCC 16842 = IMI 139628 = QM 8937 = WB 5078. Infragen. class: subgen. *Cremei*, sect. *Cremei*, ser. *Pulvini*. Reproduction: asexual. ITS barcode: EF652159 (alternative markers: *BenA* = EF652121; *CaM* = EF652139; *RPB2* = EF652104).

***Aspergillus puniceus*** Kwon-Chung & Fennell, Gen. Aspergillus: 547. 1965. [MB326652]. — Type: IMI 126692. Ex-type: CBS 495.65 = NRRL 5077 = ATCC 16800 = IMI 126692 = QM 9812 = WB 5077. Infragen. class: subgen. *Nidulantes*, sect. *Usti*, ser. *Usti*. Reproduction: asexual. ITS barcode: EF652498 (alternative markers: *BenA* = EF652322; *CaM* = EF652410; *RPB2* = EF652234).

***Aspergillus purpureocrustaceus*** Visagie, Stud. Mycol., this issue. 2020. [MB834205]. — Type: PREM 62264. Ex-type: PPRI 3840 = CMV 008B3. Infragen. class: subgen. *Nidulantes*, sect. *Nidulantes*, ser. *Multicolores*. Reproduction: asexual. ITS barcode: MK450653 (alternative markers: *BenA* = MK451138; *CaM* = MK451515; *RPB2* = MK450806).

***Aspergillus purpureus*** Samson & Mouch., Antonie van Leeuwenhoek 41: 350. 1975. [MB309237]. — Type: CBS 754.74. Ex-type: CBS 754.74 = NRRL 6133 = IMI 334937 = LCP 82.3323. Infragen. class: subgen. *Nidulantes*, sect. *Nidulantes*, ser. *Aurantiobrunnei*. Reproduction: homothallic. ITS barcode: EF652506 (alternative markers: *BenA* = EF652330; *CaM* = EF652418; *RPB2* = EF652242).

***Aspergillus puulaauensis*** Jurjević *et al.*, IMA Fungus 3: 71. 2012. [MB800602]. — Type: BPI 880911. Ex-type: CBS 145750 = NRRL 35641 = DTO 225-G5. Infragen. class: subgen. *Nidulantes*, sect. *Nidulantes*, ser. *Versicolores*. Reproduction: asexual. ITS barcode: JQ301893 (alternative markers: *BenA* = JN853979; *CaM* = JN854034; *RPB2* = JN853823).

***Aspergillus qinqixianii*** Y. Horie *et al.*, Mycoscience 41: 183. 2000. [MB464660]. — Type: CBM-FA-0866. Ex-type: IFM 55020 = CBM-FA-0866 = DTO 098-H6. Infragen. class: subgen. *Nidulantes*, sect. *Nidulantes*, ser. *Stellati*. Reproduction: homothallic. ITS barcode: KU866980 (alternative markers: *BenA* = AB524360; *CaM* = AB524051; *RPB2* = KU866980).

***Aspergillus qizutongii*** D.M. Li *et al.*, Mycoscience 39: 301. 1998. [MB446576]. — Type: CBM FD-284. Ex-type: CBM FD-284. Infragen. class: subgen.: unknown, sect.: unknown, ser.: unknown. Reproduction: asexual. ITS barcode: n.a (alternative markers: *BenA* = n.a.; *CaM* = n.a.; *RPB2* = n.a.).

***Aspergillus quadricinctus*** E. Yuill, Trans. Brit. Mycol. Soc. 36: 57. 1953. [MB292857]. — Type: IMI 48583ii. Ex-type: CBS 135.52 = NRRL 2154 = ATCC 16897 = IMI 048583ii = IMI 48583 = QM 6874 = WB 2154 = DTO 050-E9 = DTO 164-I5. Infragen. class: subgen. *Fumigati*, sect. *Fumigati*, ser. *Brevipedes*. Reproduction: homothallic. ITS barcode: EF669947 (alternative markers: *BenA* = EF669806; *CaM* = EF669875; *RPB2* = EF669735).

***Aspergillus quadrilineatus*** Thom & Raper, Mycologia 31: 660. 1939. [MB275888]. — Type: IMI 89351. Ex-type: CBS 591.65 = NRRL 201 = ATCC 16816 = IMI 089351ii = IMI 89351 = LSHBA 546 = QM 7465 = Thom 4138.N8 = WB 201 = DTO 009-C7 = DTO 048-A9. Infragen. class: subgen. *Nidulantes*, sect. *Nidulantes*, ser. *Nidulantes*. Reproduction: homothallic. ITS barcode: EF652433 (alternative markers: *BenA* = EF652257; *CaM* = EF652345; *RPB2* = EF652169).

***Aspergillus raianus*** H.J. Chowdhery, Curr. Sci. 48: 953. 1979. [MB309239]. — Type: MLLU 110. Ex-type: n.a. Infragen. class: subgen.: unknown, sect.: unknown, ser.: unknown. Reproduction: asexual. ITS barcode: n.a. (alternative markers: *BenA* = n.a.; *CaM* = n.a.; *RPB2* = n.a.).

***Aspergillus rambellii*** Frisvad & Samson, Syst. Appl. Microbiol. 28: 449. 2005. [MB501209]. — Type: CBS 101887. Ex-type: CBS 101887 = ATCC 42001 = IBT 14580. Infragen. class: subgen. *Nidulantes*, sect. *Ochraceorosei*, ser. *Ochraceorosei*. Reproduction: asexual. ITS barcode: AJ874116 (alternative markers: *BenA* = JN217228; *CaM* = KU866700; *RPB2* = JN121416).

***Aspergillus raperi*** Stolk & J.A. Mey., Trans. Brit. Mycol. Soc. 40: 190. 1957. [MB292858]. — Type: [dried culture from soil] Zaire, Yangambi, Meyer (K). Ex-type: CBS 123.56 = NRRL 2641 = ATCC 16917 = IFO 6416 = IMI 70949 = NRRL 4778 = NRRL A-7462 = QM 1898 = WB 4221 = WB 4778. Infragen. class: subgen. *Nidulantes*, sect. *Raperorum*, ser. *Raperorum*. Reproduction: asexual. ITS barcode: EF652454 (alternative markers: *BenA* = EF652278; *CaM* = EF652366; *RPB2* = EF652190).

***Aspergillus recurvatus*** Raper & Fennell, Gen. Aspergillus: 529. 1965. [MB326653]. — Type: IMI 36528. Ex-type: CBS 496.65 = NRRL 4902 = ATCC 16809 = IMI 136528 = O-566 = QM 7972 = WB 4902. Infragen. class: subgen. *Nidulantes*, sect. *Nidulantes*, ser. *Nidulantes*. Reproduction: asexual. ITS barcode: EF652482 (alternative markers: *BenA* = EF652306; *CaM* = EF652394; *RPB2* = EF652218).

***Aspergillus restrictus*** G. Sm., J. Textile Inst. 22: 115. 1931. [MB276290]. — Type: IMI 16267. Ex-type: CBS 117.33 = CBS 541.65 = NRRL 154 = ATCC 16912 = B35855 = CECT2075 = IHEM 3920 = IMI 16267 = LSHBBB 94 = LSHTM 93 = MUCL 31313 = NCTC 6976 = NRRL 4155 = QM 1979 = Thom 5660.93 = UC4312 = VTTD-77065 = WB 154. Infragen. class: subgen. *Aspergillus*, sect. *Restricti*, ser. *Restricti*. Reproduction: asexual. ITS barcode: EF652042 (alternative markers: *BenA* = EF651880; *CaM* = EF652029; *RPB2* = EF651978).

***Aspergillus reticulatus*** F. Sklenář *et al.*, Stud. Mycol. 88: 219. 2017. [MB818940]. — Type: PRM 944442. Ex-type: NRRL 25852 = CCF 5516 = DTO 356-D4 = IBT 34540. Infragen. class: subgen. *Aspergillus*, sect. *Restricti*, ser. *Penicillioides*. Reproduction: asexual. ITS barcode: KY087765 (alternative markers: *BenA* = KY117828; *CaM* = KY068315; *RPB2* = KY118006).

***Aspergillus rhizopodus*** J.N. Rai *et al.*, Trans. Brit. Mycol. Soc. 64: 515. 1975. [MB309240]. — Type: Rai *et al.* 1975, Trans. Brit. Mycol. Soc. 64: p. 516, Fig. 1 (– lectotype designated here, MBT392286; CBS H-24284 [dried culture] – epitype designated here, MBT392287). Ex-epitype: CBS 450.75 = IMI 385057 = WB 5442 = NRRL 6136. Infragen. class: subgen. *Fumigati*, sect. *Clavati*, ser. *Clavati*. Reproduction: asexual. ITS barcode: EU078652 (alternative markers: *BenA* = EU076327; *CaM* = EF669926; *RPB2* = MN969089).

***Aspergillus robustus*** M. Chr. & Raper, Mycologia 70: 200. 1978. [MB309241]. — Type: NY WB 5286. Ex-type: CBS 428.77 = NRRL 6362 = ATCC 36106 = IMI 216610 = NRRL A-17351 = WB 5286. Infragen. class: subgen. *Circumdati*, sect. *Robusti*, ser. *Robusti*. Reproduction: asexual. ITS barcode: EF661176 (alternative markers: *BenA* = EU014101; *CaM* = EF661357; *RPB2* = EF661033).

***Aspergillus roseoglobulosus*** Frisvad & Samson, Stud. Mycol. 50: 30. 2004. [MB500001]. — Type: CBS H-13438. Ex-type: CBS 112800 = NRRL 4565 = IBT 14720. Infragen. class: subgen. *Circumdati*, sect. *Circumdati*, ser. *Sclerotiorum*. Reproduction: asexual. ITS barcode: FJ491583 (alternative markers: *BenA* = AY819984; *CaM* = FJ491555; *RPB2* = EF661299).

***Aspergillus ruber*** (Jos. König *et al.*) Thom & Church, Aspergillus: 112. 1926. [MB490579]. Basionym: *Eurotium rubrum* Jos. König *et al.*, Z. Untersuch. Nahr. Genusm.: 726. 1901. [MB219613]. — Type: CBS 530.65. Ex-type: CBS 530.65 = NRRL 52 = ATCC 16441 = IMI 211380 = QM 1973 = Thom 5599B = WB 52. Infragen. class: subgen. *Aspergillus*, sect. *Aspergillus*, ser. *Rubri*. Reproduction: homothallic. ITS barcode: EF652066 (alternative markers: *BenA* = EF651920; *CaM* = EF652009; *RPB2* = EF651947).

***Aspergillus rugulosus*** Thom & Raper, Mycologia 31: 660. 1939. [MB277104]. — Type: IMI 136775. Ex-type: CBS 133.60 = NRRL 206 = ATCC 16820 = IMI 136775 = QM 1987 = Thom 4138.T11 = WB 206. Infragen. class: subgen. *Nidulantes*, sect. *Nidulantes*, ser. *Nidulantes*. Reproduction: homothallic. ITS barcode: EF652434 (alternative markers: *BenA* = EF652258; *CaM* = EF652346; *RPB2* = EF652170).

***Aspergillus saccharolyticus*** Sørensen *et al.*, Int. J. Syst. Evol. Microbiol. 61: 3081. 2011. [MB518695]. — Type: Sørenson *et al.* 2011, Int. J. Syst. Evol. Microbiol. 61: p. 3081, Fig. 2 (– lectotype designated here, MBT392288; CBS H-24285 [dried culture] – epitype designated here, MBT392289). Ex-epitype: CBS 127449 = IBT 28509. Infragen. class: subgen. *Circumdati*, sect. *Nigri*, ser. *Japonici*. Reproduction: asexual. ITS barcode: HM853552 (alternative markers: *BenA* = HM853553; *CaM* = HM853554; *RPB2* = HF559235).

***Aspergillus salinarum*** [as “*salinarus*”] (Greiner *et al.*) Zalar & Greiner, Extremophiles 21: 762. 2017. [MB818567]. Basionym: *Phialosimplex salinarum* Greiner *et al.*, IMA Fungus 5: 166. 2014. [MB809044]. — Type: CBS H-23061. Ex-type: CBS142047 = EXF-10247. Infragen. class: subgen. *Polypaecilum*, sect. *Polypaecilum*, ser. *Salinarum*. Reproduction: asexual. ITS barcode: KY980619 (alternative markers: *BenA* = KY980547; *CaM* = KY980583; *RPB2* = KY980445).

***Aspergillus salinicola*** Zalar *et al.*, Stud. Mycol. 88: 221. 2017. [MB818941]. — Type: PRM 944448. Ex-type: EXF-10401 = IBT 34266 = CCF 5526 = NRRL 66621. Infragen. class: subgen. *Aspergillus*, sect. *Restricti*, ser. *Penicillioides*. Reproduction: asexual. ITS barcode: KY087722 (alternative markers: *BenA* = KY117785; *CaM* = KY068272; *RPB2* = KY117963).

***Aspergillus salisburgensis*** Zalar *et al.*, Extremophiles 21:762. 2017. [MB818564]. — Type: CBS H-23061. Ex-type: EXF-10247 = CBS 142047 = DTO 410-E7. Infragen. class: subgen. *Polypaecilum*, sect. *Polypaecilum*, ser. *Salinarum*. Reproduction: asexual. ITS barcode: KX900623 (alternative markers: *BenA* = MN969414; *CaM* = MN969324; *RPB2* = MN969191).

***Aspergillus salwaensis*** Visagie *et al.*, Stud. Mycol. 78: 49. 2014. [MB809201]. — Type: QCC F001/14. Ex-type: CBS 138172 = DTO 297-B3. Infragen. class: subgen. *Circumdati*, sect. *Circumdati*, ser. *Sclerotiorum*. Reproduction: asexual. ITS barcode: KJ775447 (alternative markers: *BenA* = KJ775056; *CaM* = KJ775244; *RPB2* = MN969090).

***Aspergillus savannensis*** A.J. Chen *et al.*, Stud. Mycol. 84: 89. 2016. [MB816096]. — Type: CBS H-22495. Ex-type: CBS 140607 = IBT 23422 = DTO 059-H6. Infragen. class: subgen. *Nidulantes*, sect. *Nidulantes*, ser. *Nidulantes*. Reproduction: homothallic. ITS barcode: KU866581 (alternative markers: *BenA* = KU866818; *CaM* = KU866704; *RPB2* = KU866959).

***Aspergillus sclerotialis*** (W. Gams & Breton) Houbraken *et al.*, Stud. Mycol. 78: 157. 2014. [MB809596]. Basionym: *Sagenomella sclerotialis* W. Gams & Breton, Persoonia 10: 109. 1978. [MB323039]. — Type: CBS 366.77. Ex-type: CBS 366.77 = IAM 14794 = DTO 107-E2 = DTO 137-F4. Infragen. class: subgen. *Polypaecilum*, sect. *Polypaecilum*, ser. *Noonimiarum*. Reproduction: asexual. ITS barcode: KF267869 (alternative markers: *BenA* = KY980579; *CaM* = MN969232; *RPB2* = JN121505).

***Aspergillus sclerotiicarbonarius*** Noonim *et al.*, Int. J. Syst. Evol. Microbiol. 58: 1733. 2008. [MB504407]. — Type: Noonim *et al.* 2008, Int. J. Syst. Evol. Microbiol. 58: p. 1731, Fig. 2 (– lectotype designated here, MBT392290; CBS H-24286 [dried culture] – epitype designated here, MBT392291). Ex-epitype: CBS 121057 = IBT 121057. Infragen. class: subgen. *Circumdati*, sect. *Nigri*, ser. *Carbonarii*. Reproduction: Heterothallic (Darbyshir *et al.* 2013). ITS barcode: EU159216 (alternative markers: *BenA* = EU159229; *CaM* = EU159235; *RPB2* = MN969091).

***Aspergillus sclerotioniger*** Samson & Frisvad, Stud. Mycol. 50: 57. 2004. [MB500010]. — Type: CBS H-13433. Ex-type: CBS 115572 = IBT 22905. Infragen. class: subgen. *Circumdati*, sect. *Nigri*, ser. *Carbonarii*. Reproduction: protoheterothallic; unpublished (genome data, Vesth *et al.* 2018). ITS barcode: DQ900606 (alternative markers: *BenA* = FJ629304; *CaM* = FN594557; *RPB2* = HE984369).

***Aspergillus sclerotiorum*** G.A. Huber, Phytopathology 23: 306. 1933. [MB277707]. — Type: IMI 56673. Ex-type: CBS 549.65 = NRRL 415 = IBT 11931 = ATCC 16892 = DSM 870 = IFO 7542 = IMI 56732 = IMI 56673 = LCP 89.2594 = QM 6732 = Thom 5351 = WB 415. Infragen. class: subgen. *Circumdati*, sect. *Circumdati*, ser. *Sclerotiorum*. Reproduction: asexual. ITS barcode: EF661400 (alternative markers: *BenA* = EF661337; *CaM* = EF661384; *RPB2* = EF661287).

***Aspergillus seifertii*** Visagie, Stud. Mycol., this issue. 2020. [MB834206]. — Type: PREM 49066. Ex-type: PPRI 3211 = CMV 006F5. Infragen. class: subgen. *Fumigati*, sect. *Clavati*, ser. *Clavati*. Reproduction: asexual. ITS barcode: MK450647 (alternative markers: *BenA* = MK451093; *CaM* = MK451509; *RPB2* = MK450800).

***Aspergillus sergii*** P. Rodrigues, S.W. Peterson, Venâncio & N. Lima, Mycologia 104: 693. 2012. [MB561842]. — Type: MUM-H 10.219. Ex-type: CBS 130017 = DTO 223-C9 = DTO 223-D1. Infragen. class: subgen. *Circumdati*, sect. *Flavi*, ser. *Flavi*. Reproduction: protoheterothallic; MAT 1-2-1 detected (Soares *et al.* 2012). ITS barcode: JF412769 (alternative markers: *BenA* = MG517688; *CaM* = MG518059; *RPB2* = HM802985).

***Aspergillus serratalhadensis*** L.F. Oliveira *et al.*, Persoonia 40: 263. 2018. [MB824978]. — Type: URM 91189. Ex-type: URM 7866. Infragen. class: subgen. *Circumdati*, sect. *Nigri*, ser. *Japonici*. Reproduction: asexual. ITS barcode: MH169127 (alternative markers: *BenA* = LT993222; *CaM* = LT993223; *RPB2* = LT995971).

***Aspergillus sesamicola*** Visagie *et al.*, Stud. Mycol. 78: 52. 2014. [MB809202]. — Type: CBS H-21792. Ex-type: CBS 137324 = IBT 29314 = DTO 148-B4. Infragen. class: subgen. *Circumdati*, sect. *Circumdati*, ser. *Circumdati*. Reproduction: asexual. ITS barcode: KJ775437 (alternative markers: *BenA* = KJ775063; *CaM* = KJ775233; *RPB2* = MN969092).

***Aspergillus shendaweii*** (Yaguchi *et al.*) Samson *et al.*, Stud. Mycol. 78: 157. 2014. [MB809597]. Basionym: *Neosartorya shendaweii* Yaguchi *et al.*, Mycoscience 51: 260. 2010. [MB513151]. — Type: CBM FA-0958. Ex-type: IFM 57611 = CBS 128793 = DTO 148-G9. Infragen. class: subgen. *Fumigati*, sect. *Fumigati*, ser. *Neoglabri*. Reproduction: homothallic. ITS barcode: MN431374 (alternative markers: *BenA* = AB488754; *CaM* = AB488762; *RPB2* = LC367696).

***Aspergillus siamensis*** Manoch & Eamvijarn, Mycoscience 54: 403. 2013. [MB561946]. — Type: IFM 59793. Ex-type: IFM 59793 = KUFC 6349T = CCF 4685 = CBS 137452 = DTO 278-B6. Infragen. class: subgen. *Fumigati*, sect. *Fumigati*, ser. *Viridinutantes*. Reproduction: homothallic. ITS barcode: MN431375 (alternative markers: *BenA* = AB646989; *CaM* = AB776704; *RPB2* = MN969093).

***Aspergillus sigurros*** Visagie, Stud. Mycol., this issue. 2020. [MB834207]. — Type: PREM 62308. Ex-type: PPRI 15889 = CMV 005I4. Infragen. class: subgen. *Nidulantes*, sect. *Usti*, ser. *Calidousti*. Reproduction: asexual. ITS barcode: MK450650 (alternative markers: *BenA* = MK451066; *CaM* = MK451512; *RPB2* = MK450803).

***Aspergillus silvaticus*** Fennell & Raper, Mycologia 47: 83. 1955. [MB292859]. — Type: IMI 61456. Ex-type: CBS 128.55 = NRRL 2398 = ATCC 16843 = ATCC 46904 = IFO 8173 = IMI 61456 = NRRL A-3107 = QM 1912 = WB 2398. Infragen. class: subgen. *Nidulantes*, sect. *Silvatici*, ser. *Silvatici*. Reproduction: asexual. ITS barcode: EF652448 (alternative markers: *BenA* = EF652272; *CaM* = EF652360; *RPB2* = EF652184).

***Aspergillus similanensis*** Dethoup, Mycotaxon 131: 9. 2016. [MB810782]. — Type: BCC 75436. Ex-type: KUFA 0012 = KUFA 0013. Infragen. class: subgen. *Fumigati*, sect. *Fumigati*, ser. *Fennelliarum*. Reproduction: homothallic. ITS barcode: n.a. (alternative markers: *BenA* = KM095494; *CaM* = KC920701; *RPB2* = n.a.).

***Aspergillus sloanii*** Visagie *et al.*, Stud. Mycol. 78: 108. 2014. [MB809194]. — Type: CBS H-21811. Ex-type: CBS 138177 = DTO 245A1. Infragen. class: subgen. *Aspergillus*, sect. *Aspergillus*, ser. *Rubri*. Reproduction: homothallic. ITS barcode: KJ775540 (alternative markers: *BenA* = KJ775074; *CaM* = KJ775309; *RPB2* = KX463365).

***Aspergillus sojae*** Sakag. & K. Yamada ex Murak., Rep. Res. Inst. Brewing: 8. 1971. [MB292860]. — Type: IMI 191300. Ex-type: CBS 100928 = DTO 046-C3 = IMI 191300. Infragen. class: subgen. *Circumdati*, sect. *Flavi*, ser. *Flavi*. Reproduction: protoheterothallic; MAT 1-1-1 detected (Ramirez-Prado *et al.* 2008). ITS barcode: KJ175434 (alternative markers: *BenA* = KJ175494; *CaM* = KJ175550; *RPB2* = MG517831).

***Aspergillus solicola*** Samson *et al.*, Stud. Mycol. 78: 157. 2014 [MB809599]. Replaced synonym: *Neosartorya warcupii* S.W. Peterson *et al.*, Stud. Mycol. 59: 201. 2007. [MB505572]. — Type: NRRL 35723. Ex-type: NRRL 35723 = DTO 047-E8. Infragen. class: subgen. *Fumigati*, sect. *Fumigati*, ser. *Neoglabri*. Reproduction: homothallic. ITS barcode: EU220279 (alternative markers: *BenA* = MN969370; *CaM* = EU220284; *RPB2* = MN969104).

***Aspergillus sparsus*** Raper & Thom, Mycologia 36: 572. 1944. [MB284314]. — Type: IMI 19394. Ex-type: CBS 139.61 = NRRL 1933 = ATCC 16851 = IHEM 4377 = IMI 19394 = IMI 19394ii = MUCL 31314 = NCTC 6975 = QM 7470 = WB 1933. Infragen. class: subgen. *Nidulantes*, sect. *Sparsi*, ser. *Sparsi*. Reproduction: asexual. ITS barcode: EF661181 (alternative markers: *BenA* = EF661125; *CaM* = EF661173; *RPB2* = EF661071).

***Aspergillus spathulatus*** Takada & Udagawa, Mycotaxon 24: 396. 1985. [MB104019]. — Type: NHL 2947. Ex-type: NRRL 20549 = ATCC 64222 = NHL 2948. Infragen. class: subgen. *Fumigati*, sect. *Fumigati*, ser. *Spathulati*. Reproduction: Heterothallic. ITS barcode: EF669943 (alternative markers: *BenA* = EF669803; *CaM* = EF669872; *RPB2* = EF669731).

***Aspergillus spectabilis*** M. Chr. & Raper, Mycologia 70: 333. 1978. [MB309243]. — Type: NY RMFH 429. Ex-type: CBS 429.77 = NRRL 6363 = ATCC 36105 = IMI 216611 = RMFH429. Infragen. class: subgen. *Nidulantes*, sect. *Aenei*, ser. *Aenei*. Reproduction: homothallic. ITS barcode: EF652510 (alternative markers: *BenA* = EU482437; *CaM* = EF652422; *RPB2* = EF652246).

***Aspergillus spelaeus*** A. Nováková *et al.*, Mycologia 107: 194. 2015. [MB808146]. — Type: PRM 923462. Ex-type: CCF 4425 = CMF ISB 2615 = CBS 134371 = NRRL 62826. Infragen. class: subgen. *Circumdati*, sect. *Flavipedes*, ser. *Spelaei*. Reproduction: asexual. ITS barcode: HG915905 (alternative markers: *BenA* = HG916698; *CaM* = HG916741; *RPB2* = HG916719).

***Aspergillus spelunceus*** [as “*speluneus*”] Raper & Fennell, Gen. Aspergillus: 457. 1965. [MB326656]. — Type: IMI 211389. Ex-type: CBS 497.65 = NRRL 4989 = ATCC 16838 = IMI 211389 = NRRL A-3676 = QM 8898 = WB 4989. Infragen. class: subgen. *Nidulantes*, sect. *Nidulantes*, ser. *Speluncei*. Reproduction: asexual. ITS barcode: EF652490 (alternative markers: *BenA* = EF652314; *CaM* = EF652402; *RPB2* = EF652226).

***Aspergillus spinosus*** Kozak., Mycol. Pap. 161: 58. 1989. [MB127763]. — Type: IMI 211390. Ex-type: CBS 483.65 = NRRL 5034 = ATCC 16898 = IFO 8782 = IMI 211390 = NRRL A-1148 = QM 8888 = WB 5034 = DTO 050-D9. Infragen. class: subgen. *Fumigati*, sect. *Fumigati*, ser. *Fumigati*. Reproduction: homothallic. ITS barcode: EF669988 (alternative markers: *BenA* = EF669844; *CaM* = EF669914; *RPB2* = EF669775).

***Aspergillus spinulosporus*** Hubka *et al.*, Plant Syst. Evol. 302: 1290. 2016. [MB816282]. Replaced synonym: *Aspergillus nidulans* var. *echinulatus* Fennell & Raper, Mycologia 47: 79 1955. [MB346543]. — Type: IMI 061454. Ex-type: CBS 120.55 = NRRL 2395 = ATCC 16825 = IMI 61454 = LCP 84.2557 = QM 1909 = WB 2395. Infragen. class: subgen. *Nidulantes*, sect. *Nidulantes*, ser. *Nidulantes*. Reproduction: homothallic. ITS barcode: EF652445 (alternative markers: *BenA* = EF652269; *CaM* = EF652357; *RPB2* = EF652181).

***Aspergillus stella-maris*** Zalar *et al.*, Mycologia 100: 789. 2008. [MB507363]. — Type: CBS H-19887. Ex-type: CBS 113638 = IBT 23439 = DTO 011-A2. Infragen. class: subgen. *Nidulantes*, sect. *Nidulantes*, ser. *Stellati*. Reproduction: homothallic. ITS barcode: EU448269 (alternative markers: *BenA* = KU866886; *CaM* = EU443978; *RPB2* = KU866925).

***Aspergillus stellatus*** Curzi, Atti Reale Accad. Naz. Lincei, Rendiconti Cl. Sci. Fis.19: 428. 1934. [MB254841]. — Type: Bowenpilly near Secundarabad, s. coll., (K). Ex-type: CBS 598.65 = NRRL 1858 = ATCC 16819 = IMI 136778 = QM 6835 = WB 1858. Infragen. class: subgen. *Nidulantes*, sect. *Nidulantes*, ser. *Stellati*. Reproduction: homothallic. ITS barcode: EF652426 (alternative markers: *BenA* = EF652250; *CaM* = EF652338; *RPB2* = EF652162).

***Aspergillus stelliformis*** F. Sklenář *et al.*, Mycologia 112: 363. 2020. [MB832711]. — Type: PRM 951569. Ex-type: CCF 5375 = EMSL No. 2293 = NRRL 66885. Infragen. class: subgen. *Nidulantes*, sect. *Nidulantes*, ser. *Stellati*. Reproduction: homothallic. ITS barcode: MK713531 (alternative markers: *BenA* = MK695638; *CaM* = MK695649; *RPB2* = MK695660).

***Aspergillus stercorarius*** A.J. Chen *et al.*, Stud. Mycol. 84: 100. 2016. [MB816094]. — Type: CBS H-22496. Ex-type: CBS 428.93 = IBT 28024 = DTO 320-B3. Infragen. class: subgen. *Nidulantes*, sect. *Nidulantes*, ser. *Nidulantes*. Reproduction: homothallic; asexual morph unknown. ITS barcode: KU866625 (alternative markers: *BenA* = KU866865; *CaM* = KU866763; *RPB2* = KU867026).

***Aspergillus steynii*** Frisvad & Samson, Stud. Mycol. 50: 39. 2004. [MB500006]. — Type: CBS H-13445. Ex-type: CBS 112812 = NRRL 35675 = IBT 23096. Infragen. class: subgen. *Circumdati*, sect. *Circumdati*, ser. *Steyniorum*. Reproduction: asexual. ITS barcode: EF661416 (alternative markers: *BenA* = EF661347; *CaM* = EF661378; *RPB2* = JN121428).

***Aspergillus stramenius*** R.O. Novak & Raper, Gen. Aspergillus: 260. 1965. [MB326658]. — Type: IMI 172293. Ex-type: CBS 498.65 = NRRL 4652 = ATCC 16895 = IFO 9611 = IMI 172293 = QM 8900 = WB 4652 = DTO 046-E3. Infragen. class: subgen. *Fumigati*, sect. *Fumigati*, ser. *Neoglabri*. Reproduction: homothallic. ITS barcode: EF669984 (alternative markers: *BenA* = EF669840; *CaM* = EF669910; *RPB2* = EF669771).

***Aspergillus striatus*** J.N. Rai *et al.*, Canad. J. Bot. 42: 1521. 1964. [MB326659]. — Type: IMI 96679. Ex-type: CBS 283.67 = CBS 592.65 = NRRL 4699 = ATCC 16815 = IMI 96679 = QM 8901 = WB 4699. Infragen. class: subgen. *Nidulantes*, sect. *Nidulantes*, ser. *Nidulantes*. Reproduction: homothallic; asexual morph unknown. ITS barcode: EF652470 (alternative markers: *BenA* = EF652294; *CaM* = EF652382; *RPB2* = EF652206).

***Aspergillus stromatoides*** Raper & Fennell, Gen. Aspergillus: 421. 1965. [MB326660]. — Type: IMI 123750. Ex-type: CBS 500.65 = DTO 059-B3 = DTO 080-G9 = NRRL 4519 = ATCC 16854 = ATCC 24485 = IMI 123750 = QM 8959 = QM 8974 = WB 4519. Infragen. class: subgen. *Cremei*, sect. *Cremei*, ser. *Cremei*. Reproduction: asexual, *Chaetosartorya stromatoides* B.J. Wiley & E.G. Simmons, a putative sexual morph of *A*. *stromatiodes*, is not conspecific with this species as recognised by Peterson (2008). ITS barcode: EF652146 (alternative markers: *BenA* = FJ531038; *CaM* = EF652127; *RPB2* = EF652098).

***Aspergillus subalbidus*** Visagie *et al.*, Stud. Mycol. 78: 101. 2014. [MB809190]. — Type: CBS H-21807. Ex-type: CBS 567.65 = ATCC 16871 = IMI 230752 = NRRL 312 = DTO 045-D7 = DTO 045-D7. Infragen. class: subgen. *Circumdati*, sect. *Candidi*, ser. *Candidi*. Reproduction: asexual. ITS barcode: KJ866983 (alternative markers: *BenA* = MN969366; *CaM* = EF669551; *RPB2* = MN969094).

***Aspergillus subflavus*** Hubka *et al.*, Stud. Mycol. 93: 46. 2019. [MB823776]. — Type: CBS H-23364. Ex-type: CBS 143683 = DTO 326-E8 = S778 = CCF 4957 = NRRL 66254 = IBT 34939. Infragen. class: subgen. *Circumdati*, sect. *Flavi*, ser. *Flavi*. Reproduction: asexual. ITS barcode: MH279429 (alternative markers: *BenA* = MG517773; *CaM* = MG518143; *RPB2* = MG517964).

***Aspergillus sublatus*** Y. Horie, Trans. Mycol. Soc. Japan 20: 481. 1979. [MB118407]. — Type: IFM 4553. Ex-type: CBS 140630 = DTO 338-F7 = IFO 30906 = IMI 334870 = NBRC 30906 = IFM 4553. Infragen. class: subgen. *Nidulantes*, sect. *Nidulantes*, ser. *Nidulantes*. Reproduction: homothallic. ITS barcode: KU866683 (alternative markers: *BenA* = KU866920; *CaM* = KU866804; *RPB2* = KU867069).

***Aspergillus sublevisporus*** Someya *et al.*, Mycoscience 40: 405. 1999. [MB459822]. — Type: CBM PF-1207. Ex-type: CBS 128796 = IFM 53598 = DTO 148-H3. Infragen. class: subgen. *Fumigati*, sect. *Fumigati*, ser. *Fennelliarum*. Reproduction: homothallic. ITS barcode: MN431376 (alternative markers: *BenA* = AB488759; *CaM* = AB488767; *RPB2* = MN969095).

***Aspergillus subnutans*** A.J. Chen *et al.*, Stud. Mycol. 85: 83. 2016. [MB817726]. — Type: CBS H-22728. Ex-type: CBS 129386 = DTO 202–C2 = WSF 445 = IBT 34352. Infragen. class: subgen. *Fumigati*, sect. *Cervini*, ser. *Cervini*. Reproduction: asexual. ITS barcode: KX528456 (alternative markers: *BenA* = KX528454; *CaM* = KX528455; *RPB2* = KX528453).

***Aspergillus subramanianii*** Visagie *et al.*, Stud. Mycol. 78: 55. 2014. [MB809203]. — Type: CBS H-21791. Ex-type: CBS 138230 = NRRL 6161 = ATCC 18413. Infragen. class: subgen. *Circumdati*, sect. *Circumdati*, ser. *Sclerotiorum*. Reproduction: asexual. ITS barcode: EF661403 (alternative markers: *BenA* = EF661339; *CaM* = EF661397; *RPB2* = EF661289).

***Aspergillus subsessilis*** Raper & Fennell, Gen. Aspergillus: 530. 1965. [MB119551]. — Type: IMI 135820. Ex-type: CBS 502.65 = NRRL 4905 = ATCC 16808 = IMI 135820 = O-325 = QM 8035 = WB 4905. Infragen. class: subgen. *Nidulantes*, sect. *Cavernicolarum*, ser. *Cavernicolarum*. Reproduction: asexual. ITS barcode: EF652485 (alternative markers: *BenA* = EF652309; *CaM* = EF652397; *RPB2* = EF652221).

***Aspergillus subunguis*** Wadhwani *et al.*, Curr. Sci. 53: 444. 1984. [MB105934]. — Type: IMI 254637. Ex-type: IMI 254637. Infragen. class: subgen. *Nidulantes*, sect.: unknown, ser.: unknown. Reproduction: asexual. ITS barcode: n.a. (alternative markers: *BenA* = n.a.; *CaM* = n.a.; *RPB2* = n.a.).

***Aspergillus subversicolor*** Jurjević *et al.*, IMA Fungus 3: 72. 2012. [MB800603]. — Type: BPI 880918. Ex-type: CBS 145751 = NRRL 58999 = DTO 225-G9. Infragen. class: subgen. *Nidulantes*, sect. *Nidulantes*, ser. *Versicolores*. Reproduction: asexual. ITS barcode: JQ301894 (alternative markers: *BenA* = JN853970; *CaM* = JN854010; *RPB2* = JN853799).

***Aspergillus sulphureoviridis*** A.J. Chen *et al.*, Stud. Mycol. 84: 103. 2016. [MB816097]. — Type: CBS H-22497. Ex-type: CBS 140626 = IBT 21868 = DTO 325-D1). Infragen. class: subgen. *Nidulantes*, sect. *Nidulantes*, ser. *Nidulantes*. Reproduction: asexual. ITS barcode: KU866673 (alternative markers: *BenA* = KU866911; *CaM* = KU866793; *RPB2* = KU867058).

***Aspergillus suttoniae*** J.P.Z. Siqueira *et al.*, Mycosis 61: 820. 2018. [MB823689]. — Type: CBS H-23243. Ex-type: UTHSCSA DI14-215 = FMR 13523. Infragen. class: subgen. *Circumdati*, sect. *Flavipedes*, ser. *Flavipedes*. Reproduction: asexual. ITS barcode: LT899487 (alternative markers: *BenA* = LT899536; *CaM* = LT899589; *RPB2* = LT899644).

***Aspergillus sydowii*** (Bainier & Sartory) Thom & Church, Aspergilli: 147. 1926. [MB279636]. Basionym: *Sterigmatocystis sydowii* Bainier & Sartory, Ann. Mycol. 11: 25. 1913. [MB197979]. — Type: IMI 211384. Ex-type: CBS 593.65 = NRRL 250 = IMI 211384 = NRRL 254. Infragen. class: subgen. *Nidulantes*, sect. *Nidulantes*, ser. *Versicolores*. Reproduction: protoheterothallic; MAT1-2-1 detected (de Vries *et al.* 2017). ITS barcode: EF652450 (alternative markers: *BenA* = EF652274; *CaM* = EF652362; *RPB2* = EF652186).

***Aspergillus tabacinus*** Nakaz. *et al.*, J. Agric. Chem. Soc. Japan 10: 177. 1934. [MB539544]. — Type: Unknown. Ex-type: CBS 122718 = CBS H-24287 = NRRL 4791 = IFO 4098 = QM 9766 = WB 4791. Infragen. class: subgen. *Nidulantes*, sect. *Nidulantes*, ser. *Versicolores*. Reproduction: asexual. ITS barcode: EF652478 (alternative markers: *BenA* = EF652302; *CaM* = EF652390; *RPB2* = EF652214).

***Aspergillus taichungensis*** Yaguchi *et al.*, Mycoscience 36: 421 1995. [MB434473]. — Type: CBM PF-1167. Ex-type: IBT 19404 = DTO 031-C6. Infragen. class: subgen. *Circumdati*, sect. *Candidi*, ser. *Candidi*. Reproduction: asexual. ITS barcode: MN431377 (alternative markers: *BenA* = MN969367; *CaM* = EU076310; *RPB2* = MN969096).

***Aspergillus takadae*** Y. Horie *et al.*, Mycoscience 60: 358. 2019. [MB827072]. — Type: CBM-FA-929-3H. Ex-type: IFM 62979 = CBM-FA-929-1. Infragen. class: subgen. *Fumigati*, sect. *Fumigati*, ser. *Spathulati*. Reproduction: Heterothallic. ITS barcode: n.a. (alternative markers: *BenA* = LC367646; *CaM* = LC367657; *RPB2* = LC367699).

***Aspergillus takakii*** Y. Horie *et al.*, Mycoscience 42: 91. 2001. [MB467818]. — Type: CBM FA-884. Ex-type: CBM FA-884 = IFM 53599 = CBS 137454 = DTO 278-B8. Infragen. class: subgen. *Fumigati*, sect. *Fumigati*, ser. *Fumigati*. Reproduction: homotallic. ITS barcode: MN431378 (alternative markers: *BenA* = AB787221; *CaM* = AB787566; *RPB2* = MN969097).

***Aspergillus tamarii*** Kita, Centralbl. Bakteriol. 2. Abth. 37: 433. 1913. [MB191425]. — Type: CBS 104.13. Ex-type: CBS 104.13 = NRRL 20818 = QM 9374. Infragen. class: subgen. *Circumdati*, sect. *Flavi*, ser. *Kitamyces*. Reproduction: protoheterothallic; MAT 1-1-1 detected (Ramirez-Prado *et al.* 2008). ITS barcode: AF004929 (alternative markers: *BenA* = EF661474; *CaM* = EF661526; *RPB2* = EU021629).

***Aspergillus tamarindosoli*** A.J. Chen *et al.*, Stud. Mycol. 88: 123. 2017. [MB818737]. — Type: CBS H-22826. Ex-type: CBS 141775 = DTO 054-A8 = IBT 34432. Infragen. class: subgen. *Aspergillus*, sect. *Aspergillus*, ser. *Tamarindosolorum*. Reproduction: homothallic. ITS barcode: LT670981 (alternative markers: *BenA* = LT671191; *CaM* = LT671192; *RPB2* = LT671193).

***Aspergillus tanneri*** Kwon-Chung *et al.*, J. Clin. Microbiol. 50: 3312. 2012. [MB801149]. — Type: BPI 882529. Ex-type: NRRL 62426 = NIH 1005 = ATCC MYA-4905. Infragen. class: subgen. *Circumdati*, sect. *Tannerorum*, ser. *Tannerorum*. Reproduction: asexual. ITS barcode: JN853798 (alternative markers: *BenA* = JN896582; *CaM* = JN896583; *RPB2* = JN896585).

***Aspergillus tapirirae*** C. Ram & A. Ram, Atti Reale Accad. Sci. Napoli 41: 100. 1972. [MB309245]. — Type: IMUFPe 2175. Ex-type: unknown. Infragen. class: subgen. *Aspergillus*, sect. *Restricti*, ser.: unknown. Reproduction: asexual. ITS barcode: n.a. (alternative markers: *BenA* = n.a.; *CaM* = n.a.; *RPB2* = n.a.).

***Aspergillus tardicrescens*** F. Sklenář *et al.*, Stud. Mycol. 88: 221. 2017. [MB818942]. — Type: PRM 944439. Ex-type: DTO 316-B5 = CCF 5529 = IBT 34558 = NRRL 66623). Infragen. class: subgen. *Aspergillus*, sect. *Restricti*, ser. *Penicillioides*. Reproduction: asexual. ITS barcode: KY087710 (alternative markers: *BenA* = KY117772; *CaM* = KY068259; *RPB2* = KY117951).

***Aspergillus tardus*** Bissett & Widden, Canad. J. Bot. 62: 2521. 1984. [MB105071]. — Type: DAOM 183872. Ex-type: CBS 433.93 = DAOM 175187 (representative strain). Infragen. class: subgen. *Cremei*, sect. *Cremei*, ser. *Inflati*. Reproduction: asexual. ITS barcode: FJ531045 (alternative markers: *BenA* = FJ531001; *CaM* = FJ531084; *RPB2* = n.a.).

***Aspergillus tasmanicus*** Hubka *et al.*, Plant Syst. Evol. 303: 801. 2017. [MB819519]. — Type: PRM 933840. Ex-type: CBS 283.66 = KACC 41141 = IBT 3211 = NBRC 8008. Infragen. class: subgen. *Fumigati*, sect. *Fumigati*, ser. *Unilaterales*. Reproduction: protoheterothallic; MAT 1-1-1 detected (Hubka *et al.* 2017). ITS barcode: AB185279 (alternative markers: *BenA* = AY685180; *CaM* = AY689367; *RPB2* = LN874010).

***Aspergillus tatenoi*** Y. Horie *et al.*, Trans. Mycol. Soc. Japan 33: 395. 1992. [MB358433]. — Type: CBM-FA 0022. Ex-type: CBM-FA 0022 = CBS 407.93 = NRRL 4584 = DTO 046-E4. Infragen. class: subgen. *Fumigati*, sect. *Fumigati*, ser. *Thermomutati*. Reproduction: homothallic. ITS barcode: EF669982 (alternative markers: *BenA* = EF669838; *CaM* = EF669908; *RPB2* = EF669769).

***Aspergillus templicola*** Visagie *et al.*, Stud. Mycol. 78: 103. 2014. [MB809191]. — Type: CBS H-21808. Ex-type: CBS 138181 = DTO 270-C6. Infragen. class: subgen. *Circumdati*, sect. *Flavipedes*, ser. *Flavipedes*. Reproduction: asexual. ITS barcode: KJ775545 (alternative markers: *BenA* = KJ775092; *CaM* = KJ775394; *RPB2* = KP987017).

***Aspergillus tennesseensis*** Jurjević *et al.*, IMA Fungus 3: 73. 2012. [MB800604]. — Type: BPI 880917. Ex-type: CBS 145752 = NRRL 13150 = DTO 225-F5. Infragen. class: subgen. *Nidulantes*, sect. *Nidulantes*, ser. *Versicolores*. Reproduction: asexual. ITS barcode: JQ301895 (alternative markers: *BenA* = JN853976; *CaM* = JN854017; *RPB2* = JN853806).

***Aspergillus teporis*** A.J. Chen *et al.*, Stud. Mycol. 88: 123. 2017. [MB818738]. — Type: CBS H-22821. Ex-type: CBS 141768 = DTO 058-E5 = IBT 34513. Infragen. class: subgen. *Aspergillus*, sect. *Aspergillus*, ser. *Teporium*. Reproduction: homothallic. ITS barcode: LT670982 (alternative markers: *BenA* = LT671194; *CaM* = LT671195; *RPB2* = LT671196).

***Aspergillus terreus*** Thom, Amer. J. Bot. 5: 85. 1918. [MB191719]. — Type: IMI 17294. Ex-type: CBS 601.65 = NRRL 255 = ATCC 10071 = ATCC 1012 = IFO 33026 = IMI 017294ii = IMI 17294 = JCM 10257 = LSHBA c .24 = MUCL 38640 = NCTC 981 = NRRL 543 = QM 1 = QM 1991 = Thom 144 = VKMF-67 = WB 255. Infragen. class: subgen. *Circumdati*, sect. *Terrei*, ser. *Terrei*. Reproduction: Heterothallic (Arabatzis & Velegraki 2013). ITS barcode: EF669586 (alternative markers: *BenA* = EF669519; *CaM* = EF669544; *RPB2* = EF669628).

***Aspergillus thailandensis*** Tanney *et al.*, Stud. Mycol. 88: 255. 2017. [MB822734]. — Type: DAOM 745798. Ex-type: DAOMC 251755 = UAMH 11840 = CBS 143383 = KAS 8126 = SLOAN 4554 = PN10TH-749. Infragen. class: subgen. *Polypaecilum*, sect. *Polypaecilum*, ser. *Noonimiarum*. Reproduction: asexual. ITS barcode: KY980642 (alternative markers: *BenA* = KY980570; *CaM* = KY980606; *RPB2* = KY980467).

***Aspergillus thermomutatus*** (Paden) S.W. Peterson, Mycol. Res. 96: 549. 1992. [MB358403]. Basionym: *Aspergillus fischeri* var. *thermomutatus* Paden, Mycopathol. Mycol. Appl. 36: 161. 1968. [MB349035]. — Type: BPI 1108305. Ex-type: CBS 208.92 = NRRL 20748 = DTO 051-D7. Infragen. class: subgen. *Fumigati*, sect. *Fumigati*, ser. *Thermomutati*. Reproduction: homothallic. ITS barcode: EF669946 (alternative markers: *BenA* = EF669805; *CaM* = EF669874; *RPB2* = EF669734).

***Aspergillus thesauricus*** Hubka & A. Nováková, Int. J. Syst. Evol. Microbiol. 62: 2784. 2012. [MB564187]. — Type: PRM 860609. Ex-type: NRRL 62487 = CCF 4166 = CMFISB 2155. Infragen. class: subgen. *Nidulantes*, sect. *Usti*, ser. *Calidousti*. Reproduction: asexual. ITS barcode: HE615088 (alternative markers: *BenA* = HE615095; *CaM* = HE615120; *RPB2* = HE615126).

***Aspergillus togoensis*** (Henn.) Samson & Seifert, Adv. Pen. Asp. Syst.: 419. 1986 [1985]. [MB114720]. Basionym: *Stilbothamnium togoense* Henn., Bot. Jahrb. Syst. 23: 542. 1897. [MB374610]. — Type: BR B 1009. Ex-type: CBS 205.75 = NRRL 13551 = LCP 67.3456 (CBS 272.89 (representative strain)). Infragen. class: subgen. *Circumdati*, sect. *Flavi*, ser. *Coremiiformes*. Reproduction: asexual. ITS barcode: MN431379 (alternative markers: *BenA* = FJ491477; *CaM* = FJ491489; *RPB2* = JN121479).

***Aspergillus tonophilus*** Ohtsuki, Bot. Mag. (Tokyo) 75: 438. 1962. [MB326663]. — Type: IMI 108299. Ex-type: CBS 405.65 = NRRL 5124 = ATCC 16440 = ATCC 36504 = IMI 108299 = QM 8599 = WB 5124. Infragen. class: subgen. *Aspergillus*, sect. *Aspergillus*, ser. *Rubri*. Reproduction: homothallic. ITS barcode: EF652081 (alternative markers: *BenA* = EF651919; *CaM* = EF652000; *RPB2* = EF651969).

***Aspergillus transcarpathicus*** A.J. Chen *et al.*, Stud. Mycol. 85: 83. 2016. [MB817727]. — Type: CBS H-22727. Ex-type: CBS 423.68 = DTO 022-C7 = IBT 22080 = IMI 134108 = VKM F-1331. Infragen. class: subgen. *Fumigati*, sect. *Cervini*, ser. *Cervini*. Reproduction: asexual. ITS barcode: FJ491624 (alternative markers: *BenA* = FJ491632; *CaM* = FJ491610; *RPB2* = KX423680).

***Aspergillus transmontanensis*** P. Rodrigues *et al.*, Mycologia 104: 694. 2012. [MB561843]. — Type: MUM-H 10.214. Ex-type: DTO 223-C7 = CBS 130015. Infragen. class: subgen. *Circumdati*, sect. *Flavi*, ser. *Flavi*. Reproduction: protoheterothallic; both MAT idiomorphs detected (Soares *et al.* 2013). ITS barcode: JF412774 (alternative markers: *BenA* = HM803101; *CaM* = HM803020; *RPB2* = HM802980).

***Aspergillus trinidadensis*** Jurjević *et al.*, IMA Fungus 3: 170. 2012. [MB802364]. — Type: BPI 883908. Ex-type: DTO 198-D1 = NRRL 62479 = ITEM 14821. Infragen. class: subgen. *Circumdati*, sect. *Nigri*, ser. *Japonici*. Reproduction: asexual. ITS barcode: MN431380 (alternative markers: *BenA* = HE984420; *CaM* = HE984434; *RPB2* = HE984379).

***Aspergillus trisporus*** S.C. Souza *et al.*, Curr. Res. Environ. & Appl. Mycol. 9: 179. 2019. [MB822378]. — Type: CCDCA FI15. Ex-type: CML 3603. Infragen. class: subgen. *Circumdati*, sect. *Janorum*, ser. *Janorum*. Reproduction: asexual. ITS barcode: MF616388 (alternative markers: *BenA* = MF616387; *CaM* = MN013146; *RPB2* = MF616389).

***Aspergillus tritici*** [as “*triticus*”] B.S. Mehrotra & M. Basu, Nova Hedwigia 27: 599. 1976. [MB309248]. — Type: Mehrotra & Basu 1976, Nova Hedwigia 27: p. 603 Fig. 8 (– lectotype designated here, MBT392358; CBS H-24289 [dried culture] – epitype designated here, MBT392359). Ex-epitype: CBS 266.81 = DTO 031-F4. Infragen. class: subgen. *Circumdati*, sect. *Candidi*, ser. *Candidi*. Reproduction: asexual. ITS barcode: MN431381 (alternative markers: *BenA* = MN969368; *CaM* = MN969233; *RPB2* = MN969098).

***Aspergillus tsunodae*** (Yaguchi *et al.*) Samson *et al.*, Stud. Mycol. 78: 157. 2014. [MB809600]. Basionym: *Neosartorya tsunodae* Yaguchi *et al.*, Mycoscience 51: 261. 2010. [MB513152]. — Type: CBM FA-0950. Ex-type: IFM 57609 = NBRC 106416 = CBS 128794 = DTO 148-H1. Infragen. class: subgen. *Fumigati*, sect. *Fumigati*, ser. *Unilaterales*. Reproduction: homothallic. ITS barcode: HE974447 (alternative markers: *BenA* = AB488755; *CaM* = AB488763; *RPB2* = HE974400).

***Aspergillus tsurutae*** Y. Horie, Mycoscience 44: 399. 2003. [MB489534]. — Type: CBM FA-933. Ex-type: CBM FA-933 = CBS 137455 = IFM 56811 = DTO 279-D5. Infragen. class: subgen. *Fumigati*, sect. *Fumigati*, ser. *Brevipedes*. Reproduction: homothallic. ITS barcode: MN431382 (alternative markers: *BenA* = AB488760; *CaM* = AB488768; *RPB2* = MN969099).

***Aspergillus tubingensis*** Mosseray, La Cellule 43: 245. 1934. [MB255209]. — Type: Mosseray 1934, La Cellule 43: Pl. III Fig. 58 (– lectotype designated here, MBT392362; CBS H-24288 [dried culture] – epitype designated here, MBT392363). Ex-epitype: NRRL 4875 = QM 8904 = WB 4875 = CBS 133056. Infragen. class: subgen. *Circumdati*, sect. *Nigri*, ser. *Nigri*. Reproduction: Heterothallic (Horn *et al.* 2013). ITS barcode: EF661193 (alternative markers: *BenA* = EF661086; *CaM* = EF661151; *RPB2* = EF661055).

***Aspergillus tumidus*** J.P.Z. Siqueira *et al.*, Persoonia 40: 261. 2018. [MB823690]. — Type: CBS H-23244. Ex-type: FMR 15743 = CBS 143587. Infragen. class: subgen. *Nidulantes*, sect. *Nidulantes*, ser. *Multicolores*. Reproduction: asexual. ITS barcode: LT903691 (alternative markers: *BenA* = LT903682; *CaM* = LT903685; *RPB2* = LT903688).

***Aspergillus turcosus*** S.B. Hong *et al.*, Antonie van Leeuwenhoek 93: 97. 2008. [MB506378]. — Type: KACC 42091. Ex-type: KACC 42091 = DTO 035-E7. Infragen. class: subgen. *Fumigati*, sect. *Fumigati*, ser. *Unilaterales*. Reproduction: Heterothallic (Hubka *et al.* 2017). ITS barcode: MN431383 (alternative markers: *BenA* = DQ534143; *CaM* = DQ534148; *RPB2* = HF545310).

***Aspergillus turkensis*** Varga *et al.*, Stud. Mycol. 69: 91. 2011. [MB560404]. — Type: CBS H-20638. Ex-type: CBS 504.65 = NRRL A-3261 = NRRL 4993 = ATCC 16799 = IMI 135420. Infragen. class: subgen. *Nidulantes*, sect. *Usti*, ser. *Deflecti*. Reproduction: asexual. ITS barcode: FJ531160 (alternative markers: *BenA* = FJ531191; *CaM* = FJ531145; *RPB2* = EF652230).

***Aspergillus udagawae*** Y. Horie *et al.*, Mycoscience 36: 199. 1995. [MB412533]. — Type: CBM-FA-0711 (holotype); PRM 945579 (epitype, Hubka *et al.* 2018a). Ex-type: IFM 46972 = CBS 114217 = DTO 157-D7 = CBM-FA 0702 = KACC 41155 = CCF 4558. Infragen. class: subgen. *Fumigati*, sect. *Fumigati*, ser. *Viridinutantes*. Reproduction: Heterothallic. ITS barcode: AB185265 (alternative markers: *BenA* = LT796063; *CaM* = LT796064; *RPB2* = LT796065).

***Aspergillus undulatus*** H.Z. Kong & Z.T. Qi, Acta Mycol. Sin. 4: 211. 1985 [MB129004]. — Type: HMAS 47644. Ex-type: CBS 261.88 = DTO 011-A1. Infragen. class: subgen. *Nidulantes*, sect. *Nidulantes*, ser. *Stellati*. Reproduction: homothallic. ITS barcode: EU448275 (alternative markers: *BenA* = EF428363; *CaM* = EU443989; *RPB2* = KU866928).

***Aspergillus unguis*** (Émile-Weill & L. Gaudin) C.W. Dodge, Med. Mycol.: 637. 1935. [MB255264]. Basionym: *Sterigmatocystis unguis* Émile-Weill & L. Gaudin, Arch. Med. Exp. Anat. Pathol. 28: 463. 1918. [MB452891]. — Type: IMI 136526. Ex-type: CBS 132.55 = NRRL 2393 = ATCC 16812 = IMI 136526 = NRRL A-2391 = NRRL A-445 = QM 25B = WB 2393. Infragen. class: subgen. *Nidulantes*, sect. *Nidulantes*, ser. *Unguium*. Reproduction: asexual (?); contradictory (unpublished) data (Fennell & Raper 1955, Kakkar & Mehrotra 1971, Hubka *et al.* 2016a). ITS barcode: EF652443 (alternative markers: *BenA* = EF652267; *CaM* = EF652355; *RPB2* = EF652179).

***Aspergillus unilateralis*** Thrower, Austral. J. Bot. 2: 355. 1954. [MB292862]. — Type: IMI 62876. Ex-type: CBS 126.56 = NRRL 577 = ATCC 16902 = IFO 8136 = IMI 62876 = QM 8163 = WB 4366 = WB 4779 = DTO 001-E6 = DTO 050-F4. Infragen. class: subgen. *Fumigati*, sect. *Fumigati*, ser. *Unilaterales*. Reproduction: protoheterothallic; MAT 1-1-1 detected (Hubka *et al.* 2017). ITS barcode: EF669997 (alternative markers: *BenA* = EF669852; *CaM* = EF669923; *RPB2* = EF669784).

***Aspergillus urmiensis*** Arzanlou *et al.*, Mycol. Prog. 15: 1089. 2016. [MB817474]. — Type: CBS H-22671. Ex-type: CCTU 742 = C B S 139558 = IBT 32593 = DTO 203-C2. Infragen. class: subgen. *Circumdati*, sect. *Flavipedes*, ser. *Flavipedes*. Reproduction: asexual. ITS barcode: KP987073 (alternative markers: *BenA* = KP987041; *CaM* = KP987056; *RPB2* = KP987030).

***Aspergillus ustus*** (Bainier) Thom & Church, Aspergilli: 152. 1926. [MB281216]. Basionym: *Sterigmatocystis usta* Bainier, Bull. Soc. Bot. France 28: 78. 1881. [MB536545]. — Type: IMI 211805. Ex-type: CBS 261.67 = NRRL 275 = ATCC 1041 = ATCC 16818 = IMI 211805 = QM 7477 = WB 275. Infragen. class: subgen. *Nidulantes*, sect. *Usti*, ser. *Usti*. Reproduction: asexual. ITS barcode: EF652455 (alternative markers: *BenA* = EF652279; *CaM* = EF652367; *RPB2* = EF652191).

***Aspergillus uvarum*** G. Perrone *et al.*, Int. J. Syst. Evol. Microbiol. 58: 1036. 2008. [MB510962]. — Type: IMI 388523. Ex-type: CBS 121591 = IBT 26606 = IMI 388523 = ITEM 4834. Infragen. class: subgen. *Circumdati*, sect. *Nigri*, ser. *Japonici*. Reproduction: asexual. ITS barcode: AM745757 (alternative markers: *BenA* = AM745751; *CaM* = AM745755; *RPB2* = HE984370).

***Aspergillus vadensis*** R.P. de Vries *et al.*, Antonie van Leeuwenhoek 87: 201. 2005. [MB340234]. — Type: CBS 113365. Ex-type: CBS 113365 = CECT20584 = IMI 313493. Infragen. class: subgen. *Circumdati*, sect. *Nigri*, ser. *Nigri*. Reproduction: protoheterothallic (genome data, Vesth *et al.* 2018). ITS barcode: AY585549 (alternative markers: *BenA* = AY585531; *CaM* = FN594560; *RPB2* = HE984371).

***Aspergillus vandermerwei*** Frisvad *et al.*, Stud. Mycol. 93: 46. 2019. [MB823777]. — Type: CBS H-23381. Ex-type: CBS 612.78 = DTO 069-D2 = DTO 034-B5 = NRRL 5108 = CCF 5683 = IBT 13876. Infragen. class: subgen. *Circumdati*, sect. *Flavi*, ser. *Alliacei*. Reproduction: asexual. ITS barcode: EF661567 (alternative markers: *BenA* = EF661469; *CaM* = EF661540; *RPB2* = MG517838).

***Aspergillus varians*** Wehmer, Bot. Centralbl. 80: 460. 1899. [MB172782]. — Type: IMI 172297. Ex-type: CBS 505.65 = NRRL 4793 = ATCC 16836 = IFO 4114 = IMI 172297 = WB 4793. Infragen. class: subgen. *Nidulantes*, sect. *Nidulantes*, ser. *Speluncei*. Reproduction: asexual. ITS barcode: EF652479 (alternative markers: *BenA* = EF652303; *CaM* = EF652391; *RPB2* = EF652215).

***Aspergillus venenatus*** Jurjević *et al.*, IMA Fungus 3: 73. 2012. [MB800605]. — Type: BPI 880916. Ex-type: CBS 145753 = NRRL 13147 = DTO 225-F4. Infragen. class: subgen. *Nidulantes*, sect. *Nidulantes*, ser. *Versicolores*. Reproduction: asexual. ITS barcode: JQ301896 (alternative markers: *BenA* = JN854003; *CaM* = JN854014; *RPB2* = JN853803).

***Aspergillus venezuelensis*** Frisvad & Samson, Syst. Appl. Microbiol. 27: 678. 2004. [MB368544]. — Type: CBS 868.97. Ex-type: CBS 868.97 = IBT 20956 = DTO 011-A4. Infragen. class: subgen. *Nidulantes*, sect. *Nidulantes*, ser. *Stellati*. Reproduction: homothallic. ITS barcode: AJ874119 (alternative markers: *BenA* = AY339998; *CaM* = EU443977; *RPB2* = KU866931).

***Aspergillus versicolor*** (Vuill.) Tirab., Ann. Bot. (Roma) 7: 9. 1908 [MB172159]. Basionym: *Sterigmatocystis versicolor* Vuill., Erreur Dét. Asp. Paras. Homme: 15. 1903. [MB233198]. — Type: CBS 583.65. Ex-type: CBS 583.65 = NRRL 238 = ATCC 9577 = IFO 33027 = IMI 229970 = JCM 10258 = QM 7478 = Thom 5519.57 = WB 238. Infragen. class: subgen. *Nidulantes*, sect. *Nidulantes*, ser. *Versicolores*. Reproduction: protoheterothallic; MAT1-2-1 detected (de Vries *et al.* 2017). ITS barcode: EF652442 (alternative markers: *BenA* = EF652266; *CaM* = EF652354; *RPB2* = EF652178).

***Aspergillus villosus*** F. Sklenář *et al.*, Stud. Mycol. 88: 224. 2017. [MB818933]. — Type: PRM 944430. Ex-type: NRRL 25813 = CCF 5531 = DTO 356-C9 = IBT 34822. Infragen. class: subgen. *Aspergillus*, sect. *Restricti*, ser. *Restricti*. Reproduction: asexual. ITS barcode: KY087752 (alternative markers: *BenA* = KY117815; *CaM* = KY068302; *RPB2* = KY117993).

***Aspergillus vinosobubalinus*** Udagawa *et al.*, Trans. Mycol. Soc. Japan 34: 255. 1993. [MB361186]. — Type: CBM BF-33501. Ex-type: CBM BF-33501. Infragen. class: subgen.: unknown, sect.: unknown, ser.: unknown. Reproduction: asexual. ITS barcode: n.a (alternative markers: *BenA* = n.a.; *CaM* = n.a.; *RPB2* = n.a.).

***Aspergillus violaceus*** Fennell & Raper, Mycologia 47: 75. 1955. [MB292863]. — Type: IMI 61449. Ex-type: CBS 138.55 = NRRL 2240 = ATCC 16813 = CECT2587 = IFO 8106 = IMI 061449ii = IMI 61449 = LCP 82.3318 = NRRL A-3156 = QM 1905 = UC4511 = WB 2240. Infragen. class: subgen. *Nidulantes*, sect. *Nidulantes*, ser. *Nidulantes*. Reproduction: homothallic. ITS barcode: EF652438 (alternative markers: *BenA* = EF652262; *CaM* = EF652350; *RPB2* = EF652174).

***Aspergillus viridicatenatus*** A.J. Chen *et al.*, Stud. Mycol. 84: 112. 2016. [MB816088]. — Type: CBS H-22498. Ex-type: CBS 140629 = IBT 31492 = DTO 325-F4. Infragen. class: subgen. *Nidulantes*, sect. *Nidulantes*, ser. *Speluncei*. Reproduction: asexual. ITS barcode: KU866682 (alternative markers: *BenA* = KX423621; *CaM* = KU866802; *RPB2* = KU867067).

***Aspergillus viridinutans*** Ducker & Thrower, Austral. J. Bot. 2: 355. 1954. [MB292864]. — Type: IMI 62875. Ex-type: CBS 127.56 = NRRL 4365 = NRRL 4782 = NRRL 576 = NRRL A-16083 = NRRL A-6281 = ATCC 16901 = IMI 367415 = IMI 62875 = WB 4081 = WB 4365 = WB 4782 = DTO 050-F1. Infragen. class: subgen. *Fumigati*, sect. *Fumigati*, ser. *Viridinutantes*. Reproduction: protoheterothallic; MAT 1-1-1 detected (Nováková *et al.* 2014). ITS barcode: EF669978 (alternative markers: *BenA* = EF661252; *CaM* = DQ534162; *RPB2* = EF669765).

***Aspergillus vitricola*** [as “*vitricolae*”] Ohtsuki, Bot. Mag. (Tokyo) 75: 436. 1962. [MB326665]. — Type: No. 16 (Gi-4) (Herb. Nagao Institute). Ex-type: DTO 356-F7 = CBS H-24290 = CBS 146239 = NRRL 5125 = ATCC 16905 = ATCC 36505 = IMI 108298 = WB 5125. Infragen. class: subgen. *Aspergillus*, sect. *Restricti*, ser. *Vitricolarum*. Reproduction: asexual. ITS barcode: EF652046 (alternative markers: *BenA* = EF651927; *CaM* = EF652035; *RPB2* = EF651973).

***Aspergillus waksmanii*** Hubka *et al.*, Int. J. Syst. Evol. Microbiol. 63: 786. 2013. [MB801063]. — Type: PRM 860537. Ex-type: NRRL 179 = CCF 4266 = Thom 4138.H52 = IBT 31900 = DTO 239-D8. Infragen. class: subgen. *Fumigati*, sect. *Fumigati*, ser. *Unilaterales*. Reproduction: homothallic. ITS barcode: EF669934 (alternative markers: *BenA* = EF669794; *CaM* = EF669863; *RPB2* = EF669722).

***Aspergillus wangduanlii*** D.M. Li *et al.*, Mycoscience 39: 302. 1998. [MB447107]. — Type: CBM FD-283. Ex-type: CBM FD-283 = CMMB 2309. Infragen. class: subgen.: unknown, sect.: unknown, ser.: unknown. Reproduction: asexual. ITS barcode: n.a. (alternative markers: *BenA* = n.a.; *CaM* = n.a.; *RPB2* = n.a.).

***Aspergillus waynelawii*** Tanney *et al.*, Stud. Mycol. 88: 255. 2017. [MB822735]. — Type: DAOM 745796. Ex-type: DAOMC 251751 = UAMH 11926 = CBS 143384 = KAS 8123 = SLOAN 7951a = WL03MI-231. Infragen. class: subgen. *Polypaecilum*, sect. *Polypaecilum*, ser. *Noonimiarum*. Reproduction: asexual. ITS barcode: KY980639 (alternative markers: *BenA* = KY980567; *CaM* = KY980603; *RPB2* = KY980464).

***Aspergillus welwitschiae*** (Bres.) Henn., in Wehmer, Centralbl. Bakteriol. Parasitenk., 2. Abth. 18: 394. 1907. [MB490584]. Basionym: *Ustilago welwitschiae* Bres., in Saccardo, Bol. Soc. Brot. 11: 68. 1893. [MB176748]. — Type: CBS 139.54. Ex-type: CBS 139.54. Infragen. class: subgen. *Circumdati*, sect. *Nigri*, ser. *Nigri*. Reproduction: protoheterothallic; both MAT idiomorphs detected (Mageswari *et al.* 2016). ITS barcode: FJ629340 (alternative markers: *BenA* = MN969369; *CaM* = KC480196; *RPB2* = MN969100).

***Aspergillus wentii*** Wehmer, Centralbl. Bakteriol. Parasitenk., 2. Abth., 2: 149. 1896. [MB172623]. — Type: IMI 17295. Ex-type: CBS 104.07 = NRRL 375 = ATCC 1023 = IMI 17295 = NCTC 597 = NRRL 1269 = QM 7479 = Thom 116 = WB 375. Infragen. class: subgen. *Cremei*, sect. *Cremei*, ser. *Wentiorum*. Reproduction: protoheterothallic; MAT1-2-1 detected (de Vries *et al.* 2017). ITS barcode: EF652151 (alternative markers: *BenA* = EF652106; *CaM* = EF652131; *RPB2* = EF652092).

***Aspergillus westerdijkiae*** Frisvad & Samson, Stud. Mycol. 50: 30. 2004. [MB500000]. — Type: CBS H-13444. Ex-type: CBS 112803 = NRRL 3174 = IBT 10738 = ATCC 22947 = IBT 10738 = MUCL 39539. Infragen. class: subgen. *Circumdati*, sect. *Circumdati*, ser. *Circumdati*. Reproduction: asexual. ITS barcode: EF661427 (alternative markers: *BenA* = EF661329; *CaM* = EF661360; *RPB2* = EF661307).

***Aspergillus westlandensis*** Visagie *et al.*, Stud. Mycol. 78: 59. 2014. [MB809204]. — Type: CBS H-21795. Ex-type: CBS 137321 = IBT 32139 = DTO 231-A9. Infragen. class: subgen. *Circumdati*, sect. *Circumdati*, ser. *Circumdati*. Reproduction: asexual. ITS barcode: KJ775434 (alternative markers: *BenA* = KJ775066; *CaM* = KJ775230; *RPB2* = MN969101).

***Aspergillus whitfieldii*** Tanney *et al.*, Stud. Mycol. 88: 258. 2017. [MB822736]. — Type: DAOM 745799. Ex-type: DAOMC 251760 = UAMH 11842 = CBS 143385 = KAS 8129 = SLOAN 4178 = PN08TH-523. Infragen. class: subgen. *Polypaecilum*, sect. *Polypaecilum*, ser. *Whitfieldiorum*. Reproduction: asexual. ITS barcode: KY980645 (alternative markers: *BenA* = KY980573; *CaM* = KY980609; *RPB2* = KY980470).

***Aspergillus wisconsinensis*** A.J. Chen *et al.*, Stud. Mycol. 85: 86. 2016. [MB817728]. — Type: CBS H-9203. Ex-type: CBS 413.64 = DTO 022–B1 = NRRL 5027 = IBT 22042 = IBT 22082 = WSF 380 = DTO 070-A5 = WB 5027. Infragen. class: subgen. *Fumigati*, sect. *Cervini*, ser. *Cervini*. Reproduction: asexual. ITS barcode: FJ491618 (alternative markers: *BenA* = FJ491638; *CaM* = FJ491609; *RPB2* = KX423671).

***Aspergillus wyomingensis*** A. Nováková *et al.*, Fungal Diversity 64: 270. 2014. [MB803936]. — Type: PRM 861504. Ex-type: CCF 4417 = CMF ISB 2494 = CBS 135456 = DTO 332-B1 (= purified culture of DTO 311-F7). Infragen. class: subgen. *Fumigati*, sect. *Fumigati*, ser. *Viridinutantes*. Reproduction: Heterothallic. ITS barcode: HG324081 (alternative markers: *BenA* = HF933359; *CaM* = HF933397; *RPB2* = HF937378).

***Aspergillus xerophilus*** Samson & Mouch., Antonie van Leeuwenhoek 41: 348. 1975. [MB309251]. — Type: CBS 938.73. Ex-type: CBS 938.73 = NRRL 6131. Infragen. class: subgen. *Aspergillus*, sect. *Aspergillus*, ser. *Xerophili*. Reproduction: homothallic. ITS barcode: EF652085 (alternative markers: *BenA* = EF651923; *CaM* = EF651983; *RPB2* = EF651970).

***Aspergillus yunnanensis*** W.J. Cai *et al.*, Mycoscience 61: 72. 2020. [MB831500]. — Type: HMAS 248248. Ex-type: CGMCC 3.19711. Infragen. class: subgen. *Circumdati*, sect. *Janorum*, ser. *Janorum*. Reproduction: asexual. ITS barcode: MN066373 (alternative markers: *BenA* = MN072909; *CaM* = MN072911; *RPB2* = MN072913).

***Aspergillus zutongqii*** A.J. Chen *et al.*, Stud. Mycol. 88: 129. 2017. [MB818739]. — Type: CBS H-22824. Ex-type: CBS 141773 = CGMCC 3.13917 = DTO 349-E1 = IBT 34450. Infragen. class: subgen. *Aspergillus*, sect. *Aspergillus*, ser. *Rubri*. Reproduction: homothallic. ITS barcode: LT670986 (alternative markers: *BenA* = LT671206; *CaM* = LT671207; *RPB2* = LT671208).  

***Dendrosphaera***

***Dendrosphaera eberhardtii*** Pat., Bull. Soc. Mycol. France 23: 69. 1907. [MB183425]. — Type: Patouillard 1907, Bull. Soc. Mycol. France 23: Pl. VIII, Fig. 1. (– lectotype designated here, MBT392299). Ex-type: n.a. Reproduction: homothallic. ITS barcode: n.a. (alternative markers: *BenA* = n.a.; *CaM* = n.a.; *RPB2* = n.a.).  

***Dichlaena***

***Dichlaena indica*** A.B. Pawar *et al.*, Geobios New Rep. 4: 66. 1985. [MB127024]. — Type: HCIO 32780. Ex-type: n.a. Reproduction: homothallic. ITS barcode: n.a. (alternative markers: *BenA* = n.a.; *CaM* = n.a.; *RPB2* = n.a.).

***Dichlaena lentisci*** Durieu & Mont., Exploration scientifique de l'Algérie 1: 405. 1849. [MB249716]. — Type: von Höhnel (FH). Ex-type: n.a. Reproduction: homothallic. ITS barcode: n.a. (alternative markers: *BenA* = n.a.; *CaM* = n.a.; *RPB2* = n.a.).  

***Evansstolkia***

***Evansstolkia leycettana*** (H.C. Evans & Stolk) Houbraken *et al.*, this study. 2020. [MB832558]. Basionym: *Talaromyces leycettanus* H.C. Evans & Stolk, Trans. Brit. Mycol. Soc. 56: 45. 1971. [MB324419]. — Type: CBS 398.68. Ex-type: CBS 398.68 = ATCC 22469 = IMI 178525 = JCM 12814 = NRRL 5178. Reproduction: homothallic. ITS barcode: AF454080 (alternative markers: *BenA* = GU092791; *CaM* = GU092837; *RPB2* = EU021654).  

***Hamigera***

***Hamigera avellanea*** (Thom & Turesson) Stolk & Samson, Persoonia 6: 345. 1971. [MB314868]. Basionym: *Penicillium avellaneum* Thom & Turesson, Mycologia 7: 284. 1915. [MB248029]. — Type: CBS 295.48. Ex-type: CBS 295.48 = ATCC 10414 = CECT 2265 = DSM 2208 = IMI 040230 = NRRL 1938. Reproduction: homothallic. ITS barcode: AF454075 (alternative markers: *BenA* = EU021664; *CaM* = EU021682; *RPB2* = EU021627).

***Hamigera brevicompacta*** (H.Z. Kong) Samson *et al.*, this study. 2020. [MB832579]. Basionym: *Talaromyces brevicompactus* H.Z. Kong, Mycosystema 18: 9. 1999. [MB460109]. — Type: HMAS 62770. Ex-type: CBS 102661 = AS 3.4676. Reproduction: homothallic. ITS barcode: MN431402 (alternative markers: *BenA* = MN969421; *CaM* = MN969342; *RPB2* = MN969203).

***Hamigera fusca*** S.W. Peterson *et al.*, Mycologia 102: 857. 2010. [MB516020]. — Type: BPI 879307. Ex-type: DTO 194-D6 = CBS 132829 = NRRL 35601. Reproduction: homothallic. ITS barcode: GU092938 (alternative markers: *BenA* = GU092780; *CaM* = GU092813; *RPB2* = GU111755).

***Hamigera inflata*** S.W. Peterson *et al.*, Mycologia 102: 854. 2010. [MB516017]. — Type: BPI 879308. Ex-type: NRRL 58014. Reproduction: homothallic. ITS barcode: GU092949 (alternative markers: *BenA* = GU092793; *CaM* = GU092823; *RPB2* = GU092908).

***Hamigera ingelheimensis*** (J.F.H. Beyma) S.W. Peterson, Mycology 5: 105. 2014. [MB807715]. Basionym: *Penicillium ingelheimense* J.F.H. Beyma, Antonie van Leeuwenhoek 8: 109. 1942. [MB289090]. — Type: IMI 234977. Ex-type: CBS 163.42 = DTO 027-G9 = FRR 2110 = IMI 234977 = NRRL 2110. Reproduction: asexual. ITS barcode: MN431403 (alternative markers: *BenA* = GU092756; *CaM* = GU092829; *RPB2* = GU092912).

***Hamigera insecticola*** S.W. Peterson *et al.*, Mycologia 102: 852. 2010. [MB516016]. — Type: BPI 879309. Ex-type: DTO 194-D5 = CBS 132828 = NRRL 35386. Reproduction: homothallic. ITS barcode: EF634410 (alternative markers: *BenA* = GU092773; *CaM* = GU092816; *RPB2* = GU111754).

***Hamigera pallida*** S.W. Peterson *et al.*, Mycologia 102: 856. 2010. [MB516019]. — Type: BPI 879310. Ex-type: DTO 194-D7 = CBS 132830 = NRRL 35718. Reproduction: homothallic. ITS barcode: GU092950 (alternative markers: *BenA* = GU092786; *CaM* = GU092824; *RPB2* = GU111758).

***Hamigera paravellanea*** S.W. Peterson *et al.*, Mycologia 102: 852. 2010. [MB516015]. — Type: BPI 879311. Ex-type: DTO 194-D8 = CBS 132831 = NRRL 35720. Reproduction: homothallic. ITS barcode: GU092952 (alternative markers: *BenA* = GU092788; *CaM* = GU092826; *RPB2* = GU092919).

***Hamigera terricola*** S.W. Peterson *et al.*, Mycologia 102: 855. 2010. [MB516018]. — Type: BPI 879312. Ex-type: DTO 194-D4 = CBS 132827 = NRRL 29055. Reproduction: homothallic. ITS barcode: GU092946 (alternative markers: *BenA* = GU092759; *CaM* = GU092811; *RPB2* = GU111751).  

***Leiothecium***

***Leiothecium cristatum*** Y. Marín *et al.*, Int. J. Syst. Evol. Microbiol. 64: 2873. 2014. [MB803513]. — Type: CBS-H 21130. Ex-type: FMR 11998 = CBS 134260 = NBRC 109843. Reproduction: homothallic. ITS barcode: KF732838 (alternative markers: *BenA* = n.a.; *CaM* = n.a.; *RPB2* = HF954976).

***Leiothecium ellipsoideum*** Samson & Mouch., Canad. J. Bot. 53: 1634. 1975. [MB316445]. — Type: CBS 607.74. Ex-type: CBS 607.74 = ATCC 32453. Reproduction: homothallic. ITS barcode: KF732839 (alternative markers: *BenA* = KY709178; *CaM* = KY611939; *RPB2* = JN121541).  

***Monascus***

***Monascus argentinensis*** Stchigel & Guarro, Stud. Mycol. 50: 301. 2004. [MB500076]. — Type: FMR 6778. Ex-type: CBS 109402 = FMR 6778. Infragen. class: sect. *Floridani*. Reproduction: homothallic. ITS barcode: JF922046 (alternative markers: *BenA* = KY709174; *CaM* = KY611935; *RPB2* = JN121423).

***Monascus flavipigmentosus*** R.N. Barbosa *et al.*, Stud. Mycol. 86: 43. 2017. [MB820072]. — Type: URM 90064. Ex-type: URM 7536 = CBS 142366 = DTO 353-A2. Infragen. class: sect. *Floridani*. Reproduction: homothallic. ITS barcode: KY511751 (alternative markers: *BenA* = KY709168; *CaM* = KY611929; *RPB2* = MN969201).

***Monascus floridanus*** P.F. Cannon & E.L. Barnard, Mycologia 79: 480. 1987. [MB132123]. — Type: IMI 282587. Ex-type: FLAS F54662 = CBS 142228 = CGMCC 3.5843 = BCRC 33310 = UAMH 4180. Infragen. class: sect. *Floridani*. Reproduction: homothallic. ITS barcode: KY635848 (alternative markers: *BenA* = KY709172; *CaM* = KY611933; *RPB2* = KY611972).

***Monascus lunisporas*** Udagawa & H. Baba, Cryptog. Mycol. 19: 270. 1998. [MB446999]. — Type: SUM 3116. Ex-type: CBS 142230 = CGMCC 3.7951 = ATCC 204397 = NBRC 33241 = BCRC 33640. Infragen. class: sect. *Floridani*. Reproduction: homothallic. ITS barcode: KY635847 (alternative markers: *BenA* = KY709171; *CaM* = KY611932; *RPB2* = KY611971).

***Monascus mellicola*** R.N. Barbosa *et al.*, Stud. Mycol. 86: 44. 2017. [MB820073]. — Type: URM 90065. Ex-type: URM 7510 = CBS 142364 = DTO 350-E6. Infragen. class: sect. *Floridani*. Reproduction: asexual. ITS barcode: KY511726 (alternative markers: *BenA* = KY709143; *CaM* = KY611904; *RPB2* = KY611943).

***Monascus pallens*** P.F. Cannon *et al.*, Mycol. Res. 99: 659. 1995. [MB413476]. — Type: IMI 356820. Ex-type: BSRA 10266 = CBS 142229 = CGMCC 3.5844 = ATCC 200612 = BCRC 33641. Infragen. class: sect. *Floridani*. Reproduction: homothallic. ITS barcode: KY635849 (alternative markers: *BenA* = KY709173; *CaM* = KY611934; *RPB2* = KY611973).

***Monascus purpureus*** Went, Ann. Sci. Nat., Bot., Sér. 8, 1: 1. 1895. [MB235390]. — Type: IMI 210765. Ex-type: CBS 109.07 = IF0 45 13 = ATCC 16426 = NRRL 1596 = FRR 1596. Infragen. class: sect. *Rubri*. Reproduction: homothallic. ITS barcode: KY635851 (alternative markers: *BenA* = KY709176; *CaM* = KY611937; *RPB2* = JN121422).

***Monascus recifensis*** R.N. Barbosa *et al.*, Stud. Mycol. 86: 47. 2017. [MB820074]. — Type: URM 90066. Ex-type: URM 7524 = CBS 142365 = DTO 350-G6. Infragen. class: sect. *Floridani*. Reproduction: asexual. ITS barcode: KY511740 (alternative markers: *BenA* = KY709157; *CaM* = KY611918; *RPB2* = KY611957).

***Monascus ruber*** Tiegh., Bull. Soc. Mycol. France 31: 227. 1884. [MB234876]. — Type: IMI 81596. Ex-type: CBS 135.60 = IFO 8451 = ATCC 15670. Infragen. class: sect. *Rubri*. Reproduction: homothallic. ITS barcode: KY635850 (alternative markers: *BenA* = KY709175; *CaM* = KY611936; *RPB2* = KY611974).  

***Paecilomyces***

***Paecilomyces brunneolus*** (N. Inagaki) Samson & Houbraken, Persoonia 22: 21. 2009. [MB512559]. Basionym: *Paecilomyces variotii* var. *brunneolus* N. Inagaki, Trans. Mycol. Soc. Japan 4: 3. 1962. [MB353669]. — Type: unknown. Ex-type: CBS 370.70 = DTO 093-D7 = IFO 7563. Reproduction: asexual. ITS barcode: EU037050 (alternative markers: *BenA* = EU037068; *CaM* = EU037033; *RPB2* = MN969152).

***Paecilomyces dactylethromorphus*** Bat. & H. Maia, Anais Soc. Biol. Pernambuco 15: 152. 1957. [MB302183]. — Type: IMUR 235. Ex-type: DTO 280-D1 = CBS 251.55 = ATCC 11971 = IMI 065752 = MUCL 9649. Reproduction: asexual. ITS barcode: FJ389951 (alternative markers: *BenA* = FJ390002; *CaM* = FJ389960; *RPB2* = n.a.).

***Paecilomyces divaricatus*** (Thom) Samson *et al.*, Persoonia 22: 21. 2009. [MB512561]. Basionym: *Penicillium divaricatum* Thom, U.S.D.A. Bur. Ani. Ind. Bull. 118: 72. 1910. [MB170004]. — Type: Thom 1910, U.S.D.A. Bur. Animal Industr. Bull. 118: p. 73, Fig. 29. (– lectotype designated here, MBT392294; CBS 284.48 [metabolically inactive] – epitype designated here, MBT392295). Ex-epitype: DTO 093-D8 = CBS 284.48 = ATCC 10121 = ATCC 18502 = DSM 1961 = IAM 5001 = IMI 040025 = NBRC 100534 = NRRL 1115 = QM 6764 = VTT D-83214 = Thom 34. Reproduction: asexual. ITS barcode: FJ389931 (alternative markers: *BenA* = FJ389992; *CaM* = FJ389953; *RPB2* = n.a.).

***Paecilomyces formosus*** Sakag. *et al.* ex Houbraken & Samson, Persoonia 22: 21. 2009, *nom*. *inval*. [MB512562]. Basionym: *Monilia formosa* Sakag. *et al.*, Zentralbl. Bakteriol., Abt. 2 100: 302. 1939 (*nom*. *inval*.) [MB252219]. — Type: CBS 990.73B. Ex-type: DTO 093-D2 = CBS 990.73B = ATCC 10865 = IMI 058427 = LSHB Pa31 = LSHB X26 = NRRL 1282. Reproduction: protoheterothallic (Heidarian *et al.* 2018). ITS barcode: FJ389929 (alternative markers: *BenA* = FJ389993; *CaM* = FJ389978; *RPB2* = MN969154).

***Paecilomyces fulvus*** Stolk & Samson, Persoonia 6: 354. 1971. [MB319107]. — Type: CBS 132.33. Ex-type: CBS 132.33 = 1MI 58.421. Reproduction: homothallic. ITS barcode: FJ389939 (alternative markers: *BenA* = FJ389988; *CaM* = FJ389957; *RPB2* = n.a.).

***Paecilomyces lagunculariae*** (C. Ram) Houbraken *et al.*, this study. 2020. [MB832559]. Basionym: *Byssochlamys nivea* var. *lagunculariae* C. Ram, Nova Hedwigia 16: 311. 1968. [MB349108]. — Type: IMUFPe 2195. Ex-type: CBS 373.70. Reproduction: homothallic. ITS barcode: FJ389944 (alternative markers: *BenA* = FJ389995; *CaM* = FJ389965; *RPB2* = MN969204).

***Paecilomyces niveus*** Stolk & Samson, Persoonia 6: 351. 1971. [MB319117]. — Type: CBS 100.11. Ex-type: CBS 100.11 = ATCC 22260. Reproduction: homothallic. ITS barcode: FJ389934 (alternative markers: *BenA* = FJ389999; *CaM* = FJ389956; *RPB2* = JF417414).

***Paecilomyces tabacinus*** Jurjević *et al.*, Persoonia 36: 409. 2016. [MB816870]. — Type: BPI 910044. Ex-type: CBS 141098 = DTO 412-B7 = CCF 5290. Reproduction: asexual. ITS barcode: LT548280 (alternative markers: *BenA* = MN969434; *CaM* = LT548288; *RPB2* = MN969210).

***Paecilomyces variotii*** Bainier, Bull. Soc. Mycol. France 23: 27. 1907. [MB248517]. — Type: unknown. Ex-type: DTO 032-I8 = DTO 280-D5 = CBS 102.74 = CECT 2803 = NRRL 1116. Reproduction: Heterothallic (Houbraken *et al.* 2008). ITS barcode: EU037055 (alternative markers: *BenA* = EU037073; *CaM* = EU037038; *RPB2* = MN969153).

***Paecilomyces zollerniae*** Stolk & Samson, Persoonia 6: 356. 1971. [MB319129]. — Type: CBS 374.70. Ex-type: CBS 374.70 = JCM 12808. Reproduction: homothallic. ITS barcode: FJ389933 (alternative markers: *BenA* = FJ390008; *CaM* = FJ389966; *RPB2* = n.a.).  

***Penicillago***

***Penicillago kabunica*** (Baghd.) Houbraken *et al.*, this study. 2020. [MB832560]. Basionym: *Penicillium kabunicum* Baghd., Novosti Sist. Nizsh. Rast. 5: 98. 1968. [MB335738]. — Type: CBS 409.68 (neotype). Ex-type: CBS 575.90 = CBS 409.69 (dead) = DTO 105-H9 = FRR 513 = IMI 140341 = VKM F-1072. Reproduction: asexual. ITS barcode: MN431415 (alternative markers: *BenA* = MN969438; *CaM* = MN969357; *RPB2* = MN969217).

***Penicillago mirabilis*** (Beliakova & Milko) Houbraken *et al.*, this study. 2020. [MB832561]. Basionym: *Penicillium mirabile* Beliakova & Milko, Mikol. Fitopatol. 6: 145. 1972. [MB319286]. — Type: BKM F-1328. Ex-type: CBS 624.72 = DTO 304-C2 = CCRC 31665 = FRR 1959 = IMI 167383 = LCP 72.2193 = MUCL 31206 = VKM F-1328. Reproduction: asexual. ITS barcode: MN431416 (alternative markers: *BenA* = MN969439; *CaM* = MN969358; *RPB2* = MN969218).

***Penicillago moldavica*** (Milko & Beliakova) Houbraken *et al.*, this study. 2020. [MB832562]. Basionym: *Penicillium moldavicum* Milko & Beliakova, Novosti Sist. Nizsh. Rast. 4: 255. 1967. [MB335751]. — Type: IMI 129966. Ex-type: CBS 574.90 = CBS 627.67 (dead) = DTO 041-H9 = ATCC 18355 = FRR 665 = IMI 129966 = VKM F-922. Reproduction: asexual. ITS barcode: MN431417 (alternative markers: *BenA* = MN969440; *CaM* = MN969359; *RPB2* = MN969219).

***Penicillago nodositata*** (Valla) Guevara-Suarez *et al.*, Fungal Syst. Evol. 5: 64. 2020. [MB822074]. Basionym: *Penicillium nodositatum* Valla, Plant and Soil 114: 146. 1989. [MB126535]. — Type: Figs 1–4 in Valla *et al.* (1989) (lectotype, designated in Guevara-Suarez *et al.* 2020, MBT388228). Ex-epitype: CBS 333.90 = DTO 252-C7. Reproduction: asexual. ITS barcode: KC790403 (alternative markers: *BenA* = KC790399; *CaM* = MN969361; *RPB2* = MN969220).  

***Penicilliopsis***

***Penicilliopsis africana*** Samson & Seifert, Adv. Pen. Asp. Syst.: 408. 1986 [1985]. [MB114759]. — Type: Metiquette Louis 6275 (BR). Ex-type: n.a. Reproduction: homothallic. ITS barcode: n.a. (alternative markers: *BenA* = n.a.; *CaM* = n.a.; *RPB2* = n.a.).

***Penicilliopsis clavariiformis*** Solms, Ann. Jard. Bot. Buitenzorg 6: 53. 1886. [MB120178]. — Type: Bot. Garden Bogor, Solms-Laubach in herb. Hauman (BR). Ex-type: n.a. Reproduction: homothallic. ITS barcode: MN431401 (alternative markers: *BenA* = MN969420; *CaM* = n.a.; *RPB2* = EF669667).

***Penicilliopsis pseudocordyceps*** H.M. Hsieh & Y.M. Ju, Mycologia 9: 541. 2002. [MB484663]. — Type: HAST (Taiwan) Hsieh & Ju 89112611. Ex-type: BCRC 33730. Reproduction: homothallic. ITS barcode: n.a. (alternative markers: *BenA* = n.a.; *CaM* = n.a.; *RPB2* = n.a.).

***Penicilliopsis zonata*** (Kwon-Chung & Fennell) Samson *et al.*, Stud. Mycol. 85: 211. 2016. [MB819185]. Basionym: *Aspergillus zonatus* Kwon-Chung & Fennell, Gen. Aspergillus: 377. 1965. [MB326666]. — Type: WB 5079. Ex-type: DTO 022-B4 = CBS 506.65 = NRRL 5079 = ATCC 16867 = IFO 8817 = IMI 124936 = LCP 89.2588 = WB 5079. Reproduction: asexual. ITS barcode: EF669712 (alternative markers: *BenA* = EF669679; *CaM* = EF669701; *RPB2* = EF669665).  

***Penicillium***

***Penicillium abidjanum*** Stolk, Antonie van Leeuwenhoek 34: 49. 1968. [MB335705]. — Type: CBS 246.67. Ex-type: CBS 246.67 = DTO 101-B4 = ATCC 18385 = FRR 1156 = IMI 136244. Infragen. class: subgen. *Aspergilloides*, sect. *Lanata-Divaricata*, ser. *Dalearum*. Reproduction: homothallic. ITS barcode: GU981582 (alternative markers: *BenA* = GU981650; *CaM* = MN969234; *RPB2* = JN121469).

***Penicillium acidum*** Hyang B. Lee *et al.*, Fungal Diversity 89: 173. 2018. [MB822167]. — Type: CNUFC-DLW4-1. Ex-type: JMRC SF:013659 = CNUFC-DLW4-1. Infragen. class: subgen. *Aspergilloides*, sect. *Sclerotiorum*, ser. *Sclerotiorum*. Reproduction: asexual. ITS barcode: KY587441 (alternative markers: *BenA* = KY587439; *CaM* = KY587442; *RPB2* = KY587446).

***Penicillium adametzii*** K.W. Zaleski, Bull. Int. Acad. Polon. Sci., Sér. B., Sci. Nat., 1927: 507. 1927. [MB119777]. — Type: IMI 39751. Ex-type: CBS 209.28 = ATCC 10407 = IMI 039751 = MUCL 29106 = NRRL 737. Infragen. class: subgen. *Aspergilloides*, sect. *Sclerotiorum*, ser. *Adametziorum*. Reproduction: asexual. ITS barcode: JN714929 (alternative markers: *BenA* = JN625957; *CaM* = KC773796; *RPB2* = JN121455).

***Penicillium adametzioides*** S. Abe ex G. Sm., Trans. Brit. Mycol. Soc. 46: 335. 1963. [MB302372]. — Type: IMI 068227. Ex-type: CBS 313.59 = ATCC 18306 = FAT1302 = IFO 6055 = IMI 068227 = NRRL 3405 = QM 7312. Infragen. class: subgen. *Aspergilloides*, sect. *Sclerotiorum*, ser. *Adametziorum*. Reproduction: asexual. ITS barcode: JN686433 (alternative markers: *BenA* = JN799642; *CaM* = JN686387; *RPB2* = JN406578).

***Penicillium aeris*** Visagie & Samson, Persoonia 36: 139. 2016. [MB808262]. — Type: CBS H-21608. Ex-type: CBS 135897 = DTO 207-D4. Infragen. class: subgen. *Aspergilloides*, sect. *Torulomyces*, ser. *Torulomyces*. Reproduction: asexual. ITS barcode: KF303654 (alternative markers: *BenA* = KF303614; *CaM* = KF303627; *RPB2* = KF303681).

***Penicillium alagoense*** L.O. Ferro *et al.*, Persoonia 42: 447. 2019. [MB830760]. — Type: URM 93058. Ex-type: URM 8086. Infragen. class: subgen. *Aspergilloides*, sect. *Lanata-Divaricata*, ser. *Simplicissima*. Reproduction: asexual. ITS barcode: MK804503 (alternative markers: *BenA* = MK802333; *CaM* = MK802336; *RPB2* = MK802338).

***Penicillium albocoremium*** (Frisvad) Frisvad, Int. Mod. Tax. Meth. Pen. Asp. Clas.: 275. 2000. [MB459817]. Basionym: *Penicillium hirsutum* var. *albocoremium* Frisvad, Mycologia 81: 856. 1990. [MB126411]. — Type: IMI 285511. Ex-type: CBS 472.84 = FRR 2931 = IBT 10682 = IBT 21502 = IMI 285511. Infragen. class: subgen. *Penicillium*, sect. *Fasciculata*, ser. *Corymbifera*. Reproduction: asexual. ITS barcode: AJ004819 (alternative markers: *BenA* = KU896812; *CaM* = KU896819; *RPB2* = KU904344).

***Penicillium alexiae*** Visagie *et al.*, Persoonia 31: 59. 2013. [MB803785]. — Type: CBS H-21142. Ex-type: CBS 134558. Infragen. class: subgen. *Aspergilloides*, sect. *Sclerotiorum*, ser. *Adametziorum*. Reproduction: asexual. ITS barcode: KC790400 (alternative markers: *BenA* = KC773778; *CaM* = KC773803; *RPB2* = KX961291).

***Penicillium alfredii*** Visagie *et al.*, Stud. Mycol. 78: 116. 2014. [MB809180]. — Type: CBS H-21800. Ex-type: CBS 138224 = DTO 269-A4. Infragen. class: subgen. *Aspergilloides*, sect. *Alfrediorum*, ser. *Alfrediorum*. Reproduction: asexual. ITS barcode: KJ775684 (alternative markers: *BenA* = KJ775177; *CaM* = KJ775411; *RPB2* = KJ834520).

***Penicillium allii*** Vincent & Pitt, Mycologia 81: 300. 1989. [MB125498]. — Type: MU Vincent 114. Ex-type: CBS 131.89 = IMI 321505 = NRRL 13630 = ATCC 64636 = IMI 321506 = IBT 6610. Infragen. class: subgen. *Penicillium*, sect. *Fasciculata*, ser. *Corymbifera*. Reproduction: asexual. ITS barcode: AJ005484 (alternative markers: *BenA* = AY674331; *CaM* = KU896820; *RPB2* = KU904345).

***Penicillium allii-sativi*** Frisvad *et al.*, Persoonia 29: 89. 2012. [MB801873]. — Type: CBS H-21058. Ex-type: DTO 149-A8 = CBS 132074 = IBT 26507 = LJC 206. Infragen. class: subgen. *Penicillium*, sect. *Chrysogena*, ser. *Chrysogena*. Reproduction: asexual. ITS barcode: JX997021 (alternative markers: *BenA* = JX996891; *CaM* = JX996232; *RPB2* = JX996627).

***Penicillium alogum*** Visagie *et al.*, Persoonia 36: 263. 2016. [MB815772]. — Type: DAOM 695759. Ex-type: DAOMC 250543 = CBS 140996 = DTO 410-E1 = IBT 23947= KAS 2475. Infragen. class: subgen. *Aspergilloides*, sect. *Stolkia*, ser. *Stolkia*. Reproduction: asexual. ITS barcode: KT887869 (alternative markers: *BenA* = KT887830; *CaM* = KT887791; *RPB2* = MN969172).

***Penicillium alutaceum*** D.B. Scott, Mycopathol. Mycol. Appl. 36: 17. 1968. [MB335708]. — Type: CBS 317.67. Ex-type: CBS 317.67 = ATCC 18542 = FRR 1158 = IFO 31728 = IMI 136243. Infragen. class: subgen. *Aspergilloides*, sect. *Exilicaulis*, ser. *Alutacea*. Reproduction: homothallic. ITS barcode: AF033454 (alternative markers: *BenA* = KJ834430; *CaM* = KP016768; *RPB2* = JN121489).

***Penicillium amaliae*** Visagie *et al.*, Persoonia 31: 52. 2013. [MB803784]. — Type: CBS H-21141. Ex-type: CBS 134209 = CV 1875 = DTO 183F3 = DAOM 241034. Infragen. class: subgen. *Aspergilloides*, sect. *Sclerotiorum*, ser. *Adametziorum*. Reproduction: asexual. ITS barcode: JX091443 (alternative markers: *BenA* = JX091563; *CaM* = JX141557; *RPB2* = KX961292).

***Penicillium americanum*** Jurjević *et al.*, Persoonia 42: 443. 2019. [MB830667]. — Type: BPI 910642. Ex-type: NRRL 66819 = EMSL1473 = ITEM 17520. Infragen. class: subgen. *Penicillium*, sect. *Ramosum*, ser. *Soppiorum*. Reproduction: asexual. ITS barcode: MK791278 (alternative markers: *BenA* = MK803427; *CaM* = MK803428; *RPB2* = n.a.).

***Penicillium amphipolaria*** Visagie *et al.*, Persoonia 36: 269. 2016. [MB815777]. — Type: DAOM 695760. Ex-type: DAOMC 250551 = CBS 140997 = DTO 410-E2 = W 284 = KAS 2555. Infragen. class: subgen. *Aspergilloides*, sect. *Lanata-Divaricata*, ser. *Dalearum*. Reproduction: asexual. ITS barcode: KT887872 (alternative markers: *BenA* = KT887833; *CaM* = KT887794; *RPB2* = MN969177).

***Penicillium anatolicum*** Stolk, Antonie van Leenwenhoek 34: 46. 1968. [MB335710]. — Type: CBS 479.66. Ex-type: CBS 479.66 = IBT 30764. Infragen. class: subgen. *Aspergilloides*, sect. *Citrina*, ser. *Euglauca*. Reproduction: homothallic. ITS barcode: AF033425 (alternative markers: *BenA* = JN606849; *CaM* = JN606571; *RPB2* = JN606593).

***Penicillium angulare*** S.W. Peterson *et al.*, Mycologia 96: 1289. 2004. [MB487891]. — Type: BPI 842268. Ex-type: CBS 130293 = IBT 27051 = NRRL 28157. Infragen. class: subgen. *Aspergilloides*, sect. *Sclerotiorum*, ser. *Adametziorum*. Reproduction: asexual. ITS barcode: AF125937 (alternative markers: *BenA* = KC773779; *CaM* = KC773804; *RPB2* = JN406554).

***Penicillium angustiporcatum*** Takada & Udagawa, Trans. Mycol. Soc. Japan 24: 143. 1983. [MB108322]. — Type: NHL 6481. Ex-type: CBS 202.84. Infragen. class: subgen. *Aspergilloides*, sect. *Gracilenta*, ser. *Angustiporcata*. Reproduction: homothallic. ITS barcode: KC411690 (alternative markers: *BenA* = KJ834431; *CaM* = MN969235; *RPB2* = JN406617).

***Penicillium annulatum*** Visagie & K. Jacobs, Mycol. Prog. 14 (no. 96): 14. 2015. [MB809817]. — Type: CBS H-21333. Ex-type: CBS 135126 = CV 0037 = DTO 180-G7. Infragen. class: subgen. *Aspergilloides*, sect. *Lanata-Divaricata*, ser. *Rolfsiorum*. Reproduction: asexual. ITS barcode: JX091426 (alternative markers: *BenA* = JX091514; *CaM* = JX141545; *RPB2* = KF296410).

***Penicillium antarcticum*** A.D. Hocking & C.F. McRae, Polar Biol. 21: 103. 1999. [MB482749]. — Type: DAR 72813. Ex-type: CBS 100492 = FRR 4989 = DTO 187-B3. Infragen. class: subgen. *Penicillium*, sect. *Canescentia*, ser. *Atroveneta*. Reproduction: asexual. ITS barcode: KJ834503 (alternative markers: *BenA* = MN969371; *CaM* = MN969236; *RPB2* = JN406653).

***Penicillium aotearoae*** Visagie & Seifert, Persoonia 36: 265. 2016. [MB815774]. — Type: PDD 107543. Ex-type: DAOMC 250538 = CBS 140999 = DTO 410-E4 = KAS 3088. Infragen. class: subgen. *Aspergilloides*, sect. *Exilicaulis*, ser. *Lapidosa*. Reproduction: asexual. ITS barcode: KT887874 (alternative markers: *BenA* = KT887835; *CaM* = KT887796; *RPB2* = MN969174).

***Penicillium apimei*** R.N. Barbosa *et al.*, Antonie van Leeuwenhoek 111: 1891. 2018. [MB822208]. — Type: URM 90489. Ex-type: CBS 142502 = URM 7591. Infragen. class: subgen. *Aspergilloides*, sect. *Gracilenta*, ser. *Macrosclerotiorum*. Reproduction: asexual. ITS barcode: MF278310 (alternative markers: *BenA* = LT854641; *CaM* = LT882717; *RPB2* = LT854650).

***Penicillium aquaticum*** Hyang B. Lee *et al.*, Fungal Diversity 89: 173. 2018. [MB822166]. — Type: CNUFC-YSW8-1. Ex-type: JMRC SF:013660. Infragen. class: subgen. *Aspergilloides*, sect. *Gracilenta*, ser. *Macrosclerotiorum*. Reproduction: asexual. ITS barcode: KY587453 (alternative markers: *BenA* = KY587450; *CaM* = KY587447; *RPB2* = KY587449).

***Penicillium arabicum*** Baghd., Novosti Sist. Nizsh. Rast. 5: 105. 1968. [MB335711]. — Type: T16 in Universitate Mosquensi (holotype); CBS H-7471 (isotype). Ex-type: CBS 414.69 = ATCC 22347 = DSM 2205 = FRR 507 = IMI 140335 = VKMF-1077. Infragen. class: subgen. *Aspergilloides*, sect. *Exilicaulis*, ser. *Restricta*. Reproduction: asexual. ITS barcode: KC411758 (alternative markers: *BenA* = KP016750; *CaM* = KP016770; *RPB2* = KP064574).

***Penicillium araracuaraense*** Houbraken, *et al.*, Int. J. Syst. Evol. Microbiol. 61: 1469. 2011. [MB518025]. — Type: HUA 170334. Ex-type: CBS 113149 = DTO 056-D5 = DTO 297-H3 = IBT 23247. Infragen. class: subgen. *Aspergilloides*, sect. *Lanata-Divaricata*, ser. *Simplicissima*. Reproduction: asexual. ITS barcode: GU981597 (alternative markers: *BenA* = GU981642; *CaM* = MN969237; *RPB2* = KF296414).

***Penicillium ardesiacum*** Novobr., Novosti Sist. Nizsh. Rast. 11: 228. 1974. [MB319257]. — Type: IMI 174719. Ex-type: CBS 497.73 = ATCC 24719 = FRR 1479 = IFO 30540 = IMI 174719 = VKMF-1749. Infragen. class: subgen. *Aspergilloides*, sect. *Aspergilloides*, ser. *Pinetorum*. Reproduction: asexual. ITS barcode: KM189565 (alternative markers: *BenA* = KM088805; *CaM* = KM089190; *RPB2* = KM089577).

***Penicillium argentinense*** Houbraken *et al.*, Stud. Mycol. 70: 78. 2011. [MB563185]. — Type: CBS H-20461. Ex-type: CBS 130371 = IBT 30761. Infragen. class: subgen. *Aspergilloides*, sect. *Citrina*, ser. *Euglauca*. Reproduction: homothallic. ITS barcode: JN831361 (alternative markers: *BenA* = JN606815; *CaM* = JN606549; *RPB2* = MN969105).

***Penicillium arianeae*** Visagie *et al.*, Persoonia 31: 59. 2013. [MB803786]. — Type: CBS H-21143. Ex-type: CBS 134559. Infragen. class: subgen. *Aspergilloides*, sect. *Sclerotiorum*, ser. *Adametziorum*. Reproduction: asexual. ITS barcode: KC773833 (alternative markers: *BenA* = KC773784; *CaM* = KC773811; *RPB2* = KX961294).

***Penicillium arizonense*** Frisvad *et al.*, Sci. Rep. 6: 35112, 8. 2016. [MB817128]. — Type: C-F-101845. Ex-type: IBT 12289 = CBS 141311 = DTO 193-G8. Infragen. class: subgen. *Penicillium*, sect. *Canescentia*, ser. *Canescentia*. Reproduction: asexual. ITS barcode: MH492021 (alternative markers: *BenA* = MH492019; *CaM* = MH492020; *RPB2* = MH492022).

***Penicillium armarii*** Houbraken *et al.*, Stud. Mycol. 78: 410. 2014. [MB809955]. — Type: CBS H-21870. Ex-type: CBS 138171 = DTO 235-F1. Infragen. class: subgen. *Aspergilloides*, sect. *Aspergilloides*, ser. *Glabra*. Reproduction: asexual. ITS barcode: KM189758 (alternative markers: *BenA* = KM089007; *CaM* = KM089394; *RPB2* = KM089781).

***Penicillium astrolabium*** R. Serra & S.W. Peterson, Mycologia 99: 80. 2007. [MB504766]. — Type: BPI 872160. Ex-type: CBS 122427 = NRRL 35611 = MUM 06.161. Infragen. class: subgen. *Penicillium*, sect. *Brevicompacta*, ser. *Olsoniorum*. Reproduction: asexual. ITS barcode: DQ645804 (alternative markers: *BenA* = DQ645793; *CaM* = DQ645808; *RPB2* = JN406634).

***Penicillium asymmetricum*** (Subram. & Sudha) Houbraken & Samson, Stud. Mycol. 70: 47. 2011. [MB561963]. Basionym: *Thysanophora asymmetrica* Subram. & Sudha, Kavaka 13: 88. 1987. [MB135502]. — Type: unknown. Ex-type: n.a. Infragen. class: subgen. *Aspergilloides*, sect. *Thysanophora*, ser. *Thysanophora*. Reproduction: asexual. ITS barcode: n.a. (alternative markers: *BenA* = n.a.; *CaM* = n.a.; *RPB2* = n.a.).

***Penicillium athertonense*** Houbraken, Stud. Mycol. 78: 412. 2014. [MB809956]. — Type: CBS H-21874. Ex-type: CBS 138161 = DTO 030-C2. Infragen. class: subgen. *Aspergilloides*, sect. *Aspergilloides*, ser. *Pinetorum*. Reproduction: asexual. ITS barcode: KM189462 (alternative markers: *BenA* = KM088690; *CaM* = KM089075; *RPB2* = KM089462).

***Penicillium atramentosum*** Thom, U.S.D.A. Bur. Animal Industr. Bull. 118: 65. 1910. [MB237291]. — Type: IMI 39752. Ex-type: CBS 291.48 = ATCC 10104 = FRR 795 = IBT 6616 = IFO 8137 = IMI 039752 = IMI 039752ii = LSHBP 1 = MUCL 29071 = MUCL 29126 = NRRL 795 = QM 7483. Infragen. class: subgen. *Penicillium*, sect. *Paradoxa*, ser. *Atramentosa*. Reproduction: asexual. ITS barcode: AF033483 (alternative markers: *BenA* = AY674402; *CaM* = KU896821; *RPB2* = JN406584).

***Penicillium atrofulvum*** Houbraken *et al.*, Stud. Mycol. 70: 80. 2011. [MB563183]. — Type: CBS H-20650. Ex-type: CBS 109.66 = DTO 031-B2 = FRR 799 = IBT 30032 = IBT 29667. Infragen. class: subgen. *Aspergilloides*, sect. *Citrina*, ser. *Westlingiorum*. Reproduction: asexual. ITS barcode: JN617663 (alternative markers: *BenA* = JN606677; *CaM* = JN606387; *RPB2* = JN606620).

***Penicillium atrolazulinum*** Visagie & K. Jacobs, IMA Fungus 7: 91. 2016. [MB811001]. — Type: CBS H-22043. Ex-type: CBS 139136 = DAOMC 241083 = DTO 180-H4 = CV 55. Infragen. class: subgen. *Aspergilloides*, sect. *Exilicaulis*, ser. *Corylophila*. Reproduction: asexual. ITS barcode: JX140913 (alternative markers: *BenA* = JX141077; *CaM* = JX157416; *RPB2* = KP064575).

***Penicillium atrosanguineum*** B.X. Dong, Ceská Mycol. 27: 174. 1973. [MB319260]. — Type: PRC 1397 (holotype); CBS H-15524 (isotype). Ex-type: CBS 380.75 = FRR 1726 = IMI 197488. Infragen. class: subgen. *Aspergilloides*, sect. *Exilicaulis*, ser. *Lapidosa*. Reproduction: asexual. ITS barcode: JN617706 (alternative markers: *BenA* = KJ834435; *CaM* = KP016771; *RPB2* = JN406557).

***Penicillium atrovenetum*** G. Sm., Trans. Brit. Mycol. Soc. 39: 112. 1956. [MB302377]. — Type: IMI 061837. Ex-type: CBS 241.56 = ATCC 13352 = FRR 2571 = IFO 8138 = IMI 061837 = LSHBSm683 = QM 6963. Infragen. class: subgen. *Penicillium*, sect. *Canescentia*, ser. *Atroveneta*. Reproduction: asexual. ITS barcode: AF033492 (alternative markers: *BenA* = JX140944; *CaM* = KJ867004; *RPB2* = JN121467).

***Penicillium aurantiacobrunneum*** Houbraken *et al.*, Stud. Mycol. 70: 80. 2011. [MB563206]. — Type: CBS H-20662. Ex-type: CBS 126228 = DTO 078-G2 = IBT 18753. Infragen. class: subgen. *Aspergilloides*, sect. *Citrina*, ser. *Westlingiorum*. Reproduction: asexual. ITS barcode: JN617670 (alternative markers: *BenA* = JN606702; *CaM* = MN969238; *RPB2* = MN969106).

***Penicillium aurantiogriseum*** Dierckx, Ann. Soc. Sci. Bruxelles 25: 88. 1901. [MB247956]. — Type: IMI 195050. Ex-type: CBS 324.89 = ATCC 48920 = FRR 971 = IBT 14016 = IMI 195050 = MUCL 29090 = NRRL 971. Infragen. class: subgen. *Penicillium*, sect. *Fasciculata*, ser. *Viridicata*. Reproduction: asexual. ITS barcode: AF033476 (alternative markers: *BenA* = MN969372; *CaM* = KU896822; *RPB2* = JN406573).

***Penicillium aurantioviolaceum*** Biourge, Cellule 33: 282. 1923. [MB257885]. — Type: CBS H-21954. Ex-type: CBS 137777 = NRRL 762 = ATCC 14974. Infragen. class: subgen. *Aspergilloides*, sect. *Aspergilloides*, ser. *Thomiorum*. Reproduction: asexual. ITS barcode: KM189756 (alternative markers: *BenA* = KM089005; *CaM* = KM089392; *RPB2* = KM089779).

***Penicillium austricola*** Visagie & K. Jacobs, Persoonia 36: 139. 2016. [MB805184]. — Type: CBS H-21605. Ex-type: CBS 135900 = CV 1842 = DTO 183-E6 = DAOMC 241066. Infragen. class: subgen. *Aspergilloides*, sect. *Torulomyces*, ser. *Torulomyces*. Reproduction: asexual. ITS barcode: JX091466 (alternative markers: *BenA* = JX091579; *CaM* = JX141600; *RPB2* = KF303705).

***Penicillium austroafricanum*** Houbraken & Visagie, Stud. Mycol. 78: 412. 2014. [MB809957]. — Type: CBS H-21864. Ex-type: CBS 137773 = DTO 133-G5. Infragen. class: subgen. *Aspergilloides*, sect. *Aspergilloides*, ser. *Thomiorum*. Reproduction: asexual. ITS barcode: KM189610 (alternative markers: *BenA* = KM088854; *CaM* = KM089241; *RPB2* = KM089628).

***Penicillium austrosinense*** L. Cai *et al.*, Cladistics 35: 525. 2018 [2019]. [MB818164]. — Type: HMAS 247725. Ex-type: CGMCC 3.18797 = NN072318. Infragen. class: subgen. *Aspergilloides*, sect. *Lanata-Divaricata*, ser. *Dalearum*. Reproduction: asexual. ITS barcode: KY495007 (alternative markers: *BenA* = KY495116; *CaM* = MN969328; *RPB2* = KY495061).

***Penicillium austrosinicum*** X.C. Wang & W.Y. Zhuang, Sci. Rep. 7: 8233, 3. 2017. [MB570338]. — Type: HMAS 248734. Ex-type: CGMCC 3.18410. Infragen. class: subgen. *Aspergilloides*, sect. *Sclerotiorum*, ser. *Sclerotiorum*. Reproduction: asexual. ITS barcode: KX885061 (alternative markers: *BenA* = KX885041; *CaM* = KX885051; *RPB2* = KX885032).

***Penicillium balearicum*** Guevara-Suarez *et al.*, Fungal Syst. Evol. 5: 54. 2019 [2020]. [MB822061]. — Type: CBS H-23215. Ex-type: CBS 143044 = FMR 15191. Infragen. class: subgen. *Penicillium*, sect. *Paradoxa*, ser. *Atramentosa*. Reproduction: asexual. ITS barcode: LT899762 (alternative markers: *BenA* = LT898227; *CaM* = LT899758; *RPB2* = LT899760).

***Penicillium beceitense*** Guevara-Suarez *et al.*, Fungal Syst. Evol. 5: 55. 2019 [2020] [MB822063]. — Type: CBS H-23183. Ex-type: CBS 142989 = FMR 15038. Infragen. class: subgen. *Penicillium*, sect. *Ramosum*, ser. *Lanosa*. Reproduction: asexual. ITS barcode: LT899780 (alternative markers: *BenA* = LT898229; *CaM* = LT899764; *RPB2* = LT899798).

***Penicillium bialowiezense*** K.W. Zaleski, Bull. Int. Acad. Polon. Sci., Sér. B., Sci. Nat. 1927: 450. 1927. [MB258429]. — Type: IMI 092237. Ex-type: CBS 227.28 = IBT 23044 = IMI 092237 = LSHBP 71. Infragen. class: subgen. *Penicillium*, sect. *Brevicompacta*, ser. *Brevicompacta*. Reproduction: asexual. ITS barcode: EU587315 (alternative markers: *BenA* = AY674439; *CaM* = AY484828; *RPB2* = JN406604).

***Penicillium biforme*** Thom, U.S.D.A. Bur. Animal Industr. Bull. 118: 54. 1910. [MB240764]. — Type: unknown. Ex-type: DTO 060-F9 = DTO 060-F8 = CBS 297.48 = ATCC 10416 = FRR 885 = IFO 7722 = IMI 039820 = LSHB P72 = MUCL 29165 = NRRL 885 = QM 7492. Infragen. class: subgen. *Penicillium*, sect. *Fasciculata*, ser. *Camembertiorum*. Reproduction: asexual. ITS barcode: KC411731 (alternative markers: *BenA* = MN969373; *CaM* = KU896823; *RPB2* = KU904346).

***Penicillium bilaiae*** Chalab., Bot. Mater. Otd. Sporov. Rast. 6: 165. 1950. [MB302379]. — Type: IMI 113677. Ex-type: CBS 221.66 = ATCC 22348 = ATCC 48731 = CCRC 31675 = FRR 3391 = IJFM 5025 = IMI 113677 = MUCL 31187 = VKMF-854. Infragen. class: subgen. *Aspergilloides*, sect. *Sclerotiorum*, ser. *Adametziorum*. Reproduction: asexual. ITS barcode: JN714937 (alternative markers: *BenA* = JN625966; *CaM* = JN626009; *RPB2* = JN406610).

***Penicillium bissettii*** Visagie & Seifert, Persoonia 36: 269. 2016. [MB815778]. — Type: DAOM 695761. Ex-type: DAOMC 167011 = CBS 140972 = KAS 1951. Infragen. class: subgen. *Aspergilloides*, sect. *Lanata-Divaricata*, ser. *Rolfsiorum*. Reproduction: asexual. ITS barcode: KT887845 (alternative markers: *BenA* = KT887806; *CaM* = KT887767; *RPB2* = MN969178).

***Penicillium boreae*** S.W. Peterson & Sigler, Mycol. Res. 106: 1112. 2002. [MB483980]. — Type: BPI 841395. Ex-type: CBS 111717 = NRRL 31002 = UAMH 3896. Infragen. class: subgen. *Aspergilloides*, sect. *Stolkia*, ser. *Stolkia*. Reproduction: asexual. ITS barcode: AF481122 (alternative markers: *BenA* = JN617715; *CaM* = AF481138; *RPB2* = MN969107).

***Penicillium bovifimosum*** (Tuthill & Frisvad) Houbraken & Samson, Stud. Mycol. 70: 47. 2011. [MB561957]. Basionym: *Eupenicillium bovifimosum* Tuthill & Frisvad, Mycologia 94: 241. 2002. [MB456124]. — Type: WY RMF 82071. Ex-type: CBS 102825 = RMF 9598. Infragen. class: subgen. *Penicillium*, sect. *Turbata*, ser. *Turbata*. Reproduction: homothallic. ITS barcode: AF263347 (alternative markers: *BenA* = KJ834436; *CaM* = FJ530989; *RPB2* = JN406649).

***Penicillium brasilianum*** Bat., Anais Soc. Biol. Pernambuco 15: 162. 1957. [MB302381]. — Type: URM IMUR 56. Ex-type: CBS 253.55 = DTO 015-D3 = DTO 095-C4 = ATCC 12072 = FRR 3466 = QM 6947. Infragen. class: subgen. *Aspergilloides*, sect. *Lanata-Divaricata*, ser. *Simplicissima*. Reproduction: asexual. ITS barcode: GU981577 (alternative markers: *BenA* = GU981629; *CaM* = MN969239; *RPB2* = KF296420).

***Penicillium brefeldianum*** B.O. Dodge, Mycologia 25: 92. 1933. [MB258851]. — Type: IMI 216896. Ex-type: CBS 235.81 = NRRL 710 = FRR 710 = IFO 31731 = IMI 216896 = LCP 89.2573 = LCP 89.2578 = MUCL 38762 = QM 1872 = Thom 5296. Infragen. class: subgen. *Aspergilloides*, sect. *Lanata-Divaricata*, ser. *Janthinella*. Reproduction: homothallic. ITS barcode: AF033435 (alternative markers: *BenA* = GU981623; *CaM* = EU021683; *RPB2* = KF296421).

***Penicillium brevicompactum*** Dierckx, Ann. Soc. Sci. Bruxelles 25: 88. 1901. [MB149773]. — Type: IMI 40225. Ex-type: CBS 257.29 = ATCC 10418 = ATCC 9056 = DSM3825 = FRR 862 = IBT 23045 = IMI 040225 = LSHBP 75 = MUCL 28647 = MUCL 28813 = MUCL 28935 = MUCL 30240 = MUCL 30241 = MUCL 30256 = MUCL 30257 = NRRL 2011 = NRRL 862 = NRRL 864 = QM 7496. Infragen. class: subgen. *Penicillium*, sect. *Brevicompacta*, ser. *Brevicompacta*. Reproduction: asexual. ITS barcode: AY484912 (alternative markers: *BenA* = AY674437; *CaM* = AY484813; *RPB2* = JN406594).

***Penicillium brevistipitatum*** L. Wang & W.Y. Zhuang, Mycotaxon 93: 234. 2005. [MB356064]. — Type: HMAS 130354-1-4. Ex-type: DTO 105-I7 = CBS 122277 = AS 3.6887. Infragen. class: subgen. *Penicillium*, sect. *Robsamsonia*, ser. *Robsamsonia*. Reproduction: asexual. ITS barcode: DQ221696 (alternative markers: *BenA* = DQ221695; *CaM* = KU896824; *RPB2* = JN406528).

***Penicillium brocae*** S.W. Peterson *et al.*, Mycologia 95: 143. 2003. [MB373658]. — Type: BPI 841763. Ex-type: CBS 116113 = IBT 26293 = NRRL 31472. Infragen. class: subgen. *Aspergilloides*, sect. *Sclerotiorum*, ser. *Adametziorum*. Reproduction: asexual. ITS barcode: AF484398 (alternative markers: *BenA* = KC773787; *CaM* = KC773814; *RPB2* = JN406639).

***Penicillium brunneoconidiatum*** Visagie *et al.*, Stud. Mycol. 78: 415. 2014. [MB809958]. — Type: CBS H-21873. Ex-type: CBS 137732 = DTO 182-E4 = CV 949 = DAOM 241359. Infragen. class: subgen. *Aspergilloides*, sect. *Aspergilloides*, ser. *Pinetorum*. Reproduction: asexual. ITS barcode: KM189666 (alternative markers: *BenA* = KM088911; *CaM* = KM089298; *RPB2* = KM089685).

***Penicillium buchwaldii*** Frisvad & Samson, FEMS Microbiol. Lett. 339: 86. 2013. [MB800966]. — Type: IMI 304286. Ex-type: CBS 117181 = IBT 6005 = IMI 304286. Infragen. class: subgen. *Penicillium*, sect. *Brevicompacta*, ser. *Buchwaldiorum*. Reproduction: asexual. ITS barcode: JX313164 (alternative markers: *BenA* = MN969374; *CaM* = JX313148; *RPB2* = JN406637).

***Penicillium burgense*** Quintan. ex Visagie, IMA Fungus 7: 94. 2016. [MB816641]. — Type: CBS H-22567. Ex-type: CBS 325.89. Infragen. class: subgen. *Aspergilloides*, sect. *Exilicaulis*, ser. *Lapidosa*. Reproduction: asexual. ITS barcode: KC411736 (alternative markers: *BenA* = KJ834437; *CaM* = KP016772; *RPB2* = JN406572).

***Penicillium bussumense*** Houbraken, Stud. Mycol. 78: 415. 2014. [MB809959]. — Type: CBS H-21869. Ex-type: CBS 138160 = DTO 018-B2. Infragen. class: subgen. *Aspergilloides*, sect. *Aspergilloides*, ser. *Glabra*. Reproduction: asexual. ITS barcode: KM189458 (alternative markers: *BenA* = KM088685; *CaM* = KM089070; *RPB2* = KM089457).

***Penicillium cainii*** K.G. Rivera *et al.*, Stud. Mycol. 70: 147. 2011. [MB563159]. — Type: DAOM 239914. Ex-type: CCFC 239914 = DTO 328-C1. Infragen. class: subgen. *Aspergilloides*, sect. *Sclerotiorum*, ser. *Sclerotiorum*. Reproduction: asexual. ITS barcode: JN686435 (alternative markers: *BenA* = JN686366; *CaM* = JN686389; *RPB2* = MT156346).

***Penicillium cairnsense*** Houbraken *et al.*, Stud. Mycol. 70: 83. 2011. [MB563184]. — Type: CBS H-20686. Ex-type: CBS 124325 = DTO 030-E6 = IBT 29042. Infragen. class: subgen. *Aspergilloides*, sect. *Citrina*, ser. *Westlingiorum*. Reproduction: asexual. ITS barcode: JN617669 (alternative markers: *BenA* = JN606693; *CaM* = MN969240; *RPB2* = MN969108).

***Penicillium camemberti*** Thom, U.S.D.A. Bur. Animal Industr. Bull. 82: 33. 1906. [MB175171]. — Type: IMI 27831. Ex-type: DTO 246-F1 = CBS 299.48 = ATCC 1105 = ATCC 4845 = FRR 878 = IBT 21508 = IMI 027831 = IMI 092200 = LCP 66.584 = LSHBP 11 = MUCL 29790 = NCTC 582 = NRRL 877 = NRRL 878. Infragen. class: subgen. *Penicillium*, sect. *Fasciculata*, ser. *Camembertiorum*. Reproduction: asexual. ITS barcode: AB479314 (alternative markers: *BenA* = FJ930956; *CaM* = KU896825; *RPB2* = MN969109).

***Penicillium camponotum*** Visagie *et al.*, Persoonia 36: 271. 2016. [MB815779]. — Type: DAOM 695762. Ex-type: DAOMC 250557 = CBS 140982 = NBBR-2-1 = W 471 = KAS 2177. Infragen. class: subgen. *Aspergilloides*, sect. *Lanata-Divaricata*, ser. *Rolfsiorum*. Reproduction: asexual. ITS barcode: KT887855 (alternative markers: *BenA* = KT887816; *CaM* = KT887777; *RPB2* = MN969179).

***Penicillium canariense*** S.W. Peterson & Sigler, Mycol. Res. 106: 1113. 2002. [MB483981]. — Type: BPI 841396. Ex-type: CBS 111720 = NRRL 31003 = IJFM 536 = UAMH 6403. Infragen. class: subgen. *Aspergilloides*, sect. *Stolkia*, ser. *Stolkia*. Reproduction: asexual. ITS barcode: AF481121 (alternative markers: *BenA* = JN617714; *CaM* = AF481137; *RPB2* = MN969110).

***Penicillium canescens*** Sopp, Skr. Vidensk.-Selsk. Christiana Math.-Nat. Kl. 11: 181. 1912. [MB153765]. — Type: IMI 28260. Ex-type: CBS 300.48 = ATCC 10419 = DSM1215 = FRR 910 = IMI 028260 = MUCL 29169 = NCTC 6607 = NRRL 910 = QM 7550 = VKMF-1148. Infragen. class: subgen. *Penicillium*, sect. *Canescentia*, ser. *Canescentia*. Reproduction: asexual. ITS barcode: AF033493 (alternative markers: *BenA* = JX140946; *CaM* = MN969241; *RPB2* = JN121485).

***Penicillium canis*** S.W. Peterson, J. Clin. Microbiol. 52: 2450. 2014. [MB807056]. — Type: BPI 892763. Ex-type: NRRL 62798. Infragen. class: subgen. *Aspergilloides*, sect. *Exilicaulis*, ser. *Erubescentia*. Reproduction: asexual. ITS barcode: KJ511291 (alternative markers: *BenA* = KF900167; *CaM* = KF900177; *RPB2* = KF900196).

***Penicillium cantabricum*** Visagie & Samson, Persoonia 36: 142. 2016. [MB808263]. — Type: CBS H-21612. Ex-type: CBS 120415 = DTO 076-I9 = FMR 9121. Infragen. class: subgen. *Aspergilloides*, sect. *Torulomyces*, ser. *Torulomyces*. Reproduction: asexual. ITS barcode: KF303655 (alternative markers: *BenA* = KF303615; *CaM* = KF303646; *RPB2* = KF303682).

***Penicillium caperatum*** Udagawa & Y. Horie, Trans. Mycol. Soc. Japan 14: 371. 1973. [MB319262]. — Type: NHL 6454. Ex-type: CBS 443.75 = DTO 101-B2 = ATCC 28046 = DSM2209 = NHL 6465. Infragen. class: subgen. *Aspergilloides*, sect. *Lanata-Divaricata*, ser. *Janthinella*. Reproduction: homothallic. ITS barcode: KC411761 (alternative markers: *BenA* = GU981660; *CaM* = MN969242; *RPB2* = KF296422).

***Penicillium caprifimosum*** Guevara-Suarez *et al.*, Fungal Syst. Evol. 5: 55. 2019 [2020]. [MB822064]. — Type: CBS H-23184. Ex-type: CBS 142990 = FMR 15041. Infragen. class: subgen. *Penicillium*, sect. *Turbata*, ser. *Turbata*. Reproduction: asexual. ITS barcode: LT899781 (alternative markers: *BenA* = LT898238; *CaM* = LT899765; *RPB2* = LT899799).

***Penicillium capsulatum*** Raper & Fennell, Mycologia 40: 528. 1948. [MB289079]. — Type: IMI 40576. Ex-type: CBS 301.48 = ATCC 10420 = DSM2210 = FRR 2056 = IJFM 5120 = IMI 040576 = NRRL 2056 = QM 4869 = VKMF-445. Infragen. class: subgen. *Aspergilloides*, sect. *Ramigena*, ser. *Ramigena*. Reproduction: asexual. ITS barcode: AF033429 (alternative markers: *BenA* = MN969375; *CaM* = KP735539; *RPB2* = JN406582).

***Penicillium carneum*** (Frisvad) Frisvad, Microbiology 142: 546. 1996. [MB415652]. Basionym: *Penicillium roqueforti* var. *carneum* Frisvad, Mycologia 81: 858. 1990. [MB126415]. — Type: IMI 293204. Ex-type: CBS 112297 = IBT 6884 = IBT 18419 = IMI 293204. Infragen. class: subgen. *Penicillium*, sect. *Roquefortorum*, ser. *Roquefortorum*. Reproduction: asexual. ITS barcode: HQ442338 (alternative markers: *BenA* = AY674386; *CaM* = HQ442322; *RPB2* = JN406642).

***Penicillium cartierense*** Houbraken, Stud. Mycol. 78: 415. 2014. [MB809960]. — Type: CBS H-21861. Ex-type: CBS 137956 = DTO 092-H9. Infragen. class: subgen. *Aspergilloides*, sect. *Aspergilloides*, ser. *Thomiorum*. Reproduction: asexual. ITS barcode: KM189564 (alternative markers: *BenA* = KM088804; *CaM* = KM089189; *RPB2* = KM089576).

***Penicillium caseifulvum*** Lund *et al.*, J. Food Mycol. 1: 97. 1998. [MB446013]. — Type: C 24999. Ex-type: DTO 145-B8 = CBS 101134 = IBT 18282 = IBT 21510. Infragen. class: subgen. *Penicillium*, sect. *Fasciculata*, ser. *Camembertiorum*. Reproduction: asexual. ITS barcode: KJ834504 (alternative markers: *BenA* = AY674372; *CaM* = KU896826; *RPB2* = KU904347).

***Penicillium catalonicum*** Visagie & Samson, Persoonia 36: 142. 2016. [MB808265]. — Type: CBS H-21610. Ex-type: CBS 110532 = DTO 078-H5. Infragen. class: subgen. *Aspergilloides*, sect. *Torulomyces*, ser. *Torulomyces*. Reproduction: asexual. ITS barcode: KF303650 (alternative markers: *BenA* = KF303609; *CaM* = KF303644; *RPB2* = KF303683).

***Penicillium cataractarum*** Visagie *et al.*, Persoonia 36: 271. 2016. [MB819777]. — Type: DAOM 695763. Ex-type: DAOMC 250534 = CBS 140974 = DTO 410-D4 = W 4 = KAS 2145. Infragen. class: subgen. *Aspergilloides*, sect. *Lanata-Divaricata*, ser. *Simplicissima*. Reproduction: asexual. ITS barcode: KT887847 (alternative markers: *BenA* = KT887808; *CaM* = KT887769; *RPB2* = MN969180).

***Penicillium catenatum*** D.B. Scott, Mycopathol. Mycol. Appl. 36: 24. 1968. [MB335719]. — Type: CBS 352.67. Ex-type: CBS 352.67 = ATCC 18543 = CSIR 1097 = IFO 31774 = IMI 136241. Infragen. class: subgen. *Aspergilloides*, sect. *Exilicaulis*, ser. *Erubescentia*. Reproduction: homothallic. ITS barcode: KC411754 (alternative markers: *BenA* = KJ834438; *CaM* = KP016774; *RPB2* = JN121504).

***Penicillium cavernicola*** Frisvad & Samson, Stud. Mycol. 49: 31. 2004. [MB370976]. — Type: CBS H-13441. Ex-type: DTO 047-C1 = CBS 100540 = IBT 14499. Infragen. class: subgen. *Penicillium*, sect. *Fasciculata*, ser. *Camembertiorum*. Reproduction: asexual. ITS barcode: KJ834505 (alternative markers: *BenA* = KJ834439; *CaM* = KU896827; *RPB2* = KU904348).

***Penicillium chalabudae*** Visagie, IMA Fungus 7: 94. 2016. [MB816642]. — Type: CBS H-15439. Ex-type: CBS 219.66 = ATCC 18322 = ATCC 18329 = FRR 3393 = VKM F-1037. Infragen. class: subgen. *Aspergilloides*, sect. *Exilicaulis*, ser. *Restricta*. Reproduction: asexual. ITS barcode: KP016811 (alternative markers: *BenA* = KP016748; *CaM* = KP016767; *RPB2* = KP064572).

***Penicillium charlesii*** G. Sm., Trans. Brit. Mycol. Soc. 18: 90. 1933. [MB260433]. — Type: NRRL 778. Ex-type: CBS 304.48 = ATCC 8730 = CBS 342.51 = CECT 2277 = FRR 778 = IMI 040232 = LSHBBB127 = LSHBP 146 = NRRL 1887 = NRRL 778 = QM 6338 = QM 6838. Infragen. class: subgen. *Aspergilloides*, sect. *Charlesia*, ser. *Fellutana*. Reproduction: asexual. ITS barcode: AF033400 (alternative markers: *BenA* = JX091508; *CaM* = AY741727; *RPB2* = JN121486).

***Penicillium chermesinum*** Biourge, Cellule 33: 284. 1923. [MB260472]. — Type: IMI 191730. Ex-type: CBS 231.81 = FRR 2048 = IFO 31745 = IMI 191730 = NRRL 2048. Infragen. class: subgen. *Aspergilloides*, sect. *Charlesia*, ser. *Indica*. Reproduction: asexual. ITS barcode: AY742693 (alternative markers: *BenA* = KJ834441; *CaM* = AY741728; *RPB2* = MN969111).

***Penicillium choerospondiatis*** X.C. Wang & W.Y. Zhuang, Sci. Rep. 7: 8233, 5. 2017. [MB570333]. — Type: HMAS 248813. Ex-type: CGMCC 3.18411. Infragen. class: subgen. *Aspergilloides*, sect. *Sclerotiorum*, ser. *Herqueorum*. Reproduction: asexual. ITS barcode: KX885063 (alternative markers: *BenA* = KX885043; *CaM* = KX885053; *RPB2* = KX885034).

***Penicillium christenseniae*** Houbraken *et al.*, Stud. Mycol. 70: 85. 2011. [MB563187]. — Type: CBS H-20656. Ex-type: CBS 126236 = DTO 076-C3 = IBT 23355. Infragen. class: subgen. *Aspergilloides*, sect. *Citrina*, ser. *Westlingiorum*. Reproduction: asexual. ITS barcode: JN617674 (alternative markers: *BenA* = JN606680; *CaM* = MN969243; *RPB2* = JN606624).

***Penicillium chroogomphum*** F. Xu *et al.*, Mycoscience 57: 82. 2016. [MB813567]. — Type: JZBHM 002. Ex-type: CBS 136204 = DTO 351-H3 = KCTC 46041 = JZB 2120005. Infragen. class: subgen. *Penicillium*, sect. *Ramosum*, ser. *Soppiorum*. Reproduction: asexual. ITS barcode: KC594043 (alternative markers: *BenA* = KP684056; *CaM* = KP684057; *RPB2* = MN969167).

***Penicillium chrysogenum*** Thom, U.S.D.A. Bur. Animal Industr. Bull. 118: 58. 1910. [MB165757]. — Type: IMI 24314. Ex-type: DTO 012-I1 = CBS 306.48 = ATCC 10106 = ATHUM2889 = CCRC 30564 = FRR 807 = IBT 5233 = IMI 024314 = IMI 092208 = LSHBAd 3 = LSHBP 19 = MUCL 29079 = MUCL 29145 = NCTC 589 = NRRL 807 = NRRL 810 = QM 7500. Infragen. class: subgen. *Penicillium*, sect. *Chrysogena*, ser. *Chrysogena*. Reproduction: protoheterothallic (Henk *et al.* 2011); sexual reproduction described by Böhm *et al.* 2013, reidentified as *P*. *rubens* (Houbraken *et al.* 2014a). ITS barcode: AF033465 (alternative markers: *BenA* = JF909955; *CaM* = JX996273; *RPB2* = JN121487).

***Penicillium chrzaszczii*** K.W. Zaleski, Bull. Int. Acad. Polon. Sci., Sér. B., Sci. Nat. 1927: 464. 1927. [MB260609]. — Type: CBS 217.28 (lectotype, Houbraken *et al.* 2011b). Ex-type: CBS 217.28 = DTO 022-E4 = FRR 903 = MUCL 29167 = NRRL 1741 = NRRL 903. Infragen. class: subgen. *Aspergilloides*, sect. *Citrina*, ser. *Westlingiorum*. Reproduction: asexual. ITS barcode: GU944603 (alternative markers: *BenA* = JN606758; *CaM* = MN969244; *RPB2* = JN606628).

***Penicillium cinerascens*** Biourge, Cellule 33: 308, 1923. [MB260785]. — Type: IMI 92234. Ex-type: DTO 189-A9 = NRRL 748 = ATCC 48693 = BIOURGE 90 = FRR 748 = IMI 92234 = QM 7555 = Thom 4733.34. Infragen. class: subgen. *Aspergilloides*, sect. *Exilicaulis*, ser. *Citreonigra*. Reproduction: asexual. ITS barcode: AF033455 (alternative markers: *BenA* = JX141041; *CaM* = JX157405; *RPB2* = MN969112).

***Penicillium cinereoatrum*** Chalab., Bot. Mater. Otd. Sporov. Rast. 6: 167. 1950. [MB302385]. — Type: CBS H-7469. Ex-type: CBS 222.66 = ATCC 22350 = FRR 3390 = IJFM 5024 = IMI 113676 = VKMF-856. Infragen. class: subgen. *Aspergilloides*, sect. *Exilicaulis*, ser. *Restricta*. Reproduction: asexual. ITS barcode: KC411700 (alternative markers: *BenA* = KJ834442; *CaM* = KP125335; *RPB2* = JN406608).

***Penicillium cinnamopurpureum*** Abe ex Udagawa, J. Agric. Food Sci. 5: 1. 1959. [MB302386]. — Type: unknown. Ex-type: CBS 429.65 = CBS 847.68 = NRRL 162 = ATCC 18489 = CSIR 936 = FAT 362 = IAM 7016 = IFO 6032 = NHL 6359 = QM 7888. Infragen. class: subgen. *Aspergilloides*, sect. *Cinnamopurpurea*, ser. *Cinnamopurpurea*. Reproduction: homothallic (Scott & Stolk 1967). ITS barcode: EF626950 (alternative markers: *BenA* = EF626948; *CaM* = EF626949; *RPB2* = JN406533).

***Penicillium circulare*** Hyang B. Lee *et al.*, Fungal Diversity 96: 97. 2019. [MB555413]. — Type: CNUFC-GEU220-1. Ex-type: CNUFC-GEU220-1. Infragen. class: subgen. *Aspergilloides*, sect. *Sclerotiorum*, ser. *Sclerotiorum*. Reproduction: asexual. ITS barcode: n.a. (alternative markers: *BenA* = MK481057; *CaM* = MK481061; *RPB2* = MK481053).

***Penicillium citreonigrum*** Dierckx, Ann. Soc. Sci. Bruxelles 25: 86. 1901. [MB165197]. — Type: IMI 92209i. Ex-type: CBS 258.29 = ATCC 48736 = FRR 761 = IMI 092209 = LSHBP 20 = LSHBP 98 = MUCL 28648 = MUCL 29062 = MUCL 29116 = NRRL 761. Infragen. class: subgen. *Aspergilloides*, sect. *Exilicaulis*, ser. *Citreonigra*. Reproduction: asexual. ITS barcode: AF033456 (alternative markers: *BenA* = EF198621; *CaM* = EF198628; *RPB2* = JN121474).

***Penicillium citreosulfuratum*** Biourge, Cellule 33: 285, 1923. [MB260947]. — Type: France: source unknown; in Biourge, Cellule 33: Fig. 86, no. 21 (lectotype, designated in Visagie *et al.* 2016c, MBT203135); IMI 92228 (epitype). Ex-epitype: IMI 92228 = DTO 290-I4. Infragen. class: subgen. *Aspergilloides*, sect. *Exilicaulis*, ser. *Citreonigra*. Reproduction: asexual. ITS barcode: KP016814 (alternative markers: *BenA* = KP016753; *CaM* = KP016777; *RPB2* = KP064615).

***Penicillium citrinum*** Thom, U.S.D.A. Bur. Animal Industr. Bull. 118: 61. 1910. [MB165293]. — Type: IMI 92196ii. Ex-type: CBS 139.45 = DTO 022-F3 = ATCC 1109 = ATCC 36382 = CECT 2269 = FRR 1841 = IMI 091961 = IMI 092196 = LSHBAd 95 = LSHBP 25 = LSHBP 6 = MUCL 29781 = NRRL 1841 = NRRL 1842. Infragen. class: subgen. *Aspergilloides*, sect. *Citrina*, ser. *Citrina*. Reproduction: asexual. ITS barcode: AF033422 (alternative markers: *BenA* = GU944545; *CaM* = MN969245; *RPB2* = JF417416).

***Penicillium clavigerum*** Demelius, Verh. Zool.-Bot. Ges. Wien 72: 74. 1923. [MB261069]. — Type: IMI 39807. Ex-type: DTO 248-F6 = CBS 310.48 = ATCC 10427 = CBS 255.94 = FRR 1003 = IMI 039807 = IMI 039807ii = MUCL 15623 = NRRL 1003 = QM 1918. Infragen. class: subgen. *Penicillium*, sect. *Penicillium*, ser. *Clavigera*. Reproduction: asexual. ITS barcode: DQ339555 (alternative markers: *BenA* = AY674427; *CaM* = KU896828; *RPB2* = KU904349).

***Penicillium clavistipitatum*** Visagie *et al.*, Stud. Mycol. 78: 419. 2014. [MB809961]. — Type: CBS H-21882. Ex-type: CBS 138650 = DTO 182-E5 = CV 336 = KAS 4112 = DAOM 241092. Infragen. class: subgen. *Aspergilloides*, sect. *Aspergilloides*, ser. *Pinetorum*. Reproduction: asexual. ITS barcode: KM189667 (alternative markers: *BenA* = KM088912; *CaM* = KM089299; *RPB2* = KM089686).

***Penicillium cluniae*** Quintan. *nom*. *inval*. (Art. 40.7), Av. Aliment. Mejora Anim. 30: 174. 1990. [MB130240]. — Type: n.a.; typification needs correction. Ex-type: CBS 326.89 = DTO 265-A8. Infragen. class: subgen. *Aspergilloides*, sect. *Lanata-Divaricata*, ser. *Janthinella*. Reproduction: asexual. ITS barcode: MN431386 (alternative markers: *BenA* = MN969376; *CaM* = MN969246; *RPB2* = KF296424).

***Penicillium coccotrypicola*** Holdom *et al.*, Persoonia 33: 285. [MB810327]. — Type: BRIP 59608. Ex-type: BRIP 59608 = DTO 354-F5. Infragen. class: subgen. *Penicillium*, sect. *Penicillium*, ser. *Clavigera*. Reproduction: asexual. ITS barcode: KM605436 (alternative markers: *BenA* = KM605437; *CaM* = MN969321; *RPB2* = n.a.).

***Penicillium coeruleum*** Sopp in Biourge, Cellule 33: 102. 1923. [MB446014]. — Type: unknown. Ex-type: CBS 141.45 = DTO 035-H5 = NCTC 6595. Infragen. class: subgen. *Aspergilloides*, sect. *Lanata-Divaricata*, ser. *Janthinella*. Reproduction: asexual. ITS barcode: GU981606 (alternative markers: *BenA* = GU981655; *CaM* = MN969247; *RPB2* = KF296425).

***Penicillium coffeae*** S.W. Peterson *et al.*, Mycologia 97: 662. 2005. [MB340281]. — Type: BPI 863480. Ex-type: CBS 119387 = IBT 27866 = NRRL 35363. Infragen. class: subgen. *Aspergilloides*, sect. *Charlesia*, ser. *Phoenicea*. Reproduction: asexual. ITS barcode: AY742702 (alternative markers: *BenA* = KJ834443; *CaM* = AY741747; *RPB2* = JN121436).

***Penicillium colei*** S.W. Peterson *et al.*, PLoS ONE 10: 0121987, 10. 2015. [MB807368]. — Type: BPI 881281. Ex-type: NRRL 13013 = IBT 29696. Infragen. class: subgen. *Aspergilloides*, sect. *Cinnamopurpurea*, ser. *Idahoensia*. Reproduction: asexual. ITS barcode: KF932958 (alternative markers: *BenA* = KF932926; *CaM* = KF932942; *RPB2* = KF932996).

***Penicillium commune*** Thom, U.S.D.A. Bur. Animal Industr. Bull. 118: 56. 1910. [MB164241]. — Type: IMI 39812. Ex-type: DTO 052-F2 = CBS 311.48 = ATCC 10428 = ATCC 1111 = CCRC 31554 = DSM2211 = IBT 6200 = IFO 5763 = IMI 039812ii = IMI 039812iii = NRRL 890 = QM 1269 = VKMF-3233. Infragen. class: subgen. *Penicillium*, sect. *Fasciculata*, ser. *Camembertiorum*. Reproduction: asexual. ITS barcode: AY213672 (alternative markers: *BenA* = MN969377; *CaM* = KU896829; *RPB2* = KU904350).

***Penicillium compactum*** L. Wang & Houbraken, Persoonia 36: 309. 2016. [MB810216]. — Type: HMAS 245701. Ex-type: AS 3.15411 = DTO 316-B8 = CBS 138918 = IBT 33393. Infragen. class: subgen. *Penicillium*, sect. *Robsamsonia*, ser. *Robsamsonia*. Reproduction: asexual. ITS barcode: KM973207 (alternative markers: *BenA* = KM973203; *CaM* = KM973200; *RPB2* = KT698909).

***Penicillium concentricum*** Samson *et al.*, Stud. Mycol. 11: 17. 1976. [MB319263]. — Type: CBS 477.75. Ex-type: CBS 477.75 = IBT 14571 = IBT 6577. Infragen. class: subgen. *Penicillium*, sect. *Robsamsonia*, ser. *Robsamsonia*. Reproduction: asexual. ITS barcode: KC411763 (alternative markers: *BenA* = AY674413; *CaM* = DQ911131; *RPB2* = KT900575).

***Penicillium confertum*** (Frisvad *et al.*) Frisvad, Mycologia 81: 852. 1990. [MB126404]. Basionym: *Penicillium glandicola* var. *confertum* Frisvad *et al.*, Canad. J. Bot. 65: 769. 1987. [MB131769]. — Type: IMI 296930. Ex-type: CBS 171.87 = IBT 21515 = IBT 3098 = IBT 5672 = IMI 296930 = NRRL 13488 = NRRL A-26904. Infragen. class: subgen. *Penicillium*, sect. *Chrysogena*, ser. *Chrysogena*. Reproduction: asexual. ITS barcode: JX997081 (alternative markers: *BenA* = AY674373; *CaM* = JX996963; *RPB2* = JX996708).

***Penicillium coniferophilum*** Houbraken & Samson, Stud. Mycol. 70: 47. 2011. [MB561968]. Replaced synonym: *Thysanophora striatispora* G.L. Barron & W.B. Cooke, Mycopathol. Mycol. Appl. 40 (3–4): 353. 1970. [MB324607]. — Type: unknown. Ex-type: n.a. Infragen. class: subgen. *Aspergilloides*, sect. *Thysanophora*, ser. *Thysanophora*. Reproduction: asexual. ITS barcode: n.a. (alternative markers: *BenA* = n.a.; *CaM* = n.a.; *RPB2* = n.a.).

***Penicillium consobrinum*** Visagie & K. Jacobs, IMA Fungus 7: 96. 2016. [MB811002]. — Type: CBS H-22045. Ex-type: CBS 139144 = DAOMC 241072 = DTO 181-H9 = CV547. Infragen. class: subgen. *Aspergilloides*, sect. *Exilicaulis*, ser. *Corylophila*. Reproduction: asexual. ITS barcode: JX140888 (alternative markers: *BenA* = JX141135; *CaM* = JX157453; *RPB2* = KP064619).

***Penicillium contaminatum*** Houbraken, Stud. Mycol. 78: 419. 2014. [MB809962]. — Type: CBS H-21866. Ex-type: CBS 345.52 = DTO 091-A3 = IMI 049057. Infragen. class: subgen. *Aspergilloides*, sect. *Aspergilloides*, ser. *Thomiorum*. Reproduction: asexual. ITS barcode: KM189554 (alternative markers: *BenA* = KM088793; *CaM* = KM089178; *RPB2* = KM089565).

***Penicillium coprobium*** Frisvad, Mycologia 81: 853. 1990. [MB126405]. — Type: IMI 293209. Ex-type: CBS 561.90 = ATCC 58615 = IBT 21516 = IBT 4583 = IBT 6932. Infragen. class: subgen. *Penicillium*, sect. *Robsamsonia*, ser. *Robsamsonia*. Reproduction: asexual. ITS barcode: DQ339559 (alternative markers: *BenA* = AY674425; *CaM* = KU896830; *RPB2* = KT900576).

***Penicillium coprophilum*** (Berk. & M.A. Curtis) Seifert & Samson, Adv. Pen. Asp. Syst.: 145. 1986 [1985]. [MB114760]. Basionym: *Coremium coprophilum* Berk. & M.A. Curtis, J. Linn. Soc., Bot., 10: 363. 1868. [MB150510]. — Type: K(M), Wright 666; CBS 111760 (epitype, in Frisvad & Samson 2004b). Ex-epitype: CBS 110760 = IBT 5551 = IBT 3064 = NRRL 13627. Infragen. class: subgen. *Penicillium*, sect. *Robsamsonia*, ser. *Robsamsonia*. Reproduction: asexual. ITS barcode: AF033469 (alternative markers: *BenA* = AY674421; *CaM* = KU896831; *RPB2* = JN406645).

***Penicillium copticola*** Houbraken *et al.*, Stud. Mycol. 70: 88. 2011. [MB563205]. — Type: CBS H-20643. Ex-type: CBS 127355 = IBT 30771. Infragen. class: subgen. *Aspergilloides*, sect. *Citrina*, ser. *Copticolarum*. Reproduction: asexual. ITS barcode: JN617685 (alternative markers: *BenA* = JN606817; *CaM* = JN606553; *RPB2* = JN606599).

***Penicillium coralligerum*** Nicot & Pionnat, Bull. Soc. Mycol. France 78: 245. 1963 [1962]. [MB335721]. — Type: IMI 99159. Ex-type: CBS 123.65 = ATCC 16968 = FRR 3465 = IFO 9578 = IHEM 4511 = IMI 099159 = LCP 58.1674 = NRRL 3465 = DTO 104-D9. Infragen. class: subgen. *Penicillium*, sect. *Canescentia*, ser. *Atroveneta*. Reproduction: asexual. ITS barcode: JN617667 (alternative markers: *BenA* = MN969378; *CaM* = MN969248; *RPB2* = JN406632).

***Penicillium corvianum*** Visagie & Seifert, Persoonia 36: 259. 2016. [MB815770]. — Type: DAOM 695764. Ex-type: DAOMC 250517 = CBS 141000 = DTO 412-B3 = KAS 3618 = IT-2008-4-D. Infragen. class: subgen. *Penicillium*, sect. *Canescentia*, ser. *Canescentia*. Reproduction: asexual. ITS barcode: KT887875 (alternative markers: *BenA* = KT887836; *CaM* = KT887797; *RPB2* = MN969170).

***Penicillium corylophilum*** Dierckx, Ann. Soc. Sci. Bruxelles 25: 86. 1901. [MB178294]. — Type: IMI 39754. Ex-type: CBS 312.48 = TCC9784 = ATHUM2890 = CECT 2270 = FRR 802 = IMI 039754 = MUCL 28671 = MUCL 29073 = MUCL 29131 = NRRL 802 = QM 7510. Infragen. class: subgen. *Aspergilloides*, sect. *Exilicaulis*, ser. *Corylophila*. Reproduction: asexual. ITS barcode: AF033450 (alternative markers: *BenA* = JX141042; *CaM* = KP016780; *RPB2* = KP064631).

***Penicillium cosmopolitanum*** Houbraken *et al.*, Stud. Mycol. 70: 91. 2011. [MB563188]. — Type: CBS H-20665. Ex-type: CBS 126995 = DTO 092-E8 = IBT 30681. Infragen. class: subgen. *Aspergilloides*, sect. *Citrina*, ser. *Westlingiorum*. Reproduction: asexual. ITS barcode: JN617691 (alternative markers: *BenA* = JN606733; *CaM* = MN969249; *RPB2* = MN969113).

***Penicillium costaricense*** Visagie *et al.*, Persoonia 36: 263. 2016. [MB815773]. — Type: DAOM 695765. Ex-type: DAOMC 250520 = CBS 140998 = DTO 410-E3 = KAS 2597 = 01-RGTHC-294. Infragen. class: subgen. *Aspergilloides*, sect. *Charlesia*, ser. *Costaricensia*. Reproduction: asexual. ITS barcode: MN431396 (alternative markers: *BenA* = KT887834; *CaM* = KT887795; *RPB2* = MN969173).

***Penicillium cravenianum*** Visagie & K. Jacobs, IMA Fungus 7: 96. 2016. [MB811003]. — Type: CBS H-22044. Ex-type: CBS 139138 = DAOMC 241082 = DTO 180-I5 = CV 92. Infragen. class: subgen. *Aspergilloides*, sect. *Exilicaulis*, ser. *Corylophila*. Reproduction: asexual. ITS barcode: JX140900 (alternative markers: *BenA* = JX141076; *CaM* = JX157418; *RPB2* = KP064636).

***Penicillium cremeogriseum*** Chalab., Bot. Mater. Otd. Sporov. Rast. 6: 168. 1950. [MB302390]. — Type: CBS 223.66. Ex-type: CBS 223.66 = DTO 097-B1 = ATCC 18320 = ATCC 18323 = FRR 1734 = IJFM 5011 = IMI 197492 = NRRL 3389 = VKMF-1034. Infragen. class: subgen. *Aspergilloides*, sect. *Lanata-Divaricata*, ser. *Janthinella*. Reproduction: asexual. ITS barcode: GU981586 (alternative markers: *BenA* = GU981624; *CaM* = MN969250; *RPB2* = KF296426).

***Penicillium crocicola*** W. Yamam., Sci. Rep. Hyogo Univ. Agric. 2: 28. 1956. [MB302391]. — Type: CBS H-7528. Ex-type: CBS 745.70 = NRRL 6175 = ATCC 18313 = QM 7778. Infragen. class: subgen. *Aspergilloides*, sect. *Aspergilloides*, ser. *Thomiorum*. Reproduction: asexual. ITS barcode: KM189581 (alternative markers: *BenA* = KJ834445; *CaM* = KM089210; *RPB2* = JN406535).

***Penicillium crustosum*** Thom, The Penicillia: 399. 1930. [MB262401]. — Type: IMI 91917. Ex-type: CBS 115503 = ATCC 52044 = FRR 1669 = IBT 5528 = IBT 6175 = IMI 091917 = NCTC 4002. Infragen. class: subgen. *Penicillium*, sect. *Fasciculata*, ser. *Camembertiorum*. Reproduction: asexual. ITS barcode: AF033472 (alternative markers: *BenA* = MN969379; *CaM* = DQ911132; *RPB2* = MN969114).

***Penicillium cryptum*** Goch., Mycotaxon 26: 349. 1986. [MB103648]. — Type: NY 769. Ex-type: CBS 271.89 = ATCC 60138 = IMI 296794 = NRRL 13460 = DTO 122-C9. Infragen. class: subgen. *Aspergilloides*, sect. *Crypta*, ser. *Crypta*. Reproduction: homothallic. ITS barcode: KF303647 (alternative markers: *BenA* = KF303608; *CaM* = KF303628; *RPB2* = JN121478).

***Penicillium crystallinum*** (Kwon-Chung & Fennell) Samson *et al.*, Stud. Mycol. 78: 355. 2014. [MB809315]. Basionym: *Aspergillus crystallinus* Kwon-Chung & Fennell, Gen. Aspergillus: 471. 1965. [MB326624]. — Type: IMI 139270. Ex-type: CBS 479.65 = NRRL 5082 = ATCC 16833 = IMI 139270. Infragen. class: subgen. *Penicillium*, sect. *Paradoxa*, ser. *Paradoxa*. Reproduction: asexual. ITS barcode: AF033486 (alternative markers: *BenA* = EF669682; *CaM* = FJ530973; *RPB2* = EF669669).

***Penicillium cuddlyae*** Visagie & I.H. Rong, Persoonia 43: 38. 2019 [MB832433]. — Type: PREM 623302. Ex-type: PPRI 26355 = CMV016A6. Infragen. class: subgen. *Aspergilloides*, sect. *Charlesia*, ser. *Indica*. Reproduction: asexual. ITS barcode: MK951942 (alternative markers: *BenA* = MK951835; *CaM* = MK951908; *RPB2* = MN418450).

***Penicillium curticaule*** Visagie & K. Jacobs, Mycol. Prog. 14 (no. 96): 16. 2015. [MB809818]. — Type: CBS H-21334. Ex-type: CBS 135127 = CV 2842 = CV 0188 = DTO 180-D3 = DAOM 241159. Infragen. class: subgen. *Aspergilloides*, sect. *Lanata-Divaricata*, ser. *Janthinella*. Reproduction: asexual. ITS barcode: FJ231021 (alternative markers: *BenA* = JX091526; *CaM* = JX141536; *RPB2* = KF296417).

***Penicillium cvjetkovicii*** S.W. Peterson *et al.*, PLoS ONE 10: 0121987, 12. 2015. [MB807369]. — Type: BPI 881283. Ex-type: NRRL 35841 = IBT 29714. Infragen. class: subgen. *Aspergilloides*, sect. *Cinnamopurpurea*, ser. *Idahoensia*. Reproduction: asexual. ITS barcode: KF932963 (alternative markers: *BenA* = KF932931; *CaM* = KF932948; *RPB2* = KF933002).

***Penicillium cyaneum*** (Bainier & Sartory) Biourge, Cellule 33: 102. 1923. [MB251712]. Basionym: *Citromyces cyaneus* Bainier & Sartory, Bull. Soc. Mycol. France 29: 157. 1913. [MB178850]. — Type: IMI 39744. Ex-type: CBS 315.48 = ATCC 10432 = FRR 775 = IFO 5337 = IMI 039744 = NRRL 775 = QM 7516. Infragen. class: subgen. *Aspergilloides*, sect. *Ramigena*, ser. *Ramigena*. Reproduction: asexual. ITS barcode: AF033427 (alternative markers: *BenA* = JX091552; *CaM* = KP735540; *RPB2* = JN406575).

***Penicillium cyclopium*** Westling, Ark. Bot. 11: 90. 1911. [MB156739]. — Type: IMI 089372. Ex-type: DTO 163-I1 = CBS 144.45 = ATCC 8731 = ATHUM2888 = CECT 2264 = DSM1250 = IBT 5130 = IMI 089372 = LSHBP 123 = MUCL 15613 = NRRL 1888 = QM 6839 = VKMF-265. Infragen. class: subgen. *Penicillium*, sect. *Fasciculata*, ser. *Viridicata*. Reproduction: asexual. ITS barcode: JN097811 (alternative markers: *BenA* = MN969380; *CaM* = KU896832; *RPB2* = JN985388).

***Penicillium daejeonium*** S.H. Yu & H.K. Sang, J. Microbiol. 51: 537. 2013. [MB561572]. — Type: KACC 46609. Ex-type: KACC 46609. Infragen. class: subgen. *Aspergilloides*, sect. *Sclerotiorum*, ser. *Sclerotiorum*. Reproduction: asexual. ITS barcode: JX436489 (alternative markers: *BenA* = JX436493; *CaM* = JX436491; *RPB2* = n.a.).

***Penicillium daleae*** K.W. Zaleski, Bull. Int. Acad. Polon. Sci., Sér. B., Sci. Nat. 1927: 495. 1927. [MB262773]. — Type: IMI 89338. Ex-type: CBS 211.28 = DTO 105-F2 = ATCC 10435 = DSM 2449 = FRR 2025 = IFO 6087 = IFO 9072 = IMI 034910 = MUCL 29234 = NRRL 2025. Infragen. class: subgen. *Aspergilloides*, sect. *Lanata-Divaricata*, ser. *Dalearum*. Reproduction: asexual. ITS barcode: GU981583 (alternative markers: *BenA* = GU981649; *CaM* = MN969251; *RPB2* = KF296427).

***Penicillium decaturense*** S.W. Peterson *et al.*, Mycologia 96: 1290. 2004. [MB487890]. — Type: BPI 842267. Ex-type: CBS 117509 = DTO 003-F7 = NRRL 28152 = IBT 27117. Infragen. class: subgen. *Aspergilloides*, sect. *Citrina*, ser. *Westlingiorum*. Reproduction: asexual. ITS barcode: GU944604 (alternative markers: *BenA* = JN606685; *CaM* = MN969252; *RPB2* = JN606621).

***Penicillium decumbens*** Thom, U.S.D.A. Bur. Animal Industr. Bull. 118: 71. 1910. [MB156582]. — Type: IMI 190875. Ex-type: CBS 230.81 = FRR 741 = IMI 190875 = MUCL 29107 = NRRL 741. Infragen. class: subgen. *Aspergilloides*, sect. *Exilicaulis*, ser. *Alutacea*. Reproduction: asexual. ITS barcode: AY157490 (alternative markers: *BenA* = KJ834446; *CaM* = KP016782; *RPB2* = JN406601).

***Penicillium desertorum*** Frisvad *et al.*, Persoonia 29: 90. 2012. [MB801874]. — Type: CBS H-21056. Ex-type: DTO 148-I6 = CBS 131543 = IBT 16321. Infragen. class: subgen. *Penicillium*, sect. *Chrysogena*, ser. *Chrysogena*. Reproduction: asexual. ITS barcode: JX997011 (alternative markers: *BenA* = JX996818; *CaM* = JX996937; *RPB2* = JX996682).

***Penicillium diabolicalicense*** Visagie & Seifert, Persoonia 36: 265. 2016. [MB815775]. — Type: PDD 107542. Ex-type: DAOMC 250542 = CBS 140967 = KAS 1726. Infragen. class: subgen. *Aspergilloides*, sect. *Exilicaulis*, ser. *Lapidosa*. Reproduction: asexual. ITS barcode: KT887840 (alternative markers: *BenA* = KT887801; *CaM* = KT887762; *RPB2* = MN969175).

***Penicillium diatomitis*** Kubátová *et al.*, Mycol. Prog. 18 (1-2): 223. 2018. [MB824352]. — Type: PRM 861476. Ex-type: CCF 3904 = MH 53 = CBS 140107 = IBT 30728. Infragen. class: subgen. *Aspergilloides*, sect. *Lanata-Divaricata*, ser. *Oxalica*. Reproduction: asexual. ITS barcode: FJ430748 (alternative markers: *BenA* = HE651133; *CaM* = LT970912; *RPB2* = LT797560).

***Penicillium digitatum*** (Pers.) Sacc., Fung. Ital. Autogr. Delin.: tab. 894. 1881. Basionym: *Aspergillus digitatus* Pers., Disp. meth. Fung.: 41. 1794 ≡ *Monilia digitata* Pers., Syn. Meth. Fung.: 693. 1801. [*nom. sanct.*, Fr., Syst. Mycol. 3: 411. 1832]. [MB169502]. — Neotype: Saccardo, Fung. Ital. Autogr. Delin.: tab. 894. 1881 (lectotype); CBS 112082 (epitype, in Frisvad & Samson 2004b). Ex-type: CBS 112082 = IBT 13068. Infragen. class: subgen. *Penicillium*, sect. *Penicillium*, ser. *Digitata*. Reproduction: asexual. ITS barcode: KJ834506 (alternative markers: *BenA* = KJ834447; *CaM* = KU896833; *RPB2* = JN121426).

***Penicillium dimorphosporum*** H.J. Swart, Trans. Brit. Mycol. Soc. 55: 310. 1970. [MB120334]. — Type: CBS 456.70. Ex-type: CBS 456.70 = NRRL 5207 = ATCC 22783 = ATCC 52501 = FRR 1120 = IMI 149680. Infragen. class: subgen. *Aspergilloides*, sect. *Exilicaulis*, ser. *Erubescentia*. Reproduction: asexual. ITS barcode: AF081804 (alternative markers: *BenA* = KJ834448; *CaM* = KP016783; *RPB2* = JN121517).

***Penicillium dipodomyicola*** (Frisvad *et al.*) Frisvad, Int. Mod. Meth. Pen. Asp. Clas.: 275. 2000. [MB459818]. Basionym: *Penicillium griseofulvum* var. *dipodomyicola* Frisvad *et al.*, Canad. J. Bot. 65: 767. 1987. [MB131771]. — Type: IMI 296935. Ex-type: DTO 202-F7 = CBS 173.87 = IBT 21521 = IMI 296935 = ATCC 64187 = NRRL 13487. Infragen. class: subgen. *Penicillium*, sect. *Robsamsonia*, ser. *Urticicola*. Reproduction: asexual. ITS barcode: MN431387 (alternative markers: *BenA* = AY674409; *CaM* = KT900573; *RPB2* = KT900577).

***Penicillium dipodomyus*** [as “*dipodomyis*”] (Frisvad *et al.*) Banke *et al.*, Int. Mod. Meth. Pen. Asp. Clas.: 271. 2000. [MB459815]. Basionym: *Penicillium chrysogenum* var. *dipodomyus* Frisvad *et al.*, Canad. J. Bot. 65: 766. 1987. [MB635036]. — Type: IMI 296926. Ex-type: IBT 5333 = CBS 110412 = DTO 072-B6 = NRRL 13485 = NRRL A-26836 = IMI 296926. Infragen. class: subgen. *Penicillium*, sect. *Chrysogena*, ser. *Chrysogena*. Reproduction: protoheterothallic (Henk *et al.* 2011). ITS barcode: MN431359 (alternative markers: *BenA* = AY495991; *CaM* = JX996950; *RPB2* = JF909932).

***Penicillium discolor*** Frisvad & Samson, Antonie van Leeuwenhoek 72: 120. 1997. [MB442902]. — Type: IMI 285513. Ex-type: DTO 046-H9 = CBS 474.84 = IBT 21523 = IBT 5738 = IBT 14440 = IMI 285513 = FRR 2933. Infragen. class: subgen. *Penicillium*, sect. *Fasciculata*, ser. *Camembertiorum*. Reproduction: asexual. ITS barcode: AJ004816 (alternative markers: *BenA* = AY674348; *CaM* = KU896834; *RPB2* = KU904351).

***Penicillium dokdoense*** Hyang B. Lee & T.T.T. Nguyen, Fungal Diversity 95: 95. 2019. [MB554459]. — Type: CNUFC-DDS11-1. Ex-type: JMRC:SF:013606. Infragen. class: subgen. *Aspergilloides*, sect. *Citrina*, ser. *Copticolarum*. Reproduction: asexual. ITS barcode: MG906868 (alternative markers: *BenA* = MH243037; *CaM* = MH243031; *RPB2* = n.a.).

***Penicillium donkii*** Stolk, Persoonia 7: 333. 1973. [MB319267]. — Type: CBS 188.72. Ex-type: CBS 188.72 = NRRL 5562 = ATCC 48439 = CCRC 31694 = FRR 1738 = IFO 31746 = IMI 197489 = MUCL 31188. Infragen. class: subgen. *Aspergilloides*, sect. *Stolkia*, ser. *Stolkia*. Reproduction: asexual. ITS barcode: AF033445 (alternative markers: *BenA* = JN617718; *CaM* = AF481136; *RPB2* = MN969115).

***Penicillium dravuni*** Janso, Mycologia 97: 445. 2005. [MB501442]. — Type: BPI 844248. Ex-type: F01V25. Infragen. class: subgen. *Aspergilloides*, sect. *Exilicaulis*, ser. *Erubescentia*. Reproduction: asexual. ITS barcode: AY494856 (alternative markers: *BenA* = n.a.; *CaM* = n.a.; *RPB2* = n.a.).

***Penicillium dunedinense*** Visagie *et al.*, Stud. Mycol. 78: 121. 2014. [MB809183]. — Type: CBS H-21803. Ex-type: CBS 138218 = DTO 244-G1. Infragen. class: subgen. *Penicillium*, sect. *Canescentia*, ser. *Canescentia*. Reproduction: asexual. ITS barcode: KJ775678 (alternative markers: *BenA* = KJ775171; *CaM* = KJ775405; *RPB2* = MN969116).

***Penicillium echinulatum*** Raper & Thom ex Fassat., Acta Univ. Carol., Biol. 1974: 326. 1977. [MB319269]. — Type: PRM 778523. Ex-type: DTO 047-A4 = CBS 317.48 = IBT 6294 = IMI 040028 = ATCC 10434 = NRRL 1151 = FRR 1151 = IFO 7760 = MUCL 15615 = QM 7519. Infragen. class: subgen. *Penicillium*, sect. *Fasciculata*, ser. *Camembertiorum*. Reproduction: asexual. ITS barcode: AF033473 (alternative markers: *BenA* = AY674341; *CaM* = DQ911133; *RPB2* = KU904352).

***Penicillium echinulonalgiovense*** S. Abe ex Houbraken & R.N. Barbosa Antonie van Leeuwenhoek 111: 1895. 2018. [MB822213]. — Type: CBS H-23172. Ex-type: DTO 014-H5 = CBS 328.59 = ATCC 18314 = FAT 907 = FRR 638 = IFO 6229 = IMI 068213 = QM 7301. Infragen. class: subgen. *Aspergilloides*, sect. *Lanata-Divaricata*, ser. *Simplicissima*. Reproduction: asexual. ITS barcode: GU981587 (alternative markers: *BenA* = GU981631; *CaM* = KX961269; *RPB2* = KX961301).

***Penicillium egyptiacum*** J.F.H. Beyma, Zentralbl. Bakteriol. Parasitenk., Abt. 2 88: 137. 1933 [MB263790]. — Type: IMI 040580. Ex-type: CBS 244.32 = ATCC 10441 = CSIR 707 = FRR 2090 = IBT 14684 = IFO 6094 = IFO 8141 = IFO 8847 = IMI 040580 = NRRL 2090 = QM 1875. Infragen. class: subgen. *Penicillium*, sect. *Chrysogena*, ser. *Crustacea*. Reproduction: homothallic. ITS barcode: AF033467 (alternative markers: *BenA* = KU896810; *CaM* = JX996969; *RPB2* = JN406598).

***Penicillium ehrlichii*** Kleb., Ber. Deutsch. Bot. Ges. 48: 374. 1930. [MB319270]. — Type: IMI 039737. Ex-type: CBS 324.48 = DTO 097-D7 = ATCC 10442 = IMI 039737 = IMI 039737ii = NRRL 708 = QM 1874 = VKMF-273. Infragen. class: subgen. *Aspergilloides*, sect. *Lanata-Divaricata*, ser. *Janthinella*. Reproduction: homothallic. ITS barcode: GU981578 (alternative markers: *BenA* = GU981652; *CaM* = MN969253; *RPB2* = KF296428).

***Penicillium elleniae*** Houbraken *et al.*, Int. J. Syst. Evol. Microbiol. 61: 1470. 2011. [MB518028]. — Type: HUA 170339. Ex-type: CBS 118135 = DTO 057-I9 = IBT 23229. Infragen. class: subgen. *Aspergilloides*, sect. *Lanata-Divaricata*, ser. *Janthinella*. Reproduction: homothallic. ITS barcode: GU981612 (alternative markers: *BenA* = GU981663; *CaM* = MN969254; *RPB2* = KF296429).

***Penicillium ellipsoideosporum*** L. Wang & W.Y. Kong, Mycosystema 19: 463. 2000. [MB467721]. — Type: HMAS 71768. Ex-type: CBS 112493 = AS 3.5688. Infragen. class: subgen. *Aspergilloides*, sect. *Cinnamopurpurea*, ser. *Idahoensia*. Reproduction: asexual. ITS barcode: JX012224 (alternative markers: *BenA* = JQ965104; *CaM* = AY678559; *RPB2* = JN121427).

***Penicillium eremophilum*** (A.D. Hocking & Pitt) Houbraken *et al.*, Stud. Mycol. 86: 47. 2017. [MB820075]. Basionym: *Monascus eremophilus* A.D. Hocking & Pitt, Mycologia 80: 84. 1988. [MB132383]. — Type: FRR 3338. Ex-type: IMI 313774 = CBS 123361 = ATCC 62925. Infragen. class: subgen. *Aspergilloides*, sect. *Eremophila*, ser. *Eremophila*. Reproduction: asexual. ITS barcode: GU733341 (alternative markers: *BenA* = KY709170; *CaM* = KY611931; *RPB2* = KY611970).

***Penicillium erubescens*** D.B. Scott, Mycopathol. Mycol. Appl. 36: 14. 1968. [MB335726]. — Type: CBS 318.67. Ex-type: CBS 318.67 = ATCC 18544 = CSIR 1040 = FRR 814 = IFO 31734 = IMI 136204 = NRRL 6223. Infragen. class: subgen. *Aspergilloides*, sect. *Exilicaulis*, ser. *Erubescentia*. Reproduction: homothallic. ITS barcode: AF033464 (alternative markers: *BenA* = HQ646566; *CaM* = EU427281; *RPB2* = JN121490).

***Penicillium estinogenum*** A. Komatsu & S. Abe ex G. Sm., Trans. Brit. Mycol. Soc. 46: 335. 1963. [MB302397]. — Type: IMI 68241. Ex-type: CBS 329.59 = DTO 360-C8 = ATCC 18310 = CCRC 31557 = FAT1196 = FRR 3428 = IFO 6230 = IMI 068241 = QM 8149 = VKMF-274. Infragen. class: subgen. *Aspergilloides*, sect. *Gracilenta*, ser. *Estinogena*. Reproduction: asexual. ITS barcode: MN431388 (alternative markers: *BenA* = MN969381; *CaM* = MN969255; *RPB2* = n.a.).

***Penicillium euglaucum*** J.F.H. Beyma, Antonie van Leeuwenhoek 6: 269. 1940. [MB289081]. — Type: CBS 323.71. Ex-type: CBS 323.71 = IBT 30767. Infragen. class: subgen. *Aspergilloides*, sect. *Citrina*, ser. *Euglauca*. Reproduction: homothallic. ITS barcode: JN617699 (alternative markers: *BenA* = JN606856; *CaM* = JN606564; *RPB2* = JN606594).

***Penicillium excelsum*** Taniwaki *et al.*, PLoS ONE 10: e0143189, 8. 2015. [MB811066]. — Type: CCT 7772. Ex-type: DTO 357-D7 = ITAL 7572 = IBT 31516. Infragen. class: subgen. *Aspergilloides*, sect. *Lanata-Divaricata*, ser. *Rolfsiorum*. Reproduction: asexual. ITS barcode: KR815341 (alternative markers: *BenA* = KP691061; *CaM* = KR815342; *RPB2* = MN969166).

***Penicillium expansum*** Link, Mag. Ges. Naturf. Freunde Berlin 3: 16. 1809. [MB159382]. — Type: CBS H-7082. Ex-type: DTO 141-D5 = CBS 325.48 = ATCC 7861 = ATHUM2891 = CCRC 30566 = FRR 976 = IBT 3486 = IBT 5101 = IMI 039761 = IMI 039761ii = MUCL 29192 = NRRL 976 = VKMF-275. Infragen. class: subgen. *Penicillium*, sect. *Penicillium*, ser. *Penicillium*. Reproduction: asexual. ITS barcode: AY373912 (alternative markers: *BenA* = AY674400; *CaM* = DQ911134; *RPB2* = JF417427).

***Penicillium exsudans*** X.C. Wang & W.Y. Zhuang, Sci. Rep. 7: 8233, 7. 2017. [MB570336]. — Type: HMAS 248735. Ex-type: CGMCC 3.18412. Infragen. class: subgen. *Aspergilloides*, sect. *Sclerotiorum*, ser. *Sclerotiorum*. Reproduction: asexual. ITS barcode: KX885062 (alternative markers: *BenA* = KX885042; *CaM* = KX885052; *RPB2* = KX885033).

***Penicillium fagi*** C. Ramírez & A.T. Martínez, Mycopathologia 63: 57. 1978. [MB283595]. — Type: IJFM 3049. Ex-type: CBS 689.77 = CCMF-696 = IJFM 3049 = IMI 253806 = VKMF-2178. Infragen. class: subgen. *Aspergilloides*, sect. *Exilicaulis*, ser. *Corylophila*. Reproduction: asexual. ITS barcode: AF481124 (alternative markers: *BenA* = KJ834449; *CaM* = KP016784; *RPB2* = JN406540).

***Penicillium fellutanum*** Biourge, Cellule 33: 262. 1923. [MB264748]. — Type: IMI 39734. Ex-type: CBS 229.81 = CBS 326.48 = ATCC 10443 = FRR 746 = IFO 5761 = IMI 039734 = IMI 039734iii = NRRL 746 = QM 7554. Infragen. class: subgen. *Aspergilloides*, sect. *Charlesia*, ser. *Fellutana*. Reproduction: asexual. ITS barcode: AF033399 (alternative markers: *BenA* = KJ834450; *CaM* = AY741753; *RPB2* = JN121460).

***Penicillium fennelliae*** Stolk, Antonie van Leeuwenhoek 35: 261. 1969. [MB335728]. — Type: CBS 711.68. Ex-type: CBS 711.68 = ATCC 22050 = ATCC 52492 = FRR 521 = IHEM 4389 = IMI 151747 = MUCL 31322. Infragen. class: subgen. *Penicillium*, sect. *Brevicompacta*, ser. *Brevicompacta*. Reproduction: asexual. ITS barcode: JX313169 (alternative markers: *BenA* = MN969382; *CaM* = JX313151; *RPB2* = JN406536).

***Penicillium fernandesiae*** R.N. Barbosa *et al.*, Antonie van Leeuwenhoek 111: 1895. 2018. [MB822209]. — Type: URM 90490. Ex-type: CBS 142500 = URM 7600. Infragen. class: subgen. *Aspergilloides*, sect. *Sclerotiorum*, ser. *Sclerotiorum*. Reproduction: asexual. ITS barcode: MF278314 (alternative markers: *BenA* = MN969416; *CaM* = LT854649; *RPB2* = LT854654).

***Penicillium fimorum*** Frisvad & Houbraken, Persoonia 36: 309. 2016. [MB815871]. — Type: CBS H-22342. Ex-type: CBS 140575 = IBT 29495 = DTO 149-B8 = DTO 159-F1. Infragen. class: subgen. *Penicillium*, sect. *Robsamsonia*, ser. *Robsamsonia*. Reproduction: asexual. ITS barcode: KU904343 (alternative markers: *BenA* = KT698889; *CaM* = KT698898; *RPB2* = KT698908).

***Penicillium fimosum*** Guevara-Suarez *et al.*, Fungal Syst. Evol. 5: 58. 2019 [2020]. [MB822069]. — Type: CBS H-23185. Ex-type: CBS 142991 = FMR 15104. Infragen. class: subgen. *Penicillium*, sect. *Paradoxa*, ser. *Atramentosa*. Reproduction: asexual. ITS barcode: LT970836 (alternative markers: *BenA* = LT898273; *CaM* = LT970837; *RPB2* = n.a.).

***Penicillium flavigenum*** Frisvad & Samson, Mycol. Res. 101: 620. 1997. [MB437441]. — Type: CBS 419.89. Ex-type: CBS 419.89 = BT21526 = IBT 3091 = IBT V1035 = IMI 293207. Infragen. class: subgen. *Penicillium*, sect. *Chrysogena*, ser. *Chrysogena*. Reproduction: asexual. ITS barcode: JX997105 (alternative markers: *BenA* = AY495993; *CaM* = JX996281; *RPB2* = JN406551).

***Penicillium flaviroseum*** L. Cai & X.Z. Jiang, Cladistics 35: 528. 2018 [2019]. [MB818159]. — Type: HMAS 247727. Ex-type: CGMCC 3.18805 = NN072483 = CBS 144479. Infragen. class: subgen. *Aspergilloides*, sect. *Lanata-Divaricata*, ser. *Rolfsiorum*. Reproduction: asexual. ITS barcode: KY495032 (alternative markers: *BenA* = KY495141; *CaM* = MN969329; *RPB2* = KY495083).

***Penicillium flavisclerotiatum*** Visagie *et al.*, Houbraken & K. Jacobs, Stud. Mycol. 78: 419. 2014 [MB809963]. — Type: CBS H-21879. Ex-type: CBS 137750 = DTO 180-I8 = CV 100 = DAOM 241157. Infragen. class: subgen. *Aspergilloides*, sect. *Aspergilloides*, ser. *Pinetorum*. Reproduction: asexual. ITS barcode: KM189644 (alternative markers: *BenA* = KM088888; *CaM* = KM089275; *RPB2* = KM089662).

***Penicillium fluviserpens*** S.W. Peterson *et al.*, PLoS ONE 10: 0121987, 14. 2015. [MB807370]. — Type: BPI 881284. Ex-type: NRRL 35838 = IBT 29686. Infragen. class: subgen. *Aspergilloides*, sect. *Cinnamopurpurea*, ser. *Idahoensia*. Reproduction: asexual. ITS barcode: KF932961 (alternative markers: *BenA* = KF932929; *CaM* = KF932946; *RPB2* = KF933000).

***Penicillium formosanum*** H.M. Hsieh *et al.*, Trans. Mycol. Soc. Rep. China 2: 159. 1987. [MB126488]. — Type: PPEH 10001. Ex-type: DTO 206-F3 = CBS 211.92 = IBT 19748 = IBT 21527. Infragen. class: subgen. *Penicillium*, sect. *Formosana*, ser. *Formosana*. Reproduction: asexual. ITS barcode: KC411696 (alternative markers: *BenA* = AY674426; *CaM* = KU896835; *RPB2* = JN406615).

***Penicillium fortuitum*** Visagie & Seifert, Persoonia 41: 387. 2018. [MB827860]. — Type: DAOM 745786. Ex-type: DTO 313-A3 = DAOMC 251497. Infragen. class: subgen. *Aspergilloides*, sect. *Aspergilloides*, ser. *Fortuita*. Reproduction: asexual. ITS barcode: MF803942 (alternative markers: *BenA* = MF803836; *CaM* = MF803932; *RPB2* = MN969206).

***Penicillium fractum*** Udagawa, Trans. Mycol. Soc. Japan 9: 51. 1968. [MB335729]. — Type: CBS H-7086. Ex-type: CBS 124.68 = ATCC 18567 = FRR 3448 = IMI 136701 = NHL 6104 = NRRL 3448. Infragen. class: subgen. *Aspergilloides*, sect. *Inusitata*, ser. *Inusitata*. Reproduction: homothallic. ITS barcode: KC411674 (alternative markers: *BenA* = KJ834452; *CaM* = MN969256; *RPB2* = JN121441).

***Penicillium freii*** Frisvad & Samson, Stud. Mycol. 49: 28. 2004. [MB369274]. — Type: IMI 285513. Ex-type: DTO 158-D2 = CBS 476.84 = IBT 5137 = IMI 285513. Infragen. class: subgen. *Penicillium*, sect. *Fasciculata*, ser. *Viridicata*. Reproduction: asexual. ITS barcode: MN431389 (alternative markers: *BenA* = KU896813; *CaM* = KU896836; *RPB2* = KU904353).

***Penicillium frequentans*** Westling, Ark. Bot. 11: 133. 1911. [MB152118]. — Type: CBS 105.11. Ex-type: CBS 105.11. Infragen. class: subgen. *Aspergilloides*, sect. *Aspergilloides*, ser. *Glabra*. Reproduction: asexual. ITS barcode: KM189525 (alternative markers: *BenA* = KM088762; *CaM* = KM089147; *RPB2* = KM089534).

***Penicillium fructuariae-cellae*** Lorenzini *et al.*, Phytopathol. Medit. 58: 337. 2019. [MB831228]. — Type: ITEM 18276. Ex-type: CBS 145110. Infragen. class: subgen. *Aspergilloides*, sect. *Lanata-Divaricata*, ser. *Rolfsiorum*. Reproduction: asexual. ITS barcode: MK039434 (alternative markers: *BenA* = KU554679; *CaM* = MK045337; *RPB2* = n.a.).

***Penicillium fundyense*** Visagie *et al.*, Persoonia 36: 265. 2016. [MB815776]. — Type: DAOM 695767. Ex-type: DAOMC 250519 = CBS 140980 = DTO 410-D5 = NBBR-2-3 = W466 = KAS 2174. Infragen. class: subgen. *Aspergilloides*, sect. *Exilicaulis*, ser. *Citreonigra*. Reproduction: asexual. ITS barcode: KT887853 (alternative markers: *BenA* = KT887814; *CaM* = KT887775; *RPB2* = MN969176).

***Penicillium fuscum*** (Sopp) Biourge, Cellule 33: 103. 1923. [MB289082]. Basionym: *Citromyces fuscus* Sopp, Skr. Vidensk.-Selsk. Christiana Math.-Nat. Kl. 11: 120. 1912. [MB178643]. — Type: WIS WSF 15-C. Ex-type: CBS 295.62 = ATCC 14770 = CCRC 31517 = DSM2438 = IFO 7743 = IMI 094209 = MUCL 31196 = NRRL 3008 = WSF15c. Infragen. class: subgen. *Aspergilloides*, sect. *Aspergilloides*, ser. *Pinetorum*. Reproduction: homothallic (Stolk and Samson, 1983, Houbraken et al., 2014b). ITS barcode: AF033411 (alternative markers: *BenA* = GQ367513; *CaM* = GQ367539; *RPB2* = JN121483).

***Penicillium fusisporum*** L. Wang, PLoS ONE 9: e101454, 2. 2014. [MB806119]. — Type: HMAS 244961. Ex-type: CBS 137463 = NRRL 62805 = AS 3.15338. Infragen. class: subgen. *Aspergilloides*, sect. *Aspergilloides*, ser. *Thomiorum*. Reproduction: asexual. ITS barcode: KF769424 (alternative markers: *BenA* = KF769400; *CaM* = KF769413; *RPB2* = MN969117).

***Penicillium gallaicum*** C. Ramírez *et al.*, Mycopathologia 72: 30. 1980. [MB113021]. — Type: CBS H-7464. Ex-type: CBS 167.81 = ATCC 42232 = IJFM 5597. Infragen. class: subgen. *Aspergilloides*, sect. *Citrina*, ser. *Gallaica*. Reproduction: asexual. ITS barcode: JN617690 (alternative markers: *BenA* = JN606837; *CaM* = JN606548; *RPB2* = JN606609).

***Penicillium georgiense*** S.W. Peterson & B.W. Horn, Mycologia 101: 79. 2009. [MB509290]. — Type: BPI 877332. Ex-type: CBS 132826 = NRRL 35509 = DTO 194-D3. Infragen. class: subgen. *Aspergilloides*, sect. *Ramigena*, ser. *Georgiensia*. Reproduction: asexual. ITS barcode: EF422852 (alternative markers: *BenA* = EF506223; *CaM* = EF506239; *RPB2* = KM089734).

***Penicillium geumsanense*** Hyang B. Lee *et al.*, Fungal Diversity 96: 101. 2019. [MB555412]. — Type: CNUFC-GEU2229-1. Ex-type: CNUFC-GEU2229-1. Infragen. class: subgen. *Penicillium*, sect. *Robsamsonia*, ser. *Glandicolarum*. Reproduction: asexual. ITS barcode: n.a. (alternative markers: *BenA* = MK481059; *CaM* = MK481062; *RPB2* = MK481055).

***Penicillium glabrum*** (Wehmer) Westling, Ark. Bot. 11: 131. 1911. [MB120545]. Basionym: *Citromyces glaber* Wehmer, Beitr. Einh. Pilze 1: 24. 1893. [MB178959]. — Type: IMI 91944. Ex-type: CBS 125543 = IBT 22658 = IMI 91944. Infragen. class: subgen. *Aspergilloides*, sect. *Aspergilloides*, ser. *Glabra*. Reproduction: asexual. ITS barcode: GU981567 (alternative markers: *BenA* = GU981619; *CaM* = KM089152; *RPB2* = JF417447).

***Penicillium gladioli*** L. McCulloch & Thom, Science 67: 217. 1928. [MB266048]. — Type: IMI 34911. Ex-type: CBS 332.48 = ATCC 10448 = FRR 939 = IBT 14772 = IMI 034911 = IMI 034911ii = LCP 89.202 = MUCL 29174 = NRRL 939 = QM 1955 = VKMF-2088. Infragen. class: subgen. *Penicillium*, sect. *Fasciculata*, ser. *Gladioli*. Reproduction: asexual. ITS barcode: AF033480 (alternative markers: *BenA* = AY674287; *CaM* = KU896837; *RPB2* = MN969118).

***Penicillium glandicola*** (Oudem.) Seifert & Samson, Adv. Pen. Asp. Syst.: 147. 1986 [1985]. [MB114761]. Basionym: *Coremium glandicola* Oudem., Ned. Kruidk. Arch. 2: 918. 1903. [MB240065]. — Type: Netherlands, Valkenburg, Jul 1901, Rick in herb. Oudemans (L); CBS 498.75 (epitype). Ex-epitype: CBS 498.75 = IBT 21529 = IMI 154241. Infragen. class: subgen. *Penicillium*, sect. *Robsamsonia*, ser. *Glandicolarum*. Reproduction: asexual. ITS barcode: AB479308 (alternative markers: *BenA* = KU896814; *CaM* = KU896838; *RPB2* = KU904354).

***Penicillium glaucoalbidum*** (Desmazières) Houbraken & Samson, Stud. Mycol. 70: 47. 2011. [MB561965]. Basionym: *Sclerotium glaucoalbidum* Desm., Ann. Sci. Nat., Bot., sér. 3, 16: 329. 1851. [MB212120]. — Type: unknown. Ex-type: n.a. Infragen. class: subgen. *Aspergilloides*, sect. *Thysanophora*, ser. *Thysanophora*. Reproduction: asexual. ITS barcode: n.a. (alternative markers: *BenA* = n.a.; *CaM* = n.a.; *RPB2* = n.a.).

***Penicillium glaucoroseum*** Demelius, Verh. Zool.-Bot. Ges. Wien 72: 72. 1923. [MB158423]. — Type: Fig. 3. (Demelius, Verh. Zool.-Bot. Ges. Wien 72: 73, 1923 (1922) (lectotype, Visagie *et al.* 2015); CBS H-22050 (epitype). Ex-epitype: DTO 225-E8 = CBS 138908 = NRRL 908 (authentic acc. Raper & Thom 1949). Infragen. class: subgen. *Aspergilloides*, sect. *Lanata-Divaricata*, ser. *Janthinella*. Reproduction: asexual. ITS barcode: MN431390 (alternative markers: *BenA* = MN969383; *CaM* = MN969257; *RPB2* = MN969119).

***Penicillium globosum*** L. Cai *et al.*, Cladistics 35: 529. 2018 [2019]. [MB818149]. — Type: HMAS 247726. Ex-type: CGMCC 3.18800 = NN072354 = CBS 144639. Infragen. class: subgen. *Aspergilloides*, sect. *Lanata-Divaricata*, ser. *Simplicissima*. Reproduction: asexual. ITS barcode: KY495014 (alternative markers: *BenA* = KY495123; *CaM* = MN969330; *RPB2* = KY495067).

***Penicillium godlewskii*** K.W. Zaleski, Bull. Int. Acad. Polon. Sci., Sér. B., Sci. Nat. 1927: 466. 1927. [MB266206]. — Type: CBS 215.28 (lectotype, Houbraken *et al.* 2011a). Ex-type: CBS 215.28 = DTO 022-E2 = ATCC 10449 = ATCC 48714 = FRR 2111 = IFO 7724 = IMI 040591 = MUCL 29243 = NRRL 2111 = QM 7566 = VKMF-1826. Infragen. class: subgen. *Aspergilloides*, sect. *Citrina*, ser. *Westlingiorum*. Reproduction: asexual. ITS barcode: JN617692 (alternative markers: *BenA* = JN606768; *CaM* = MN969258; *RPB2* = JN606626).

***Penicillium goetzii*** J. Rogers *et al.*, Persoonia 29: 92. 2012. [MB801876]. — Type: CBS H-21061. Ex-type: DTO 088-G6 = CBS 285.73 = IBT 30199. Infragen. class: subgen. *Penicillium*, sect. *Chrysogena*, ser. *Goetziorum*. Reproduction: asexual. ITS barcode: JX997091 (alternative markers: *BenA* = KU896815; *CaM* = JX996971; *RPB2* = JX996716).

***Penicillium gorlenkoanum*** Baghd., Novosti Sist. Nizsh. Rast. 5: 97. 1968. [MB335731]. — Type: CBS H-7490. Ex-type: CBS 408.69 = DTO 023-A5 = DTO 034-E3 = FRR 511 = IMI 140339 = VKMF-1079. Infragen. class: subgen. *Aspergilloides*, sect. *Citrina*, ser. *Citrina*. Reproduction: asexual. ITS barcode: GU944581 (alternative markers: *BenA* = GU944520; *CaM* = MN969259; *RPB2* = JN606601).

***Penicillium gracilentum*** Udagawa & Y. Horie, Trans. Mycol. Soc. Japan 14: 373. 1973. [MB319272]. — Type: NHL 6452. Ex-type: CBS 599.73 = ATCC 28047 = ATCC 48258 = FRR 1557 = IMI 216900 = NHL 6452 = DTO 095-D8. Infragen. class: subgen. *Aspergilloides*, sect. *Gracilenta*, ser. *Gracilenta*. Reproduction: homothallic. ITS barcode: KC411768 (alternative markers: *BenA* = KJ834453; *CaM* = MN969260; *RPB2* = JN121537).

***Penicillium grancanariae*** C. Ramírez *et al.*, Mycopathologia 66: 79. 1978. [MB319273]. — Type: IJFM 3745. Ex-type: CBS 687.77 = IJFM 3745 = IMI 253783. Infragen. class: subgen. *Aspergilloides*, sect. *Aspergilloides*, ser. *Spinulosa*. Reproduction: asexual. ITS barcode: KM189529 (alternative markers: *BenA* = KM088766; *CaM* = KM089151; *RPB2* = KM089538).

***Penicillium granulatum*** Bainier, Bull. Soc. Mycol. Fr. 21: 126. 1905. [MB174620]. — Type: CBS 333.48. Ex-type: CBS 333.48 = DTO 246-F5 = ATCC 10450 = FRR 2036 = IBT 6592 = IMI 040220 = MUCL 15621 = NRRL 2036 = QM 6868. Infragen. class: subgen. *Penicillium*, sect. *Robsamsonia*, ser. *Glandicolarum*. Reproduction: asexual. ITS barcode: DQ339565 (alternative markers: *BenA* = MT478038.; *CaM* = MT478036; *RPB2* = MT478035).

***Penicillium gravinicasei*** S.W. Peterson *et al.*, Int. J. Food Microbiol. 282: 67. 2018. [MB823510]. — Type: BPI 910534. Ex-type: ITEM 17411 = NRRL 66733. Infragen. class: subgen. *Aspergilloides*, sect. *Cinnamopurpurea*, ser. *Cinnamopurpurea*. Reproduction: asexual. ITS barcode: MG600580 (alternative markers: *BenA* = MG600565; *CaM* = MG600570; *RPB2* = MG600575).

***Penicillium grevilleicola*** Houbraken & Quaedvlieg, Stud. Mycol. 78: 423. 2014. [MB809964]. — Type: CBS H-21871. Ex-type: CBS 137775 = DTO 174-E6. Infragen. class: subgen. *Aspergilloides*, sect. *Aspergilloides*, ser. *Thomiorum*. Reproduction: asexual. ITS barcode: KM189630 (alternative markers: *BenA* = KM088874; *CaM* = KM089261; *RPB2* = KM089648).

***Penicillium griseoazureum*** C. Moreau & M. Moreau ex C. Ramírez, Manual and Atlas of the Penicillia: 61, 1982; *Penicillium griseo-azureum* C. Moreau & M. Moreau, Revue Mycol. 6: 59, 1941 (*nom*. *inval*., Art. 36.1). [MB115800]. — type: CBS 162.42 (holotype). ex-type: CBS 162.42 = FRR 1361. Infragen. class: subgen. *Penicillium*, sect. *Canescentia*, ser. *Canescentia*. Reproduction: asexual. ITS barcode: KC411679 (alternative markers: *BenA* = KP016919; *CaM* = KP016823; *RPB2* = KP016852).

***Penicillium griseoflavum*** L. Cai & X.Z. Jiang, Cladistics 35: 531. 2018 [2019]. [MB818151]. — Type: HMAS 247729. Ex-type: CGMCC 3.18799 = NN072331. Infragen. class: subgen. *Aspergilloides*, sect. *Lanata-Divaricata*, ser. *Simplicissima*. Reproduction: asexual. ITS barcode: KY495011 (alternative markers: *BenA* = KY495120; *CaM* = MN969331; *RPB2* = KY495064).

***Penicillium griseofulvum*** Dierckx, Ann. Soc. Sci. Bruxelles 25: 88. 1901. [MB120566]. — Type: IMI 75832. Ex-type: DTO 072-A5 = CBS 185.27 = ATCC 11885 = ATHUM2893 = CECT 2605 = DSM896 = IBT 6740 = IFO 7640 = IFO 7641 = IMI 075832 = IMI 075832ii = LCP 79.3245 = LSHBP 68 = MUCL 28643 = NRRL 2152 = NRRL 2300 = QM 6902 = VKMF-286. Infragen. class: subgen. *Penicillium*, sect. *Robsamsonia*, ser. *Urticicola*. Reproduction: asexual. ITS barcode: AF033468 (alternative markers: *BenA* = JF909942; *CaM* = KT900574; *RPB2* = JN121449).

***Penicillium griseolum*** G. Sm., Trans. Brit. Mycol. Soc. 40: 485. 1957. [MB302401]. — Type: IMI 071626. Ex-type: CBS 277.58 = ATCC 18239 = FRR 2671 = IFO 8175 = IMI 071626 = LSHBB323 = NRRL 2671 = QM 7523. Infragen. class: subgen. *Aspergilloides*, sect. *Griseola*, ser. *Griseola*. Reproduction: asexual. ITS barcode: EF422848 (alternative markers: *BenA* = EF506213; *CaM* = EF506232; *RPB2* = JN121480).

***Penicillium griseopurpureum*** G. Sm., Trans. Brit. Mycol. Soc. 48: 275. 1965. [MB335732]. — Type: IMI 96157. Ex-type: CBS 406.65 = DTO 383-E5 = ATCC 22353 = FRR 3429 = IFO 9147 = IMI 096157. Infragen. class: subgen. *Aspergilloides*, sect. *Lanata-Divaricata*, ser. *Dalearum*. Reproduction: asexual. ITS barcode: KF296408 (alternative markers: *BenA* = KF296467; *CaM* = MN969261; *RPB2* = KF296431).

***Penicillium guaibinense*** J.P. Andrade *et al.*, Persoonia 41: 389. 2018. [MB827182]. — Type: HURB 18573. Ex-type: CCDCA 11512 = 23EM8. Infragen. class: subgen. *Aspergilloides*, sect. *Lanata-Divaricata*, ser. *Dalearum*. Reproduction: asexual. ITS barcode: MH674389 (alternative markers: *BenA* = MH674391; *CaM* = MH674393; *RPB2* = n.a.).

***Penicillium guanacastense*** K.G. Rivera *et al.*, Mycotaxon 119: 324. 2011. [MB563044]. — Type: DAOM 239912. Ex-type: CCFC 239912. Infragen. class: subgen. *Aspergilloides*, sect. *Sclerotiorum*, ser. *Sclerotiorum*. Reproduction: asexual. ITS barcode: JN626098 (alternative markers: *BenA* = JN625967; *CaM* = JN626010; *RPB2* = KX961295).

***Penicillium guangxiense*** L. Cai & X.Z. Jiang, Cladistics 35: 533. 2018 [2019]. [MB818150]. — Type: HMAS 247737. Ex-type: CGMCC 3.18793 = NN044175 = CBS 144526. Infragen. class: subgen. *Aspergilloides*, sect. *Lanata-Divaricata*, ser. *Simplicissima*. Reproduction: asexual. ITS barcode: KY494986 (alternative markers: *BenA* = KY495095; *CaM* = MN969332; *RPB2* = KY495045).

***Penicillium guttulosum*** J.C. Gilman & E.V. Abbott, Iowa St. Coll. J. Sci. 1: 298. 1927. [MB266689]. — Type: Iowa St. Coll. J. Sci. 1: 298, 1927 Fig. 33 (lectotype), Visagie *et al.* 2016c; CBS H-22566 (epitype). Ex-epitype: NRRL 907 = ATCC 48734 = FRR 907 = Thom 4894.16. Infragen. class: subgen. *Aspergilloides*, sect. *Exilicaulis*, ser. *Erubescentia*. Reproduction: asexual. ITS barcode: HQ646592 (alternative markers: *BenA* = HQ646576; *CaM* = HQ646587; *RPB2* = MG386247).

***Penicillium hainanense*** L. Cai & X.Z. Jiang, Cladistics 35: 534. 2018 [2019]. [MB818156]. — Type: HMAS 247730. Ex-type: CGMCC 3.18798 = NN072329 = CBS 144527. Infragen. class: subgen. *Aspergilloides*, sect. *Lanata-Divaricata*, ser. *Rolfsiorum*. Reproduction: asexual. ITS barcode: KY495009 (alternative markers: *BenA* = KY495118; *CaM* = MN969333; *RPB2* = KY495062).

***Penicillium halotolerans*** Frisvad *et al.*, Persoonia 29: 92. 2012. [MB801875]. — Type: CBS H-21060. Ex-type: DTO 148-H9 = CBS 131537 = IBT 4315. Infragen. class: subgen. *Penicillium*, sect. *Chrysogena*, ser. *Chrysogena*. Reproduction: asexual. ITS barcode: JX997005 (alternative markers: *BenA* = JX996816; *CaM* = JX996935; *RPB2* = JX996680).

***Penicillium hemitrachum*** Visagie & K. Jacobs, IMA Fungus 7: 99. 2016. [MB811004]. — Type: CBS H-22042. Ex-type: CBS 139134 = DAOMC 241098 = DTO 180-D8 = CV 2845. Infragen. class: subgen. *Aspergilloides*, sect. *Exilicaulis*, ser. *Lapidosa*. Reproduction: asexual. ITS barcode: FJ231003 (alternative markers: *BenA* = JX141048; *CaM* = JX157526; *RPB2* = KP064642).

***Penicillium hennebertii*** Houbraken & Samson, Stud. Mycol. 70: 47. 2011. [MB561964]. Replaced synonym: *Thysanophora canadensis* Stolk & Hennebert, Persoonia 5: 189. 1968. [MB340084]. — Type: CBS H-7854. Ex-type: CBS 334.68 = ATCC 18741 = IMI 137644 = MUCL 21216 = VKMF-2999. Infragen. class: subgen. *Aspergilloides*, sect. *Thysanophora*, ser. *Thysanophora*. Reproduction: asexual. ITS barcode: KJ834507 (alternative markers: *BenA* = KJ834454; *CaM* = MN969262; *RPB2* = JN121493).

***Penicillium hermansii*** Houbraken *et al.*, Mycol. Prog. 18: 232. 2018. [MB823949]. — Type: CBS H-21028. Ex-type: CBS 124296 = DTO 079-D5. Infragen. class: subgen. *Aspergilloides*, sect. *Exilicaulis*, ser. *Erubescentia*. Reproduction: asexual. ITS barcode: MG333472 (alternative markers: *BenA* = MG386214; *CaM* = MG386229; *RPB2* = MG386242).

***Penicillium herquei*** Bainier & Sartory, Bull. Soc. Mycol. France 28: 121. 1912. [MB536431]. — Type: IMI 28809. Ex-type: CBS 336.48 = NRRL 1040 = ATCC 10118 = BIOURGE 452 = FRR 1040 = IFO 31747 = IMI 28809 = MUCL 29213 = NCTC 1721 = QM 1926 = Thom 4640.447. Infragen. class: subgen. *Aspergilloides*, sect. *Sclerotiorum*, ser. *Herqueorum*. Reproduction: asexual. ITS barcode: JN626101 (alternative markers: *BenA* = JN625970; *CaM* = JN626013; *RPB2* = JN121494).

***Penicillium heteromorphum*** H.Z. Kong & Z.T. Qi, Mycosystema 1: 107. 1988. [MB135444]. — Type: CBS 226.89. Ex-type: CBS 226.89. Infragen. class: subgen. *Aspergilloides*, sect. *Exilicaulis*, ser. *Restricta*. Reproduction: asexual. ITS barcode: KC411702 (alternative markers: *BenA* = KJ834455; *CaM* = KP016786; *RPB2* = JN406605).

***Penicillium hetheringtonii*** Houbraken *et al.*, Fungal Diversity 44: 125. 2010. [MB518292]. — Type: CBS 122392. Ex -type: CBS 122392 = DTO 005-H9 = IBT 29057. Infragen. class: subgen. *Aspergilloides*, sect. *Citrina*, ser. *Citrina*. Reproduction: asexual. ITS barcode: GU944558 (alternative markers: *BenA* = GU944538; *CaM* = MN969263; *RPB2* = JN606606).

***Penicillium hirayamae*** Udagawa, J. Agric. Soc. Tokyo 5: 6. 1959. [MB302402]. — Type: IMI 78255. Ex-type: CBS 229.60 = ATCC 18312 = IFO 6435 = IMI 078255 = IMI 078255ii = NHL 6046 = NRRL 143 = QM 7885. Infragen. class: subgen. *Aspergilloides*, sect. *Sclerotiorum*, ser. *Sclerotiorum*. Reproduction: homothallic (Scott & Stolk 1967). ITS barcode: JN626095 (alternative markers: *BenA* = JN625955; *CaM* = JN626003; *RPB2* = JN121459).

***Penicillium hirsutum*** Dierckx, Ann. Soc. Sci. Bruxelles 25: 89. 1901. [MB152720]. — Type: IMI 40213. Ex-type: CBS 135.41 = ATCC 10429 = FRR 2032 = IBT 21531 = IFO 6092 = IMI 040213 = MUCL 15622 = NRRL 2032. Infragen. class: subgen. *Penicillium*, sect. *Fasciculata*, ser. *Corymbifera*. Reproduction: asexual. ITS barcode: AY373918 (alternative markers: *BenA* = MN969384; *CaM* = KU896840; *RPB2* = JN406629).

***Penicillium hispanicum*** C. Ramírez *et al.*, Mycopathologia 66: 77. 1978. [MB319274]. — Type: IJFM 3223. Ex-type: CBS 691.77 = ATCC 38667 = DSM2416 = IJFM 3223 = IMI 253785 = VKMF-2179. Infragen. class: subgen. *Aspergilloides*, sect. *Ramigena*, ser. *Ramigena*. Reproduction: asexual. ITS barcode: JX841247 (alternative markers: *BenA* = KJ834456; *CaM* = MN969264; *RPB2* = JN406539).

***Penicillium hoeksii*** Houbraken, Stud. Mycol. 78: 423. 2014. [MB809965]. — Type: CBS H-21860. Ex-type: CBS 137776 = DTO 192-H4. Infragen. class: subgen. *Aspergilloides*, sect. *Aspergilloides*, ser. *Hoeksiorum*. Reproduction: asexual. ITS barcode: KM189707 (alternative markers: *BenA* = KM088954; *CaM* = KM089341; *RPB2* = KM089728).

***Penicillium hordei*** Stolk, Antonie van Leeuwenhoek 35: 270. 1969. [MB335734]. — Type: CBS 701.68. Ex-type: CBS 701.68 = DTO 303-B6 = ATCC 22053 = CECT 2290 = FRR 815 = IBT 17804 = IBT 6980 = IMI 151748 = MUCL 39559. Infragen. class: subgen. *Penicillium*, sect. *Fasciculata*, ser. *Corymbifera*. Reproduction: asexual. ITS barcode: MN431391 (alternative markers: *BenA* = MN969385; *CaM* = KU896841; *RPB2* = KU904355).

***Penicillium ibericum*** Guevara-Suarez *et al.*, Fungal Syst. Evol. 5: 58. 2019 [2020]. [MB822070]. — Type: CBS H-23186. Ex-type: CBS 142992 = FMR 15040. Infragen. class: subgen. *Penicillium*, sect. *Paradoxa*, ser. *Atramentosa*. Reproduction: asexual. ITS barcode: LT899782 (alternative markers: *BenA* = LT898285; *CaM* = LT899766; *RPB2* = LT899800).

***Penicillium idahoense*** Paden, Mycopathol. Mycol. Appl. 43: 259. 1971. [MB319275]. — Type: UVIC JWP 66-32. Ex-type: CBS 341.68 = NRRL 5274 = ATCC 22055 = FRR 881 = IMI 148393. Infragen. class: subgen. *Aspergilloides*, sect. *Cinnamopurpurea*, ser. *Idahoensia*. Reproduction: homothallic. ITS barcode: KC411747 (alternative markers: *BenA* = EF626953; *CaM* = EF626954; *RPB2* = JN121499).

***Penicillium improvisum*** Visagie *et al.*, Persoonia 36: 256. 2016. [MB815769]. — Type: DAOM 695768. Ex-type: DAOMC 250547 = CBS 140994 = DTO 410-D9 = KAS 2386 = W 156. Infragen. class: subgen. *Aspergilloides*, sect. *Aspergilloides*, ser. *Improvisa*. Reproduction: asexual. ITS barcode: KT887867 (alternative markers: *BenA* = KT887828; *CaM* = KT887789; *RPB2* = MN969169).

***Penicillium incoloratum*** L.Q. Huang & Z.T. Qi, Acta Mycol. Sin. 13: 264. 1994. [MB363421]. — Type: HMAS 65949. Ex-type: CBS 101753 = AS 3.4672. Infragen. class: subgen. *Aspergilloides*, sect. *Cinnamopurpurea*, ser. *Nodula*. Reproduction: asexual. ITS barcode: KJ834508 (alternative markers: *BenA* = KJ834457; *CaM* = KJ866984; *RPB2* = JN406651).

***Penicillium indicum*** D.K. Sandhu & R.S. Sandhu, Canad. J. Bot. 41: 1273. 1963. [MB335735]. — Type: New Delhi-6, No. Pe 1602; CBS H-7476 (isotype). Ex-type: CBS 115.63 = NRRL 3387 = ATCC 18324 = FRR 3387 = IFO 31744 = IMI 166620. Infragen. class: subgen. *Aspergilloides*, sect. *Charlesia*, ser. *Indica*. Reproduction: asexual. ITS barcode: AY742699 (alternative markers: *BenA* = EU427263; *CaM* = AY741744; *RPB2* = JN406640).

***Penicillium infra-aurantiacum*** Visagie *et al.*, Stud. Mycol. 78: 426. 2014 [MB809966]. — Type: CBS H-21880. Ex-type: CBS 137747 = DTO 183-C3 = CV 1518 = DAOM 241145. Infragen. class: subgen. *Aspergilloides*, sect. *Aspergilloides*, ser. *Sublectatica*. Reproduction: asexual. ITS barcode: KM189684 (alternative markers: *BenA* = KM088930; *CaM* = KM089317; *RPB2* = KM089704).

***Penicillium infrabuccalum*** Visagie *et al.*, Persoonia 36: 275. 2016. [MB815782]. — Type: DAOM 695769. Ex-type: DAOMC 250537 = CBS 140983 = DTO 410-D6 = NBSM-6-2 = W 475 = KAS 2181. Infragen. class: subgen. *Aspergilloides*, sect. *Lanata-Divaricata*, ser. *Simplicissima*. Reproduction: asexual. ITS barcode: KT887856 (alternative markers: *BenA* = KT887817; *CaM* = KT887778; *RPB2* = MN969181).

***Penicillium infrapurpureum*** Visagie *et al.*, Stud. Mycol. 78: 116. 2014. [MB809181]. — Type: CBS H-21801. Ex-type: CBS 138219 = DTO 235-F6. Infragen. class: subgen. *Aspergilloides*, sect. *Cinnamopurpurea*, ser. *Idahoensia*. Reproduction: asexual. ITS barcode: KJ775679 (alternative markers: *BenA* = KJ775172; *CaM* = KJ775406; *RPB2* = MN969120).

***Penicillium inusitatum*** D.B. Scott, Mycopathol. Mycol. Appl. 36: 20. 1968. [MB335736]. — Type: CBS 351.67. Ex-type: CBS 351.67 = ATCC 18622 = CSIR 1096 = FRR 1163 = IMI 136214 = NRRL 5810. Infragen. class: subgen. *Aspergilloides*, sect. *Inusitata*, ser. *Inusitata*. Reproduction: homothallic. ITS barcode: AF033431 (alternative markers: *BenA* = KJ834458; *CaM* = MN969265; *RPB2* = JN121503).

***Penicillium isariiforme*** Stolk & J.A. Mey., Trans. Brit. Mycol. Soc. 40: 187. 1957. [MB302403]. — Type: IMI 60371. Ex-type: CBS 247.56 = ATCC 18425 = CCRC 31699 = IFO 6393 = IHEM 4376 = IMI 060371 = LSHBBB308 = MUCL 31191 = MUCL 31323 = NRRL 2638 = QM 1897. Infragen. class: subgen. *Aspergilloides*, sect. *Ochrosalmonea*, ser. *Ochrosalmonea*. Reproduction: asexual. ITS barcode: AF454077 (alternative markers: *BenA* = KJ834459; *CaM* = MN969266; *RPB2* = JN121470).

***Penicillium italicum*** Wehmer, Hedwigia 33: 211. 1894. [MB162660]. — Type: CBS 339.48. Ex-type: CBS 339.48 = ATCC 10454 = DSM 2754 = FRR 983 = IBT 23029 = IMI 039760 = MUCL 15608 = NRRL 983 = QM 7572. Infragen. class: subgen. *Penicillium*, sect. *Penicillium*, ser. *Italica*. Reproduction: asexual. ITS barcode: KJ834509 (alternative markers: *BenA* = AY674398; *CaM* = DQ911135; *RPB2* = JN121496).

***Penicillium jacksonii*** K.G. Rivera *et al.*, Stud. Mycol. 70: 151. 2011. [MB563160]. — Type: DAOM 239937. Ex-type: CCFC 239937. Infragen. class: subgen. *Aspergilloides*, sect. *Sclerotiorum*, ser. *Sclerotiorum*. Reproduction: asexual. ITS barcode: JN686437 (alternative markers: *BenA* = JN686368; *CaM* = JN686391; *RPB2* = n.a.).

***Penicillium jamesonlandense*** Frisvad & Overy, Int. J. Syst. Evol. Microbiol. 56: 1435. 2006. [MB521421]. — Type: DAOM 234087. Ex-type: CBS 102888 = DAOM 234087 = IBT 21984 = IBT 24411. Infragen. class: subgen. *Penicillium*, sect. *Ramosum*, ser. *Lanosa*. Reproduction: asexual. ITS barcode: DQ267912 (alternative markers: *BenA* = DQ309448; *CaM* = KJ866985; *RPB2* = MN969121).

***Penicillium janczewskii*** K.W. Zaleski, Bull. Int. Acad. Polon. Sci., Sér. B., Sci. Nat. 1927: 488. 1927. [MB120703]. — Type: IMI 191499. Ex-type: CBS 221.28 = FRR 919 = IMI 191499 = NRRL 919. Infragen. class: subgen. *Penicillium*, sect. *Canescentia*, ser. *Canescentia*. Reproduction: asexual. ITS barcode: AY157487 (alternative markers: *BenA* = MN969386; *CaM* = MN969267; *RPB2* = JN406612).

***Penicillium janthinellum*** Biourge, Cellule 33: 258. 1923. [MB119134]. — Type: IMI 40238. Ex-type: CBS 340.48 = DTO 095-C3 = ATCC 10455 = IMI 040238 = NRRL 2016 = QM 6865. Infragen. class: subgen. *Aspergilloides*, sect. *Lanata-Divaricata*, ser. *Janthinella*. Reproduction: asexual. ITS barcode: GU981585 (alternative markers: *BenA* = GU981625; *CaM* = MN969268; *RPB2* = JN121497).

***Penicillium javanicum*** J.F.H. Beyma, Verh. Kon. Ned. Akad. Wetensch., Afd. Natuurk. 26: 17. 1929. [MB268394]. — Type: IMI 39733. Ex-type: CBS 341.48 = DTO 097-F9 = ATCC 9099 = CSIR 831 = FRR 707 = IFO 31735 = IMI 039733 = MUCL 29099 = NRRL 707 = QM 1876. Infragen. class: subgen. *Aspergilloides*, sect. *Lanata-Divaricata*, ser. *Janthinella*. Reproduction: homothallic. ITS barcode: GU981613 (alternative markers: *BenA* = GU981657; *CaM* = MN969269; *RPB2* = JN121498).

***Penicillium jejuense*** M.S. Park & Y.W. Lim, Mycologia 107: 212. 2015. [MB808392]. — Type: SFC 20140101-M756. Ex-type: CBS 138646 = KCTC 46212. Infragen. class: subgen. *Aspergilloides*, sect. *Aspergilloides*, ser. *Thomiorum*. Reproduction: asexual. ITS barcode: KF818464 (alternative markers: *BenA* = KF818461; *CaM* = KF818470; *RPB2* = KF818467).

***Penicillium jensenii*** K.W. Zaleski, Bull. Int. Acad. Polon. Sci., Sér. B., Sci. Nat. 1927: 494. 1927. [MB120708]. — Type: IMI 39768. Ex-type: CBS 327.59 = ATCC 18317 = FRR 909 = IFO 5764 = IMI 039768 = LCP 89.1389 = NRRL 909 = QM 7587. Infragen. class: subgen. *Penicillium*, sect. *Canescentia*, ser. *Canescentia*. Reproduction: asexual. ITS barcode: AY443470 (alternative markers: *BenA* = JX140954; *CaM* = AY443490; *RPB2* = JN406614).

***Penicillium jianfenglingense*** L. Cai & X.Z. Jiang, Cladistics 35: 535. 2018 [2019]. [MB818161]. — Type: HMAS 247731. Ex-type: CGMCC 3.18802 = NN072384 = CBS 144640. Infragen. class: subgen. *Aspergilloides*, sect. *Lanata-Divaricata*, ser. *Dalearum*. Reproduction: asexual. ITS barcode: KY495016 (alternative markers: *BenA* = KY495125; *CaM* = MN969334; *RPB2* = KY495069).

***Penicillium jiangxiense*** H.Z. Kong & Z.Q. Liang, Mycosystema 22: 4. 2003. [MB489161]. — Type: HMAS 82540. Ex-type: AS 3.6521 = DTO 309-A7. Infragen. class: subgen. *Aspergilloides*, sect. *Cinnamopurpurea*, ser. *Jiangxiensia*. Reproduction: asexual. ITS barcode: KJ890411 (alternative markers: *BenA* = KJ890409; *CaM* = KJ890407; *RPB2* = MN969122).

***Penicillium johnkrugii*** K.G. Rivera *et al.*, Stud. Mycol. 70: 151. 2011. [MB563161]. — Type: DAOM 239943. Ex-type: CCFC 239943. Infragen. class: subgen. *Aspergilloides*, sect. *Sclerotiorum*, ser. *Sclerotiorum*. Reproduction: asexual. ITS barcode: JN686447 (alternative markers: *BenA* = JN686378; *CaM* = JN686401; *RPB2* = n.a.).

***Penicillium jugoslavicum*** C. Ramírez & Munt.-Cvetk., Mycopathologia 88: 65. 1984. [MB124173]. — Type: CBS 192.87. Ex-type: CBS 192.87 = IJFM 7785 = IMI 314508. Infragen. class: subgen. *Aspergilloides*, sect. *Sclerotiorum*, ser. *Adametziorum*. Reproduction: asexual. ITS barcode: KC773836 (alternative markers: *BenA* = KC773789; *CaM* = KC773815; *RPB2* = JN406618).

***Penicillium kananaskense*** Seifert *et al.*, Canad. J. Bot. 72: 20. 1994. [MB362160]. — Type: DAOM 216105. Ex-type: CBS 530.93 = ATCC 90282 = DAOM 216105 = IBT 11775 = IMI 356791. Infragen. class: subgen. *Aspergilloides*, sect. *Aspergilloides*, ser. *Livida*. Reproduction: asexual. ITS barcode: KM189780 (alternative markers: *BenA* = KM089030; *CaM* = KM089417; *RPB2* = KM089804).

***Penicillium katangense*** Stolk, Antonie van Leeuwenhoek 34: 42. 1968. [MB120725]. — Type: CBS 247.67. Ex-type: CBS 247.67 = ATCC 18388 = IMI 136206 = NRRL 5182. Infragen. class: subgen. *Aspergilloides*, sect. *Exilicaulis*, ser. *Restricta*. Reproduction: homothallic. ITS barcode: AF033458 (alternative markers: *BenA* = KP016757; *CaM* = KP016788; *RPB2* = KP064646).

***Penicillium kewense*** G. Sm., Trans. Brit. Mycol. Soc. 44: 42. 1961. [MB335740]. — Type: L.S.H.T.M. BB 400 (holotype); CBS H-7077 (isotype). Ex-type: CBS 344.61 = ATCC 18240 = FRR 3441 = IFO 8113 = IMI 086561 = LSHBBB400 = MUCL 2685 = NRRL 3332 = QM 7958. Infragen. class: subgen. *Penicillium*, sect. *Chrysogena*, ser. *Crustacea*. Reproduction: homothallic. ITS barcode: AF033466 (alternative markers: *BenA* = KU896816; *CaM* = JX996973; *RPB2* = JF417428).

***Penicillium kiamaense*** Houbraken & Pitt, Stud. Mycol. 78: 426. 2014. [MB809967]. — Type: CBS H-21857. Ex-type: CBS 137947 = FRR 6087 = DTO 056-I6. Infragen. class: subgen. *Aspergilloides*, sect. *Aspergilloides*, ser. *Kiamaensia*. Reproduction: asexual. ITS barcode: KM189506 (alternative markers: *BenA* = KM088743; *CaM* = KM089128; *RPB2* = KM089515).

***Penicillium kojigenum*** G. Sm., Trans. Brit. Mycol. Soc. 44: 43. 1961. [MB335741]. — Type: L.S.H.T.M. BB 39. Ex-type: CBS 345.61 = ATCC 18227 = CCRC 31515 = FRR 3442 = IFO 9581 = IMI 086562 = LSHBBB394 = MUCL 2457 = NRRL 3442 = QM 7957. Infragen. class: subgen. *Penicillium*, sect. *Ramosum*, ser. *Lanosa*. Reproduction: asexual. ITS barcode: AF033489 (alternative markers: *BenA* = KJ834463; *CaM* = KJ867011; *RPB2* = JN406564).

***Penicillium kongii*** L. Wang, Mycologia 105: 1549. 2013. [MB803185]. — Type: HMAS 244382. Ex-type: AS 3.15329. Infragen. class: subgen. *Penicillium*, sect. *Brevicompacta*, ser. *Brevicompacta*. Reproduction: asexual. ITS barcode: KC427191 (alternative markers: *BenA* = KC427171; *CaM* = KC427151; *RPB2* = n.a.).

***Penicillium koreense*** S.B. Hong *et al.*, J. Microbiol. Biotechnol 24: 1607. 2014. [MB808759]. — Type: KACC 47721. Ex-type: CBS 141338 = KACC 47721 = DTO 347-C1. Infragen. class: subgen. *Aspergilloides*, sect. *Lanata-Divaricata*, ser. *Janthinella*. Reproduction: asexual. ITS barcode: KJ801939 (alternative markers: *BenA* = KM000846; *CaM* = MN969317; *RPB2* = MN969159).

***Penicillium kurssanovii*** Chalab., Bot. Mater. Otd. Sporov. Rast. 6: 168. 1950. [MB274327]. — Type: unknown. Ex-type: CBS 625.67 = ATCC 18387 = FRR 3381 = IJFM 5045 = IMI 129965 = NRRL 3381 = VKMF-1244. Infragen. class: subgen. *Aspergilloides*, sect. *Exilicaulis*, ser. *Restricta*. Reproduction: asexual. ITS barcode: EF422849 (alternative markers: *BenA* = KP016758; *CaM* = KP016789; *RPB2* = KP064647).

***Penicillium labradorum*** Gibas *et al.*, Med. Mycol., doi.org/10.1093/mmy/myaa016. 2020. [MB831086]. — Type: CBS H-24321. Ex-type UTHSCSA DI19-20 = CBS 145775. Infragen. class: subgen. *Aspergilloides*, sect. *Exilicaulis*, ser. *Erubescentia*. Reproduction: asexual. ITS barcode: MK881918 (alternative markers: *BenA* = MK887898; *CaM* = MK887899; *RPB2* = MK887900).

***Penicillium laeve*** (K. Ando & Manoch) Houbraken & Samson, Stud. Mycol. 70: 47. 2011. [MB561960]. Basionym: *Torulomyces laevis* K. Ando & Manoch, Mycoscience 39: 317. 1998. [MB447110]. — Type: TNS-F-238517. Ex-type: CBS 136665 = KY 12727 = NBRC 109724 = DTO 270-G8. Infragen. class: subgen. *Aspergilloides*, sect. *Exilicaulis*, ser. *Erubescentia*. Reproduction: homothallic. ITS barcode: KF667369 (alternative markers: *BenA* = KF667365; *CaM* = KF667367; *RPB2* = KF667371).

***Penicillium laevigatum*** L. Cai *et al.*, Cladistics 35: 537. 2018 [2019]. [MB818154]. — Type: HMAS 247728. Ex-type: CGMCC 3.18801 = NN072364. Infragen. class: subgen. *Aspergilloides*, sect. *Lanata-Divaricata*, ser. *Simplicissima*. Reproduction: asexual. ITS barcode: KY495015 (alternative markers: *BenA* = KY495124; *CaM* = MN969335; *RPB2* = KY495068).

***Penicillium lagena*** (Delitsch) Stolk & Samson, Stud. Mycol. 23: 100. 1983. [MB109162]. Basionym: *Torulomyces lagena* Delitsch, Systematik der Schimmelpilze: 9. 1943. [MB340152]. — Type: Fig. 233 (Delitsch, Ergebnisse der theoretischen und angewandten Mikrobiologie: Band I: Systematik der Schimmelpilze. J. Neumann, Neudamm, Germany, Tafel 30, 1943, Visagie *et al.* 2016a, MBT203020 (lectotype); CBS 185.65 (epitype). Ex-type: CBS 185.65 = MUCL 8221 = JCM10149 = OAC10034 = DTO 077-I8. Infragen. class: subgen. *Aspergilloides*, sect. *Torulomyces*, ser. *Torulomyces*. Reproduction: asexual. ITS barcode: KF303665 (alternative markers: *BenA* = KF303619; *CaM* = KF303634; *RPB2* = JN121450).

***Penicillium lanosocoeruleum*** Thom, Penicillia: 322. 1930. [MB268949]. — Type: NRRL 888. Ex-type: CBS 215.30 = CBS 334.48 = ATCC 10459 = IFO 7761 = IMI 039818 = NRRL 888 = QM 6755 = VKMF-3089. Infragen. class: subgen. *Penicillium*, sect. *Chrysogena*, ser. *Aethiopica*. Reproduction: asexual. ITS barcode: JX997110 (alternative markers: *BenA* = KU896817; *CaM* = JX996967; *RPB2* = JX996723).

***Penicillium lanosum*** Westling, Ark. Bot. 11: 97. 1911. [MB178497]. — Type: IMI 40224. Ex-type: DTO 060-F7 = CBS 106.11 = ATCC 10458 = FRR 2009 = IFO 5851 = IFO 6099 = IMI 040224 = LSHBP 86 = MUCL 29232 = NRRL 2009 = QM 7591. Infragen. class: subgen. *Penicillium*, sect. *Ramosum*, ser. *Lanosa*. Reproduction: asexual. ITS barcode: DQ304540 (alternative markers: *BenA* = DQ285627; *CaM* = FJ530974; *RPB2* = KU904356).

***Penicillium lapidosum*** Raper & Fennell, Mycologia 40: 524, 1948. [MB289094]. — Type: IMI 39743. Ex-type: CBS 343.48 = ATCC 10462 = CCT4477 = IFO 6100 = IMI 039743 = NRRL 718 = QM 1928. Infragen. class: subgen. *Aspergilloides*, sect. *Exilicaulis*, ser. *Lapidosa*. Reproduction: homothallic (Scott & Stolk 1967). ITS barcode: MN431392 (alternative markers: *BenA* = KJ834465; *CaM* = FJ530984; *RPB2* = JN121500).

***Penicillium lassenii*** Paden, Mycopathol. Mycol. Appl. 43: 266. 1971. [MB319281]. — Type: UVIC JWP 69-26. Ex-type: CBS 277.70 = NRRL 5272 = ATCC 22054 = FRR 858 = IMI 148395 = DTO 095-D6. Infragen. class: subgen. *Aspergilloides*, sect. *Lasseniorum*, ser. *Lasseniorum*. Reproduction: homothallic. ITS barcode: KF303648 (alternative markers: *BenA* = KF303607; *CaM* = KF303629; *RPB2* = JN121481).

***Penicillium lemhiflumine*** S.W. Peterson *et al.*, PLoS ONE 10: 0121987, 4. 2015. [MB807371]. — Type: BPI 881287. Ex-type: NRRL 35843 = IBT 29684. Infragen. class: subgen. *Aspergilloides*, sect. *Cinnamopurpurea*, ser. *Idahoensia*. Reproduction: asexual. ITS barcode: KF932964 (alternative markers: *BenA* = KF932932; *CaM* = KF932949; *RPB2* = KF933003).

***Penicillium lenticrescens*** Visagie *et al.*, Stud. Mycol. 78: 123. 2014. [MB809184]. — Type: CBS H-21804. Ex-type: CBS 138215 = DTO 129-A8. Infragen. class: subgen. *Penicillium*, sect. *Ramosum*, ser. *Soppiorum*. Reproduction: asexual. ITS barcode: KJ775675 (alternative markers: *BenA* = KJ775168; *CaM* = KJ775404; *RPB2* = MN969123).

***Penicillium levitum*** Raper & Fennell, Mycologia 40: 511. 1948. [MB289096]. — Type: IMI 039735. Ex-type: CBS 345.48 = DTO 096-I7 = ATCC 10464 = IFO 6101 = IFO 8849 = IMI 039735 = NRRL 705 = QM 1877. Infragen. class: subgen. *Aspergilloides*, sect. *Lanata-Divaricata*, ser. *Janthinella*. Reproduction: homothallic. ITS barcode: GU981607 (alternative markers: *BenA* = GU981654; *CaM* = MN969270; *RPB2* = KF296432).

***Penicillium lilacinoechinulatum*** S. Abe ex G. Sm., Trans. Brit. Mycol. Soc. 46: 335. 1963. [MB120793]. — Type: IMI 068211. Ex-type: CBS 454.93 = ATCC 18309 = FAT 84 = FRR 3451 = IFO 6231 = IMI 068211 = QM 7289. Infragen. class: subgen. *Aspergilloides*, sect. *Sclerotiorum*, ser. *Adametziorum*. Reproduction: asexual. ITS barcode: AY157489 (alternative markers: *BenA* = KC773790; *CaM* = KC773816; *RPB2* = KX961293).

***Penicillium limosum*** S. Ueda, Mycoscience 36: 451. 1995. [MB415136]. — Type: CBM NEI-5220. Ex-type: CBS 339.97 = DTO 096-H8 = NEI5220. Infragen. class: subgen. *Aspergilloides*, sect. *Lanata-Divaricata*, ser. *Janthinella*. Reproduction: homothallic. ITS barcode: GU981568 (alternative markers: *BenA* = GU981621; *CaM* = MN969271; *RPB2* = KF296433).

***Penicillium lineolatum*** Udagawa & Y. Horie, Mycotaxon 5: 493. 1977. [MB319283]. — Type: NHL 2776. Ex-type: CBS 188.77 = DTO 097-E1 = NHL 2776. Infragen. class: subgen. *Aspergilloides*, sect. *Lanata-Divaricata*, ser. *Janthinella*. Reproduction: homothallic. ITS barcode: GU981579 (alternative markers: *BenA* = GU981620; *CaM* = MN969272; *RPB2* = KF296434).

***Penicillium lividum*** Westling, Ark. Bot. 11: 134. 1911. [MB178817]. — Type: IMI 39736. Ex-type: CBS 347.48 = ATCC 10102 = CCRC 31286 = DSM1180 = IFO 6102 = IMI 039736 = NRRL 754 = QM 1930 = VKMF-303. Infragen. class: subgen. *Aspergilloides*, sect. *Aspergilloides*, ser. *Livida*. Reproduction: asexual. ITS barcode: KM189582 (alternative markers: *BenA* = KM088825; *CaM* = KM089211; *RPB2* = KM089598).

***Penicillium longicatenatum*** Visagie *et al.*, Stud. Mycol. 78: 429. 2014. [MB809968]. — Type: CBS H-21875. Ex-type: CBS 137735 = DTO 180-D9 = CV 2847 = DAOM 241119. Infragen. class: subgen. *Aspergilloides*, sect. *Aspergilloides*, ser. *Longicatenata*. Reproduction: asexual. ITS barcode: KM189636 (alternative markers: *BenA* = KM088880; *CaM* = KM089267; *RPB2* = KM089654).

***Penicillium longisporum*** (W.B. Kend.) Houbraken & Samson, Stud. Mycol. 70: 47. 2011. [MB561966]. Basionym: *Thysanophora longispora* W.B. Kend., Canad. J. Bot. 39: 826. 1961. [MB340086]. — Type: DAOM 63073. Ex-type: CBS 354.62 = DAOM 63073 = MUCL 4168. Infragen. class: subgen. *Aspergilloides*, sect. *Thysanophora*, ser. *Thysanophora*. Reproduction: asexual. ITS barcode: n.a. (alternative markers: *BenA* = n.a.; *CaM* = n.a.; *RPB2* = n.a.).

***Penicillium ludwigii*** Udagawa, Trans. Mycol. Soc. Japan 10: 2. 1969. [MB335744]. — Type: NHL 6118. Ex-type: CBS 417.68 = DTO 094-D8 = DTO 264-I9 = FRR 559. Infragen. class: subgen. *Aspergilloides*, sect. *Lanata-Divaricata*, ser. *Janthinella*. Reproduction: homothallic. ITS barcode: KF296409 (alternative markers: *BenA* = KF296468; *CaM* = MN969273; *RPB2* = KF296435).

***Penicillium lunae*** Visagie & N. Yilmaz, Persoonia 42: 449. 2019. [MB830682]. — Type: PREM 62233. Ex-type: PPRI 25881 = CMV006E6. Infragen. class: subgen. *Aspergilloides*, sect. *Charlesia*, ser. *Indica*. Reproduction: asexual. ITS barcode: MK450725 (alternative markers: *BenA* = MK451088; *CaM* = MK451660; *RPB2* = MK450863).

***Penicillium lusitanum*** Gonçalves & Alves, Int. J. Syst. Evol. Microbiol. 69: 3020. 2019. [MB830331]. — Type: MUM-H 18.49. Ex-type: MUM 18.49 = CMG8. Infragen. class: subgen. *Penicillium*, sect. *Ramosum*, ser. *Soppiorum*. Reproduction: asexual. ITS barcode: MK702084 (alternative markers: *BenA* = MK702085; *CaM* = n.a.; *RPB2* = n.a.).

***Penicillium maclennaniae*** H.Y. Yip, Trans. Brit. Mycol. Soc. 77: 202. 1981. [MB112523]. — Type: DAR 35238. Ex-type: CBS 198.81 = DAR 35238. Infragen. class: subgen. *Aspergilloides*, sect. *Exilicaulis*, ser. *Lapidosa*. Reproduction: asexual. ITS barcode: KC411689 (alternative markers: *BenA* = KJ834468; *CaM* = KP016791; *RPB2* = KP064648).

***Penicillium macrosclerotiorum*** L. Wang *et al.*, Mycol. Res. 111: 1244. 2007. [MB492622]. — Type: HMAS 133177-1-4. Ex-type: CBS 116871 = AS 3.6581. Infragen. class: subgen. *Aspergilloides*, sect. *Gracilenta*, ser. *Macrosclerotiorum*. Reproduction: asexual. ITS barcode: KJ834511 (alternative markers: *BenA* = KJ834469; *CaM* = DQ911123; *RPB2* = JN121432).

***Penicillium madriti*** G. Sm., Trans. Brit. Mycol. Soc. 44: 44. 1961. [MB335747]. — Type: IMI 86563. Ex-type: CBS 347.61 = ATCC 18233 = CCRC 31672 = FRR 3452 = IFO 9148 = IMI 086563 = LSHBBB389 = MUCL 2456 = MUCL 31193 = NRRL 3452 = QM 7959. Infragen. class: subgen. *Penicillium*, sect. *Turbata*, ser. *Turbata*. Reproduction: asexual. ITS barcode: AF033482 (alternative markers: *BenA* = KJ834470; *CaM* = EU644076; *RPB2* = JN406561).

***Penicillium magnielliptisporum*** Visagie *et al.*, Stud. Mycol. 78: 127. 2014. [MB809186]. — Type: CBS H-21806. Ex-type: CBS 138225 = DTO 128-H8. Infragen. class: subgen. *Penicillium*, sect. *Paradoxa*, ser. *Atramentosa*. Reproduction: asexual. ITS barcode: KJ775686 (alternative markers: *BenA* = KJ775179; *CaM* = KJ775413; *RPB2* = MN969124).

***Penicillium malacaense*** C. Ramírez & A.T. Martínez, Mycopathologia 72: 186. 1980. [MB113025]. — Type: IJFM 7093. Ex-type: CBS 160.81 = NRRL 35754 = ATCC 42241 = IJFM 7093 = IMI 253801 = VKMF-2197. Infragen. class: subgen. *Aspergilloides*, sect. *Cinnamopurpurea*, ser. *Idahoensia*. Reproduction: asexual. ITS barcode: EU427300 (alternative markers: *BenA* = EU427268; *CaM* = KJ866997; *RPB2* = JN406626).

***Penicillium malachiteum*** (Yaguchi & Udagawa) Houbraken & Samson, Stud. Mycol. 70: 47. 2011. [MB561971]. Basionym: *Chromocleista malachitea* Yaguchi & Udagawa, Trans. Mycol. Soc. Japan 34: 102. 1993. [MB360067]. — Type: CBS 647.95. Ex-type: CBS 647.95 = IBT 17515. Infragen. class: subgen. *Aspergilloides*, sect. *Sclerotiorum*, ser. *Herqueorum*. Reproduction: homothallic. ITS barcode: KC773838 (alternative markers: *BenA* = KC773794; *CaM* = KC773820; *RPB2* = MN969125).

***Penicillium malacosphaerulum*** Visagie & K. Jacobs, Mycol. Prog. 14: 96, 16. 2015. [MB809819]. — Type: CBS H-21332. Ex-type: CBS 135120 = CV 2855 = CV 0311 = DTO 180-E6 = DAOM 241161. Infragen. class: subgen. *Aspergilloides*, sect. *Lanata-Divaricata*, ser. *Janthinella*. Reproduction: homothallic. ITS barcode: FJ231026 (alternative markers: *BenA* = JX091524; *CaM* = JX141542; *RPB2* = KF296438).

***Penicillium mali-pumilae*** Hyang B. Lee *et al.*, Fungal Diversity 96: 101. 2019. [MB555410]. — Type: CBS H-22503. Ex-type: CBS 140671 = DTO 327-D1= EML-MP6080-1 = IBT 33672. Infragen. class: subgen. *Penicillium*, sect. *Fasciculata*, ser. *Corymbifera*. Reproduction: asexual. ITS barcode: KP900991 (alternative markers: *BenA* = MT425584; *CaM* = KP900992; *RPB2* = MN969213).

***Penicillium mallochii*** K.G. Rivera *et al.*, Mycotaxon 119: 322. 2012. [MB563043]. — Type: DAOM 239917. Ex-type: CCFC 239917. Infragen. class: subgen. *Aspergilloides*, sect. *Sclerotiorum*, ser. *Sclerotiorum*. Reproduction: asexual. ITS barcode: JN626104 (alternative markers: *BenA* = JN625973; *CaM* = JN626016; *RPB2* = KX961296).

***Penicillium malmesburiense*** Visagie *et al.*, Stud. Mycol. 78: 429. 2014. [MB809969]. — Type: CBS H-21872. Ex-type: CBS 137744 = DTO 182-H5 = CV 1180 = DAOM 241144. Infragen. class: subgen. *Aspergilloides*, sect. *Aspergilloides*, ser. *Sublectatica*. Reproduction: asexual. ITS barcode: KM189676 (alternative markers: *BenA* = KM088921; *CaM* = KM089308; *RPB2* = KM089695).

***Penicillium malodoratum*** (Kwon-Chung & Fennell) Samson *et al.*, Stud. Mycol. 78: 355. 2014. [MB809316]. Basionym: *Aspergillus malodoratus* Kwon-Chung & Fennell, Gen. Aspergillus: 468. 1965. [MB326644]. — Type: IMI 172289. Ex-type: CBS 490.65 = NRRL 5083 = IMI 172289 = ATCC 16834. Infragen. class: subgen. *Penicillium*, sect. *Paradoxa*, ser. *Paradoxa*. Reproduction: asexual. ITS barcode: AF033485 (alternative markers: *BenA* = EF669681; *CaM* = FJ530972; *RPB2* = EF669672).

***Penicillium manginii*** Duché & R. Heim, Trav. Cryptog.: 450. 1931. [MB270490]. — Type: CBS 253.31. Ex-type: CBS 253.31 = DTO 022-E9 = NRRL 2134. Infragen. class: subgen. *Aspergilloides*, sect. *Citrina*, ser. *Westlingiorum*. Reproduction: asexual. ITS barcode: GU944599 (alternative markers: *BenA* = JN606651; *CaM* = MN969274; *RPB2* = JN606618).

***Penicillium mariae-crucis*** Quintan., Av. Aliment. Mejora Anim. 23: 334. 1982. [MB114171]. — Type: CBS 270.83. Ex-type: CBS 271.83 = IMI 256075. Infragen. class: subgen. *Aspergilloides*, sect. *Lanata-Divaricata*, ser. *Simplicissima*. Reproduction: asexual. ITS barcode: GU981593 (alternative markers: *BenA* = GU981630; *CaM* = MN969275; *RPB2* = KF296439).

***Penicillium marinum*** Frisvad & Samson, Stud. Mycol. 49: 20. 2004. [MB370974]. — Type: CBS 109550. Ex-type: DTO 141-E5 = CBS 109550 = IBT 14360. Infragen. class: subgen. *Penicillium*, sect. *Penicillium*, ser. *Penicillium*. Reproduction: asexual. ITS barcode: KJ834512 (alternative markers: *BenA* = AY674392; *CaM* = KU896842; *RPB2* = KU904357).

***Penicillium marthae-christenseniae*** Visagie & Samson, Persoonia 36: 145. 2016. [MB808267]. — Type: CBS H-21613. Ex-type: CBS 129213 = DTO 201-B5. Infragen. class: subgen. *Aspergilloides*, sect. *Torulomyces*, ser. *Torulomyces*. Reproduction: asexual. ITS barcode: KF303651 (alternative markers: *BenA* = KF303613; *CaM* = KF303645; *RPB2* = KF303711).

***Penicillium maximae*** Visagie *et al.*, Persoonia 31: 52. 2013. [MB803783]. — Type: CBS H-21144. Ex-type: CBS 134565 = NRRL 2060. Infragen. class: subgen. *Aspergilloides*, sect. *Sclerotiorum*, ser. *Sclerotiorum*. Reproduction: asexual. ITS barcode: EU427298 (alternative markers: *BenA* = KC773795; *CaM* = KC773821; *RPB2* = MN969126).

***Penicillium mediterraneum*** Guevara-Suarez *et al.*, Fungal Syst. Evol. 5: 61. 2019 [2020]. [MB822071]. — Type: CBS H-23143. Ex-type: CBS 142754 = FMR 15188. Infragen. class: subgen. *Penicillium*, sect. *Roquefortorum*, ser. *Roquefortorum*. Reproduction: asexual. ITS barcode: LT899784 (alternative markers: *BenA* = LT898291; *CaM* = LT899768; *RPB2* = LT899802).

***Penicillium melanoconidium*** (Frisvad) Frisvad & Samson, Stud. Mycol. 49: 28. 2004. [MB368219]. Basionym: *Penicillium aurantiogriseum* var. *melanoconidium* Frisvad, Mycologia 81: 849. 1989. [MB126407]. — Type: IMI 321503. Ex-type: DTO 158-D1 = CBS 115506 = IBT 3444 = IMI 321503. Infragen. class: subgen. *Penicillium*, sect. *Fasciculata*, ser. *Viridicata*. Reproduction: asexual. ITS barcode: MN431393 (alternative markers: *BenA* = MN969387; *CaM* = KU896843; *RPB2* = KU904358).

***Penicillium melanostipe*** Houbraken & Samson, Stud. Mycol. 70: 47. 2011. [MB561970]. Replaced synonym: *Thysanophora verrucosa* Mercado *et al.*, Mycotaxon 67: 419. 1998. [MB443755]. — Type: HAC (M) 9165. Ex-type: n.a. Infragen. class: subgen. *Aspergilloides*, sect. *Thysanophora*, ser. *Thysanophora*. Reproduction: asexual. ITS barcode: n.a. (alternative markers: *BenA* = n.a.; *CaM* = n.a.; *RPB2* = n.a.).

***Penicillium melinii*** Thom, Penicillia: 273. 1930. [MB270876]. — Type: IMI 40216. Ex-type: CBS 218.30 = ATCC 10469 = FRR 2041 = IFO 7675 = IMI 040216 = MUCL 29235 = NRRL 2041 = QM 7599. Infragen. class: subgen. *Aspergilloides*, sect. *Exilicaulis*, ser. *Lapidosa*. Reproduction: asexual. ITS barcode: AF033449 (alternative markers: *BenA* = KJ834471; *CaM* = KP016792; *RPB2* = JN406613).

***Penicillium meliponae*** R.N. Barbosa *et al.*, Antonie van Leeuwenhoek 111: 1897. 2018. [MB822210]. — Type: URM 90491. Ex-type: CBS 142495 = URM 7602. Infragen. class: subgen. *Aspergilloides*, sect. *Sclerotiorum*, ser. *Sclerotiorum*. Reproduction: asexual. ITS barcode: MF278315 (alternative markers: *BenA* = MN969418; *CaM* = LT854648; *RPB2* = LT854653).

***Penicillium mellis*** R.N. Barbosa *et al.*, Antonie van Leeuwenhoek 111: 1900. 2018. [MB822211]. — Type: URM 90492. Ex-type: CBS 142499 = URM 7605. Infragen. class: subgen. *Aspergilloides*, sect. *Sclerotiorum*, ser. *Adametziorum*. Reproduction: asexual. ITS barcode: MN431398 (alternative markers: *BenA* = MN969417; *CaM* = MN969327; *RPB2* = LT854652).

***Penicillium meloforme*** Udagawa & Y. Horie, Trans. Mycol. Soc. Japan 14: 376. 1973. [MB120882]. — Type: NHL 6468. Ex-type: CBS 445.74 = DTO 101-B3 = ATCC 28049 = IMI 216903 = NHL 6468. Infragen. class: subgen. *Aspergilloides*, sect. *Lanata-Divaricata*, ser. *Janthinella*. Reproduction: homothallic. ITS barcode: KC411762 (alternative markers: *BenA* = GU981656; *CaM* = MN969276; *RPB2* = KF296440).

***Penicillium menonorum*** S.W. Peterson, IMA Fungus 2: 122. 2011. [MB519297]. — Type: BPI 881018. Ex-type: NRRL 50410. Infragen. class: subgen. *Aspergilloides*, sect. *Exilicaulis*, ser. *Erubescentia*. Reproduction: asexual. ITS barcode: HQ646591 (alternative markers: *BenA* = HQ646573; *CaM* = HQ646584; *RPB2* = KF900194).

***Penicillium meridianum*** D.B. Scott, Mycopathol. Mycol. Appl. 36: 12. 1968. [MB335750]. — Type: CBS 314.67. Ex-type: CBS 314.67 = ATCC 18545 = CSIR 1052 = IMI 136209. Infragen. class: subgen. *Aspergilloides*, sect. *Exilicaulis*, ser. *Restricta*. Reproduction: homothallic. ITS barcode: AF033451 (alternative markers: *BenA* = KJ834472; *CaM* = KP016794; *RPB2* = JN406576).

***Penicillium mexicanum*** Visagie *et al.*, Stud. Mycol. 78: 125. 2014. [MB809185]. — Type: CBS H-21805. Ex-type: CBS 138227 = DTO 270-F1. Infragen. class: subgen. *Penicillium*, sect. *Paradoxa*, ser. *Atramentosa*. Reproduction: asexual. ITS barcode: KJ775685 (alternative markers: *BenA* = KJ775178; *CaM* = KJ775412; *RPB2* = MN969127).

***Penicillium miczynskii*** K.W. Zaleski, Bull. Int. Acad. Polon. Sci., Sér. B., Sci. Nat. 1927: 482. 1927. [MB271171]. — Type: IMI 40030. Ex-type: CBS 220.28 = DTO 022-E5 = ATCC 10470 = DSM2437 = FRR 1077 = IFO 7730 = IMI 040030 = MUCL 29228 = NRRL 1077 = QM 1957. Infragen. class: subgen. *Aspergilloides*, sect. *Citrina*, ser. *Westlingiorum*. Reproduction: asexual. ITS barcode: GU944600 (alternative markers: *BenA* = JN606706; *CaM* = MN969277; *RPB2* = JN606623).

***Penicillium minnesotense*** Jurjević *et al.*, Persoonia 42: 445. 2019. [MB830666]. — Type: BPI 910934. Ex-type: NRRL 66823 = ITEM 17524 = EMSL 1719. Infragen. class: subgen. *Aspergilloides*, sect. *Cinnamopurpurea*, ser. *Idahoensia*. Reproduction: asexual. ITS barcode: MK791277 (alternative markers: *BenA* = MK803429; *CaM* = MK803430; *RPB2* = MK796158).

***Penicillium momoii*** Visagie & K. Jacobs, IMA Fungus 7: 99. 2016. [MB811007]. — Type: CBS H-22046. Ex-type: CBS 139157 = DAOMC 241077 = DTO 182-G4 = CV 1015. Infragen. class: subgen. *Aspergilloides*, sect. *Exilicaulis*, ser. *Corylophila*. Reproduction: asexual. ITS barcode: JX140895 (alternative markers: *BenA* = JX141073; *CaM* = JX157479; *RPB2* = KP064673).

***Penicillium mononematosum*** (Frisvad *et al.*) Frisvad, Mycologia 81: 857. 1990. [MB126406]. Basionym: *Penicillium glandicola* var. *mononematosum* Frisvad, Filt. & Wicklow, Canad. J. Bot. 65: 767. 1987. [MB131770]. — Type: IMI 296925. Ex-type: CBS 172.87 = IBT 3072 = IBT 5518 = IBT 21535 = IMI 296925 = NRRL 13482 = NRRL A-26709. Infragen. class: subgen. *Penicillium*, sect. *Chrysogena*, ser. *Chrysogena*. Reproduction: asexual. ITS barcode: JX997082 (alternative markers: *BenA* = AY495997; *CaM* = JX996964; *RPB2* = JX996709).

***Penicillium monsgalena*** S.W. Peterson *et al.*, PLoS ONE 10: 0121987, 17. 2015. [MB807372]. — Type: BPI 881282. Ex-type: NRRL 22302 = IBT 29713. Infragen. class: subgen. *Aspergilloides*, sect. *Cinnamopurpurea*, ser. *Idahoensia*. Reproduction: asexual. ITS barcode: KF932959 (alternative markers: *BenA* = KF932927; *CaM* = KF932943; *RPB2* = KF932997).

***Penicillium monsserratidens*** S.W. Peterson *et al.*, PLoS ONE 10: 0121987, 19. 2015. [MB807373]. — Type: BPI 881285. Ex-type: NRRL 35884 = IBT 29695. Infragen. class: subgen. *Aspergilloides*, sect. *Cinnamopurpurea*, ser. *Idahoensia*. Reproduction: asexual. ITS barcode: KF932962 (alternative markers: *BenA* = KF932930; *CaM* = KF932947; *RPB2* = KF933001).

***Penicillium montanense*** M. Chr. & Backus, Mycologia 54: 574. 1962. [MB335752]. — Type: WIS Cryptogamic Herb. No. GW1-6. Ex-type: CBS 310.63 = ATCC 14941 = FRR 3407 = IFO 7740 = IHEM 4375 = IMI 099468 = MUCL 31326 = NRRL 3407. Infragen. class: subgen. *Aspergilloides*, sect. *Aspergilloides*, ser. *Pinetorum*. Reproduction: asexual. ITS barcode: KM189551 (alternative markers: *BenA* = KM088789; *CaM* = KM089174; *RPB2* = KM089561).

***Penicillium murcianum*** C. Ramírez & A.T. Martínez, Mycopathologia 74: 37. 1981 [MB112524]. — Type: IJFM 7031. Ex-type: DTO 036-A2 = CBS 161.81 = ATCC 42239 = IJFM 7031 = IMI 253800 = VKMF-2196. Infragen. class: subgen. *Penicillium*, sect. *Canescentia*, ser. *Canescentia*. Reproduction: asexual. ITS barcode: MN431400 (alternative markers: *BenA* = MN969419; *CaM* = MN969341; *RPB2* = MN969202).

***Penicillium nalgiovense*** Laxa, Zentralbl. Bakteriol. Parasitenk., Abt. 2 86: 160. 1932. [MB114239]. — Type: CBS 352.48. Ex-type: CBS 352.48 = ATCC 10472 = IBT 21536 = IMI 039804 = MUCL 31194 = NRRL 911. Infragen. class: subgen. *Penicillium*, sect. *Chrysogena*, ser. *Chrysogena*. Reproduction: asexual. ITS barcode: AY371617 (alternative markers: *BenA* = KU896811; *CaM* = JX996974; *RPB2* = JX996719).

***Penicillium namyslowskii*** K.W. Zaleski, Bull. Int. Aead. Polonc. Sci., Cl. Sci. Math., Sér. B, Sci. Nat. 1927: 479. 1927. [MB272006]. — Type: CBS 353.48. Ex-type: CBS 353.48 = ATCC 11127 = IMI 040033 = MUCL 29226 = NRRL 1070. Infragen. class: subgen. *Aspergilloides*, sect. *Exilicaulis*, ser. *Lapidosa*. Reproduction: asexual. ITS barcode: AF033463 (alternative markers: *BenA* = JX141067; *CaM* = KP016795; *RPB2* = JF417430).

***Penicillium neocrassum*** R. Serra & S.W. Peterson, Mycologia 99: 81. 2007. [MB504767]. — Type: BPI 872161. Ex-type: CBS 122428 = NRRL 35639 = MUM 06.160. Infragen. class: subgen. *Penicillium*, sect. *Brevicompacta*, ser. *Brevicompacta*. Reproduction: asexual. ITS barcode: DQ645805 (alternative markers: *BenA* = DQ645794; *CaM* = DQ645809; *RPB2* = JN406633).

***Penicillium neoechinulatum*** (Frisvad *et al.*) Frisvad & Samson, Stud. Mycol. 49: 28. 2004. [MB368218]. Basionym: *Penicillium aurantiogriseum* var. *neoechinulatum* Frisvad *et al.*, Canad. J. Bot. 65: 767. 1987. [MB131767]. — Type: IMI 296937. Ex-type: CBS 169.87 = CBS 101135 = IBT 3493 = IBT 21537 = IMI 296937 = NRRL 13486. Infragen. class: subgen. *Penicillium*, sect. *Fasciculata*, ser. *Viridicata*. Reproduction: asexual. ITS barcode: JN942722 (alternative markers: *BenA* = MN969388; *CaM* = KU896844; *RPB2* = JN985406).

***Penicillium neomiczynskii*** A.L.J. Cole *et al.*, Stud. Mycol. 70: 105. 2011. [MB563192]. — Type: CBS H-20661. Ex-type: CBS 126231 = DTO 078-C2 = IBT 23560. Infragen. class: subgen. *Aspergilloides*, sect. *Citrina*, ser. *Westlingiorum*. Reproduction: asexual. ITS barcode: JN617671 (alternative markers: *BenA* = JN606705; *CaM* = MN969278; *RPB2* = MN969128).

***Penicillium nepalense*** Takada & Udagawa, Trans. Mycol. Soc. Japan 24: 146. 1983. [MB108327]. — Type: NHL 6482. Ex-type: CBS 203.84 = NHL 6482. Infragen. class: subgen. *Aspergilloides*, sect. *Exilicaulis*, ser. *Erubescentia*. Reproduction: homothallic. ITS barcode: KC411692 (alternative markers: *BenA* = KJ834474; *CaM* = KP016796; *RPB2* = JN121453).

***Penicillium nigricans*** Bainier ex Thom, Penicillia: 351. 1930. [MB119303]. — Type: CBS H-22051. Ex-type: CBS 354.48 = ATCC 10115 = IFO 6103 = IMI 039767 = NRRL 915 = QM 1933 = VKMF-313. Infragen. class: subgen. *Penicillium*, sect. *Canescentia*, ser. *Canescentia*. Reproduction: asexual. ITS barcode: KC411755 (alternative markers: *BenA* = KJ866965; *CaM* = KJ867012; *RPB2* = KP016857).

***Penicillium nodulum*** H.Z. Kong & Z.T. Qi, Mycosystema 1: 108. 1988. [MB135445]. — Type: CBS 227.89. Ex-type: CBS 227.89. Infragen. class: subgen. *Aspergilloides*, sect. *Cinnamopurpurea*, ser. *Nodula*. Reproduction: asexual. ITS barcode: KC411703 (alternative markers: *BenA* = KJ834475; *CaM* = KJ867003; *RPB2* = JN406603).

***Penicillium nordicum*** Dragoni & Cantoni ex C. Ramírez, Adv. Pen. Asp. Syst: 139. 1986 [1985]. [MB114762]. — Type: ATCC 44219. Ex-type: DTO 098-F7 = ATCC 44219 = IBT 13307. Infragen. class: subgen. *Penicillium*, sect. *Fasciculata*, ser. *Verrucosa*. Reproduction: asexual. ITS barcode: KJ834513 (alternative markers: *BenA* = MN969389; *CaM* = KU896845; *RPB2* = KU904359).

***Penicillium nothofagi*** Houbraken *et al.*, Stud. Mycol. 70: 105. 2011. [MB563189]. — Type: CBS H-20655. Ex-type: CBS 130383 = DTO 076-C2 = IBT 23018 = DTO 076-C2. Infragen. class: subgen. *Aspergilloides*, sect. *Citrina*, ser. *Westlingiorum*. Reproduction: asexual. ITS barcode: JN617712 (alternative markers: *BenA* = JN606732; *CaM* = JN606507; *RPB2* = MN969129).

***Penicillium novae-zeelandiae*** J.F.H. Beyma, Antonie van Leeuwenhoek 6: 275. 1940. [MB522253]. — Type: IMI 40584ii. Ex-type: CBS 137.41 = ATCC 10473 = IFO 31748 = IMI 040584ii = NRRL 2128 = QM 1934 = VKMF-2886 = DTO 035-D8. Infragen. class: subgen. *Penicillium*, sect. *Canescentia*, ser. *Atroveneta*. Reproduction: asexual. ITS barcode: JN617688 (alternative markers: *BenA* = MN969390; *CaM* = MN969279; *RPB2* = JN406628).

***Penicillium nucicola*** Visagie *et al.*, Persoonia 36: 259. 2016. [MB815771]. — Type: DAOM 695770. Ex-type: DAOMC 250522 = CBS 140987 = DTO 410-D7 = W 59 = KAS 2203. Infragen. class: subgen. *Penicillium*, sect. *Canescentia*, ser. *Atroveneta*. Reproduction: asexual. ITS barcode: KT887860 (alternative markers: *BenA* = KT887821; *CaM* = KT887782; *RPB2* = MN969171).

***Penicillium ochrochloron*** Biourge, Cellule 33: 269. 1923. [MB272701]. — Type: IMI 39806. Ex-type: CBS 357.48 = DTO 097-G2 = ATCC 10540 = IMI 039806 = NRRL 926 = QM 7604. Infragen. class: subgen. *Aspergilloides*, sect. *Lanata-Divaricata*, ser. *Rolfsiorum*. Reproduction: asexual. ITS barcode: GU981604 (alternative markers: *BenA* = GU981672; *CaM* = MN969280; *RPB2* = KF296445).

***Penicillium ochrosalmoneum*** Udagawa, J. Agric. Sci. Tokyo Nogyo Daig. 5: 10. 1959. [MB302409]. — Type: NHL 6048. Ex-type: CBS 489.66 = ATCC 18338 = CSIR 145 = IMI 116248ii = NRRL 35499. Infragen. class: subgen. *Aspergilloides*, sect. *Ochrosalmonea*, ser. *Ochrosalmonea*. Reproduction: homothallic (Scott & Stolk 1967). ITS barcode: EF626961 (alternative markers: *BenA* = EF506212; *CaM* = EF506237; *RPB2* = JN121524).

***Penicillium odoratum*** M. Chr. & Backus, Mycologia 53: 459. 1961. [MB335755]. — Type: WSF 2000. Ex-type: CBS 294.62 = CBS 296.62 = ATCC 14769 = DSM2419 = IFO 7741 = IMI 094208ii = NRRL 3007 = WSF2000. Infragen. class: subgen. *Aspergilloides*, sect. *Aspergilloides*, ser. *Livida*. Reproduction: asexual. ITS barcode: KC411730 (alternative markers: *BenA* = KJ834478; *CaM* = KM089363; *RPB2* = JN406583).

***Penicillium olsonii*** Bainier & Sartory, Ann. Mycol. 10: 398. 1912. [MB121021]. — Type: IMI 192502. Ex-type: CBS 232.60 = IBT 23473 = IMI 192502 = NRRL 13058 = NRRL 13716. Infragen. class: subgen. *Penicillium*, sect. *Brevicompacta*, ser. *Olsoniorum*. Reproduction: asexual. ITS barcode: EU587341 (alternative markers: *BenA* = AY674445; *CaM* = DQ658165; *RPB2* = JN121464).

***Penicillium onobense*** C. Ramírez & A.T. Martínez, Mycopathologia 74: 44. 1981. [MB112525]. — Type: CBS 174.81. Ex-type: CBS 174.81 = DTO 036-A8 = ATCC 42225 = IJFM 3026 = VKMF-2183. Infragen. class: subgen. *Aspergilloides*, sect. *Lanata-Divaricata*, ser. *Simplicissima*. Reproduction: asexual. ITS barcode: GU981575 (alternative markers: *BenA* = GU981627; *CaM* = MN969281; *RPB2* = KF296447).

***Penicillium oregonense*** Visagie & Samson, Persoonia 36: 145. 2016. [MB808268]. — Type: CBS H-21607. Ex-type: CBS 129775 = DTO 208-A5. Infragen. class: subgen. *Aspergilloides*, sect. *Torulomyces*, ser. *Torulomyces*. Reproduction: asexual. ITS barcode: KF303668 (alternative markers: *BenA* = KF303623; *CaM* = KF303640; *RPB2* = KF303710).

***Penicillium ornatum*** Udagawa, Trans. Mycol. Soc. Japan 9: 49. 1968. [MB335756]. — Type: NHL 6101. Ex-type: CBS 190.68 = ATCC 18608 = IFO 31739 = IMI 137977 = NHL 6101 = NRRL 3471. Infragen. class: subgen. *Aspergilloides*, sect. *Ramigena*, ser. *Ramigena*. Reproduction: homothallic. ITS barcode: KC411687 (alternative markers: *BenA* = KJ834479; *CaM* = MN969282; *RPB2* = JN121451).

***Penicillium ortum*** Visagie & K. Jacobs, Mycol. Prog. 14 (no. 96): 18. 2015. [MB809820]. — Type: CBS H-21602. Ex-type: CBS 135669 = CV 0102 = DTO 180-I9. Infragen. class: subgen. *Aspergilloides*, sect. *Lanata-Divaricata*, ser. *Janthinella*. Reproduction: asexual. ITS barcode: JX091427 (alternative markers: *BenA* = JX091520; *CaM* = JX141551; *RPB2* = KF296443).

***Penicillium osmophilum*** Stolk & Veenb.-Rijks, Antonie van Leeuwenhoek 40: 1. 1974. [MB319288]. — Type: CBS 462.72. Ex-type: DTO 092-C5 = CBS 462.72 = CBS 439.73 = IBT 14678 = NRRL 5922. Infragen. class: subgen. *Penicillium*, sect. *Osmophila*, ser. *Osmophila*. Reproduction: homothallic. ITS barcode: EU427295 (alternative markers: *BenA* = MN969391; *CaM* = KU896846; *RPB2* = JN121518).

***Penicillium ovatum*** (K. Ando & Nawawi) Houbraken & Samson, Stud. Mycol. 70: 48. 2011. [MB561961]. Basionym: *Torulomyces ovatus* K. Ando & Nawawi, Mycoscience 39: 317. 1998. [MB447111]. — Type: TNS-F-238518. Ex-type: CBS 136664 = KY 12726 = DTO 270-G7. Infragen. class: subgen. *Aspergilloides*, sect. *Exilicaulis*, ser. *Erubescentia*. Reproduction: asexual. ITS barcode: KF667370 (alternative markers: *BenA* = KF667366; *CaM* = KF667368; *RPB2* = KF667372).

***Penicillium oxalicum*** Currie & Thom, J. Biol. Chem. 22: 289. 1915. [MB121033]. — Type: IMI 192332. Ex-type: CBS 219.30 = ATCC 1126 = FRR 787 = IMI 192332 = MUCL 29047 = NRRL 787 = QM 7606. Infragen. class: subgen. *Aspergilloides*, sect. *Lanata-Divaricata*, ser. *Oxalica*. Reproduction: asexual. ITS barcode: AF033438 (alternative markers: *BenA* = KF296462; *CaM* = MN969283; *RPB2* = JN121456).

***Penicillium pagulum*** Visagie & K. Jacobs, IMA Fungus 7: 102. 2016. [MB811005]. — Type: CBS H-22049. Ex-type: CBS 139166 = DAOMC 241069 = DTO 183-H2 = CV 2224. Infragen. class: subgen. *Aspergilloides*, sect. *Exilicaulis*, ser. *Corylophila*. Reproduction: asexual. ITS barcode: JX140898 (alternative markers: *BenA* = JX141070; *CaM* = JX157519; *RPB2* = KP064655).

***Penicillium palitans*** Westling, Ark Bot. 11: 83. 1911. [MB203604]. — Type: CBS H-7531. Ex-type: DTO 206-F6 = CBS 107.11 = ATCC 10477 = IBT 23034 = IMI 040215 = NRRL 2033 = VKMF-3088. Infragen. class: subgen. *Penicillium*, sect. *Fasciculata*, ser. *Camembertiorum*. Reproduction: asexual. ITS barcode: KJ834514 (alternative markers: *BenA* = KJ834480; *CaM* = KU896847; *RPB2* = KU904360).

***Penicillium palmense*** C. Ramírez & A.T. Martínez, Mycopathologia 66: 80. 1978. [MB319289]. — Type: CBS 336.79. Ex-type: CBS 336.79 = ATCC 38669 = IJFM 3840. Infragen. class: subgen. *Aspergilloides*, sect. *Aspergilloides*, ser. *Spinulosa*. Reproduction: asexual. ITS barcode: KJ834515 (alternative markers: *BenA* = GQ367508; *CaM* = GQ367534; *RPB2* = JN406566).

***Penicillium pancosmium*** Houbraken *et al.*, Stud. Mycol. 70: 108. 2011. [MB563191]. — Type: CBS H-20651. Ex-type: CBS 276.75 = DTO 031-B4 = DAOM 147467 = IBT 29991. Infragen. class: subgen. *Aspergilloides*, sect. *Citrina*, ser. *Westlingiorum*. Reproduction: asexual. ITS barcode: JN617660 (alternative markers: *BenA* = JN606790; *CaM* = MN969284; *RPB2* = MN969130).

***Penicillium paneum*** Frisvad, Microbiology 142: 546. 1996. [MB415570]. — Type: C 25000. Ex-type: CBS 101032 = IBT 21541 = IBT 12407. Infragen. class: subgen. *Penicillium*, sect. *Roquefortorum*, ser. *Roquefortorum*. Reproduction: asexual. ITS barcode: HQ442346 (alternative markers: *BenA* = AY674387; *CaM* = HQ442331; *RPB2* = KU904361).

***Penicillium panissanguineum*** Visagie *et al.*, Persoonia 36: 275. 2016. [MB815783]. — Type: DAOM 695771. Ex-type: DAOMC 250562 = CBS 140989 = DTO 410-D8 = W 93 = KAS 2209. Infragen. class: subgen. *Aspergilloides*, sect. *Lanata-Divaricata*, ser. *Simplicissima*. Reproduction: asexual. ITS barcode: KT887862 (alternative markers: *BenA* = KT887823; *CaM* = KT887784; *RPB2* = MN969182).

***Penicillium paradoxum*** (Fennell & Raper) Samson *et al.*, Stud. Mycol. 78: 352. 2014. [MB547045]. Basionym: *Aspergillus paradoxus* Fennell & Raper, Mycologia 47: 69. 1955. [MB292853]. — Type: IMI 061446. Ex-type: CBS 527.65 = NRRL 2162 = ATCC 16918 = IMI 061446. Infragen. class: subgen. *Penicillium*, sect. *Paradoxa*, ser. *Paradoxa*. Reproduction: homothallic. ITS barcode: EF669707 (alternative markers: *BenA* = EF669683; *CaM* = EF669692; *RPB2* = EF669670).

***Penicillium paraherquei*** S. Abe ex G. Sm., Trans. Brit. Mycol. Soc. 46: 335. 1963. [MB302412]. — Type: IMI 68220. Ex-type: CBS 338.59 = DTO 015-D4 = DTO 097-F3 = ATCC 22354 = ATCC 46903 = FAT964 = FRR 3454 = IFO 6234 = IMI 068220 = NRRL 3454. Infragen. class: subgen. *Aspergilloides*, sect. *Lanata-Divaricata*, ser. *Simplicissima*. Reproduction: asexual. ITS barcode: AF178511 (alternative markers: *BenA* = KF296465; *CaM* = MN969285; *RPB2* = KF296449).

***Penicillium parviverrucosum*** (K. Ando & Pitt) Houbraken & Samson, Stud. Mycol. 70: 48. 2011. [MB561962]. Basionym: *Torulomyces parviverrucosus* K. Ando & Pitt, Mycoscience 39: 317. 1998. [MB447109]. — Type: TNS-F-238516. Ex-type: KY 12720. Infragen. class: subgen. *Aspergilloides*, sect. *Torulomyces*, ser. *Torulomyces*. Reproduction: asexual. ITS barcode: n.a. (alternative markers: *BenA* = n.a.; *CaM* = n.a.; *RPB2* = n.a.).

***Penicillium parvofructum*** Guevara-Suarez *et al.*, Persoonia 38: 353. 2017. [MB819947]. — Type: CBS H-22733. Ex-type: FMR 15047 = CBS 141690 = DTO 410-E6. Infragen. class: subgen. *Aspergilloides*, sect. *Exilicaulis*, ser. *Erubescentia*. Reproduction: asexual. ITS barcode: LT559091 (alternative markers: *BenA* = LT627645; *CaM* = LT627646; *RPB2* = MN969197).

***Penicillium parvulum*** S.W. Peterson & B.W. Horn, Mycologia 101: 75. 2009. [MB509289]. — Type: BPI 877331. Ex-type: CBS 132825 = NRRL 35504. Infragen. class: subgen. *Aspergilloides*, sect. *Cinnamopurpurea*, ser. *Cinnamopurpurea*. Reproduction: asexual. ITS barcode: EF422845 (alternative markers: *BenA* = EF506218; *CaM* = EF506225; *RPB2* = MN969131).

***Penicillium parvum*** Raper & Fennell, Mycologia 40: 508. 1948. [MB289101]. — Type: CBS 359.48. Ex-type: CBS 359.48 = ATCC 10479 = IFO 7732 = IMI 040587 = NRRL 2095 = QM 1878. Infragen. class: subgen. *Aspergilloides*, sect. *Exilicaulis*, ser. *Erubescentia*. Reproduction: homothallic. ITS barcode: AF033460 (alternative markers: *BenA* = HQ646568; *CaM* = KF900173; *RPB2* = JN406559).

***Penicillium pasqualense*** Houbraken *et al.*, Stud. Mycol. 70: 108. 2011. [MB563190]. — Type: CBS H-20663. Ex-type: CBS 126330 = DTO 080-D5 = IBT 14235. Infragen. class: subgen. *Aspergilloides*, sect. *Citrina*, ser. *Westlingiorum*. Reproduction: asexual. ITS barcode: JN617676 (alternative markers: *BenA* = JN606673; *CaM* = MN969286; *RPB2* = JN606617).

***Penicillium paxilli*** Bainier, Bull. Soc. Mycol. France 23: 95. 1907. [MB203838]. — Type: IMI 40226. Ex-type: CBS 360.48 = ATCC 10480 = FRR 2008 = IMI 040226 = NRRL 2008 = QM 725. Infragen. class: subgen. *Aspergilloides*, sect. *Citrina*, ser. *Paxillorum*. Reproduction: asexual. ITS barcode: GU944577 (alternative markers: *BenA* = JN606844; *CaM* = JN606566; *RPB2* = JN606610).

***Penicillium pedernalense*** Laich & J. Andrade, Index Fungorum 361: 1. 2018. [MB554533]. — Type: CBS 140770. Ex-type: CBS 140770 = CECT 20949 = DTO 366-A3. Infragen. class: subgen. *Aspergilloides*, sect. *Lanata-Divaricata*, ser. *Simplicissima*. Reproduction: asexual. ITS barcode: KU255398 (alternative markers: *BenA* = KU255396; *CaM* = MN969322; *RPB2* = MN969184).

***Penicillium penarojense*** Houbraken *et al.*, Int. J. Syst. Evol. Microbiol. 61: 1471. 2011. [MB518024]. — Type: HUA 170335. Ex-type: CBS 113178 = DTO 056-D1 = DTO 297-H9 = IBT 23262. Infragen. class: subgen. *Aspergilloides*, sect. *Lanata-Divaricata*, ser. *Dalearum*. Reproduction: asexual. ITS barcode: GU981570 (alternative markers: *BenA* = GU981646; *CaM* = MN969287; *RPB2* = KF296450).

***Penicillium persicinum*** L. Wang *et al.*, Antonie van Leeuwenhoek 86: 177. 2004. [MB500259]. — Type: HMAS 80638-1-4. Ex-type: CBS 111235 = AS 3.5891 = IBT 24565. Infragen. class: subgen. *Penicillium*, sect. *Chrysogena*, ser. *Persicina*. Reproduction: asexual. ITS barcode: JX997072 (alternative markers: *BenA* = JF909951; *CaM* = JX996954; *RPB2* = JN406644).

***Penicillium philippinense*** Udagawa & Y. Horie, J. Jap. Bot. 47: 341. 1972. [MB319291]. — Type: NHL 6130. Ex-type: CBS 623.72 = FRR 1532 = NHL 6130. Infragen. class: subgen. *Aspergilloides*, sect. *Exilicaulis*, ser. *Restricta*. Reproduction: homothallic. ITS barcode: KC411770 (alternative markers: *BenA* = KJ834482; *CaM* = KP016799; *RPB2* = JN406543).

***Penicillium phoeniceum*** J.F.H. Beyma, Zentralbl. Bakteriol. Parasitenk., Abt. 2 88: 136. 1933. [MB274284]. — Type: IMI 40585. Ex-type: CBS 249.32 = ATCC 10481 = IJFM 5122 = IMI 040585 = NRRL 2070 = QM 7608 = VKMF-321. Infragen. class: subgen. *Aspergilloides*, sect. *Charlesia*, ser. *Phoenicea*. Reproduction: asexual. ITS barcode: KC411711 (alternative markers: *BenA* = KJ834483; *CaM* = AY741729; *RPB2* = JN406597).

***Penicillium pimiteouiense*** S.W. Peterson, Mycologia 91: 271. 1999. [MB460126]. — Type: BPI 806262. Ex-type: CBS 102479 = NRRL 25542. Infragen. class: subgen. *Aspergilloides*, sect. *Exilicaulis*, ser. *Erubescentia*. Reproduction: asexual. ITS barcode: AF037431 (alternative markers: *BenA* = HQ646569; *CaM* = HQ646580; *RPB2* = JN406650).

***Penicillium piscarium*** Westling, Ark. Bot. 11: 86. 1911. [MB211321]. — Type: IMI 40032. Ex-type: CBS 362.48 = DTO 014-G9 = DTO 100-C1 = ATCC 10482 = FRR 1075 = IFO 8111 = IMI 040032 = NRRL 1075 = VKMF-1823. Infragen. class: subgen. *Aspergilloides*, sect. *Lanata-Divaricata*, ser. *Rolfsiorum*. Reproduction: asexual. ITS barcode: GU981600 (alternative markers: *BenA* = GU981668; *CaM* = MN969288; *RPB2* = KF296451).

***Penicillium polonicum*** K.W. Zaleski, Bull. Int. Acad. Polon. Sci., Sér. B., Sci. Nat. 1927: 445. 1927. [MB274889]. — Type: CBS 222.28. Ex-type: CBS 222.28 = IBT 12821 = IMI 291194 = MUCL 29204 = NRRL 995. Infragen. class: subgen. *Penicillium*, sect. *Fasciculata*, ser. *Viridicata*. Reproduction: asexual. ITS barcode: AF033475 (alternative markers: *BenA* = MN969392; *CaM* = KU896848; *RPB2* = JN406609).

***Penicillium porphyreum*** Houbraken & Samson, Stud. Mycol. 70: 48. 2011. [MB561959]. Replaced synonym: *Monocillium humicola* var. *brunneum* M. Chr. & Backus, Mycologia 56: 498.1964. [MB353642]. — Type: NY 00985491. Ex-type: CBS 382.64 = KY 12723 = DTO 078-G7. Infragen. class: subgen. *Aspergilloides*, sect. *Torulomyces*, ser. *Torulomyces*. Reproduction: asexual. ITS barcode: KF303666 (alternative markers: *BenA* = KF303621; *CaM* = KF303636; *RPB2* = KF303677).

***Penicillium psychrosexuale*** [as “*psychrosexualis*”] Houbraken & Samson, IMA Fungus 1: 174. 2010. [MB834590]. — Type: CBS H-20501. Ex-type: DTO 070-G9 = CBS 128137 = IBT 29551. Infragen. class: subgen. *Penicillium*, sect. *Roquefortorum*, ser. *Roquefortorum*. Reproduction: homothallic. ITS barcode: HQ442345 (alternative markers: *BenA* = HQ442356; *CaM* = HQ442330; *RPB2* = KU904362).

***Penicillium psychrotrophicum*** Hyang B. Lee *et al.*, Fungal Diversity 96: 103. 2019. [MB555409]. — Type: CBS H-22504. Ex-type: CBS 140670 = DTO 327-C9 = EML-COD3 = IBT 33673. Infragen. class: subgen. *Penicillium*, sect. *Fasciculata*, ser. *Corymbifera*. Reproduction: asexual. ITS barcode: KP941754 (alternative markers: *BenA* = KP900995; *CaM* = KP900994; *RPB2* = MN969212).

***Penicillium pullum*** S.W. Peterson & Sigler, Mycol. Res. 106: 1115. 2002. [MB483982]. — Type: BPI 841398. Ex-type: CBS 331.48 = ATCC 10447 = NRRL 721 = FRR 721 = IFO 6097 = IMI 39747 = QM 1925 = Thom 5179.4. Infragen. class: subgen. *Aspergilloides*, sect. *Stolkia*, ser. *Stolkia*. Reproduction: asexual. ITS barcode: AF033443 (alternative markers: *BenA* = JN617719; *CaM* = AF481134; *RPB2* = MN969132).

***Penicillium pulvillorum*** Turfitt, Trans. Brit. Mycol. Soc. 23: 186. 1939. [MB275682]. — Type: CBS 280.39. Ex-type: CBS 280.39 = DTO 014-G7 = DTO 094-D5 = IFO 7763 = NRRL 2026. Infragen. class: subgen. *Aspergilloides*, sect. *Lanata-Divaricata*, ser. *Rolfsiorum*. Reproduction: asexual. ITS barcode: AF178517 (alternative markers: *BenA* = GU981670; *CaM* = MN969289; *RPB2* = KF296452).

***Penicillium pulvis*** Houbraken *et al.*, Stud. Mycol. 78: 429. 2014. [MB809970]. — Type: CBS H-21878. Ex-type: CBS 138432 = DTO 180-B7. Infragen. class: subgen. *Aspergilloides*, sect. *Aspergilloides*, ser. *Glabra*. Reproduction: asexual. ITS barcode: KM189632 (alternative markers: *BenA* = KM088876; *CaM* = KM089263; *RPB2* = KM089650).

***Penicillium punicae*** Hyang B. Lee *et al.*, Fungal Diversity 83: 103. 2017. [MB818233]. — Type: CNUFC-FP2-1. Ex-type: JMRC:SF:12421. Infragen. class: subgen. *Aspergilloides*, sect. *Exilicaulis*, ser. *Corylophila*. Reproduction: asexual. ITS barcode: n.a. (alternative markers: *BenA* = KX839673; *CaM* = KX839671; *RPB2* = KX839675).

***Penicillium purpurescens*** [as “*purpurascens*”] (Sopp) Biourge, La Cellule 33: 105. 1923. [MB335761]. Basionym: *Citromyces purpurrescens* Sopp, Skr. Vidensk.-Selsk. Christiana, Math.-Naturvidensk. Kl. 11: 117. 1912. [MB568761]. — Type: IMI 39745. Ex-type: CBS 366.48 = NRRL 720 = FRR 720 = ATCC 10485 = IMI 39745. Infragen. class: subgen. *Aspergilloides*, sect. *Aspergilloides*, ser. *Glabra*. Reproduction: asexual. ITS barcode: KM189561 (alternative markers: *BenA* = KM088801; *CaM* = KM089186; *RPB2* = KM089573).

***Penicillium pusillum*** G. Sm., Trans. Brit. Mycol. Soc. 22 (3–4): 254. 1939. [MB275810]. — Type: unknown. Ex-type: CBS 312.63 = FRR 1541 = IMI 089286 = LSHBB147 = NRRL 2498. Infragen. class: subgen. *Aspergilloides*, sect. *Cinnamopurpurea*, ser. *Jiangxiensia*. Reproduction: asexual. ITS barcode: EF626951 (alternative markers: *BenA* = KF932925; *CaM* = KF932941; *RPB2* = KF932995).

***Penicillium quebecense*** Houbraken *et al.*, Stud. Mycol. 70: 111. 2011. [MB563202]. — Type: CBS H-20666. Ex-type: CBS 101623 = DTO 009-B8 = IBT 29050. Infragen. class: subgen. *Aspergilloides*, sect. *Citrina*, ser. *Westlingiorum*. Reproduction: asexual. ITS barcode: JN617661 (alternative markers: *BenA* = JN606700; *CaM* = JN606509; *RPB2* = JN606622).

***Penicillium quercetorum*** Baghd., Novosti Sist. Nizsh. Rast. 5: 110. 1968. [MB335762]. — Type: CBS H-7527. Ex-type: CBS 417.69 = NRRL 3758 = ATCC 48727 = CCRC 31668 = FRR 516 = IFO 31749 = IMI 140342 = MUCL 31203 = VKMF-1074. Infragen. class: subgen. *Aspergilloides*, sect. *Aspergilloides*, ser. *Quercetorum*. Reproduction: asexual. ITS barcode: KM189556 (alternative markers: *BenA* = KM088795; *CaM* = KM089180; *RPB2* = KM089567).

***Penicillium raciborskii*** K.W. Zaleski, Bull. Int. Acad. Polon. Sci., Sér. B., Sci. Nat. 1927: 454. 1927. [MB276002]. — Type: IMI 40568. Ex-type: CBS 224.28 = ATCC 10488 = DSM2422 = FRR 2150 = IFO 7676 = IMI 040568 = LSHBP 92 = MUCL 29246 = NRRL 2150. Infragen. class: subgen. *Aspergilloides*, sect. *Exilicaulis*, ser. *Lapidosa*. Reproduction: asexual. ITS barcode: AF033447 (alternative markers: *BenA* = JX141069; *CaM* = KP016800; *RPB2* = JN406607).

***Penicillium radiatolobatum*** Lörinczi, Publ. Soc. Nat. Rom. Pent. Stiinta Sol. 10B: 435. 1972. [MB114326]. — Type: CBS H-7530. Ex-type: CBS 340.79. Infragen. class: subgen. *Penicillium*, sect. *Canescentia*, ser. *Canescentia*. Reproduction: asexual. ITS barcode: KC411745 (alternative markers: *BenA* = MN969413; *CaM* = MT066183; *RPB2* = MN969168).

***Penicillium radicicola*** Overy & Frisvad, Syst. Appl. Microbiol. 26: 633. 2003. [MB488233]. — Type: C 60161. Ex-type: CBS 112430 = IBT 10696 = DTO 051-E1. Infragen. class: subgen. *Penicillium*, sect. *Fasciculata*, ser. *Corymbifera*. Reproduction: asexual. ITS barcode: KJ834516 (alternative markers: *BenA* = MN969393; *CaM* = MN969290; *RPB2* = MN969133).

***Penicillium raistrickii*** G. Sm., Trans. Brit. Mycol. Soc. 18: 90. 1933. [MB276069]. — Type: IMI 40221. Ex-type: CBS 261.33 = ATCC 10490 = FRR 1044 = IFO 6104 = IMI 040221 = LSHBB100 = NRRL 1044 = NRRL 2039 = QM 1936 = VKMF-337. Infragen. class: subgen. *Penicillium*, sect. *Ramosum*, ser. *Raistrickiorum*. Reproduction: asexual. ITS barcode: AY373927 (alternative markers: *BenA* = KJ834485; *CaM* = KJ867006; *RPB2* = JN406592).

***Penicillium ramusculum*** Bat. & H. Maia, Anais Soc. Biol. Pernambuco 13: 27. 1955. [MB302419]. — Type: unknown. Ex-type: CBS 251.56 = ATCC 12292 = FRR 3459 = IMI 063546 = IMUR478 = LSHBBB324 = NRRL 3459 = QM 7057. Infragen. class: subgen. *Aspergilloides*, sect. *Ramigena*, ser. *Ramigena*. Reproduction: asexual. ITS barcode: EF433765 (alternative markers: *BenA* = EU427269; *CaM* = EU427278; *RPB2* = JN121472).

***Penicillium ranomafanaense*** Houbraken & Hagen, Stud. Mycol. 78: 433. 2014. [MB809971]. — Type: CBS H-21862. Ex-type: CBS 137953 = DTO 085-A5. Infragen. class: subgen. *Aspergilloides*, sect. *Aspergilloides*, ser. *Verhageniorum*. Reproduction: asexual. ITS barcode: KM189541 (alternative markers: *BenA* = KM088779; *CaM* = KM089164; *RPB2* = KM089551).

***Penicillium raperi*** G. Sm., Trans. Brit. Mycol. Soc. 40: 486. 1957. [MB302421]. — Type: IMI 71625. Ex-type: CBS 281.58 = DTO 097-F6 = DTO 014-H4 = ATCC 22355 = IFO 8179 = IMI 071625 = LSHBBB338 = NRRL 2674 = QM 7527. Infragen. class: subgen. *Aspergilloides*, sect. *Lanata-Divaricata*, ser. *Janthinella*. Reproduction: asexual. ITS barcode: AF033433 (alternative markers: *BenA* = GU981622; *CaM* = MN969291; *RPB2* = KF296453).

***Penicillium raphiae*** Houbraken *et al.*, Stud. Mycol. 70: 114. 2011. [MB563203]. — Type: CBS H-20660. Ex-type: CBS 126234 = DTO 078-B8 = IBT 22407. Infragen. class: subgen. *Aspergilloides*, sect. *Citrina*, ser. *Westlingiorum*. Reproduction: asexual. ITS barcode: JN617673 (alternative markers: *BenA* = JN606657; *CaM* = MN969292; *RPB2* = JN606619).

***Penicillium reconvexovelosoi*** J.P. Andrade *et al.*, Persoonia 43: 383, 2019. [MB832747]. — Type: HURB 18575. Ex-type: CCDCA 11500. Infragen. class: subgen. *Aspergilloides*, sect. *Sclerotiorum*, ser. *Adametziorum*. Reproduction: asexual. ITS barcode: n.a. (alternative markers: *BenA* = MN497417; *CaM* = MN497418; *RPB2* = n.a.).

***Penicillium repensicola*** Visagie & K. Jacobs, IMA Fungus 7: 102. 2016. [MB811006]. — Type: CBS H-22047. Ex-type: CBS 139160 = DAOMC 241080 = DTO 183-B8 = CV 1495. Infragen. class: subgen. *Aspergilloides*, sect. *Exilicaulis*, ser. *Corylophila*. Reproduction: asexual. ITS barcode: JX140893 (alternative markers: *BenA* = JX141150; *CaM* = JX157490; *RPB2* = KP064660).

***Penicillium restingae*** J.P. Andrade *et al.*, Persoonia 32: 293. 2014. [MB807051]. — Type: CMR H-12. Ex-type: CBS 140379 = URM 7075 = DTO 331-H7. Infragen. class: subgen. *Aspergilloides*, sect. *Sclerotiorum*, ser. *Adametziorum*. Reproduction: asexual. ITS barcode: KF803355 (alternative markers: *BenA* = KF803349; *CaM* = KF803352; *RPB2* = MN969134).

***Penicillium restrictum*** J.C. Gilman & E.V. Abbott, Iowa St. Coll. J. Sci. 1: 297. 1927. [MB276289]. — Type: IMI 40228. Ex-type: CBS 367.48 = ATCC 11257 = FRR 1748 = IMI 040228 = NRRL 1748 = QM 1962. Infragen. class: subgen. *Aspergilloides*, sect. *Exilicaulis*, ser. *Restricta*. Reproduction: asexual. ITS barcode: AF033457 (alternative markers: *BenA* = KJ834486; *CaM* = KP016803; *RPB2* = JN121506).

***Penicillium reticulisporum*** Udagawa, Trans. Mycol. Soc. Japan 9: 52. 1968. [MB335763]. — Type: NHL 6105. Ex-type: CBS 122.68 = ATCC 18566 = IFO 9024 = IMI 136700 = NHL 6105 = NRRL 3447 = DTO 097-C4. Infragen. class: subgen. *Aspergilloides*, sect. *Lanata-Divaricata*, ser. *Janthinella*. Reproduction: homothallic. ITS barcode: AF033437 (alternative markers: *BenA* = MN969394; *CaM* = MN969293; *RPB2* = KF296454).

***Penicillium ribium*** Frisvad & Overy, Int. J. Syst. Evol. Microbiol. 56: 1436. 2006. [MB501061]. — Type: DAOM 234091. Ex-type: CBS 127809 = DAOM 234091 = IBT 16537 = IBT 24431. Infragen. class: subgen. *Penicillium*, sect. *Ramosum*, ser. *Lanosa*. Reproduction: asexual. ITS barcode: DQ267916 (alternative markers: *BenA* = MN969395; *CaM* = KJ866995; *RPB2* = JN406631).

***Penicillium riverlandense*** Visagie & K. Jacobs, Persoonia 36: 149. 2016. [MB808269]. — Type: CBS H-21606. Ex-type: CBS 135896 = CV 0979 = DTO 182-F6 = DAOMC 241060. Infragen. class: subgen. *Aspergilloides*, sect. *Torulomyces*, ser. *Torulomyces*. Reproduction: asexual. ITS barcode: JX091457 (alternative markers: *BenA* = JX091580; *CaM* = JX141593; *RPB2* = KF303685).

***Penicillium robsamsonii*** Frisvad & Houbraken, Persoonia 36: 313. 2016. [MB815872]. — Type: CBS H-22341. Ex-type: CBS 140573 = IBT 29466 = DTO 149-B6. Infragen. class: subgen. *Penicillium*, sect. *Robsamsonia*, ser. *Robsamsonia*. Reproduction: asexual. ITS barcode: KU904339 (alternative markers: *BenA* = KT698885; *CaM* = KT698894; *RPB2* = KT698904).

***Penicillium rolfsii*** Thom, Penicillia: 489. 1930. [MB276674]. — Type: IMI 40029. Ex-type: CBS 368.48 = DTO 106-H8 = ATCC 10491 = FRR 1078 = IFO 7735 = IMI 040029 = MUCL 29229 = NRRL 1078 = QM 1961. Infragen. class: subgen. *Aspergilloides*, sect. *Lanata-Divaricata*, ser. *Rolfsiorum*. Reproduction: asexual. ITS barcode: JN617705 (alternative markers: *BenA* = GU981667; *CaM* = MN969294; *RPB2* = KF296455).

***Penicillium roqueforti*** Thom, U.S.D.A. Bur. Animal Industr. Bull. 82: 35. 1906. [MB213525]. — Type: IMI 24313. Ex-type: CBS 221.30 = ATCC 10110 = ATCC 1129 = CECT 2905 = IBT 6754 = IFO 5459 = IMI 024313 = LSHBP 93 = NCTC 588 = NRRL 849 = QM 1937. Infragen. class: subgen. *Penicillium*, sect. *Roquefortorum*, ser. *Roquefortorum*. Reproduction: Heterothallic (Ropars *et al.* 2014). ITS barcode: HQ442347 (alternative markers: *BenA* = MN969396; *CaM* = HQ442332; *RPB2* = JN406611).

***Penicillium roseomaculatum*** Biourge, Cellule 33: 301. 1923. [MB276785]. — Type: unknown. Ex-type: CBS 137962 = IMI 189696 = NRRL 728 = FRR 728. Infragen. class: subgen. *Aspergilloides*, sect. *Aspergilloides*, ser. *Spinulosa*. Reproduction: asexual. ITS barcode: KM189755 (alternative markers: *BenA* = KM089004; *CaM* = KM089391; *RPB2* = KM089778).

***Penicillium roseopurpureum*** Dierckx, Ann. Soc. Sci. Bruxelles 25: 86. 1901. [MB213447]. — Type: IMI 40573. Ex-type: CBS 226.29 = ATCC 10492 = ATHUM2895 = FRR 2064 = IMI 040573 = MUCL 28654 = MUCL 29237 = NRRL 2064 = NRRL 2064A. Infragen. class: subgen. *Aspergilloides*, sect. *Citrina*, ser. *Roseopurpurea*. Reproduction: asexual. ITS barcode: GU944605 (alternative markers: *BenA* = JN606838; *CaM* = JN606556; *RPB2* = JN606613).

***Penicillium roseoviride*** Stapp & Bortels, Zentralbl. Bakteriol. Parasitenk., Abt. 2 93: 51. 1935. [MB492646]. — Type: unknown. Ex-type: CBS 267.35 = ATCC 10412 = IFO 6089 = IMI 039740ii = NRRL 760 = QM 7485. Infragen. class: subgen. *Aspergilloides*, sect. *Aspergilloides*, ser. *Thomiorum*. Reproduction: asexual. ITS barcode: KM189549 (alternative markers: *BenA* = KM088787; *CaM* = KM089172; *RPB2* = KM089559).

***Penicillium rubefaciens*** Quintan., Mycopathologia 80: 73. 1982. [MB109998]. — Type: CBS 145.83. Ex-type: CBS 145.83 = CECT 2752. Infragen. class: subgen. *Aspergilloides*, sect. *Exilicaulis*, ser. *Corylophila*. Reproduction: asexual. ITS barcode: KC411677 (alternative markers: *BenA* = KJ834487; *CaM* = KP016804; *RPB2* = JN406627).

***Penicillium rubens*** Biourge, Cellule 33: 265. 1923. [MB276884]. — Type: CBS H-20595. Ex-type: DTO 098-E8 = CBS 129667 = NRRL 792 = IBT 30129 = ATCC 9783. Infragen. class: subgen. *Penicillium*, sect. *Chrysogena*, ser. *Chrysogena*. Reproduction: Heterothallic (Böhm *et al.* 2013; reported as *P. chrysogenum*, Houbraken *et al.* 2014a). ITS barcode: JX997057 (alternative markers: *BenA* = JF909949; *CaM* = JX996263; *RPB2* = JX996658).

***Penicillium rubidurum*** Udagawa & Y. Horie, Trans. Mycol. Soc. Japan 14: 381. 1973. [MB319295]. — Type: NHL 6460. Ex-type: CBS 609.73 = NRRL 6033 = ATCC 28051 = ATCC 48238 = FRR 1558 = IMI 228551 = NHL 6460. Infragen. class: subgen. *Aspergilloides*, sect. *Exilicaulis*, ser. *Erubescentia*. Reproduction: homothallic. ITS barcode: AF033462 (alternative markers: *BenA* = HQ646574; *CaM* = HQ646585; *RPB2* = JN406545).

***Penicillium rubriannulatum*** L. Cai *et al.*, Cladistics 35: 539. 2018 [2019]. [MB818162]. — Type: HMAS 247732. Ex-type: CGMCC 3.18804 = NN072456 = CBS 144641. Infragen. class: subgen. *Aspergilloides*, sect. *Lanata-Divaricata*, ser. *Dalearum*. Reproduction: asexual. ITS barcode: KY495029 (alternative markers: *BenA* = KY495138; *CaM* = MN969336; *RPB2* = KY495080).

***Penicillium rudallense*** Houbraken *et al.*, Stud. Mycol. 78: 433. 2014. [MB809972]. — Type: CBS H-21867. Ex-type: CBS 138162 = FRR 6085 = DTO 056-I4. Infragen. class: subgen. *Aspergilloides*, sect. *Aspergilloides*, ser. *Glabra*. Reproduction: asexual. ITS barcode: KM189504 (alternative markers: *BenA* = KM088741; *CaM* = KM089126; *RPB2* = KM089513).

***Penicillium sacculum*** E. Dale, Ann. Mycol. 24: 137. 1926. [MB277209]. — Type: CBS 231.61. Ex-type: CBS 231.61 = ATCC 18350 = IFO 8114 = IFO 9454 = IMI 051498 = LSHBBB298 = UC4505. Infragen. class: subgen. *Penicillium*, sect. *Eladia*, ser. *Eladia*. Reproduction: asexual. ITS barcode: KC411707 (alternative markers: *BenA* = KJ834488; *CaM* = KU896849; *RPB2* = JN121462).

***Penicillium sajarovii*** Quintan., Av. Aliment. Majora Anim. 22: 539. 1981. [MB114172]. — Type: CBS 277.83. Ex-type: CBS 277.83 = DTO 334-D4 = CECT 2751 = IMI 259992. Infragen. class: subgen. *Penicillium*, sect. *Ramosum*, ser. *Raistrickiorum*. Reproduction: asexual. ITS barcode: KC411724 (alternative markers: *BenA* = MN969397; *CaM* = MN969295; *RPB2* = JN406588).

***Penicillium salamii*** G. Perrone *et al.*, Int. J. Food Microb. 193: 93. 2014. [MB809645]. — Type: CBS H-21341. Ex-type: CBS 135391 = DTO 198-E1 = ITEM 15291. Infragen. class: subgen. *Penicillium*, sect. *Brevicompacta*, ser. *Olsoniorum*. Reproduction: asexual. ITS barcode: HG514431 (alternative markers: *BenA* = HG514437; *CaM* = HG514432; *RPB2* = MN969160).

***Penicillium salmoniflumine*** S.W. Peterson *et al.*, PLoS ONE 10: 0121987, 21. 2015. [MB807374]. — Type: BPI 881286. Ex-type: NRRL 35837 = IBT 29673. Infragen. class: subgen. *Aspergilloides*, sect. *Cinnamopurpurea*, ser. *Idahoensia*. Reproduction: asexual. ITS barcode: KF932960 (alternative markers: *BenA* = KF932928; *CaM* = KF932945; *RPB2* = KF932999).

***Penicillium samsonianum*** L. Wang *et al.*, Persoonia 36: 313. 2016. [MB815873]. — Type: HMAS 245107. Ex-type: AS3.15403 = CBS 138919 = IBT 33392 = DTO 316-B7. Infragen. class: subgen. *Penicillium*, sect. *Osmophila*, ser. *Samsoniorum*. Reproduction: asexual. ITS barcode: KJ668590 (alternative markers: *BenA* = KJ668582; *CaM* = KJ668586; *RPB2* = KT698899).

***Penicillium sanguifluum*** (Sopp) Biourge, Cellule 33: 105. 1923. [MB356682]. Basionym: *Citromyces sanguifluus* Sopp, Skr. Vidensk.-Selsk. Christiana Math.-Nat. Kl. 11: 115. 1912. [MB491120]. — Type: CBS H-20645. Ex-type: CBS 127032 = IBT 29041 = DTO 020-B7. Infragen. class: subgen. *Aspergilloides*, sect. *Citrina*, ser. *Roseopurpurea*. Reproduction: asexual. ITS barcode: JN617681 (alternative markers: *BenA* = JN606819; *CaM* = JN606555; *RPB2* = MN969135).

***Penicillium sanshaense*** X.C. Wang & W.Y. Zhuang, Sci. Rep. 7: 8233, 9. 2017. [MB570337]. — Type: HMAS 248820. Ex-type: CGMCC 3.18413. Infragen. class: subgen. *Aspergilloides*, sect. *Sclerotiorum*, ser. *Herqueorum*. Reproduction: asexual. ITS barcode: KX885070 (alternative markers: *BenA* = KX885050; *CaM* = KX885060; *RPB2* = n.a.).

***Penicillium saturniforme*** (L. Wang & W.Y. Zhuang) Houbraken & Samson, Stud. Mycol. 70: 48. 2011. [MB561958]. Basionym: *Eupenicillium saturniforme* L. Wang & W.Y. Zhuang Mycopathologia 167: 300. 2009. [MB541663]. — Type: AS 3.6886. Ex-type: CBS 122276 = AS 3.6886. Infragen. class: subgen. *Aspergilloides*, sect. *Aspergilloides*, ser. *Saturniformia*. Reproduction: homothallic. ITS barcode: EU644081 (alternative markers: *BenA* = EU644080; *CaM* = EU644062; *RPB2* = JN121439).

***Penicillium scabrosum*** Frisvad *et al.*, Persoonia 14: 177. 1990. [MB136735]. — Type: IMI 285533. Ex-type: CBS 683.89 = FRR 2950 = IBT 3736 = IMI 285533 = DAOM 214786. Infragen. class: subgen. *Penicillium*, sect. *Ramosum*, ser. *Scabrosa*. Reproduction: asexual. ITS barcode: DQ267906 (alternative markers: *BenA* = DQ285610; *CaM* = FJ530987; *RPB2* = JN406541).

***Penicillium sclerotigenum*** W. Yamam., Sci. Rep. Hyogo Univ Agric. 1: 69. 1955. [MB302424]. — Type: IMI 68616. Ex-type: DTO 128-D7 = CBS 101033 = CBS 343.59 = ATCC 18488 = IBT 14346 = IFO 6167 = IMI 068616 = NRRL 3461 = QM 7779. Infragen. class: subgen. *Penicillium*, sect. *Penicillium*, ser. *Sclerotigena*. Reproduction: asexual. ITS barcode: AF033470 (alternative markers: *BenA* = AY674393; *CaM* = KU896850; *RPB2* = JN406652).

***Penicillium sclerotiorum*** J.F.H. Beyma, Zentralbl. Bakteriol. Parasitenk., Abt. 2 96: 418. 1937. [MB277708]. — Type: IMI 40569. Ex-type: CBS 287.36 = ATCC 10494 = IFO 6105 = IMI 040569 = NRRL 2074 = QM 1938 = VKMF-353. Infragen. class: subgen. *Aspergilloides*, sect. *Sclerotiorum*, ser. *Sclerotiorum*. Reproduction: asexual. ITS barcode: JN626132 (alternative markers: *BenA* = JN626001; *CaM* = JN626044; *RPB2* = JN406585).

***Penicillium senticosum*** D.B. Scott, Mycopathol. Mycol. Appl. 36: 5. 1968. [MB335764]. — Type: CBS 316.67. Ex-type: CBS 316.67 = ATCC 18623 = CSIR 1042 = IMI 136211 = IMI 216905. Infragen. class: subgen. *Penicillium*, sect. *Eladia*, ser. *Eladia*. Reproduction: homothallic. ITS barcode: KC411733 (alternative markers: *BenA* = KJ834490; *CaM* = MN969296; *RPB2* = MN969136).

***Penicillium setosum*** Tijith *et al.*, Mycology 10: 55. 2018. [MB818581]. — Type: WSR 62. Ex-type: CBS 144865 = MCC 1370 = NCFT NO 8222.16 = AMH-9974. Infragen. class: subgen. *Aspergilloides*, sect. *Lanata-Divaricata*, ser. *Janthinella*. Reproduction: asexual. ITS barcode: KT852579 (alternative markers: *BenA* = MF184995; *CaM* = MH105905; *RPB2* = MH016196).

***Penicillium shearii*** Stolk & D.B. Scott, Persoonia 4: 396. 1967. [MB335765]. — Type: CBS 290.48. Ex-type: CBS 290.48 = ATCC 10410 = IFO 6088 = IMI 039739 = IMI 039739iv = NRRL 715 = QM 1870. Infragen. class: subgen. *Aspergilloides*, sect. *Citrina*, ser. *Sheariorum*. Reproduction: homothallic. ITS barcode: GU944606 (alternative markers: *BenA* = JN606840; *CaM* = EU644068; *RPB2* = JN121482).

***Penicillium shennongjianum*** [as “*shennonghianum*”] H.Z. Kong & Z.T. Qi, Mycosystema 1: 110. 1988. [MB587562]. — Type: CBS 228.89. Ex-type: CBS 228.89. Infragen. class: subgen. *Aspergilloides*, sect. *Cinnamopurpurea*, ser. *Nodula*. Reproduction: asexual. ITS barcode: KC411705 (alternative markers: *BenA* = KJ834491; *CaM* = AY678561; *RPB2* = JN121458).

***Penicillium simile*** Davolos *et al.*, Int. J. Syst. Evol. Microbiol. 62: 457. 2012. [MB509645]. — Type: ATCC MYA-4591. Ex-type: CBS 129191 = ATCC MYA-4591 = DTO 159-F7. Infragen. class: subgen. *Penicillium*, sect. *Ramosum*, ser. *Raistrickiorum*. Reproduction: asexual. ITS barcode: FJ376592 (alternative markers: *BenA* = FJ376595; *CaM* = GQ979710; *RPB2* = MN969137).

***Penicillium simplicissimum*** (Oudem.) Thom, Penicillia: 335. 1930. [MB278201]. Basionym: *Spicaria simplicissima* Oudem., Ned. Kruidk. Arch. 2: 763. 1902. [MB245011]. — Type: CUP Jensen. 1912: No. 5921 (CUP). Ex-type: CBS 372.48 = DTO 014-H2 = ATCC 10495 = FRR 902 = IFO 5762 = IMI 039816 = QM 1939. Infragen. class: subgen. *Aspergilloides*, sect. *Lanata-Divaricata*, ser. *Simplicissima*. Reproduction: asexual. ITS barcode: GU981588 (alternative markers: *BenA* = GU981632; *CaM* = MN969297; *RPB2* = JN121507).

***Penicillium sinaicum*** Udagawa & S. Ueda, Mycotaxon 14: 266. 1982. [MB110862]. — Type: NHL 2894. Ex-type: CBS 279.82 = NHL 2894. Infragen. class: subgen. *Penicillium*, sect. *Chrysogena*, ser. *Crustacea*. Reproduction: homothallic. ITS barcode: JX997090 (alternative markers: *BenA* = KU896818; *CaM* = JX996970; *RPB2* = JN406587).

***Penicillium singorense*** Visagie *et al.*, Stud. Mycol. 78: 119. 2014. [MB809182]. — Type: CBS H-21802. Ex-type: CBS 138214 = DTO 133-C6. Infragen. class: subgen. *Aspergilloides*, sect. *Lanata-Divaricata*, ser. *Dalearum*. Reproduction: asexual. ITS barcode: KJ775674 (alternative markers: *BenA* = KJ775167; *CaM* = KJ775403; *RPB2* = MN969138).

***Penicillium sizovae*** Baghd., Novosti Sist. Nizsh. Rast. 1968: 103. 1968. [MB335767]. — Type: CBS 413.69. Ex-type: CBS 413.69 = DTO 023-A7 = FRR 518 = IMI 140344 = VKMF-1073. Infragen. class: subgen. *Aspergilloides*, sect. *Citrina*, ser. *Citrina*. Reproduction: asexual. ITS barcode: GU944588 (alternative markers: *BenA* = GU944535; *CaM* = MN969298; *RPB2* = JN606603).

***Penicillium skrjabinii*** Schmotina & Golovleva, Mikol. Fitopatol. 8: 530. 1974. [MB319296]. — Type: IMI 196528. Ex-type: CBS 439.75 = DTO 095-C8 = NRRL 13055 = FRR 1945 = IMI 196528 = VKMF-1940. Infragen. class: subgen. *Aspergilloides*, sect. *Lanata-Divaricata*, ser. *Simplicissima*. Reproduction: asexual. ITS barcode: GU981576 (alternative markers: *BenA* = GU981626; *CaM* = MN969299; *RPB2* = EU427252).

***Penicillium smithii*** Quintan., Av. Aliment. Majora Anim. 23: 340. 1982. [MB114173]. — Type: CBS 276.83. Ex-type: CBS 276.83 = CECT 2744 = IMI 259693. Infragen. class: subgen. *Aspergilloides*, sect. *Exilicaulis*, ser. *Lapidosa*. Reproduction: asexual. ITS barcode: KC411723 (alternative markers: *BenA* = KJ834492; *CaM* = KP016806; *RPB2* = JN406589).

***Penicillium soliforme*** L. Cai *et al.*, Cladistics 35: 540. 2018 [2019]. [MB818158]. — Type: HMAS 247733. Ex-type: CGMCC 3.18806 = NN072519 = CBS 144482. Infragen. class: subgen. *Aspergilloides*, sect. *Lanata-Divaricata*, ser. *Rolfsiorum*. Reproduction: asexual. ITS barcode: KY495038 (alternative markers: *BenA* = KY495147; *CaM* = MN969337; *RPB2* = KY495047).

***Penicillium solitum*** Westling, Ark. Bot. 11: 65. 1911. [MB206172]. — Type: CBS 424.89. Ex-type: DTO 248-E4 = DTO 047-B2 = CBS 424.89 = ATCC 9923 = CBS 288.36 = FRR 937 = IBT 3948 = IFO 7765 = IMI 039810 = IMI 092225 = LSHBP 52 = MUCL 28668 = MUCL 29173 = NRRL 937. Infragen. class: subgen. *Penicillium*, sect. *Fasciculata*, ser. *Camembertiorum*. Reproduction: asexual. ITS barcode: AY373932 (alternative markers: *BenA* = MN969398; *CaM* = KU896851; *RPB2* = KU904363).

***Penicillium soosanum*** Kubátová *et al.*, Mycol. Prog. 18 (1-2): 223. 2018. [MB824353]. — Type: PRM 861478. Ex-type: CCF 3778 = MH 344 = CBS 140106 = IBT 30727. Infragen. class: subgen. *Aspergilloides*, sect. *Lanata-Divaricata*, ser. *Oxalica*. Reproduction: asexual. ITS barcode: FJ430745 (alternative markers: *BenA* = FM865811; *CaM* = LT970913; *RPB2* = LT797561).

***Penicillium soppii*** K.W. Zaleski, Bull. Int. Acad. Polon. Sci., Cl. Sci. Math., Sér. B., Sci. Nat. 1927: 476. 1927. [MB121424]. — Type: IMI 40217. Ex-type: CBS 226.28 = ATCC 10496 = FRR 2023 = IFO 7766 = IMI 040217 = MUCL 29233 = NRRL 2023 = QM 1964 = IBT 18220. Infragen. class: subgen. *Penicillium*, sect. *Ramosum*, ser. *Soppiorum*. Reproduction: asexual. ITS barcode: AF033488 (alternative markers: *BenA* = MN969399; *CaM* = KJ867002; *RPB2* = JN406606).

***Penicillium spathulatum*** Frisvad & Samson, FEMS Microbiol. Lett. 339: 88. 2013. [MB492650]. — Type: CBS 117192. Ex-type: DTO 187-D8 = CBS 117192 = IBT 22220. Infragen. class: subgen. *Penicillium*, sect. *Brevicompacta*, ser. *Buchwaldiorum*. Reproduction: asexual. ITS barcode: JX313165 (alternative markers: *BenA* = MN969400; *CaM* = JX313149; *RPB2* = JN406636).

***Penicillium speluncae*** Visagie & N. Yilmaz, Fungal Syst. Evol. 5: 10. 2020. [MB828614]. — Type: DAOM 745788. Ex-type: DAOMC 251701 = KAS 7512 = P06201. Infragen. class: subgen. *Penicillium*, sect. *Fasciculata*, ser. *Camembertiorum*. Reproduction: asexual. ITS barcode: MG490869 (alternative markers: *BenA* = MG490889; *CaM* = MG490959; *RPB2* = MN170741).

***Penicillium spinuliferum*** L. Cai & X.Z. Jiang, Cladistics 35: 542. 2018 [2019]. [MB818153]. — Type: HMAS 247734. Ex-type: CGMCC 3.18807 = NN072545 = CBS 144483. Infragen. class: subgen. *Aspergilloides*, sect. *Lanata-Divaricata*, ser. *Simplicissima*. Reproduction: asexual. ITS barcode: KY495040 (alternative markers: *BenA* = KY495149; *CaM* = MN969338; *RPB2* = KY495090).

***Penicillium spinulosum*** Thom, U.S.D.A. Bur. Animal Industr. Bull. 118: 76. 1910. [MB215401]. — Type: IMI 24316i. Ex-type: CBS 374.48 = ATCC 10498 = FRR 1750 = IMI 024316 = LSHBAd 29 = MUCL 13910 = MUCL 13911 = NCTC 591 = NRRL 1750 = QM 7654. Infragen. class: subgen. *Aspergilloides*, sect. *Aspergilloides*, ser. *Spinulosa*. Reproduction: asexual. ITS barcode: AF033410 (alternative markers: *BenA* = KJ834493; *CaM* = GQ367524; *RPB2* = JN406558).

***Penicillium steckii*** K.W. Zaleski, Bull. Int. Acad. Polon. Sci., Sér. B., Sci. Nat. 1927: 469. 1927. [MB278769]. — Type: IMI 40583. Ex-type: CBS 260.55 = DTO 022-G5 = ATCC 10499 = CECT 2268 = DSM1252 = IMI 040583 = NRRL 2140 = QM 6413. Infragen. class: subgen. *Aspergilloides*, sect. *Citrina*, ser. *Citrina*. Reproduction: asexual. ITS barcode: GU944597 (alternative markers: *BenA* = GU944522; *CaM* = MN969300; *RPB2* = JN606602).

***Penicillium sterculiniicola*** Houbraken, Stud. Mycol. 78: 436. 2014 [MB809973]. — Type: CBS H-21877. Ex-type: CBS 122426 = DTO 031-A4. Infragen. class: subgen. *Aspergilloides*, sect. *Aspergilloides*, ser. *Spinulosa*. Reproduction: asexual. ITS barcode: KM189464 (alternative markers: *BenA* = KM088693; *CaM* = KM089078; *RPB2* = KM089465).

***Penicillium stolkiae*** D.B. Scott, Mycopathol. Mycol. Appl. 36: 8. 1968. [MB335768]. — Type: CBS 315.67. Ex-type: CBS 315.67 = ATCC 18546 = CSIR 1041 = FRR 534 = IMI 136210 = NRRL 5816. Infragen. class: subgen. *Aspergilloides*, sect. *Stolkia*, ser. *Stolkia*. Reproduction: homothallic. ITS barcode: AF033444 (alternative markers: *BenA* = JN617717; *CaM* = AF481135; *RPB2* = JN121488).

***Penicillium striatisporum*** Stolk, Antonie van Leeuwenhoek 35: 268. 1969. [MB335769]. — Type: CBS 705.68. Ex-type: CBS 705.68 = ATCC 22052 = CCRC 31679 = FRR 827 = IMI 151749 = MUCL 31202. Infragen. class: subgen. *Aspergilloides*, sect. *Exilicaulis*, ser. *Erubescentia*. Reproduction: asexual. ITS barcode: AF038938 (alternative markers: *BenA* = MN969401; *CaM* = KP016807; *RPB2* = JN406538).

***Penicillium subarcticum*** S.W. Peterson & Sigler, Mycol. Res. 106: 1116. 2002. [MB483983]. — Type: BPI 841397. Ex-type: CBS 111719 = NRRL 31108 = UAMH 3897. Infragen. class: subgen. *Aspergilloides*, sect. *Stolkia*, ser. *Stolkia*. Reproduction: asexual. ITS barcode: AF481120 (alternative markers: *BenA* = JN617716; *CaM* = AF481141; *RPB2* = MN969139).

***Penicillium sublectaticum*** Houbraken *et al.*, Stud. Mycol. 78: 436. 2014. [MB809974]. — Type: CBS H-21955. Ex-type: CBS 138217= DTO 244-G2. Infragen. class: subgen. *Aspergilloides*, sect. *Aspergilloides*, ser. *Sublectatica*. Reproduction: asexual. ITS barcode: KM189761 (alternative markers: *BenA* = KM089010; *CaM* = KM089397; *RPB2* = KM089784).

***Penicillium subrubescens*** Houbraken *et al.*, Antonie van Leeuwenhoek 103: 1354. 2013. [MB801306]. — Type: CBS H-21029. Ex-type: CBS 132785 = DTO 188-D6 = FBCC 1632 = IBT 31985. Infragen. class: subgen. *Aspergilloides*, sect. *Lanata-Divaricata*, ser. *Rolfsiorum*. Reproduction: asexual. ITS barcode: KC346350 (alternative markers: *BenA* = KC346327; *CaM* = KC346330; *RPB2* = KC346306).

***Penicillium subspinulosum*** Houbraken, Stud. Mycol. 78: 436. 2014. [MB809975]. — Type: CBS H-21856. Ex-type: CBS 137946 = DTO 041-F2. Infragen. class: subgen. *Aspergilloides*, sect. *Aspergilloides*, ser. *Spinulosa*. Reproduction: asexual. ITS barcode: KM189483 (alternative markers: *BenA* = KM088719; *CaM* = KM089104; *RPB2* = KM089491).

***Penicillium subturcoseum*** Visagie & K. Jacobs, IMA Fungus 7: 105. 2016. [MB811008]. — Type: CBS H-22041. Ex-type: CBS 139132 = DAOMC 241096 = DTO 180-C9 = CV 2835. Infragen. class: subgen. *Aspergilloides*, sect. *Exilicaulis*, ser. *Corylophila*. Reproduction: asexual. ITS barcode: FJ231006 (alternative markers: *BenA* = JX141161; *CaM* = JX157532; *RPB2* = KP064674).

***Penicillium sucrivorum*** Visagie & K. Jacobs, Mycologia 106: 546. 2014. [MB804723]. — Type: CBS H-21331. Ex-type: CBS 135116 = DAOM 241042 = DTO 183-E5. Infragen. class: subgen. *Aspergilloides*, sect. *Citrina*, ser. *Westlingiorum*. Reproduction: asexual. ITS barcode: JX140872 (alternative markers: *BenA* = JX141015; *CaM* = JX141506; *RPB2* = MN969140).

***Penicillium sumatraense*** [as “*sumatrense*”] Szilvinyi, Archiv. Hydrobiol.14 Suppl. 6: 535. 1936. [MB319297]. — Type: CBS 281.36. Ex-type: CBS 281.36 = DTO 022-F1 = NRRL 779 = FRR 779. Infragen. class: subgen. *Aspergilloides*, sect. *Citrina*, ser. *Sumatraensia*. Reproduction: asexual. ITS barcode: GU944578 (alternative markers: *BenA* = JN606639; *CaM* = MN969301; *RPB2* = EF198541).

***Penicillium svalbardense*** Frisvad *et al.*, Antonie van Leeuwenhoek 92: 48. 2007. [MB529943]. — Type: EX-F 1307. Ex-type: CBS 122416 = IBT 23856 = DTO 048-D5 = EXF-1307. Infragen. class: subgen. *Aspergilloides*, sect. *Lanata-Divaricata*, ser. *Rolfsiorum*. Reproduction: asexual. ITS barcode: GU981603 (alternative markers: *BenA* = DQ486644; *CaM* = KC346338; *RPB2* = KF296457).

***Penicillium swiecickii*** K.W. Zaleski, Bull. Int. Acad. Polon. Sci., Sér. B., Sci. Nat. 1927: 474. 1927. [MB534781]. — Type: unknown. Ex-type: CBS 119391 = FRR 918 = IBT 27865 = IMI 191500 = NRRL 918. Infragen. class: subgen. *Penicillium*, sect. *Ramosum*, ser. *Lanosa*. Reproduction: asexual. ITS barcode: AF033490 (alternative markers: *BenA* = KJ834494; *CaM* = KJ866993; *RPB2* = JN406635).

***Penicillium synnematicola*** Guevara-Suarez *et al.*, Fungal Syst. Evol. 5: 62. 2020. [MB822072]. — Type: CBS H-23132. Ex-type: CBS 142669 = FMR 15192. Infragen. class: subgen. *Penicillium*, sect. *Robsamsonia*, ser. *Glandicolarum*. Reproduction: asexual. ITS barcode: LT898167 (alternative markers: *BenA* = LT898172; *CaM* = LT898137; *RPB2* = LT898142).

***Penicillium taiwanense*** (Matsushima) Houbraken & Samson, Stud. Mycol. 70: 48. 2011. [MB561969]. Basionym: *Phialomyces taiwanensis* Matsush., Matsushima Mycological Memoirs 4: 12. 1985. [MB105680]. — Type: unknown. Ex-type: n.a. Infragen. class: subgen. *Aspergilloides*, sect. *Thysanophora*, ser. *Thysanophora*. Reproduction: asexual. ITS barcode: n.a. (alternative markers: *BenA* = n.a.; *CaM* = n.a.; *RPB2* = n.a.).

***Penicillium tanzanicum*** Visagie *et al.*, Persoonia 36: 278. 2016. [MB815781]. — Type: DAOM 695766. Ex-type: DAOMC 250514 = CBS 140968 = DTO 410-D3 = 50.118 = KAS 1946. Infragen. class: subgen. *Aspergilloides*, sect. *Lanata-Divaricata*, ser. *Simplicissima*. Reproduction: asexual. ITS barcode: KT887841 (alternative markers: *BenA* = KT887802; *CaM* = KT887763; *RPB2* = MN969183).

***Penicillium tardochrysogenum*** Frisvad *et al.*, Persoonia 29: 93. 2012. [MB801877]. — Type: CBS H-21057. Ex-type: DTO 149-B9 = CBS 132200 = IBT 30075. Infragen. class: subgen. *Penicillium*, sect. *Chrysogena*, ser. *Chrysogena*. Reproduction: asexual. ITS barcode: JX997028 (alternative markers: *BenA* = JX996898; *CaM* = JX996239; *RPB2* = JX996634).

***Penicillium taxi*** R. Schneid., Zentralbl. Bakteriol. Parasitenk., Abt. 2 110: 43. 1956. [MB282799]. — Type: unknown. Ex-type: CBS 206.57 = ATCC 18484 = BBA 7480 = MUCL 11402 = QM 8153. Infragen. class: subgen. *Aspergilloides*, sect. *Thysanophora*, ser. *Thysanophora*. Reproduction: asexual. ITS barcode: KJ834517 (alternative markers: *BenA* = KJ834495; *CaM* = MN969302; *RPB2* = JN121454).

***Penicillium terrarumae*** Houbraken *et al.*, Phytotaxa 273: 170. 2016. [MB801431]. — Type: HGUPd2020. Ex-type: HGUP2025 = CBS 131811 = DTO 174-H2. Infragen. class: subgen. *Aspergilloides*, sect. *Lanata-Divaricata*, ser. *Rolfsiorum*. Reproduction: asexual. ITS barcode: MN431397 (alternative markers: *BenA* = KX650295; *CaM* = MN969323; *RPB2* = MN969185).

***Penicillium terrenum*** D.B. Scott, Mycopathol. Mycol. Appl. 36: 1. 1968. [MB335771]. — Type: CBS 313.67. Ex-type: CBS 313.67 = ATCC 18547 = CSIR 1022 = IMI 136208. Infragen. class: subgen. *Aspergilloides*, sect. *Exilicaulis*, ser. *Lapidosa*. Reproduction: homothallic. ITS barcode: AM992111 (alternative markers: *BenA* = KJ834496; *CaM* = KP016808; *RPB2* = JN406577).

***Penicillium terrigenum*** Seifert *et al.*, Stud. Mycol. 70: 125. 2011. [MB563204]. — Type: CBS H-20667. Ex-type: CBS 127354 = IBT 30769. Infragen. class: subgen. *Aspergilloides*, sect. *Citrina*, ser. *Copticolarum*. Reproduction: asexual. ITS barcode: JN617684 (alternative markers: *BenA* = JN606810; *CaM* = JN606583; *RPB2* = JN606600).

***Penicillium thiersii*** S.W. Peterson *et al.*, Mycologia 96: 1283. 2004. [MB487738]. — Type: BPI 842269. Ex-type: CBS 117503 = IBT 27050 = NRRL 28162. Infragen. class: subgen. *Aspergilloides*, sect. *Aspergilloides*, ser. *Thiersiorum*. Reproduction: homothallic (Houbraken *et al.* 2014b). ITS barcode: AF125936 (alternative markers: *BenA* = KJ834497; *CaM* = AY741726; *RPB2* = JN121434).

***Penicillium thomii*** Maire, Bull. Soc. Hist. Nat. Afrique N. 8: 189. 1917. [MB202819]. — Type: IMI 189694. Ex-type: CBS 225.81 = IMI 189694 = NRRL 2077. Infragen. class: subgen. *Aspergilloides*, sect. *Aspergilloides*, ser. *Thomiorum*. Reproduction: asexual. ITS barcode: KM189560 (alternative markers: *BenA* = KM088799; *CaM* = KM089184; *RPB2* = KM089571).

***Penicillium thymicola*** Frisvad & Samson, Stud. Mycol. 49: 29. 2004. [MB370969]. — Type: CBS 111225. Ex-type: CBS 111225 = IBT 5891. Infragen. class: subgen. *Penicillium*, sect. *Fasciculata*, ser. *Verrucosa*. Reproduction: asexual. ITS barcode: KJ834518 (alternative markers: *BenA* = MN969402; *CaM* = FJ530990; *RPB2* = KU904364).

***Penicillium tricolor*** Frisvad *et al.*, Canad. J. Bot. 72: 937. 1994. [MB541710]. — Type: DAOM 216240. Ex-type: DTO 157-A4 = CBS 635.93 = IBT 12493 = DAOM 216240. Infragen. class: subgen. *Penicillium*, sect. *Fasciculata*, ser. *Viridicata*. Reproduction: asexual. ITS barcode: JN942704 (alternative markers: *BenA* = MN969403; *CaM* = KU896852; *RPB2* = JN985422).

***Penicillium tropicoides*** Houbraken *et al.*, Fungal Divers. 44: 127. 2010. [MB518293]. — Type: CBS 122410. Ex-type: CBS 122410 = DTO 010-C4 = IBT 29043. Infragen. class: subgen. *Aspergilloides*, sect. *Citrina*, ser. *Citrina*. Reproduction: homothallic. ITS barcode: GU944584 (alternative markers: *BenA* = GU944531; *CaM* = MN969303; *RPB2* = JN606608).

***Penicillium tropicum*** Houbraken *et al.*, Fungal Divers. 44: 129. 2010. [MB518294]. — Type: SC42-1. Ex-type: CBS 112584 = DTO 031-B1 = IBT 24580. Infragen. class: subgen. *Aspergilloides*, sect. *Citrina*, ser. *Citrina*. Reproduction: homothallic. ITS barcode: GU944582 (alternative markers: *BenA* = GU944532; *CaM* = MN969304; *RPB2* = JN606607).

***Penicillium trzebinskii*** K.W. Zaleski, Bull. Int. Acad. Polon. Sci., Sér. B., Sci. Nat. 1927: 498. 1927. [MB280795]. — Type: unknown. Ex-type: CBS 382.48 = ATCC 10507 = FRR 731 = IFO 6110 = IMI 039749 = MUCL 29102 = NRRL 731 = QM 7678. Infragen. class: subgen. *Aspergilloides*, sect. *Aspergilloides*, ser. *Spinulosa*. Reproduction: asexual. ITS barcode: KM189784 (alternative markers: *BenA* = KM089034; *CaM* = KM089421; *RPB2* = KM089808).

***Penicillium tsitsikammaense*** Houbraken, Stud. Mycol. 78: 440. 2014. [MB809976]. — Type: CBS H-21881. Ex-type: CBS 328.71 = DTO 006-I3 = CSIR 1092. Infragen. class: subgen. *Aspergilloides*, sect. *Aspergilloides*, ser. *Pinetorum*. Reproduction: homothallic (Stolk & Samson 1983:127, CBS 328.71). ITS barcode: KM189451 (alternative markers: *BenA* = KM088675; *CaM* = KM089060; *RPB2* = KM089447).

***Penicillium tubakianum*** Visagie & Samson, Persoonia 36: 151. 2016. [MB808270]. — Type: CBS H-21604. Ex-type: CBS 287.66 = DTO 138-D9 = MUCL 8519 = IFO 8315. Infragen. class: subgen. *Aspergilloides*, sect. *Torulomyces*, ser. *Torulomyces*. Reproduction: asexual. ITS barcode: KF303652 (alternative markers: *BenA* = KF303611; *CaM* = KF303637; *RPB2* = KF303712).

***Penicillium tularense*** Paden, Mycopathol. Mycol. Appl. 43: 264. 1971. [MB319298]. — Type: UVIC JWP 68-31. Ex-type: CBS 430.69 = ATCC 22056 = FRR 899 = IFO 31740 = IMI 148394 = NRRL 5273 = AS 3.14006. Infragen. class: subgen. *Penicillium*, sect. *Brevicompacta*, ser. *Tularensia*. Reproduction: homothallic. ITS barcode: AF033487 (alternative markers: *BenA* = KC427175; *CaM* = JX313135; *RPB2* = JN121516).

***Penicillium tulipae*** Overy & Frisvad, Syst. Appl. Microbiol. 26: 634. 2003. [MB488954]. — Type: C 60162. Ex-type: CBS 109555 = CBS 187.88 = IBT 3458. Infragen. class: subgen. *Penicillium*, sect. *Fasciculata*, ser. *Corymbifera*. Reproduction: asexual. ITS barcode: KJ834519 (alternative markers: *BenA* = MN969404; *CaM* = MN969305; *RPB2* = MN969141).

***Penicillium tunisiense*** S. Ouhibi *et al.*, Int. J. Syst. Evol. Microbiol. 68: 3224. 2018. [MB823626]. — Type: MUM-H 17.62. Ex-type: MUM 17.62 = ITEM 17445. Infragen. class: subgen. *Penicillium*, sect. *Ramosum*, ser. *Soppiorum*. Reproduction: asexual. ITS barcode: MG586956 (alternative markers: *BenA* = MG586970; *CaM* = MG586974; *RPB2* = n.a.).

***Penicillium turbatum*** Westling, Ark. Bot. 11: 128. 1911. [MB202895]. — Type: IMI 39738. Ex-type: CBS 383.48 = CBS 237.60 = ATCC 9782 = DSM2426 = FRR 757 = IFO 7767 = IMI 039738 = MUCL 29115 = NRRL 757 = NRRL 758 = QM 1941. Infragen. class: subgen. *Penicillium*, sect. *Turbata*, ser. *Turbata*. Reproduction: homothallic (Scott and Stolk 1967; as E. baarnense). ITS barcode: AF034454 (alternative markers: *BenA* = KJ834499; *CaM* = KU896853; *RPB2* = JN406556).

***Penicillium turcosoconidiatum*** Visagie *et al.*, Stud. Mycol. 78: 440. 2014. [MB809977]. — Type: CBS H-21876. Ex-type: CBS 138557 = DTO 181-A3 = CV 110 = DAOM 241130. Infragen. class: subgen. *Aspergilloides*, sect. *Aspergilloides*, ser. *Pinetorum*. Reproduction: asexual. ITS barcode: KM189645 (alternative markers: *BenA* = KM088889; *CaM* = KM089276; *RPB2* = KM089663).

***Penicillium ubiquetum*** Houbraken *et al.*, Stud. Mycol. 70: 127. 2011. [MB563201]. — Type: CBS H-20659. Ex-type: CBS 126437 = DTO 078-B5 = IBT 22226. Infragen. class: subgen. *Aspergilloides*, sect. *Citrina*, ser. *Westlingiorum*. Reproduction: asexual. ITS barcode: JN617680 (alternative markers: *BenA* = JN606800; *CaM* = MN969306; *RPB2* = MN969142).

***Penicillium ulaiense*** H.M. Hsieh *et al.*, Trans. Mycol. Soc. Rep. China 2: 161. 1987. [MB126489]. — Type: PPEH 29001.87. Ex-type: CBS 210.92 = CBS 261.94 = CCRC 32655 = IBT 18387 = IBT 23037. Infragen. class: subgen. *Penicillium*, sect. *Penicillium*, ser. *Italica*. Reproduction: asexual. ITS barcode: KC411695 (alternative markers: *BenA* = AY674408; *CaM* = KU896854; *RPB2* = KU904365).

***Penicillium uruguayense*** Guevara-Suarez *et al.*, Persoonia 39: 323. 2017. [MB822920]. — Type: FMR H-14490. Ex-type: CBS 143247 = FMR 14490 = DTO 410-E9. Infragen. class: subgen. *Aspergilloides*, sect. *Lanata-Divaricata*, ser. *Janthinella*. Reproduction: homothallic. ITS barcode: LT904729 (alternative markers: *BenA* = LT904699; *CaM* = LT904698; *RPB2* = MN969200).

***Penicillium vagum*** Houbraken *et al.*, Stud. Mycol. 78: 443. 2014. [MB809978]. — Type: CBS H-21926. Ex-type: CBS 137728 = DTO 180-G3 = CV 25 = DAOM 241357. Infragen. class: subgen. *Aspergilloides*, sect. *Aspergilloides*, ser. *Longicatenata*. Reproduction: asexual. ITS barcode: KM189642 (alternative markers: *BenA* = KM088886; *CaM* = KM089273; *RPB2* = KM089660).

***Penicillium valentinum*** C. Ramírez & A.T. Martínez, Mycopathologia 72: 183. 1980. [MB113027]. — Type: IJFM 5071. Ex-type: CBS 172.81 = ATCC 42227 = IJFM 5071. Infragen. class: subgen. *Aspergilloides*, sect. *Aspergilloides*, ser. *Thomiorum*. Reproduction: asexual. ITS barcode: KM189550 (alternative markers: *BenA* = KM088788; *CaM* = KM089173; *RPB2* = KM089560).

***Penicillium vancouverense*** Houbraken *et al.*, Stud. Mycol. 70: 131. 2011. [MB563207]. — Type: CBS H-20646. Ex-type: CBS 126323 = DTO 082-B8 = IBT 20700. Infragen. class: subgen. *Aspergilloides*, sect. *Citrina*, ser. *Westlingiorum*. Reproduction: asexual. ITS barcode: JN617675 (alternative markers: *BenA* = JN606663; *CaM* = MN969307; *RPB2* = MN969143).

***Penicillium vanderhammenii*** Houbraken *et al.*, Int. J. Syst. Evol. Microbiol. 61: 1473. 2011. [MB518027]. — Type: HUA 170337. Ex-type: CBS 126216 = DTO 097-A3 = DTO 297-I2 = IBT 23203. Infragen. class: subgen. *Aspergilloides*, sect. *Lanata-Divaricata*, ser. *Dalearum*. Reproduction: homothallic. ITS barcode: GU981574 (alternative markers: *BenA* = GU981647; *CaM* = MN969308; *RPB2* = KF296458).

***Penicillium vanluykii*** Frisvad *et al.*, Persoonia 29: 97. 2012. [MB801878]. — Type: CBS H-21059. Ex-type: DTO 148-I2 = CBS 131539 = IBT 14505. Infragen. class: subgen. *Penicillium*, sect. *Chrysogena*, ser. *Chrysogena*. Reproduction: asexual. ITS barcode: JX997007 (alternative markers: *BenA* = JX996879; *CaM* = JX996220; *RPB2* = JX996615).

***Penicillium vanoranjei*** Visagie *et al.*, Persoonia 31: 46. 2013. [MB803782]. — Type: CBS H-21145. Ex-type: CBS 134406. Infragen. class: subgen. *Aspergilloides*, sect. *Sclerotiorum*, ser. *Sclerotiorum*. Reproduction: asexual. ITS barcode: KC695696 (alternative markers: *BenA* = KC695686; *CaM* = KC695691; *RPB2* = n.a.).

***Penicillium variratense*** Visagie & Samson, Persoonia 36: 151. 2016. [MB808271]. — Type: CBS H-21611. Ex-type: CBS 337.97 = DTO 137-C8. Infragen. class: subgen. *Aspergilloides*, sect. *Torulomyces*, ser. *Torulomyces*. Reproduction: asexual. ITS barcode: KF303649 (alternative markers: *BenA* = KF303610; *CaM* = KF303630; *RPB2* = KF303675).

***Penicillium vasconiae*** C. Ramírez & A.T. Martínez, Mycopathologia 72: 189. 1980. [MB113028]. — Type: CBS 339.79. Ex-type: CBS 339.79 = DTO 076-H1 = ATCC 42224 = IJFM 3008. Infragen. class: subgen. *Aspergilloides*, sect. *Lanata-Divaricata*, ser. *Rolfsiorum*. Reproduction: asexual. ITS barcode: GU981599 (alternative markers: *BenA* = GU981653; *CaM* = MN969309; *RPB2* = MN969144).

***Penicillium vascosobrinhous*** R.N. Barbosa & J.D.P. Bezerra, Acta Bot. Bras. 2020. [MB833816]. — Type: URM 94140. Ex-type: URM 8193. Infragen. class: subgen. *Aspergilloides*, sect. *Citrina*, ser. *Euglauca*. Reproduction: asexual. ITS barcode: LR744067 (alternative markers: *BenA* = LR744069; *CaM* = LR744063; *RPB2* = LR744065).

***Penicillium velutinum*** J.F.H. Beyma, Zentralbl. Bakteriol. Parasitenk., Abt. 2 91: 353. 1935. [MB283175]. — Type: IMI 40571. Ex-type: CBS 250.32 = ATCC 10510 = CECT 2318 = IJFM 5108 = IMI 040571 = NRRL 2069 = QM 7686 = VKMF-379. Infragen. class: subgen. *Aspergilloides*, sect. *Exilicaulis*, ser. *Lapidosa*. Reproduction: asexual. ITS barcode: AF033448 (alternative markers: *BenA* = JX141170; *CaM* = MT478037; *RPB2* = KP064682).

***Penicillium venetum*** (Frisvad) Frisvad, Int. Mod. Meth. Pen. Asp. Clas.: 275. 2000. [MB459816]. Basionym: *Penicillium hirsutum* var. *venetum* Frisvad, Mycologia 81: 856. 1990. [MB126414]. — Type: IMI 321520. Ex-type: IBT 10661 = IMI 321520. Infragen. class: subgen. *Penicillium*, sect. *Fasciculata*, ser. *Corymbifera*. Reproduction: asexual. ITS barcode: AJ005485 (alternative markers: *BenA* = AY674335; *CaM* = KU896855; *RPB2* = KU904366).

***Penicillium verhagenii*** Houbraken, Stud. Mycol. 78: 443. 2014. [MB809979]. — Type: CBS H-21865. Ex-type: CBS 137959 = DTO 193-A1. Infragen. class: subgen. *Aspergilloides*, sect. *Aspergilloides*, ser. *Verhageniorum*. Reproduction: asexual. ITS barcode: KM189708 (alternative markers: *BenA* = KM088955; *CaM* = KM089342; *RPB2* = KM089729).

***Penicillium verrucisporum*** X.C. Wang & W.Y. Zhuang, Sci. Rep. 7: 8233, 10. 2017. [MB570339]. — Type: HMAS 248819. Ex-type: CGMCC 3.18415. Infragen. class: subgen. *Aspergilloides*, sect. *Sclerotiorum*, ser. *Herqueorum*. Reproduction: asexual. ITS barcode: KX885069 (alternative markers: *BenA* = KX885049; *CaM* = KX885059; *RPB2* = KX885040).

***Penicillium verrucosum*** Dierckx, Ann. Soc. Sci. Bruxelles 25: 88. 1901. [MB212252]. — Type: IMI 200310. Ex-type: CBS 603.74 = ATCC 48957 = ATHUM 2897 = CECT 2906 = FRR 965 = IBT 12809 = IBT 4733 = IMI 200310 = IMI 200310ii = MUCL 28674 = MUCL 29089 = MUCL 29186 = NRRL 965. Infragen. class: subgen. *Penicillium*, sect. *Fasciculata*, ser. *Verrucosa*. Reproduction: asexual. ITS barcode: AY373938 (alternative markers: *BenA* = MN969405; *CaM* = DQ911138; *RPB2* = JN121539).

***Penicillium vinaceum*** J.C. Gilman & E.V. Abbott, Iowa St. Coll. J. Sci. 1: 299. 1927. [MB281754]. — Type: IMI 29189. Ex-type: CBS 389.48 = ATCC 10514 = FRR 739 = IMI 029189 = NRRL 739 = QM 6746. Infragen. class: subgen. *Aspergilloides*, sect. *Exilicaulis*, ser. *Erubescentia*. Reproduction: asexual. ITS barcode: AF033461 (alternative markers: *BenA* = HQ646575; *CaM* = HQ646586; *RPB2* = JN406555).

***Penicillium virgatum*** Nirenberg & Kwasna, Mycol. Res. 109: 977. 2005. [MB341488]. — Type: BBA 65745. Ex-type: CBS 114838 = BBA 65745. Infragen. class: subgen. *Penicillium*, sect. *Ramosum*, ser. *Virgata*. Reproduction: asexual. ITS barcode: AJ748692 (alternative markers: *BenA* = KJ834500; *CaM* = KJ866992; *RPB2* = JN406641).

***Penicillium viridicatum*** Westling, Ark. Bot. 11: 88. 1911. [MB163349]. — Type: IMI 39758ii. Ex-type: DTO 005-C9 = CBS 390.48 = ATCC 10515 = IBT 23041 = IFO 7736 = IMI 039758 = IMI 039758ii = NRRL 963 = QM 7683. Infragen. class: subgen. *Penicillium*, sect. *Fasciculata*, ser. *Viridicata*. Reproduction: asexual. ITS barcode: AY373939 (alternative markers: *BenA* = MN969406; *CaM* = KU896856; *RPB2* = JN121511).

***Penicillium viridissimum*** L. Cai & X.Z. Jiang, Cladistics 35: 543. 2018 [2019]. [MB818160]. — Type: HMAS 247735. Ex-type: CGMCC 3.18796 = NN072081 = CBS 144484. Infragen. class: subgen. *Aspergilloides*, sect. *Lanata-Divaricata*, ser. *Dalearum*. Reproduction: asexual. ITS barcode: KY495004 (alternative markers: *BenA* = KY495113; *CaM* = MN969339; *RPB2* = KY495059).

***Penicillium viticola*** Nonaka & Masuma, Mycoscience 52: 339. 2011. [MB516048]. — Type: TNS-F38702. Ex-type: JCM 17636 = FKI-4410. Infragen. class: subgen. *Aspergilloides*, sect. *Sclerotiorum*, ser. *Sclerotiorum*. Reproduction: asexual. ITS barcode: AB606414 (alternative markers: *BenA* = AB540174; *CaM* = n.a.; *RPB2* = n.a.).

***Penicillium vulpinum*** (Cooke & Massee) Seifert & Samson, Adv. Pen. Asp. Syst.: 144. 1986 [1985]. [MB114763]. Basionym: *Coremium vulpinum* Cooke & Massee, Grevillea 16: 81. 1888. [MB183683]. — Type: "on dung", s. coll., in herb. Cooke (K). Ex-type: CBS 126.23 = ATCC 10426 = IMI 040237 = NRRL 2031 = VKMF-257. Infragen. class: subgen. *Penicillium*, sect. *Robsamsonia*, ser. *Claviformia*. Reproduction: asexual. ITS barcode: AF506012 (alternative markers: *BenA* = KJ834501; *CaM* = KU896857; *RPB2* = KU904367).

***Penicillium waksmanii*** K.W. Zaleski, Bull. Int. Acad. Polon. Sci., Sér. B., Sci. Nat.: 468. 1927. [MB121677]. — Type: IMI 39746i. Ex-type: CBS 230.28 = DTO 022-E6 = ATCC 10516 = FRR 777 = IFO 7737 = IMI 039746 = IMI 039746i = MUCL 29120 = NRRL 777 = QM 7681. Infragen. class: subgen. *Aspergilloides*, sect. *Citrina*, ser. *Westlingiorum*. Reproduction: asexual. ITS barcode: GU944602 (alternative markers: *BenA* = JN606779; *CaM* = MN969310; *RPB2* = JN606627).

***Penicillium wellingtonense*** A.L.J. Cole *et al.*, Stud. Mycol. 70: 133. 2011. [MB563208]. — Type: CBS H-20657. Ex-type: CBS 130375 = DTO 076-C6 = IBT 23557 = DTO 76C6. Infragen. class: subgen. *Aspergilloides*, sect. *Citrina*, ser. *Westlingiorum*. Reproduction: asexual. ITS barcode: JN617713 (alternative markers: *BenA* = JN606670; *CaM* = MN969311; *RPB2* = JN606616).

***Penicillium westlingii*** K.W. Zaleski, Bull. Int. Acad. Polon. Sci., Sér. B., Sci. Nat. 1927: 473. 1927. [MB282076]. — Type: IMI 92272. Ex-type: CBS 231.28 = DTO 022-E7 = IMI 092272. Infragen. class: subgen. *Aspergilloides*, sect. *Citrina*, ser. *Westlingiorum*. Reproduction: asexual. ITS barcode: GU944601 (alternative markers: *BenA* = JN606718; *CaM* = MN969312; *RPB2* = JN606625).

***Penicillium williamettense*** Visagie & Samson, Persoonia 36: 151. 2016. [MB808272]. — Type: CBS H-21609. Ex-type: CBS 129774 = DTO 208-A4. Infragen. class: subgen. *Aspergilloides*, sect. *Torulomyces*, ser. *Torulomyces*. Reproduction: asexual. ITS barcode: KF303667 (alternative markers: *BenA* = KF303622; *CaM* = KF303639; *RPB2* = KF303709).

***Penicillium wisconsinense*** Visagie & Samson, Persoonia 36: 151. 2016. [MB808273]. — Type: CBS H-21614. Ex-type: CBS 128279 = DTO 198-H7 = WSF 3132. Infragen. class: subgen. *Aspergilloides*, sect. *Torulomyces*, ser. *Torulomyces*. Reproduction: asexual. ITS barcode: KF303670 (alternative markers: *BenA* = KF303624; *CaM* = KF303641; *RPB2* = KF303706).

***Penicillium wollemiicola*** Visagie *et al.*, Persoonia 36: 153. 2016. [MB808274]. — Type: DAOM 675862. Ex-type: CBS 137177 = DTO 297-E3. Infragen. class: subgen. *Aspergilloides*, sect. *Torulomyces*, ser. *Torulomyces*. Reproduction: homothallic. ITS barcode: KJ174314 (alternative markers: *BenA* = KJ174315; *CaM* = KJ174316; *RPB2* = KJ174313).

***Penicillium wotroi*** Houbraken *et al.*, Int. J. Syst. Evol. Microbiol. 61: 1474. 2011. [MB518026]. — Type: HUA 170336. Ex-type: CBS 118171 = DTO 056-E5 = DTO 297-I3 = IBT 23253. Infragen. class: subgen. *Aspergilloides*, sect. *Lanata-Divaricata*, ser. *Simplicissima*. Reproduction: asexual. ITS barcode: GU981591 (alternative markers: *BenA* = GU981637; *CaM* = MN969313; *RPB2* = KF296460).

***Penicillium xanthomelinii*** Visagie & K. Jacobs, IMA Fungus 7: 105. 2016. [MB811009]. — Type: CBS H-22048. Ex-type: CBS 139163 = DAOMC 241104 = DTO 183-C7 = CV 1677. Infragen. class: subgen. *Aspergilloides*, sect. *Exilicaulis*, ser. *Lapidosa*. Reproduction: asexual. ITS barcode: JX140921 (alternative markers: *BenA* = JX141120; *CaM* = JX157495; *RPB2* = KP064683).

***Penicillium yarmokense*** Baghd., Novosti Sist. Nizsh. Rast. 5: 99. 1968. [MB335774]. — Type: CBS H-7536. Ex-type: CBS 410.69 = FRR 520 = IMI 140346 = VKMF-1076. Infragen. class: subgen. *Penicillium*, sect. *Canescentia*, ser. *Canescentia*. Reproduction: asexual. ITS barcode: KC411757 (alternative markers: *BenA* = MN969407; *CaM* = MN969314; *RPB2* = JN406553).

***Penicillium yezoense*** Hanzawa ex Houbraken, Stud. Mycol. 78: 443. 2014. [MB809980]. — Type: CBS H-21863. Ex-type: CBS 350.59 = ATCC 18333 = FRR 3395 = IFO 5362 = IMI 068615. Infragen. class: subgen. *Aspergilloides*, sect. *Aspergilloides*, ser. *Thomiorum*. Reproduction: asexual. ITS barcode: KM189553 (alternative markers: *BenA* = KM088792; *CaM* = KM089177; *RPB2* = KM089564).

***Penicillium yunnanense*** L. Cai & X.Z. Jiang, Cladistics 35: 545. 2018 [2019]. [MB818163]. — Type: HMAS 247736. Ex-type: CGMCC 3.18794 = NN051336 = CBS 144485. Infragen. class: subgen. *Aspergilloides*, sect. *Lanata-Divaricata*, ser. *Janthinella*. Reproduction: asexual. ITS barcode: KY494990 (alternative markers: *BenA* = KY495099; *CaM* = MN969340; *RPB2* = KY495048).

***Penicillium zhuangii*** L. Wang, PLoS ONE 9: e101454, 4. 2014. [MB805945]. — Type: HMAS 244922. Ex-type: CBS 137464 = NRRL 62806 = AS 3.15341. Infragen. class: subgen. *Aspergilloides*, sect. *Aspergilloides*, ser. *Hoeksiorum*. Reproduction: asexual. ITS barcode: KF769435 (alternative markers: *BenA* = KF769411; *CaM* = KF769422; *RPB2* = MN969145).

***Penicillium zonatum*** Hodges & J.J. Perry, Mycologia 65: 697. 1973. [MB319303]. — Type: BPI FSL 525. Ex-type: CBS 992.72 = DTO 096-I3 = ATCC 24353. Infragen. class: subgen. *Aspergilloides*, sect. *Lanata-Divaricata*, ser. *Dalearum*. Reproduction: homothallic. ITS barcode: GU981581 (alternative markers: *BenA* = GU981651; *CaM* = MN969315; *RPB2* = KF296461).  

***Phialomyces***

***Phialomyces arenicola*** (Chalab.) Houbraken *et al.*, this study. [MB832563]. Basionym: *Penicillium arenicola* Chalab., Not. Syst. Crypt. Inst. bot. Acad. Sci. USSR: 161-167. 1950. [MB302375]. — Type: IMI 117658. Ex-type: CBS 220.66 = ATCC 18321 = ATCC 18330 = DSM 2435 = FRR 3392 = IMI 117658 = NRRL 3392 = VKM F-1035. Reproduction: asexual. ITS barcode: GU092964 (alternative markers: *BenA* = GU092771; *CaM* = GU092801; *RPB2* = GU092935).

***Phialomyces fusiformis*** G. Delgado & Decock, Mycologia 95: 896. 2003. [MB489106]. — Type: MUCL 43747. Ex-type: MUCL 43747. Reproduction: asexual. ITS barcode: n.a. (alternative markers: *BenA* = n.a.; *CaM* = n.a.; *RPB2* = n.a.).

***Phialomyces humicoloides*** (Bills & Heredia) Houbraken *et al.*, this study. [MB832564]. Basionym: *Merimbla humicoloides* Bills & Heredia, Mycol. Res. 105: 1276. 2001. [MB474487]. — Type: BPI 748244. Ex-type: CBS 102854 = NRRL 35712. Reproduction: asexual. ITS barcode: GU092965 (alternative markers: *BenA* = GU092782; *CaM* = GU092804; *RPB2* = GU092937).

***Phialomyces macrosporus*** P.C. Misra & P.H.B. Talbot, Canad. J. Bot. 42: 1287. 1964. [MB336291]. — Type: Waite Instit. 15645. Ex-type: CBS 430.64 = ATCC 16661= IMI 110130 = MUCL 9776. Reproduction: asexual. ITS barcode: MN431404 (alternative markers: *BenA* = MN969422; *CaM* = MN969343; *RPB2* = JN121515).  

***Pseudohamigera***

***Pseudohamigera striata*** (Raper & Fennell) Houbraken *et al.*, this study. [MB832565]. Basionym: *Penicillium striatum* Raper & Fennell, Mycologia 40: 521. 1948. [MB289109]. — Type: IMI 39741. Ex-type: CBS 377.48 = ATCC 10501 = IFO 6106 = IMI 039741 = NRRL 717 = QM 1857 = VKM F-2044. Reproduction: homothallic. ITS barcode: AF454073 (alternative markers: *BenA* = GU092799; *CaM* = GU092841; *RPB2* = GU092928).  

***Pseudopenicillium***

***Pseudopenicillium cervifimosum*** Guevara-Suarez *et al.*, Fungal Syst. Evol. 5: 66. 2020. [MB822079]. — Type: CBS H-23133. Ex-type: CBS 142670 = FMR 15299. Reproduction: asexual. ITS barcode: LT899789 (alternative markers: *BenA* = LT898315; *CaM* = n.a.; *RPB2* = LT899807).

***Pseudopenicillium giganteum*** (R.Y. Roy & G.N. Singh) Guevara-Suarez *et al.*, Fungal Syst. Evol. 5: 68. 2020. [MB822077]. Basionym: *Penicillium giganteum* R.Y. Roy & G.N. Singh, Trans. Brit. Mycol. Soc. 51: 805. 1968. [MB335730]. — Type: IMI 132774. Ex-type: DTO 036-H2 = CBS 144.69 = ATCC 48996 = FRR 535 = IMI 132774 = NRRL 3553. Reproduction: asexual. ITS barcode: GU092966 (alternative markers: *BenA* = GU092779; *CaM* = GU092847; *RPB2* = GU092923).

***Pseudopenicillium megasporum*** (Orpurt & Fennell) Guevara-Suarez *et al.*, Fungal Syst. Evol. 5: 68. 2020. [MB822078]. Basionym: *Penicillium megasporum* Orpurt & Fennell, Mycologia 47: 233. 1955. [MB302408]. — Type: IMI 216904. Ex-type: CBS 256.55 = ATCC 12322 = FRR 2232 = IMI 216904 = NRRL 2232 = QM 6879 = WB 2232. Reproduction: asexual. ITS barcode: AF033494 (alternative markers: *BenA* = GU092757; *CaM* = GU092846; *RPB2* = GU092921).  

***Rasamsonia***

***Rasamsonia aegroticola*** Houbraken *et al.*, J. Clin. Microbiol. 51: 25. 2013. [MB801150]. — Type: CBS H-21031. Ex-type: DTO 137-A8 = CBS 132819 = IHEM 22641. Reproduction: asexual. ITS barcode: JX272988 (alternative markers: *BenA* = JX273020; *CaM* = JX272956; *RPB2* = MN969193).

***Rasamsonia argillacea*** (Stolk *et al.*) Houbraken & Frisvad, Antonie van Leeuwenhoek 101: 412. 2012. [MB519878]. Basionym: *Penicillium argillaceum* Stolk, H.C. Evans & T. Nilsson, Trans. Brit. Mycol. Soc. 53: 307. 1969. [MB335712]. — Type: CBS 101.69. Ex-type: DTO 097-E4 = CBS 101.69 = IBT 31199. Reproduction: asexual. ITS barcode: JF417491 (alternative markers: *BenA* = JF417456; *CaM* = JF417501; *RPB2* = JF417415).

***Rasamsonia brevistipitata*** Houbraken & Frisvad, Antonie van Leeuwenhoek 101: 413. 2012. [MB519870]. — Type: CBS H-20546. Ex-type: DTO 025-H2 = CBS 128785 = IBT 31187. Reproduction: asexual. ITS barcode: JF417488 (alternative markers: *BenA* = JF417454; *CaM* = JF417499; *RPB2* = JN406530).

***Rasamsonia byssochlamydoides*** (Stolk & Samson) Houbraken & Frisvad, Antonie van Leeuwenhoek 101: 415. 2012. [MB519877]. Basionym: *Talaromyces byssochlamydoides* Stolk & Samson, Stud. Mycol. 2: 45. 1972. [MB324415]. — Type: CBS 413.71. Ex-type: DTO 149-D6 = DTO 108-B4 = CBS 413.71 = IMI 178524 = JCM 12813 = NRRL 3658. Reproduction: homothallic. ITS barcode: JF417476 (alternative markers: *BenA* = JF417460; *CaM* = JF417512; *RPB2* = JF417437).

***Rasamsonia columbiensis*** Jurjević *et al.*, Persoonia 36: 405. 2016. [MB816869]. — Type: BPI 910043. Ex-type: CBS 141097 = CCF 5289. Reproduction: asexual. ITS barcode: LT548281 (alternative markers: *BenA* = LT548285; *CaM* = MN969326; *RPB2* = MN969195).

***Rasamsonia composticola*** Y.Y. Su & L. Cai, Mycol. Prog. 12: 217. 2013. [MB800249]. — Type: HMAS 242447. Ex-type: CGMCC 3.13669. Reproduction: homothallic. ITS barcode: JF970184 (alternative markers: *BenA* = JF970183; *CaM* = JQ729688; *RPB2* = JQ729684).

***Rasamsonia cylindrospora*** (G. Sm.) Houbraken & Frisvad, Antonie van Leeuwenhoek 101: 415. 2012. [MB519876]. Basionym: *Penicillium cylindrosporum* G. Sm., Trans. Brit. Mycol. Soc. 40: 483. 1957. [MB302392]. — Type: IMI 71623. Ex-type: DTO 138-F8 = CBS 275.58 = IBT 31202 = IMI 071623. Reproduction: asexual. ITS barcode: JF417470 (alternative markers: *BenA* = JF417448; *CaM* = JF417493; *RPB2* = JF417423).

***Rasamsonia eburnea*** (Yaguchi *et al.*) Houbraken & Frisvad, Antonie van Leeuwenhoek 101: 416. 2012. [MB519875]. Basionym: *Talaromyces eburneus* Yaguchi *et al.*, Mycoscience 35: 249. 1994. [MB362928]. — Type: PF 1151. Ex-type: DTO 105-D6 = CBS 100538 = IBT 17519. Reproduction: homothallic; maybe heterothallic (De Ravin *et al.* 2011). ITS barcode: JF417483 (alternative markers: *BenA* = JF417462; *CaM* = JF417494; *RPB2* = JN406532).

***Rasamsonia emersonii*** (Stolk) Houbraken & Frisvad, Antonie van Leeuwenhoek 101: 417. 2012. [MB519874]. Basionym: *Talaromyces emersonii* Stolk, Antonie van Leeuwenhoek 31: 262. 1965. [MB339920]. — Type: CBS 393.64. Ex-type: DTO 164-E9 = DTO 048-I1 = CBS 393.64 = ATCC 16479 = CECT 2607 = IFO 31232 = IMI 116815 = IMI 116815ii. Reproduction: homothallic. ITS barcode: JF417478 (alternative markers: *BenA* = JF417463; *CaM* = JF417510; *RPB2* = XM013471581).

***Rasamsonia frigidotolerans*** Rodr.-Andr. *et al.*, Microorganisms 2020, 8, 12: 13. 2020 [MB830608]. — Type: CBS H-23373. Ex-type: CBS 143845 = FMR 16675. Reproduction: asexual. ITS barcode: LT985886 (alternative markers: *BenA* = LT985895; *CaM* = LT985897; *RPB2* = n.a.).

***Rasamsonia piperina*** Houbraken *et al.*, J. Clin. Microbiol. 51: 26. 2013. [MB801151]. — Type: CBS H-21030. Ex-type: DTO 138-G3 = CBS 408.73 = IJFM 1326. Reproduction: asexual. ITS barcode: JX272968 (alternative markers: *BenA* = JX273000; *CaM* = JX272936; *RPB2* = MN969194).

***Rasamsonia pulvericola*** Tanney & Seifert, IMA Fungus 4: 207. 2013. [MB804677]. — Type: DAOM 242435. Ex-type: DAOM 242435. Reproduction: asexual. ITS barcode: KF242514 (alternative markers: *BenA* = KF242520; *CaM* = KF242522; *RPB2* = KF242518).  

***Sagenomella***

***Sagenomella diversispora*** (J.F.H. Beyma) W. Gams, Persoonia 10: 102. 1978. [MB323034]. Basionym: Scopulariopsis diversispora J.F.H. Beyma, Zentralbl. Bakteriol. Parasitenk., Abt. 2 96: 430. 1937. [MB263394]. — Type: van Beyma 1937, Zentralbl. Bakteriol. Parasitenk., Abt. 2 96: p. 431 Abb. 1 (– lectotype designated here, MBT392364). Ex-type: CBS 354.36 = IAM 14790 = MUCL 9029. Reproduction: asexual. ITS barcode: MN431407 (alternative markers: *BenA* = MN969427; *CaM* = MN969348; *RPB2* = MN969207).

***Sagenomella griseoviridis*** (Onions & G.L. Barron) W. Gams, Persoonia 10: 102. 1978. [MB323035]. Basionym: *Paecilomyces griseoviridis* Onions & G.L. Barron, Mycol. Pap. 107: 22. 1967. [MB335525]. — Type: CBS 426.67. Ex-type: CBS 426.67 = ATCC 18505 = IMI 113160. Reproduction: asexual. ITS barcode: MN431406 (alternative markers: *BenA* = MN969426; *CaM* = MN969347; *RPB2* = JF417438).

***Sagenomella humicola*** (Onions & G.L. Barron) W. Gams, Persoonia 10: 102. 1978. [MB323036]. Basionym: *Paecilomyces humicola* Onions & G.L. Barron, Mycol. Pap. 107: 20. 1967. [MB335526]. — Type: IMI 113166. Ex-type: CBS 427.67 = ATCC 18506 = IAM 14793 = IMI 113166. Reproduction: asexual. ITS barcode: MH859021 (alternative markers: *BenA* = MN969428; *CaM* = MN969349; *RPB2* = JF417439).

***Sagenomella ocotl*** (Bills & Heredia) Samson *et al.*, Stud. Mycol. 70: 179. 2011. [MB560681]. Basionym: *Talaromyces ocotl* Bills & Heredia, Mycologia 93: 533. 2001. [MB467796]. — Type: BPI GB6125. Ex-type: CBS 102855 = BPI GB6125. Reproduction: homothallic. ITS barcode: AF285113 (alternative markers: *BenA* = MN969431; *CaM* = MN969352; *RPB2* = n.a.).

***Sagenomella striatispora*** (Onions & G.L. Barron) W. Gams, Persoonia 10: 102. 1978. [MB323040]. Basionym: *Paecilomyces striatisporus* Onions & G.L. Barron, Mycol. Pap. 107: 19. 1967. [MB335536]. — Type: IMI 113163. Ex-type: CBS 429.67 = DTO 107-B2 = ATCC 18510 = IAM 14795 = IMI 113163. Reproduction: asexual. ITS barcode: MN431408 (alternative markers: *BenA* = MN969429; *CaM* = MN969350; *RPB2* = JF417440).

***Sagenomella verticillata*** W. Gams & B.E. Söderstr., Persoonia 10: 107. 1978. [MB323041]. — Type: CBS 414.78. Ex-type: CBS 414.78 = IAM 14697. Reproduction: asexual. ITS barcode: MN431409 (alternative markers: *BenA* = MN969430; *CaM* = MN969351; *RPB2* = MN969208).  

***Sclerocleista***

***Sclerocleista ornata*** (Raper *et al.*) Subram., Curr. Sci. 41: 757. 1972. [MB323241]. Basionym: *Aspergillus ornatus* Raper *et al.*, Mycologia 45: 678. 1953. [MB292852]. — Type: IMI 55295. Ex-type: CBS 124.53 = ATCC 16921 = IMI 055295 = LSHB BB311 = MUCL 15643 = NRRL 2256 = QM 1951 = UC 4518 = WB 2256. Reproduction: homothallic. ITS barcode: EF669704 (alternative markers: *BenA* = EF669676; *CaM* = EF669690; *RPB2* = EF669663).

***Sclerocleista thaxteri*** Subram., Curr. Sci. 41: 757. 1972. [MB323242]. — Type: On caterpillar dung, "Kittery Point", coll. R. Thaxter ex Farlow Herbarium. Harvard University. Ex-type: CBS 105.25 = IFO 4042 = IFO 8130 = IMI 055296 = NRRL 2292 = WB 2292. Reproduction: homothallic. ITS barcode: EU021599 (alternative markers: *BenA* = EU021668; *CaM* = EU021689; *RPB2* = EU021630).  

***Talaromyces***

***Talaromyces acaricola*** Visagie *et al.*, Persoonia 36: 49. 2016. [MB810899]. — Type: CBS H-21632. Ex-type: CBS 137386 = DTO 183-B3 = DAOM 241025 = IBT 32387. Infragen. class: sect. *Islandici*. Reproduction: asexual. ITS barcode: JX091476 (alternative markers: *BenA* = JX091610; *CaM* = JX140729; *RPB2* = KF984956).

***Talaromyces aculeatus*** (Raper & Fennell) Samson *et al.*, Stud. Mycol. 71: 174. 2011. [MB560639]. Basionym: *Penicillium aculeatum* Raper & Fennell, Mycologia 40: 535. 1948. [MB289073]. — Type: IMI 040588. Ex-type: CBS 289.48 = ATCC 10409 = IMI 040588 = NRRL 2129 = NRRL A-1474. Infragen. class: sect. *Talaromyces*. Reproduction: asexual. ITS barcode: KF741995 (alternative markers: *BenA* = KF741929; *CaM* = KF741975; *RPB2* = MH793099).

***Talaromyces adpressus*** A.J. Chen *et al.*, Stud. Mycol. 84:124. 2016 [MB817397]. — Type: CBS H-22507. Ex-type: CBS 140620 = CGMCC3.18211 = DTO 317-G4. Infragen. class: sect. *Talaromyces*. Reproduction: asexual. ITS barcode: KU866657 (alternative markers: *BenA* = KU866844; *CaM* = KU866741; *RPB2* = KU867001).

***Talaromyces aerius*** A.J. Chen *et al.*, Stud. Mycol. 84:124. 2016 [MB817398]. — Type: CBS H-22506. Ex-type: CBS 140611 = CGMCC3.18197 = DTO 317-C7. Infragen. class: sect. *Trachyspermi*. Reproduction: asexual. ITS barcode: KU866647 (alternative markers: *BenA* = KU866835; *CaM* = KU866731; *RPB2* = KU866991).

***Talaromyces aerugineus*** (Samson) N. Yilmaz *et al.*, Stud. Mycol. 78: 210. 2014. [MB809553]. Basionym: *Paecilomyces aerugineus* Samson, Stud. Mycol. 6: 20. 1974. [MB319096]. — Type: CBS H-7448. Ex-type: CBS 350.66 = BDUN 276 = IMI 105412. Infragen. class: sect. *Helici*. Reproduction: asexual. ITS barcode: AY753346 (alternative markers: *BenA* = KJ865736; *CaM* = KJ885285; *RPB2* = JN121502).

***Talaromyces affinitatimellis*** Rodr.-Andr. *et al.*, IMA Fungus 10: 20. 2019. [MB823591]. — Type: CBS H-23370. Ex-type: FMR 15690 = CBS 143840. Infragen. class: sect. *Trachyspermi*. Reproduction: asexual. ITS barcode: LT906543 (alternative markers: *BenA* = LT906552; *CaM* = LT906549; *RPB2* = LT906546).

***Talaromyces albobiverticillius*** (H.-M. Hsieh *et al.*) Samson *et al.*, Stud. Mycol. 71: 174. 2011. [MB560683]. Basionym: *Penicillium albobiverticillium* H.M. Hsieh, Y.M. Ju & S.Y. Hsieh, Fungal Science 25: 26. 2010. [MB519193]. — Type: BCRC 34774. Ex-type: CBS 133440 = DTO 166-E5 = YMJ 1292. Infragen. class: sect. *Trachyspermi*. Reproduction: asexual. ITS barcode: HQ605705 (alternative markers: *BenA* = KF114778; *CaM* = KJ885258; *RPB2* = KM023310).

***Talaromyces allahabadensis*** (B.S. Mehrotra & D. Kumar) Samson *et al.*, Stud. Mycol. 71: 174. 2011. [MB560640]. Basionym: *Penicillium allahabadense* B.S. Mehrotra & D. Kumar, Canad. J. Bot. 40: 1399. 1962. [MB335707]. — Type: University of Allahabad P-26. Ex-type: CBS 453.93 = ATCC 15067 = CBS 304.63. Infragen. class: sect. *Islandici*. Reproduction: asexual. ITS barcode: KF984873 (alternative markers: *BenA* = KF984614; *CaM* = KF984768; *RPB2* = KF985006).

***Talaromyces alveolaris*** Guevara-Suarez *et al.*, Mycoses 60: 656. 2017. [MB820459]. — Type: CBS H-22999. Ex-type: CBS 142379 = UTHSC DI16-147 = FMR 13963. Infragen. class: sect. *Talaromyces*. Reproduction: asexual. ITS barcode: LT558969 (alternative markers: *BenA* = LT559086; *CaM* = LT795596; *RPB2* = LT795597).

***Talaromyces amazonensis*** N. Yilmaz *et al.*, Mycol. Prog. 15: 1052. 2016. [MB816230]. — Type: HUA 197223. Ex-type: CBS 140373 = IBT 23215 = DTO 093-F9. Infragen. class: sect. *Talaromyces*. Reproduction: homothallic. ITS barcode: KX011509 (alternative markers: *BenA* = KX011490; *CaM* = KX011502; *RPB2* = MN969186).

***Talaromyces amestolkiae*** N. Yilmaz *et al.*, Persoonia 29: 48. 2012. [MB801358]. — Type: CBS H-21050. Ex-type: CBS 132696 = DTO 179-F5. Infragen. class: sect. *Talaromyces*. Reproduction: Heterothallic (Yilmaz *et al.* 2016). ITS barcode: JX315660 (alternative markers: *BenA* = JX315623; *CaM* = KF741937; *RPB2* = JX315698).

***Talaromyces amyrossmaniae*** Rajeshkumar *et al.*, MycoKeys 45: 47. 2019. [MB518601]. — Type: AMH 9330. Ex-type: NFCCI 1919 = KAS 3038. Infragen. class: sect. *Trachyspermi*. Reproduction: asexual. ITS barcode: MH909062 (alternative markers: *BenA* = MH909064; *CaM* = MH909068; *RPB2* = MH909066).

***Talaromyces angelicae*** S.H. Yu *et al.*, J. Microbiol. 51: 707. 2013. [MB818651]. — Type: KACC 46611. Ex-type: KACC 46611. Infragen. class: sect. *Talaromyces*. Reproduction: asexual. ITS barcode: KF183638 (alternative markers: *BenA* = KF183640; *CaM* = KJ885259; *RPB2* = KX961275).

***Talaromyces annesophieae*** Houbraken, Persoonia 39: 461. 2017. [MB823027]. — Type: CBS H-23216. Ex-type: CBS 142939 = DTO 377-F3 = JW9011. Infragen. class: sect. *Talaromyces*. Reproduction: asexual. ITS barcode: MF574592 (alternative markers: *BenA* = MF590098; *CaM* = MF590104; *RPB2* = MN969199).

***Talaromyces apiculatus*** Samson *et al.*, Stud. Mycol. 71: 174. 2011. [MB560641]. — Type: CBS H-20755. Ex-type: CBS 312.59 = ATCC 18315 = FRR 635 = IMI 068239. Infragen. class: sect. *Talaromyces*. Reproduction: asexual. ITS barcode: JN899375 (alternative markers: *BenA* = KF741916; *CaM* = KF741950; *RPB2* = KM023287).

***Talaromyces argentinensis*** Jurjević & S.W. Peterson, Fungal Biol. 123: 751. 2019. [MB827826]. — Type: BPI 910716. Ex-type: NRRL 28750. Infragen. class: sect. *Talaromyces*. Reproduction: homothallic. ITS barcode: MH793045 (alternative markers: *BenA* = MH792917; *CaM* = MH792981; *RPB2* = MH793108).

***Talaromyces assiutensis*** Samson & Abdel-Fattah, Persoonia 9: 501. 1978. [MB324414]. — Type: CBS 147.78. Ex-type: CBS 147.78. Infragen. class: sect. *Trachyspermi*. Reproduction: homothallic. ITS barcode: JN899323 (alternative markers: *BenA* = KJ865720; *CaM* = KJ885260; *RPB2* = KM023305).

***Talaromyces atricola*** S.W. Peterson & Jurjević, PLoS ONE 8: e78084, 8. 2013. [MB804733]. — Type: unknown. Ex-type: CBS 255.31 = NRRL 1052 = FRR 1052 = Thom 4640.439 = ATCC 52257. Infragen. class: sect. *Islandici*. Reproduction: asexual. ITS barcode: KF984859 (alternative markers: *BenA* = KF984566; *CaM* = KF984719; *RPB2* = KF984948).

***Talaromyces atroroseus*** N. Yilmaz *et al.*, PLoS ONE 8: e84102, 8. 2013. [MB804901]. — Type: CBS H-21790. Ex-type: CBS 133442 = IBT 32470 = DTO 178-A4. Infragen. class: sect. *Trachyspermi*. Reproduction: asexual. ITS barcode: KF114747 (alternative markers: *BenA* = KF114789; *CaM* = KJ775418; *RPB2* = KM023288).

***Talaromyces aurantiacus*** (J.H. Mill. *et al.*) Samson *et al.*, Stud. Mycol. 71: 175. 2011. [MB560642]. Basionym: *Penicillium aurantiacum* J.H. Mill. *et al.*, Mycologia 49: 797. 1957. [MB302378]. — Type: No. 1736 (A.A. Foster). Ex-type: CBS 314.59 = ATCC 13216 = IMI 099722 = NRRL 3398. Infragen. class: sect. *Talaromyces*. Reproduction: asexual. ITS barcode: JN899380 (alternative markers: *BenA* = KF741917; *CaM* = KF741951; *RPB2* = KX961285).

***Talaromyces australis*** Visagie *et al.*, Mycoscience 56: 492 2015. [MB808236]. — Type: CBS H-21598. Ex-type: CBS 137102 = DTO 273-F5 = IBT 14256 = FRR 2005. Infragen. class: sect. *Talaromyces*. Reproduction: asexual. ITS barcode: KF741991 (alternative markers: *BenA* = KF741922; *CaM* = KF741971; *RPB2* = KX961284).

***Talaromyces austrocalifornicus*** Yaguchi & Udagawa, Trans. Mycol. Soc. Japan 34: 245. 1993. [MB361182]. — Type: CBM-PF 1117. Ex-type: CBS 644.95 = IBT 17522. Infragen. class: sect. *Trachyspermi*. Reproduction: homothallic. ITS barcode: JN899357 (alternative markers: *BenA* = KJ865732; *CaM* = KJ885261; *RPB2* = MN969147).

***Talaromyces bacillisporus*** [as “*bacillosporus*”] (Swift) C.R. Benj., Mycologia 47: 682. 1955. [MB118745]. Basionym: *Penicillium bacillisporum* Swift, Bull. Torrey Bot. Club: 221. 1932. [MB119961]. — Type: CBS H-7813 (Isotype). Ex-type: CBS 296.48 = ATCC 10126 = IMI 040045 = NRRL 1025. Infragen. class: sect. *Bacillispori*. Reproduction: homothallic. ITS barcode: KM066182 (alternative markers: *BenA* = AY753368; *CaM* = KJ885262; *RPB2* = JF417425).

***Talaromyces basipetosporus*** Stchigel *et al.*, IMA Fungus 10: 20, 17. 2019. [MB823589]. — Type: CBS H-23365. Ex-type: FMR 9720 = CBS 143836. Infragen. class: sect. *Trachyspermi*. Reproduction: asexual. ITS barcode: LT906542 (alternative markers: *BenA* = LT906563; *CaM* = n.a.; *RPB2* = LT906545).

***Talaromyces beijingensis*** A.J. Chen *et al.*, Stud. Mycol. 84:125. 2016 [MB817395]. — Type: CBS H-22508. Ex-type: CBS 140617 = CGMCC3.18200 = DTO 317-D8. Infragen. class: sect. *Talaromyces*. Reproduction: asexual. ITS barcode: KU866649 (alternative markers: *BenA* = KU866837; *CaM* = KU866733; *RPB2* = KU866993).

***Talaromyces bohemicus*** (Fassat. & Pěčková) N. Yilmaz *et al.*, Stud. Mycol. 78: 227. 2014 [MB809554]. Basionym: *Sagenomella bohemica* Fassat. & Pecková, Ceská Mykologie 44: 240. 1990. [MB361859]. — Type: unknown. Ex-type: CBS 545.86 = CCF 2330 = IAM 14789. Infragen. class: sect. *Helici*. Reproduction: asexual. ITS barcode: JN899400 (alternative markers: *BenA* = KJ865719; *CaM* = KJ885286; *RPB2* = JN121532).

***Talaromyces boninensis*** (Yaguchi & Udagawa) Samson *et al.*, Stud. Mycol. 71: 175. 2011. [MB560643]. Basionym: *Talaromyces helicus* var. *boninensis* Yaguchi & Udagawa, Trans. Mycol. Soc. Japan 33: 511. 1992. [MB359708]. — Type: CBM PF-1103. Ex-type: CBS 650.95 = IBT 17516 . Infragen. class: sect. *Helici*. Reproduction: homothallic. ITS barcode: JN899356 (alternative markers: *BenA* = KJ865721; *CaM* = KJ885263; *RPB2* = KM023276).

***Talaromyces borbonicus*** Houbraken, Mycologia 110: 318. 2018. [MB821643]. — Type: CBS H-22672. Ex-type: CBS 141340 = DTO 351-D3. Infragen. class: sect. *Helici*. Reproduction: asexual. ITS barcode: MG827091 (alternative markers: *BenA* = MG855687; *CaM* = MG855688; *RPB2* = MG855689).

***Talaromyces brasiliensis*** R.N. Barbosa *et al.*, Antonie van Leeuwenhoek 111: 1902. 2018 [MB822214]. — Type: URM 90494. Ex-type: CBS 142493 = URM 7618. Infragen. class: sect. *Trachyspermi*. Reproduction: asexual. ITS barcode: MF278323 (alternative markers: *BenA* = LT855560; *CaM* = LT855563; *RPB2* = MN969198).

***Talaromyces brunneosporus*** Rodr.-Andr. *et al.*, IMA Fungus 10: 20, 19. 2019. [MB823590]. — Type: CBS H-23375. Ex-type: FMR 16566 = CBS 144320. Infragen. class: sect. *Purpurei* (tentative, see Supplementary Fig. S3). Reproduction: asexual. ITS barcode: LT962487 (alternative markers: *BenA* = LT962483; *CaM* = LT962488; *RPB2* = LT962485).

***Talaromyces brunneus*** (Udagawa) Samson *et al.*, Stud. Mycol. 71: 175. 2011. [MB560644]. Basionym: *Penicillium brunneum* Udagawa, J. Agric. Sci. Tokyo Nogyo Daig. 5: 16. 1959. [MB302383]. — Type: NHL 6054. Ex-type: CBS 227.60 = ATCC 18229 = FRR 646 = IFO 6438 = IHEM 3907 = IMI 078259 = MUCL 31318. Infragen. class: sect. *Islandici*. Reproduction: asexual. ITS barcode: JN899365 (alternative markers: *BenA* = KJ865722; *CaM* = KJ885264; *RPB2* = KM023272).

***Talaromyces calidicanius*** (J.L. Chen) Samson *et al.*, Stud. Mycol. 71: 175. 2011. [MB560645]. Basionym: *Penicillium calidicanium* J.L. Chen, Mycologia 94: 870. 2002. [MB483963]. — Type: CFC-7 (isotype TNM F12246). Ex-type: CBS 112002. Infragen. class: sect. *Talaromyces*. Reproduction: asexual. ITS barcode: JN899319 (alternative markers: *BenA* = HQ156944; *CaM* = KF741934; *RPB2* = KM023311).

***Talaromyces californicus*** Jurjević & S.W. Peterson, Fungal Biol. 123: 752. 2019. [MB827827]. — Type: BPI 910714. Ex-type: NRRL 58168. Infragen. class: sect. *Talaromyces*. Reproduction: asexual. ITS barcode: MH793056 (alternative markers: *BenA* = MH792928; *CaM* = MH792992; *RPB2* = MH793119).

***Talaromyces catalonicus*** Guevara-Suarez *et al.*, Fungal Syst. Evol. 5: 68. 2020. [MB822080]. — Type: CBS H-23212. Ex-type: CBS 143039 = FMR 16441. Infragen. class: sect. *Trachyspermi*. Reproduction: asexual. ITS barcode: LT899793 (alternative markers: *BenA* = LT898318; *CaM* = LT899775; *RPB2* = LT899811).

***Talaromyces cecidicola*** (Seifert *et al.*) Samson *et al.*, Stud. Mycol. 71: 175. 2011. [MB560646]. Basionym: *Penicillium cecidicola* Seifert, Hoekstra & Frisvad, Stud. Mycol. 50: 520. 2004. [MB500150]. — Type: DAOM 233329. Ex-type: CBS 101419 = DAOM233329. Infragen. class: sect. *Purpurei*. Reproduction: asexual. ITS barcode: AY787844 (alternative markers: *BenA* = FJ753295; *CaM* = KJ885287; *RPB2* = KM023309).

***Talaromyces cerinus*** A.J. Chen *et al.*, Stud. Mycol. 84:125. 2016 [MB817393]. — Type: CBS H-22513. Ex-type: CBS 140622 = CGMCC3.18212 = DTO 318-A2. Infragen. class: sect. *Islandici*. Reproduction: asexual. ITS barcode: KU866658 (alternative markers: *BenA* = KU866845; *CaM* = KU866742; *RPB2* = KU867002).

***Talaromyces chlamydosporus*** A.J. Chen *et al.*, Stud. Mycol. 84:136. 2016 [MB817392]. — Type: CBS H-22509. Ex-type: CBS 140635 = CGMCC 3.18199 = DTO 317-D5. Infragen. class: sect. *Islandici*. Reproduction: asexual. ITS barcode: KU866648 (alternative markers: *BenA* = KU866836; *CaM* = KU866732; *RPB2* = KU866992).

***Talaromyces chlorolomus*** [as “*chloroloma*”] Visagie & K. Jacobs, Persoonia 28: 18. 2012. [MB585178]. — Type: PREM 60033. Ex-type: DAOM 241016 = CV 2802. Infragen. class: sect. *Purpurei*. Reproduction: asexual. ITS barcode: FJ160273 (alternative markers: *BenA* = GU385736; *CaM* = KJ885265; *RPB2* = KM023304).

***Talaromyces cinnabarinus*** (S.C. Jong & E.E. Davis) N. Yilmaz *et al.*, Stud. Mycol. 78: 234. 2014. [MB809557]. Basionym: *Paecilomyces cinnabarinus* S.C. Jong & E.E. Davis, Mycologia 67: 1144. 1976. [MB319103]. — Type: CBS H-6686. Ex-type: CBS 267.72 = ATCC 26215 = NHL 2673. Infragen. class: sect. *Helici*. Reproduction: homothallic. ITS barcode: JN899376 (alternative markers: *BenA* = AY753377; *CaM* = KJ885256; *RPB2* = JN121477).

***Talaromyces clemensii*** Viisagie & N. Yilmaz, Persoonia 43: 407. 2019. [MB832488]. — Type: PREM 62301. Ex-type: PPRI 26753 = CMV016A4. Infragen. class: sect. *Trachyspermi*. Reproduction: asexual. ITS barcode: MK951940 (alternative markers: *BenA* = MK951833; *CaM* = MK951906; *RPB2* = MN418451).

***Talaromyces cnidii*** S.H. Yu *et al.*, J. Microbiol. 51: 707. 2013. [MB804809]. — Type: KACC 46617. Ex-type: KACC 46617. Infragen. class: sect. *Talaromyces*. Reproduction: asexual. ITS barcode: KF183639 (alternative markers: *BenA* = KF183641; *CaM* = KJ885266; *RPB2* = KM023299).

***Talaromyces coalescens*** (Quintan.) Samson *et al.*, Stud. Mycol. 71: 175. 2011. [MB560647]. Basionym: *Penicillium coalescens* Quintan., Mycopathologia 84 (2–3): 115. 1984. [MB107101]. — Type: CBS 103.83. Ex-type: CBS 103.83. Infragen. class: sect. *Purpurei*. Reproduction: asexual. ITS barcode: JN899366 (alternative markers: *BenA* = JX091390; *CaM* = KJ885267; *RPB2* = KM023277).

***Talaromyces columbiensis*** N. Yilmaz *et al.*, Mycol. Prog. 15: 1053. 2016. [MB816231]. — Type: HUA 197225. Ex-type: CBS 113151 = IBT 23206 = DTO 058-F3. Infragen. class: sect. *Bacillispori*. Reproduction: homothallic. ITS barcode: KX011503 (alternative markers: *BenA* = KX011488; *CaM* = KX011499; *RPB2* = MN969187).

***Talaromyces columbinus*** S.W. Peterson & Jurjević, PLoS ONE 8: e78084, 6. 2013. [MB804732]. — Type: BPI 892668. Ex-type: NRRL 58811. Infragen. class: sect. *Islandici*. Reproduction: asexual. ITS barcode: KJ865739 (alternative markers: *BenA* = KF196843; *CaM* = KJ885288; *RPB2* = KM023270).

***Talaromyces convolutus*** Udawaga, Mycotaxon 48: 141. 1993. [MB360474]. — Type: CBM SUM-3018. Ex-type: CBS 100537 = IBT 14989. Infragen. class: sect. *Trachyspermi*. Reproduction: homothallic. ITS barcode: JN899330 (alternative markers: *BenA* = KF114773; *CaM* = MN969316; *RPB2* = JN121414).

***Talaromyces coprophilus*** Guevara-Suarez *et al.*, Fungal Syst. Evol. 5: 68. 2020. [MB822088]. — Type: CBS H-23144. Ex-type: CBS 142756 = FMR 15199. Infragen. class: sect. *Talaromyces*. Reproduction: homothallic. ITS barcode: LT899794 (alternative markers: *BenA* = LT898319; *CaM* = LT899776; *RPB2* = LT899812).

***Talaromyces crassus*** Visagie *et al.*, Persoonia 36: 49. 2016. [MB810900]. — Type: CBS H-21631. Ex-type: CBS 137381 = DTO 181-C5 = DAOM 241027 = IBT 32814. Infragen. class: sect. *Islandici*. Reproduction: asexual. ITS barcode: JX091472 (alternative markers: *BenA* = JX091608; *CaM* = JX140727; *RPB2* = KF984914).

***Talaromyces cucurbitiradicus*** L. Su & Y.C. Niu, Mycologia 110: 380. 2018. [MB820559]. — Type: HMAS 247175. Ex-type: ACCC 39155. Infragen. class: sect. *Talaromyces*. Reproduction: asexual. ITS barcode: KY053254 (alternative markers: *BenA* = KY053228; *CaM* = KY053246; *RPB2* = n.a.).

***Talaromyces delawarensis*** Jurjević & S.W. Peterson, Mycologia 109: 541. 2017. [MB817998]. — Type: BPI-910148. Ex-type: NRRL 58874. Infragen. class: sect. *Islandici*. Reproduction: asexual. ITS barcode: KX657324 (alternative markers: *BenA* = KX657055; *CaM* = KX657158; *RPB2* = KX657490).

***Talaromyces dendriticus*** (Pitt) Samson *et al.*, Stud. Mycol. 71: 175. 2011. [MB560648]. Basionym: *Penicillium dendriticum* Pitt, Gen. Penicillium: 413. 1980. [MB116318]. — Type: IMI 216897. Ex-type: CBS 660.80 = IMI 216897. Infragen. class: sect. *Purpurei*. Reproduction: asexual. ITS barcode: JN899339 (alternative markers: *BenA* = JX091391; *CaM* = KF741965; *RPB2* = KM023286).

***Talaromyces derxii*** Takada & Udagawa, Mycotaxon 31: 418. 1988. [MB133755]. — Type: NHL 2980. Ex-type: CBS 412.89 = NHL 2981. Infragen. class: sect. *Talaromyces*. Reproduction: Heterothallic. ITS barcode: JN899327 (alternative markers: *BenA* = JX494306; *CaM* = KF741959; *RPB2* = KM023282).

***Talaromyces dimorphus*** X.Z. Jiang & L. Wang, Sci. Rep. 8: 4932, 2. 2018. [MB570521]. — Type: HMAS 247023. Ex-type: AS3.15692 = NN072337. Infragen. class: sect. *Talaromyces*. Reproduction: asexual. ITS barcode: KY007095 (alternative markers: *BenA* = KY007111; *CaM* = KY007103; *RPB2* = KY112593).

***Talaromyces diversiformis*** A.J. Chen *et al.*, Stud. Mycol. 84:136. 2016 [MB818696]. — Type: CBS H-22841. Ex-type: CBS 141931 = CGMCC 3.18204 = DTO 317-E3. Infragen. class: sect. *Helici*. Reproduction: asexual. ITS barcode: KX961215 (alternative markers: *BenA* = KX961216; *CaM* = KX961259; *RPB2* = KX961274).

***Talaromyces diversus*** (Raper & Fennell) Samson *et al.*, Stud. Mycol. 71: 175. 2011. [MB560649]. Basionym: *Penicillium diversum* Raper & Fennell, Mycologia 40: 539. 1948. [MB289080]. — Type: IMI 040579. Ex-type: CBS 320.48 = ATCC 10437 = DSM 2212 = IMI 040579 = IMI 040579ii = NRRL 2121. Infragen. class: sect. *Trachyspermi*. Reproduction: asexual. ITS barcode: KJ865740 (alternative markers: *BenA* = KJ865723; *CaM* = KJ885268; *RPB2* = KM023285).

***Talaromyces domesticus*** Jurjević & S.W. Peterson, Fungal Biol. 123: 754. 2019. [MB827828]. — Type: BPI 910711. Ex-type: NRRL 58121. Infragen. class: sect. *Talaromyces*. Reproduction: asexual. ITS barcode: MH793055 (alternative markers: *BenA* = MH792927; *CaM* = MH792991; *RPB2* = MH793118).

***Talaromyces duclauxii*** (Delacr.) Samson *et al.*, Stud. Mycol. 71: 175. 2011. [MB560650]. Basionym: *Penicillium duclauxii* Delacr., Bull. Soc. Mycol. France 7: 107. 1891. [MB169645]. — Type: IMI 24312. Ex-type: CBS 322.48 = ATCC 10439 = IMI 040044 = MUCL 28672 = MUCL 29094 = MUCL 29212 = NRRL 1030. Infragen. class: sect. *Talaromyces*. Reproduction: asexual. ITS barcode: JN899342 (alternative markers: *BenA* = JX091384; *CaM* = KF741955; *RPB2* = JN121491).

***Talaromyces emodensis*** Udagawa, Mycotaxon 48: 146. 1993. [MB360476]. — Type: CBM SUM-3025. Ex-type: CBS 100536 = IBT 14990. Infragen. class: sect. *Bacillispori*. Reproduction: homothallic. ITS barcode: JN899337 (alternative markers: *BenA* = KJ865724; *CaM* = KJ885269; *RPB2* = JF417445).

***Talaromyces endophyticus*** L. Su & Y.C. Niu, Mycologia 110: 380. 2018. [MB817808]. — Type: HMAS 254730. Ex-type: ACCC 39141. Infragen. class: sect. *Islandici*. Reproduction: asexual. ITS barcode: KX639168 (alternative markers: *BenA* = KX639174; *CaM* = KX639165; *RPB2* = n.a.).

***Talaromyces erythromellis*** (A.D. Hocking) Samson *et al.*, Stud. Mycol. 71: 175. 2011. [MB560652]. Basionym: *Penicillium erythromellis* A.D. Hocking, The Genus Penicillium: 459. 1980 [1979]. [MB116314]. — Type: IMI 216899. Ex-type: CBS 644.80 = FRR 1868 = IMI 216899. Infragen. class: sect. *Trachyspermi*. Reproduction: protoheterothallic, MAT1-1-1 detected (López-Villavicencio *et al.* 2010). ITS barcode: JN899383 (alternative markers: *BenA* = HQ156945; *CaM* = KJ885270; *RPB2* = KM023290).

***Talaromyces euchlorocarpius*** Yaguchi *et al.*, Mycoscience 40: 133. 1999. [MB460481]. — Type: CBM-PF 1203. Ex-type: PF 1203 = DTO 176-I3 = DTO 176-I4. Infragen. class: sect. *Talaromyces*. Reproduction: homothallic. ITS barcode: AB176617 (alternative markers: *BenA* = KJ865733; *CaM* = KJ885271; *RPB2* = KM023303).

***Talaromyces flavovirens*** (Durieu & Mont.) Visagie *et al.*, Mycotaxon 122: 404. 2012. [MB800438]. Basionym: *Lasioderma flavovirens* Durieu & Mont., Ann. Sci. Nat. Bot. 4: 364. 1845. [MB120473]. — Type: PC 0088796 (lectotype); BCC 473 (epitype) = BCN 473. Ex-type: CBS 102801 = IBT 27044. Infragen. class: sect. *Talaromyces*. Reproduction: Heterothallic. ITS barcode: JN899392 (alternative markers: *BenA* = JX091376; *CaM* = KF741933; *RPB2* = KX961283).

***Talaromyces flavus*** (Klöcker) Stolk & Samson, Stud. Mycol. 2: 10. 1972. [MB324416]. Basionym: *Gymnoascus flavus* Klöcker, Hedwigia 41: 80. 1902. [MB225997]. — Type: CBS H-7820. Ex-type: CBS 310.38 = IMI 197477 = NRRL 2098. Infragen. class: sect. *Talaromyces*. Reproduction: homothallic. ITS barcode: JN899360 (alternative markers: *BenA* = JX494302; *CaM* = KF741949; *RPB2* = JF417426).

***Talaromyces francoae*** N. Yilmaz *et al.*, Mycol. Prog. 15: 1054. 2016. [MB816232]. — Type: HUA 197224. Ex-type: CBS 113134 = IBT 23221 = DTO 056-D9. Infragen. class: sect. *Talaromyces*. Reproduction: asexual. ITS barcode: KX011510 (alternative markers: *BenA* = KX011489; *CaM* = KX011501; *RPB2* = MN969188).

***Talaromyces funiculosus*** (Thom) Samson *et al.*, Stud. Mycol. 71: 176. 2011. [MB560653]. Basionym: *Penicillium funiculosum* Thom, U.S.D.A. Bur. Ani. Ind. Bull. 118: 69. 1910. [MB152047]. — Type: IMI 193019. Ex-type: CBS 272.86 = IMI 193019. Infragen. class: sect. *Talaromyces*. Reproduction: protoheterothallic, both mating types detected (López-Villavicencio *et al.* 2010). ITS barcode: JN899377 (alternative markers: *BenA* = MN969408; *CaM* = KF741945; *RPB2* = KM023293).

***Talaromyces fuscoviridis*** Visagie *et al.*, Mycoscience 56: 492. 2015. [MB808234]. — Type: CBS H-21603. Ex-type: CBS 193.69 = DTO 258-I7 = IBT 14846 = IBT 32646. Infragen. class: sect. *Talaromyces*. Reproduction: asexual. ITS barcode: KF741979 (alternative markers: *BenA* = KF741912; *CaM* = KF741942; *RPB2* = MN969156).

***Talaromyces fusiformis*** A.J. Chen *et al.*, Stud. Mycol. 84:139. 2016 [MB817396]. — Type: CBS H-22510. Ex-type: CBS 140637 = CGMCC3.18210 = DTO 317-F4. Infragen. class: sect. *Talaromyces*. Reproduction: asexual. ITS barcode: KU866656 (alternative markers: *BenA* = KU866843; *CaM* = KU866740; *RPB2* = KU867000).

***Talaromyces galapagensis*** Samson & Mahoney, Trans. Brit. Mycol. Soc. 69: 158. 1977. [MB324417]. — Type: CBS H-7489. Ex-type: CBS 751.74 = IFO 31796. Infragen. class: sect. *Talaromyces*. Reproduction: homothallic. ITS barcode: JN899358 (alternative markers: *BenA* = JX091388; *CaM* = KF741966; *RPB2* = KX961280).

***Talaromyces georgiensis*** M. Guevara-Suarez *et al.*, Mycoses 60: 656. 2017. [MB820460]. — Type: CBS H-23000. Ex-type: UTHSC DI16-145 = CBS 142380 = DTO 410-E8 = FMR 14270. Infragen. class: sect. *Helici*. Reproduction: asexual. ITS barcode: LT558967 (alternative markers: *BenA* = LT559084; *CaM* = n.a.; *RPB2* = LT795606).

***Talaromyces guatemalensis*** A. Nováková *et al.*, Persoonia 43: 409. 2019. [MB832313]. — Type: PRM 952195. Ex-type: CCF 6215 = GUA2˗1. Infragen. class: sect. *Trachyspermi*. Reproduction: asexual. ITS barcode: MN322789 (alternative markers: *BenA* = MN329687; *CaM* = MN329688; *RPB2* = MN329689).

***Talaromyces hachijoensis*** Yaguchi *et al.*, Mycoscience 37: 157. 1996. [MB416016]. — Type: PF 1174. Ex-type: CBM-PF 1174 = IFM 53624. Infragen. class: sect. *Bacillispori*. Reproduction: homothallic. ITS barcode: AB176620 (alternative markers: *BenA* = n.a.; *CaM* = n.a.; *RPB2* = n.a.).

***Talaromyces halophytorum*** Y.H. You & S.B. Hong, Mycobiology, accepted. 2020. [MB830295]. — Type: KACC 48127. Ex-type: KACC 48127 = NIBRFGC 000501933. Infragen. class: sect. *Trachyspermi*. Reproduction: asexual. ITS barcode: MH725786 (alternative markers: *BenA* = MH729367; *CaM* = MK111426; *RPB2* = MK111427).

***Talaromyces heiheensis*** X.C. Wang & W.Y. Zhuang, Mycol. Prog. 16: 75. 2016. [MB570286]. — Type: HMAS 248789. Ex-type: CGMCC 3.18012. Infragen. class: sect. *Trachyspermi*. Reproduction: asexual. ITS barcode: KX447526 (alternative markers: *BenA* = KX447525; *CaM* = KX447532; *RPB2* = KX447529).

***Talaromyces helicus*** (Raper & Fennel) C.R. Benj., Mycologia 47: 684. 1955. [MB306715]. Basionym: *Penicillium helicum* Raper & Fennell, Mycologia 40: 515. 1948. [MB289086]. — Type: IMI 040593. Ex-type: CBS 335.48 = ATCC 10451 = DSM 3705 = IMI 040593 = NRRL 2106. Infragen. class: sect. *Helici*. Reproduction: homothallic. ITS barcode: JN899359 (alternative markers: *BenA* = KJ865725; *CaM* = KJ885289; *RPB2* = KM023273).

***Talaromyces herodensis*** Jurjević & S.W. Peterson, Mycologia 109: 546. 2017. [MB818001]. — Type: BPI-910152. Ex-type: NRRL 62467. Infragen. class: sect. *Islandici*. Reproduction: asexual. ITS barcode: KX657338 (alternative markers: *BenA* = KX657061; *CaM* = KX657182; *RPB2* = KX657524).

***Talaromyces indigoticus*** Takada & Udagawa, Mycotaxon 46: 129. 1993. [MB359290]. — Type: CBM SUM-3010. Ex-type: CBS 100534 = IBT 17590. Infragen. class: sect. *Talaromyces*. Reproduction: homothallic. ITS barcode: JN899331 (alternative markers: *BenA* = JX494308; *CaM* = KF741931; *RPB2* = KX961278).

***Talaromyces infraolivaceus*** Visagie *et al.*, Persoonia 36: 52. 2016. [MB810901]. — Type: CBS H-21633. Ex-type: CBS 137385 = DTO 182-I2 = DAOM 241024 = IBT 32487. Infragen. class: sect. *Islandici*. Reproduction: asexual. ITS barcode: JX091481 (alternative markers: *BenA* = JX091615; *CaM* = JX140734; *RPB2* = KF984949).

***Talaromyces intermedius*** (Apinis) Stolk & Samson, Stud. Mycol. 2: 21. 1972. [MB324418]. Basionym: *Arachniotus intermedius* Apinis, Mycol. Pap. 96: 45. 1964. [MB326426]. — Type: CBS H-7828. Ex-type: CBS 152.65 = BDUN 267 = IFO 31752 = IMI 100874. Infragen. class: sect. *Talaromyces*. Reproduction: homothallic. ITS barcode: JN899332 (alternative markers: *BenA* = JX091387; *CaM* = KJ885290; *RPB2* = KX961282).

***Talaromyces iowaense*** Jurjević *et al.*, Persoonia 41: 407. 2018. [MB828092]. — Type: BPI 910643. Ex-type: NRRL 66822 = ITEM 17527 = EMSL 2233. Infragen. class: sect. *Purpurei*. Reproduction: asexual. ITS barcode: MH281565 (alternative markers: *BenA* = MH282578; *CaM* = MH282579; *RPB2* = MH282577).

***Talaromyces islandicus*** (Sopp) Samson *et al.*, Stud. Mycol. 71: 176. 2011. [MB560654]. Basionym: *Penicillium islandicum* Sopp, Skr. VidenskSelsk. Christiania, Kl. I, Math.-Natur. (no. 11): 161. 1912. [MB162841]. — Type: IMI 040042. Ex-type: CBS 338.48 = ATCC 10127 = IMI 040042 = MUCL 31324 = NRRL 1036. Infragen. class: sect. *Islandici*. Reproduction: asexual. ITS barcode: KF984885 (alternative markers: *BenA* = KF984655; *CaM* = KF984780; *RPB2* = KF985018).

***Talaromyces juglandicola*** Jurjević & S.W. Peterson, Mycologia 109: 551. 2017. [MB818006]. — Type: BPI-910144. Ex-type: NRRL 32382. Infragen. class: sect. *Islandici*. Reproduction: asexual. ITS barcode: KX657330 (alternative markers: *BenA* = KX657122; *CaM* = KX657184; *RPB2* = KX657573).

***Talaromyces kabodanensis*** Houbraken *et al.*, Persoonia 37: 253. 2016. [MB819001]. — Type: CBS H-22857. Ex-type: CBS 139564 = DTO 204-F2 = CCTU 850. Infragen. class: sect. *Talaromyces*. Reproduction: asexual. ITS barcode: KP851981 (alternative markers: *BenA* = KP851986; *CaM* = KP851995; *RPB2* = MN969190).

***Talaromyces kendrickii*** Visagie *et al.*, Mycoscience 56: 493. 2015. [MB808235]. — Type: CBS H-21599. Ex-type: CBS 136666 = DTO 273-F4 = IBT 14128. Infragen. class: sect. *Talaromyces*. Reproduction: asexual. ITS barcode: KF741987 (alternative markers: *BenA* = KF741921; *CaM* = KF741967; *RPB2* = MN969158).

***Talaromyces kilbournensis*** Jurjević & S.W. Peterson, Mycologia 109: 545. 2017. [MB818000]. — Type: BPI-910153. Ex-type: NRRL 62700. Infragen. class: sect. *Islandici*. Reproduction: asexual. ITS barcode: KX657344 (alternative markers: *BenA* = KX657068; *CaM* = KX657183; *RPB2* = KX657545).

***Talaromyces lentulus*** X.Z. Jiang & L. Wang, Sci. Rep. 8: 4932, 3. 2018. [MB570522]. — Type: HMAS 247024. Ex-type: AS3.15689 = NN071323. Infragen. class: sect. *Talaromyces*. Reproduction: asexual. ITS barcode: KY007088 (alternative markers: *BenA* = KY007104; *CaM* = KY007096; *RPB2* = KY112586).

***Talaromyces liani*** (Kamyschko) N. Yilmaz *et al.*, Stud. Mycol. 78: 266. 2014. [MB809555]. Basionym: *Penicillium liani* Kamyschko, Not. Syst. Crypt. Inst. bot. Acad. Sci. USSR: 86. 1962. [MB335742]. — Type: unknown. Ex-type: CBS 225.66 = ATCC 18325 = ATCC 18331 = IMI 098480 = NRRL 3380 = VKM F-301. Infragen. class: sect. *Talaromyces*. Reproduction: homothallic. ITS barcode: JN899395 (alternative markers: *BenA* = JX091380; *CaM* = KJ885257; *RPB2* = KX961277).

***Talaromyces loliensis*** (Pitt) Samson *et al.*, Stud. Mycol. 71: 176. 2011. [MB560655]. Basionym: *Penicillium loliense* Pitt, Gen. Penicillium: 450. 1980. [MB116312]. — Type: IMI 216901. Ex-type: CBS 643.80 = ATCC 52252 = FRR 1798 = IMI 216901 = MUCL 31325. Infragen. class: sect. *Islandici*. Reproduction: asexual. ITS barcode: KF984888 (alternative markers: *BenA* = KF984658; *CaM* = KF984783; *RPB2* = KF985021).

***Talaromyces louisianensis*** Jurjević & S.W. Peterson, Fungal Biol. 123: 755. 2019. [MB827829]. — Type: BPI 910715. Ex-type: NRRL 35823. Infragen. class: sect. *Talaromyces*. Reproduction: asexual. ITS barcode: MH793052 (alternative markers: *BenA* = MH792924; *CaM* = MH792988; *RPB2* = MH793115).

***Talaromyces macrosporus*** (Stolk & Samson) Frisvad *et al.*, Antonie van Leeuwenhoek 57: 186. 1990. [MB126704]. Basionym: *Talaromyces flavus* var. *macrosporus* Stolk & Samson, Stud. Mycol 2: 15. 1972. [MB348888]. — Type: CBS H-7822. Ex-type: CBS 317.63 = FRR 404 = IMI 197478. Infragen. class: sect. *Talaromyces*. Reproduction: homothallic. ITS barcode: JN899333 (alternative markers: *BenA* = JX091382; *CaM* = KF741952; *RPB2* = KM023292).

***Talaromyces mae*** X.-Z. Jiang & L. Wang, Sci. Rep. 8: 4932, 4. 2018. [MB570523]. — Type: HMAS 247025. Ex-type: AS3.15690 = NN071328. Infragen. class: sect. *Talaromyces*. Reproduction: asexual. ITS barcode: KY007090 (alternative markers: *BenA* = KY007106; *CaM* = KY007098; *RPB2* = KY112588).

***Talaromyces malicola*** Jurjević & S.W. Peterson, Fungal Biol. 123: 756. 2019. [MB827830]. — Type: BPI 910712. Ex-type: NRRL 3724. Infragen. class: sect. *Talaromyces*. Reproduction: asexual. ITS barcode: MH909513 (alternative markers: *BenA* = MH909406; *CaM* = MH909459; *RPB2* = MH909567).

***Talaromyces mangshanicus*** X.C. Wang & W.Y. Zhuang, Mycol. Prog. 16:77. 2016. [MB570288]. — Type: HMAS 248733. Ex-type: CGMCC 3.18013. Infragen. class: sect. *Talaromyces*. Reproduction: asexual. ITS barcode: KX447531 (alternative markers: *BenA* = KX447530; *CaM* = KX447528; *RPB2* = KX447527).

***Talaromyces marneffei*** (Segretain *et al.*) Samson *et al.*, Stud. Mycol. 71: 176. 2011. [MB560656]. Basionym: *Penicillium marneffei* Segretain *et al.*, Bull. Soc. Mycol. France 75: 416. 1959. [MB335749]. — Type: IMI 68794iii. Ex-type: CBS 388.87 = ATCC 18224 = CBS 334.59 = IMI 068794ii = IMI 068794iii. Infragen. class: sect. *Talaromyces*. Reproduction: protoheterothallic (Woo *et al.* 2006). ITS barcode: JN899344 (alternative markers: *BenA* = JX091389; *CaM* = KF741958; *RPB2* = KM023283).

***Talaromyces mimosinus*** A.D. Hocking, Gen. Penicillium: 507. 1980. [MB116382]. — Type: IMI 223991. Ex-type: CBS 659.80 = FRR 1875 = IMI 223991. Infragen. class: sect. *Bacillispori*. Reproduction: homothallic. ITS barcode: JN899338 (alternative markers: *BenA* = KJ865726; *CaM* = KJ885272; *RPB2* = MN969149).

***Talaromyces minioluteus*** (Dierckx) Samson *et al.*, Stud. Mycol. 71: 176. 2011. [MB560657]. Basionym: *Penicillium minioluteum* Dierckx, Ann. Soc. Sci. Bruxelles. 25: 87. 1901. [MB157378]. — Type: CBS 642.68. Ex-type: CBS 642.68 = DTO 304-C4 = IMI 089377 = MUCL 28666. Infragen. class: sect. *Trachyspermi*. Reproduction: asexual. ITS barcode: JN899346 (alternative markers: *BenA* = MN969409; *CaM* = KJ885273; *RPB2* = JF417443).

***Talaromyces minnesotensis*** Guevara-Suarez *et al.*, Mycoses 60: 657. 2017. [MB820463]. — Type: CBS H-23001. Ex-type: UTHSC DI16-144 = CBS 142381 = FMR 14265. Infragen. class: sect. *Trachyspermi*. Reproduction: asexual. ITS barcode: LT558966 (alternative markers: *BenA* = LT559083; *CaM* = LT795604; *RPB2* = LT795605).

***Talaromyces muroii*** Yaguchi *et al.*, Mycoscience 35: 252. 1994. [MB362930]. — Type: CBM PF-1153. Ex-type: CBS 756.96 = PF 1153. Infragen. class: sect. *Talaromyces*. Reproduction: homothallic. ITS barcode: MN431394 (alternative markers: *BenA* = KJ865727; *CaM* = KJ885274; *RPB2* = KX961276).

***Talaromyces musae*** Houbraken *et al.*, Persoonia 39: 341. 2017. [MB821051]. — Type: CBS H-23138. Ex-type: CBS 142504 = DTO 366-C5. Infragen. class: sect. *Islandici*. Reproduction: asexual. ITS barcode: MF072316 (alternative markers: *BenA* = MF093729; *CaM* = MF093728; *RPB2* = MF093727).

***Talaromyces mycothecae*** R.N. Barbosa *et al.*, Antonie van Leeuwenhoek 111: 1902. 2018 [MB822215]. — Type: URM 90495. Ex-type: CBS 142494 = URM 7622. Infragen. class: sect. *Talaromyces*. Reproduction: asexual. ITS barcode: MF278326 (alternative markers: *BenA* = LT855561; *CaM* = LT855564; *RPB2* = LT855567).

***Talaromyces neofusisporus*** L. Wang, Sci. Rep. 6: 18622, 2. 2016. [MB811447]. — Type: HMAS 246033. Ex-type: CBS 139516 = DTO 410-D2 = AS 3.15415. Infragen. class: sect. *Talaromyces*. Reproduction: asexual. ITS barcode: KP765385 (alternative markers: *BenA* = KP765381; *CaM* = KP765383; *RPB2* = MN969165).

***Talaromyces neorugulosus*** A.J. Chen *et al.*, Stud. Mycol. 84:139. 2016 [MB817394]. — Type: CBS H-22511. Ex-type: CBS 140623 = CGMCC3.18215 = DTO 318-A8. Infragen. class: sect. *Islandici*. Reproduction: asexual. ITS barcode: KU866659 (alternative markers: *BenA* = KU866846; *CaM* = KU866743; *RPB2* = KU867003).

***Talaromyces novojersensis*** Jurjević & S.W. Peterson, Mycologia 109: 544. 2017. [MB817999]. — Type: BPI-910146. Ex-type: NRRL 35858. Infragen. class: sect. *Islandici*. Reproduction: asexual. ITS barcode: KX657319 (alternative markers: *BenA* = KX657050; *CaM* = KX657151; *RPB2* = KX657503).

***Talaromyces oumae-annae*** Visagie *et al.*, Stud. Mycol. 78: 130. 2014. [MB809187]. — Type: CBS H-21797. Ex-type: CBS 138208 = DTO 269-E8. Infragen. class: sect. *Talaromyces*. Reproduction: asexual. ITS barcode: KJ775720 (alternative markers: *BenA* = KJ775213; *CaM* = KJ775425; *RPB2* = KX961281).

***Talaromyces palmae*** (Samson *et al.*) Samson *et al.*, Stud. Mycol. 71: 176. 2011. [MB560658]. Basionym: *Penicillium palmae* Samson, Stolk & Frisvad, Stud. Mycol 31: 135. 1989. [MB125456]. — Type: CBS 442.88. Ex-type: CBS 442.88 = IMI 343640. Infragen. class: sect. *Subinflati*. Reproduction: asexual. ITS barcode: JN899396 (alternative markers: *BenA* = HQ156947; *CaM* = KJ885291; *RPB2* = KM023300).

***Talaromyces panamensis*** (Samson *et al.*) Samson *et al.*, Stud. Mycol. 71: 176. 2011. [MB560659]. Basionym: *Penicillium panamense* Samson, Stolk & Frisvad, Stud. Mycol. 31: 136. 1989. [MB125457]. — Type: CBS 128.89. Ex-type: CBS 128.89 = IMI 297546. Infragen. class: sect. *Talaromyces*. Reproduction: asexual. ITS barcode: JN899362 (alternative markers: *BenA* = HQ156948; *CaM* = KF741936; *RPB2* = KM023284).

***Talaromyces paucisporus*** (Yaguchi *et al.*) Samson & Houbraken, Stud. Mycol. 71: 176. 2011. [MB560684]. Basionym: *Erythrogymnotheca paucispora* Yaguchi *et al.*, Mycoscience 35: 219. 1994. [MB362926]. — Type: CBM-PF 1150. Ex-type: PF 1150 = IFM 53616. Infragen. class: sect. *Talaromyces*. Reproduction: homothallic. ITS barcode: AB176603 (alternative markers: *BenA* = n.a.; *CaM* = n.a.; *RPB2* = n.a.).

***Talaromyces pernambucoensis*** R. Cruz *et al.*, Persoonia 42: 467. 2019. [MB830189]. — Type: URM 93054. Ex-type: URM 6894. Infragen. class: sect. *Trachyspermi*. Reproduction: asexual. ITS barcode: LR535947 (alternative markers: *BenA* = LR535945; *CaM* = LR535946; *RPB2* = LR535948).

***Talaromyces piceus*** (Raper & Fennell) Samson *et al.*, Stud. Mycol. 71: 176. 2011. [MB560661]. Basionym: *Penicillium piceum* Raper & Fennell, Mycologia 40: 533. 1948. [MB289103]. — Type: IMI 040038. Ex-type: CBS 361.48 = ATCC 10519 = IMI 040038 = NRRL 1051. Infragen. class: sect. *Islandici*. Reproduction: asexual. ITS barcode: KF984792 (alternative markers: *BenA* = KF984668; *CaM* = KF984680; *RPB2* = KF984899).

***Talaromyces pigmentosus*** R.N. Barbosa *et al.*, Antonie van Leeuwenhoek 111: 1905. 2018 [MB822216]. — Type: URM 90496. Ex-type: CBS 142805 = URM 7624. Infragen. class: sect. *Helici*. Reproduction: asexual. ITS barcode: MF278330 (alternative markers: *BenA* = LT855562; *CaM* = LT855565; *RPB2* = LT855568).

***Talaromyces pinophilus*** (Hedgc.) Samson *et al.*, Stud. Mycol. 71: 176. 2011. [MB560662]. Basionym: *Penicillium pinophilum* Hedgc., U.S.D.A. Bur. Ani. Ind. Bull. 118: 37. 1910. [MB211142]. — Type: IMI 114933. Ex-type: CBS 631.66 = ATCC 36839 = CECT 2809 = DSM 1944 = IAM 7013 =IMI 114933. Infragen. class: sect. *Talaromyces*. Reproduction: protoheterothallic, both mating types detected (López-Villavicencio *et al.* 2010). ITS barcode: JN899382 (alternative markers: *BenA* = JX091381; *CaM* = KF741964; *RPB2* = KM023291).

***Talaromyces pittii*** (Quintan.) Samson *et al.*, Stud. Mycol. 71: 176. 2011. [MB560663]. Basionym: *Penicillium pittii* Quintan., Mycopathologia 91: 69. 1985. [MB105612]. — Type: CBS 139.84. Ex-type: CBS 139.84 = IMI 327871. Infragen. class: sect. *Purpurei*. Reproduction: asexual. ITS barcode: JN899325 (alternative markers: *BenA* = KJ865728; *CaM* = KJ885275; *RPB2* = KM023297).

***Talaromyces pratensis*** Jurjević & S.W. Peterson, Fungal Biol. 123: 756. 2019. [MB827831]. — Type: BPI 910710. Ex-type: NRRL 62170. Infragen. class: sect. *Talaromyces*. Reproduction: asexual. ITS barcode: MH793075 (alternative markers: *BenA* = MH792948; *CaM* = MH793012; *RPB2* = MH793139).

***Talaromyces primulinus*** (Pitt) Samson *et al.*, Stud. Mycol. 71: 176. 2011. [MB560664]. Basionym: *Penicillium primulinum* Pitt, The Genus Penicillium: 455. 1980 [1979]. [MB116330]. — Type: IMI 040031. Ex-type: CBS 321.48 = ATCC 10438 = CBS 439.88 = FRR 1074 = IMI 040031 = MUCL 31321 = MUCL 31330 = NRRL 1074. Infragen. class: sect. *Talaromyces*. Reproduction: asexual. ITS barcode: JN899317 (alternative markers: *BenA* = JX494305; *CaM* = KF741954; *RPB2* = KM023294).

***Talaromyces proteolyticus*** (Kamyschko) Samson *et al.*, Stud. Mycol. 71: 176. 2011. [MB560665]. Basionym: *Penicillium proteolyticum* Kamyschko, Nov. Sist. niz. Rast.: 228. 1961. [MB335760]. — Type: CBS 303.67. Ex-type: CBS 303.67 = ATCC 18326 = NRRL 3378. Infragen. class: sect. *Bacillispori*. Reproduction: asexual. ITS barcode: JN899387 (alternative markers: *BenA* = KJ865729; *CaM* = KJ885276; *RPB2* = KM023301).

***Talaromyces pseudofuniculosus*** Guevara-Suarez *et al.*, Fungal Syst. Evol. 5: 69. 2020. [MB822090]. — Type: CBS H-23214. Ex-type: CBS 143041 = FMR 15307. Infragen. class: sect. *Talaromyces*. Reproduction: asexual. ITS barcode: LT899796 (alternative markers: *BenA* = LT898323; *CaM* = LT899778; *RPB2* = LT899814).

***Talaromyces pseudostromaticus*** (Hodges *et al.*) Samson *et al.*, Stud. Mycol. 71: 176. 2011. [MB560666]. Basionym: *Penicillium pseudostromaticum* Hodges *et al.*, Mycologia 62: 1106. 1970. [MB319292]. — Type: Warner 18 (NY). Ex-type: CBS 470.70 = ATCC 18919 = FRR 2039. Infragen. class: sect. *Purpurei*. Reproduction: asexual. ITS barcode: JN899371 (alternative markers: *BenA* = HQ156950; *CaM* = KJ885277; *RPB2* = KM023298).

***Talaromyces ptychoconidius*** [as “*ptychoconidium*”] Visagie & K. Jacobs, Persoonia 28: 18. 2012. [MB626028]. — Type: PREM 60041. Ex-type: DAOM 241017 = CV 2808 = DTO 180-E7. Infragen. class: sect. *Purpurei*. Reproduction: asexual. ITS barcode: FJ160266 (alternative markers: *BenA* = GU385733; *CaM* = JX140701; *RPB2* = KM023278).

***Talaromyces purgamentorum*** N. Yilmaz *et al.*, Mycol. Prog. 15: 1054. 2016. [MB816233]. — Type: HUA 197222. Ex-type: CBS 113145 = IBT 23220 = DTO 056-E1. Infragen. class: sect. *Talaromyces*. Reproduction: asexual. ITS barcode: KX011504 (alternative markers: *BenA* = KX011487; *CaM* = KX011500; *RPB2* = MN969189).

***Talaromyces purpureogenus*** [as “*purpurogenus*”] (Stoll) Samson *et al.*, Stud. Mycol. 71: 177. 2011. [MB585068]. Basionym: *Penicillium purpureogenum* Stoll, Beitr. Charakt. Penicillium-Arten: 32. 1904. [MB585067]. — Type: IMI 091926. Ex-type: CBS 286.36 = IMI 091926. Infragen. class: sect. *Talaromyces*. Reproduction: asexual. ITS barcode: JN899372 (alternative markers: *BenA* = JX315639; *CaM* = KF741947; *RPB2* = JX315709).

***Talaromyces purpureus*** (E. Müll. & Pacha-Aue) Stolk & Samson, Stud. Mycol. 2: 57. 1972. [MB324420]. Basionym: *Arachniotus purpureus* E. Müll. & Pacha-Aue, Nova Hedwigia 15: 552. 1968. [MB326429]. — Type: CBS H-7832 (isotype). Ex-type: CBS 475.71 = ATCC 24069 = ATCC 52513 = FRR 1731 = IMI 181546. Infragen. class: sect. *Purpurei*. Reproduction: homothallic. ITS barcode: JN899328 (alternative markers: *BenA* = GU385739; *CaM* = KJ885292; *RPB2* = JN121522).

***Talaromyces qii*** L. Wang, Sci. Rep. 6: 18622, 2. 2016. [MB811448]. — Type: HMAS 246032. Ex-type: CBS 139515 = DTO 410-D1 = AS 3.15414. Infragen. class: sect. *Talaromyces*. Reproduction: asexual. ITS barcode: KP765384 (alternative markers: *BenA* = KP765380; *CaM* = KP765382; *RPB2* = MN969164).

***Talaromyces rademirici*** (Quintan.) Samson *et al.*, Stud. Mycol. 71: 177. 2011. [MB560668]. Basionym: *Penicillium rademirici* Quintan., Mycopathologia 91: 69. 1985. [MB105613]. — Type: CBS 140.84. Ex-type: CBS 140.84 = CECT 2771 = IMI 282406 = IMI 327870. Infragen. class: sect. *Purpurei*. Reproduction: asexual. ITS barcode: JN899386 (alternative markers: *BenA* = KJ865734; *CaM* = n.a.; *RPB2* = KM023302).

***Talaromyces radicus*** (A.D. Hocking & Whitelaw) Samson *et al.*, Stud. Mycol. 71: 177. 2011. [MB560669]. Basionym: *Penicillium radicum* A.D. Hocking & Whitelaw, Mycol. Res. 102: 802. 1998. [MB445182]. — Type: DAR 72374. Ex-type: CBS 100489 = FRR 4718. Infragen. class: sect. *Islandici*. Reproduction: asexual. ITS barcode: KF984878 (alternative markers: *BenA* = KF984599; *CaM* = KF984773; *RPB2* = KF985013).

***Talaromyces ramulosus*** (Visagie & K. Jacobs) Samson *et al.*, Stud. Mycol. 71: 177. 2011. [MB560670]. Basionym: *Penicillium ramulosum* Visagie & K. Jacobs, Mycologia 101: 890. 2008. [MB512023]. — Type: PREM 59947. Ex-type: DAOM 241660 = CV 2837 = DTO 184-B8. Infragen. class: sect. *Purpurei*. Reproduction: asexual. ITS barcode: EU795706 (alternative markers: *BenA* = FJ753290; *CaM* = JX140711; *RPB2* = KM023281).

***Talaromyces rapidus*** Guevara-Suarez *et al.*, Mycoses 60: 658. 2017. [MB820464]. — Type: CBS H-23002. Ex-type: UTHSC DI16-148 = CBS 142382 = FMR 14293. Infragen. class: sect. *Talaromyces*. Reproduction: asexual. ITS barcode: LT558970 (alternative markers: *BenA* = LT559087; *CaM* = LT795600; *RPB2* = LT795601).

***Talaromyces resedanus*** (McLennan & Ducker) A.J. Chen *et al.*, under review. [MB811695]. Basionym: *Penicillium resedanum* McLennan & Ducker, Australian J. Bot: 360. 1954. [MB302422]. — Type: IMI 62877. Ex-type: CBS 181.71 = DTO 376-A7 = ATCC 22356 = FRR 578 = IMI 062877 = NRRL 578. Infragen. class: sect. *Subinflati*. Reproduction: asexual. ITS barcode: MN431413 (alternative markers: *BenA* = MN969436; *CaM* = MN969355; *RPB2* = MN969214).

***Talaromyces resinae*** (Z.T. Qi & H.Z. Kong) Houbraken & X.C. Wang, this study. 2020. [MB833989]. Basionym: *Penicillium resinae* Z.T. Qi & H.Z. Kong, Acta Mycol. Sin.: 103. 1982. [MB110236]. — Type: HMAS 42799. Ex-type: AS 3.4387 = CBS 324.83 = DTO 027-G5. Infragen. class: sect. *Trachyspermi*. Reproduction: asexual. ITS barcode: MT079858 (alternative markers: *BenA* = MN969442; *CaM* = MT066184; *RPB2* = MN969221).

***Talaromyces reverso-olivaceus*** A.J. Chen *et al.*, Stud. Mycol. 84:141. 2016 [MB817391]. — Type: CBS H-22512. Ex-type: CBS 140672 = CGMCC 3.18195 = DTO 317-C3. Infragen. class: sect. *Helici*. Reproduction: asexual. ITS barcode: KU866646 (alternative markers: *BenA* = KU866834; *CaM* = KU866730; *RPB2* = KU866990).

***Talaromyces ricevillensis*** Jurjević & S.W. Peterson, Mycologia 109: 547. 2017. [MB818003]. — Type: BPI-910151. Ex-type: NRRL 62296. Infragen. class: sect. *Islandici*. Reproduction: asexual. ITS barcode: KX657343 (alternative markers: *BenA* = KX657056; *CaM* = KX657249; *RPB2* = KX657582).

***Talaromyces rogersiae*** Jurjević & S.W. Peterson, Mycologia 109: 550. 2017. [MB818005]. — Type: BPI-910149. Ex-type: NRRL 62223. Infragen. class: sect. *Islandici*. Reproduction: asexual. ITS barcode: KX657332 (alternative markers: *BenA* = KX657125; *CaM* = KF196891; *RPB2* = KX657581).

***Talaromyces rotundus*** (Raper & Fennell) C.R. Benj., Mycologia 47: 683. 1955. [MB306719]. Basionym: *Penicillium rotundum* Raper & Fennell, Mycologia 40: 518. 1948. [MB289106]. — Type: IMI 040589. Ex-type: CBS 369.48 = ATCC 10493 = IMI 040589 = NRRL 2107. Infragen. class: sect. *Islandici*. Reproduction: homothallic. ITS barcode: JN899353 (alternative markers: *BenA* = KJ865730; *CaM* = KJ885278; *RPB2* = KM023275).

***Talaromyces ruber*** (Stoll) N. Yilmaz *et al.*, Persoonia 29: 48. 2012. [MB801360]. Basionym: *Penicillium rubrum* Stoll, Beitr. Charakt. Penicillium-Arten: 35. 1904. [MB205727]. — Type: CBS H-21052. Ex-type: CBS 132704 = DTO 193-H6 = IBT 10703 = CBS 113137. Infragen. class: sect. *Talaromyces*. Reproduction: protoheterothallic, MAT1-1-1 detected (López-Villavicencio *et al.* 2010). ITS barcode: JX315662 (alternative markers: *BenA* = JX315629; *CaM* = KF741938; *RPB2* = JX315700).

***Talaromyces rubicundus*** (J.H. Mill. *et al.*) Samson *et al.*, Stud. Mycol. 71: 177. 2011. [MB560671]. Basionym: *Penicillium rubicundum* J.H. Mill., Giddens & A.A. Foster, Mycologia 49: 797. 1957. [MB302423]. — Type: No. 2531 (A.A. Foster). Ex-type: CBS 342.59 = ATCC 13217 = IMI 099723 = NRRL 3400. Infragen. class: sect. *Talaromyces*. Reproduction: asexual. ITS barcode: JN899384 (alternative markers: *BenA* = JX494309; *CaM* = KF741956; *RPB2* = KM023296).

***Talaromyces rugulosus*** (Thom) Samson *et al.*, Stud. Mycol. 71: 177. 2011. [MB560672]. Basionym: *Penicillium rugulosum* Thom, U.S.D.A. Bur. Ani. Ind. Bull. 118: 60. 1910. [MB210907]. — Type: IMI 040041. Ex-type: CBS 371.48 = ATCC 10128 = IMI 040041 = MUCL 31201 = NRRL 1045. Infragen. class: sect. *Islandici*. Reproduction: asexual. ITS barcode: KF984834 (alternative markers: *BenA* = KF984575; *CaM* = KF984702; *RPB2* = KF984925).

***Talaromyces ryukyuensis*** (S. Ueda & Udagawa) Arx, Persoonia 13: 282. 1987. [MB132096]. Basionym: *Sagenoma ryukyuense* S. Ueda & Udagawa, Mycotaxon 20: 499. 1984. [MB124389]. — Type: unknown. Ex-type: NHL 2917 = DTO 176-I6. Infragen. class: sect. *Helici*. Reproduction: homothallic. ITS barcode: AB176628 (alternative markers: *BenA* = n.a.; *CaM* = n.a.; *RPB2* = n.a.).

***Talaromyces sayulitensis*** Visagie *et al.*, Stud. Mycol. 78: 132. 2014. [MB809188]. — Type: CBS H-21798. Ex-type: CBS 138204 = DTO 245-H1. Infragen. class: sect. *Talaromyces*. Reproduction: asexual. ITS barcode: KJ775713 (alternative markers: *BenA* = KJ775206; *CaM* = KJ775422; *RPB2* = MN969146).

***Talaromyces scorteus*** (Nakazawa *et al.*) S.W. Peterson & Jurjević, PLoS ONE 8: e78084, 8. 2013. [MB804734]. Basionym: *Penicillium scorteum* Nakazawa *et al.*, J. Agric. Chem. Soc. Japan: 103. 1934. [MB492647]. — Type: unknown. Ex-type: CBS 340.34 = NRRL 1129 = FRR 1129. Infragen. class: sect. *Islandici*. Reproduction: asexual. ITS barcode: KF984892 (alternative markers: *BenA* = KF984565; *CaM* = KF984684; *RPB2* = KF984916).

***Talaromyces siamensis*** (Manoch & C. Ramírez) Samson *et al.*, Stud. Mycol. 71: 177. 2011. [MB560674]. Basionym: *Penicillium siamense* Manoch & C. Ramírez, Mycopathologia 101: 32. 1988. [MB133414]. — Type: CBS 475.88. Ex-type: CBS 475.88 = IMI 323204. Infragen. class: sect. *Talaromyces*. Reproduction: asexual. ITS barcode: JN899385 (alternative markers: *BenA* = JX091379; *CaM* = KF741960; *RPB2* = KM023279).

***Talaromyces siglerae*** S.W. Peterson & Jurjević, Mycologia 109: 540. 2017. [MB818002]. — Type: BPI-910143. Ex-type: NRRL 28620. Infragen. class: sect. *Islandici*. Reproduction: asexual. ITS barcode: KX657351 (alternative markers: *BenA* = KX657135; *CaM* = KX657236; *RPB2* = KX657497).

***Talaromyces soli*** Jurjević & S.W. Peterson, Fungal Biol. 123: 757. 2019. [MB827832]. — Type: BPI 910709. Ex-type: NRRL 62165. Infragen. class: sect. *Talaromyces*. Reproduction: asexual. ITS barcode: MH793074 (alternative markers: *BenA* = MH792947; *CaM* = MH793011; *RPB2* = MH793138).

***Talaromyces solicola*** Visagie & K. Jacobs, Persoonia 28: 20. 2012. [MB564328]. — Type: PREM 60037. Ex-type: DAOM 241015 = CV 2800 = DTO 180-D4. Infragen. class: sect. *Trachyspermi*. Reproduction: asexual. ITS barcode: FJ160264 (alternative markers: *BenA* = GU385731; *CaM* = KJ885279; *RPB2* = KM023295).

***Talaromyces speluncarum*** Rodr.-Andr. *et al.*, Microorganisms 2020, 8, 12: 10. 2020 [MB830606]. — Type: CBS H-23372. Ex-type: CBS 143844 = FMR 16671. Infragen. class: sect. *Trachyspermi*. Reproduction: asexual. ITS barcode: LT985890 (alternative markers: *BenA* = LT985901; *CaM* = LT985906; *RPB2* = LT985911).

***Talaromyces stellenboschensis*** [as “*stellenboschiensis*”] Visagie & K. Jacobs, Mycoscience 56:497. 2015. [MB834764]. — Type: CBS H-21601. Ex-type: CBS 135665 = DTO 181-A2 = DAOM 241021 = IBT 32631. Infragen. class: sect. *Talaromyces*. Reproduction: asexual. ITS barcode: JX091471 (alternative markers: *BenA* = JX091605; *CaM* = JX140683; *RPB2* = MN969157).

***Talaromyces stipitatus*** (Thom) C.R. Benj., Mycologia 47: 684. 1955. [MB306722]. Basionym: *Penicillium stipitatum* Thom, Mycologia 27: 138. 1935. [MB119544]. — Type: CBS H-7835. Ex-type: CBS 375.48 = ATCC 10500 = NRRL 1006 = IMI 39805. Infragen. class: sect. *Talaromyces*. Reproduction: homothallic. ITS barcode: JN899348 (alternative markers: *BenA* = KM111288; *CaM* = KF741957; *RPB2* = KM023280).

***Talaromyces stollii*** N. Yilmaz *et al.*, Persoonia 29: 52. 2012. [MB801359]. — Type: CBS H-21053. Ex-type: CBS 408.93. Infragen. class: sect. *Talaromyces*. Reproduction: asexual. ITS barcode: JX315674 (alternative markers: *BenA* = JX315633; *CaM* = JX315646; *RPB2* = JX315712).

***Talaromyces striatoconidius*** (R.F. Castañeda & W. Gams) Houbraken *et al.*, this study. 2020. [MB832566]. Replaced synonym: *Phialomyces striatus* R.F. Castañeda & W. Gams, Mycotaxon 42: 239. 1991. [MB358862]. — Type: INIFAT C89/170. Ex-type: CBS 550.89 = DTO 418-H4. Infragen. class: sect. *Talaromyces*. Reproduction: asexual. ITS barcode: MN431418 (alternative markers: *BenA* = MN969441; *CaM* = MN969360; *RPB2* = MT156347).

***Talaromyces subaurantiacus*** Visagie *et al.*, Persoonia 36: 52. 2016. [MB810902]. — Type: CBS H-21630. Ex-type: CBS 137383 = DTO 181-I2 = DAOM 241020 = IBT 32383. Infragen. class: sect. *Islandici*. Reproduction: asexual. ITS barcode: JX091475 (alternative markers: *BenA* = JX091609; *CaM* = JX140728; *RPB2* = KF984960).

***Talaromyces subericola*** Rodr.-Andr. *et al.*, Microorganisms 2020, 8, 12: 12. 2020 [MB830607]. — Type: CBS H-23366. Ex-type: CBS 144322 = FMR 15656. Infragen. class: sect. *Trachyspermi*. Reproduction: asexual. ITS barcode: LT985888 (alternative markers: *BenA* = LT985899; *CaM* = LT985904; *RPB2* = LT985909).

***Talaromyces subinflatus*** Yaguchi & Udagawa, Trans. Mycol. Soc. Japan 34: 249. 1993. [MB361184]. — Type: CBM PF-1113. Ex-type: CBS 652.95 = DTO 105-D5 = IBT 17520. Infragen. class: sect. *Subinflati*. Reproduction: homothallic. ITS barcode: JN899397 (alternative markers: *BenA* = MK450890; *CaM* = KJ885280; *RPB2* = KM023308).

***Talaromyces subtropicalis*** Jurjević & S.W. Peterson, Mycologia 109: 548. 2017. [MB818004]. — Type: BPI-910147. Ex-type: NRRL 58084. Infragen. class: sect. *Islandici*. Reproduction: asexual. ITS barcode: KX657337 (alternative markers: *BenA* = KX657060; *CaM* = KX657250; *RPB2* = KX657531).

***Talaromyces systylus*** S.M. Romero *et al.*, Nova Hedwigia 102: 244. 2016. [MB812381]. — Type: BAFC 52367. Ex-type: BAFCcult 3419. Infragen. class: sect. *Trachyspermi*. Reproduction: asexual. ITS barcode: KP026917 (alternative markers: *BenA* = KR233838; *CaM* = KR233837; *RPB2* = n.a.).

***Talaromyces tabacinus*** Jurjević *et al.*, Persoonia 40: 323. 2018. [MB823318]. — Type: BPI 910533. Ex-type: NRRL 66727 = EMSL 2174. Infragen. class: sect. *Helici*. Reproduction: asexual. ITS barcode: MG182613 (alternative markers: *BenA* = MG182627; *CaM* = MG182606; *RPB2* = MG182620).

***Talaromyces tardifaciens*** Udagawa, Mycotaxon 48: 150. 1993. [MB360478]. — Type: CBM SUM 3017. Ex-type: CBS 250.94. Infragen. class: sect. *Islandici*. Reproduction: homothallic. ITS barcode: JN899361 (alternative markers: *BenA* = KF984560; *CaM* = KF984682; *RPB2* = KF984908).

***Talaromyces thailandensis*** Manoch *et al.*, Mycoscience 54: 339. 2013. [MB801737]. — Type: CBS H-21075. Ex-type: CBS 133147 = KUFC 3399. Infragen. class: sect. *Talaromyces*. Reproduction: homothallic. ITS barcode: JX898041 (alternative markers: *BenA* = JX494294; *CaM* = KF741940; *RPB2* = KM023307).

***Talaromyces tiftonensis*** Jurjević & S.W. Peterson, Mycologia 109: 552. 2017. [MB818008]. — Type: BPI-910150. Ex-type: NRRL 62264. Infragen. class: sect. *Islandici*. Reproduction: asexual. ITS barcode: KX657353 (alternative markers: *BenA* = KX657129; *CaM* = KX657163; *RPB2* = KX657602).

***Talaromyces trachyspermus*** (Shear) Stolk & Samson, Stud. Mycol. 2: 32. 1972. [MB324421]. Basionym: *Arachniotus trachyspermus* Shear, Science New York 16: 138. 1902. [MB164396]. — Type: IMI 040043. Ex-type: CBS 373.48 = ATCC 10497 = IMI 040043 = NRRL 1028. Infragen. class: sect. *Trachyspermi*. Reproduction: homothallic. ITS barcode: JN899354 (alternative markers: *BenA* = KF114803; *CaM* = KJ885281; *RPB2* = JF417432).

***Talaromyces tratensis*** Manoch *et al.*, Mycoscience 54: 337. 2013. [MB801738]. — Type: CBS H-21074. Ex-type: CBS 133146 = KUFC 3383. Infragen. class: sect. *Islandici*. Reproduction: homothallic. ITS barcode: KF984891 (alternative markers: *BenA* = KF984559; *CaM* = KF984690; *RPB2* = KF984911).

***Talaromyces tumuli*** Jurjević & S.W. Peterson, Fungal Biol. 123: 758. 2019. [MB827833]. — Type: BPI 910713. Ex-type: NRRL 62151. Infragen. class: sect. *Talaromyces*. Reproduction: asexual. ITS barcode: MH793071 (alternative markers: *BenA* = MH792944; *CaM* = MH793008; *RPB2* = MH793135).

***Talaromyces tzapotlensis*** Jurjević & S.W. Peterson, Mycologia 109: 553. 2017. [MB818009]. — Type: BPI-910145. Ex-type: NRRL 35203. Infragen. class: sect. *Subinflati*. Reproduction: asexual. ITS barcode: KX946902 (alternative markers: *BenA* = KX946884; *CaM* = KX946893; *RPB2* = KX946922).

***Talaromyces ucrainicus*** (Panas.) Udagawa, Trans. Mycol. Soc. Japan 7: 94. 1966. [MB449587]. Basionym: *Penicillium ucrainicum* Panas., Mycologia 56: 59. 1964. [MB335773]. — Type: unknown. Ex-type: CBS 162.67 = ATCC 22344 = FRR 3462 = NHL 6086. Infragen. class: sect. *Trachyspermi*. Reproduction: homothallic. ITS barcode: JN899394 (alternative markers: *BenA* = KF114771; *CaM* = KJ885282; *RPB2* = KM023289).

***Talaromyces udagawae*** Stolk & Samson, Stud. Mycol. 2: 36. 1972. [MB324424]. — Type: CBS H-7841. Ex-type: CBS 579.72 = FRR 1727 = IMI 197482. Infragen. class: sect. *Trachyspermi*. Reproduction: homothallic. ITS barcode: JN899350 (alternative markers: *BenA* = KF114796; *CaM* = KX961260; *RPB2* = MN969148).

***Talaromyces unicus*** Tzean *et al.*, Mycologia 84: 739. 1992. [MB360172]. — Type: PPH 16 Nat. Taiwan Univ. Ex-type: CBS 100535 = CCRC 32703 = IBT 18385. Infragen. class: sect. *Bacillispori*. Reproduction: homothallic. ITS barcode: JN899336 (alternative markers: *BenA* = KJ865735; *CaM* = KJ885283; *RPB2* = MN969150).

***Talaromyces varians*** (G. Sm.) Samson *et al.*, Stud. Mycol. 71: 177. 2011. [MB560677]. Basionym: *Penicillium varians* G. Sm., Trans. Brit. Mycol. Soc. 18: 89. 1933. [MB289112]. — Type: IMI 040586. Ex-type: CBS 386.48 = ATCC 10509 = IMI 040586 = NRRL 2096. Infragen. class: sect. *Helici*. Reproduction: asexual. ITS barcode: JN899368 (alternative markers: *BenA* = KJ865731; *CaM* = KJ885284; *RPB2* = KM023274).

***Talaromyces veerkampii*** Visagie *et al.*, Mycoscience 56: 497. 2015. [MB808233]. — Type: CBS H-21600. Ex-type: CBS 500.78; DTO 258-I8; IBT 14845; IBT 32648. Infragen. class: sect. *Talaromyces*. Reproduction: asexual. ITS barcode: KF741984 (alternative markers: *BenA* = KF741918; *CaM* = KF741961; *RPB2* = KX961279).

***Talaromyces verruculosus*** (Peyronel) Samson *et al.*, Stud. Mycol. 71: 177. 2011. [MB560678]. Basionym: *Penicillium verruculosum* Peyronel, I germi astmosferici dei fungi con micelio, Diss. (Padova): 22. 1913. [MB166576]. — Type: IMI 040039. Ex-type: DTO 264-I8 = CBS 136671 = ATCC 10513 = DSM 2263 = IMI 040039 = NRRL 1050. Infragen. class: sect. *Talaromyces*. Reproduction: asexual. ITS barcode: KF741994 (alternative markers: *BenA* = KF741928; *CaM* = KF741944; *RPB2* = KM023306).

***Talaromyces versatilis*** Bridge & Buddie, Index Fungorum 26: 1. 2013. [MB550190]. — Type: IMI 134755. Ex-type: DTO 326-B7 = IMI 134755. Infragen. class: sect. *Talaromyces*. Reproduction: asexual. ITS barcode: MN431395 (alternative markers: *BenA* = MN969412; *CaM* = MN969319; *RPB2* = MN969161).

***Talaromyces viridis*** (Stolk & G.F. Orr) Arx, Persoonia 13: 2821. 1987. [MB132097]. Basionym: *Sagenoma viride* Stolk & G.F. Orr, Mycologia 66: 677. 1974. [MB323033]. — Type: CBS H-7732 (isotype), CBS H-7733 (isotype), CBS H-7734 (isotype). Ex-type: CBS 114.72 = ATCC 22467 = NRRL 5575. Infragen. class: sect. *Talaromyces*. Reproduction: homothallic. ITS barcode: AF285782 (alternative markers: *BenA* = JX494310; *CaM* = KF741935; *RPB2* = JN121430).

***Talaromyces viridulus*** Samson *et al.*, Stud. Mycol. 71: 177. 2011. [MB560679]. — Type: FRR 1863. Ex-type: CBS 252.87 = FRR 1863 = IMI 288716. Infragen. class: sect. *Talaromyces*. Reproduction: asexual. ITS barcode: JN899314 (alternative markers: *BenA* = JX091385; *CaM* = KF741943; *RPB2* = JF417422).

***Talaromyces wortmannii*** (Klöcker) C.R. Benj., Mycologia 47: 683. 1955. [MB344294]. Basionym: *Penicillium wortmannii* Klöcker, Compt. Rend. Trav. Lab. Carlsberg: serie Physiologique 6: 100. 1903. [MB119648]. — Type: IMI 040047. Ex-type: CBS 391.48 = ATCC 10517 = IMI 040047 = NRRL 1017. Infragen. class: sect. *Islandici*. Reproduction: homothallic. ITS barcode: KF984829 (alternative markers: *BenA* = KF984648; *CaM* = KF984756; *RPB2* = KF984977).

***Talaromyces xishaensis*** X.C. Wang *et al.*, Phytotaxa 267: 193. 2016. [MB815751]. — Type: HMAS 248732. Ex-type: CGMCC 3.17995. Infragen. class: sect. *Talaromyces*. Reproduction: asexual. ITS barcode: KU644580 (alternative markers: *BenA* = KU644581; *CaM* = KU644582; *RPB2* = n.a.).

***Talaromyces yelensis*** Visagie *et al.*, Stud. Mycol. 78: 134. 2014. [MB809189]. — Type: CBS H-21799. Ex-type: CBS 138209 = DTO 268-E5. Infragen. class: sect. *Islandici*. Reproduction: asexual. ITS barcode: KJ775717 (alternative markers: *BenA* = KJ775210; *CaM* = KP119161; *RPB2* = KP119163).  

***Thermoascus***

***Thermoascus aegyptiacus*** S. Ueda & Udagawa, Trans. Mycol. Soc. Japan 24: 135. 1983. [MB108584]. — Type: NHL 2914. Ex-type: DTO 424-H2 = CBS 146342 = NHL 2914 = IFM 61569. Reproduction: homothallic. ITS barcode: MT068197 (alternative markers: *BenA* = MN969411; *CaM* = MN969318; *RPB2* = n.a.).

***Thermoascus aurantiacus*** Miehe, Die Selbsterhitzung des Heus, Jena: 70. 1907. [MB122028]. — Type: UC M206516. Ex-type: n.a. Reproduction: homothallic. ITS barcode: EU021617 (alternative markers: *BenA* = EU021667; *CaM* = n.a.; *RPB2* = EU021657).

***Thermoascus crustaceus*** (Apinis & Chesters) Stolk, Antonie van Leeuwenhoek 31: 272. 1965. [MB340045]. Basionym: *Dactylomyces crustaceus* Apinis & Chesters, Trans. Brit. Mycol. Soc. 47: 428. 1964. [MB329643]. — Type: IMI 102470. Ex-type: IMI 102470 = BDUN 378. Reproduction: homothallic. ITS barcode: MN431405 (alternative markers: *BenA* = MN969423; *CaM* = MN969344; *RPB2* = MN969205).

***Thermoascus taitungiacus*** K.Y. Chen & Z.C. Chen, Mycotaxon 60: 226. 1996. [MB436720]. — Type: TAl-Mycology K-Y Chen 8709-2. Ex-type: n.a. Reproduction: homothallic. ITS barcode: n.a. (alternative markers: *BenA* = n.a.; *CaM* = n.a.; *RPB2* = n.a.).

***Thermoascus thermophilus*** (Sopp) Arx, The genera of fungi sporulating in pure culture: 94. 1974. [MB324541]. Basionym: *Dactylomyces thermophilus* Sopp, Skr. VidenskSelsk. Christiania, Kl. I, Math.-Natur. (no. 11): 35. 1912. [MB224271]. — Type: CBS 528.71. Ex-type: CBS 528.71 = BDUN 394 = IMI 123298 = NRRL 5208. Reproduction: homothallic. ITS barcode: MH860254 (alternative markers: *BenA* = MN969424; *CaM* = MN969345; *RPB2* = JF417442).

***Thermoascus verrucosus*** (Samson & Tansey) Houbraken *et al.*, this study. 2020. [MB832567]. Basionym: *Byssochlamys verrucosa* Samson & Tansey, Trans. Brit. Mycol. Soc. 65: 512. 1975. [MB310015]. — Type: CBS 605.74. Ex-type: CBS 605.74 = ATCC 34163. Reproduction: homothallic. ITS barcode: DQ073329 (alternative markers: *BenA* = MN969425; *CaM* = MN969346; *RPB2* = JN121540).

***Thermoascus yaguchii*** (Yaguchi *et al.*) Houbraken *et al.*, this study. 2020. [MB833988]. Replaced synonym: *Thermoascus crustaceus* var. *verrucosus* Yaguchi *et al.*, Mycoscience 36: 151. 1995. [MB414278]. — Type: PF-1160. Ex-type: DTO 424-H3 = CBS 146343 = PF-1160 = IFM 66000. Reproduction: homothallic. ITS barcode: MT068196 (alternative markers: *BenA* = MT070995; *CaM* = MN969362; *RPB2* = n.a.).  

***Thermomyces***

***Thermomyces dupontii*** (Griffon & Maubl.) Houbraken & Samson, Adv. Appl. Microbiol. 86: 218. 2014. [MB805186]. Basionym: *Penicillium dupontii* Griffon & Maubl., Bull. Soc. Mycol. France 27: 73. 1911. [MB120357]. — Type: CBS 236.58. Ex-type: CBS 236.58 = ATCC 10518 = ATCC 16461 = ATCC 52514 = IFO 31798 = IMI 048593 = NRRL 2155. Reproduction: homothallic (*fide* Emerson, in Raper & Thom 1949). ITS barcode: MN431410 (alternative markers: *BenA* = MN969432; *CaM* = MT066185; *RPB2* = JF417420).

***Thermomyces lanuginosus*** Tsikl., Ann. Inst. Pasteur 13: 500. 1899. [MB239786]. — Type: IMI 84400 (Pugh *et al.*). Ex-type: CBS 632.91 = DTO 024-B3 = ATCC 16455 = DAOM 232588 (representative). Reproduction: asexual. ITS barcode: MN431411 (alternative markers: *BenA* = MN969433; *CaM* = MN969353; *RPB2* = MN969209).  

***Trichocoma***

***Trichocoma paradoxa*** Jungh., Praemissa in floram cryptogamicam Javae insulae: 9. 1838. [MB161024]. — Type: "*Trichocoma paradoxa*, Junghuhn (BO)". Ex-type: CBS 247.57, CBS 103.73, CBS 788.83 (representative strains). Reproduction: homothallic. ITS barcode: MH860643 (alternative markers: *BenA* = JF417469; *CaM* = JF417506; *RPB2* = JN121417).  

***Warcupiella***

***Warcupiella spinulosa*** (Warcup) Subram., Curr. Sci. 41: 757. 1972. [MB325542]. Basionym: *Aspergillus spinulosus* Warcup, Gen. Aspergillus: 204. 1965. [MB326657]. — Type: IMI 75885. Ex-type: CBS 512.65 = DTO 021-H3 = ATCC 16919 = IFO 31800 = IMI 075885 = IMI 075885ii = IMI 075885iii = IMI 075885iv = NRRL 4376 = WB 4376. Reproduction: homothallic. ITS barcode: EF669706 (alternative markers: *BenA* = MN969410; *CaM* = EF669698; *RPB2* = EF669666).  

***Xerochrysium***

***Xerochrysium dermatitidis*** (A. Agostini) Pitt, IMA Fungus 4: 237. 2013. [MB807005]. Basionym: *Glenosporella dermatitidis* A. Agostini, Atti Ist. Bot. R. Univ. Pavia, 3 Sér. 4: 98. 1931. [MB536220]. — Type: CBS 132.31 (Pitt *et al.* 2013). Ex-type: DTO 375-C6 = IMI 96729 = UAMH 802 = FRR 2376. Reproduction: asexual. ITS barcode: KY635853 (alternative markers: *BenA* = MT070993; *CaM* = MT066186; *RPB2* = JN121443).

***Xerochrysium xerophilum*** (Pitt) Pitt, IMA Fungus 4: 238. 2013. [MB807006]. Basionym: *Chrysosporium xerophilum* Pitt, Trans. Brit. Mycol. Soc. 49: 468. 1966. [MB328223]. — Type: UAMH 2368. Ex-type: CBS 153.67 = FRR 503 = ATCC 18053 = IMI 126287. Reproduction: asexual. ITS barcode: GU733339 (alternative markers: *BenA* = n.a.; *CaM* = n.a.; *RPB2* = n.a.).  

***Xeromyces***

***Xeromyces bisporus*** L.R. Fraser, Proc. Linn. Soc. New South Wales 78: 245. 1953. [MB307870]. — Type: unknown. Ex-type: CBS 236.71 = IMI 063718 = VKM F-1978 = FRR 525. Reproduction: homothallic. ITS barcode: KY635854 (alternative markers: *BenA* = KY709179; *CaM* = Genome sequence; CCCX01000040.1:6800–8000; *RPB2* = JN121466).

## General discussion

### Infrageneric classification

Dierckx (1901) proposed the first infrageneric classification of *Penicillium*. Various other monographs used an infrageneric classification to sort *Aspergillus* and *Penicillium* species in groups. The use of subgenera and sections are therefore well-established for these genera; however, a series classification based on sequence data was lacking. This was considered problematic as both *Aspergillus* and *Penicillium* have grown significantly in species numbers the past few years. To make working with these genera and sections easier, various taxonomic studies often refer to a “clade” or a “species complex”. Here we show that series are often the equivalents of these “clades” and “species complexes”. The use of a formal series classification makes working with large speciose genera easier and stabilises its taxonomy. As mentioned in the introduction, the allocation of a species to a series can be highly predictive in what functional characters the species might have. On the other hand, these functional characters are not known in various series (*e.g*., in *Aspergillus* sect. *Fumigati*) and future studies might reveal those. Furthermore, the application of a series classification might be useful when using a phenotype-based identification. Phenotypic identification of strains on species level can be difficult and identification on series rank can be a suitable alternative: a series name can be used for a phylogenetically defined complex of species that are morphological highly similar. An example is ser. *Versicolores* in *Aspergillus*, which includes commonly occurring species that are morphologically highly similar and usually have a similar impact or function. Species in *Penicillium* and *Aspergillus* series are in many cases phenotypically recognizable, for example by colony obverse and reverse colours, colony texture (for example by image analysis; (Dörge *et al.* 2000), ecophysiological features, nutritional features (Ahmad & Malloch 1999), growth on alternative nitrogen sources and enzyme profiles (Barrett *et al.* 2020). Until now, profiles of CAZymes appear to be very promising to be used for classification of species into sections, as these are congruent with a phylogenetic cladification (Barrett *et al.* 2020); it is likely that these methods will be applicable on series as well. While most series are phylogenetically recognizable, there were some examples of a more provisional designation of series (*e.g*., series in *Aspergillus* sect. *Fumigati*; series *Claviformia*, *Glandicolarum* and *Longicatenata* in *Penicillium*). Genome sequence data will help circumscribing such series more accurately (Vesth et al., 2018, Kjærbølling et al., 2020), preferably using a comprehensive phenotypic characterization on top of the phylogenetic characterization.

It has been an experience that species in filamentous fungi are clear cut with no intergrading strains. Only a few hybrids have ever been reported in fungal taxonomy. Of course, individual characters may be overlapping, such as the size, form and ornamentation of conidia or growth and conidiation as related to temperature, but a large number of characters are non-overlapping. This is especially true concerning small molecule extrolites characters (Frisvad *et al.* 2008). Species are thus stable for many years (punctuated equilibria), and may then speciate under certain circumstances (Gould and Eldredge, 1977, Gould and Eldredge, 1993, Zander, 2010). Thus, selection can not only work at the individual level but also at the species level (Gould 1998). However, there is one problem with the use of clear non-overlapping characters: these characters are not necessarily present in all isolates of a species or all species in a series, section, subgenus or genus. This phenomenon may be caused by horizontal transfer of genes or gene clusters, epigenetical factors or mutation-based loss of certain genes or cluster. For this reason, the concept of polythetic classes is very valuable, *i.e.* that any individual character is present in most, but not all members of the class and that most members of the class also possess any individual character. Polythetic classes can be found by a character analysis (cluster analysis) of several operational taxonomic units (OTUs) using several measured features, but not by a cladistic analysis. Thus, while cladistic analysis appear to be optimal for DNA sequence data, cluster analysis appears to be most effective for phenotypic characters.

In some cases, small molecule extrolites (secondary metabolites) support the species relationship in a series, in other cases this is not supported, at least not with currently available data. Genome sequencing may help in determining if cryptic gene clusters for secondary metabolites will support the species series as proposed by household gene sequence data, apart from the extrolites that are actually produced on agar media. For example, citreoviridin production is present among the two species in *Penicillium* series *Ochrosalmonea*: while one species is slow-growing and produces no synnemata (*P*. *ochrosalmoneum*), the other species grows fast and produces synnemata (*P*. *isariiforme*). Citreoviridin is also produced by species in other series, and therefore ideally several secondary metabolite families need to be characterised for each series. More data on physiology such as water activity relations, temperature, pH and exoenzyme profiles may help to circumscribe the series phenotypically in a more accurate way and help discriminate between series. Examples of series that are both phylogenetically and phenotypically consistent are series *Viridicata* and *Roquefortorum*, and in sect. *Sclerotiorum*, series *Adametziorum*, *Herqueorum* and *Sclerotiorum* where each have unique extrolites typical of the series (sclerotiorins, herqueinones and dithiodiketopiperazines, respectively).

### The fundamental species level

The species is the fundamental unit of taxonomy of fungi (Kirk *et al.* 2008) reflecting both evolution and ecology (Rollo, 1994, Sneath, 1995, Westoby et al., 1995, West-Eberhard, 2005). For this reason, as many unweighted characters as possible should be used in classification and for discovering species according to Sneath and Sokal, 1962, Sneath and Sokal, 1973 and Sneath (1995). This taxometric concept has been used for many years, even though the weighting of the characters has been discussed, like many characters, for example, chemotaxonomic characters based on the primary metabolism, that is shared with most other species and are constantly and quantitatively in flux, are hardly useful at all in a numerical taxonomy. For this reason it was proposed to use a weighted omnispective (Blackwelder 1964), polyphasic (Vandamme *et al.* 1996) or consilient (as consolidated) (Quaedvlieg *et al.* 2014) approach in discovering species based on different kinds of distinctive characters, especially morphological, nutritional, physiological, chemical and molecular (sequence-based) features. Following Hennig (1966), many scientists have emphasised synapomorphic features only, but using this approach in a strict sense will result in many important features not being considered in taxonomy (Sneath 1995).

For many years sequencing of household and other genes has been used solely to develop a cladification of isolates or species using the DNA sequence-based phylogenetic species recognition approach based on concordance of multiple gene genealogies (Taylor *et al.* 2000), but this approach does not take into account anagenesis (Mayr, 1981, Mayr, 1995), the functional phenotype (Rollo 1994) or epigenetics (Zander 2010). Thus, a polyphasic approach to taxonomy using symplesiomorphies, synapomorphies, and autapomorphies in conjunction with quantitative ecophysiological characters and sequence-based analyses appears to be an effective approach to discover species. The removal of autapomorphies in cladistic analyses has been criticised by Yeates (1992) and those autapomorphies are clear species-specific features. The autapomorphies may also help explain anagenetic evolution. Recently genome sequencing has added a lot of potentially new features, provided these features can be annotated, but this genome-based approach does not directly take into account epigenetics and phenotypic evolution (Rollo, 1994, Schlichting and Pigliucci, 1998, West-Eberhard, 2003, West-Eberhard, 2005). However, genomics, in conjunction with transcriptomics and exometabolomics, will undoubtedly be extremely valuable in taxonomy and cladonomy (Uilenberg et al., 2004, Zhi et al., 2012, Thompson et al., 2015). The revision of the species, series and sections in *Aspergillus* and *Penicillium* was guided by sequences of several primary metabolism “house-hold” genes. The results are often strongly supported by phenotypic features, showing that a combination of phenomics and genomics (a polyphasic approach) is needed for a predictive cladification and classification. Often series suggested by early phenotypic studies were confirmed by phylogenetic data, but in other cases, surprising relationships were encountered that were not expected from pure morphological data (for example, see Frisvad & Samson 2004b). It is crucial to take all important phenotypic features into account, as it is rarely the case that one type of phenotypic features can be used for classification that is in agreement with a sequence-based cladification. Until now the most predictive classification was based on profiles of function / family annotated CAZymes (Barrett *et al.* 2020), but in-depth comparative studies on the genomes and phenomes of *Aspergillus*, *Penicillium* and *Talaromyces* species will help suggesting a highly predictive taxonomy and phylogeny in the future.

## Dedication and acknowledgments

This work is dedicated to Keith A. Seifert for his invaluable contributions to the taxonomy of the *Eurotiales* on the occasion of his retirement from Ottawa Research and Development Centre, Agriculture and Agri-Food Canada (Biodiversity: Mycology and Microbiology). Jens Frisvad thanks Agilent for a Thought leader award from Agilent (# 2871) and support from the 10.13039/501100001732Danish National Research Foundation for the center of excellence CeMiSt (DNRF137). We are grateful to David Malloch for his valuable suggestions to improve the manuscript and together with Lothar Kriegsteiner for making specimens of *Dichlaena lentisci* available. This work was partially supported by the 10.13039/501100003549Hungarian Research Fund OTKA K115690 via Sándor Kocsubé. We thank Takashi Yaguchi and the CBS culture collection for supplying cultures for our study and Shaun Pennycook for his nomenclatural advice. Cony Decock is acknowledged for supplying literature of P. Biourge.

## Appendix A Supplementary data

Supplementary Fig. S1 Phylogenetic relationship within the *Eurotiales* based on a combined ML phylogeny using nine loci (*RPB1*, *RPB2*, *Cct8*, *Tsr1*, *CaM*, *BenA*, SSU, LSU, ITS) and a partition containing a binary matrix of indels present in the *Tsr1*, *CaM*, *BenA* and ITS dataset. Bootstrap values are above or below branches. The phylogram is based on 263 species belonging to the order *Eurotiales* and 16 species from the order *Onygenales* (used an outgroup). Supplementary Fig. S1

Supplementary Fig. S2 Combined phylogeny for *BenA*, *CaM* and *RPB2* data sets showing the phylogenetic relation of species within the genus *Hamigera*. The new combination *Hamigara brevicompacta* is shown in bold font. The BI posterior probability (pp) values and bootstrap percentages of the maximum likelihood (ML) analysis are presented at the nodes; fully supported branches are thickened. Values less than 70 % bootstrap support (ML) or less than 0.95 posterior probability (Bayesian analysis) are indicated with a hyphen or not shown. The bar indicates the number of substitutions per site. The phylogram is rooted with *Aspergillus glaucus*. Supplementary Fig. S2

Supplementary Fig. S3 Combined phylogeny for *BenA*, *CaM* and *RPB2* data sets showing the phylogenetic relation of species within the genus *Talaromyces*. The new combinations *Tal*. *resinae* and *Tal*. *striatoconidium* are shown in bold font. The BI posterior probability (pp) values and bootstrap percentages of the maximum likelihood (ML) analysis are presented at the nodes; fully supported branches are thickened. Values less than 70 % bootstrap support (ML) or less than 0.95 posterior probability (Bayesian analysis) are indicated with a hyphen or not shown. The bar indicates the number of substitutions per site. The phylogram is rooted with *Ascospirella lutea*. Supplementary Fig. S3

Supplementary Fig. S4 Phylogenetic trees based on *BenA*, *CaM* and *RPB2* sequence data sets showing the relationship between *Penicillium cellarum* and *P*. *aurantiogriseum*. The data of species mentioned in the phylogram can be found in the “accepted species” list in this article. The BI posterior probability (pp) values and bootstrap percentages of the maximum likelihood (ML) analysis are presented at the nodes; fully supported branches are thickened. Values less than 70 % bootstrap support (ML) or less than 0.95 posterior probability (Bayesian analysis) are indicated with a hyphen or not shown. The bar indicates the number of substitutions per site. The phylogram is rooted with *Penicillium chrysogenum* NRRL 20818. Supplementary Fig. S4
