# Supplementary figures and images for: Classification of Aspergillus, Penicillium, Talaromyces and related genera (Eurotiales): An overview of families, genera, subgenera, sections, series and species
Source: Stud Mycol. 2020 Jun 27;95:5–169. doi: 10.1016/j.simyco.2020.05.002 (PMC7426331; doi:10.1016/j.simyco.2020.05.002)

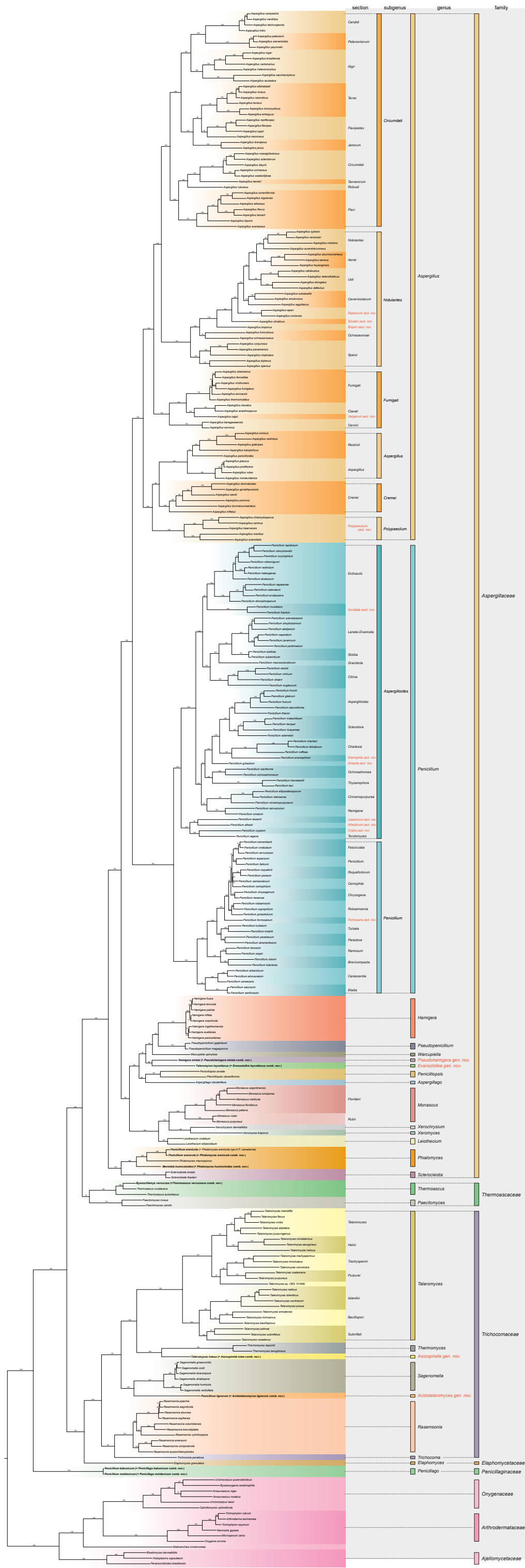

Supplement: Supplementary Fig. S1 — Phylogenetic relationship within the Eurotiales based on a combined ML phylogeny using nine loci (RPB1, RPB2, Cct8, Tsr1, CaM, BenA, SSU, LSU, ITS) and a partition containing a binary matrix of indels present in the Tsr1, CaM, BenA and ITS dataset. Bootstrap values are above or below branches. The phylogram is based on 263 species belonging to the order Eurotiales and 16 species from the order Onygenales (used an outgroup). [file mmc1.pdf]

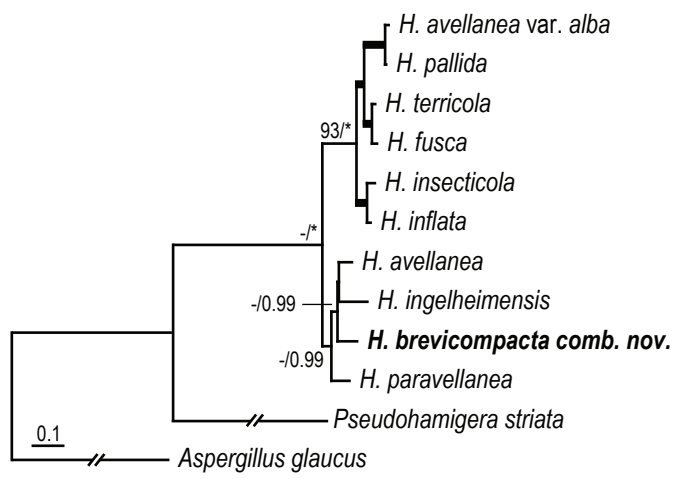

Supplement: Supplementary Fig. S2 — Combined phylogeny for BenA, CaM and RPB2 data sets showing the phylogenetic relation of species within the genus Hamigera. The new combination Hamigara brevicompacta is shown in bold font. The BI posterior probability (pp) values and bootstrap percentages of the maximum likelihood (ML) analysis are presented at the nodes; fully supported branches are thickened. Values less than 70 % bootstrap support (ML) or less than 0.95 posterior probability (Bayesian analysis) are indicated with a hyphen or not shown. The bar indicates the number of substitutions per site. The phylogram is rooted with Aspergillus glaucus. [file mmc2.pdf]

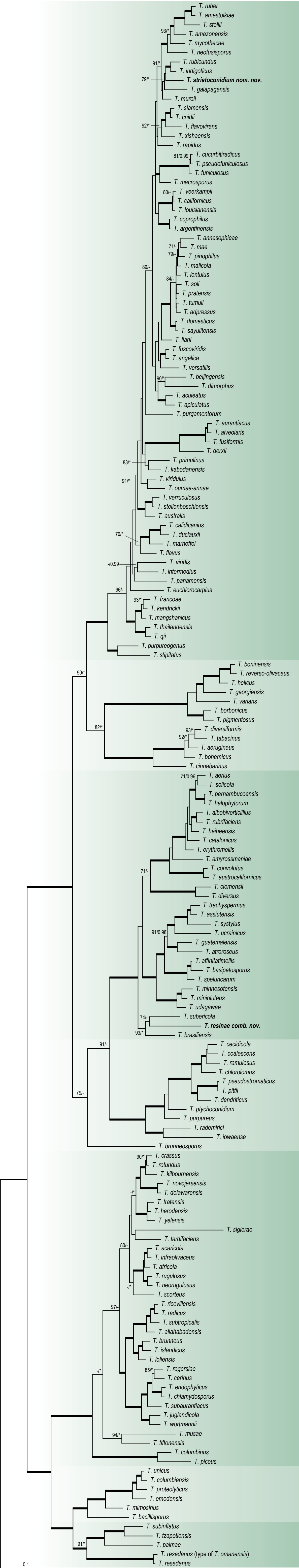

Supplement: Supplementary Fig. S3 — Combined phylogeny for BenA, CaM and RPB2 data sets showing the phylogenetic relation of species within the genus Talaromyces. The new combinations Tal. resinae and Tal. striatoconidium are shown in bold font. The BI posterior probability (pp) values and bootstrap percentages of the maximum likelihood (ML) analysis are presented at the nodes; fully supported branches are thickened. Values less than 70 % bootstrap support (ML) or less than 0.95 posterior probability (Bayesian analysis) are indicated with a hyphen or not shown. The bar indicates the number of substitutions per site. The phylogram is rooted with Ascospirella lutea. [file mmc3.pdf]

BenA

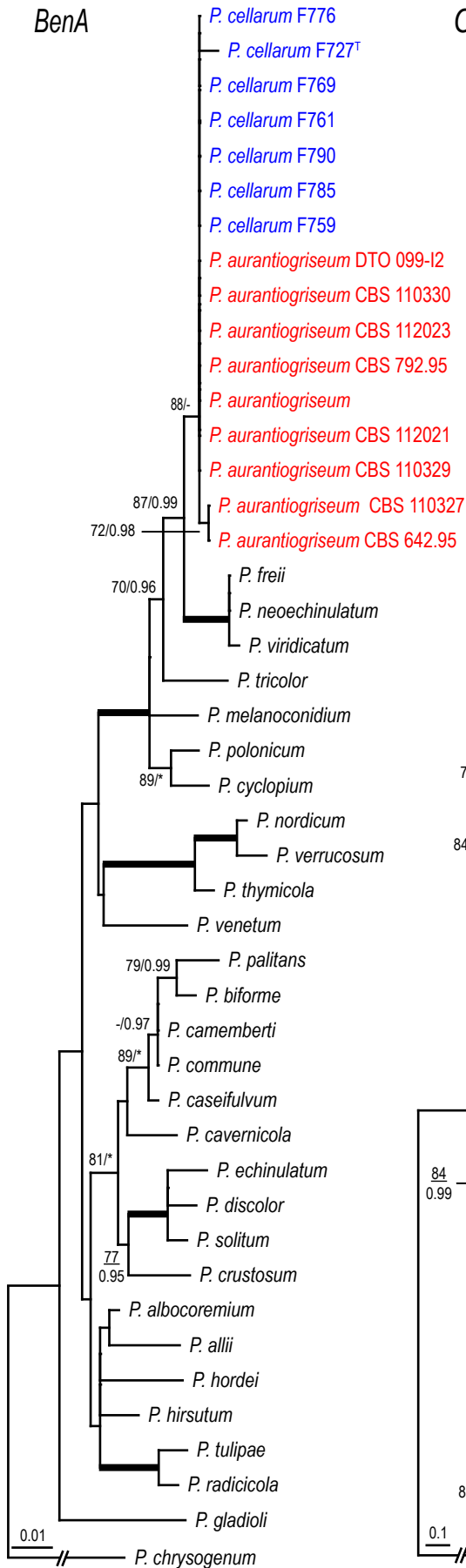

CaM

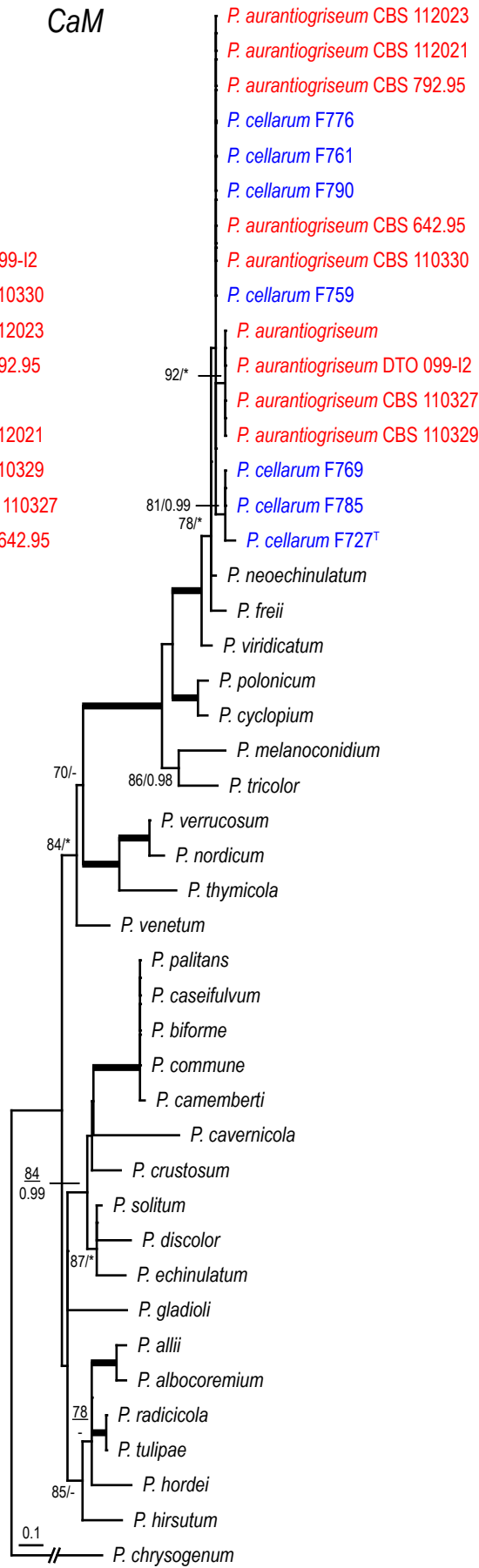

RPB2

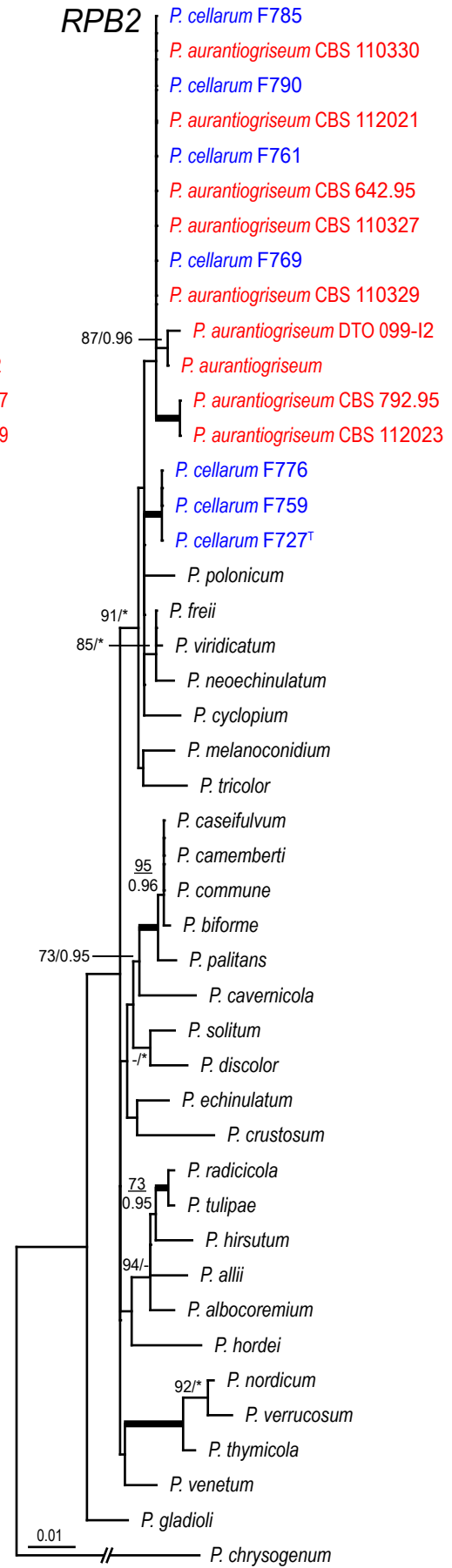

Supplement: Supplementary Fig. S4 — Phylogenetic trees based on BenA, CaM and RPB2 sequence data sets showing the relationship between Penicillium cellarum and P. aurantiogriseum. The data of species mentioned in the phylogram can be found in the “accepted species” list in this article. The BI posterior probability (pp) values and bootstrap percentages of the maximum likelihood (ML) analysis are presented at the nodes; fully supported branches are thickened. Values less than 70 % bootstrap support (ML) or less than 0.95 posterior probability (Bayesian analysis) are indicated with a hyphen or not shown. The bar indicates the number of substitutions per site. The phylogram is rooted with Penicillium chrysogenum NRRL 20818. [file mmc4.pdf]
